# Photocatalytic Annulation of Primary Amines to δ‑Lactams: Access to Privileged (Spiro)piperidine Space

**DOI:** 10.1021/acs.orglett.6c02237

**Published:** 2026-07-17

**Authors:** Jacob C. Turner-Dore, Joseph J. Bell-Tyrer, Hannah E. Askey, Darren L. Richards, Oleksandr Polishchuk, Oleksandr P. Datsenko, Jamie A. Cadge, Maria Ciaccia, Pavel K. Mykhailiuk, Alexander J. Cresswell

**Affiliations:** † Department of Chemistry, 1555 University of Bath, Claverton Down, Bath BA2 7AY, U.K.; ‡ Enamine Ltd., Winston Churchill Street 78, 02094 Kyiv, Ukraine; § Syngenta, Jealott’s Hill International Research Centre, Bracknell, Berkshire RG42 6EY, U.K.

## Abstract

Piperidines are the most prevalent saturated nitrogen heterocycles in pharmaceuticals, yet efficient and general methods for accessing α-disubstituted and spirocyclic variants have remained a long-standing synthetic challenge. A broadly applicable annulation strategy from unprotected primary alkylamines would represent an important advance, enabling access to privileged piperidine scaffolds from the most accessible nitrogen feedstocks. Here we disclose a general photoredox/hydrogen atom transfer dual catalytic annulation that converts primary amines into α-mono- and α-disubstituted δ-lactams, versatile intermediates that provide streamlined access to diverse piperidines. The method proceeds under mild conditions, can be scaled to ∼100 g in continuous flow, and affords products capable of downstream modification. This work provides a broadly applicable solution to a challenging problem in heterocycle synthesis, expanding the structural space accessible to drug discovery.

## Graphical abstract

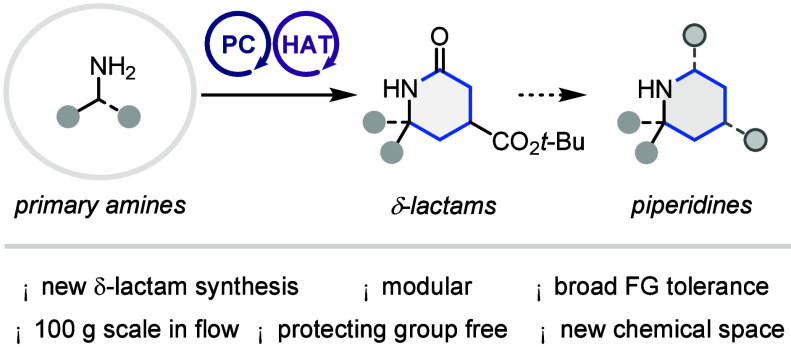

Piperidine rings are the most prevalent saturated nitrogen heterocycles in drug discovery,^2^ and substitution α to nitrogen, as well as at more remote positions, is a defining feature of numerous bioactive piperidines and alkaloids. Conventional strategies for the synthesis of α-substituted piperidines^3^ include pyridine reduction^4^ or dearomatization,^5^ cyclization via C–N or C–C bond formation,^6,7^ [4+2] cycloadditions of imines,^8,9^ double alkylation of primary amines,^10^ and peripheral functionalization of preassembled piperidine or δ-lactam scaffolds.^11^ Despite these advances, general and scalable routes to α-disubstituted and spirocyclic piperidines remain limited.^12^

We questioned whether such architectures could be accessed from abundant primary amines through an annulation process that simultaneously forges a C–C bond α to nitrogen^13^ and a C–N bond at the amine terminus (Figure 1A). Herein, we report a dual photoredox/hydrogen atom transfer (HAT) catalytic annulation of unprotected primary alkylamines **1** with itaconic acid-derived diester **2**, delivering α-(di)­substituted δ-lactams **3** (Figure 1B).^1^ These δ-lactams serve as versatile intermediates, undergoing lactam reduction and/or manipulation of the C(4) ester to furnish structurally diverse piperidines **4**. Collectively, this strategy establishes a new annulation-based retrosynthetic entry to α-substituted and spirocyclic piperidines from primary amines, proceeding through readily reduced δ-lactam intermediates and enabling substitution patterns that remain challenging to access using conventional approaches.

**Figure 1:**
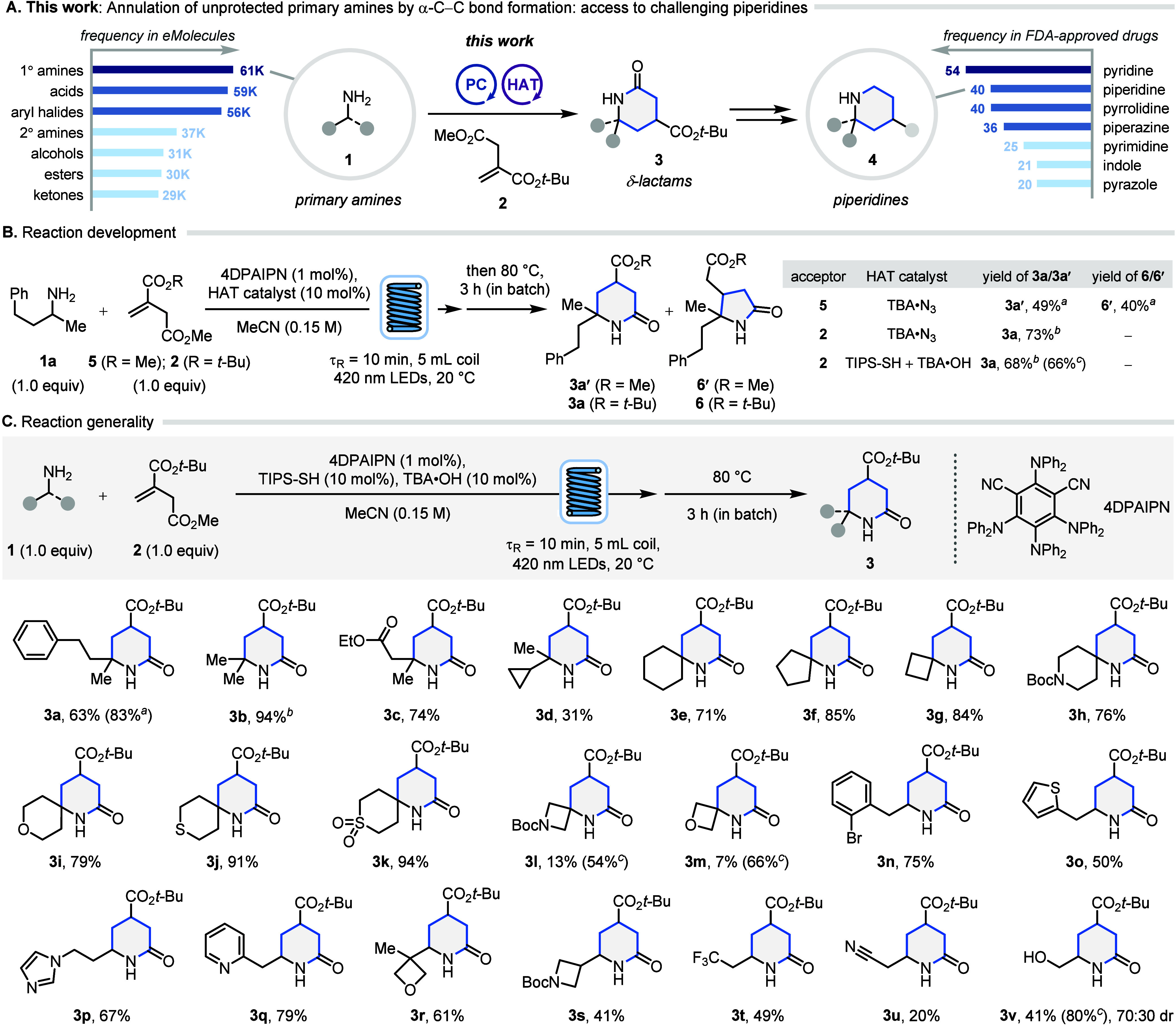
(A) This work. Annulation of unprotected primary amines by α-C–C bond formation, access to challenging piperidines. (B) Reaction development studies. ^*a*^Isolated yield. ^*b*^NMR yield against 1,3,5-trimethoxybenzene as an internal standard. ^*c*^NMR yield for the same reaction conducted in batch for 20 h with a 425 nm LED lamp. (C) Reaction generality. Unless otherwise stated, all compounds were obtained as ∼1:1 mixtures of diastereomers. ^*a*^With 1.2 equiv of acceptor **2**. ^*b*^With 2.0 equiv of amine used due to the volatility of **1b**. ^*c*^With 3.0 equiv of amine. Boc = *tert*-butoxycarbonyl.

We began our studies with 4-phenylbutan-2-amine (**1a**) and commercial dimethyl itaconate (**5**), using 4DPAIPN^14^ as the photocatalyst and TBA·N_3_ (TBA = tetrabutylammonium) as the HAT catalyst,^15^ in MeCN as the solvent. Irradiation at 420 nm in flow, followed by thermal lactamization, afforded a mixture of δ-lactam **3a′** and γ-lactam **6′** in 89% combined yield (55:45 ratio (Figure 1B)). To address this regioselectivity issue, we utilized sterically differentiated mixed itaconate ester **2**, readily prepared on a decagram scale from monomethyl itaconate.^16^ This reagent proved to be highly selective, delivering δ-lactam **3a** as a single regioisomer in 73% yield. Further evaluation revealed that triisopropylsilanethiolate (TIPS-S^–^), generated *in situ* from TIPS-SH, could substitute for azide as the HAT catalyst. Although yields with TIPS-S^–^ were slightly diminished (∼5% lower than with N_3_^–^), we adopted this system for the substrate scope to demonstrate that the annulation is not uniquely dependent on azide catalysis and to provide an effective alternative HAT catalyst for practitioners who prefer to avoid azide reagents. Finally, we confirmed that the transformation proceeds with comparable efficiency in batch (66% yield) and in continuous flow (68% yield), underscoring the robustness of the protocol.

With the optimized conditions established, we next explored the generality of the annulation with respect to the amine component (Figure 1C). Acyclic substrates performed well. Isopropylamine (**1b**) delivered *gem*-dimethylated lactam **3b** in 94% yield, while a γ-amino ester (**1c**) provided **3c** bearing two differentiated esters.

Cyclopropylamine (**1d**) gave **3d** in modest yield (33%), consistent with competitive β-scission of the α-amino radical, whereas larger cycloalkylamines such as cyclopentyl- and cyclohexylamine, as well as various heterocyclic derivatives, furnished spirocyclic lactams (**3e**, **3f**, and **3h**–**k**) in 71–94% yields. Smaller-ring partners showed mixed reactivity. Cyclobutylamine (**1g**) was competent (84%), but azetidine- and oxetane-containing substrates (**1l** and **1m**, respectively) required excess amine for good yields, reflecting the higher percent *s*-character of their α-C–H bonds.^15^ A particularly notable outcome was the good compatibility with α-monoalkylated amines (**1n**–**v**), and the lack of overalkylation observed. Ethanolamine (**1v**) proved to be less reactive, likely due to intramolecular hydrogen bonding,^17^ but yields improved dramatically (41% → 80%) with increased amine loading.

The reaction displayed broad functional group tolerance, accommodating esters, carbamates, ethers, thioethers, sulfones, cyano, silyl, hydroxyl, and acetal substituents as well as diverse heterocycles, including imidazoles, pyridines, thiophenes, oxetanes, and azetidines. Together, these results highlight the wide structural latitude of the annulation, spanning acyclic, cyclic, spirocyclic, and heterocyclic amines, and demonstrate the robustness of the protocol across the pharmaceutically relevant chemical space.

To further quantify the diversity of the scope, we compared the starting amines used with the Enamine library of primary amines (>59K compounds).^18^ The structures of the amines act as a surrogate to assess the diversity of the δ-lactams obtained. The properties for the amine library were obtained using a combination of Morgan fingerprints (1024-bit) and ADME-type features using RDKit.^19^ The feature space was reduced to two dimensions using the UMAP algorithm^20^ for visualization. The amine surrogates of scope compounds **3** are plotted in this resultant chemical space map (Figure 2A). Overall, the compounds selected showed a good spread across the UMAP space, indicating a high degree of structural diversity. Furthermore, the Vendi score was calculated for the scope to quantify this observed structural diversity. The Vendi score, recently used for the assessment of diversity of the training set for machine learning models,^21^ was calculated using the Tanimoto similarity matrix from 1024-bit Morgan fingerprints. A score of 19 was determined, indicating 19 unique structures of 26 amines in the substrate scope.

**Figure 2:**
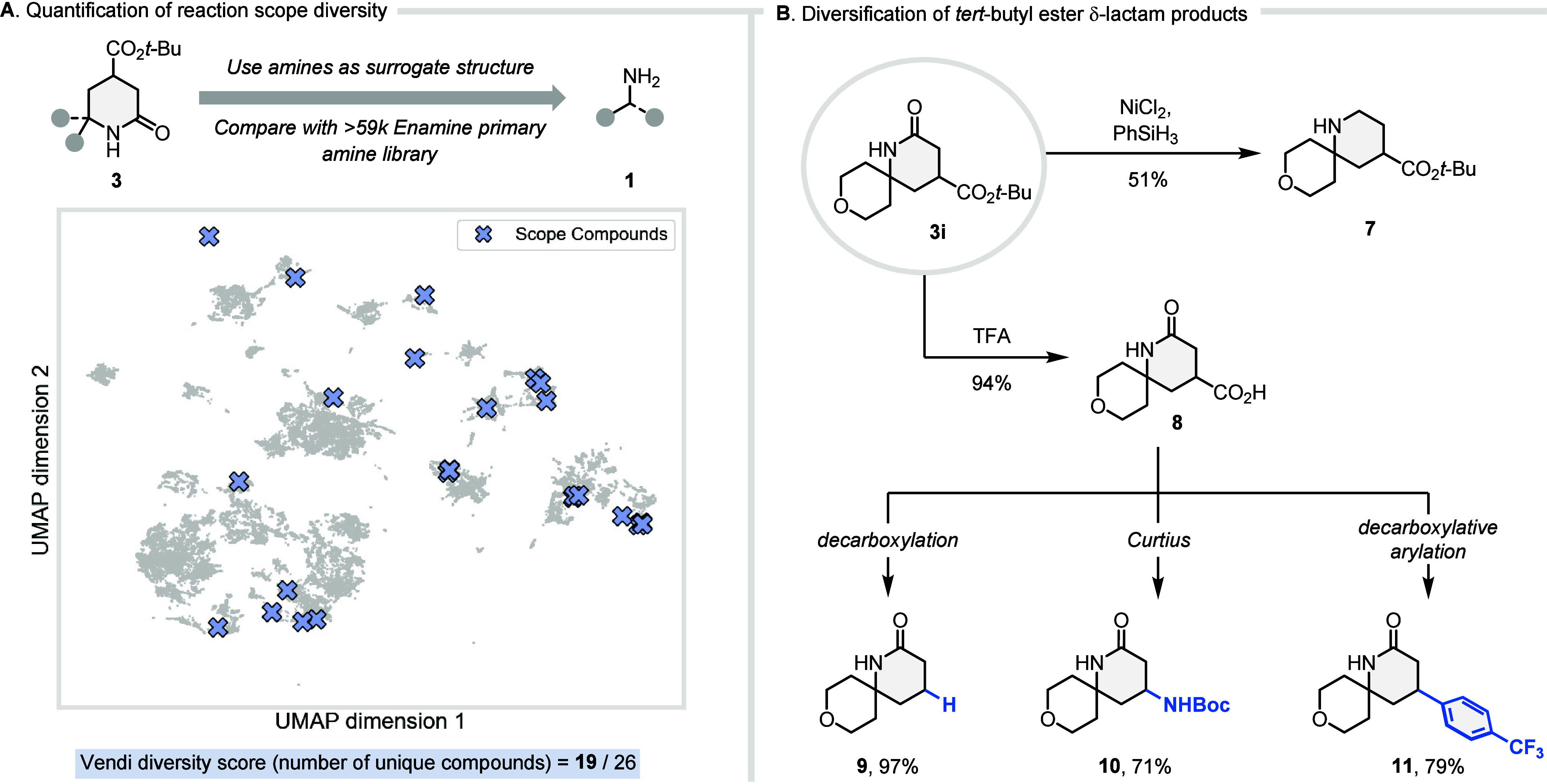
(A) Quantification of reaction scope diversity, compared to the Enamine library of >59K primary amines. (B) Diversification of *tert*-butyl ester δ-lactam products.

The synthetic potential of this methodology was next demonstrated through several representative transformations of δ-lactam *tert*-butyl ester **3i** (Figure 2B). Chemoselective reduction of the lactam provided piperidine scaffold **7**,^22^ while hydrolysis of the *tert*-butyl ester afforded free acid **8**. From this intermediate, persulfate-mediated decarboxylation delivered **9**,^23^ Curtius rearrangement enabled access to C(4)-amino derivative **10**, and photocatalytic decarboxylative arylation furnished γ-aryl lactam **11**.^24^

To evaluate scalability, large-scale flow reactions were conducted in collaboration with Enamine Ltd. (Figure 3A). Using isopropylamine **1a** (30 g) and cyclobutylamine **1g** (50 g), δ-lactams **13** and **14** were obtained in 58 and 94 g quantities, respectively. Operational modifications were implemented to accommodate scale-up considerations, including the use of cheaper NaN_3_ as the HAT catalyst, adoption of DMF as the solvent, and substitution of 3CzClIPN for 4DPAIPN. The latter change was made solely because 3CzClIPN was available from existing in-house stocks at Enamine Ltd. Notably, benzyl itaconate **12** proved to be an effective alternative to *tert*-butyl ester acceptor **2**, revealing that the bulky *tert*-butyl group is not essential for regiocontrol during lactamization. These results highlight the adaptability of the method and its promise for pharmaceutical process development.

**Figure 3:**
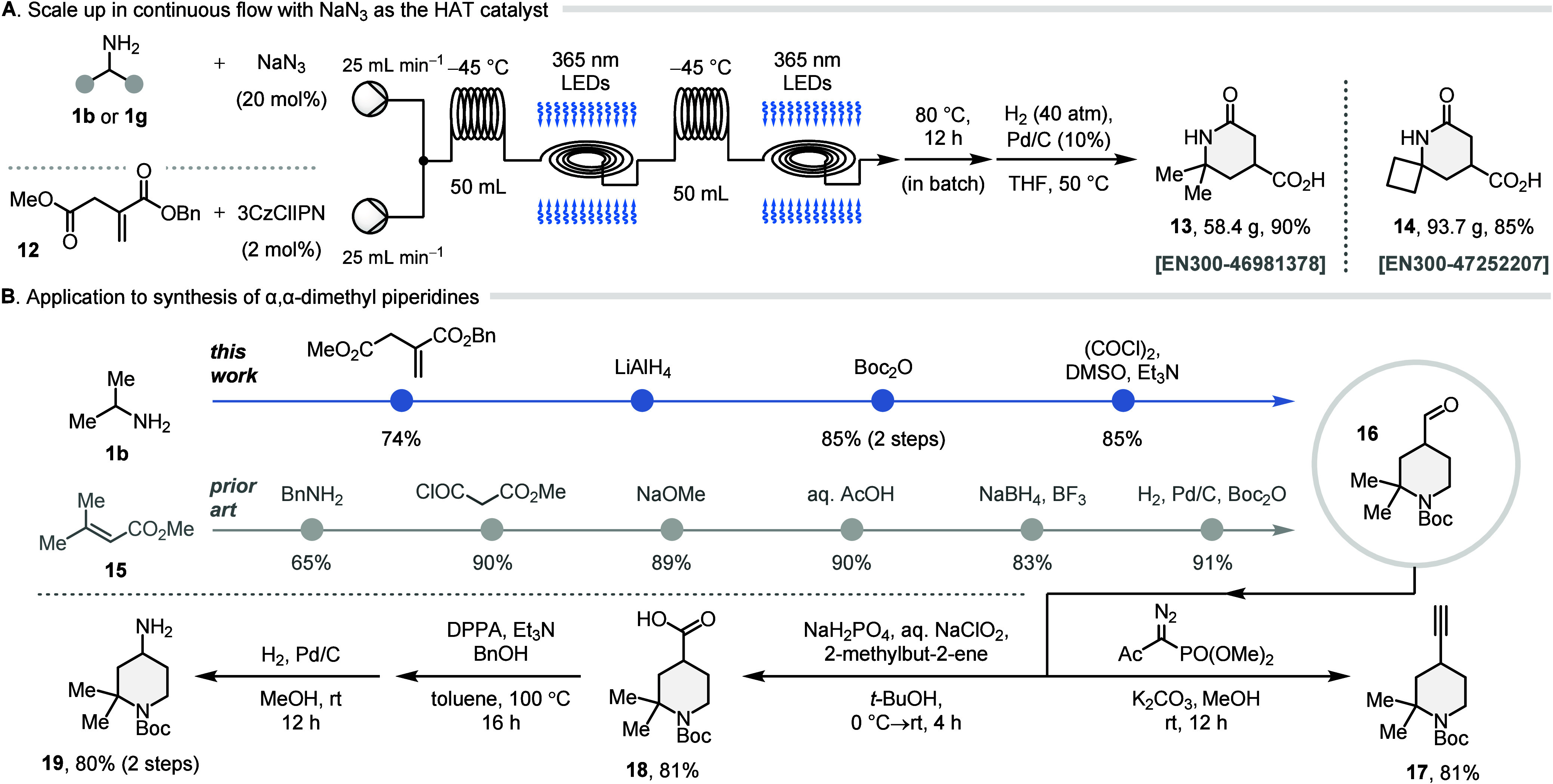
(A) Scale-up in continuous flow with NaN_3_ as the HAT catalyst. (B) Application to the synthesis of α,α-dimethylpiperidines.

To highlight the broad utility of this methodology for the concise synthesis of α-disubstituted piperidines, we next performed a gram-scale preparation of *N*-Boc 4-formyl-2,2-dimethylpiperidine **16** from isopropylamine **1b** (four steps, 53% yield) (Figure 3B). By comparison, the current state-of-the-art synthesis of **16** requires a six-step sequence (35% yield) from enoate **15**.^25^ The versatility of **16** was further demonstrated through elaboration to 4-substituted 2,2-dimethylpiperidines **17**–**19** via the Ohira–Bestmann reaction, Pinnick oxidation, and Curtius rearrangement, respectively.

In summary, we have developed a dual photoredox/HAT catalytic annulation of unprotected primary alkylamines with itaconate-derived acceptors, delivering α-mono- and α-disubstituted δ-lactams as versatile intermediates. These products provide streamlined entry to diverse (spiro)­piperidines, including substitution patterns that remain challenging by conventional methods. The reaction proceeds under mild conditions, is readily executed in continuous flow on up to 100 g scale, and displays a broad substrate scope and functional group tolerance.

Beyond establishing a scalable annulation strategy from abundant amine feedstocks, this work positions δ-lactams as practical gateways to privileged piperidine architectures central to drug discovery. The combination of generality, operational simplicity, and downstream diversification potential underscores the utility of this approach as a platform for medicinal chemistry and heterocycle synthesis. More broadly, this work illustrates how photocatalysis can enable direct annulations of simple feedstock amines into complex heterocyclic architectures.

## Supplementary Material



## Data Availability

The data underlying this study are available in the published article and its Supporting Information.

## Notes

All experimental procedures and compound characterization, including ^1^H and ^13^C NMR spectra (PDF)
