# Supplementary material for: Photocatalytic Annulation of Primary Amines to δ‑Lactams: Access to Privileged (Spiro)piperidine Space
Source: Org Lett. 2026 Jul 17;28(30):9603–8. doi: 10.1021/acs.orglett.6c02237 (PMC13435316; doi:10.1021/acs.orglett.6c02237)

# Photocatalytic Annulation of Primary Amines to δ-Lactams: Access to Privileged (Spiro)Piperidine Space

Jacob C. Turner-Dore,[1] Joseph J. Bell-Tyrer,[1] Hannah E. Askey,[1] Darren L. Richards,[1] Oleksandr Polishchuk,[2] Oleksandr Datsenko,[2] Jamie A. Cadge,[1] Maria Ciaccia,[3] Pavel K. Mykhailiuk,*[2] Alexander J. Cresswell*[1]

[1]Department of Chemistry, University of Bath, Claverton Down, Bath, BA2 7AY, U.K., E-mail: a.j.cresswell@bath.ac.uk.
[2] Enamine Ltd, Winston Churchill St. 78, 02094 Kyiv, Ukraine
[3] Syngenta, Jealott's Hill International Research Centre, Bracknell, Berkshire, RG42 6EY, U.K.

**SUPPLEMENTARY INFORMATION: TABLE OF CONTENTS**

**A**. General Experimental *S2*
**B**. General Procedures *S6*
**C**. Reaction Optimisation *S7*
**D**. Preparation of Starting Materials *S9*
**E**. Reaction Generality *S11*
**F**. Assessment of Scope Diversity *S42*
**G**. Large-Scale Synthesis *S47*
**H**. Product Derivatisations *S57*
**I.** Supplementary References *S74*
**J.** $^1$H and $^{13}$C NMR Spectra *S75*

## A. General Experimental

***General Setup***: Procedures employing oxygen- and/or moisture-sensitive materials were performed with anhydrous solvents (*vide infra*) using standard inert atmosphere techniques (atmosphere of anhydrous nitrogen). Where stated, purge box work was undertaken in an MBRAUN acrylic purge box, which was purged with anhydrous nitrogen for 20 min before use. Room temperature (rt) typically ranged between 20–25 °C.

***Batch Photochemistry***: All batch photoreactions were conducted in commercially available EvoluChem PhotoRedOx Box reactors purchased from HepatoChem Inc. (100 Cummings Center, Suite 451C, Beverly, MA 01915 USA). EvoluChem 18 W LED lamps (425 nm or 365 nm, as appropriate) were used. Temperature was maintained at rt (or close to rt) using a 12 V DC axial fan (40 × 40 × 10 mm, 1.2W, 11.4 $m^3/h$) and light leakage was contained using aluminium foil. Vial holders were 3D-printed out of Tough PLA (purchased from RS components) to fit 18-mL scintillation vials (36 mm OD).

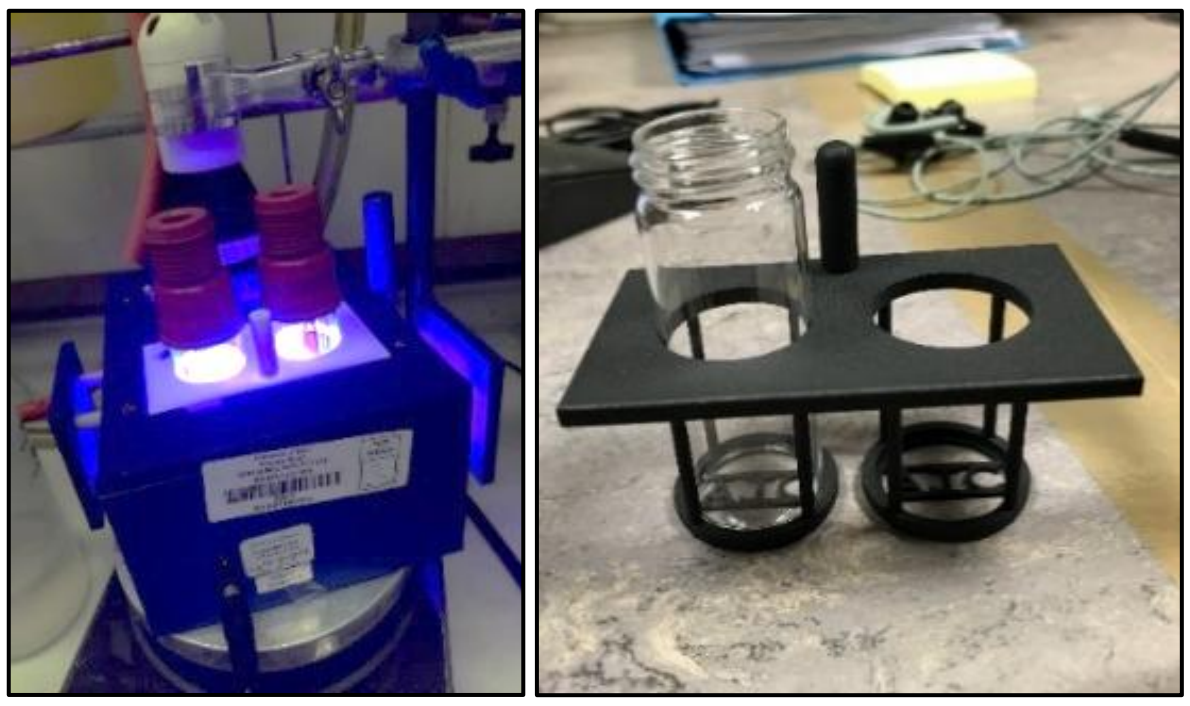

**Figure S1.** Left: EvoluChem PhotoRedOx Box equipped with 425 nm lamp.
Right: 3D-printed vial holder (PLA, for 18-mL scintillation vial).

***Vapourtec R-Series Flow Chemistry System***: Flow reactions were conducted on a fully automated Vapourtec RS-400 system comprising an R2C+ pump module (piston pumps), an R2S+ pump module (peristaltic pumps), an R4 reactor module, a GX-271 autosampler with GSIOC interface (3 × 204 sample racks, up to 27 vials each), and a touchscreen interface. Vials used on the autosampler were Wheaton™ 20-mL glass liquid scintillation vials (Fisher, Cat. No. 10698822) equipped with Wheaton™ white polypropylene open-top caps with PTFE faced silicone liners (Fisher, Cat. No. 10119922). System pressure was regulated using commercial Vapourtec acid-resistant BPRs (which were calibrated before use). A constant pressure of nitrogen [1.5 bar(g)] from a gas cylinder was regulated by an internal inert gas manifold to provide a constant pressure of 50 mbar(g) within reagent/solvent vessels.

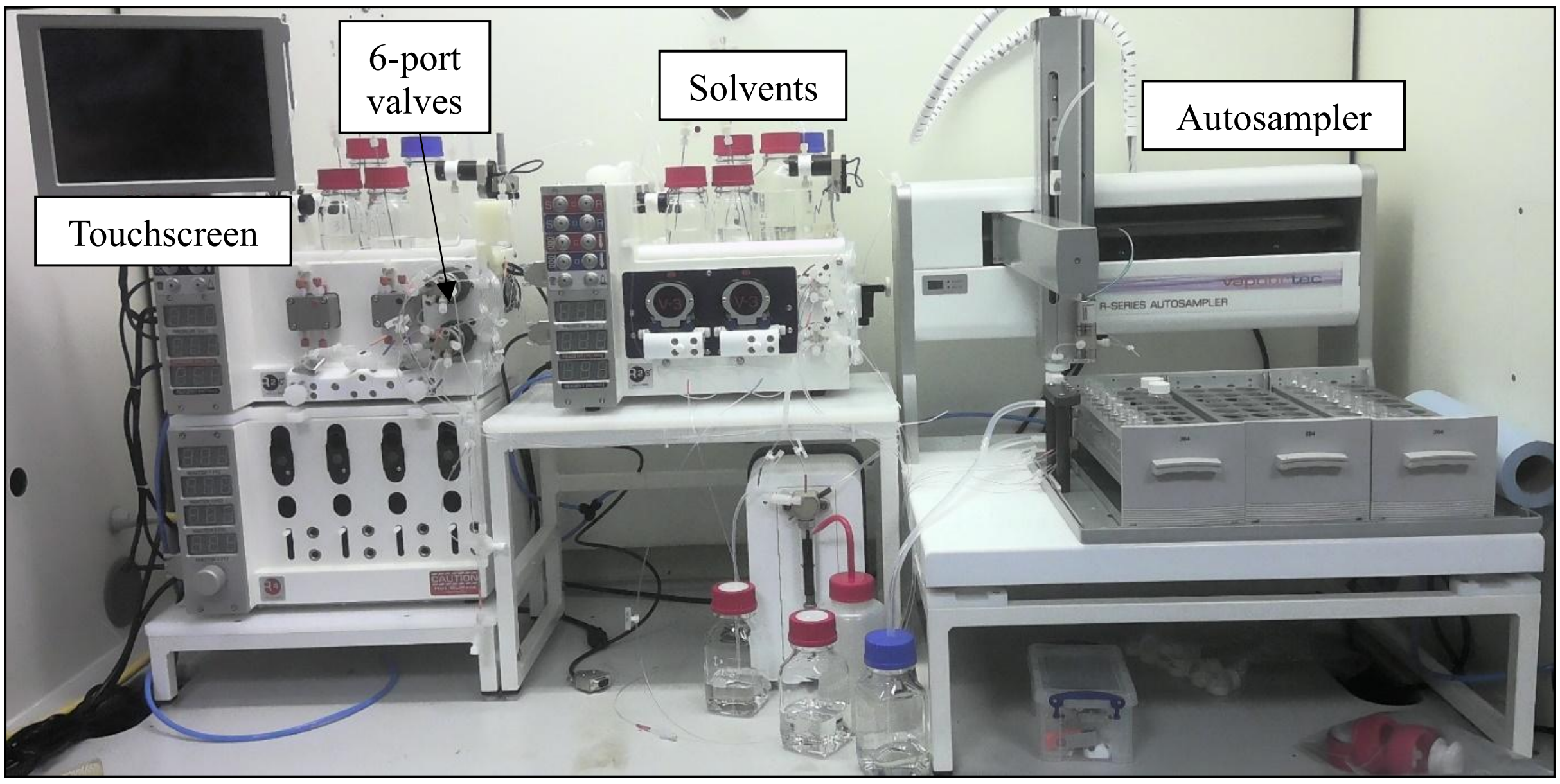


**Figure S2.** Vapourtec R-Series flow chemistry system

***Uniqsis PhotoSyn HP LED Photoreactor***: Flow photochemical reactions were conducted using an Uniqsis PhotoSyn HP LED photoreactor, equipped with a high-power LED array (716 individual diodes) emitting at 420 nm. The reactor consumes ~600 W at 100% output (1400 mA) and delivers a total optical output of up to ~350 W at 420 nm. It features a coil of perfluoroalkoxy polymer (PFA) tubing (5 mL volume, 1.0 mm ID, unless otherwise specified) mounted on an Uniqsis Cold Coil module. The LED array was cooled to +0.3 °C using a Julabo FL1203 recirculating chiller, whilst the Cold Coil module was cooled to 0 °C using a Huber CC-805 recirculating chiller. The internal volume of the PhotoSyn was continuously flushed with anhydrous nitrogen during the reaction (and for 5 min prior to initiating cooling) to prevent condensation/icing.

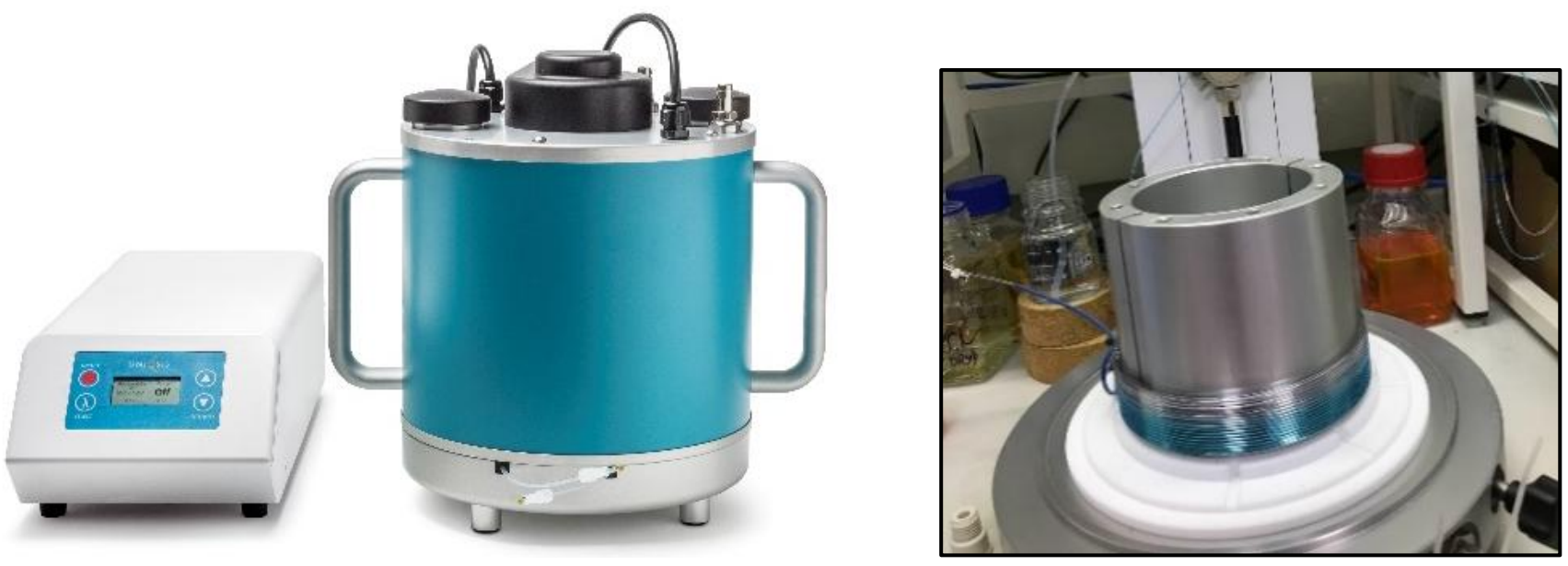

**Figure S3.** Left: Uniqsis PhotoSyn HP LED photoreactor. Right: Uniqsis Cold Coil module.

***Solvent Evaporation***: Solvent evaporation was carried out using either a Biotage V-10 evaporator, an Asynt Spiral Evaporator, or a Genevac EZ-2 Plus.

***NMR Spectroscopy***: $^{1}$H, $^{13}$C{$^{1}$H}, and $^{19}$F{H} spectra were recorded at 400/500 MHz, 101/126 MHz, 376/471 MHz, respectively, using a Bruker Avance NEO spectrometer (400 MHz, equipped with an iProbe SmartProbe 400), a Bruker Avance NEO spectrometer (500 MHz, equipped with an iProbe BBO 500), or an Agilent ProPulse spectrometer (500 MHz, equipped with a OneNMR probe). Spectra were

recorded at 298 K (25 °C), unless otherwise stated. $^{1}H$ and $^{13}C\{^{1}H\}$ NMR spectra were referenced to residual solvent peaks; chemical shifts are reported in parts per million (ppm) relative to residual chloroform (δ = 7.26 ppm, $^{1}H$; 77.16 ppm, $^{13}C$) or acetic acid (δ = 2.04 ppm, $^{1}H$; 178.99 ppm, $^{13}C$). $^{19}F\{H\}$ spectra were referenced against fluorobenzene (δ = –113.15 ppm, $^{19}F$). All $^{13}C\{^{1}H\}$ resonances are assumed to be singlets, unless stated otherwise. Coupling constants, *J*, reported in Hertz (Hz), were calculated using Mestrenova 14 to the nearest 0.1 Hz. The following abbreviations (and their combinations) are used to label the multiplicities: br (broad), s (singlet), d (doublet), t (triplet), q (quartet), (p) pentet, sept (septet), and m (multiplet). $^{1}H$ and $^{13}C\{^{1}H\}$ assignments for novel compounds are corroborated through 2D (COSY, HSQC, HMBC).

***Infrared Spectroscopy***: Infrared (IR) spectra of neat compounds were recorded over the range 4000–650 $cm^{-1}$ using a PerkinElmer Spectrum 100 ATR-FTIR spectrometer. Peaks are reported in $cm^{-1}$ with indicated relative intensities: s (strong, 0–33% T); m (medium, 34–66% T), w (weak, 67–100% T), and br (broad).

***Mass Spectrometry***: Electrospray ionisation (ESI) spectra were recorded on either an Agilent Electrospray Quadrupole Time-of-Flight mass spectrometer (ESI/QTOF) or a Bruker maXis HD High Res QTOF mass spectrometer (ESI/QTOF, maXis). Samples were analysed by direct injection and data are reported in the form of m/z (intensity relative to the base peak = 100).

***Melting Points***: Uncorrected melting points (mp) were determined on either a Stuart SMP10 capillary melting point apparatus or a Stanford Research Systems OptiMelt automated capillary melting point apparatus at 2 °C $min^{-1}$ in open capillary tubes.

***Thin Layer Chromatography***: Analytical thin-layer chromatography was performed on Merck silica gel 60 $F_{254}$ aluminium-backed plates. Visualisation was accomplished with UV light (254 nm), iodine ($I_2$) on silica, aqueous basic potassium permanganate ($KMnO_4$), phosphomolybdic acid (PMA), and/or bromocresol green solutions.

***Flash Column Chromatography***: Flash column chromatography was performed on either Teledyne Isco RediSep Bronze® silica columns or RediSep Gold® $C_{18}$ columns using a CombiFlash NextGen 300 (or 300+) system equipped with a UV detector. Samples were dry-loaded from Celite®. Manual flash column chromatography was performed using high-purity grade silica gel, pore size 60 Å, 200–400 mesh particle size (Sigma-Aldrich, cat. no. 288594).

***Solvents***: Reaction solvent MeCN was dried by percolation through a column packed with neutral alumina under a positive pressure of $N_2$. *t*-BuOH was distilled onto activated 3 Å molecular sieves before use. All other solvents, including those used for filtration, transfers, chromatography, and recrystallization, were used as received (unless otherwise stated). HPLC-grade $H_2O$, MeCN, and MeOH were used for reversed phase chromatography. Where stated, solvents containing $NH_4OH$ were prepared using aqueous ammonia (28–30%), purchased from Merck.

***Tetrabutylammonium Azide (TBA•N$_3$)***: TBA•$N_3$ (CAS# 993-22-6) is extremely hygroscopic and best handled under inert atmosphere. It can be conveniently prepared *in situ* by stirring TBA•Cl with $NaN_3$ in MeCN, followed by filtration of NaCl. We found that the commercial material (FluoroChem or TCI) can be contaminated with NaCl, such that dissolution in MeCN is visibly incomplete, leading to a slightly cloudy solution.. Upon standing, the NaCl residues will settle, and the clear supernatant solution can be used. Whilst TBA•$N_3$ should be treated with the same precautions taken for sodium azide ($NaN_3$)[1] (i.e., not ingested/inhaled, not exposed to acids or chlorinated solvents, not stored in metal containers), it has no impact sensitivity and a decomposition temperature of 196 °C.[2] TBA•$N_3$ or its solutions should never be directly disposed of down the drain, where highly sensitive metal azide crystals could slowly accumulate on metal plumbing (plus azide is also highly toxic to aquatic life). Adequate precautions are necessary for the safe and environmentally responsible disposal of azide solution residues. Small amounts of azide-containing waste and azide dust can be decomposed harmlessly with ceric ammonium nitrate. Larger quantities must be destroyed by addition of a sodium nitrite solution followed by a mineral acid solution.

***Other Chemicals***: Commercial chemicals were purchased from one of the following suppliers: Merck, Fisher Scientific, ThermoFisher, VWR, TCI, Apollo, Enamine, Doug Discovery (formerly Fluorochem). Primary amines were purified by distillation, aqueous work up or column chromatography before use. The following amines were purchased as HCl salts: 4-aminotetrahydro-2*H*-thiopyran 1,1-dioxide, (3-methyloxetan-3-yl)methanamine, 3,3,3-trifluoropropanamine; in these cases, the free amine was obtained as follows: the amine hydrochloride (~0.5 g) was dissolved in 2.5 M aq. KOH (10 mL) and was extracted with $CH_2Cl_2$ (3 × 10 mL). The combined organic extracts were washed with brine (5 mL), then dried ($Na_2SO_4$), filtered, and gently concentrated *in vacuo* to give the corresponding free amine. TBAOH•30$H_2O$ was titrated according to the following procedure: a solution of TBAOH•30$H_2O$ (400 mg) in $H_2O$ (50 mL, *total solution volume*) in a 50-mL volumetric flask was titrated against 0.020 M aq. HCl with phenolphthalein indicator; this procedure was repeated in triplicate to give the hydroxide concentration as 9.95 mM (1.24 mmol $g^{-1}$). All other chemicals purchased from commercial suppliers were used as received.

## B. General Procedures

***General Procedure 1 for δ-lactam formation from primary amines***

A Vapourtec RS-400 flow chemistry system was configured as indicated in **Figure S4**, with a Uniqsis PhotoSyn HP LED photoreactor equipped with 420 nm LEDs. Pumps A and B were V-3 peristaltic pumps (equipped with blue pump tubes), whereas pump B1 was a syringe pump associated with the liquid handler. All solvent lines for pumps A, B, A1, and B1 were fed from a reservoir of MeCN. Reagent feeds A and B were simultaneously injected into the 5.0 mL coil of the Uniqsis PhotoSyn HP LED photoreactor (3.00 mL aliquots, flow rate = 0.25 mL min$^{-1}$) to give an overall flow rate of 0.50 mL min$^{-1}$ and a $t_R$ = 10 min. Based on the prediction from Vapourtec's proprietary dispersion model, a 3.00 mL aliquot was collected from the predicted steady-state product mixture, corresponding to a theoretical maximum yield of product of 0.45 mmol. Following collection, the 3.00 mL aliquot of reaction mixture was transferred to a 5-mL, crimp-sealed microwave vial equipped with a stirrer bar and stirred at 80 °C for 3 h, followed by concentration *in vacuo*.

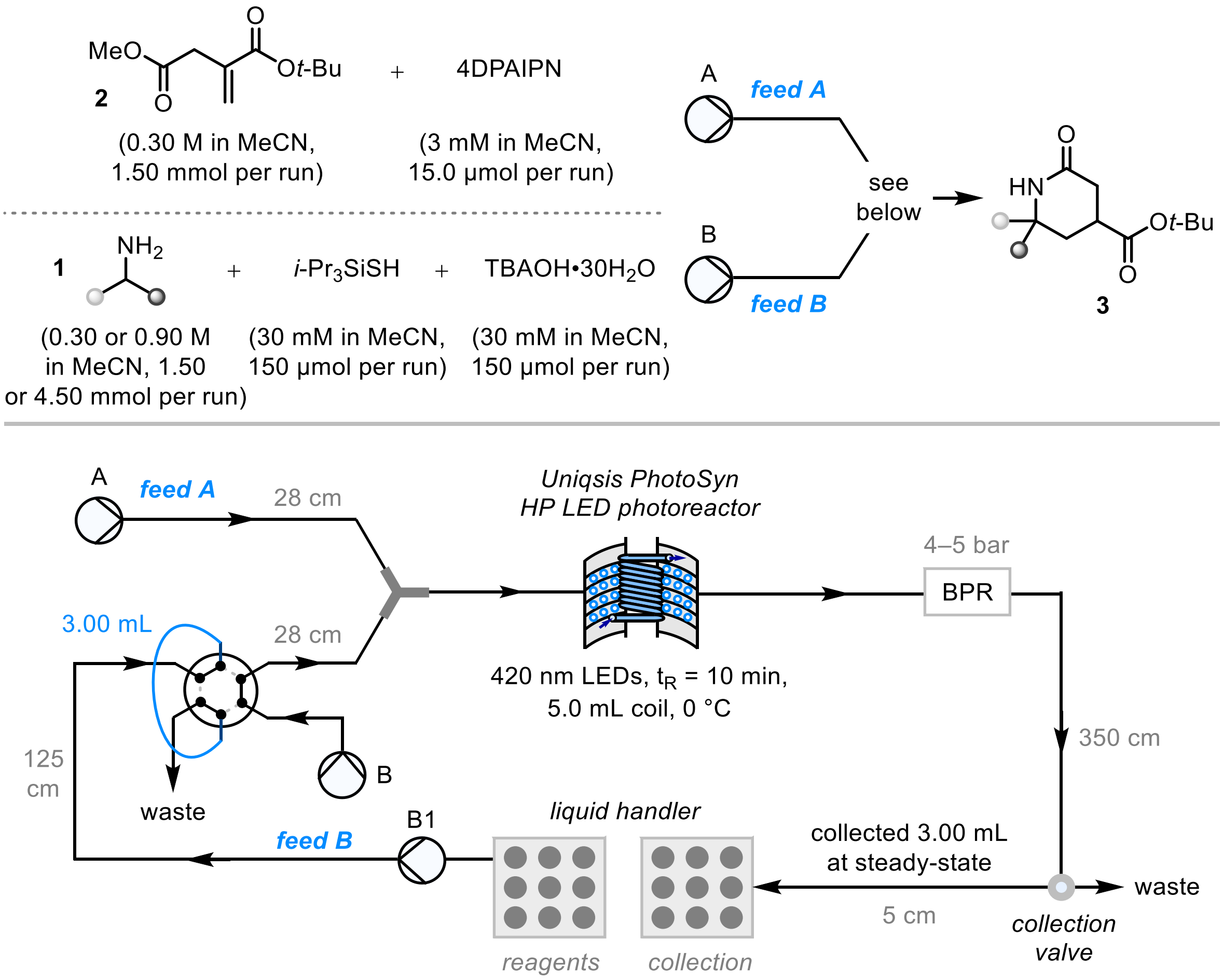


**Figure S4.**

## C. Reaction Optimisation

**Methyl 2-methyl-6-oxo-2-phenethylpiperidine-4-carboxylate (3a′) and methyl 2-(2-methyl-5-oxo-2-phenethylpyrrolidin-3-yl)acetate (6′)**

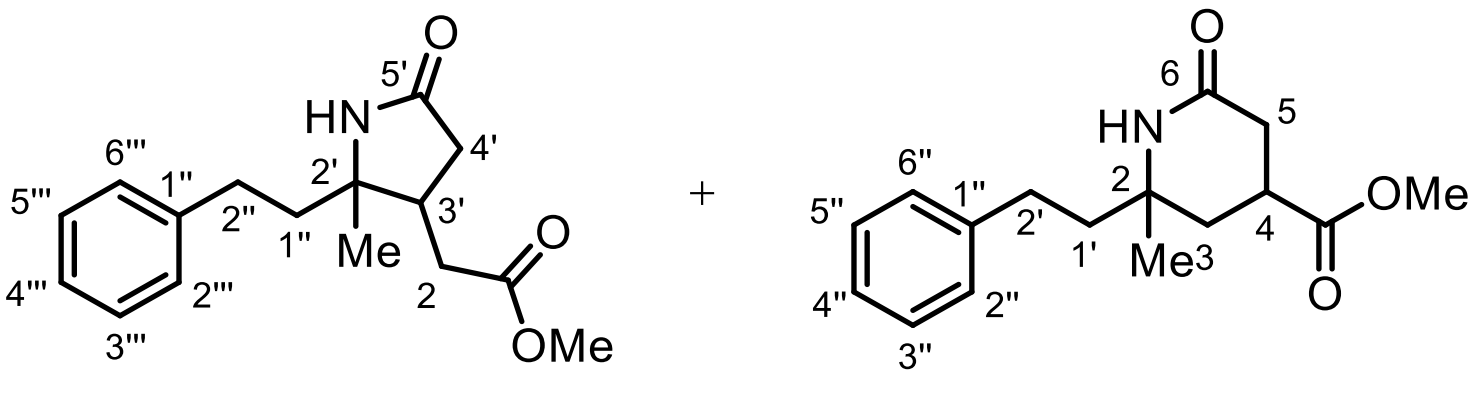


**6′** **3a′**

Following a modification of ***General Procedure 1*** (with TBA•N$_3$ in place of *i*-Pr$_3$SiSH + TBAOH•30H$_2$O, dimethyl itaconate **5** in place of **2**, and post-flow heating at 80 °C for 17 h), 4-phenylbutan-2-amine **1a** (241 µL, 1.50 mmol, 1.0 equiv), dimethyl itaconate **5** (237 mg, 1.50 mmol, 1.0 equiv), 4DPAIPN (12.4 mg, 15 µmol, 1 mol%), and TBA•N$_3$ (43 mg, 0.15 mmol, 10 mol%) were reacted. Note that the ratio of **6′**:**3a′** in the crude product mixture could not be determined by $^1$H NMR spectroscopy, due to signal overlap. Purification *via* flash column chromatography on silica gel (12 g) in CH$_2$Cl$_2$/MeOH, 0% (5 CV) then 0→5% (20 CV) then 5→10% (5 CV) then 10% (5 CV) then 15% (5 CV), followed by reversed-phase flash column chromatography on C$_{18}$ silica gel (15.5 g) in H$_2$O/MeOH 2% (2 CV) then 2→100% (28 CV) then 100% (7 CV), gave **6′** as a colourless solid (40.4 mg, 28%, 55:45 dr) and **3a′** as a colourless solid (17.5 mg, 12%, 67:33 dr). [N.B. Assignments of **6′** and **3a′** were secured based on HMBC, HSQC, and COSY correlations to the methine groups].

Data for **3a′**: (Peaks attributed to a diastereomer are denoted by *)

$^1$H NMR: (400 MHz, CDCl$_3$)

7.32–7.24 (m, 4H, C(3″)*H*, C(5″)*H*, C(3″)*H*$^*$, C(5″)*H*$^*$), 7.22–7.13 (m, 6H, C(2″)*H*, C(4″)*H*, C(6″)*H*, C(2″)*H*$^*$, C(4″)*H*$^*$, C(6″)*H*$^*$), 6.11 (br s, 1H, N*H*), 6.05 (br s, 1H, N*H*$^*$), 3.69 (s, 6H, O*Me*, O*Me*$^*$), 3.06–2.87 (m, 4H, C(5)$H_A$, C(4)*H*, C(5)$H_A$$^*$, C(4)*H*$^*$), 2.71–2.55 (m, 4H, C(2′)$H_2$, C(2′)$H_2$$^*$), 2.46–2.37 (m, 2H, C(5)$H_B$, C(5)$H_B$$^*$), 2.36–2.19 (m, 2H, C(3)$H_A$, C(3)$H_A$$^*$), 1.85 (app t, $J$ = 12.0 Hz, 4H, C(1′)$H_2$, C(1′)$H_2$$^*$), 1.78–1.63 (m, 2H, C(3)$H_B$, C(3)$H_B$$^*$), 1.36 (s, 3H, C(2)*Me*), 1.33 (s, 3H, C(2)*Me*$^*$)

$^{13}$C NMR: (101 MHz, CDCl$_3$)

176.9 (*C*(O)O), 176.8 (*C*(O)O$^*$), 172.7 (*C*(6)), 172.6 (*C*(6)$^*$), 141.4 (*C*(1″)), 141.3 (*C*(1″)$^*$), 128.7 (*C*(3″), *C*(5″), *C*(3″)$^*$, *C*(5″)$^*$), 128.4 (*C*(2″), *C*(6″)), 128.3 (*C*(2″)$^*$, *C*(6″)$^*$), 126.3 (*C*(4″)), 126.2 (*C*(4″)$^*$), 57.2 (*C*(2)), 57.1 (*C*(2)$^*$), 51.9 (CO(O*Me*), 44.4 (*C*(1′)), 43.9 (*C*(1′)$^*$), 40.9 (*C*(3)), 40.6 (*C*(3)$^*$), 38.2 (*C*(4)), 37.6 (*C*(4)$^*$), 35.8 (*C*(5)), 35.4 (*C*(5)$^*$), 31.0 (*C*(2′)), 30.8 (*C*(2′)$^*$), 28.3 (C(2)*Me*), 26.7 (C(2)*Me*$^*$)

IR: (neat)

3855 (w), 3218 (w), 2953 (w), 1737 (w), 1694 (m), 1497 (w), 1437 (w), 1365 (w), 1259 (w), 1173 (w), 752 (w), 701 (w)

MS: (ESI$^+$/QTOF)

300 (3%), 299 (18%), 298 (100%), 276 (15%), 275 (3%), 259 (2%), 258 (1%), 257 (7%)

HRMS: (ESI$^+$/QTOF)

Calcd for $C_{16}H_{21}NO_3Na^+$ [M+Na]$^+$: 298.1414; found, 298.1414

Data for **6′**: (Peaks attributed to a diastereomer are denoted by *)

$^1$H NMR: (400 MHz, $CDCl_3$)

7.37–7.29 (m, 4H, C(3‴)*H*, C(5‴)*H*, C(3‴)*H*$^*$, C(5‴)*H*$^*$), 7.27–7.17 (m, 6H, C(2‴)*H*, C(4‴)*H*, C(6‴)*H*, C(2‴)*H*$^*$, C(4‴)*H*$^*$, C(6‴)*H*$^*$), 7.02 (br s, 1H, N*H*), 6.85 (br s, 1H, N*H*$^*$), 3.79 (s, 3H, O*Me*), 3.77 (s, 3H, O*Me*$^*$), 3.09–2.93 (m, 2H, C(3′)*H*, C(3′)*H*$^*$), 2.74–2.39 (m, 8H, C(2″)$H_2$, C(2)$H_2$, C(2″)$H_2{}^*$, C(2)$H_2{}^*$), 2.25–1.99 (m, 2H, C(4′)$H_A$, C(4′)$H_A{}^*$), 1.97–1.79 (m, 4H, C(1″)$H_2$, C(1″)$H_2{}^*$), 1.75 (app t, $J$ = 12.0 Hz, 2H, C(4′)$H_B$, C(4′)$H_B{}^*$), 1.40 (s, 3H, C(2′)*Me*), 1.37 (s, 3H, C(2′)*Me*$^*$)

$^{13}$C NMR: (101 MHz, $CDCl_3$)

174.1 (*C*(O)O), 174.0 (*C*(O)O$^*$), 170.5 (*C*(5′)), 170.3 (*C*(5′)$^*$), 141.5 (*C*(1‴)), 141.3 (*C*(1‴)$^*$), 128.6 (*C*(3‴), *C*(5‴), *C*(3‴)$^*$, *C*(5‴)$^*$), 128.4 (*C*(2‴), *C*(6‴)), 128.3 (*C*(2‴)$^*$, *C*(6‴)$^*$), 126.20 (*C*(4‴), 126.18 (*C*(4‴)$^*$), 54.6 (*C*(2′)), 54.2 (*C*(2′)$^*$), 52.3 (CO(O*Me*), 45.7 (*C*(1″)), 44.0 (*C*(1″)$^*$), 36.6 (*C*(4′)), 35.7 (*C*(4′)$^*$), 35.5 (*C*(3′)), 35.1 (*C*(3′)$^*$), 33.4 (*C*(2)), 32.9 (*C*(2)$^*$), 30.6 (*C*(2″)), 30.0 (*C*(2″)$^*$), 28.5 (C(2′)*Me*), 28.2 (C(2′)*Me*$^*$)

IR: (neat)

3737 (w), 3196 (w), 3030 (w), 2953 (w), 2363 (w), 1732 (m), 1655 (s), 1603 (w), 1496 (w), 1453 (w), 1436 (m), 1400 (w), 1262 (w), 1196 (m), 1171 (m), 1070 (w), 1016 (w), 913 (w), 753 (w), 700 (m)

MS: (ESI$^+$/QTOF)

300 (2%), 299 (18%), 298 (100%), 276 (23%), 275 (1%), 259 (1%), 257 (3%)

HRMS: (ESI$^+$/QTOF)

Calcd for $C_{16}H_{21}NO_3Na^+$ [M+Na]$^+$: 298.1414; found, 298.1413

## D. Preparation of Starting Materials

### 2,4,5,6-Tetrakis(diphenylamino)isophthalonitrile (4DPAIPN)

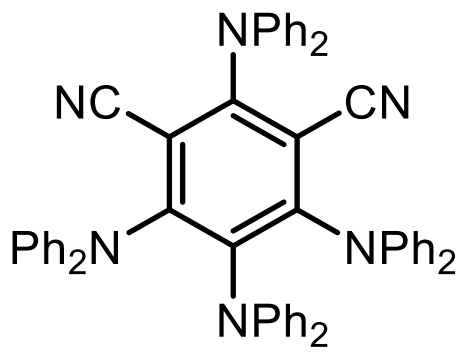

A flame-dried, nitrogen-flushed, 100-mL, 2-necked, round-bottomed flask equipped with a stirrer bar was charged with NaH (640 mg, 60% suspension in mineral oil, 16.0 mmol, 8.0 equiv) and anhydrous DMF (20 mL). Diphenylamine (2.03 g, 12.0 mmol, 6.0 equiv) was added portionwise under a stream of $N_2$, then the mixture was stirred at 50 °C for 1 h. Tetrafluoroisophthalonitrile (400 mg, 2.00 mmol, 1.0 equiv) was then added, and the mixture was stirred at 50 °C for a further 15 h. The mixture was cooled to 0 °C and pieces of ice were added to quench the reaction, followed by concentration *in vacuo*. The resultant residue was re-dissolved in $CH_2Cl_2$ and filtered through a Celite® pad, then the yellow-brown filtrate was purified *via* flash column chromatography on silica gel in 60% $CH_2Cl_2$ in 40–60 °C petroleum ether. The resultant orange solid was ground to a powder and dried under high vacuum overnight to give 4DPAIPN as a yellow solid (1.43 g, 89%). *$^{1}$H and $^{13}$C NMR spectroscopic data was in accordance with the literature*.[3]

Data for 4DPAIPN:

$^{1}$H NMR: (500 MHz, $CDCl_3$)

7.32–7.23 (m, 4H), 7.13–7.00 (m, 14H), 6.95–6.85 (m, 8H), 6.75–6.67 (m, 10H), 6.60–6.54 (m, 4H)

$^{13}$C NMR: (126 MHz, $CDCl_3$)

154.3, 151.9, 145.7, 144.8, 143.3, 140.5, 129.52, 128.7, 127.7, 124.3, 124.1, 123.1, 122.8, 122.7, 121.2, 113.3, 113.2

### 1-(*tert*-Butyl) 4-methyl 2-methylenesuccinate (2)

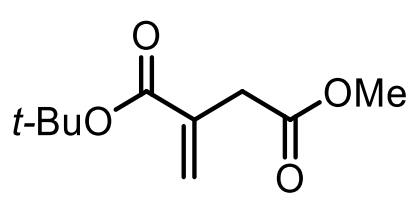

A nitrogen-flushed, 500-mL round-bottomed flask equipped with a stirrer bar was charged with 4-methoxy-2-methylene-4-oxobutanoic acid (5.77 g, 40.0 mmol, 1.0 equiv) and EtOAc (150 mL, dried over 4Å MS for 16 h). *tert*-Butyl 2,2,2-trichloroacetimidate (21.5 mL, 26.2 g, 120 mmol, 3.0 equiv) was then added and the mixture was cooled to 0 °C, followed by dropwise addition of $BF_3$•$OEt_2$ (507

μL, 568 mg, 4.00 mmol, 10 mol%) over 3 min. The mixture was allowed to warm to rt then stirred at rt for 18 h. The resultant mixture was quenched with $H_2O$ (5 mL) then washed with $H_2O$ (2 × 50 mL) and brine (40 mL). The organic phase was dried ($MgSO_4$), filtered, and concentrated *in vacuo* to give an colourless oily solid (29 g). Purification *via* flash column chromatography on silica gel (220 g, 6.5 × 16 cm) in 10% EtOAc in pentane (2 CV) then 15% EtOAc in pentane (2 CV) then 20% EtOAc in pentane (2 CV) gave **2** as a colourless oil (6.94 g, 87%). *$^{1}$H and $^{13}$C NMR spectroscopic data was in accordance with the literature*.[4]

Data for **2**:

Density: 1.04 g $mL^{-1}$

$^{1}$H NMR: (500 MHz, $CDCl_3$)

6.28–6.22 (m, 1H), 5.62–5.58 (m, 1H), 3.69 (s, 3H), 3.29 (s, 2H), 1.47 (s, 9H)

$^{13}$C NMR: (126 MHz, $CDCl_3$)

171.5, 165.4, 135.6, 127.5, 81.3, 52.1, 38.0, 28.1

IR: (neat)

2979 (w), 1742 (m), 1709 (m), 1641 (w), 1436 (w), 1420 (w), 1393 (w), 1368 (w), 1342 (w), 1323 (m), 1256 (m), 1204 (m), 1141 (s), 1016 (w), 943 (w), 879 (w), 851 (m), 816 (w), 744 (w)

MS: ($ESI^+$/QTOF)

224 (11%), 223 ($[M+Na]^+$, 100%), 145 (19%), 127 (14%), 102 (12%), 81 (14%), 74 (16%)

HRMS: ($ESI^+$/QTOF)

Calcd for $C_{10}H_{16}O_4Na^+$ $[M+Na]^+$: 223.0941, found: 223.0943

## E. Reaction Generality

### *tert*-Butyl (4*RS*,6*RS*)- and (4*RS*,6*SR*)-6-methyl-2-oxo-6-phenethylpiperidine-4-carboxylate (3a)

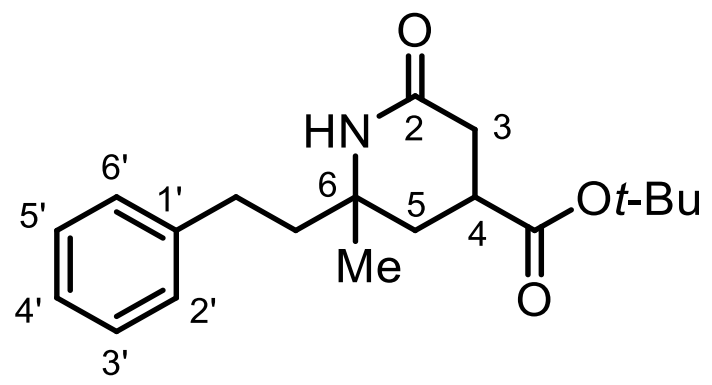


**3a**

Following a modification of ***General Procedure 1*** (with 1.2 equiv of **2**), 4-phenylbutan-2-amine **1a** (241 µL, 224 mg, 1.50 mmol, 1.0 equiv), 1-(*tert*-butyl) 4-methyl 2-methylenesuccinate **2** (360 mg, 1.80 mmol, 1.2 equiv), 4DPAIPN (12.0 mg, 15.0 µmol, 1 mol%), *i*-$Pr_3SiSH$ (32 µL, 29 mg, 0.15 mmol, 10 mol%), and TBAOH•$30H_2O$ (121 mg, 0.15 mmol, 10 mol%) were reacted. Purification *via* flash column chromatography on silica gel (12 g) in pentane/[EtOAc:EtOH (3:1), $NH_4OH$ (2%)], 0% (3 CV) then 0→30% (24 CV) then 30→100% (20 CV) then 100% (5 CV), followed by reversed-phase flash column chromatography on $C_{18}$ silica gel (15.5 g) in $H_2O$/MeCN, 20% (2 CV) then 20→100% (27 CV) then 100% (7 CV), gave **3a** as a colourless solid (119 mg, 83%, 56:44 dr).

Data for **3a**: (Peaks attributed to a diastereomer are denoted by *)

$^1$H NMR: (500 MHz, $CDCl_3$)

7.31–7.23 (m, 4H, C(3′)*H*, C(5′)*H*, C(3′)*H*$^*$, C(5′)*H*$^*$), 7.23–7.13 (m, 7H, C(2′)*H*, C(4′)*H*, C(6′)*H*, C(2′)*H*$^*$, C(4′)*H*$^*$, C(6′)*H*$^*$, N*H*), 7.00 (br s, 1H, N*H*$^*$), 2.88–2.76 (m, 2H, C(4)*H*, C(4)*H*$^*$), 2.69–2.53 (m, 6H, $PhCH_2CH_2$, $PhCH_2CH_2^*$, C(3)$H_A$, C(3)$H_A^*$), 2.47–2.33 (m, 2H, C(3)$H_B$, C(3)$H_B^*$), 2.13–2.06 (m, 1H, C(5)$H_A$), 1.98–1.68 (m, 6H, $PhCH_2CH_2$, $PhCH_2CH_2^*$, C(5)$H_B$, C(5)$H_A^*$), 1.63 (app t, $J$ = 13.3 Hz, 1H, C(5)$H_B^*$), 1.45 (m, 18H, $CO_2CMe_3$, $CO_2CMe_3^*$), 1.34 (s, 3H, C(6)*Me*), 1.30 (s, 3H, C(6)*Me*$^*$)

$^{13}$C NMR: (126 MHz, $CDCl_3$)

172.9 ($CO_2CMe_3$), 172.8 ($CO_2CMe_3^*$), 170.9 (*C*(2)), 170.8 (*C*(2)$^*$), 141.5 (*C*(1′)), 141.4 (*C*(1′)$^*$), 128.6 (*C*(3′), *C*(5′)), 128.5 (*C*(3′)$^*$, *C*(5′)$^*$), 128.4 (*C*(2′), *C*(6′)), 128.3 (*C*(2′)$^*$, *C*(6′)$^*$), 126.1 (*C*(4′)), 126.0 (*C*(4′)$^*$), 81.3 ($CO_2CMe_3$), 81.2 ($CO_2CMe_3^*$), 54.5 (*C*(6)), 54.1 (*C*(6)$^*$), 45.7 ($PhCH_2CH_2$), 44.0 ($PhCH_2CH_2^*$), 36.6 (*C*(5)), 36.3 (*C*(4)), 36.1 (*C*(4)$^*$), 35.4 (*C*(5)$^*$), 33.6 (*C*(3)), 33.1 (*C*(3)$^*$), 30.5 ($PhCH_2CH_2$), 30.0 ($PhCH_2CH_2^*$), 28.4 (C(6)*Me*), 28.1 (C(6)*Me*$^*$), 28.04 ($CO_2CMe_3$), 28.02 ($CO_2CMe_3^*$)

IR: (neat)

3183 (w), 3060 (w), 2974 (w), 1718 (w), 1661 (m), 1455 (w), 1404 (w), 1368 (w), 1293 (w), 1263 (w), 1224 (w), 1154 (w), 969 (w), 857 (w), 769 (w), 750 (w), 697 (w)

MS: ($ESI^+$/QTOF)

341 (10%), 340 ($[M+Na]^+$, 50%), 319 (20%), 318 ($[M+H]^+$, 100%)

HRMS: ($ESI^+$/QTOF)

Calcd for $C_{19}H_{27}NO_3Na^+$ $[M+Na]^+$, 340.1883; found, 340.1878

***tert*-Butyl 6,6-dimethyl-2-oxopiperidine-4-carboxylate (3b)**

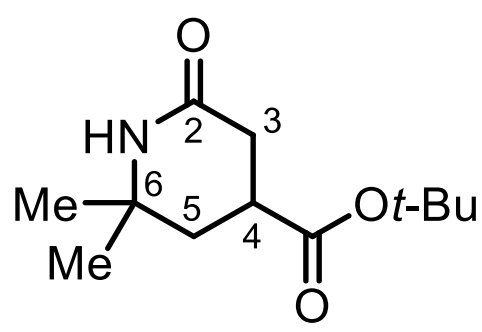


**3b**

Following a modification of ***General Procedure 1*** (with 2.0 equiv of amine **1b** due to volatility), propan-2-amine **1b** (258 µL, 177 mg, 3.00 mmol, 2.0 equiv), 1-(*tert*-butyl) 4-methyl 2-methylenesuccinate **2** (300 mg, 1.50 mmol, 1.0 equiv), 4DPAIPN (12.0 mg, 15.0 µmol, 1 mol%), *i*-$Pr_3SiSH$ (32 µL, 29 mg, 0.15 mmol, 10 mol%), and TBAOH•$30H_2O$ (121 mg, 0.15 mmol, 10 mol%) were reacted. Purification *via* flash column chromatography on silica gel (12 g) in pentane/[EtOAc:EtOH (3:1), $NH_4OH$ (2%)], 0% (2 CV) then 0→20% (20 CV) then 20→100% (15 CV) then 100% (8 CV), followed by reversed-phase flash column chromatography on $C_{18}$ silica gel (15.5 g) in $H_2O$/MeCN, 20% (2 CV) then 20→100% (30 CV) then 100% (8 CV), gave **3b** as a colourless solid (96.5 mg, 94%).

Data for **3b**:

mp: 177–179 °C

$^1$H NMR: (500 MHz, $CDCl_3$)

7.21 (br s, 1H, N*H*), 2.79–2.69 (m, 1H, C(4)*H*), 2.50 (ddd, *J* = 17.7, 5.6, 1.9 Hz, 1H, C(3)$H_A$), 2.32 (dd, *J* = 17.7, 12.0 Hz, 1H, C(3)$H_B$), 1.94–1.86 (m, 1H, C(5)$H_A$), 1.61–1.53 (m, 1H, C(5)$H_B$), 1.39 (s, 9H, $CO_2CMe_3$), 1.24 (s, 3H, C(6)$Me_A$)), 1.19 (s, 3H, C(6)$Me_B$)

<u>$^{13}$C NMR:</u> (126 MHz, $CDCl_3$)

172.8 (*C*O$_2$CMe$_3$), 170.6 (*C*(2)), 81.0 (CO$_2$*C*Me$_3$), 51.9 (*C*(6)), 38.0 (*C*(5)), 36.5 (*C*(4)), 33.2 (*C*(3)), 31.4 (CO$_2$C*Me*$_3$), 29.3 (C(6)*Me*$_A$), 28.0 (C(6)*Me*$_B$)

<u>IR:</u> (neat)

3198 (w), 3063 (w), 2974 (w), 2948 (w), 1715 (m), 1661 (s), 1453 (w), 1405 (w), 1366 (m), 1325 (w), 1307 (m), 1255 (m), 1224 (w), 1203 (w), 1157 (m), 1132 (m), 1112 (w), 1047 (w), 1015 (w), 961 (w), 914 (w), 884 (w), 867 (w), 850 (m), 821 (w), 767 (w), 742 (w), 609 (w)

<u>MS:</u> (ESI$^+$/QTOF)

251 (10%), 250 ([M+Na]$^+$, 79%), 229 (13%), 228 ([M+H]$^+$, 100%)

<u>HRMS:</u> (ESI$^+$/QTOF)

Calcd for $C_{12}H_{22}NO_3^+$ [M+H]$^+$, 228.1594; found, 228.1595

***tert*-Butyl (4*RS*,6*RS*)- and (4*RS*,6*SR*)-6-(1′-ethoxy-1′-oxoethyl)-6-methyl-2-oxopiperidine-4-carboxylate (3c)**

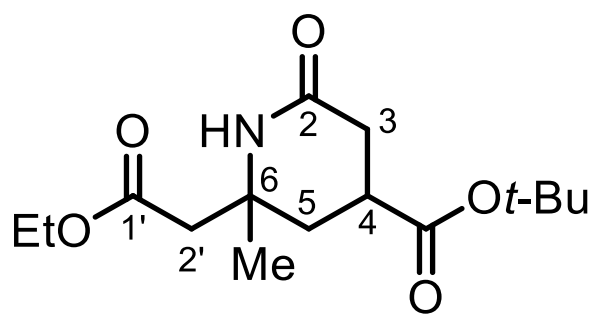


**3c**

Following ***General Procedure 1***, ethyl (*RS*)-3-aminobutyrate **1c** (220 μL, 179 mg, 1.50 mmol, 1.0 equiv), 1-(*tert*-butyl) 4-methyl 2-methylenesuccinate **2** (300 mg, 1.50 mmol, 1.0 equiv), 4DPAIPN (12.0 mg, 15.0 μmol, 1 mol%), *i*-Pr$_3$SiSH (32 μL, 29 mg, 0.15 mmol, 10 mol%), and TBAOH•30H$_2$O (121 mg, 0.15 mmol, 10 mol%) were reacted. Purification *via* flash column chromatography on silica gel (12 g) in pentane/[EtOAc:EtOH (3:1), $NH_4OH$ (2%)], 0% (2 CV) then 0→40% (25 CV) then 40→100% (15 CV) then 100% (10 CV), followed by reversed-phase flash column chromatography on $C_{18}$ silica gel (15.5 g) in $H_2O$/MeCN, 5% (2 CV) then 5→100% (30 CV) then 100% (8 CV), gave **3c** as a colourless solid (100 mg, 74%, 53:47 dr).

<u>Data for **3c**:</u> (Peaks attributed to a diastereomer are denoted by *)

<u>$^1$H NMR:</u> (500 MHz, $CDCl_3$)

6.96 (br s, 1H, N*H*), 6.75 (br s, 1H, N*H*$^*$), 4.14–4.05 (m, 4H, CO$_2$C*H*$_2$Me, CO$_2$C*H*$_2$Me$^*$), 2.82–2.67 (m, 2H, C(4)*H*, C(4)*H*$^*$), 2.60–2.28 (m, 8H, C(3)*H*$_A$, C(3)*H*$_B$, C(2′)*H*$_2$, C(3)*H*$_A$$^*$,

C(3)$H_B$*, C(2′)$H_2$*), 2.10–2.04 (m, 1H, C(5)$H_A$), 1.98–1.91 (m, 1H, C(5)$H_A$*), 1.70–1.56 (m, 2H, C(5)$H_B$, C(5)$H_B$*), 1.42–1.36 (m, 18H, $CO_2C$*Me*$_3$, $CO_2C$*Me*$_3$*), 1.36 (s, 3H, C(6)*Me*), 1.29 (s, 3H, C(6)*Me**), 1.24–1.16 (m, 6H, $CO_2CH_2$*Me*, $CO_2CH_2$*Me**)

$^{13}$C NMR: (126 MHz, $CDCl_3$)

172.4 (*C*$O_2CMe_3$), 172.4 (*C*$O_2CMe_3$*), 170.5 (*C*$O_2CH_2Me$), 170.4 (*C*$O_2CH_2Me$*), 170.0 (*C*(2)), 169.8 (*C*(2)*), 81.4 ($CO_2$*C*$Me_3$), 81.3 ($CO_2$*C*$Me_3$*), 60.9 ($CO_2$*C*$H_2Me$), 60.8 ($CO_2$*C*$H_2Me$*), 52.9 (*C*(6)), 52.8 (*C*(6)*), 47.2 (*C*(2′)), 45.3 (*C*(2′)*), 37.1 (*C*(5)), 37.1 (*C*(5)*), 36.2 (*C*(4)), 36.0 (*C*(4)*), 33.6 (*C*(3)), 33.3 (*C*(3)*), 28.9 (*C*(6)*Me*), 28.0 ($CO_2C$*Me*$_3$, $CO_2C$*Me*$_3$*), 26.8 (C(6)*Me**), 14.18 ($CO_2CH_2$*Me*), 14.16 ($CO_2CH_2$*Me**)

IR: (neat)

3181 (w), 3057 (w), 2977 (w), 2935 (w), 1717 (s), 1661 (s), 1448 (w), 1404 (w), 1384 (m), 1366 (m), 1326 (m), 1312 (m), 1295 (m), 1273 (m), 1236 (m), 1219 (m), 1198 (w), 1151 (s), 1110 (m), 1091 (m), 1026 (m), 967 (w), 847 (m), 754 (w), 648 (w), 617 (w)

MS: (ESI$^+$/QTOF)

323 (12%), 322 ($[M+Na]^+$, 76%), 301 (17%), 300 ($[M+H]^+$, 100%)

HRMS: (ESI$^+$/QTOF)

Calcd for $C_{15}H_{26}NO_5^+$ $[M+H]^+$, 300.1805; found, 300.1807

**_tert_-Butyl (4*RS*,6*RS*)- and (4*RS*,6*SR*)-6-cyclopropyl-6-methyl-2-oxopiperidine-4-carboxylate (3d)**

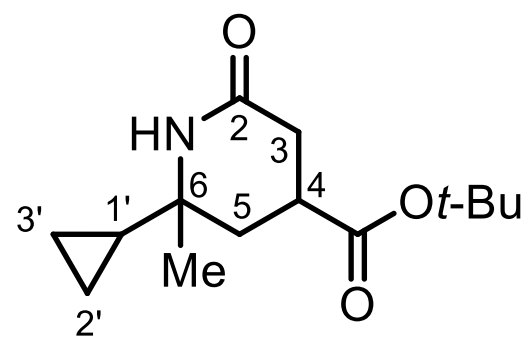


**3d**

Following ***General Procedure 1***, (*RS*)-1-cyclopropylethan-1-amine **1d** (139 µL, 128 mg, 1.50 mmol, 1.0 equiv), 1-(*tert*-butyl) 4-methyl 2-methylenesuccinate **2** (300 mg, 1.50 mmol, 1.0 equiv), 4DPAIPN (12.0 mg, 15.0 µmol, 1 mol%), *i*-$Pr_3SiSH$ (32 µL, 29 mg, 0.15 mmol, 10 mol%), and TBAOH•$30H_2O$ (121 mg, 0.15 mmol, 10 mol%) were reacted. Purification *via* flash column chromatography on silica gel (12 g) in pentane/[EtOAc:EtOH (3:1), $NH_4OH$ (2%)], 0% (3 CV) then 0→30% (22 CV) then 30→100% (18 CV) then 100% (7 CV), followed by reversed-phase flash column chromatography on $C_{18}$ silica gel (15.5 g) in $H_2O$/MeCN, 10% (2 CV) then 10→100% (30 CV) then 100% (8 CV), gave **1d** as a colourless solid (32.2 mg, 31%, 56:44 dr).

Data for **3d**: (Peaks attributed to a diastereomer are denoted by *)

$^1$H NMR: (500 MHz, $CDCl_3$)

5.83 (br s, 1H, N*H*), 5.66 (br s, 1H, N*H**), 3.12–3.02 (m, 1H, C(4)*H*), 2.84–2.74 (m, 1H, C(4)*H**), 2.62–2.53 (m, 2H, C(3)$H_A$, C(3)$H_A$*), 2.44–2.33 (m, 2H, C(3)$H_B$*, C(3)$H_B$*), 2.18–2.12 (m, 1H, C(5)$H_A$), 2.02–1.95 (m, 1H, C(5)$H_A$*), 1.75–1.66 (m, 2H, C(5)$H_B$, C(5)$H_B$*), 1.50–1.39 (m, 18H, $CO_2CMe_3$, $CO_2CMe_3$*), 1.23 (s, 3H, C(6)*Me*), 1.11 (s, 3H, C(6)*Me* *), 1.01–0.93 (m, 1H, C(1′)*H*), 0.89–0.80 (m, 1H, C(1′)*H**), 0.57–0.21 (m, 7H, C(2′)$H_2$, C(3′)$H_2$, and [C(2′)$H_2$*, C(3′)$H_A$*] or [C(2′)$H_A$*, C(3′)$H_2$*]), 0.13–0.04 (m, 1H, C(2′)$H_B$* or C(3′)$H_B$*)

$^{13}$C NMR: (126 MHz, $CDCl_3$)

173.2 ($CO_2CMe_3$), 172.9 ($CO_2CMe_3$*), 171.4 (*C*(2)), 170.7 (*C*(2)*), 81.4 ($CO_2CMe_3$), 81.3 ($CO_2CMe_3$*), 54.0 (*C*(6)), 53.2 (*C*(6)*), 38.9 (*C*(5)), 37.3 (*C*(5)*), 36.5 (*C*(4)), 36.2 (*C*(4)*), 33.8 (*C*(3)), 33.2 (*C*(3)*), 29.3 (C(6)*Me*), 28.2 ($CO_2CMe_3$, $CO_2CMe_3$*), 25.8 (C(6)*Me**), 23.3 (*C*(1′)), 21.3 (*C*(1′)*), 2.2, 1.0 (*C*(2′), *C*(3′)), 0.3, –1.5 (*C*(2′)*, *C*(3′)*)

IR: (neat)

3184 (w), 3062 (w), 3003 (w), 2973 (w), 2933 (w), 1719 (m), 1655 (s), 1436 (w), 1405 (w), 1393 (w), 1366 (m), 1285 (m), 1256 (m), 1219 (w), 1154 (m), 1072 (w), 1021 (w), 1004 (w), 964 (w), 906 (w), 847 (m), 800 (w), 781 (w), 753 (w), 730 (w), 680 (w)

MS: ($ESI^+$/QTOF)

254 ($[M+H]^+$, 100%), 158 (45%), 152 (34%), 150 (63), 142 (65%), 111 (39%), 110 (37%), 85 (78%)

HRMS: ($ESI^+$/QTOF)

Calcd for $C_{14}H_{24}NO_3^+$ $[M+H]^+$, 254.1751; found, 254.1751

***tert*-Butyl (*RS*)-2-oxo-1-azaspiro[5.5]undecane-4-carboxylate (3e)**

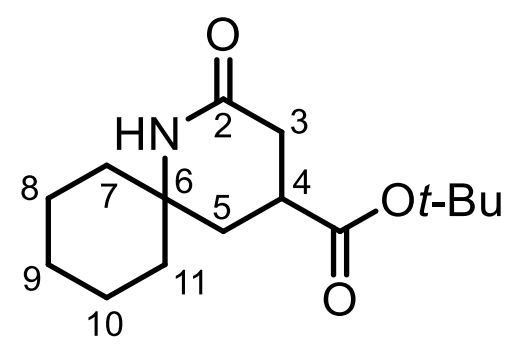


**3e**

Following ***General Procedure 1***, cyclohexylamine **1e** (172 µL, 149 mg, 1.50 mmol, 1.0 equiv), 1-(*tert*-butyl) 4-methyl 2-methylenesuccinate **2** (300 mg, 1.50 mmol, 1.0 equiv), 4DPAIPN (12.0 mg, 15.0 µmol, 1 mol%), *i*-$Pr_3SiSH$ (32 µL, 29 mg, 0.15 mmol, 10 mol%), and TBAOH•30$H_2O$ (121 mg, 0.15

mmol, 10 mol%) were reacted. Purification *via* flash column chromatography on silica gel (12 g) in pentane/[EtOAc:EtOH (3:1), $NH_4OH$ (2%)], 0% (3 CV) then 0→20% (25 CV) then 20→100% (18 CV) then 100% (4 CV), followed by reversed-phase flash column chromatography on $C_{18}$ silica gel (15.5 g) in $H_2O$/MeCN, 20% (2 CV) then 20→100% (30 CV) then 100% (8 CV), gave **3e** as a colourless solid (85.4 mg, 71%).

Data for **3e**:

mp: 174–175 °C

$^1$H NMR: (500 MHz, $CDCl_3$)

6.41 (br s, 1H, N*H*), 2.80–2.70 (m, 1H, C(4)*H*), 2.61–2.52 (m, 1H, C(3)$H_A$), 2.39 (dd, $J$ = 17.7, 11.9 Hz, 1H, C(3)$H_B$), 2.18–2.10 (m, 1H, C(5)$H_A$), 1.60–1.37 (m, 20H, C(5)$H_B$, $CO_2C$*Me*$_3$, C(7)$H_2$, C(8)$H_2$, C(9)$H_2$, C(10)$H_2$, C(11)$H_2$)

$^{13}$C NMR: (126 MHz, $CDCl_3$)

173.1 (*C*$O_2CMe_3$), 170.2 (*C*(2)), 81.3 ($CO_2$*C*$Me_3$), 53.7 (*C*(6)), 40.4, 37.7 (*C*(7), *C*(11)), 35.9 (*C*(4)), 35.5 (br s, *C*(5)), 33.8 (*C*(3)), 28.1 ($CO_2C$*Me*$_3$), 25.3, 22.1, 21.7 (*C*(8), *C*(9), *C*(10))

IR: (neat)

3169 (w), 3042 (w), 2975 (w), 2932 (w), 2865 (w), 1721 (m), 1655 (m), 1483 (w), 1456 (w), 1434 (w), 1404 (w), 1393 (w), 1368 (w), 1284 (w), 1264 (w), 1249 (w), 1225 (w), 1146 (m), 1119 (w), 1044 (w), 975 (w), 928 (w), 909 (w), 864 (w), 845 (w), 836 (w), 785 (w), 758 (w), 727 (w), 634 (w)

MS: (ESI$^+$/QTOF)

291 (11%), 290 ($[M+Na]^+$, 68%), 269 (17%), 268 ($[M+H]^+$, 100%)

HRMS: (ESI$^+$/QTOF)

Calcd for $C_{15}H_{26}NO_3^+$ $[M+H]^+$, 268.1907; found, 268.1904

***tert*-Butyl (*RS*)-2-oxo-1-azaspiro[4.5]decane-4-carboxylate (3f)**

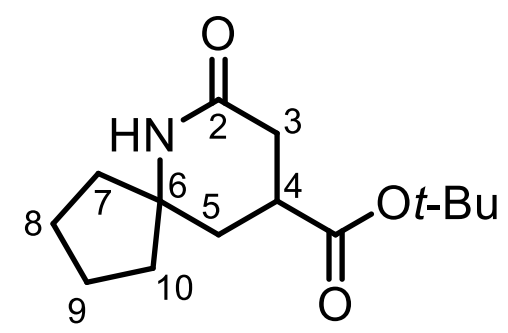


**3f**

Following ***General Procedure 1***, cyclopentylamine **1f** (148 µL, 128 mg, 1.50 mmol, 1.0 equiv), 1-(*tert*-butyl) 4-methyl 2-methylenesuccinate **2** (300 mg, 1.50 mmol, 1.0 equiv), 4DPAIPN (12.0 mg, 15.0 µmol, 1 mol%), *i*-$Pr_3SiSH$ (32 µL, 29 mg, 0.15 mmol, 10 mol%), and TBAOH•$30H_2O$ (121 mg, 0.15 mmol, 10 mol%) were reacted. Purification *via* flash column chromatography on silica gel (12 g) in pentane/[EtOAc:EtOH (3:1), $NH_4OH$ (2%)], 0% (3 CV) then 0→20% (25 CV) then 20→100% (18 CV) then 100% (4 CV), followed by reversed-phase flash column chromatography on $C_{18}$ silica gel (15.5 g) in $H_2O$/MeCN, 20% (2 CV) then 20→100% (30 CV) then 100% (8 CV), gave **3f** as a colourless solid (97.1 mg, 85%).

Data for **3f**:

mp: 168–170 °C

$^1$H NMR: (500 MHz, $CDCl_3$)

6.28 (br s, 1H, N*H*), 2.80–2.71 (m, 1H, C(4)*H*), 2.60 (ddd, *J* = 17.7, 5.7, 1.9 Hz, 1H, C(3)$H_A$), 2.43 (dd, *J* = 17.7, 11.9 Hz, 1H, C(3)$H_B$), 2.03–1.97 (m, 1H, C(5)$H_A$), 1.80–1.62 (m, 9H, C(5)$H_B$, C(7)$H_2$, C(8)$H_2$, C(9)$H_2$, C(10)$H_2$), 1.45 (s, 9H, $CO_2CMe_3$)

$^{13}$C NMR: (126 MHz, $CDCl_3$)

172.9 ($CO_2CMe_3$), 170.4 (*C*(2)), 81.3 ($CO_2CMe_3$), 62.9 (*C*(6)), 41.4, 39.9 (*C*(7), *C*(10)), 37.6 (*C*(4)), 36.4 (*C*(5)), 33.7 (*C*(3)), 28.1 ($CO_2CMe_3$), 23.4, 23.0 (*C*(8), *C*(9))

IR: (neat)

3187 (w), 3055 (w), 2976 (w), 2932 (w), 2881 (w), 1720 (m), 1652 (m), 1472 (w), 1452 (w), 1410 (w), 1391 (w), 1365 (m), 1290 (w), 1255 (w), 1238 (w), 1153 (m), 1108 (w), 1069 (w), 978 (w), 925 (w), 854 (m), 826 (w), 766 (w), 735 (w)

MS: (ESI$^+$/QTOF)

277 (15%), 276 ([M+Na]$^+$, 100%), 254 ([M+H]$^+$, 57%)

HRMS: (ESI$^+$/QTOF)

Calcd for $C_{14}H_{24}NO_3^+$ [M+H]$^+$, 254.1751; found, 254.1747

**_tert_-Butyl (_RS_)-2-oxo-1-azaspiro[3.5]nonane-4-carboxylate (3g)**

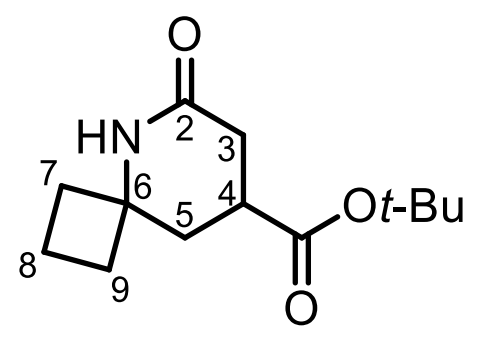


**3g**

Following ***General Procedure 1***, cyclobutylamine **1g** (128 µL, 107 mg, 1.50 mmol, 1.0 equiv), 1-(*tert*-butyl) 4-methyl 2-methylenesuccinate **2** (300 mg, 1.50 mmol, 1.0 equiv), 4DPAIPN (12.0 mg, 15.0 µmol, 1 mol%), *i*-$Pr_3SiSH$ (32 µL, 29 mg, 0.15 mmol, 10 mol%), and TBAOH•$30H_2O$ (121 mg, 0.15 mmol, 10 mol%) were reacted. Purification *via* flash column chromatography on silica gel (12 g) in pentane/[EtOAc:EtOH (3:1), $NH_4OH$ (2%)], 0% (2 CV) then 0→20% (18 CV) then 20→100% (12 CV) then 100% (5 CV), followed by reversed-phase flash column chromatography on $C_{18}$ silica gel (15.5 g) in $H_2O$/MeCN, 5% (2 CV) then 5→100% (30 CV) then 100% (8 CV), gave **3g** as a colourless solid (90.6 mg, 84%).

Data for **3g**:

mp: 171–172 °C

$^1$H NMR: (500 MHz, $CDCl_3$)

7.77 (br s, 1H, N*H*), 2.60 (m, 1H, C(4)*H*), 2.50 (ddd, *J* = 17.7, 5.4, 1.8 Hz, 1H, C(3)$H_A$), 2.35 (dd, *J* = 17.7, 11.9 Hz, 1H, C(3)$H_B$), 2.27 (br d, *J* = 13.2 Hz, 1H, C(5)$H_A$), 2.22–2.14 (m, 1H, C(7)$H_A$), 2.07–1.98 (m, 3H, C(7)$H_B$, C(9)$H_2$), 1.76–1.57 (m, 3H, C(5)$H_B$, C(8)$H_2$), 1.35–1.45 (m, 9H, $CO_2CMe_3$)

$^{13}$C NMR: (126 MHz, $CDCl_3$)

172.7 ($CO_2CMe_3$), 170.6 (*C*(2)), 81.2 ($CO_2CMe_3$), 55.7 (*C*(6)), 36.6 (*C*(4)), 36.3, 36.2 (*C*(7), *C*(9)), 35.0 (*C*(5)), 33.7 (*C*(3)), 28.0 ($CO_2CMe_3$), 13.1 (*C*(8))

IR: (neat)

3178 (w), 3057 (w), 2980 (w), 2935 (w), 1723 (m), 1661 (m), 1404 (w), 1368 (w), 1296 (m), 1267 (w), 1248 (w), 1208 (w), 1153 (m), 1129 (w), 1114 (m), 1071 (w), 1031 (w), 999 (w), 965 (w), 940 (w), 863 (w), 848 (m), 806 (w), 760 (w), 740 (w), 687 (w)

MS: (ESI$^+$/QTOF)

263 (11%), 262 ([M+Na]$^+$, 76%), 240 ([M+H]$^+$, 100%), 241 (14%)

HRMS: (ESI$^+$/QTOF)

Calcd for $C_{13}H_{21}NO_3Na^+$ [M+Na]$^+$, 262.1414; found, 262.1408

**Di-*tert*-butyl (*RS*)-2-oxo-1,9-diazaspiro[5.5]undecane-4,9-dicarboxylate (3h)**

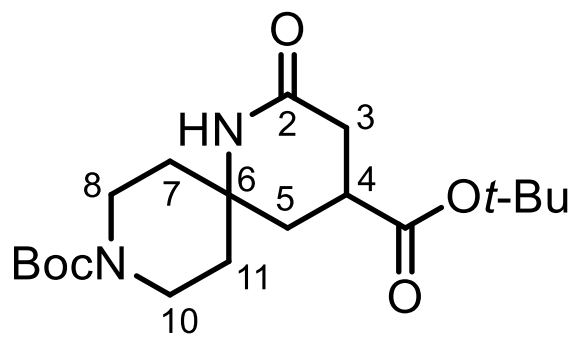

**3h**

Following ***General Procedure 1***, *tert*-butyl 4-aminopiperidine-1-carboxylate **1h** (300 mg, 1.50 mmol, 1.0 equiv), 1-(*tert*-butyl) 4-methyl 2-methylenesuccinate **2** (300 mg, 1.50 mmol, 1.0 equiv), 4DPAIPN (12.0 mg, 15.0 µmol, 1 mol%), *i*-$Pr_3SiSH$ (32 µL, 29 mg, 0.15 mmol, 10 mol%), and TBAOH•$30H_2O$ (121 mg, 0.15 mmol, 10 mol%) were reacted. Purification *via* flash column chromatography on silica gel (12 g) in pentane/[EtOAc:EtOH (3:1), $NH_4OH$ (2%)], 0% (3 CV) then 0→20% (25 CV) then 20→100% (18 CV) then 100% (4 CV), followed by reversed-phase flash column chromatography on $C_{18}$ silica gel (15.5 g) in $H_2O$/MeCN, 20% (2 CV) then 20→100% (30 CV) then 100% (8 CV), gave **3h** as a colourless solid (126 mg, 76%).

Data for **3h**:

mp: 202–205 °C (decomp)

$^1$H NMR: (500 MHz, $CDCl_3$)

7.47 (br s, 1H, N*H*), 3.58–3.51 (m, 1H, C(8)$H_A$ or C(10)$H_A$), 3.48–3.34 (m, 3H, [C(8)$H_A$ or C(10)$H_A$] and [C(8)$H_2$ or C(10)$H_2$]), 2.78–2.69 (m, 1H, C(4)*H*), 2.55 (ddd, *J* = 17.8, 5.6, 1.5 Hz, 1H, C(3)$H_A$), 2.38 (dd, *J* = 17.8, 11.9 Hz, 1H, C(3)$H_B$), 2.16–2.07 (m, 1H, C(5)$H_A$), 1.65–1.48 (m, 5H, C(5)$H_B$, C(7)$H_2$, C(11)$H_2$), 1.45–1.31 (m, 18H, C(4)$CO_2$C*Me*$_3$, N$CO_2$C*Me*$_3$)

$^{13}$C NMR: (126 MHz, $CDCl_3$)

172.6 (C(4)*C*$O_2$CMe$_3$), 170.9 (*C*(2)), 154.6 (N*C*$O_2$CMe$_3$), 81.4, 79.8 (C(4)$CO_2$*C*Me$_3$, N$CO_2$*C*Me$_3$), 52.3 (*C*(6)), 39.5 (br, *C*(8), *C*(10)), 39.2 (br), 37.0 (br) (*C*(7), *C*(11)), 35.6 (*C*(4)), 34.8 (br, *C*(5)), 33.7 (*C*(3)), 28.5, 28.0 (C(4)$CO_2$C*Me*$_3$, N$CO_2$C*Me*$_3$)

IR: (neat)

2975 (w), 1719 (w), 1682 (w), 1649 (m), 1411 (w), 1392 (w), 1366 (w), 1296 (w), 1277 (w), 1248 (w), 1155 (m), 1090 (w), 1076 (w), 966 (w), 846 (w), 764 (w)

MS: (ESI$^+$/QTOF)

392 (23%), 391 ([M+Na]$^+$, 100%), 370 (10%), 369 ([M+H]$^+$, 47%)

<u>HRMS:</u> (ESI$^+$/QTOF)

Calcd for $C_{19}H_{33}N_2O_5^+$ [M+H]$^+$, 369.2311; found, 369.2381

**_tert_-Butyl (_RS_)-2-oxo-9-oxa-1-azaspiro[5.5]undecane-4-carboxylate (3i)**

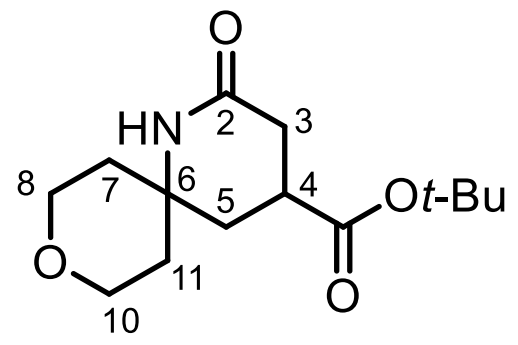


**3i**

Following ***General Procedure 1***, 4-aminotetrahydro-2*H*-pyran **1i** (155 µL, 151 mg, 1.50 mmol, 1.0 equiv), 1-(*tert*-butyl) 4-methyl 2-methylenesuccinate **2** (300 mg, 1.50 mmol, 1.0 equiv), 4DPAIPN (12.0 mg, 15.0 µmol, 1 mol%), *i*-$Pr_3SiSH$ (32 µL, 29 mg, 0.15 mmol, 10 mol%), and TBAOH•30$H_2O$ (121 mg, 0.15 mmol, 10 mol%) were reacted. Purification *via* flash column chromatography on silica gel (12 g) in pentane/[EtOAc:EtOH (3:1), $NH_4OH$ (2%)], 0% (2 CV) then 0→20% (18 CV) then 20→100% (12 CV) then 100% (5 CV), followed by reversed-phase flash column chromatography on $C_{18}$ silica gel (15.5 g) in $H_2O$/MeCN, 5% (2 CV) then 5→100% (30 CV) then 100% (8 CV), gave **3i** as a colourless solid (95.4 mg, 79%).

<u>Data for **3i**:</u>

<u>mp:</u> 190–193 °C

<u>$^1$H NMR:</u> (500 MHz, $CDCl_3$)

7.53 (br s, 1H, N*H*), 3.82–3.75 (m, 1H, C(8)$H_A$ or C(10)$H_A$), 3.73–3.58 (m, 3H, [C(8)$H_B$ or C(10)$H_B$] and [C(8)$H_2$ or C(10)$H_B$]), 2.86–2.77 (m, 1H, C(4)*H*), 2.55 (ddd, $J$ = 17.8, 5.6, 1.8 Hz, 1H, C(3)$H_A$), 2.37 (dd, $J$ = 17.8, 11.8 Hz, 1H, C(3)$H_B$), 2.20–2.14 (m, 1H, C(5)$H_A$), 1.70–1.59 (m, 4H, C(7)$H_2$, C(11)$H_2$), 1.52 (app t, $J$ = 13.2 Hz, 1H, C(5)$H_B$), 1.39 (s, 9H, $CO_2CMe_3$)

Concentration dependence was observed; the data reported is consistent at analyte concentrations of ≥0.15 M. At analyte concentrations of ≤0.10 M, the resonance at δ 3.82–3.75 moves under the resonance at δ 3.73–3.58, and the resonance at δ 1.52 moves under the resonance at δ 2.20–2.14. We have not checked whether this phenomenon is common to all of the δ-lactams reported in this work.

<u>$^{13}$C NMR:</u> (126 MHz, $CDCl_3$)

172.6 ($CO_2CMe_3$), 170.8 (*C*(2)), 81.3 ($CO_2CMe_3$), 63.7, 63.1 (*C*(8), *C*(10)), 51.4 (*C*(6)), 39.9, 37.9 (*C*(7), *C*(11)), 35.5 (*C*(4)), 35.3 (*C*(5)), 33.7 (*C*(3)), 28.0 ($CO_2CMe_3$)

<u>IR:</u> (neat)

3200 (w), 2976 (w), 2921 (w), 2869 (w), 1715 (m), 1647 (m), 1459 (w), 1406 (w), 1390 (w), 1368 (w), 1296 (w), 1272 (w), 1248 (m), 1202 (w), 1154 (m), 1105 (m), 1092 (m), 1033 (w), 1017 (w), 974 (w), 936 (w), 871 (w), 843 (m), 806 (w), 789 (w), 766 (w), 729 (w), 682 (w), 637 (w)

<u>MS:</u> ($ESI^+$/QTOF)

293 (10%), 292 ($[M+Na]^+$, 70%), 271 (14%), 270 ($[M+H]^+$, 100%)

<u>HRMS:</u> ($ESI^+$/QTOF)

Calcd for $C_{14}H_{23}NO_4Na^+$ $[M+Na]^+$, 292.1519; found, 292.1519

***tert*-Butyl (*RS*)-2-oxo-9-thia-1-azaspiro[5.5]undecane-4-carboxylate (3j)**

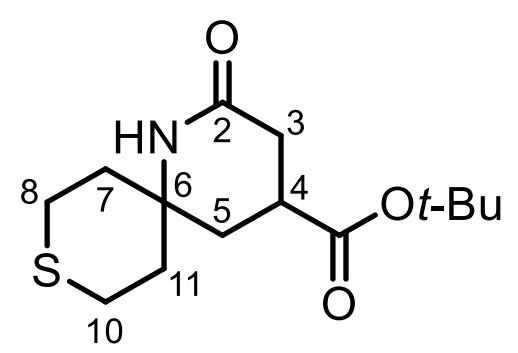


**3j**

Following ***General Procedure 1***, tetrahydro-2*H*-thiopyran-4-amine **1j** (176 mg, 1.50 mmol, 1.0 equiv), 1-(*tert*-butyl) 4-methyl 2-methylenesuccinate **2** (300 mg, 1.50 mmol, 1.0 equiv), 4DPAIPN (12.0 mg, 15.0 µmol, 1 mol%), *i*-$Pr_3SiSH$ (32 µL, 29 mg, 0.15 mmol, 10 mol%), and TBAOH•$30H_2O$ (121 mg, 0.15 mmol, 10 mol%) were reacted. Purification *via* flash column chromatography on silica gel (12 g) in pentane/[EtOAc:EtOH (3:1), $NH_4OH$ (2%)], 0% (2 CV) then 0→30% (20 CV) then 30→100% (15 CV) then 100% (8 CV), followed by reversed-phase flash column chromatography on $C_{18}$ silica gel (15.5 g) in $H_2O$/MeCN, 20% (2 CV) then 20→100% (30 CV) then 100% (8 CV), gave **3j** as a colourless solid (117 mg, 91%).

<u>Data for **3j**:</u>

<u>mp:</u> 200–202 °C

<u>$^1$H NMR:</u> (500 MHz, $CDCl_3$)

7.39 (br s, 1H, N*H*), 2.80–2.68 (m, 2H, C(4)*H*, C(8)$H_A$ or C(10)$H_A$), 2.68–2.51 (m, 4H, C(3)$H_A$, [C(8)$H_B$ or C(10)$H_B$], [C(8)$H_2$ or C(10)$H_2$]), 2.35 (dd, $J$ = 17.8, 11.9 Hz, 1H, C(3)$H_B$), 2.16–2.08 (m, 1H, C(5)$H_A$), 1.91–1.78 (m, 4H, C(7)$H_2$, C(11)$H_2$), 1.53–1.20 (m, 10H, C(5)$H_B$, $CO_2$C*Me*$_3$)

<u>$^{13}$C NMR:</u> (126 MHz, $CDCl_3$)

172.6 (*C*$O_2$CMe$_3$), 170.7 (*C*(2)), 81.3 ($CO_2$*C*Me$_3$), 52.6 (*C*(6)), 41.1, 38.6 (*C*(8), *C*(10)), 35.3 (*C*(4)), 35.0 (*C*(5)), 33.7 (*C*(3)), 28.0 ($CO_2$C*Me*$_3$), 24.1, 23.6 (*C*(7), *C*(11))

<u>IR:</u> (neat)

3204 (w), 3073 (w), 2977 (w), 2909 (w), 1714 (m), 1646 (m), 1448 (w), 1440 (w), 1407 (w), 1389 (w), 1366 (w), 1297 (w), 1273 (w), 1259 (w), 1240 (w), 1232 (w), 1216 (w), 1152 (m), 1079 (w), 989 (w), 963 (w), 935 (w), 919 (w), 844 (w), 826 (w), 810 (w), 779 (w), 765 (w), 727 (w), 666 (w), 615 (w)

<u>MS:</u> (ESI$^+$/QTOF)

309 (17%), 308 ([M+Na]$^+$, 100%), 287 (17%), 286 ([M+H]$^+$, 94%)

<u>HRMS:</u> (ESI$^+$/QTOF)

Calcd for $C_{14}H_{23}NO_3SNa^+$ [M+Na]$^+$, 308.1291; found, 308.1298

***tert*-Butyl (*RS*)-2-oxo-9-thia-1-azaspiro[5.5]undecane-4-carboxylate 9,9-dioxide (3k)**

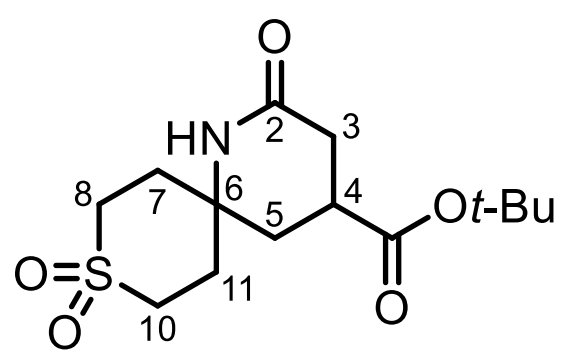


**3k**

Following ***General Procedure 1***, 4-aminotetrahydro-2*H*-thiopyran 1,1-dioxide **1k** (224 mg, 1.50 mmol, 1.0 equiv), 1-(*tert*-butyl) 4-methyl 2-methylenesuccinate **2** (300 mg, 1.50 mmol, 1.0 equiv), 4DPAIPN (12.0 mg, 15.0 µmol, 1 mol%), *i*-$Pr_3SiSH$ (32 µL, 29 mg, 0.15 mmol, 10 mol%), and TBAOH•30$H_2O$ (121 mg, 0.15 mmol, 10 mol%) were reacted. Purification *via* flash column chromatography on silica gel (12 g) in pentane/[EtOAc:EtOH (3:1), $NH_4OH$ (2%)], 0% (2 CV) then 0→20% (18 CV) then 20→100% (12 CV) then 100% (5 CV), followed by reversed-phase flash column chromatography on $C_{18}$ silica gel (15.5 g) in $H_2O$/MeCN, 5% (2 CV) then 5→100% (30 CV) then 100% (8 CV), gave **3k** as a colourless solid (134 mg, 94%).

<u>Data for **3k**:</u>

mp: 214–217 °C

$^1$H NMR: (500 MHz, $CDCl_3$)

9.00 (br s, 1H, N*H*), 3.75–3.63 (m, 1H, C(8)$H_A$ or C(10)$H_A$), 3.42–3.30 (m, 1H, C(8)$H_B$ or C(10)$H_B$), 2.98–2.85 (m, 2H, C(8)$H_2$ or C(10)$H_2$), 2.80–2.70 (m, 1H, C(4)*H*), 2.61 (ddd, $J$ = 18.0, 5.5, 1.5 Hz, 1H, C(3)$H_A$), 2.41 (dd, $J$ = 18.0, 11.8 Hz, 1H, C(3)$H_B$), 2.35–2.25 (m, 1H, C(7)$H_A$ or C(11)$H_A$), 2.24–2.16 (m, 2H, C(7)$H_2$ or C(11)$H_2$), 2.11–2.02 (m, 2H, [C(7)$H_B$ or C(11)$H_B$], C(5)$H_A$), 1.69 (app t, $J$ = 13.3 Hz, 1H, C(5)$H_B$), 1.44 (s, 9H, $CO_2CMe_3$)

$^{13}$C NMR: (126 MHz, $CDCl_3$)

172.11, 172.07 (*C*(2), *C*$O_2CMe_3$), 81.9 ($CO_2$*C*$Me_3$), 51.3 (*C*(6)), 46.4, 46.3 (*C*(8), *C*(10)), 37.7 (*C*(7) or *C*(11)), 36.4 (*C*(5)), 36.1 (*C*(11) or *C*(7)), 35.8 (*C*(4)), 33.6 (*C*(3)), 28.1 ($CO_2C$*Me*$_3$)

IR: (neat)

3186 (w), 3066 (w), 2937 (w), 1719 (m), 1655 (m), 1436 (w), 1393 (w), 1366 (w), 1328 (w), 1295 (m), 1272 (m), 1235 (w), 1137 (m), 1112 (m), 1087 (w), 1036 (w), 993 (w), 936 (w), 919 (w), 844 (m), 759 (w), 712 (w), 671 (w)

MS: ($ESI^+$/QTOF)

340 ($[M+Na]^+$, 10%), 319 (18%), 318 ($[M+H]^+$, 100%)

HRMS: ($ESI^+$/QTOF)

Calcd for $C_{14}H_{24}NO_5S^+$ $[M+H]^+$, 318.1370; found, 318.1366

**Di-*tert*-butyl (*RS*)-2-oxo-1,8-diazaspiro[3.5]nonane-4,8-dicarboxylate (3l)**

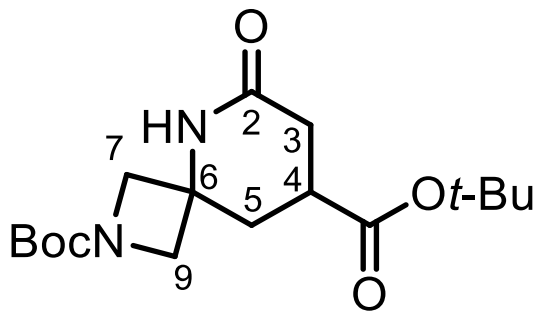


**3l**

Following a modification of ***General Procedure 1*** (with 3.0 equiv of amine **1l**), *tert*-butyl 3-aminoazetidine-1-carboxylate **1l** (705 µL, 774 mg, 4.50 mmol, 3.0 equiv), 1-(*tert*-butyl) 4-methyl 2-methylenesuccinate **2** (300 mg, 1.50 mmol, 1.0 equiv), 4DPAIPN (12.0 mg, 15.0 µmol, 1 mol%), *i*-$Pr_3SiSH$ (32 µL, 29 mg, 0.15 mmol, 10 mol%), and TBAOH•30$H_2O$ (121 mg, 0.15 mmol, 10 mol%)

were reacted. Purification *via* flash column chromatography on silica gel (12 g) in pentane/[EtOAc:EtOH (3:1), $NH_4OH$ (2%)], 0% (3 CV) then 0→20% (25 CV) then 20→100% (18 CV) then 100% (4 CV), followed by reversed-phase flash column chromatography on $C_{18}$ silica gel (15.5 g) in $H_2O$/MeCN, 20% (2 CV) then 20→100% (30 CV) then 100% (8 CV), gave **3l** as a colourless solid (83 mg, 54%).

Data for **3l**:

mp: 213–215 °C

$^1$H NMR: (500 MHz, $CDCl_3$)

6.90 (br s, 1H, N*H*), 3.95–3.87 (m, 3H, [C(7)$H_2$ or C(9)$H_2$] and [C(7)$H_A$ or C(9)$H_A$]), 3.83 (d, $J$ = 8.8 Hz, 1H, C(7)$H_B$ or C(9)$H_B$), 2.71–2.62 (m, 1H, C(4)*H*), 2.58 (ddd, $J$ = 17.8, 5.4, 1.3 Hz, 1H, C(3)$H_A$), 2.49–2.37 (m, 2H, C(3)$H_B$, C(5)$H_A$), 1.94 (app t, $J$ = 12.6 Hz, 1H, C(5)$H_B$), 1.48–1.41 (m, 18H, C(4)$CO_2$C$Me_3$, N$CO_2$C$Me_3$)

$^{13}$C NMR: (126 MHz, $CDCl_3$)

171.9 (C(4)*C*$O_2$CMe$_3$), 170.5 (*C*(2)), 156.2 (N*C*$O_2$CMe$_3$), 81.9, 80.4 (C(4)$CO_2$*C*Me$_3$, N$CO_2$*C*Me$_3$), 62.9 (br), 62.2 (br) (*C*(7), *C*(8)), 51.7 (*C*(6)), 36.7 (*C*(4)), 35.1 (*C*(5)), 33.5 (*C*(3)), 28.5, 28.1 (C(4)$CO_2$C$Me_3$, N$CO_2$C$Me_3$)

IR: (neat)

3187 (w), 3075 (w), 2977 (w), 2872 (w), 1712 (m), 1669 (m), 1479 (w), 1457 (w), 1381 (w), 1365 (m), 1310 (w), 1291 (w), 1250 (w), 1214 (w), 1148 (m), 1118 (w), 1101 (m), 985 (w), 958 (w), 908 (w), 857 (w), 842 (w), 777 (w), 764 (w), 741 (w), 608 (w)

MS: (ESI$^+$/QTOF)

1044 (13%), 771 (19%), 703 ([2M+Na]$^+$, 100%), 363 ([M+Na]$^+$, 65%), 318 (15%)

HRMS: (ESI$^+$/QTOF)

Calcd for $C_{17}H_{28}N_2O_5Na^+$ [M+Na]$^+$, 363.1890; found, 363.1892

***tert*-Butyl (*RS*)-2-oxo-8-oxa-1-azaspiro[3.5]nonane-4-carboxylate (3m)**

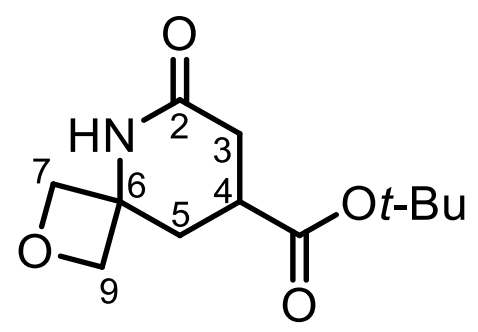

**3m**

Following a modification of ***General Procedure 1*** (with 3.0 equiv of amine **1m**), 3-aminooxetane **1m** (315 µL, 330 mg, 4.50 mmol, 3.0 equiv), 1-(*tert*-butyl) 4-methyl 2-methylenesuccinate **7** (300 mg, 1.50 mmol, 1.0 equiv), 4DPAIPN (12.0 mg, 15.0 µmol, 1 mol%), *i*-$Pr_3SiSH$ (32 µL, 29 mg, 0.15 mmol, 10 mol%), and TBAOH•$30H_2O$ (121 mg, 0.15 mmol, 10 mol%) were reacted. Purification *via* flash column chromatography on silica gel (12 g) in pentane/[EtOAc:EtOH (3:1), $NH_4OH$ (2%)], 0% (3 CV) then 0→30% (2 CV) then 30→100% (18 CV) then 100% (7 CV), followed by reversed-phase flash column chromatography on $C_{18}$ silica gel (15.5 g) in $H_2O$/MeCN, 10% (2 CV) then 10→100% (30 CV) then 100% (8 CV), gave **3m** as a colourless solid (72 mg, 66%).

Data for **3m**:

mp: 198–204 °C

$^1$H NMR: (500 MHz, $CDCl_3$)

6.95 (br s, 1H, N*H*), 4.65–4.51 (m, 4H, C(7)$H_2$, C(9)$H_2$), 2.70–2.52 (m, 3H, C(4)*H*, C(3)$H_A$, C(5)$H_A$), 2.51–2.41 (m, 1H, C(3)$H_B$), 2.09–2.01 (m, 1H, C(5)$H_B$), 1.47 (s, 9H, $CO_2CMe_3$)

$^{13}$C NMR: (126 MHz, $CDCl_3$)

172.0 ($CO_2CMe_3$), 170.3 (*C*(2)), 84.0, 83.3 (*C*(7), *C*(9)), 81.9 ($CO_2CMe_3$), 56.5 (*C*(6)), 36.6 (*C*(4)), 34.2, 33.6 (*C*(3), *C*(5)), 28.1 ($CO_2CMe_3$)

IR: (neat)

3182 (w), 3066 (w), 2976 (w), 2951 (w), 2869 (w), 1719 (m), 1663 (m), 1476 (w), 1394 (w), 1380 (w), 1370 (w), 1338 (w), 1326 (w), 1308 (w), 1294 (w), 1258 (w), 1212 (w), 1157 (m), 1140 (m), 1119 (w), 1081 (w), 982 (m), 953 (w), 924 (w), 837 (w), 804 (w), 758 (w), 742 (w)

MS: (ESI$^+$/QTOF)

505 ([2M+Na]$^+$, 24%), 324 (24%), 264 ([M+Na]$^+$, 100%), 242 ([M+H]$^+$, 57%), 186 (53%), 142 (20%), 133 (18%), 126 (42%), 85 (15%)

HRMS: (ESI$^+$/QTOF)

Calcd for $C_{12}H_{19}NO_4Na^+$ [M+Na]$^+$, 264.1206; found, 264.1208

***tert*-Butyl (4*RS*,6*RS*)- and (4*RS*,6*SR*)-6-[(4-fluorophenyl)methyl]-2-oxopiperidine-4-carboxylate (S1)**

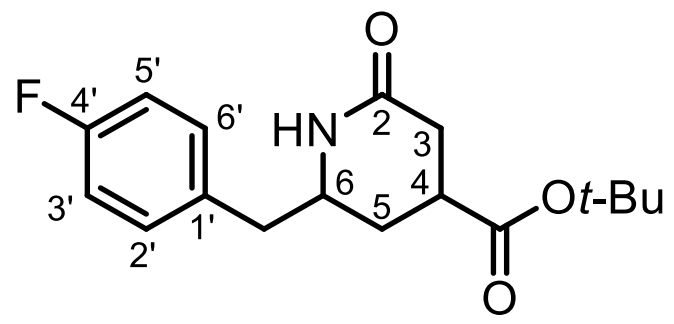


**S1**

Following ***General Procedure 1***, 2-(4-fluorophenyl)ethan-1-amine (197 µL, 209 mg, 1.50 mmol, 1.0 equiv), 1-(*tert*-butyl) 4-methyl 2-methylenesuccinate **2** (300 mg, 1.50 mmol, 1.0 equiv), 4DPAIPN (12.0 mg, 15.0 µmol, 1 mol%), *i*-$Pr_3SiSH$ (32 µL, 29 mg, 0.15 mmol, 10 mol%), and TBAOH•$30H_2O$ (121 mg, 0.15 mmol, 10 mol%) were reacted. Purification *via* flash column chromatography on silica gel (12 g) in $CH_2Cl_2$/MeOH, 0% (5 CV) then 0→5% (25 CV) then 5% (5 CV) then 15% (5 CV), gave **S1** as a colourless solid (114 mg, 83%, 51:49 dr).

Data for **S1**: (Peaks attributed to a diastereomer are denoted by *)

$^1$H NMR: (500 MHz, $CDCl_3$)

7.16–7.09 (m, 4H, C(2′)*H*, C(2′)*H*$^*$, C(6′)*H*, C(6′)*H*$^*$), 7.04–6.97 (m, 4H, C(3′)*H*, C(3′)*H*$^*$, C(5′)*H*, C(5′)*H*$^*$), 6.05 (br s, 1H, N*H*), 6.01 (br s, 1H, N*H*$^*$), 3.69–3.53 (m, 2H, C(6)*H*, C(6)*H*$^*$), 2.90–2.82 (m, 2H, C(4)*H*, ArC$H_A$), 2.79 (dd, $J$ = 13.6, 6.0 Hz, 1H, ArC$H_A{}^*$), 2.72–2.56 (m, 5H, C(4)*H*$^*$, ArC$H_B$, ArC$H_B{}^*$, C(3)$H_A$, C(3)$H_A{}^*$), 2.48–2.36 (m, 2H, C(3)$H_B$, C(3)$H_B{}^*$), 2.21–2.14 (m, 1H, C(5)$H_A$), 2.13–2.05 (m, 1H, C(5)$H_A{}^*$), 1.77–1.69 (m, 1H, C(5)$H_B$), 1.53–1.37 (m, 19H, C(5)$H_B{}^*$, $CO_2CMe_3$, $CO_2CMe_3{}^*$)

$^{13}$C NMR: (126 MHz, $CDCl_3$)

172.6 (*C*$O_2CMe_3$), 172.4 (*C*$O_2CMe_3{}^*$), 170.9 (*C*(2)), 170.8 (*C*(2)$^*$), 162.13 (d, $J$ = 246 Hz, *C*(4′)), 162.06 (d, $J$ = 246 Hz, *C*(4′)$^*$), 132.4 (d, $J$ = 3.3 Hz, *C*(1′)), 132.0 (d, = 3.3 Hz, *C*(1′)$^*$), 130.83 (d, $J$ = 7.9 Hz, *C*(2′), *C*(6′)), 130.78 (d, $J$ = 7.9 Hz, *C*(2′)$^*$, *C*(6′)$^*$), 116.0 (d, $J$ = 21.3 Hz, *C*(3′), *C*(5′)), 115.9 (d, $J$ = 21.3 Hz, *C*(3′)$^*$, *C*(5′)$^*$), 81.51 ($CO_2CMe_3$), 81.46 ($CO_2CMe_3{}^*$), 53.7 (*C*(6)), 51.6 (*C*(6)$^*$), 42.4 (*C*(1′)), 42.3 (*C*(1′)$^*$), 39.1 (*C*(4)), 36.7 (*C*(4)$^*$), 33.9 (*C*(3)), 33.2 (*C*(3)$^*$), 31.9 (*C*(5)), 30.1 (*C*(5)$^*$), 28.10 ($CO_2CMe_3$), 28.11 ($CO_2CMe_3{}^*$)

<u>$^{19}$F NMR:</u> (471 MHz, $CDCl_3$)

–115.5, –115.6

<u>IR:</u> (neat)

3184 (w), 3083 (w), 2975 (w), 2965 (w), 1718 (m), 1660 (m), 1511 (m), 1402 (w), 1390 (w), 1364 (w), 1350 (w), 1292 (w), 1223 (m), 1154 (m), 1092 (m), 847 (w), 820 (m), 787 (w), 754 (w), 657 (w)

<u>MS:</u> ($ESI^+$/QTOF)

331 (17%), 330 ($[M+H]^+$, 100%), 309 (17%), 308 ($[M+H]^+$, 89%)

<u>HRMS:</u> ($ESI^+$/QTOF)

Calcd for $C_{17}H_{23}FNO_3^+$ $[M+H]^+$, 308.1656; found 308.1667

***tert*-Butyl 2-(2-bromobenzyl)-6-oxopiperidine-4-carboxylate (3n)**

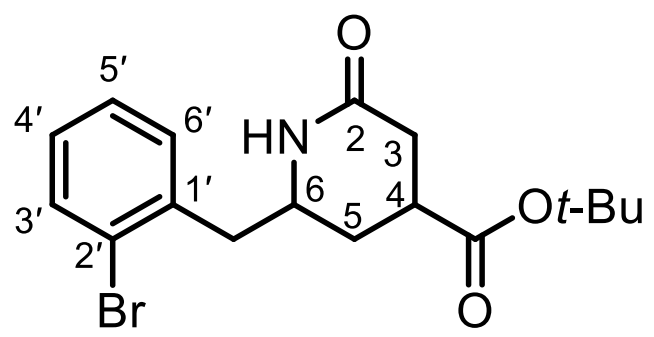


**3n**

Following ***General Procedure 1***, 2-(2-bromophenyl)ethan-1-amine **1n** (87.6 mg, 0.44 mmol, 1.0 equiv), 1-(*tert*-butyl) 4-methyl 2-methylenesuccinate **2** (87.6 mg, 0.44 mmol, 1.0 equiv), 4DPAIPN (3.5 mg, 4.4 µmol, 1 mol%), *i*-$Pr_3SiSH$ (9.5 µL, 8.6 mg, 44 µmmol, 10 mol%), and TBAOH•$30H_2O$ (35.5 mg, 44 µmmol, 10 mol%) were reacted. Purification *via* flash column chromatography on silica gel (12 g) in $CH_2Cl_2$/MeOH, 0% (5 CV) then 0→5% (20 CV) then 5% (5 CV) then 10% (5 CV) then 15% (5 CV), gave **3n** as a yellow solid (122 mg, 75%, 50:50 dr).

<u>Data for **3n**:</u> (Peaks attributed to a diastereomer are denoted by *)

<u>$^1$H NMR:</u> (400 MHz, $CDCl_3$)

7.60–7.52 (m, 2H, C(3′)*H*, C(3′)*H**), 7.30–7.10 (m, 6H, C(4′)*H*, C(4′)*H**, C(5′)*H*, C(5′)*H**, C(6′), C(6′)*), 5.66 (br s, 2H, N*H*, N*H**), 3.85–3.66 (m, 2H, C(6)*H*, C(6)*H**), 3.10–2.98 (m, 2H, ArC*H*), 2.97–2.87 (m, 2H, C(4)*H*), C(4)*H**), 2.84–2.72 (m, 2H, ArC*H**), 2.71–2.39 (m, 4H, C(3)$H_A$, C(3)$H_A$*), 2.26–2.19 (m, 1H, C(5)$H_A$), 2.18–2.08 (m, 1H, C(5)$H_B$), 1.88–1.77 (m, 1H, C(5)$H_B$*), 1.63–1.49 (m, 1H, C(5)$H_A$*), 1.44 (s, 18H, $CO_2CMe_3$, $CO_2CMe_3$*)

<u>$^{13}$C NMR:</u> (101 MHz, $CDCl_3$)

172.58 ($CO_2CMe_3$), 172.46 ($CO_2CMe_3$*), 170.77 (*C*(2)), 170.66 (*C*(2)*), 136.2 (*C*(1′)), 135.8 (*C*(1′)*), 133.6 (*C*(3′)), 133.5 (*C*(3′)*), 131.7 (*C*(6′)), 131.65 (*C*(6′)*), 129.2 (*C*(4′)), 129.1 (*C*(4′)*), 128.1 (*C*(5′)), 128.0 (*C*(5′)*), 124.9 (*C*(2′)), 81.55 ($CO_2CMe_3$), 81.5 ($CO_2CMe_3$*), 51.7 (*C*(6)), 49.85 (*C*(6)*), 43.5 (Ar*C*H), 43.1 (Ar*C*H*), 36.8 (*C*(4)), 33.8 (*C*(3)), 33.2 (*C*(5)), 32.0 (*C*(5)*), 28.1 ($CO_2CMe_3$)

<u>IR:</u> (neat)

2975 (w), 2928 (w), 1724 (s), 1661 (s), 1472 (w), 1445 (w), 1393 (w), 1367 (m), 1345 (w), 1297 (w), 1254 (w), 1153 (s), 1085 (w), 1024 (w), 994 (w), 846 (w), 753 (m), 661 (w), 551 (w), 501 (w), 455 (w)

<u>MS:</u> ($ESI^+$/QTOF)

922 (23%), 167 (38%), 158 (8%), 151 (8%), 126 (8%), 121 (7%), 102 (54%), 82 (42%), 74 (36%), 64 (100%)

<u>HRMS:</u> ($ESI^+$/QTOF)

Calcd for $C_{17}H_{22}BrNO_3Na^+$ $[M+Na]^+$, 390.0675; found, 390.0676

**_tert_-Butyl (4*RS*,6*RS*)- and (4*RS*,6*SR*)-2-oxo-6-(thiophen-2-ylmethyl)piperidine-4-carboxylate (3o)**

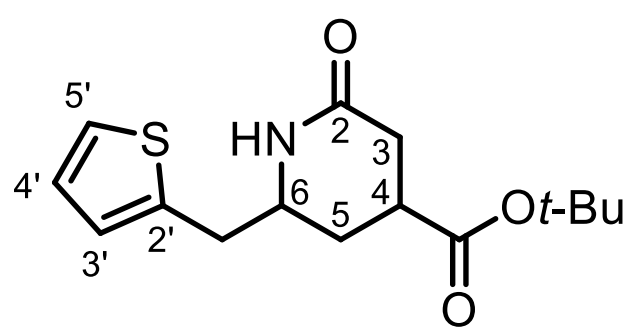


**3o**

Following ***General Procedure 1***, 2-(thiophen-2-yl)ethan-1-amine **1o** (176 µL, 191 mg, 1.50 mmol, 1.0 equiv), 1-(*tert*-butyl) 4-methyl 2-methylenesuccinate **2** (300 mg, 1.50 mmol, 1.0 equiv), 4DPAIPN (12.0 mg, 15.0 µmol, 1 mol%), *i*-$Pr_3SiSH$ (32 µL, 29 mg, 0.15 mmol, 10 mol%), and TBAOH•$30H_2O$ (121 mg, 0.15 mmol, 10 mol%) were reacted. Purification *via* flash column chromatography on silica gel (12 g) in pentane/[EtOAc:EtOH (3:1), $NH_4OH$ (2%)], 0% (3 CV) then 0→20% (27 CV) then 20→100% (17 CV) then 100% (5 CV), followed by reversed-phase flash column chromatography on $C_{18}$ silica gel (15.5 g) in $H_2O$/MeCN, 20% (2 CV) then 20→100% (28 CV) then 100% (8 CV), gave **3o** as a colourless solid (66.9 mg, 50%, 50:50 dr).

<u>Data for **3o**:</u> (Peaks attributed to a diastereomer are denoted by *)

<u>$^1$H NMR:</u> (500 MHz, $CDCl_3$)

7.20–7.16 (m, 2H, C(5′)*H*, C(5′)*H**), 6.96–6.91 (m, 2H, C(4′)*H*, C(4′)*H**), 6.87–6.82 (m, 2H, C(3′)*H*, C(3′)*H**), 6.23 (br s, 1H, N*H*), 6.18 (br s, 1H, N*H**), 3.71–3.57 (m, 2H, C(6)*H*, C(6)*H**), 3.09–2.81 (m, 5H, ArC$H_2$, ArC$H_2$*, C(4)*H*), 2.72–2.63 (m, 1H, C(4)*H**), 2.63–2.56 (m, 2H, C(3)$H_A$, C(3)$H_A$*), 2.47–2.38 (m, 2H, C(3)$H_B$, C(3)$H_B$*), 2.26–2.20 (m, 1H, C(5)$H_A$), 2.17–2.10 (m, 1H, C(5)$H_A$*), 1.79–1.71 (m, 1H, C(5)$H_B$), 1.52–1.36 (m, 19H, C(5)$H_B$*, $CO_2CMe_3$, $CO_2CMe_3$*)

<u>$^{13}$C NMR:</u> (126 MHz, $CDCl_3$)

172.5 (*C*$O_2$CMe$_3$), 172.4 (*C*$O_2$CMe$_3$*), 170.8 (*C*(2)), 170.7 (*C*(2)*), 138.7 (*C*(2′)), 138.3 (*C*(2′)*), 127.4 (*C*(4′)), 127.3 (*C*(4′)*), 126.9 (*C*(3′)), 126.6 (*C*(3′)*), 125.1 (*C*(5′)), 124.9 (*C*(5′)*), 81.5 ($CO_2$*C*Me$_3$), 81.4 ($CO_2$*C*Me$_3$*), 53.8 (*C*(6)), 51.7 (*C*(6)*), 39.0 (*C*(4)), 37.2 (Ar*C*$H_2$), 37.1 (Ar*C*$H_2$*), 36.7 (*C*(4)*), 33.8 (*C*(3)), 33.2 (*C*(3)*), 31.7 (*C*(5)), 30.0 (*C*(5)*), 28.09 ($CO_2$C*Me*$_3$), 28.08 ($CO_2$C*Me*$_3$*)

<u>IR:</u> (neat)

3193 (w), 3072 (w), 2975 (w), 2935 (w), 1723 (m), 1661 (m), 1492 (w), 1436 (w), 1409 (w), 1388 (w), 1366 (w), 1340 (w), 1310 (w), 1294 (w), 1250 (w), 1217 (w), 1158 (m), 1095 (w), 1080 (w), 1014 (w), 932 (w), 884 (w), 843 (m), 819 (w), 696 (m), 663 (w)

<u>MS:</u> ($ESI^+$/QTOF)

319 (17%), 318 ($[M+Na]^+$, 100%), 297 (14%), 296 ($[M+H]^+$, 88%)

<u>HRMS:</u> ($ESI^+$/QTOF)

Calcd for $C_{15}H_{21}NO_3SNa^+$ $[M+Na]^+$, 318.1134; found, 318.1135

***tert*-Butyl (4*RS*,6*RS*)- and (4*RS*,6*SR*)-6-(2-(1*H*-imidazol-1-yl)ethyl)-2-oxopiperidine-4-carboxylate (3p)**

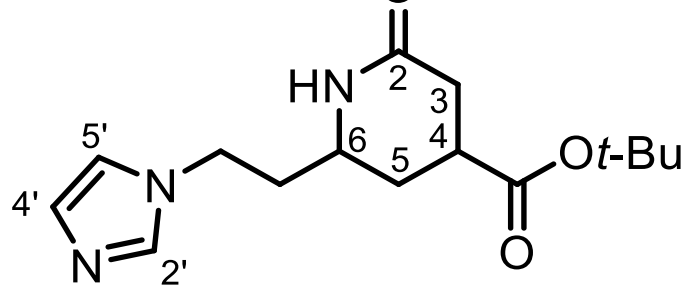


**3p**

Following ***General Procedure 1***, 3-(1*H*-imidazol-1-yl)propan-1-amine **1p** (179 μL, 188 mg, 1.50 mmol, 1.0 equiv), 1-(*tert*-butyl) 4-methyl 2-methylenesuccinate **2** (300 mg, 1.50 mmol, 1.0 equiv),

4DPAIPN (12.0 mg, 15.0 µmol, 1 mol%), *i*-$Pr_3SiSH$ (32 µL, 29 mg, 0.15 mmol, 10 mol%), and TBAOH•$30H_2O$ (121 mg, 0.15 mmol, 10 mol%) were reacted. Purification *via* flash column chromatography on silica gel (12 g) in $CH_2Cl_2$/MeOH, 0% (5 CV) then 0→10% (20 CV) then 10% (10 CV) then 15% (5 CV), gave **3p** as a colourless solid (88 mg, 67%, 53:47 dr).

Data for **3p**: (Peaks attributed to a diastereomer are denoted by *)

$^1$H NMR: (500 MHz, $CDCl_3$)

8.31–8.19 (m, 2H, N*H*, N*H*$^*$), 7.60–7.54 (m, 2H, C(2′)*H*, C(2′)*H*$^*$), 7.06–7.01 (m, 2H, C(4′)*H*, C(4′)*H*$^*$), 6.98–6.93 (m, 2H, C(5′)*H*, C(5′)*H*$^*$), 4.29–4.05 (m, 4H, C(6)$CH_2$C*H*$_2$Ar, C(6)$CH_2$C*H*$_2$Ar$^*$), 3.38–3.23 (m, 2H, C(6)*H*, C(6)*H*$^*$), 2.86–2.77 (m, 1H, C(4)*H*), 2.68–2.52 (m, 3H, C(4)*H*$^*$, C(3)$H_A$, C(3)$H_A{}^*$), 2.47–2.33 (m, 2H, C(3)$H_B$, C(3)$H_B{}^*$), 2.12–1.88 (m, 6H, C(5)$H_A$, C(5)$H_A{}^*$, C(6)C*H*$_2$$CH_2$Ar, C(6)C*H*$_2$$CH_2$Ar$^*$), 1.72–1.63 (m, 1H, C(5)$H_B$), 1.51–1.34 (m, 19H, C(5)$H_B{}^*$, $CO_2$C*Me*$_3$, $CO_2$C*Me*$_3{}^*$)

$^{13}$C NMR: (126 MHz, $CDCl_3$)

172.3, 172.2 (*C*$O_2$CMe$_3$, *C*(2)), 172.0, 171.9 (*C*$O_2$CMe$_3{}^*$, *C*(2)$^*$), 137.4 (*C*(2′)), 137.3 (*C*(2′)$^*$), 129.7 (*C*(4′), *C*(4′)$^*$), 118.7 (*C*(5′), *C*(5′)$^*$), 81.6 ($CO_2$*C*Me$_3$), 81.5 ($CO_2$*C*Me$_3{}^*$), 49.1 (*C*(6)), 47.0 (*C*(6)$^*$), 42.9 (C(6)$CH_2$*C*H$_2$Ar), 42.5 (C(6)$CH_2$*C*H$_2$Ar$^*$), 38.7 (*C*(4)), 38.0 (C(6)*C*H$_2$$CH_2$Ar), 37.9 (C(6)*C*H$_2$$CH_2$Ar$^*$), 36.5 (*C*(4)$^*$), 33.5 (*C*(3)), 33.0 (*C*(3)$^*$), 31.5 (*C*(5)), 30.3 (*C*(5)$^*$), 27.98 ($CO_2$C*Me*$_3$), 27.95 ($CO_2$C*Me*$_3{}^*$)

IR: (neat)

3194 (w), 3089 (w), 2972 (w), 2937 (w), 1717 (w), 1652 (m), 1507 (w), 1457 (w), 1392 (w), 1367 (w), 1335 (w), 1290 (w), 1256 (w), 1226 (w), 1155 (m), 1107 (w), 1079 (w), 1033 (w), 907 (w), 843 (w), 809 (w), 743 (w), 659 (w), 629 (w)

MS: (ESI$^+$/QTOF)

295 (17%), 294 ([M+H]$^+$, 100%)

HRMS: (ESI$^+$/QTOF)

Calcd for $C_{15}H_{24}N_3O_3{}^+$ [M+H]$^+$, 294.1812; found, 294.1814

***tert*-Butyl (4*RS*,6*RS*)- and (4*RS*,6*SR*)-2-oxo-6-(pyridin-2-ylmethyl)piperidine-4-carboxylate (3q)**

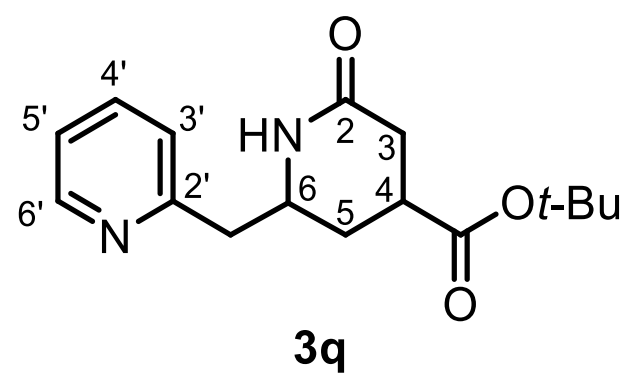


**3q**

Following ***General Procedure 1***, 2-(pyridin-2-yl)ethan-1-amine **1q** (179 µL, 183 mg, 1.50 mmol, 1.0 equiv), 1-(*tert*-butyl) 4-methyl 2-methylenesuccinate **2** (300 mg, 1.50 mmol, 1.0 equiv), 4DPAIPN (12.0 mg, 15.0 µmol, 1 mol%), *i*-$Pr_3SiSH$ (32 µL, 29 mg, 0.15 mmol, 10 mol%), and TBAOH•30$H_2O$ (121 mg, 0.15 mmol, 10 mol%) were reacted. Purification *via* flash column chromatography on silica gel (12 g) in $CH_2Cl_2$/MeOH, 0% (5 CV) then 0→5% (25 CV) then 5% (5 CV) then 15% (5 CV), gave **3q** as a yellow solid (103 mg, 79%, 52:48 dr).

Data for **3q**: (Peaks attributed to a diastereomer are denoted by *)

$^1$H NMR: (500 MHz, $CDCl_3$)

8.51 (d, *J* = 4.8 Hz, 2H, C(6′)*H*, C(6′)*H**), 7.63–7.56 (m, 2H, C(4′)*H*, C(4′)*H**), 7.18–7.02 (m, 5H, C(3′)*H*, C(3′)*H**, C(5′)*H*, C(5′)*H**, N*H*), 6.83 (br s, 1H, N*H**), 4.03–3.95 (m, 1H, C(6)*H*), 3.90–3.82 (m, 1H, C(6)*H**), 2.99–2.81 (m, 5H, PyC$H_2$, PyC$H_2$*, C(4)*H*), 2.74–2.65 (m, 1H, C(4)*H**), 2.64–2.55 (m, 2H, C(3)$H_A$, C(3)$H_A$*), 2.50–2.38 (m, 2H, C(3)$H_B$, C(3)$H_B$*), 2.24–2.11 (m, 2H, C(5)$H_A$, C(5)$H_A$*), 1.84–1.77 (m, 1H, C(5)$H_B$), 1.61–1.51 (m, 1H, C(5)$H_B$*), 1.45–1.40 (m, 18H, $CO_2$C*Me*$_3$, $CO_2$C*Me*$_3$*)

$^{13}$C NMR: (126 MHz, $CDCl_3$)

172.7 (*C*$O_2$CMe$_3$), 172.5 (*C*$O_2$CMe$_3$*), 170.4 (*C*(2)), 170.3 (*C*(2)*), 157.80 (*C*(2′)), 157.77 (*C*(2′)*), 149.6 (*C*(6′)), 149.5 (*C*(6′)*), 136.9 (*C*(4′)), 136.8 (*C*(4′)*), 123.77, 123.75 (*C*(3′), *C*(5′)), 122.1, 122.0 (*C*(3′)*, *C*(5′)*), 81.4 ($CO_2$*C*Me$_3$), 81.3 ($CO_2$*C*Me$_3$*), 52.0 (*C*(6)), 49.7 (*C*(6)*), 44.1 (Py*C*$H_2$), 44.0 (Py*C*$H_2$*), 39.1 (*C*(4)), 36.9 (*C*(4)*), 33.7 (*C*(3)), 33.2 (*C*(3)*), 32.3 (*C*(5)), 30.6 (*C*(5)*), 28.07 ($CO_2$C*Me*$_3$), 28.06 ($CO_2$C*Me*$_3$*)

IR: (neat)

3185 (w), 3075 (w), 2976 (w), 1723 (m), 1662 (m), 1589 (w), 1568 (w), 1474 (w), 1433 (w), 1412 (w), 1388 (w), 1365 (w), 1298 (w), 1253 (w), 1222 (w), 1156 (m), 1093 (w), 1048 (w), 994 (w), 932 (w), 846 (w), 781 (w), 761 (w), 748 (w), 666 (w)

MS: ($ESI^+$/QTOF)

313 (20%), 292 (17%), 291 ($[M+H]^+$, 100%)

HRMS: (ESI$^+$/QTOF)

Calcd for $C_{16}H_{22}N_2O_3Na^+$ [M+Na]$^+$, 313.1523; found, 313.1526

**_tert_-Butyl (4_RS_,6_RS_)- and (4_RS_,6_SR_)-6-(3-methyloxetan-3-yl)-6-oxopiperidine-4-carboxylate (3r)**

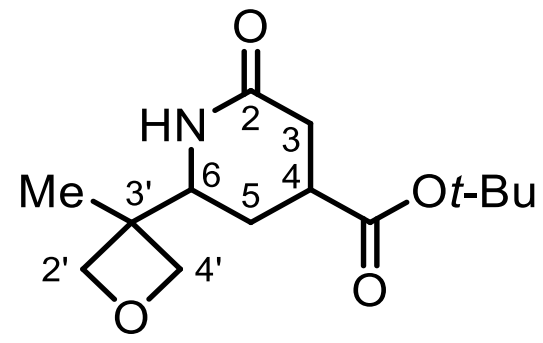


**3r**

Following ***General Procedure 1***, (3-methyloxetan-3-yl)methanamine **1r** (158 µL, 152 mg, 1.50 mmol, 1.0 equiv), 1-(*tert*-butyl) 4-methyl 2-methylenesuccinate **2** (300 mg, 1.50 mmol, 1.0 equiv), 4DPAIPN (12.0 mg, 15.0 µmol, 1 mol%), *i*-$Pr_3SiSH$ (32 µL, 29 mg, 0.15 mmol, 10 mol%), and TBAOH•30$H_2O$ (121 mg, 0.15 mmol, 10 mol%) were reacted. Purification *via* flash column chromatography on silica gel (12 g) in pentane/[EtOAc:EtOH (3:1), $NH_4OH$ (2%)], 0% (3 CV) then 0→20% (25 CV) then 20→100% (18 CV) then 100% (4 CV), followed by reversed-phase flash column chromatography on $C_{18}$ silica gel (15.5 g) in $H_2O$/MeCN, 10% (2 CV) then 10→100% (28 CV) then 100% (8 CV), gave **3r** as a colourless solid (73.8 mg, 61%, 50:50 dr).

Data for **3r**: (Peaks attributed to a diastereomer are denoted by *)

$^1$H NMR: (500 MHz, $CDCl_3$)

7.31 (br s, 1H, N*H*), 7.13 (br s, 1H, N*H*$^*$), 4.53–4.49 (m, 2H, C(2′)$H_A$, C(2′)$H_A{}^*$), 4.47–4.40 (m, 2H, C(4′)$H_A$, C(4′)$H_A{}^*$), 4.39–4.30 (m, 4H, C(2′)$H_B$, C(2′)$H_B{}^*$, C(4′)$H_B$, C(4′)$H_B{}^*$), 3.82–3.73 (m, 2H, C(6)*H*, C(6)*H*$^*$), 2.87–2.81 (m, 1H, C(4)*H*), 2.71–2.61 (m, 2H, C(3)$H_A$, C(4)*H*$^*$), 2.61–2.54 (ddd, $J$ = 17.6, 5.4, 2.1 Hz, 1H, C(3)$H_A{}^*$), 2.38–2.28 (m, 2H, C(3)$H_B$, C(3)$H_B{}^*$), 2.07–1.93 (m, 2H, C(5)$H_A$, C(5)$H_A{}^*$), 1.57–1.36 (m, 19H, $CO_2CMe_3$, $CO_2CMe_3{}^*$, C(5)$H_B$), 1.35–1.24 (m, 7H, C(3′)*Me*, C(3′)*Me*$^*$, C(5)$H_B{}^*$)

$^{13}$C NMR: (126 MHz, $CDCl_3$)

172.48 (*C*$O_2CMe_3$), 172.47 (*C*$O_2CMe_3{}^*$), 172.1 (*C*(2)), 172.0 (*C*(2)$^*$), 81.6 ($CO_2CMe_3$), 81.5 ($CO_2CMe_3{}^*$), 80.8, 80.6 (*C*(2′), *C*(4′)), 79.2, 79.1 (*C*(2′)$^*$, *C*(4′)$^*$), 58.0 (*C*(6)), 55.3 (*C*(6)$^*$), 42.23 (*C*(3′)), 42.21 (*C*(3′)$^*$), 38.8 (*C*(4)), 37.0 (*C*(4)$^*$), 33.8 (*C*(3)), 32.6 (*C*(3)$^*$), 28.10 ($CO_2CMe_3$), 28.05 ($CO_2CMe_3{}^*$), 26.1 (*C*(5)), 25.3 (*C*(5)$^*$), 18.8 (C(3′)*Me*), 18.4 (C(3′)*Me*$^*$)

IR: (neat)

3202 (w), 3085 (w), 2967 (w), 2922 (w), 2866 (w), 1719 (m), 1650 (m), 1459 (w), 1407 (w), 1391 (w), 1382 (w), 1368 (w), 1325 (w), 1303 (w), 1259 (w), 1230 (w), 1216 (w), 1147 (m), 1094 (w), 1071 (w), 981 (w), 970 (m), 841 (m), 777 (w), 757 (w), 659 (w)

MS: ($ESI^+$/QTOF)

292 ($[M+Na]^+$, 43%), 271 (15%), 270 ($[M+H]^+$, 100%)

HRMS: ($ESI^+$/QTOF)

Calcd for $C_{14}H_{24}NO_4^+$ $[M+H]^+$, 270.1700; found, 270.1700

***tert*-Butyl (4*RS*,6*RS*)- and (4*RS*,6*SR*)-6-(1-(*tert*-butoxycarbonyl)azetidin-3-yl)-2-oxopiperidine-4-carboxylate (3s)**

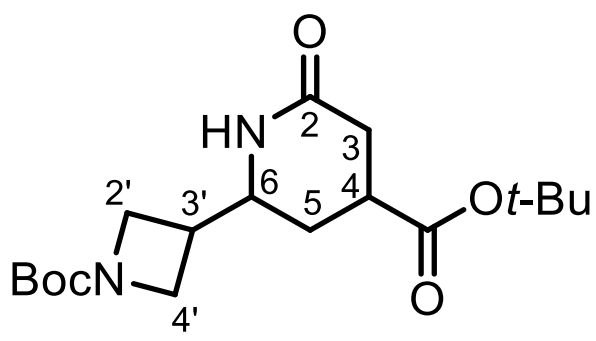


**3s**

Following ***General Procedure 1***, *tert*-butyl 3-(aminomethyl)azetidine-1-carboxylate **1s** (273 µL, 279 mg, 1.50 mmol, 1.0 equiv), 1-(*tert*-butyl) 4-methyl 2-methylenesuccinate **2** (300 mg, 1.50 mmol, 1.0 equiv), 4DPAIPN (12.0 mg, 15.0 µmol, 1 mol%), *i*-$Pr_3SiSH$ (32 µL, 29 mg, 0.15 mmol, 10 mol%), and TBAOH•$30H_2O$ (121 mg, 0.15 mmol, 10 mol%) were reacted. Purification *via* flash column chromatography on silica gel (12 g) in pentane/[EtOAc:EtOH (3:1), $NH_4OH$ (2%)], 0% (3 CV) then 0→20% (22 CV) then 20→100% (18 CV) then 100% (7 CV), followed by reversed-phase flash column chromatography on $C_{18}$ silica gel (15.5 g) in $H_2O$/MeCN, 10% (2 CV) then 10→100% (30 CV) then 100% (8 CV), gave **3s** as a colourless solid (64.6 mg, 41%, 53:47 dr).

Data for **3s**: (Peaks attributed to a diastereomer are denoted by *)

$^1H$ NMR: (500 MHz, $CDCl_3$)

6.93–6.74 (m, 2H, N*H*, N*H**), 4.04–3.91 (m, 4H, C(2′)$H_A$, C(2′)$H_A^*$, C(4′)$H_A$, C(4′)$H_A^*$), 3.76–3.69 (m, 2H, C(2′)$H_B$ or C(4′)$H_B$), 3.68–3.63 (dd, *J* = 5.6, 9.0 Hz, 1H, C(2′)$H_B$ or C(4′)$H_B$), 3.62–3.52 (m, 3H, [C(2′)$H_B^*$ or C(4′)$H_B^*$], [C(2′)$H_B^*$ or C(4′)$H_B^*$], C(6)*H*, C(6)*H**), 2.82–2.75 (m, 1H, C(4)*H*), 2.71–2.48 (m, 5H, C(4)*H**, C(3′)*H*, C(3′)*H**, C(3)$H_A$, C(3)$H_A^*$), 2.48–2.33 (m, 2H, C(3)$H_B$, C(3)$H_B^*$), 2.15–2.00 (m, 1H, C(5)$H_A$, C(5)$H_A^*$),

1.64–1.56 (m, 1H, C(5)$H_B$), 1.52–1.20 (m, 13H, C(5)$H_B$*, C(4)$CO_2CMe_3$, C(4)$CO_2CMe_3$*, $NCO_2CMe_3$, $NCO_2CMe_3$*)

<u>$^{13}$C NMR:</u> (126 MHz, $CDCl_3$)

172.4 (C(4)$CO_2CMe_3$), 172.3 (C(4)$CO_2CMe_3$*), 171.5 (*C*(2)), 171.2 (*C*(2)*), 156.2 ($NCO_2CMe_3$, $NCO_2CMe_3$*), 81.7, 81.6 (C(4)$CO_2CMe_3$, $NCO_2CMe_3$), 79.86, 79.84 (C(4)$CO_2CMe_3$*, $NCO_2CMe_3$*), 54.7 (*C*(6)), 52.9 (*C*(6)*), 50.9 (br, *C*(2′), *C*(2′)*, *C*(4′), *C*(4′)*), 38.7 (*C*(4)), 36.4 (*C*(4)*), 33.9 (*C*(3′), *C*(3′)*), 33.7 (*C*(3)), 33.1 (*C*(3)*), 28.9 (br, *C*(5)), 28.5 ($NCO_2CMe_3$, $NCO_2CMe_3$*), 28.10 (C(4)$CO_2CMe_3$), 28.08 (C(4)$CO_2CMe_3$*), 27.5 (br, *C*(5)*)

<u>IR:</u> (neat)

3203 (w), 3086 (w), 2977 (w), 2943 (w), 2249 (w), 2170 (w), 1723 (w), 1687 (m), 1655 (m), 1477 (w), 1417 (w), 1367 (m), 1326 (w), 1295 (w), 1252 (w), 1210 (w), 1147 (m), 1083 (w), 971 (w), 921 (w), 863 (w), 842 (w), 767 (w), 728 (m)

<u>MS:</u> ($ESI^+$/QTOF)

731 ($[2M+Na]^+$12%), 378 (21%), 377 ($[M+Na]^+$, 100%), 300 (10%), 299 (66%), 243 (50%), 142 (14%), 126 (30%), 85 (10%)

<u>HRMS:</u> ($ESI^+$/QTOF)

Calcd for $C_{18}H_{30}N_2O_5Na^+$ $[M+Na]^+$, 377.2047; found 377.2049

**tert-Butyl (4*RS*,6*RS*)- and (4*RS*,6*SR*)-2-oxo-6-(2,2,2-trifluoroethyl)piperidine-4-carboxylate (3t)**

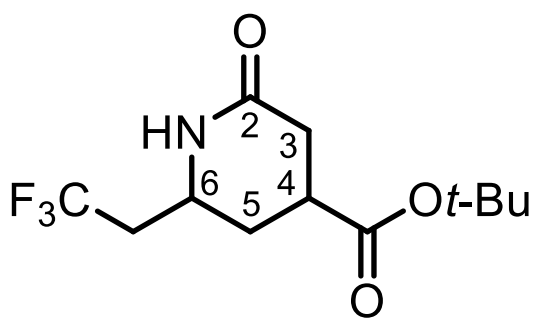


**3t**

Following ***General Procedure 1***, 3,3,3-trifluoropropan-1-amine **1t** (147 µL, 170 mg, 1.50 mmol, 1.0 equiv), 1-(*tert*-butyl) 4-methyl 2-methylenesuccinate **2** (300 mg, 1.50 mmol, 1.0 equiv), 4DPAIPN (12.0 mg, 15.0 µmol, 1 mol%), *i*-$Pr_3SiSH$ (32 µL, 29 mg, 0.15 mmol, 10 mol%), and TBAOH•30$H_2O$ (121 mg, 0.15 mmol, 10 mol%) were reacted. Purification *via* flash column chromatography on silica gel (12 g) in pentane/[EtOAc:EtOH (3:1), $NH_4OH$ (2%)], 0% (2 CV) then 0→20% (24 CV) then 20→100% (18 CV) then 100% (8 CV), followed by reversed-phase flash column chromatography on $C_{18}$ silica gel (15.5 g) in $H_2O$/MeCN, 5% (2 CV) then 5→100% (30 CV) then 100% (10 CV), gave **3t** as a colourless solid (61.6 mg, 49%, 53:47 dr).

Data for **3t**: (Peaks attributed to a diastereomer are denoted by *)

$^{1}$H NMR: (500 MHz, $CDCl_3$)

6.59–6.26 (m, 2H, N*H* and N*H*$^{*}$), 3.90–3.82 (m, 1H, C(6)*H*), 3.82–3.73 (m, 1H, C(6)*H*$^{*}$), 2.89–2.81 (m, 1H, C(4)*H*), 2.76–2.67 (m, 1H, C(4)*H*$^{*}$), 2.67–2.59 (m, 2H, C(3)$H_A$, C(3)$H_A{}^{*}$), 2.51–2.40 (m, 2H, C(3)$H_B$, C(3)$H_B{}^{*}$), 2.40–2.17 (m, 6H, $CF_3CH_2$, $CF_3CH_2{}^{*}$, C(5)$H_A$, C(5)$H_A{}^{*}$), 1.80 (ddd, *J* = 13.7, 8.0, 3.9 Hz, 1H, C(5)$H_B$), 1.60–1.49 (m, 1H, C(5)$H_B{}^{*}$), 1.49–1.38 (m, 18H, $CO_2CMe_3$, $CO_2CMe_3{}^{*}$)

$^{13}$C NMR: (126 MHz, $CDCl_3$)

172.1 ($CO_2CMe_3$), 171.9 ($CO_2CMe_3{}^{*}$), 170.8 (*C*(2)), 170.6 (*C*(2)$^{*}$), 125.84 (q, *J* = 277.2 Hz, $CF_3$), 125.80 (q, *J* = 277.0 Hz, $CF_3{}^{*}$), 82.0 ($CO_2CMe_3$), 81.8 ($CO_2CMe_3{}^{*}$), 47.2 (q, *J* = 2.9 Hz, *C*(6)), 45.2 (q, *J* = 2.8 Hz, *C*(6)$^{*}$), 40.7 (q, *J* = 27.4 Hz, $CF_3CH_2$), 40.8 (q, *J* = 27.6 Hz, $CF_3CH_2{}^{*}$), 38.8 (*C*(4)), 36.6 (*C*(4)$^{*}$), 33.5 (*C*(3)), 32.9 (*C*(3)$^{*}$), 31.7 (*C*(5)), 30.4 (*C*(5)$^{*}$), 28.10 ($CO_2CMe_3$), 28.08 ($CO_2CMe_3{}^{*}$)

$^{19}$F NMR: (471 MHz, $CDCl_3$):

–63.4, –63.6

IR: (neat)

3199 (w), 3072 (w), 2981 (w), 2938 (w), 1725 (m), 1672 (m), 1499 (w), 1453 (w), 1387 (m), 1369 (m), 1308 (w), 1254 (m), 1211 (w), 1155 (m), 1121 (m), 1101 (m), 1040 (w), 997 (w), 963 (w), 897 (w), 842 (m), 748 (w), 686 (w), 638 (m)

MS: ($ESI^+$/QTOF)

305 (13%), 304 ($[M+Na]^+$, 100%), 282 ($[M+H]^+$, 63%)

HRMS: ($ESI^+$/QTOF)

Calcd for $C_{12}H_{19}F_3NO_3{}^+$ $[M+H]^+$, 282.1312; found, 282.1311

***tert*-Butyl (4*RS*,6*RS*)- and (4*RS*,6*SR*)-6-(cyanomethyl)-2-oxopiperidine-4-carboxylate (3u)**

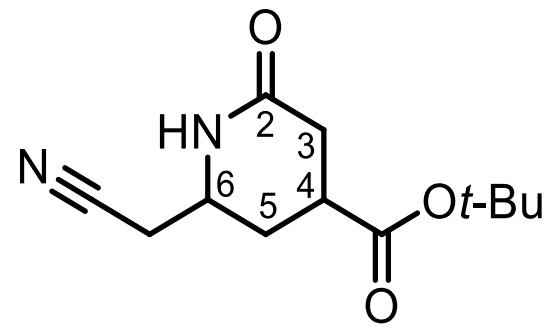


**3u**

Following ***General Procedure 1***, 3-aminopropanenitrile **1u** (110 µL, 105 mg, 1.50 mmol, 1.0 equiv), 1-(*tert*-butyl) 4-methyl 2-methylenesuccinate **2** (300 mg, 1.50 mmol, 1.0 equiv), 4DPAIPN (12.0 mg,

15.0 µmol, 1 mol%), *i*-$Pr_3SiSH$ (32 µL, 29 mg, 0.15 mmol, 10 mol%), and TBAOH•$30H_2O$ (121 mg, 0.15 mmol, 10 mol%) were reacted. Purification *via* flash column chromatography on silica gel (12 g) in pentane/[EtOAc:EtOH (3:1), $NH_4OH$ (2%)], 0% (3 CV) then 0→30% (22 CV) then 30→100% (18 CV) then 100% (7 CV), followed by reversed-phase flash column chromatography on $C_{18}$ silica gel (15.5 g) in $H_2O$/MeCN, 10% (2 CV) then 10→100% (30 CV) then 100% (8 CV), gave **3u** as a colourless solid (21.1 mg, 20%, 53:47 dr).

Data for **3u**: (Peaks attributed to a diastereomer are denoted by *)

$^1$H NMR: (500 MHz, $CDCl_3$)

7.59 (br s, 1H, N*H*), 7.43 (br s, 1H, N*H*$^*$), 3.87–3.79 (m, 1H, C(6)*H*), 3.77–3.68 (m, 1H, C(6)*H*$^*$), 2.96–2.87 (m, 1H C(4)*H*), 2.78–2.67 (m, 1H, C(4)*H*$^*$), 2.66–2.55 (m, 6H, C$H_2$CN, C$H_2$CN$^*$, C(3)$H_A$, C(3)$H_A{}^*$), 2.52–2.39 (m, 2H, C(3)$H_B$, C(3)$H_B{}^*$), 2.39–2.30 (m, 1H, C(5)$H_A$), 2.29–2.20 (m, 1H, C(5)$H_A{}^*$), 1.94–1.86 (m, 1H C(5)$H_B$), 1.68–1.57 (m, 1H, C(5)$H_B{}^*$), 1.53–1.37 (m, 18H, $CO_2$C*Me*$_3$, $CO_2$C*Me*$_3{}^*$)

$^{13}$C NMR: (126 MHz, $CDCl_3$)

172.0, 171.8 (*C*$O_2$CMe$_3$, *C*(2)), 171.6, 171.5 (*C*$O_2$CMe$_3{}^*$, *C*(2)$^*$), 116.4 (C$H_2$*C*N), 116.1 (C$H_2$*C*N$^*$), 82.1 ($CO_2$*C*Me$_3$), 81.9 ($CO_2$*C*Me$_3{}^*$), 48.8 (*C*(6)), 46.9 (*C*(6)$^*$), 38.5 (*C*(4)), 36.4 (*C*(4)$^*$), 33.4 (*C*(3)), 32.9 (*C*(3)$^*$), 30.8 (*C*(5)), 29.2 (*C*(5)$^*$), 28.1 ($CO_2$C*Me*$_3$, $CO_2$C*Me*$_3{}^*$), 25.11 (C$H_2$*C*N)), 25.09 (C$H_2$*C*N$^*$)

IR: (neat)

3188 (w), 3083 (w), 2970 (w), 2933 (w), 2251 (w), 2165 (w), 1718 (m), 1667 (m), 1492 (w), 1475 (w), 1459 (w), 1409 (w), 1391 (m), 1365 (w), 1338 (m), 1308 (m), 1256 (w), 1233 (w), 1214 (w), 1160 (m), 1078 (w), 1001 (w), 964 (w), 924 (w), 904 (w), 842 (m), 776 (w), 735 (w), 671 (w)

MS: (ESI$^+$/QTOF)

262 (14%), 261 ($[M+Na]^+$, 100%), 239 ($[M+H]^+$, 32%)

HRMS: (ESI$^+$/QTOF)

Calcd for $C_{12}H_{18}N_2O_3Na^+$ $[M+Na]^+$, 261.1210; found, 261.1216

***tert*-Butyl (4*RS*,6*RS*)- and (4*RS*,6*SR*)-2-oxo-6-(trimethylsilyl)piperidine-4-carboxylate (S2)**

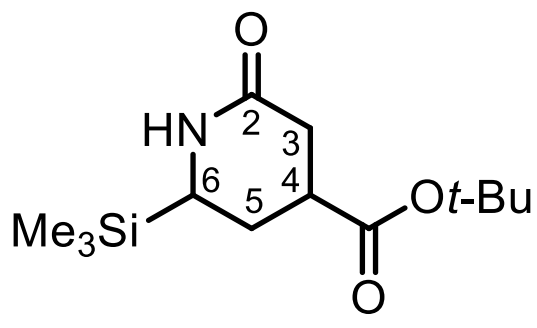


**S2**

Following ***General Procedure 1***, (trimethylsilyl)methanamine (201 µL, 155 mg, 1.50 mmol, 1.0 equiv), 1-(*tert*-butyl) 4-methyl 2-methylenesuccinate **2** (300 mg, 1.50 mmol, 1.0 equiv), 4DPAIPN (12.0 mg, 15.0 µmol, 1 mol%), *i*-$Pr_3SiSH$ (32 µL, 29 mg, 0.15 mmol, 10 mol%), and TBAOH•$30H_2O$ (121 mg, 0.15 mmol, 10 mol%) were reacted. Purification *via* flash column chromatography on silica gel (12 g) in pentane/[EtOAc:EtOH (3:1), $NH_4OH$ (2%)], 0% (2 CV) then 0→20% (20 CV) then 20→100% (29 CV) then 100% (8 CV), followed by reversed-phase flash column chromatography on $C_{18}$ silica gel (15.5 g) in $H_2O$/MeCN, 5% (2 CV) then 5→100% (30 CV) then 100% (16 CV), gave **S2** as a colourless solid (35 mg, 29%, 59:41 dr).

Data for **S2**: (Peaks attributed to a diastereomer are denoted by *)

$^1$H NMR: (500 MHz, $CDCl_3$)

5.66 (br s, 1H, N*H*), 5.61 (br s, 1H, N*H*$^*$), 2.91–2.82 (m, 3H, C(6)*H*, C(6)*H*$^*$, C(4)*H*), 2.74–2.57 (m, 3H, C(4)*H*$^*$, C(3)$H_A$, C(3)$H_A{}^*$), 2.48–2.35 (m, 2H, C(3)$H_B$, C(3)$H_B{}^*$), 2.16–2.04 (m, 1H, C(5)$H_A$), 1.79–1.73 (m, 1H, C(5)$H_A{}^*$), 1.61–1.50 (m, 2H, C(5)$H_B$, C(5)$H_B{}^*$), 1.49–1.37 (m, 18H, $CO_2CMe_3$, $CO_2CMe_3{}^*$), 0.12–0.00 (m, 18H, Si*Me*$_3$, Si*Me*$_3{}^*$)

$^{13}$C NMR: (126 MHz, $CDCl_3$)

172.89 (*C*$O_2$CMe$_3$), 172.94 (*C*$O_2$CMe$_3{}^*$), 171.28 (*C*(2)), 171.31 (*C*(2)$^*$), 81.3 ($CO_2$*C*Me$_3$), 77.4 ($CO_2$*C*Me$_3{}^*$), 43.5 (*C*(6)), 41.0 (*C*(4)), 40.7 (*C*(6)$^*$), 38.6 (*C*(4)$^*$), 33.9 (*C*(3)), 32.9 (*C*(3)$^*$), 28.17 ($CO_2$C*Me*$_3$), 28.15 ($CO_2$C*Me*$_3{}^*$), 27.0 (*C*(5)), 26.4 (*C*(5)$^*$), –4.3 (Si*Me*$_3$), –4.4 (Si*Me*$_3{}^*$)

IR: (neat)

3194 (w), 3054 (w), 2974 (w), 2905 (w), 1719 (m), 1641 (m), 1403 (w), 1369 (w), 1302 (w), 1251 (m), 1147 (m), 1102 (w), 1063 (w), 974 (w), 838 (m), 756 (w), 726 (w), 698 (w), 625 (w)

MS: (ESI$^+$/QTOF)

295 (19%), 294 ([M+Na]$^+$, 99%), 273 (19%), 272 ([M+H]$^+$, 100%)

HRMS: (ESI+/QTOF)

Calcd for $C_{13}H_{25}NO_3SiNa^+$ [M+Na]+, 294.1501; found, 294.1497

**tert-Butyl (4RS,6RS)- and (4RS,6SR)-6-(hydroxymethyl)-2-oxopiperidine-4-carboxylate (3v)**

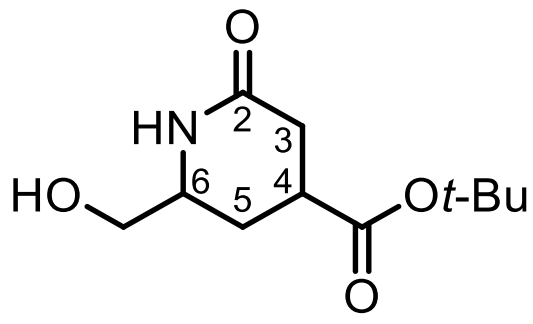

**3v**

Following ***General Procedure 1***, ethanolamine **1v** (816 µL, 825 mg, 13.5 mmol, 1.0 equiv), 1-(*tert*-butyl) 4-methyl 2-methylenesuccinate **2** (300 mg, 1.50 mmol, 1.0 equiv), 4DPAIPN (12.0 mg, 15.0 µmol, 1 mol%), *i*-$Pr_3SiSH$ (32 µL, 29 mg, 0.15 mmol, 10 mol%), and TBAOH•$30H_2O$ (121 mg, 0.15 mmol, 10 mol%) were reacted. Purification *via* flash column chromatography on silica gel (12 g) in pentane/[EtOAc:EtOH (3:1), $NH_4OH$ (2%)], 0% (3 CV) then 0→20% (22 CV) then 20→100% (18 CV) then 100% (7 CV), followed by reversed-phase flash column chromatography on $C_{18}$ silica gel (15.5 g) in $H_2O$/MeCN, 10% (2 CV) then 10→100% (30 CV) then 100% (8 CV), gave **3v** as a colourless solid (83 mg, 80%, 70:30 dr).

Data for **3v**: (Peaks attributed to a diastereomer are denoted by *)

$^1$H NMR: (400 MHz, $CDCl_3$)

7.30 (s, 1H, N*H*), 7.21 (s, 1H, N*H*$^*$) 4.16 (s, 2H, O*H*, O*H*$^*$), 3.77–3.59 (m, 2H, C$H_A$$H_B$OH, C$H_A$$H_B$OH$^*$), 3.59–3.36 (m, 4H, C$H_A$$H_B$OH, C$H_A$$H_B$OH$^*$ and C(6)*H*, C(6)*H*$^*$), 2.92–2.70 (m, 1H, C(4)*H*)), 2.77–2.64 (m, 1H, C(4)*H*$^*$), 2.64–2.52 (m, 2H, C(3)$H_A$, C(3)$H_A$$^*$), 2.49–2.29 (m, 2H, C(3)$H_B$, C(3)$H_B$$^*$), 2.15–1.94 (m, 2H, C(5)$H_2$), 1.80–1.67 (m, 1H, C(5)$H_A$$^*$), 1.43 (app s, 19H, $CO_2CMe_3$, $CO_2CMe_3$$^*$ and C(5)$H_B$$^*$)

$^{13}$C NMR: (101 MHz, $CDCl_3$)

172.8, 172.5 (*C*(2), *C*$O_2CMe_3$), 172.1, 172.0 (*C*(2)$^*$, *C*$O_2CMe_3$$^*$), 81.7 ($CO_2$*C*$Me_3$), 81.5 ($CO_2$*C*$Me_3$$^*$), 65.8 (*C*$H_2$OH, *C*$H_2$OH*), 54.3 (*C*(6)), 52.0 (*C*(6)$^*$), 38.6 (*C*(4)), 36.8 (*C*(4)$^*$), 33.6 (*C*(3)), 33.0 (*C*(3)$^*$), 28.1 ($CO_2C$*$Me_3$*, $CO_2C$*$Me_3$**), 27.6 (*C*(5)), 26.5 (*C*(5)$^*$)

IR: (neat)

3320 (w), 3201 (w), 2981 (w), 2934 (w), 2873 (w), 1723 (s), 1668 (s), 1366 (m), 1240 (m), 1149 (s), 843 (w)

HRMS: (ESI+/QTOF)

Calcd for $C_{11}H_{19}NO_4Na^+$ [M+Na]+, 252.1206; found, 252.1208

***tert*-Butyl (4*RS*,6*RS*)- and (4*RS*,6*SR*)-2-(1′,1′-diethoxypropyl)-6-oxopiperidine-4-carboxylate (S3)**

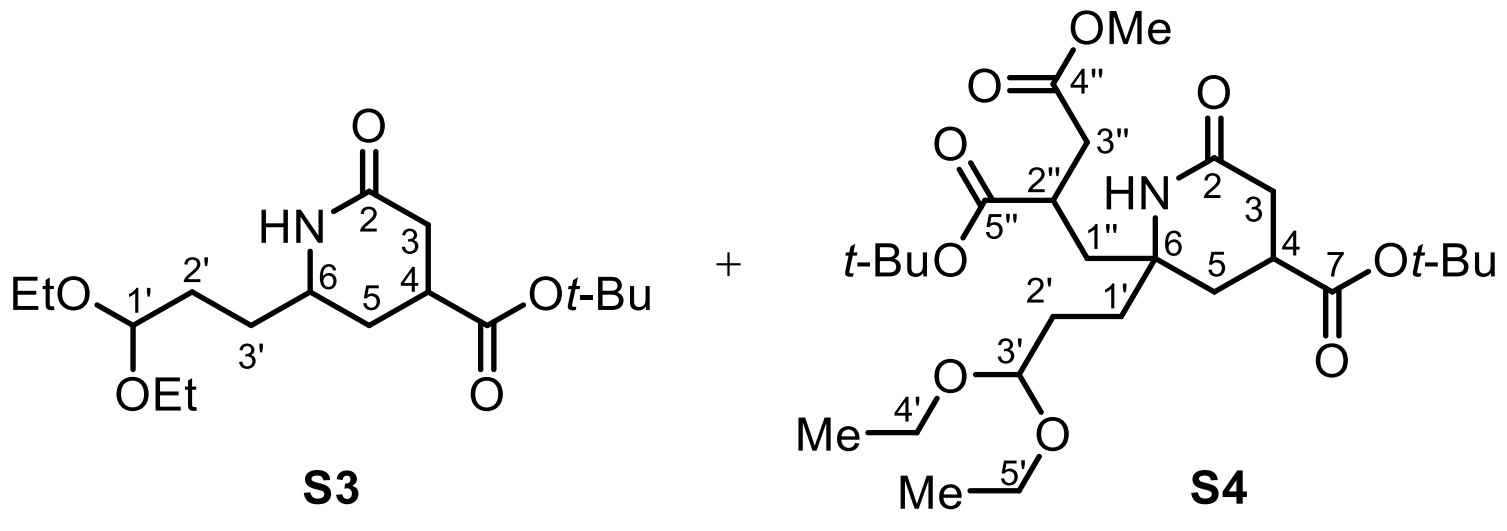


*dialkylation by-product*

Following ***General Procedure 1***, 4,4-diethoxybutan-1-amine (259 µL, 242 mg, 1.50 mmol, 1.0 equiv), 1-(*tert*-butyl) 4-methyl 2-methylenesuccinate **2** (300 mg, 1.50 mmol, 1.0 equiv), 4DPAIPN (12.0 mg, 15.0 µmol, 1 mol%), *i*-$Pr_3SiSH$ (32 µL, 29 mg, 0.15 mmol, 10 mol%), and TBAOH•$30H_2O$ (121 mg, 0.15 mmol, 10 mol%) were reacted. Purification *via* flash column chromatography on silica gel (12 g) in pentane/[EtOAc:EtOH (3:1), $NH_4OH$ (2%)], 0% (3 CV) then 0→30% (22 CV) then 30→100% (18 CV) then 100% (7 CV), followed by reversed-phase flash column chromatography on $C_{18}$ silica gel (15.5 g) in $H_2O$/MeCN, 10% (2 CV) then 10→100% (30 CV) then 100% (8 CV), gave **S3** as a colourless solid (48.5 mg, 33%, 50:50 dr) and dialkylated product **S4** as a colourless gum (43.7 mg, 18%, 20:24:28:28 dr).

Data for **S3**: (Peaks attributed to a diastereomer are denoted by *)

$^1$H NMR: (500 MHz, $CDCl_3$)

6.40–6.28 (m, 2H, N*H*, N*H**), 4.48–4.41 (m, 2H, C(1′)*H*, C(1′)*H**), 3.68–3.57 (m, 4H, OC*H*$_2$Me, OC*H*$_2$Me*), 3.51–3.34 (m, 6H, OC*H*$_2$Me, OC*H*$_2$Me*, C(6)*H*, C(6)*H**), 2.85–2.77 (m, 1H, C(4)*H*), 2.68–2.50 (m, 3H, C(4)*H**, C(3)*H*$_A$, C(3)*H*$_A$*), 2.46–2.33 (m, 2H, C(3)*H*$_B$, C(3)*H*$_B$*), 2.18–2.11 (m, 1H, C(5)*H*$_A$), 2.09–2.01 (m, 1H, C(5)*H*$_A$*), 1.73–1.50 (m, 9H, C(3′)*H*$_2$, C(2′)*H*$_2$, C(3′)*H*$_2$*, C(2′)*H*$_2$*, C(5)*H*$_B$), 1.47–1.32 (m, 19H, C(5)*H*$_B$*, $CO_2$C*Me*$_3$, $CO_2$C*Me*$_3$*), 1.22–1.14 (m, 12H, C(1′)(OCH$_2$*Me*)$_2$, C(1′)(OCH$_2$*Me*)$_2$*)

<u>$^{13}$C NMR:</u> (126 MHz, $CDCl_3$)

172.8 ($CO_2CMe_3$), 172.6 ($CO_2CMe_3$*), 171.0 (*C*(2)), 170.8 (*C*(2)*), 102.60 (*C*(1′)), 102.55 (*C*(1′)*), 81.4 ($CO_2CMe_3$), 81.3 ($CO_2CMe_3$*), 61.8 (O*C*$H_2$Me), 61.7 (O*C*$H_2$Me), 61.61 (O*C*$H_2$Me*), 61.56 (O*C*$H_2$Me*), 52.4 (*C*(6)), 50.0 (*C*(6)*), 39.1 (*C*(4)), 36.6 (*C*(4)*), 33.7 (*C*(3)), 33.2 (*C*(3)*), 31.8, 31.7, 31.6 (*C*(5), *C*(3′), *C*(2′)), 30.2, 29.8, 29.5 (*C*(5)*, *C*(3′)*, *C*(2′)*), 28.1 ($CO_2CMe_3$, $CO_2CMe_3$*), 15.4 (C(1′)($OCH_2Me)_2$), 15.4 (C(1′)($OCH_2Me)_2$*)

<u>IR:</u> (neat)

3207 (w), 3087 (w), 2976 (w), 2929 (w), 2899 (w), 2869 (w), 1723 (m), 1663 (s), 1478 (w), 1456 (w), 1413 (w), 1389 (w), 1369 (m), 1344 (w), 1288 (w), 1255 (m), 1205 (w), 1157 (m), 1120 (m), 1093 (m), 1061 (m), 996 (m), 960 (w), 847 (m), 824 (w), 759 (w)

<u>MS:</u> ($ESI^+$/QTOF)

682 (33%), 681 ($[2M+Na]^+$, 100%), 353 (10%), 352 ($[M+Na]^+$, 76%)

<u>HRMS:</u> ($ESI^+$/QTOF)

Calcd for $C_{17}H_{31}NO_5Na^+$ $[M+Na]^+$, 352.2094; found, 352.2108

<u>Data for **S4**</u>: (Peaks attributed to diastereomers are denoted by *, †, §)

<u>$^1$H NMR:</u> (500 MHz, $CDCl_3$)

6.27 (br s, 1H, N*H*), 5.88 (br s, 1H, N*H**), 5.77 (br s, 1H, N*H*†), 5.70 (br s, 1H, N*H*§), 4.47–4.39 (m, 4H, C(3′)*H*, C(3′)*H**, C(3′)*H*†, C(3′)*H*§), 3.70–3.57 (m, 20H, O*Me*, O*Me**, O*Me*†, O*Me*§, C(5′)$H_2$, C(5′)$H_2$*, C(5′)$H_2$†, C(5′)$H_2$§), 3.52–3.41 (m, 8H, C(5′)$H_2$, C(5′)$H_2$*, C(5′)$H_2$†, C(5′)$H_2$§), 2.89–2.69 (m, 8H, C(4)*H*, C(4)*H**, C(4)*H*†, C(4)*H*§, C(2″)*H*, C(2″)*H**, C(2″)*H*†, C(2″)*H*§), 2.66–2.50 (m, 8H, C(3)$H_A$, C(3)$H_A$*, C(3)$H_A$†, C(3)$H_A$§, C(3″)$H_A$, C(3″)$H_A$*, C(3″)$H_A$†, C(3″)$H_A$§), 2.48–2.25 (m, 8H, C(3)$H_B$, C(3)$H_B$*, C(3)$H_B$†, C(3)$H_B$§, C(3″)$H_B$, C(3″)$H_B$*, C(3″)$H_B$†, C(3″)$H_B$§), 2.97–1.18 (m, 4H, C(5)$H_A$, C(5)$H_A$*, C(5)$H_A$†, C(5)$H_A$§), 1.93–1.73 (m, 4H, C(1″)$H_A$, C(1″)$H_A$*, C(1″)$H_A$†, C(1″)$H_A$§), 1.71–1.34 (m, 96H, C(5)$H_B$, C(5)$H_B$*, C(5)$H_B$†, C(5)$H_B$§, C(1″)$H_B$, C(1″)$H_B$*, C(1″)$H_B$†, C(1″)$H_B$§, C(1′)$H_2$, C(1′)$H_2$*, C(1′)$H_2$†, C(1′)$H_2$§, C(2′)$H_2$, C(2′)$H_2$*, C(2′)$H_2$†, C(2′)$H_2$§, C(4)C(O)C$Me_3$, C(4)C(O)C$Me_3$*, C(4)C(O)C$Me_3$†, C(4)C(O)C$Me_3$§, C(2″)C(O)C$Me_3$, C(2″)C(O)C$Me_3$*, C(2″)C(O)C$Me_3$†, C(2″)C(O)C$Me_3$§), 1.22–1.14 (m, 24H, C(4′)*Me*, C(4′)*Me**, C(4′)*Me*†, C(4′)*Me*§, C(5′)*Me*, C(5′)*Me**, C(5′)*Me*†, C(5′)*Me*§)

<u>$^{13}$C NMR:</u> (126 MHz, $CDCl_3$)

174.3 (*C*(7)), 174.2 (*C*(7)$^*$), 173.91 (*C*(7)$^†$), 173.86 (*C*(7)$^§$), 172.8 (*C*(5″)), 172.7 (*C*(5″)$^*$), 172.71 (*C*(5″)$^†$), 172.70 (*C*(5″)$^§$), 172.1 (*C*(4″)), 171.73 (*C*(4″)$^*$), 171.67 (*C*(4″)$^†$), 171.6 (*C*(4″)$^§$), 170.8 (*C*(2)), 170.6 (*C*(2)$^*$), 170.3 (*C*(2)$^†$), 170.2 (*C*(2)$^§$), 102.73 (*C*(3′)), 102.69 (*C*(3′)$^*$, *C*(3′)$^†$), 102.68 (*C*(3′)$^§$), 82.12, 82.08 (C(7)(O)O*C*$Me_3$, C(5″)(O)O*C*$Me_3$), 81.9, 81.8 (C(7)(O)O*C*$Me_3{}^*$, C(5″)(O)O*C*$Me_3{}^*$), 81.42 (C(7)(O)O*C*$Me_3{}^†$, C(5″)(O)O*C*$Me_3{}^†$), 81.40, 81.3 (C(7)(O)O*C*$Me_3{}^§$, C(5″)(O)O*C*$Me_3{}^§$), 61.9, 61.8 (*C*(4′), *C*(5′)), 61.7, 61.66 (*C*(4′)$^*$, *C*(5′)$^*$), 61.65, 61.60 (*C*(4′)$^†$, *C*(5′)$^†$), 61.59, 61.5 (*C*(4′)$^§$, *C*(5′)$^§$), 56.7 (*C*(6)), 56.4 (*C*(6)$^*$), 56.2 (*C*(6)$^†$), 53.6 (*C*(6)$^§$), 52.1 (O*Me*), 51.97 (O*Me*$^*$), 51.95 (O*Me*$^†$, O*Me*$^§$), 42.1 (*C*(5)), 41.7 (*C*(5)$^*$), 40.9 (*C*(5)$^†$), 40.4 (*C*(5)$^§$), 38.43 ([a]), 38.39 ([a]), 38.2 ([a]), 38.1 ([a]), 37.7 ([a]), 37.6 ([a]), 36.5 ([a]), 36.1 ([a]), 36.0 ([a]), 35.9 ([a]), 35.8 ([a]), 35.7 ([a]), 35.3 ([a]), 34.5 ([a]), 33.6 ([a]), 33.52 ([a]), 33.46 ([a]), 33.4 ([a]), 33.3 ([a]), 33.2 ([a]), 32.8 ([a]), 28.4 ([b]), 28.2 ([b]), 28.13 ([b]), 28.12 ([b]), 28.11 ([b]), 28.10 ([b]), 28.073 ([b]), 28.066 ([b]), 28.03 ([b]), 27.98 ([b]), 27.9 ([b]), 27.8 ([b]), 15.4 (C(4′)*Me*, C(4′)*Me*$^*$, C(4′)*Me*$^†$, C(4′)*Me*$^§$, C(5′)*Me*, C(5′)*Me*$^*$, C(5′)*Me*$^†$, C(5′)*Me*$^§$)

[a]Any one of *C*(4), *C*(2), *C*(3), *C*(3″), *C*(1″), and {*C*(1′) or *C*(2′)} for any of the four diastereomers. It should be noted that this represents 24 carbon environments but only 21 signals are observed from δ = 39–32; this is likely due to overlapping signals.
[b]Any one of C(4)C(O)C*Me*$_3$, C(2″)C(O)C*Me*$_3$, and {*C*(1′) or *C*(2′)} for any of the four diastereomers.

<u>IR:</u> (neat)

2975 (w), 2935 (w), 2875 (w), 1725 (m), 1660 (m), 1439 (w), 1393 (w), 1368 (m), 1256 (m), 1219 (w), 1148 (s), 1059 (m), 920 (w), 846 (m), 732 (w)

<u>MS:</u> ($ESI^+$/QTOF)

1082 (25%), 1081 ($[2M+Na]^+$, 43%), 553 (23%), 552 ($[M+Na]^+$, 100%), 242 (18%)

<u>HRMS:</u> ($ESI^+$/QTOF)

Calcd for $C_{27}H_{47}NO_9Na^+$ $[M+Na]^+$, 552.3143; found: 552.3184

**F. Assessment of Scope Diversity**

***Data availability:*** *All structures (as SMILES strings), features and code used to generate the UMAP chemical spaces and determination of the Vendi scores can be found on this GitHub repository:* *https://github.com/jcadge/DeltaLactam-ChemicalSpace*

The diversity of the 26 compounds generated in the reaction scope (see Section E, above) was assessed initially by comparison of the starting amines with the Enamine primary amine library chemical space. The Enamine primary amine library of 59,438 total compounds was obtained from the Enamine website in the form of 2D structures (SD files): https://enamine.net/building-blocks/functional-classes/primary-amines, version dated 09/07/2025. Any amine salts present in the library were converted to their free amine structures using the RDKit (version 2025.3.5)[5] `SaltRemover` function. Any metal complexes were also removed by removing structures where their SMILES string contained a metal atom. Five structures in the scope were not present in the Enamine library, and these were added manually. This gave the final total of primary amine structures to 59,419. These structures are given in Figure S5 along with their Enamine catalogue numbers (those absent from the Enamine library were labelled AJC001–AJC005). It was envisaged that the diversity of the primary amines would act as a surrogate structure to assess the diversity of the final δ-lactam products.

The primary amines were featurized by two means: (1) Morgan fingerprints and (2) RDKit ADME-type 2D descriptors. Morgan fingerprints were generated for all of the structures in the filtered Enamine library using the RDKit `rdFingerprintGenerator.GetMorganGenerator` function using `radius=2`. Two fingerprint sizes were examined: `fpSize=1024` (1024-bit) and `fpSize=2048` (2048-bit). RDKit ADME descriptors were generated using the Descriptors.CalcMolDescriptors function. The list of descriptors was subjected to a variance (0.01 threshold) and collinearity (0.90 threshold) cuts to remove features which had low variance and were highly collinear, respectively. Descriptors related to counts or numbers of a given value (*i.e.*, functional group or aromatic rings) were also discarded leaving a total of 80 features. Before use in any chemical space mapping, these features were scaled using the `StandardScaler` function as implemented in scikit-learn.[6]

Due to the large dataset size, the Uniform Manifold Approximation Projection (UMAP) dimensionality reduction algorithm (version 0.5.9.post2)[7] was selected to maintain the overall global structure of the primary amine space. Four spaces were generated: (1) Morgan fingerprints (1024-bit); (2) Morgan fingerprints (2048-bit); (3) RDKit ADME descriptors and (4) Mixture of 1024-bit Morgan fingerprints and RDKit descriptors. These are shown in Figures S6–S8. For each of the spaces, the UMAP hyperparameters used were: `n_components=2`, `random_state=42`, `n_neighbors=30`, `min_dist=0.1`.

In addition to the UMAP chemical space analysis, then Vendi score of the substrate scope was also determined. This was derived from a Tanimoto similarity matrix using 1024-bit Morgan fingerprints (as described above). The `vendi-score` Python package[8] version 0.0.3 was used to determine the Vendi score and the Tanimoto similarity scores were calculated in RDKit using the `DataStructs.TanimotoSimilarity` function. The similarity matrix obtained is shown in Figure S9 which gave a Vendi score of 19 (19 unique elements in the substrate scope. The Vendi score was also calculated using 2048-bit Morgan fingerprints in the same manner which gave a value of 20 (similarity matrix given in Figure S10).

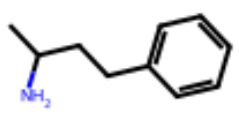
8a (EN300-20027)

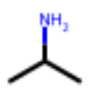
8b (EN300-18989)

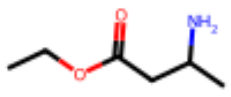
8c (AJC001)

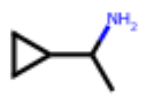
8d (EN300-13206)

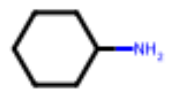
8e (EN300-16958)

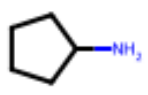
8f (EN300-20348)

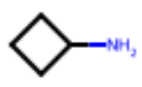
8g (EN300-62688)

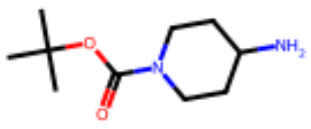
8h (AJC002)

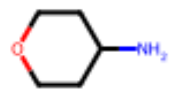
8i (EN300-43319)

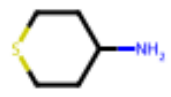
8j (EN300-61954)

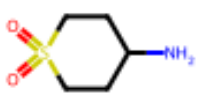
8k (EN300-39266)

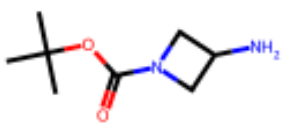
8l (AJC003)

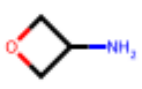
8m (EN300-49064)

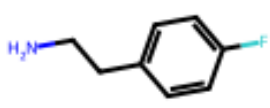
8n (EN300-18739)

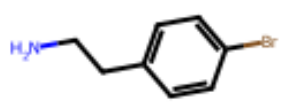
8o (EN300-77249)

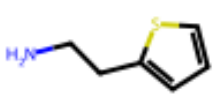
8p (EN300-12163)

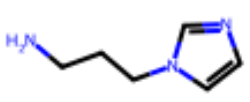
8q (EN300-19571)

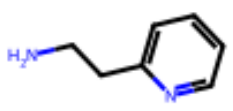
8r (EN300-20439)

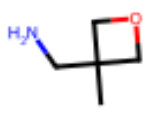
8s (EN300-75686)

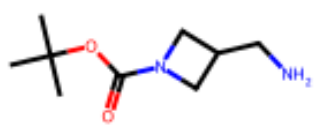
8t (AJC004)

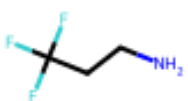
8u (EN300-41781)

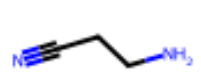
8v (EN300-21136)

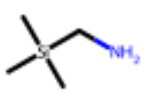
8w (EN300-7578636)

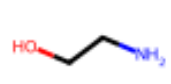
8x (EN300-19392)

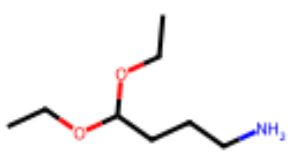
8y (EN300-20438)

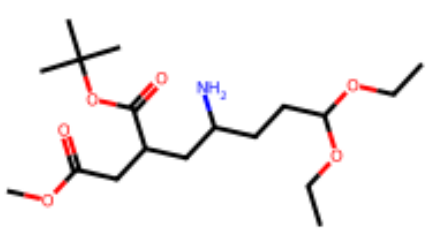
12 (AJC005)

**Figure S5.** Structures of all the primary amines used in the chemical space analysis and determination of the Vendi scores along with their Enamine catalogue numbers.

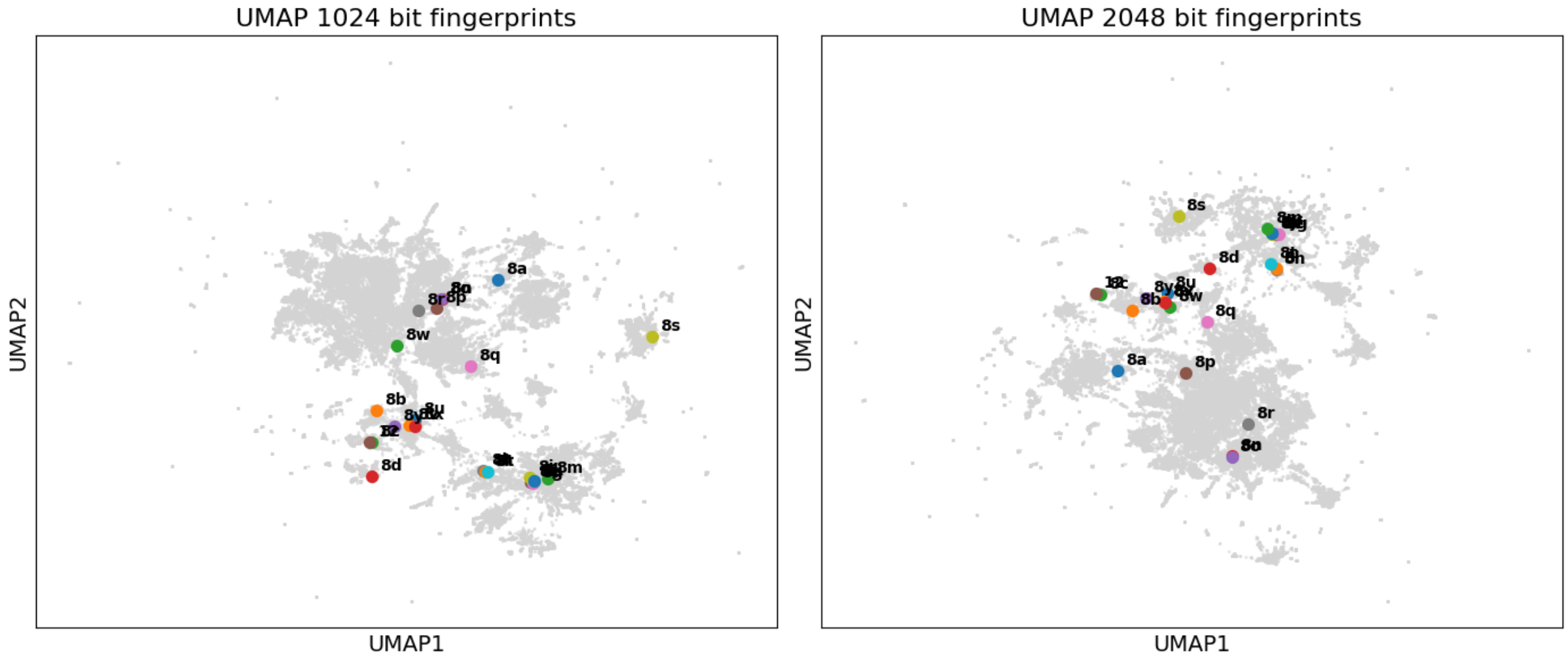


**Figure S6.** UMAP chemical spaces of the Enamine library primary amines with the substrate scope highlighted using 1024-bit (left) and 2049-bit (right) Morgan fingerprints.

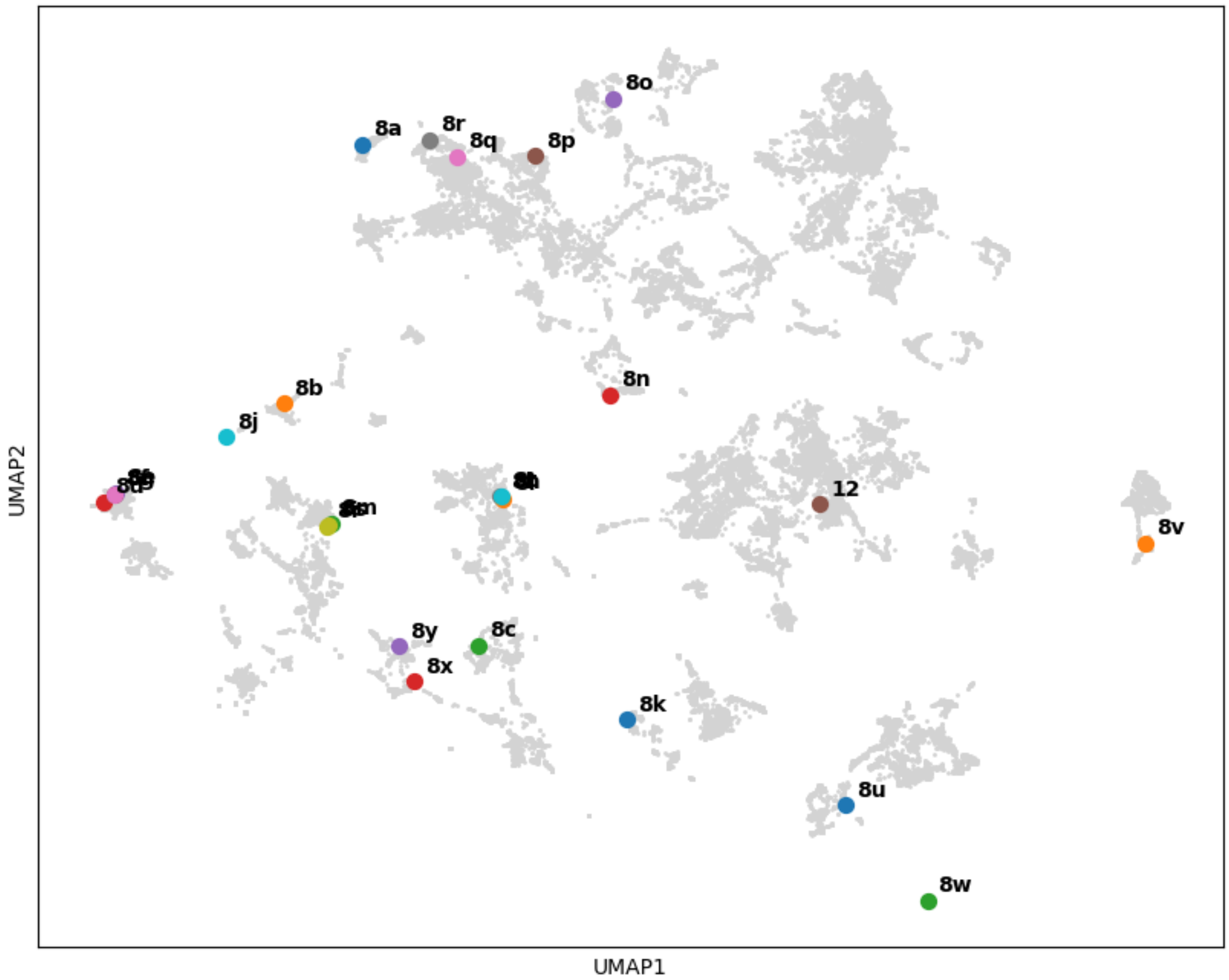


**Figure S7.** UMAP space of the Enamine primary amines with the substrate scope highlighted using RDKit ADME-type features.

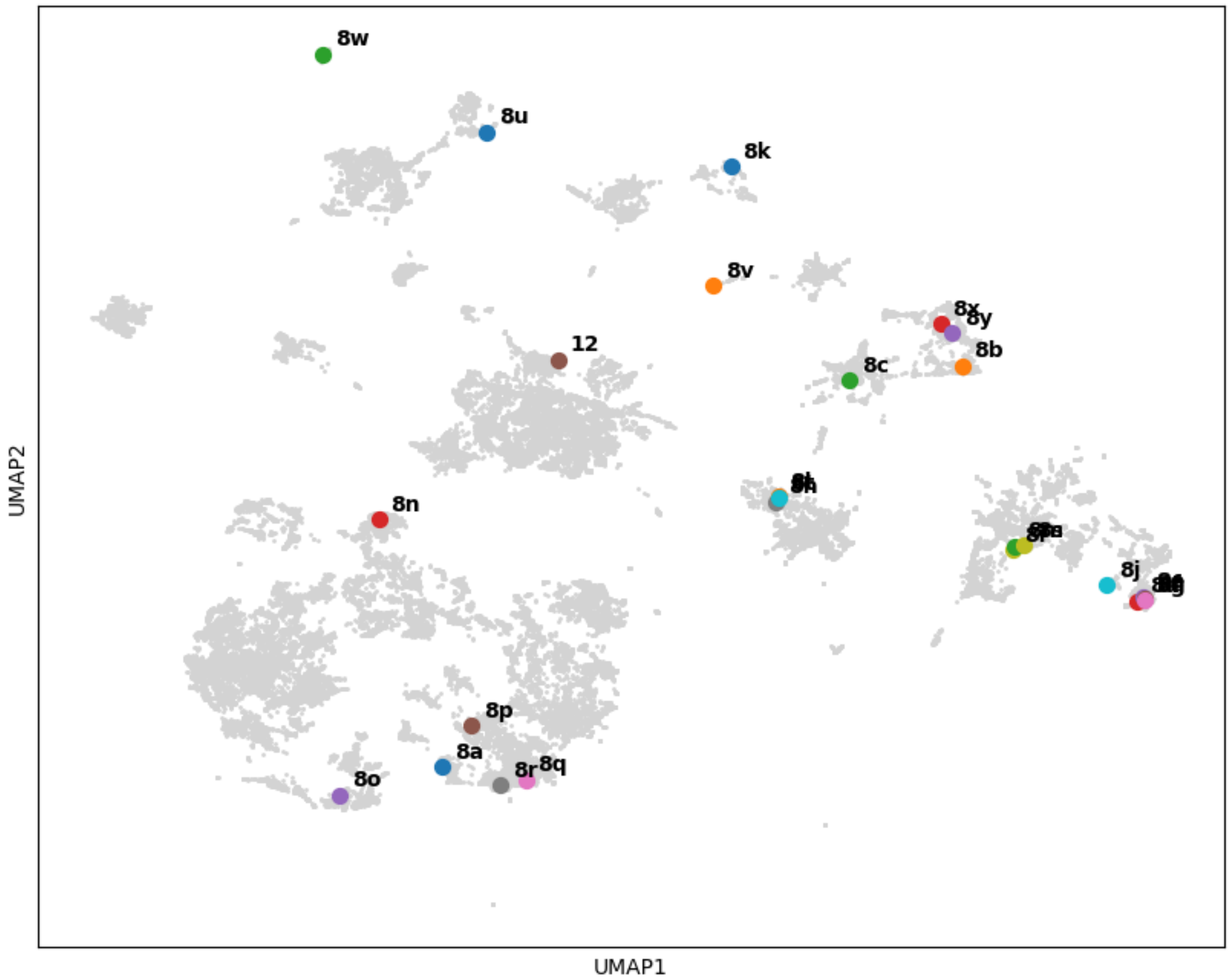


**Figure S8.** UMAP of the Enamine primary amines with the substrate scope highlighted using 1024-bit Morgan fingerprints *and* RDKit ADME-type features.

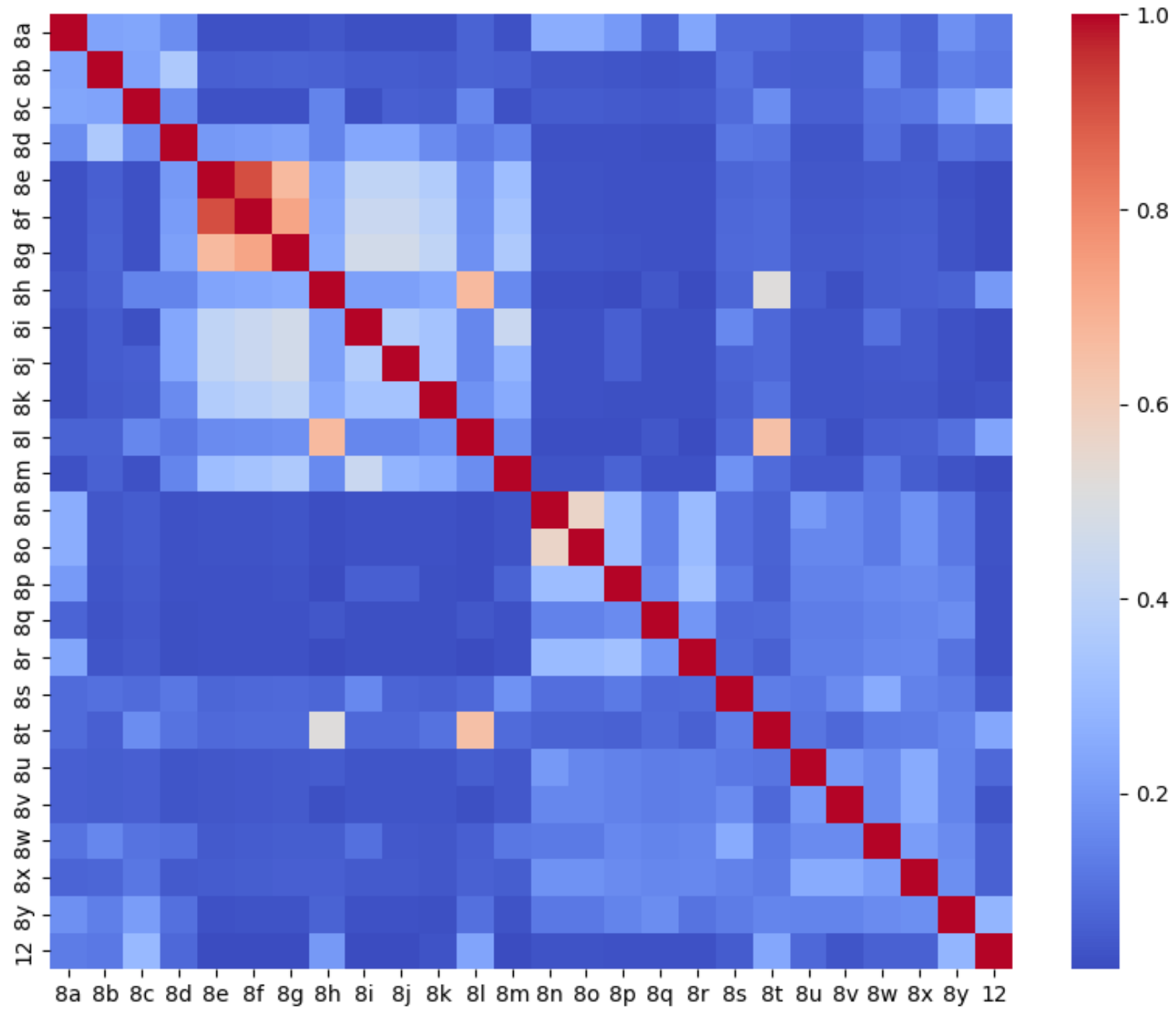


**Figure S9.** Tanimoto similarity matrix for the substrate scope using 1024-bit Morgan fingerprints.

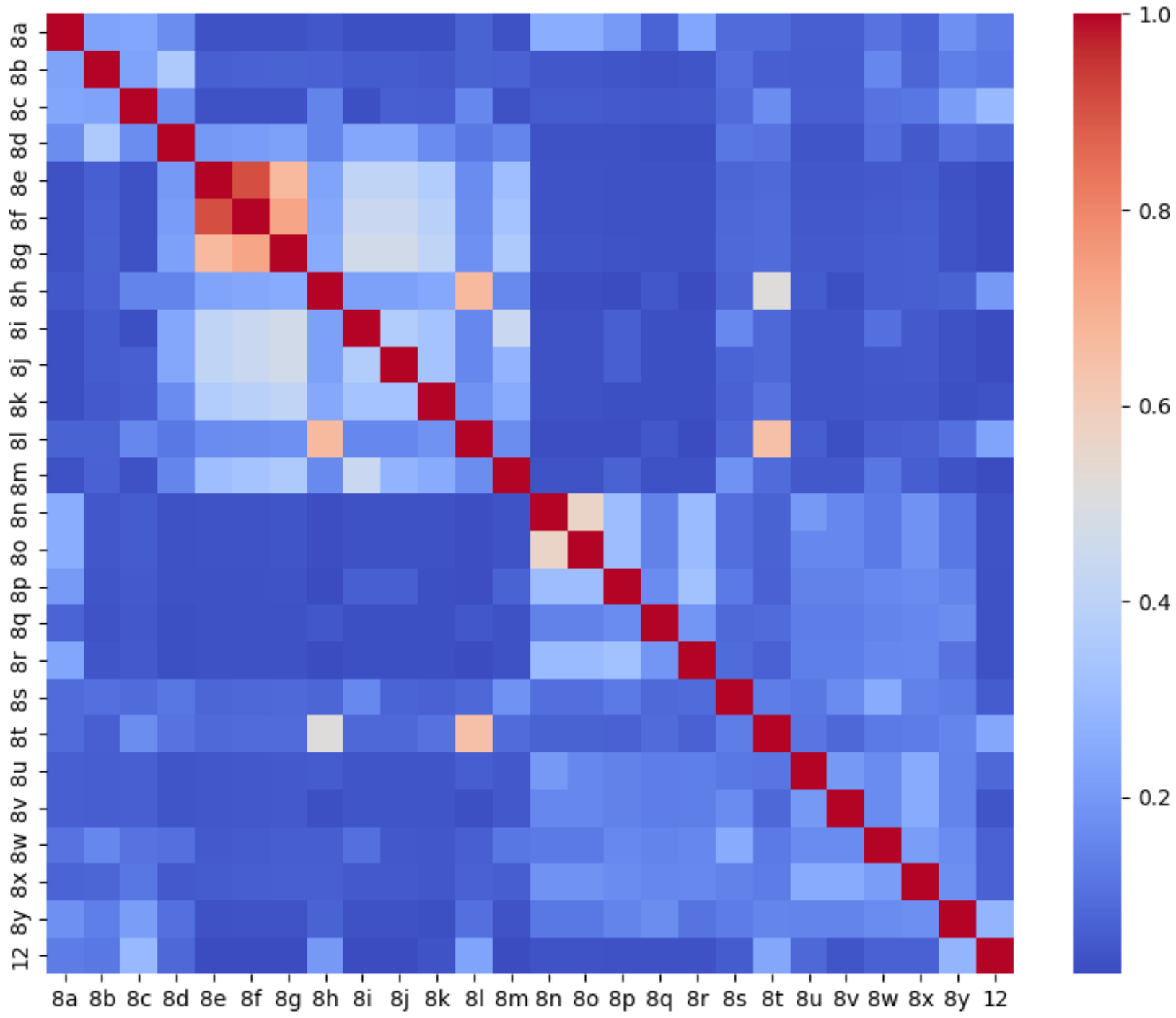


**Figure S10.** Tanimoto similarity matrix for the substrate scope using 2048-bit Morgan fingerprints.

## G. Large-Scale Synthesis

### *G.1. Optimisation for Scale Up*

Following ***General Procedure 1***, HAT catalyst (TBA•SS*i*-$Pr_3$) loading and reaction concentration were investigated.

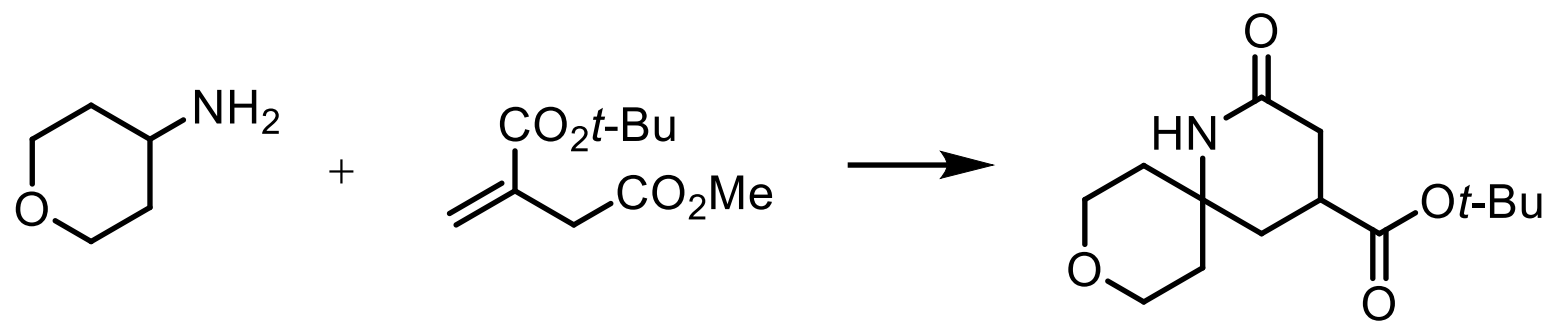

**1i** **2** **3i**

| | HAT catalyst loading | concentration | 8i yield[a] |
|---|---|---|---|
| 1 | 10 mol% | 0.15 M | 89% |
| 2 | 5 mol% | 0.15 M | 86% |
| 3 | 2 mol% | 0.15 M | 78% |
| 4 | 5 mol% | 0.25 M | 87% |
| 5 | 5 mol% | 0.40 M | 92% |
| 6 | 5 mol% | 0.50 M | 76% |

[a]Yield calculated by $^1H$ NMR, against $Bu_4N^+$ as an internal standard.

**Figure S11.**

### *G.2. Gram Scale Reaction in Continuous Flow*

A Vapourtec RS-400 flow chemistry system was configured as indicated in **Figure S12**, with an Uniqsis PhotoSyn HP LED photoreactor equipped with 420 nm LEDs. Pumps A and B were V-3 peristaltic pumps (equipped with blue pump tubes). Solvent lines for pumps A and B were fed from a reservoir of MeCN. Reagent feeds A and B were simultaneously injected into the 5.0 mL coil of the Uniqsis PhotoSyn HP LED photoreactor (49.0 mL aliquots, flow rate = 0.25 mL $min^{-1}$) to give an overall flow rate of 0.50 mL $min^{-1}$ and a $t_R$ = 10 min. Based on the prediction from Vapourtec's proprietary dispersion model, a 97.0 mL aliquot was collected from the predicted steady-state product mixture in a 250-mL, round-bottomed flask, corresponding to a theoretical maximum yield of product of 38.8 mmol. Following collection, the 49.0 mL aliquot of reaction mixture was concentrated on a rotary evaporator at 50 °C until material started to precipitate, then the flask was allowed to cool to rt. It was then left to stand in the freezer for 72 h, to allow crystallization of the product. Afterwards, the colourless, crystalline solid was collected by filtration and washed with minimal amounts of cold MeCN. The solid was ground to a powder and dried under high vacuum to give **3i** as a colourless, crystalline solid (8.33 g, 80%). The filtrate was concentrated *in vacuo* to give additional impure **3i** as a yellow solid (7.54 g). Purification of the material in three portions *via* flash column chromatography on silica gel (25 g) in [EtOAc:EtOH (3:1), $NH_4OH$ (2%)]/pentane, 0% (2 CV) then 0→30% (23 CV) then 30→100% (1 CV) then 100% (5 CV), gave additional **3i** as a pale yellow, crystalline solid (1.30 g, 94% purity, contained

6% of 4DPAIPN). Taken together, the total combined yield of **3i** (corrected to remove mass of 4DPAIPN impurity in the additional crop) was 9.58 g (92%), corresponding to a productivity of 2.97 g h$^{-1}$.

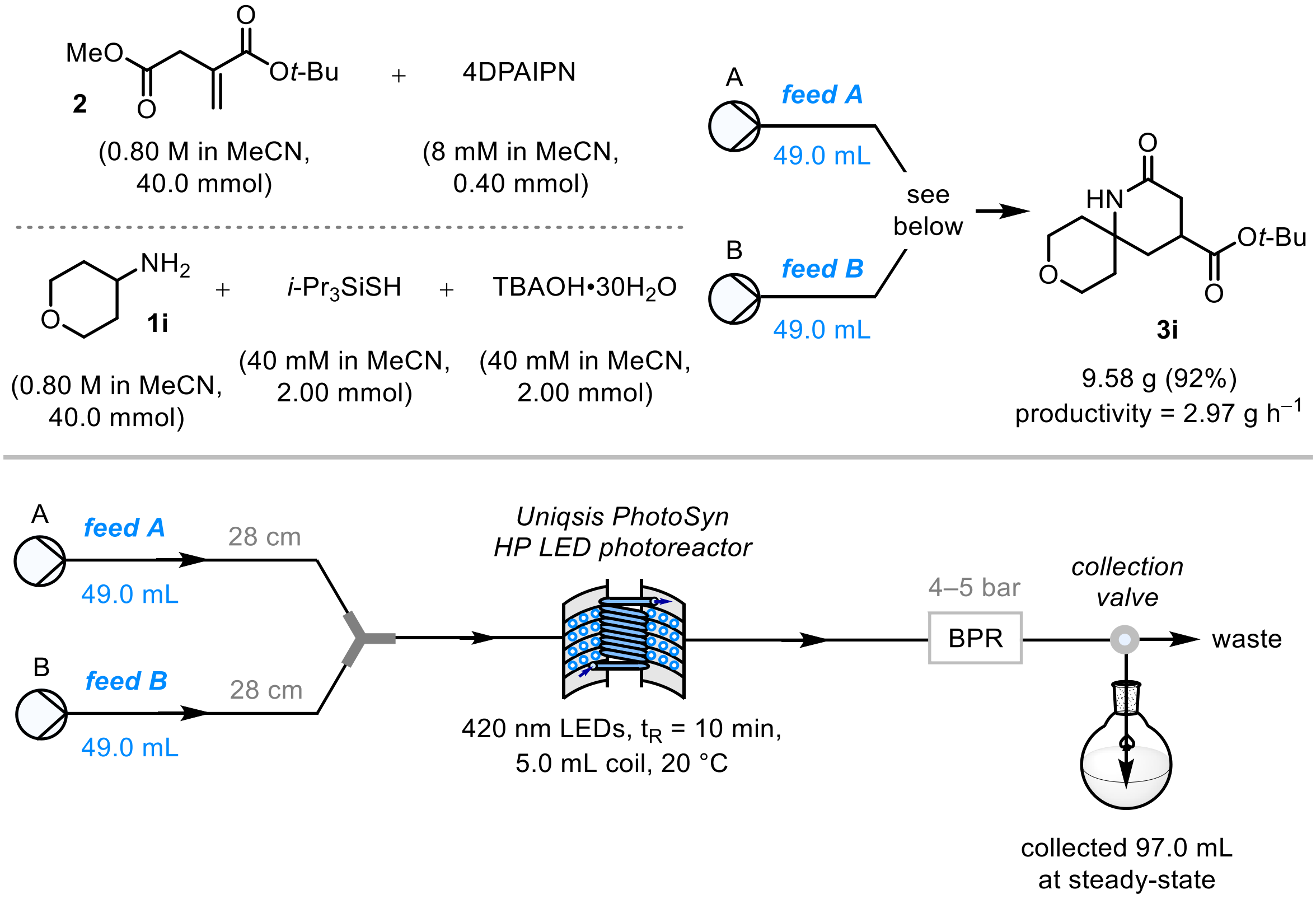


**Figure S12.**

### *G.3. Decagram Scale Reaction in Continuous Flow*

A decagram-scale synthesis of 2,2-dimethyl-6-oxopiperidine-4-carboxylic acid (**13**) and 6-oxo-5-azaspiro[3.5]nonane-8-carboxylic acid (**14**) was performed at Enamine Ltd in Kyiv, Ukraine. For convenience, 3CzClIPN (CAS# 1469704-61-7) was used in place of 4DPAIPN as the photocatalyst, due to the former catalyst being more readily available at Enamine Ltd. A customised flow photoreactor was employed that featured two 160-mL reactor coils arranged in series, each irradiated with 365 nm LEDs (SST-10-UV-B130-F365 surface-mounted LEDs from Luminus Devices Inc., 2 kW input power per coil). The tubing used was ALTAFLUOR® 200 FEP tubing (ID = 1/4 inch; OD = 5/16 inch). Each reactor coil was immediately preceded by a 50-mL pre-cooling coil, positioned directly beneath each irradiated coil (with cooling to –45 °C provided by a Huber Unistat 510 chiller).

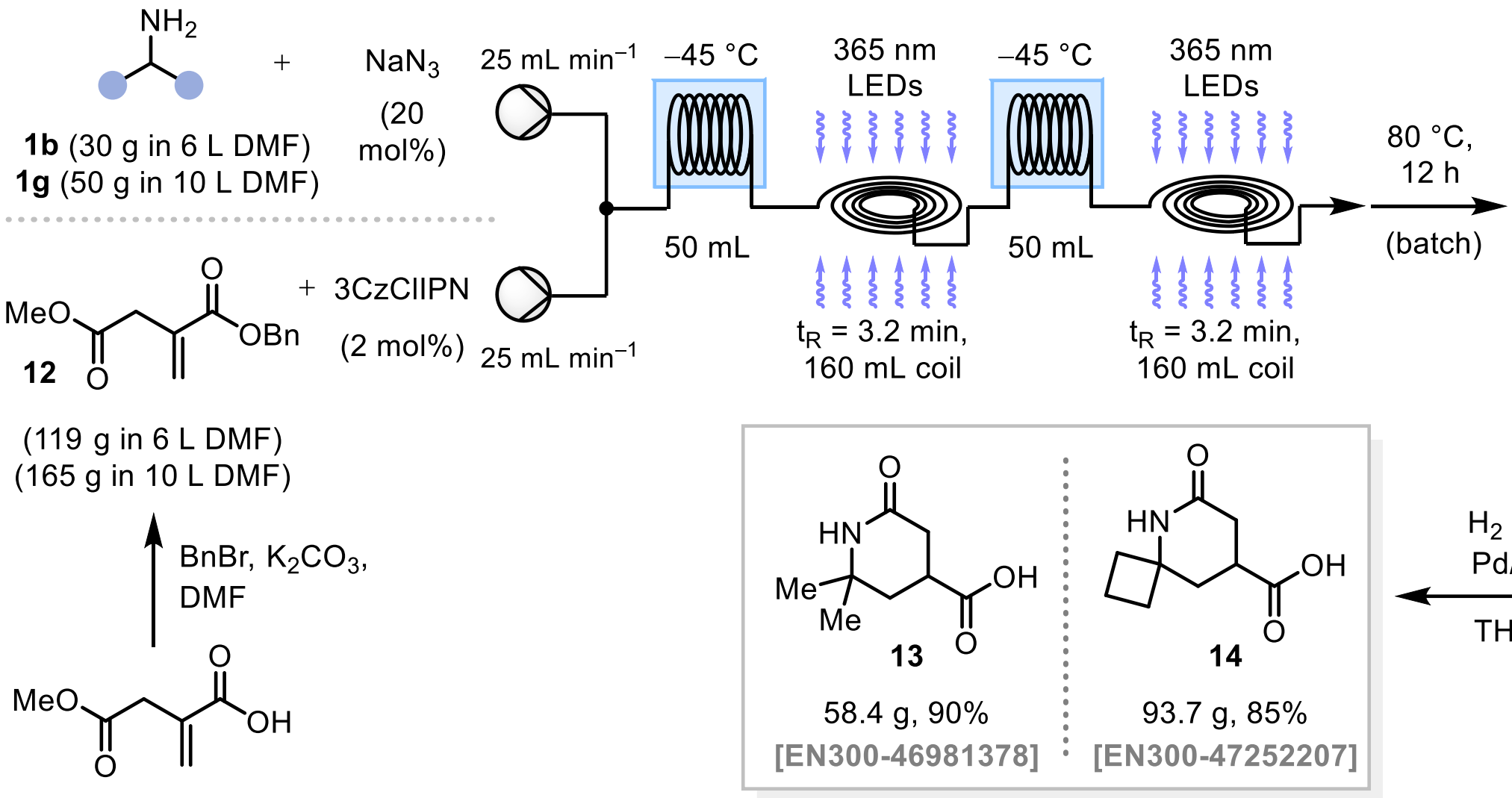


**Figure S13.** Decagram scale reaction in continuous flow.

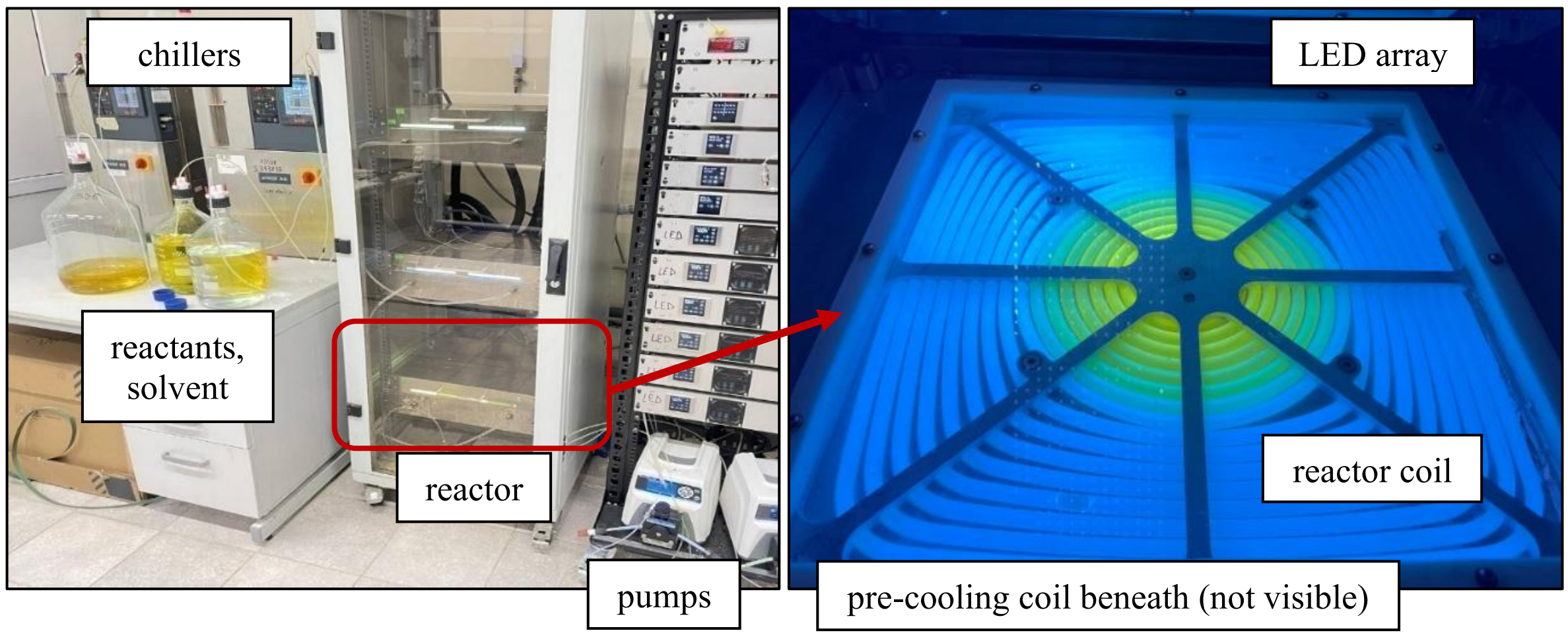


**Figure S14.** Left: Photograph of flow chemistry setup. Right: Photograph of one of the reactor coils.

### 1-Benzyl 4-methyl 2-methylenesuccinate (12)

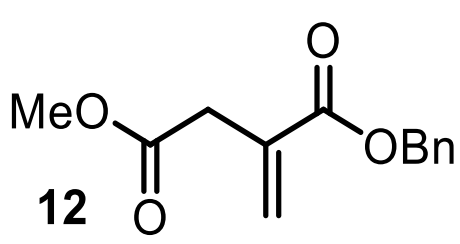


To a solution of 4-methoxy-2-methylene-4-oxobutanoic acid (90.00 g, 0.625 mol, 1.00 equiv) in DMF (600 mL) was added $K_2CO_3$ (120.8 g, 0.875 mol, 1.40 equiv) in portions at 0 °C (ice-water bath). The resultant mixture was stirred for 30 min at 0 °C (ice-water bath), and then BnBr (117.5 g, 0.687 mol, 1.10 equiv) was added dropwise. The mixture was allowed to warm to rt overnight, then was diluted with $H_2O$ (2.0 L) and extracted with MTBE (3 × 500 mL). The combined organic layers were washed sequentially with $H_2O$ (2 × 300 mL) and brine (300 mL), them dried ($Na_2SO_4$), filtered, and

concentrated *in vacuo* to give crude **12** as a colourless oil (137.5 g, 94%), which was used without further purification.

Data for **12**:

| | |
|---|---|
| $^{1}$H NMR: | (500 MHz, $CDCl_3$) |
| | 7.41–7.27 (m, 5H), 6.39 (s, 1H), 5.74 (s, 1H), 5.21 (s, 2H), 3.64 (s, 3H), 3.36 (s, 2H) |
| $^{13}$C NMR: | (126 MHz, $CDCl_3$) |
| | 171.2, 166.1, 135.9, 133.9, 129.0, 128.7, 128.4, 128.2, 66.9, 52.1, 37.7 |
| MS: | ($EI^+$) |
| | 234 $[M]^+$ |
| HRMS: | ($ESI^+$) |
| | Calcd for $C_{13}H_{18}NO_4^+$ $[M+NH_4]^+$: 252.1230 found: 252.1243 |

**Benzyl 2,2-dimethyl-6-oxopiperidine-4-carboxylate (S5)**

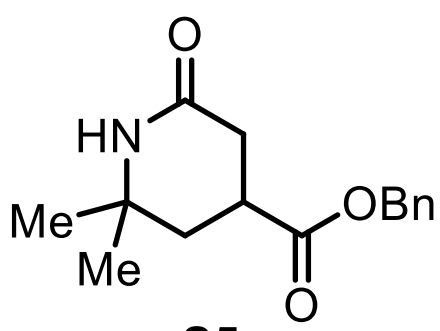

**S5**

Using the aforementioned flow set up (in **Fig S13** and **Fig S14**), a solution of **12** (118.9 g, 508 mmol, 1.00 equiv) and 3CzClIPN (6.68 g, 10.1 mmol, 2 mol%) in DMF (6.0 L) and a solution of isopropylamine **1b** (30.0 g, 508 mmol, 1.0 equiv) and $NaN_3$ (6.60 g, 102 mmol, 20 mol%) in DMF (6.0 L) were degassed with argon for 20 min. The two solutions were each pumped at 25 mL min$^{-1}$ flow rate, giving a total residence time of 6.4 min under 365 nm irradiation. Following collection of the entire 12 L volume of reaction mixture, the solution was heated at 80 °C for 12 h (to ensure complete lactamisation). The mixture was then concentrated *in vacuo*, and the residue was dissolved in EtOAc (1.0 L) and washed sequentially with $H_2O$ (3 × 300 mL) and brine (200 mL), then dried ($Na_2SO_4$), filtered, and concentrated *in vacuo*. The residue was triturated in MTBE, then filtered and dried to give **S5** as a beige solid (98.6 g, 74%).

Data for **S5**:

| | |
|---|---|
| mp | 121–122 °C |
| $^{1}$H NMR: | (500 MHz, DMSO-$d_6$) |
| | 7.59 (s, 1H), 7.42–7.27 (m, 5H), 5.12 (s, 2H), 2.36–2.29 (m, 1H), 2.22 (dd, $J$ = 17.2, 11.5 Hz, 1H), 1.93 (d, $J$ = 13.0 Hz, 1H), 1.56 (t, $J$ = 13.0 Hz, 1H), 1.17 (s, 3H), 1.16 (s, 3H) |

$^{13}$C NMR: (126 MHz, DMSO-$d_6$)

173.4, 168.0, 136.0, 128.4, 128.0, 127.8, 65.7, 51.1, 37.6, 35.0, 32.9, 30.7, 28.8

MS: (ES-API)

262 [M+H]$^+$

HRMS: (ESI$^+$)

Calcd for $C_{15}H_{19}NNaO_3^+$ [M+Na]$^+$: 284.1257 found: 284.1259

**2,2-Dimethyl-6-oxopiperidine-4-carboxylic acid (13)**

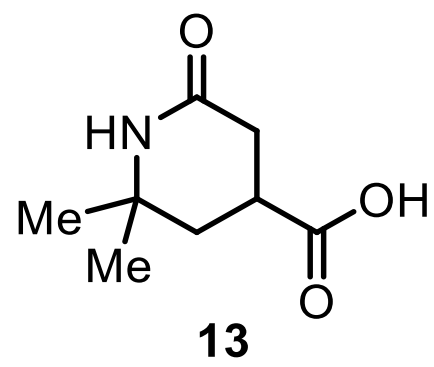


**13**

**S5** (98.6 g, 377 mmol) was dissolved in THF (1.0 L), and 10% Pd/C (10.0 g) was added, then the suspension was placed in an autoclave and hydrogenated under 40 atm of $H_2$ at 50 °C. Upon completion of the reaction, *i*-PrOH (1.0 L) was added and the heterogeneous mixture was heated to reflux. The hot suspension was then filtered, and the filtrate was set aside. *i*-PrOH (500 mL) was added to the collected solid residue and the mixture was again heated to reflux, followed by another hot filtration. The procedure was repeated until no product **13** remained in the solid residue. The combined filtrates were then concentrated *in vacuo* to give **13** as a colourless solid (58.4 g, 90%). This product is commercially available through the Enamine chemical supplier from stock under the code EN300-46981378.

Data for **13**:

mp 220–221 °C

$^1$H NMR: (500 MHz, DMSO-$d_6$)

12.48 (br s, 1H), 7.56 (s, 1H), 2.88–2.75 (m, 1H), 2.27 (dd, $J$ = 17.2, 5.0 Hz, 1H), 2.16 (dd, $J$ = 17.2, 11.6 Hz, 1H), 1.89 (d, $J$ = 13.0 Hz, 1H), 1.49 (t, $J$ = 13.0 Hz, 1H), 1.18 (s, 3H), 1.15 (s, 3H)

$^{13}$C NMR: (126 MHz, DMSO-$d_6$)

175.1, 168.5, 51.1, 38.0, 34.9, 33.1, 30.8, 28.9

MS: (ES-API)

172 [M+H]$^+$

HRMS: (ESI$^+$)

Calcd for $C_8H_{13}NNaO_3^+$ [M+Na]$^+$: 194.0788 found: 194.0794

**Benzyl 6-oxo-5-azaspiro[3.5]nonane-8-carboxylate (S6)**

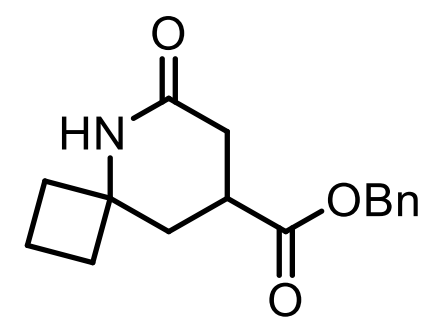

**S6**

Using the aforementioned flow set up (in **Fig S13** and **Fig S14**), a solution of **12** (164.7 g, 703 mmol, 1.00 equiv) and 3CzClIPN (9.25 g, 14.1 mmol, 2 mol%) in DMF (10 L) and a solution of cyclobutylamine **1g** (50.0 g, 703 mmol, 1.0 equiv) and $NaN_3$ (9.14 g, 141 mmol, 20 mol%) in DMF (10 L) were degassed with argon for 20 min. The two solutions were each pumped at 25 mL $min^{-1}$ flow rate, giving a total residence time of 6.4 min under 365 nm irradiation. Following collection of the entire 20 L volume of reaction mixture, the solution was heated at 80 °C for 12 h (to ensure complete lactamisation). The mixture was then concentrated *in vacuo*, and the residue was dissolved in EtOAc (1.0 L) and washed sequentially with $H_2O$ (3 × 300 mL) and brine (200 mL), then dried ($Na_2SO_4$), filtered, and concentrated *in vacuo*. The residue was triturated in MTBE, then filtered and dried to give **S6** as a beige solid (165 g, 86%).

Data for **S6**:

mp: 141–142 °C

$^{1}$H NMR: (500 MHz, DMSO-$d_6$)

8.02 (s, 1H), 8.02 (s, 1H), 7.49–7.21 (m, 5H), 5.14 (d, $J$ = 12.7 Hz, 1H), 5.11 (d, $J$ = 12.7 Hz, 1H), 2.90–2.79 (m, 1H), 2.37–2.15 (m, 4H), 2.05–1.94 (m, 2H), 1.92–1.83 (m, 1H), 1.68 (t, $J$ = 12.4 Hz, 1H), 1.64–1.49 (m, 2H)

$^{13}$C NMR: (126 MHz, DMSO-$d_6$)

173.1, 167.8, 136.0, 128.4, 128.0, 127.8, 65.7, 54.9, 35.8, 35.1, 35.0, 34.5, 33.2, 12.3

MS: (ES-API)

274 $[M+H]^+$

HRMS: (ESI$^+$)

Calcd for $C_{16}H_{19}NNaO_3^+$ $[M+Na]^+$: 296.1257 found: 296.1258

**6-Oxo-5-azaspiro[3.5]nonane-8-carboxylic acid (14)**

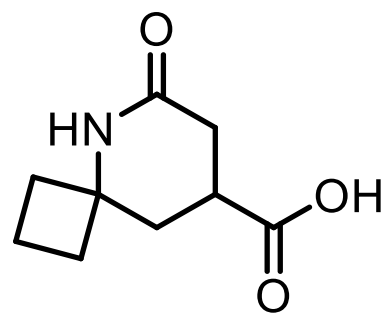

**14**

**S6** (165.20 g, 604 mmol) was dissolved in THF (2.0 L), and 10% Pd/C (16.0 g) was added, then the suspension was placed in an autoclave and hydrogenated under 40 atm of $H_2$ at 50 °C. Upon completion of the reaction, *i*-PrOH (1.0 L) was added and the heterogeneous mixture was heated to reflux. The hot suspension was then filtered, and the filtrate was set aside. *i*-PrOH (500 mL) was added to the collected solid residue and the mixture was again heated to reflux, followed by another hot filtration. The procedure was repeated until no product **14** remained in the solid residue. The combined filtrates were then concentrated *in vacuo* to give **14** as a colourless solid (93.7 g, 85%). This product is commercially available through the Enamine chemical supplier from stock under the code EN300-47252207.

Data for **14**:

mp 205–206 °C

$^1$H NMR: (500 MHz, DMSO-$d_6$)

12.48 (br s, 1H), 7.97 (s, 1H), 2.70–2.62 (m, 1H), 2.31–2.14 (m, 4H), 2.07–1.80 (m, 3H), 1.72–1.47 (m, 3H)

$^{13}$C NMR: (126 MHz, DMSO-$d_6$)

174.9, 168.3, 55.0, 35.8, 35.2, 35.0, 34.8, 33.4, 12.3

MS: (ES-API)

184 $[M+H]^+$

HRMS: ($ESI^+$)

Calcd for $C_9H_{13}NNaO_3^+$ $[M+Na]^+$: 206.0788 found: 206.0788

Crystals of the compound suitable for X-ray diffraction studies were obtained by slow evaporation of a solution of MeOH. X-Ray diffraction studies were performed on an automatic "Bruker APEX II" diffractometer (graphite monochromated MoKα radiation, CCD-detector, φ- and ω-scanning) at 100 K. The structures were solved by the direct method using OLEX2[9] package with SHELXT[10] and SHELXL modules.[11] Positions of the hydrogen atoms were located from electron density difference maps and refined by "riding" model with $U_{iso} = nU_{eq}$ of the carrier atom (n = 1.5 for methyl groups and n = 1.2 for other hydrogen atoms). Crystallographic data have been deposited at the Cambridge Crystallographic Data Centre. CCDC number: 2496300. Copies of the data are available free of charge

upon application to the CCDC, 12 Union Road, Cambridge CB21EZ, UK, (fax: +44-(0)1223-336033 or e-mail: deposit@ccdc.cam.ac.uk).

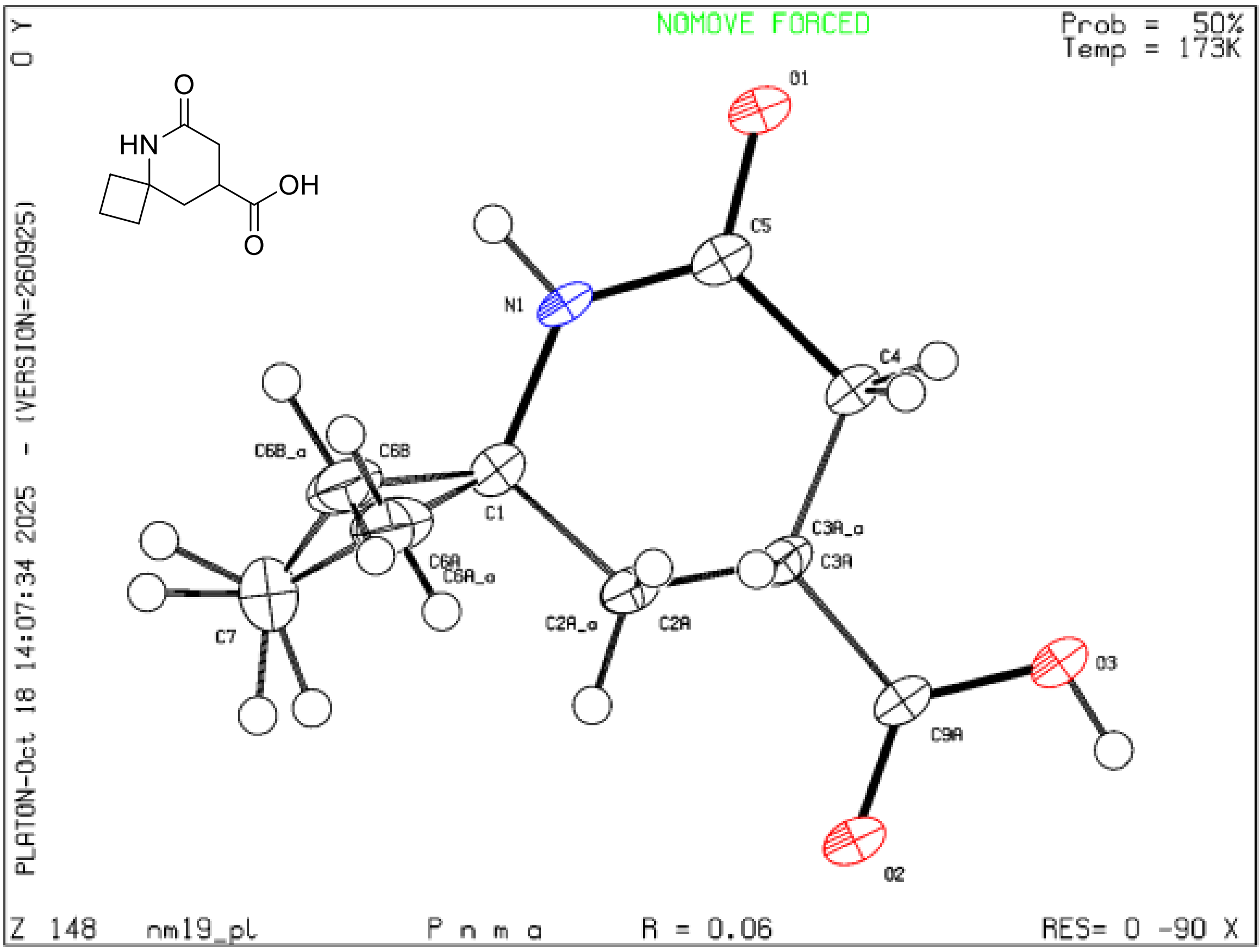


**Figure S15.** Molecular structure of 6-oxo-5-azaspiro[3.5]nonane-8-carboxylic acid (CCDC 2496300) according to X-ray diffraction data. Thermal ellipsoids are shown at a 50% probability level.

**Crystal structure determination of 6-oxo-5-azaspiro[3.5]nonane-8-carboxylic acid**

**data_nm19_pl**

_chemical_formula_moiety 'C9H17NO3'

_chemical_formula_sum 'C9H17NO3'

_chemical_formula_weight 187.23

_space_group_crystal_system 'orthorhombic'

_space_group_IT_number 62

_space_group_name_H-M_alt 'P n m a'

_space_group_name_Hall '-P 2ac 2n'

_cell_length_a 17.3679(12)

_cell_length_b 6.6550(5)

_cell_length_c 7.7222(6)

_cell_angle_alpha 90

_cell_angle_beta 90
_cell_angle_gamma 90
_cell_volume 892.56(11)
_cell_formula_units_Z 4
_cell_measurement_reflns_used 3261
_cell_measurement_temperature 173(2)
_cell_measurement_theta_max 26.40
_cell_measurement_theta_min 2.35
_shelx_estimated_absorpt_T_max 0.989
_shelx_estimated_absorpt_T_min 0.977
_exptl_absorpt_coefficient_mu 0.104
_exptl_absorpt_correction_T_max 0.7454
_exptl_absorpt_correction_T_min 0.5407
_exptl_absorpt_correction_type multi-scan
_exptl_absorpt_process_details SADABS-2014/5
_exptl_crystal_colour colourless
_exptl_crystal_colour_primary colourless
_exptl_crystal_density_diffrn 1.393
_exptl_crystal_description block
_exptl_crystal_F_000 408
_exptl_crystal_size_max 0.23
_exptl_crystal_size_mid 0.14
_exptl_crystal_size_min 0.11
_diffrn_reflns_av_R_equivalents 0.0653
_diffrn_reflns_av_unetI/netI 0.0301
_diffrn_reflns_Laue_measured_fraction_full 1.000
_diffrn_reflns_Laue_measured_fraction_max 1.000
_diffrn_reflns_limit_h_max 21
_diffrn_reflns_limit_h_min -21
_diffrn_reflns_limit_k_max 8
_diffrn_reflns_limit_k_min -8
_diffrn_reflns_limit_l_max 9
_diffrn_reflns_limit_l_min -9
_diffrn_reflns_number 12462
_diffrn_reflns_point_group_measured_fraction_full 1.000
_diffrn_reflns_point_group_measured_fraction_max 1.000
_diffrn_reflns_theta_full 25.242

```
_diffrn_reflns_theta_max          26.445
_diffrn_reflns_theta_min          2.345
_diffrn_ambient_temperature       173(2)
_diffrn_measured_fraction_theta_full  1.000
_diffrn_measured_fraction_theta_max  1.000
_diffrn_measurement_device_type   'Bruker APEX-II CCD'
_diffrn_measurement_method        '\f and \w scans'
_diffrn_radiation_type            MoK\a
_diffrn_radiation_wavelength      0.71073
_diffrn_source_current            30.0
_diffrn_source_power              1.2
_diffrn_source_voltage            40.0
_diffrn_standards_number          0
_reflns_Friedel_coverage          0.000
```

**H. Product Derivatisations**

***tert*-Butyl (*RS*)-9-oxa-1-azaspiro[5.5]undecane-4-carboxylate (7)**

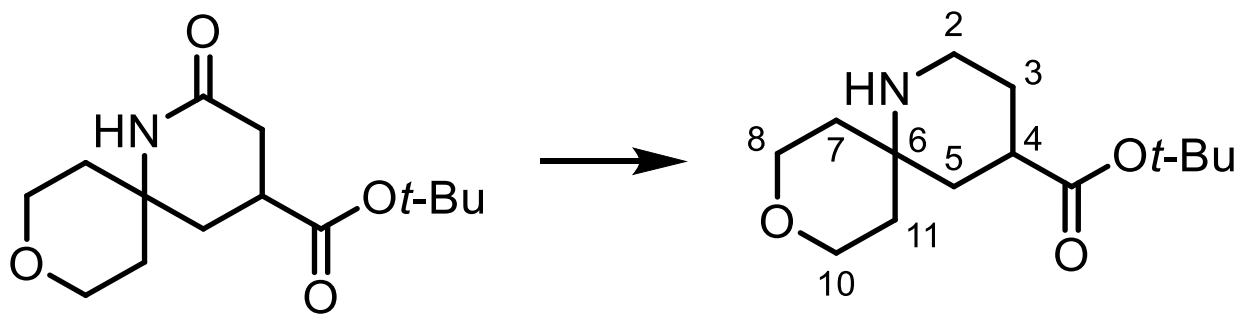


**3i** **7**

In an $N_2$-filled purge box, a J-Young NMR tube was charged with **3i** (27 mg, 0.10 mmol, 1.0 equiv), $NiCl_2$•dme (2 mg, 0.01 mmol, 10 mol%) and PhMe (100 µL). $PhSiH_3$ (24 µL, 0.20 mmol, 2.0 equiv) was then added, and the sealed NMR tube was heated at 115 °C for 48 h. The reaction mixture was allowed to cool to rt followed by addition of 1 M aq. NaOH (2 mL). The mixture was extracted with EtOAc (5 × 2 mL) and the combined organic extracts were then dried ($MgSO_4$), filtered, and concentrated *in vacuo*. Purification *via* flash column chromatography on silica gel (12 g) in pentane/[EtOAc:EtOH (3:1), $NH_4OH$ (2%)], 20→50% (14 CV) then 50% (6 CV) then 80% (4 CV), gave **7** as a colourless solid (13 mg, 51%).

Data for **7**:

$^1$H NMR: (400 MHz, $CDCl_3$)

3.84–3.52 (m, 4H, C(8)$H_2$, C(10)$H_2$), 2.97–2.86 (m, 1H, C(2)$H_A$), 2.82–2.71 (m, 1H, C(2)$H_B$), 2.47 (app tt, $J$ = 12.3, 3.8 Hz, 1H, C(4)$H$), 1.93–1.55 (m, 5H) and 1.49–1.19 (m, 12H) (C(3)$H_2$, C(5)$H_2$, C(7)$H_2$, C(11)$H_2$, $CO_2CMe_3$)

$^{13}$C NMR: (101 MHz, $CDCl_3$)

174.9 ($CO_2CMe_3$), 80.4 ($CO_2CMe_3$), 63.8, 63.5 ($C$(8), $C$(10)), 48.9 ($C$(6)), 41.2 ($C$(7) or $C$(11)), 39.8 ($C$(2)), 39.2 ($C$(5)), 38.4 ($C$(4)), 32.2 ($C$(11) or $C$(7)), 29.9 ($C$(3)), 28.2 ($CO_2CMe_3$)

IR: (neat)

2931 (m), 2859 (w), 1720 (s), 1448 (m), 1365 (w), 1303 (w), 1238 (w), 1148 (s), 1101 (m), 1015 (w)

HRMS: (ESI$^+$/QTOF)

Calcd for $C_{14}H_{26}NO_3^+$ $[M+H]^+$, 256.1907; found, 256.1908

**(*RS*)-2-Oxo-9-oxa-1-azaspiro[5.5]undecane-4-carboxylic acid (8)**

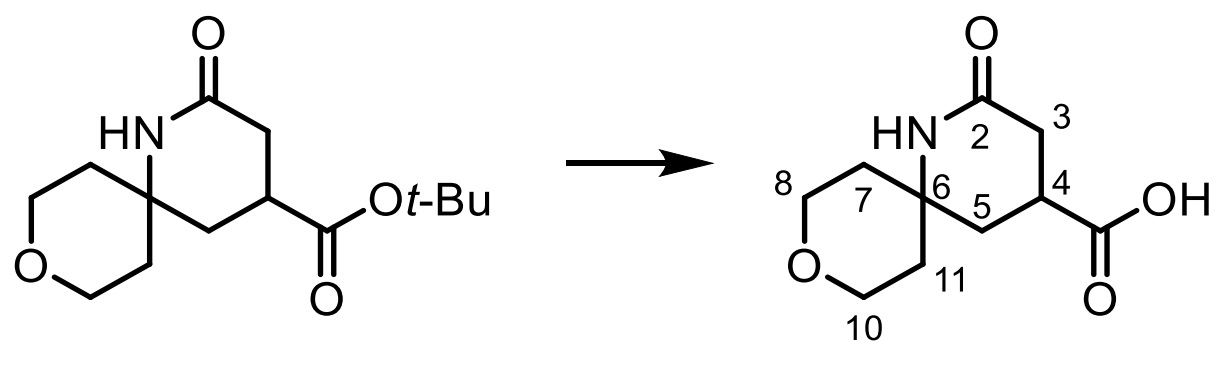


**3i** **8**

A nitrogen-flushed, 100-mL, round-bottomed flask equipped with a stirrer bar was charged with **3i** (5.39 g, 20.0 mmol, 1.0 equiv) and $CH_2Cl_2$ (100 mL). The solution was cooled in an ice-water bath under a stream of $N_2$ and $CF_3CO_2H$ (15.4 mL, 22.8 g, 200 mmol, 10 equiv) was added *via* syringe pump (1 mL $min^{-1}$). After stirring the mixture for an additional 5 min, the ice bath was removed and replaced with an oil bath. A nitrogen-flushed, water-jacketed reflux condenser was fitted and the mixture was stirred at 40 °C for 2 h, during which time it changed colour from pale orange (due to minor photocatalyst contaminants) to dark purple. The resultant mixture was concentrated on a rotary evaporator to afford a purple-black oil (4.16 g) [CAUTION: TFA is highly corrosive; upon completion, the rotary evaporator was rinsed extensively with sat. aq. $Na_2CO_3$ and a mixture of $H_2O$ and acetone was evaporated through the diaphragm pump to purge it of any residual TFA]. The purple residue was triturated in 3:1 MeOH: $CH_2Cl_2$ (100 mL) and filtered through a sintered funnel. The resultant solid was ground to a powder and dried under high vacuum to give **8** an off-white solid (3.24 g, 76%). The filtrate was concentrated *in vacuo* to give a purple solid (940 mg), which was dissolved in hot AcOH (50 mL, 50 °C). To the hot solution was added activated charcoal (50 mg) and the mixture was stirred at 50 °C for 30 min, then hot-filtered through a Celite® pad. The hot charcoal filtration was repeated twice, then the resultant solution was concentrated *in vacuo* to give additional **8** as a pale purple solid (779 mg, 18%). Overall, the combined yield of **8** was 4.02 g (94%).

Data for **8**:

mp: 223–225 °C

$^{1}$H NMR: (500 MHz, AcOH-$d_4$)

3.99–3.91 (m, 1H) and 3.85–3.75 (m, 3H) (C(8)$H_2$, C(10)$H_2$), 3.13–3.03 (m, 1H, C(4)$H$), 2.76 (ddd, $J$ = 18.2, 5.6, 1.6 Hz, 1H, C(3)$H_A$), 2.62 (dd, $J$ = 18.2, 11.5 Hz, 1H, C(3)$H_B$), 2.41–2.33 (m, 1H, C(5)$H_A$), 1.87–1.78 (m, 3H) and 1.78–1.68 (m, 2H) (C(5)$H_B$, C(7)$H_2$, C(11)$H_2$)

$^{13}$C NMR: (126 MHz, AcOH-$d_4$)

179.8 ($C$O$_2$H), 175.7 ($C$(2)), 64.7, 64.2 ($C$(8), $C$(10)), 53.5 ($C$(6)), 40.2, 38.5 ($C$(7), $C$(11)), 36.5 ($C$(5)), 35.3 ($C$(4)), 33.5 ($C$(3))

IR: (neat)

3263 (w), 2963 (w), 2933 (w), 2866 (w), 2436 (w), 2033 (w), 1932 (w), 1690 (m), 1604 (m), 1449 (w), 1406 (m), 1372 (w), 1359 (w), 1320 (m), 1297 (m), 1255 (w), 1244 (m), 1210 (m), 1200 (m), 1188 (m), 1141 (m), 1106 (m), 1094 (m), 1048 (w), 1010 (m), 970 (m), 935 (w), 913 (w), 876 (w), 843 (m), 810 (w), 784 (w), 762 (w), 741 (w), 687 (m)

MS: ($ESI^{-}$/QTOF)

213 (11%), 212 ($[M–H]^{-}$, 100%)

HRMS: ($ESI^{-}$/QTOF)

Calcd for $C_{10}H_{14}NO_4^{-}$ $[M–H]^{-}$: 212.0928, found: 212.0930

**9-Oxa-1-azaspiro[5.5]undecan-2-one (9)**

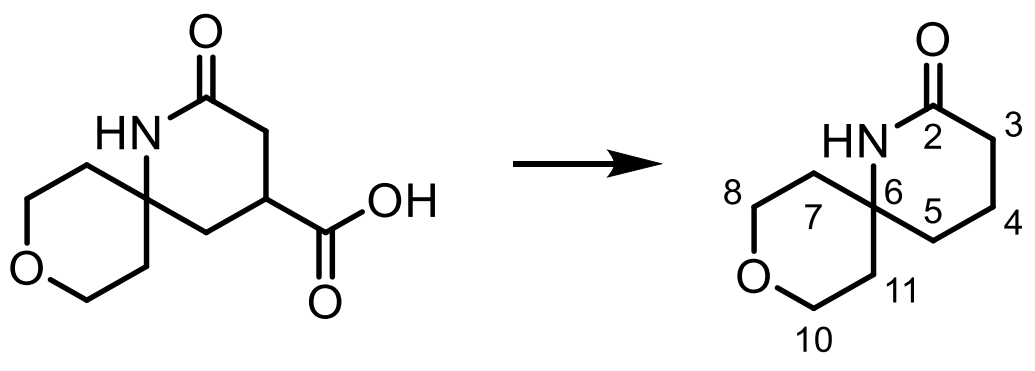


An oven-dried, 5 -mL microwave vial equipped with a stirrer bar was charged with **8** (43 mg, 0.2 mmol, 1.0 equiv), $(NH_4)_2S_2O_8$ (137 mg, 0.6 mmol, 3.0 equiv) and 2,4,6-collidine (73 mg, 79 µL, 0.6 mmol, 3.0 equiv) and dry DMSO (0.4 mL). The vial was crimp-sealed, sparged with argon for 10 min and then placed in a heating block at 60 °C and stirred for 2 h. The reaction mixture was cooled to rt and partitioned between $CH_2Cl_2$ (20 mL) and brine (70 mL). The layers were separated and the aqueous phase was re-extracted with $CH_2Cl_2$ (3 × 20 mL). The organic extracts were combined, dried ($Na_2SO_4$), filtered, and concentrated *in vacuo*. Purification *via* flash column chromatography on silica gel (12 g) in $CH_2Cl_2$/[MeOH, $NH_4OH$ (9:1)], 0% (5 CV), 0 → 10% (15 CV), 10% (5 CV), gave **9** as a colourless, amorphous solid (33 mg, 97%).

Data for **9**:

$^{1}H$ NMR: (500 MHz, $CDCl_3$)

6.44 (br s, 1H, N*H*), 3.76–3.63 (m, 4H, C(8)$H_2$, C(10)$H_2$), 2.34 (t, *J* = 6.6 Hz, 2H, C(3)$H_2$), 1.84–1.61 (m, 8H, C(4)$H_2$, C(5)$H_2$, C(7)$H_2$, C(11)$H_2$)

$^{13}$C NMR: (126 MHz, $CDCl_3$)

172.1 (*C*(2)), 63.7 (*C*(8), *C*(10)), 51.9 (*C*(6)), 39.1 (*C*(7), *C*(11)), 32.8 (*C*(5)), 31.5 (*C*(3)), 16.8 (*C*(4))

IR: (neat)

3421 (w), 3275 (w), 3216 (w), 3069 (w), 2943 (w), 2913 (w), 2880 (w), 2848 (w), 1639 (s), 1483 (w), 1467 (w), 1450 (w), 1430 (w), 1404 (m), 1387 (w), 1356 (w), 1341 (w), 1333 (w), 1292 (w), 1247 (w), 1219 (m), 1185 (w), 1174 (m), 1138 (w), 1121 (w), 1104 (m), 1095 (m), 1065 (w), 1051 (w), 1034 (w), 1017 (m), 977 (w), 948 (w), 913 (w), 847 (m), 821 (m), 785 (m), 724 (w), 659 (w)

MS: ($ESI^+$/QTOF)

193 (11%), 192 ($[M+Na]^+$, 90%), 171 (11%), 170 ($[M+H]^+$, 100%)

HRMS: ($ESI^+$/QTOF)

Calcd for $C_9H_{16}NO_2^+$ $[M+H]^+$, 170.1176; found, 170.1173

**_tert_-Butyl (_RS_)-(2-oxo-9-oxa-1-azaspiro[5.5]undecan-4-yl)carbamate (10)**

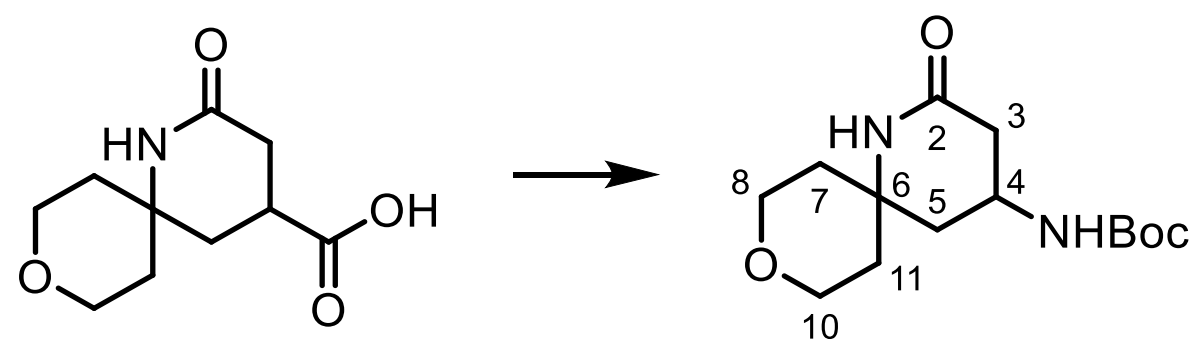


**8** **10**

To a stirred solution of **14** (21 mg, 0.10 mmol, 1.0 equiv) and $Et_3N$ (16 µL, 12 mg, 0.12 mmol, 1.2 equiv) in *t*-BuOH (300 µL) was added diphenylphosphoryl azide (30 µL, 39 mg, 0.14 mmol, 1.4 equiv) followed by heating at reflux for 5 h. The reaction mixture was then allowed to cool to rt, quenched by addition of 1 M aq. citric acid (1.0 mL), and extracted with $CHCl_3$ (3 × 2 mL). The combined organic extracts were dried ($MgSO_4$), filtered, and concentrated *in vacuo*. Purification *via* flash column chromatography on silica gel (12 g) in pentane/[EtOAc:EtOH (3:1), $NH_4OH$ (2%)], 20→50% (10 CV) then 50% (6 CV), gave **10** as a colourless oil (20 mg, 71%).

Data for **10**:

$^{1}$H NMR: (400 MHz, $CDCl_3$)

6.71 (br s, 1H, N(1)*H*), 4.53 (br s, 1H, C(4)N*H*), 4.01 (br s, 1H, C(4)*H*), 3.84–3.62 (m, 4H, C(8)$H_2$, C(10)$H_2$), 2.85–2.68 (m, 1H, C(3)$H_A$), 2.41–2.26 (m, 1H, C(5)$H_A$), 2.12

(dd, $J$ = 17.2, 11.3 Hz, 1H, C(3)$H_B$), 1.91–1.56 (m, 4H, C(7)$H_2$, C(11)$H_2$), 1.52–1.32 (m, 10H, $CO_2CMe_3$, C(5)$H_B$)

<u>$^{13}$C NMR:</u> (101 MHz, $CDCl_3$)

170.1 (*C*(2)), 155.0 ($NCO_2C(CH_3)_3$), 80.1 ($CO_2CMe_3$), 64.0, 63.3 (*C*(8), *C*(10), 51.3 (*C*(6)), 42.1 (*C*(4)), 40.2, 39.7, 38.4 (*C*(3), *C*(5), *C*(7), *C*(11)), 28.5 ($CO_2CMe_3$)

<u>IR:</u> (neat)

3270 (w), 2961 (w), 2931 (w), 1692 (m), 1680 (s), 1525 (w), 1389 (w), 1365 (w), 1295 (w), 1249 (w), 1167 (w), 1103 (m), 730 (m)

<u>HRMS:</u> ($ESI^+$/QTOF)

Calcd for $C_{14}H_{24}N_2O_4Na^+$ $[M+Na]^+$, 307.1628; found, 307.1621

**(*RS*)-4-(1′-(Trifluoromethyl)phenyl)-2-oxo-9-oxa-1-azaspiro[5.5]undecane (11)**

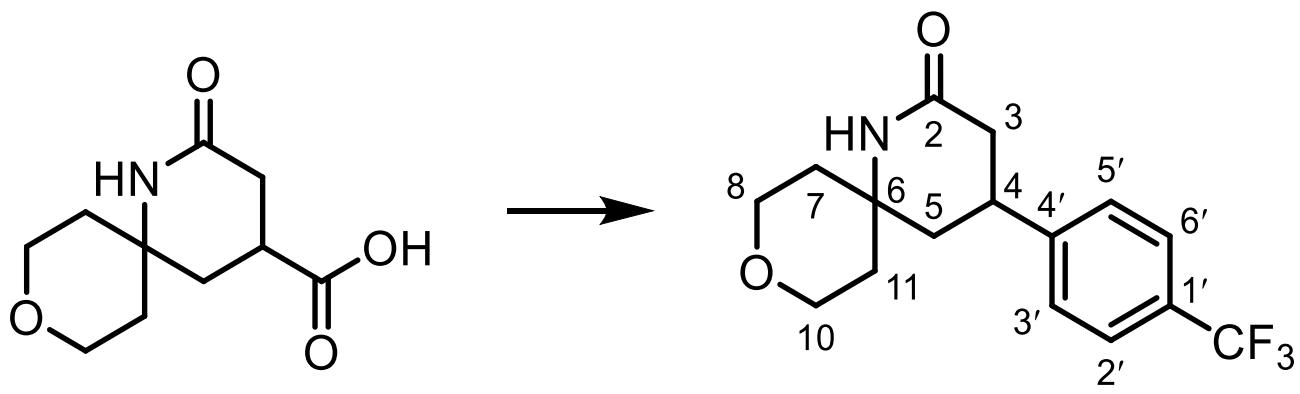


**8** **11**

A 20-mL vial equipped with a stirrer bar was charged with **8** (64 mg, 0.30 mmol, 1.5 equiv), 1-bromo-4-(trifluoromethyl)benzene (28 µL, 59 mg, 0.20 mmol, 1.0 equiv), $Ir[dF(CF_3)ppy]_2(dtbbpy)PF_6$ (2 mg, 2.0 µmol, 1 mol%), $NiCl_2$(glyme) (2 mg, 10.0 µmol, 5 mol%), 4,4′-di-*tert*-butyl-4,4′-pyridine (3 mg, 10 µmol, 5.3 mol%), phthalimide (29 mg, 0.2 mmol, 1.0 equiv), and DMSO (2 mL). BTMG (56 µL, 0.30 mmol, 1.5 equiv) was then added and the mixture was sparged with $N_2$ gas for 15 min. The mixture was stirred under irradiation (425 nm LEDs) for 24 h and then concentrated *in vacuo*. Purification *via* flash column chromatography on silica gel (12 g) in pentane/[EtOAc:EtOH (3:1), $NH_4OH$ (2%)], 5% (5 CV) then 5→15% (20 CV), gave **11** as a colourless solid (50 mg, 79%).

<u>Data for **11**:</u>

<u>mp:</u> 235–240 °C

<u>$^1$H NMR:</u> (400 MHz, $CDCl_3$)

7.62 (d, $J$ = 8.1 Hz, 2H, C(2′)*H*, C(6′)*H*), 7.34 (d, $J$ = 8.0 Hz, 2H, C(3′)*H*, C(5′)*H*), 6.47 (br s, 1H, N*H*), 3.85–3.66 (m, 4H, C(8)$H_2$, C(10)$H_2$), 3.36–3.18 (m, 1H, C(4)*H*), 2.77–

2.63 (m, 1H, C(3)$H_A$), 2.45 (dd, $J$ = 17.5, 12.4 Hz, 1H, C(3)$H_B$), 2.28–2.17 (m, 1H, C(5)$H_A$), 1.90–1.65 (m, 5H, C(5)$H_B$, C(7)$H_2$, C(11)$H_2$)

$^{13}$C NMR: (101 MHz, CDCl$_3$)

170.9 ($C$(2)), 147.2 ($C$(4′)), 129.6 (d, $J$ = 32.5 Hz, $C$(1′)) 127.2 ($C$(2′), $C$(6′)), 126.05 (q, $J$ = 3.8 Hz, $C$(3′), $C$(5′)), 124.2 (d, $J$ = 272 Hz, $C$F$_3$), 64.0, 63.4 ($C$(8), $C$(10)), 52.0 ($C$(6)), 40.6, 40.6, 39.0, 38.3 ($C$(3), $C$(5), $C$(7), $C$(11)), 34.6 ($C$(4))

IR: (neat)

3204 (w), 2921 (w), 2852 (w), 1649 (s), 1326 (m), 1174 (w), 1124 (w), 1105 (w), 1068 (w)

HRMS: (ESI$^+$/QTOF)

Calcd for $C_{16}H_{19}F_3NO_2^+$ [M+H]$^+$, 314.1368; found, 314.1365

**(*RS*)-*tert*-Butyl (2,2-dimethyl-6-oxopiperidin-4-yl)carbamate (S7)**

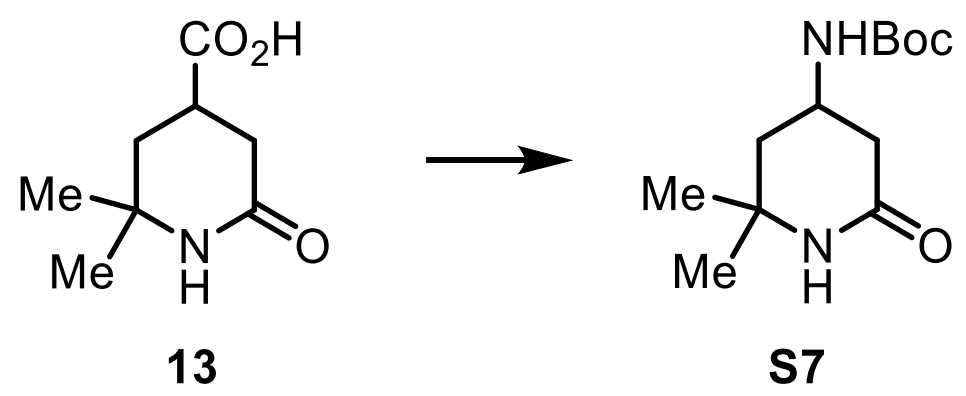

A mixture of 2,2-dimethyl-6-oxopiperidine-4-carboxylic acid **13** (1.00 g, 0.0058 mol, 1.00 equiv), DPPA (1.76 g, 0.0064 mol, 1.10 equiv), Et$_3$N (0.65 g, 0.0064 mol, 1.10 equiv) in *t*-BuOH (20 mL) was heated under reflux (in an oil bath with a thermocouple) for 10 h. The reaction mixture was cooled to rt and concentrated *in vacuo*. The residue was dissolved in MTBE (20 mL) and washed with a 20% aq. NaOH solution (2 × 10 mL), brine (10 mL), dried (Na$_2$SO$_4$), filtered, and concentrated *in vacuo* to give **S7** as a colourless solid (1.26 g, 90%), which was used for the next step without purification.

Data for **S7**:

$^1$H NMR: (500 MHz, CDCl$_3$)

5.77 (s, 1H), 4.43 (s, 1H), 4.05 (br s, 1H), 2.75 (dd, $J$ = 17.0, 4.8 Hz, 1H), 2.15 – 1.97 (m, 2H), 1.64 (s, 1H), 1.45 (s, 9H), 1.31 (s, 3H), 1.28 (s, 3H)

$^{13}$C NMR: (126 MHz, CDCl$_3$)

169.6, 155.0, 80.1, 51.8, 43.0, 42.5, 38.1, 32.1, 30.1, 28.5

HRMS: (ESI+/TOF)

Calcd for $C_{12}H_{22}N_2NaO_3^+$ [M+Na]+, 265.1523; found, 265.1526

**(*RS*)-4-Amino-6,6-dimethylpiperidin-2-one hydrochloride (S8)**

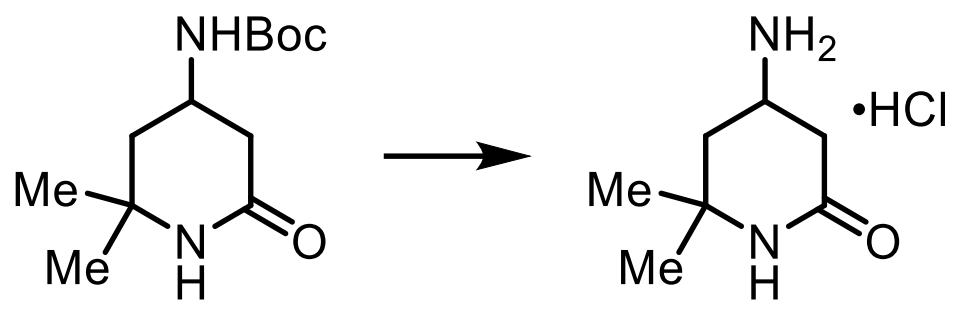


**S7** **S8**

A solution of *tert*-butyl (2,2-dimethyl-6-oxopiperidin-4-yl)carbamate **S7** (1.25 g, 0.0052 mol, 1.00 equiv) in a mixture of MTBE (10 mL) and 5 M HCl in dioxane (10 mL) was stirred for 12 h at rt. The solution was filtered, and the precipitate was washed with MTBE (100 mL) and dried to give **S8** as a colourless solid (0.90 g, 98%).

Data for **S8**:

$^1$H NMR: (500 MHz, DMSO-$d_6$)

8.29 (br s, 3H), 7.76 (s, 1H), 3.56 (s, 1H), 2.22 (dd, $J$ = 16.6, 11.4 Hz, 1H), 1.99 (d, $J$ = 12.5 Hz, 1H), 1.59 (t, $J$ = 12.7 Hz, 1H), 1.20 (s, 3H), 1.16 (s, 3H)

$^{13}$C NMR: (126 MHz, DMSO-$d_6$)

166.7, 50.5, 42.7, 35.3, 30.9, 29.0

MS: (API-ES)

143 ([M+H]+)

HRMS: (ESI+/TOF)

Calcd for $C_7H_{14}N_2NaO^+$ [M+Na]+, 165.0998; found, 165.1005

**(*RS*)-*tert*-Butyl (6-oxo-5-azaspiro[3.5]nonan-8-yl)carbamate (S9)**

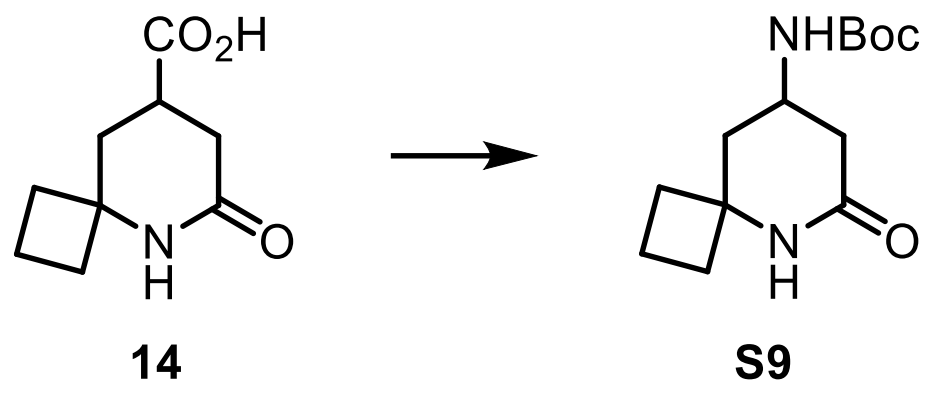

A mixture of 6-oxo-5-azaspiro[3.5]nonane-8-carboxylic acid **14** (2.00 g, 0.011 mol, 1.00 equiv), DPPA (3.33 g, 0.012 mol, 1.10 equiv), $Et_3N$ (1.21 g, 0.012 mol, 1.10 equiv) in *t*-BuOH (30 mL) was heated under reflux (in an oil bath with a thermocouple) for 10 h. The reaction mixture was cooled to rt and concentrated *in vacuo*. The residue was dissolved in MTBE (30 mL) and washed with a 20% aq. NaOH solution (2 × 10 mL), brine (10 mL), dried ($Na_2SO_4$), filtered, and concentrated *in vacuo* to give **S9** as a colourless solid (2.57 g, 92%), which was used for the next step without purification.

Data for **S9**:

$^1$H NMR: (500 MHz, $CDCl_3$)

6.17 (s, 1H), 4.48 (s, 1H), 3.89 (br s, 1H), 2.69 (dd, *J* = 17.1, 5.4 Hz, 1H), 2.38 (d, *J* = 12.7 Hz, 1H), 2.25 (s, 1H), 2.16–2.02 (m, 4H), 1.81–1.70 (m, 2H), 1.61–1.54 (m, 1H), 1.46 (s, 9H)

$^{13}$C NMR: (126 MHz, $CDCl_3$)

169.7, 155.1, 80.0, 55.2, 43.0, 39.2, 38.3, 36.9, 28.5, 13.2

HRMS: ($ESI^+$/TOF)

Calcd for $C_{13}H_{22}N_2NaO_3^+$ $[M+Na]^+$, 277.1523; found, 277.1519

**(*RS*)-8-Amino-5-azaspiro[3.5]nonan-6-one hydrochloride (S10)**

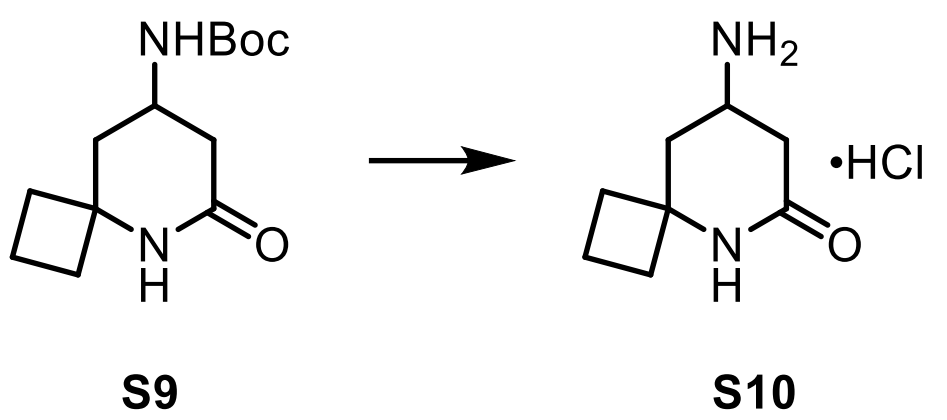

A solution of *tert*-butyl (6-oxo-5-azaspiro[3.5]nonan-8-yl)carbamate **S9** (1.00 g, 0.0039 mol) in a mixture of MTBE (10 mL) and 5 M HCl in dioxane (10 mL) was stirred for 12 h at rt. The solution was filtered, and the precipitate was washed with MTBE (5 mL) and dried to give **S10** as a colourless solid (0.67 g, 90%).

Data for **S10**:

mp: 230–231 °C

$^1$H NMR: (500 MHz, DMSO-$d_6$)

8.36 (s, 3H), 8.20 (s, 1H), 3.45 (s, 1H), 2.49–2.45 (m, 1H), 2.38 (d, $J$ = 12.5 Hz, 1H), 2.34–2.22 (m, 2H), 2.00 (t, $J$ = 8.3 Hz, 2H), 1.96–1.89 (m, 1H), 1.68 (t, $J$ = 12.4 Hz, 1H), 1.64–1.54 (m, 2H)

$^{13}$C NMR: (151 MHz, DMSO-$d_6$)

166.5, 53.9, 42.6, 35.8, 35.7, 35.6, 35.1, 12.5

MS: (API-ES)

155 ($[M+H]^+$)

HRMS: ($ESI^+$/TOF)

Calcd for $C_8H_{14}N_2NaO^+$ $[M+Na]^+$, 177.0998; found, 177.0993

**(*RS*)-(2,2-Dimethylpiperidin-4-yl)methanol hydrochloride (S11)**

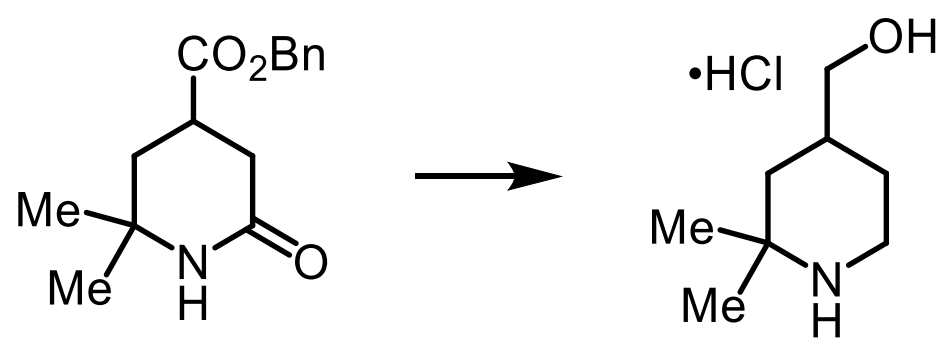


**S5** **S11**

To a suspension of $LiAlH_4$ (0.94 g, 0.0248 mol, 1.30 equiv) in THF (50 mL) was added a solution of benzyl 2,2-dimethyl-6-oxopiperidine-4-carboxylate **S5** (5.00 g, 0.0191 mol, 1.00 equiv) in THF (10 mL) at 0 °C under Ar. The resulting mixture was heated under reflux overnight. The excess of $LiAlH_4$ was neutralized with $H_2O$ (1 mL, 0.057 mol, 3.00 equiv) and 20% aq. solution of NaOH (10 mL). The suspension was filtered through $Na_2SO_4$, washed with THF, and concentrated *in vacuo*. The residue was dissolved in 5 M HCl in dioxane (20 mL), and the solution was concentrated *in vacuo* to give **S11** as a beige solid (3.05 g, 89%), which was used for the next step without purification.

Data for **S11**:

mp: 135–136 °C

<u>$^{1}$H NMR:</u> (500 MHz, DMSO-$d_6$)

9.11 (br s, 1H), 8.78 (br s, 1H), 4.64 (br s, 1H), 3.37–3.26 (m, 1H), 3.22–3.17 (m, 1H), 3.11–2.88 (m, 2H), 1.88–1.68 (m, 2H), 1.62 (d, $J$ = 13.7 Hz, 1H), 1.33 (s, 3H), 1.27 (s, 3H), 1.25–1.12 (m, 2H)

<u>$^{13}$C NMR:</u> (151 MHz, DMSO-$d_6$)

65.3, 54.0, 38.5, 37.9, 32.5, 28.3, 24.8, 20.7

<u>MS:</u> (API-ES)

144 ([M+H]$^+$)

<u>HRMS:</u> (ESI$^+$/TOF)

Calcd for $C_8H_{17}NNaO^+$ [M+Na]$^+$, 166.1202; found, 166.1211

**(*RS*)-*tert*-Butyl 4-(hydroxymethyl)-2,2-dimethylpiperidine-1-carboxylate (S12)**

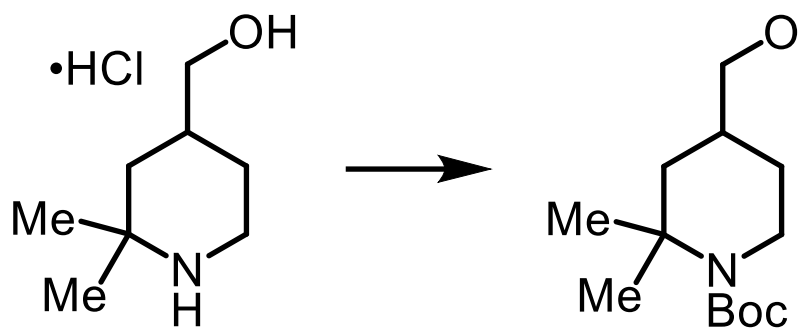


**S11** **S12**

(2,2-Dimethylpiperidin-4-yl)methanol hydrochloride **S11** (3.00 g, 0.0167 mol, 1.00 equiv) was dissolved in $CH_2Cl_2$ (30 mL) and $Et_3N$ (4.22 g, 0.0418 mol, 2.50 equiv) was added to the mixture. $Boc_2O$ (3.64 g, 0.0167 mol, 1.00 equiv) in $CH_2Cl_2$ (10 mL) was added dropwise at 10 °C (the mixture was cooled in a $H_2O$ bath). The solution was stirred for 2 h at rt. The mixture was washed with 0.5 M HCl (10 mL), $H_2O$ (10 mL), brine (10 mL), dried ($Na_2SO_4$), filtered, and concentrated *in vacuo* to give **S12** as a yellow oil (3.86 g, 95%), which was used for the next step without purification.

<u>Data for **S12**:</u>

<u>$^{1}$H NMR:</u> (500 MHz, DMSO-$d_6$)

4.49 (t, $J$ = 5.2 Hz, 1H), 3.58–3.47 (m, 1H), 3.27–3.18 (m, 2H), 3.15–3.08 (m, 1H), 1.75–1.62 (m, 2H), 1.48–1.44 (m, 1H), 1.42 (s, 3H), 1.39 (s, 9H), 1.24 (s, 3H), 1.30–1.16 (m, 1H), 1.10–1.01 (m, 1H)

<u>$^{13}$C NMR:</u> (151 MHz, DMSO-$d_6$)

155.1, 78.3, 65.8, 53.9, 42.8, 40.8, 32.7, 30.7, 28.2, 27.1, 23.1

HRMS: (ESI$^+$/TOF)

Calcd for $C_{13}H_{25}NNaO_3^+$ [M+Na]$^+$, 266.1727; found, 266.1713

**(*RS*)-*tert*-Butyl 4-formyl-2,2-dimethylpiperidine-1-carboxylate (16)**

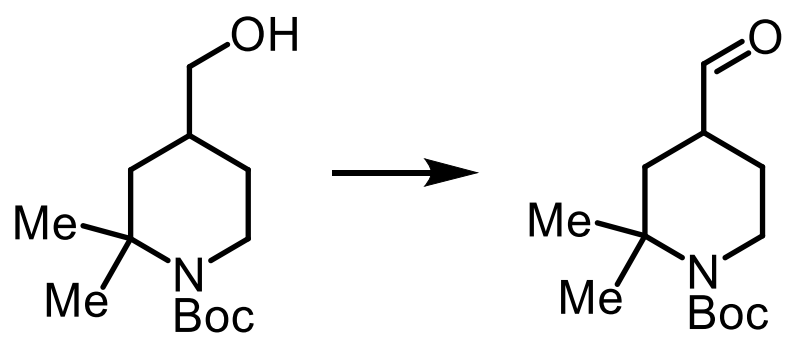

**S12** **16**

To a stirred solution of oxalyl chloride (1.36 g, 0.0107 mol, 1.30 equiv) in 20 mL of $CH_2Cl_2$ (20 mL) was added a solution of DMSO (0.97 g, 0.0124 mol, 1.51 equiv) in $CH_2Cl_2$ (5 mL) dropwise at –78 °C. The resulting mixture was stirred at the same temperature for 1 h, and the solution of (*tert*-butyl 4-(hydroxymethyl)-2,2-dimethylpiperidine-1-carboxylate **S12** (2.00 g, 0.00823 mol, 1.00 equiv) in $CH_2Cl_2$ (20 mL) was added dropwise. The mixture was stirred for 4 h at –60 °C and $Et_3N$ (3.33 g, 0.033 mol, 4.00 equiv) was added. The resulting solution was slowly warmed and stirred at rt overnight. The organic layer was washed with $H_2O$ (2 × 20 mL), brine (10 mL), dried ($Na_2SO_4$), filtered, and concentrated *in vacuo* to give **16** as a yellow oil (1.69 g, 85%), which was used for the next step without purification.

Data for **16**:

$^1$H NMR: (500 MHz, $CDCl_3$)

9.67 (s, 1H), 3.60–3.52 (m, 1H), 3.46–3.35 (m, 1H), 2.69–2.54 (m, 1H), 1.97–1.87 (m, 1H), 1.84–1.69 (m, 3H), 1.49 (s, 3H), 1.46 (s, 12H)

$^{13}$C NMR: (126 MHz, $CDCl_3$)

203.1, 155.9, 79.9, 54.3, 43.5, 40.2, 38.8, 30.0, 28.7, 24.5, 23.4

HRMS: (ESI$^+$/TOF)

Not detected under the experimental conditions

**(*RS*)-2,2-Dimethylpiperidine-4-carboxylic acid hydrochloride (S13)**

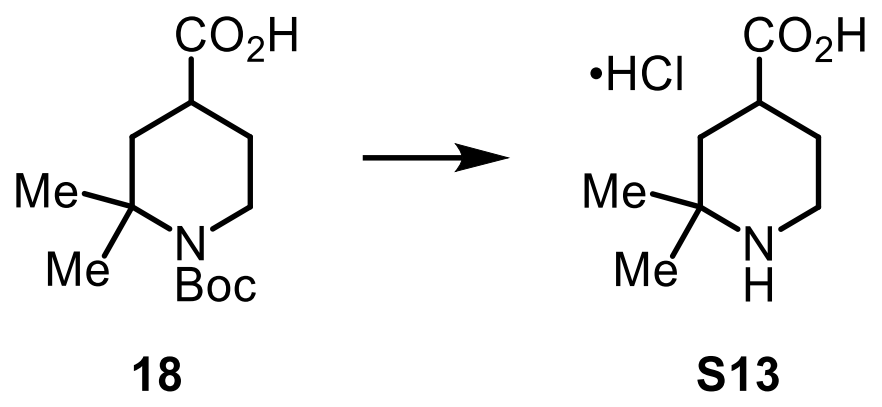

A solution of 1-(*tert*-butoxycarbonyl)-2,2-dimethylpiperidine-4-carboxylic acid **18** (0.80 g, 0.00311 mol, 1.00 equiv) in a mixture of MTBE (10 mL) and 5 M HCl in dioxane (10 mL) was stirred for 12 h at rt. The solution was filtered, and the precipitate was washed with MTBE (10 mL) and dried to give **S13** as a beige solid (0.54 g, 90%).

Data for **S13**:

mp: 176–177 °C

$^1$H NMR: (500 MHz, $D_2O$)

3.37–3.29 (m, 1H), 3.29–3.19 (m, 1H), 2.90 (tt, $J$ = 12.3, 3.7 Hz, 1H), 2.19 (d, $J$ = 14.3 Hz, 1H), 2.09 (d, $J$ = 14.3 Hz, 1H), 1.81-1.68 (m, 2H), 1.44 (s, 3H), 1.40 (s, 3H)

$^{13}$C NMR: (126 MHz, DMSO-$d_6$)

174.9, 53.8, 38.0, 36.8, 35.1, 27.7, 24.1, 20.5

MS: (API-ES)

158 ([M+H]$^+$)

HRMS: (ESI$^+$/TOF)

Calcd for $C_8H_{16}NO_2^+$ [M+H]$^+$, 158.1176; found, 158.1177

**(*RS*)-1-(((9*H*-Fluoren-9-yl)methoxy)carbonyl)-2,2-dimethylpiperidine-4-carboxylic acid (S14)**

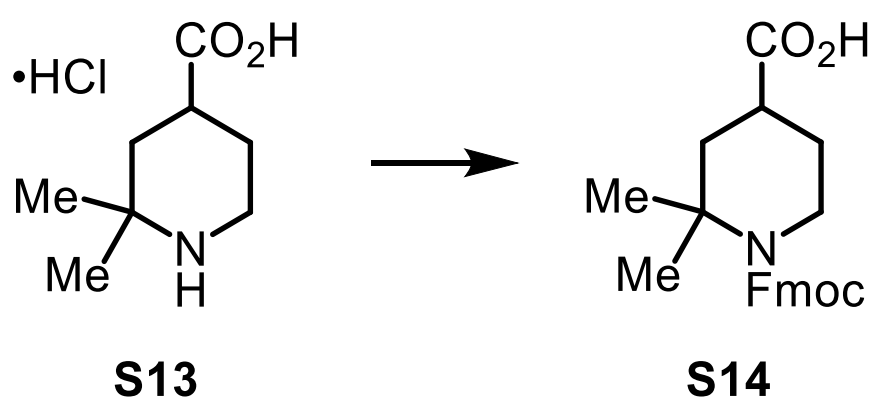

$Na_2CO_3$ (1.36 g, 0.0129 mol, 5.00 equiv), 1,4-dioxane (10 mL) and 9-fluorenylmethyloxycarbonyl chloride (0.73 g, 0.00284 mol, 1.10 equiv) were added to a solution of 2,2-dimethylpiperidine-4-carboxylic acid hydrochloride **S13** (0.50 g, 0.00258 mol, 1.00 equiv) in $H_2O$ (10 mL). After stirring for 16 h at rt, the reaction mixture was poured into $H_2O$ (10 mL), and the solution was extracted with EtOAc

(3 × 10 mL). The aqueous layer was acidified with 2 M aq HCl solution to pH 2. The mixture was extracted with EtOAc (3 × 10 mL) and the combined organic layers were dried ($Na_2SO_4$), filtered, and concentrated *in vacuo* to give **S14** as a colourless solid (0.88 g, 90%).

Data for **S14**:

mp: 122–123 °C

$^1$H NMR: (500 MHz, DMSO-$d_6$)

12.25 (br s, 1H), 7.88 (d, $J$ = 7.4 Hz, 2H), 7.62 (d, $J$ = 5.9 Hz, 2H), 7.40 (t, $J$ = 7.3 Hz, 2H), 7.33 (t, $J$ = 7.2 Hz, 2H), 4.53–4.37 (m, 2H), 4.33–4.23 (m, 1H), 3.53–3.42 (m, 1H), 3.14–3.00 (m, 1H), 1.80–1.69 (m, 1H), 1.65–1.42 (m, 3H), 1.09 (s, 3H), 0.97 (s, 3H)

$^{13}$C NMR: (101 MHz, DMSO-$d_6$)

176.3, 155.2, 144.2, 144.1, 140.9, 127.5, 127.1, 124.8, 124.7, 120.1, 65.8, 54.0, 46.8, 41.0, 35.2, 29.0, 26.2, 22.7

MS: (API-ES)

380 ($[M+H]^+$)

HRMS: ($ESI^+$/TOF)

Calcd for $C_{23}H_{25}NNaO_4^+$ $[M+Na]^+$, 402.1676; found, 402.1667

**(*RS*)-Methyl 2,2-dimethylpiperidine-4-carboxylate (S15)**

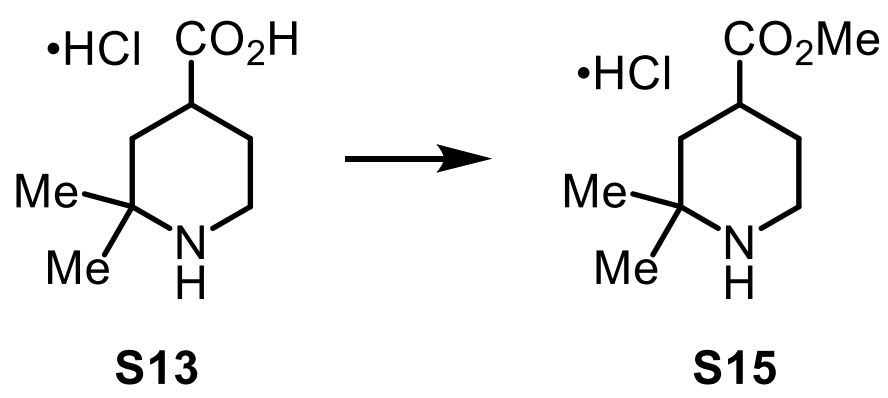


To a solution of 2,2-dimethylpiperidine-4-carboxylic acid hydrochloride **S13** (0.30 g, 0.00155 mol, 1.00 equiv) in MeOH (5 mL) was added $SOCl_2$ (0.37 g, 0.0031 mol, 2.00 equiv) dropwise at 0–5 °C (ice-water bath). The resulting mixture was heated under reflux for 2 h and then concentrated *in vacuo*. The residue was washed with hexane (3 mL) and dried to give **S15** as a brown solid (0.31 g, 95%).

Data for **S15**:

mp: 147–149 °C

<u>$^1$H NMR:</u> (500 MHz, DMSO-$d_6$)

9.14 (br s, 1H), 8.95 (br s, 1H), 3.63 (s, 3H), 3.14–2.99 (m, 2H), 2.86–2.77 (m, 1H), 1.94 (d, $J$ = 13.0 Hz, 1H), 1.87 (d, $J$ = 13.6 Hz, 1H), 1.73–1.57 (m, 2H), 1.35 (s, 3H), 1.29 (s, 3H)

<u>$^{13}$C NMR:</u> (126 MHz, DMSO-$d_6$)

173.8, 53.7, 51.7, 37.8, 36.6, 35.1, 27.6, 24.0, 20.5

<u>HRMS:</u> (ESI$^+$/TOF)

Calcd for $C_9H_{18}NO_2^+$ [M+H]$^+$, 172.1332; found, 172.1334

**(*RS*)-*tert*-Butyl 4-ethynyl-2,2-dimethylpiperidine-1-carboxylate (17)**

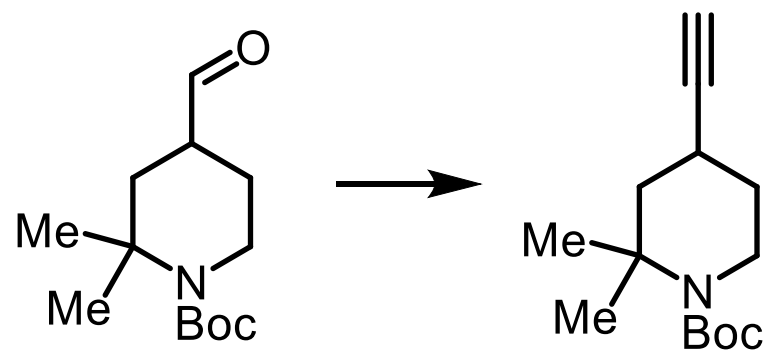


**16** **17**

To a solution of *tert*-butyl 4-formyl-2,2-dimethylpiperidine-1-carboxylate **16** (0.51 g, 0.0021 mol, 1.00 equiv) in MeOH (10 mL), $K_2CO_3$ (0.57 g, 0.004 mol, 2.00 equiv) was added in one portion. The mixture was cooled to 0 °C (ice-water bath), and dimethyldiazomethylphosphonate (Ohira-Bestmann reagent) (0.48 g, 0.0025 mol, 1.20 equiv) was added in portions. The reaction mixture was left to stir at rt overnight. The mixture was diluted with $H_2O$ (10 mL), partially concentrated *in vacuo*, and extracted with MTBE (3 × 5 mL). The combined organic layers were dried ($Na_2SO_4$), filtered, and concentrated *in vacuo* to give **17** as a yellow oil (0.40 g, 81%).

<u>Data for **17**:</u>

<u>$^1$H NMR:</u> (500 MHz, $CDCl_3$)

3.78–3.69 (m, 1H), 3.22–3.10 (m, 1H), 2.64–2.52 (m, 1H), 2.06 (d, $J$ = 2.4 Hz, 1H), 2.03–1.94 (m, 1H), 1.70 (d, $J$ = 7.9 Hz, 2H), 1.62–1.56 (m, 1H), 1.51 (s, 3H), 1.45 (s, 9H), 1.29 (s, 3H)

<u>$^{13}$C NMR:</u> (126 MHz, $CDCl_3$)

156.3, 87.9, 79.7, 68.6, 54.5, 46.1, 41.0, 31.4, 30.4, 28.7, 23.6, 23.3

<u>GC/MS:</u> (EI)

237 ([M]$^+$)

HRMS: (ESI$^+$/TOF)

Calcd for $C_{14}H_{24}NO_2^+$ [M+H]$^+$, 238.1802; found, 238.1805

**(*RS*)-1-(*tert*-Butoxycarbonyl)-2,2-dimethylpiperidine-4-carboxylic acid (18)**

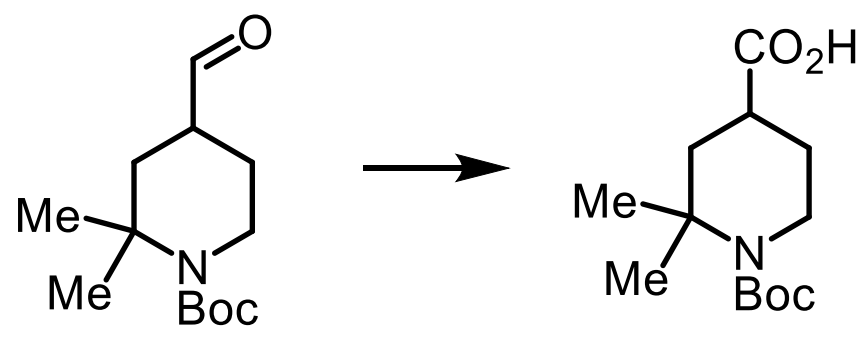

To a stirred solution of *tert*-butyl 4-formyl-2,2-dimethylpiperidine-1-carboxylate **16** (1.00 g, 0.00415 mol, 1.00 equiv), 2-methylbut-2-ene (1.23 g, 0.0176 mol, 4.25 equiv) and $NaH_2PO_4$ (0.55 g, 0.00457 mol, 1.10 equiv) in a mixture of $H_2O$ (15 mL) and *t*-BuOH (15 mL) was added a solution of $NaClO_2$ (0.41 g, 0.00457 mol, 1.10 equiv) in $H_2O$ (2 mL) dropwise at 0 °C (ice-water bath). The reaction mixture was slowly warmed to rt and stirred for 4 h. The solution was diluted with $H_2O$ (20 mL), acidified with $NaHSO_4$ to pH ~ 6, and extracted with EtOAc (3 × 10 mL). The combined organic layers were washed with $H_2O$ (2 × 10 mL), brine (10 mL), dried ($Na_2SO_4$), filtered, and concentrated *in vacuo* to give **18** as a yellow solid (0.86 g, 81%).

Data for **18**:

mp: 80–81 °C

$^1$H NMR: (500 MHz, $CDCl_3$)

3.72–3.61 (m, 1H), 3.36–3.23 (m, 1H), 2.74–2.63 (m, 1H), 2.04–1.92 (m, 1H), 1.89–1.67 (m, 3H), 1.51 (s, 3H), 1.46 (s, 9H), 1.34 (s, 3H)

$^{13}$C NMR: (126 MHz, DMSO-$d_6$)

176.2, 155.0, 78.6, 53.7, 41.5, 35.5, 29.9, 28.1, 26.4, 23.0

HRMS: (ESI$^+$/TOF)

Calcd for $C_9H_{15}NNaO_4^+$ [M–*t*Bu+Na]$^+$, 224.0893; found, 224.0891

**(*RS*)-*tert*-Butyl 4-amino-2,2-dimethylpiperidine-1-carboxylate (19)**

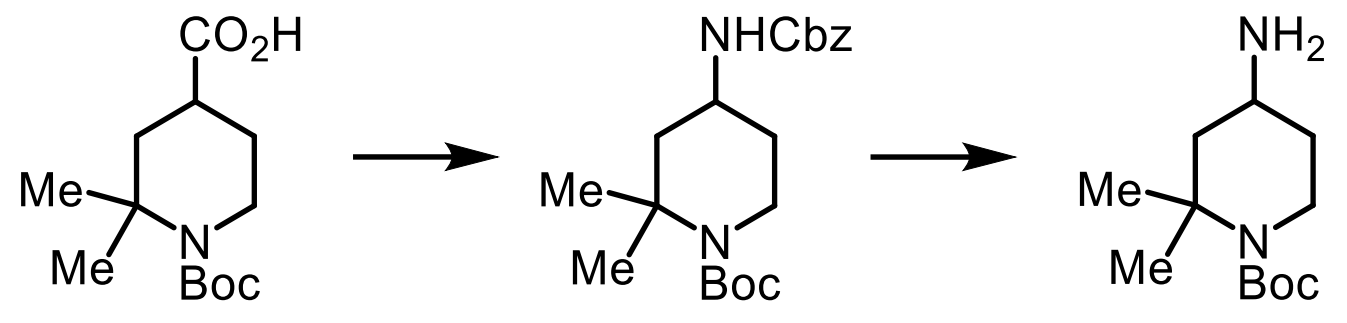

16 S16 19

1-(*tert*-Butoxycarbonyl)-2,2-dimethylpiperidine-4-carboxylic acid **16** (1.70 g, 0.0066 mol, 1.00 equiv) was dissolved in anhydrous toluene (25 mL), and $Et_3N$ (0.80 g, 0.0079 mol, 1.20 equiv) was added. The mixture was heated to 80 °C, and DPPA (2.00 g, 0.0073 mol, 1.10 equiv) was added dropwise. The reaction mixture was then heated at 100 °C for 2 h. Benzyl alcohol (1.05 g, 0.0099 mol, 1.50 equiv) was then added to the reaction mixture, and heating was continued overnight. The reaction mixture was concentrated *in vacuo* and the residue was dissolved in MTBE (20 mL) and washed with a 20% NaOH aq solution (2 × 5 mL). The organic layer was dried ($Na_2SO_4$), filtered, and concentrated *in vacuo* to give **S16** as a yellow oil (2.13 g, 89%), which was used for the next step without purification.

*tert*-Butyl 4-(((benzyloxy)carbonyl)amino)-2,2-dimethylpiperidine-1-carboxylate **S16** (0.50 g, 0.00138 mol, 1.00 equiv) was dissolved in MeOH (5 mL), and Pd/C (10%, 0.06 g) was added. The reaction vessel was evacuated and connected to a hydrogen balloon. The mixture was stirred overnight at rt. The next morning, the reaction mixture was filtered to remove the catalyst, and the filtrate was concentrated *in vacuo* to give **19** as a yellow oil (0.28 g, 90%).

Data for **19**:

$^{1}$H NMR: (500 MHz, $CDCl_3$)

3.85 (dt, $J$ = 13.7, 4.7 Hz, 1H), 3.12–3.02 (m, 1H), 3.00–2.88 (m, 1H), 1.94–1.84 (m, 1H), 1.67–1.58 (m, 1H), 1.52 (s, 3H), 1.45 (s, 9H), 1.40–1.30 (m, 3H), 1.27 (s, 3H), 1.25–1.19 (m, 1H)

$^{13}$C NMR: (126 MHz, $CDCl_3$)

156.4, 79.6, 55.1, 51.1, 44.9, 41.6, 35.6, 31.8, 28.7, 23.2

HRMS: (ESI$^{+}$/TOF)

Calcd for $C_{12}H_{24}N_2NaO_2^{+}$ [M+Na]$^{+}$, 251.1730; found, 251.1729

## I. Supplementary References

1. Jobelius, H. H.; Scharff, H.-D. Hydrazoic acid and azides. Ullmann's Encyclopedia of Industrial Chemistry (Wiley-VCH, 2000).

2. Shalibor, A.; Modarresi-Alam, A. R. A green and simple process for preparation of tetraalkylammonium azide with excellent environmental factor: comparison of batch and flow column reactor. *Org. Proc. Res. Dev.* **2018**, *22*, 1753–1760.

3. Chernowsky, C. P.; Chmiel, A. F.; Wickens, Z. K. Electrochemical activation of diverse conventional photoredox catalysts induces potent photoreductant activity. *Angew. Chem. Int. Ed.* **2021**, *60*, 21418–21425.

4. Liu, D.; Xiao, W.; Li, H.; Zhang, Y.; Yuan, S.; Li, C.; Dong, S.; Wang, C. Discovery of itaconate-mediated lysine acylation. *J. Am. Chem. Soc.* **2023**, *145*, 12673–12681.

5. RDKit: Open-source cheminformatics. https://www.rdkit.org. (Version 2025.3.5, https://doi.org/10.5281/zenodo.16996017).

6. Pedreogosa, F.; Varoquaux, G.; Gramfort, A.; Michel, V.; Thirion, B.; Grisel, O.; Blondel, M.; Prettenhofer, P.; Weiss, R.; Dubourg, V.; Vanderplas, J.; Passos, A.; Cournapeau, D.; Brucher, M.; Perrot, M.; Duchesnay, E. Scikit-learn: Machine learning in Python. *J. Mach. Learn. Res.* **2011**, *12*, 2825–2830.

7. McInnes, L; Healy, J, UMAP: Uniform manifold approximation and projection for dimension reduction, ArXiv e-prints 1802.03426, 2018.

8. (a) Friedman, D.; Dieng, A. B. The Vendi score: A diversity evaluation metric for machine learning, ArXiv e-prints, 2023. (b) Pasarkar, A. P.; Dieng, A. B. Cousins of the Vendi Score: A family of similarity-based diversity metrics for science and machine learning. ArXiv e-prints, 2024. (c) https://github.com/vertaix/Vendi-Score.

9. O. V. Dolomanov, L. J. Bourhis, R. J. Gildea, J. A. K. Howard, H. Puschmann. OLEX2 : a complete structure solution, refinement and analysis program. *J. Appl. Crystallogr*. *42*, **2009**, 339–341.

10. G. M. Sheldrick. SHELXT – Integrated space-group and crystal-structure determination. *Acta Crystallogr. Sect. A Found. Adv. 71*, **2015** 3–8.

11. G. M. Sheldrick. Crystal structure refinement with SHELXL. *Acta Crystallogr. Sect. C*, *71*, **2015**, 3–8.

# J. $^{1}H$ and $^{13}C$ NMR Spectra

## $^{1}H$ NMR (400 MHz, $CDCl_3$)

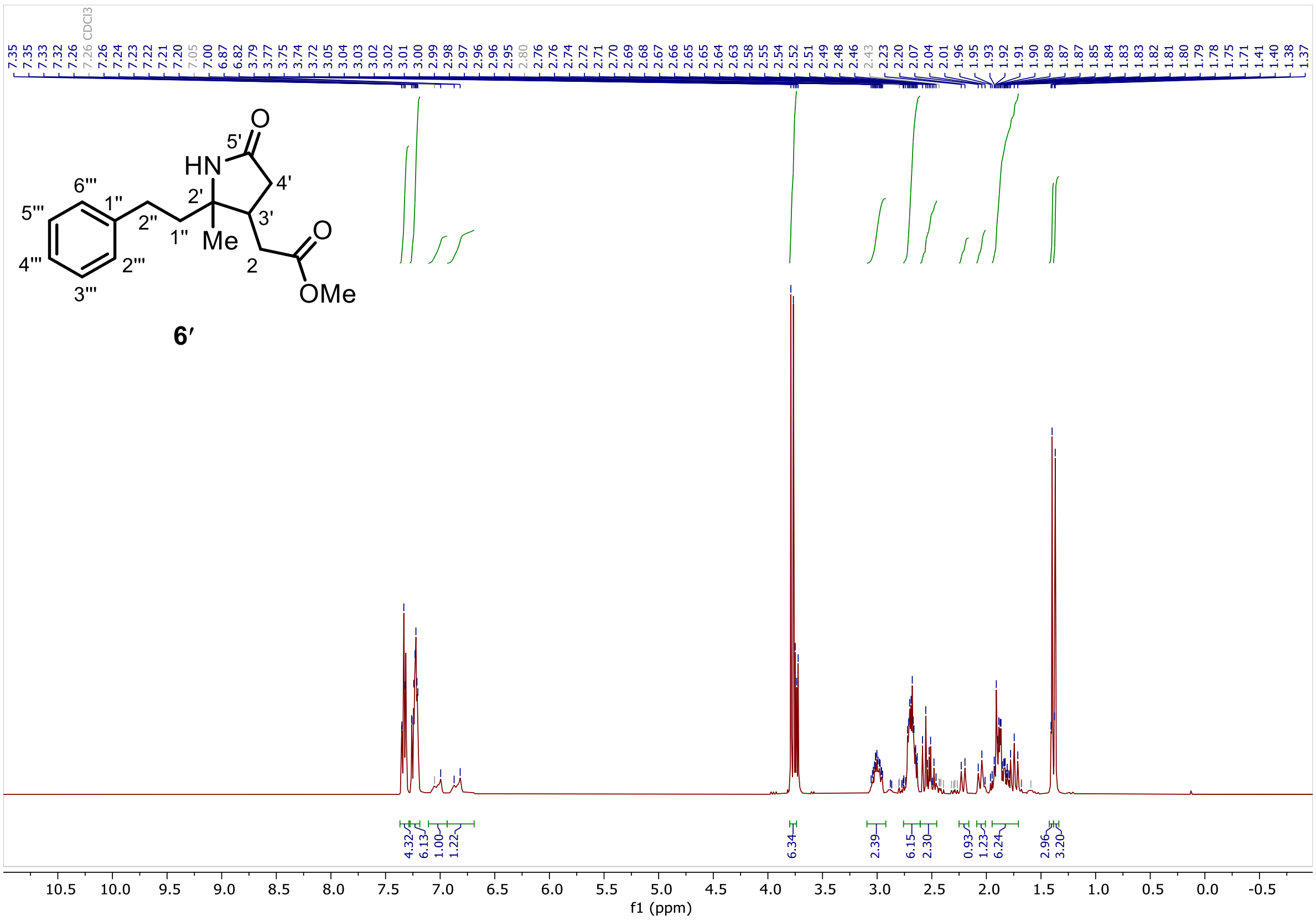

## $^{13}C$ NMR (101 MHz, $CDCl_3$)

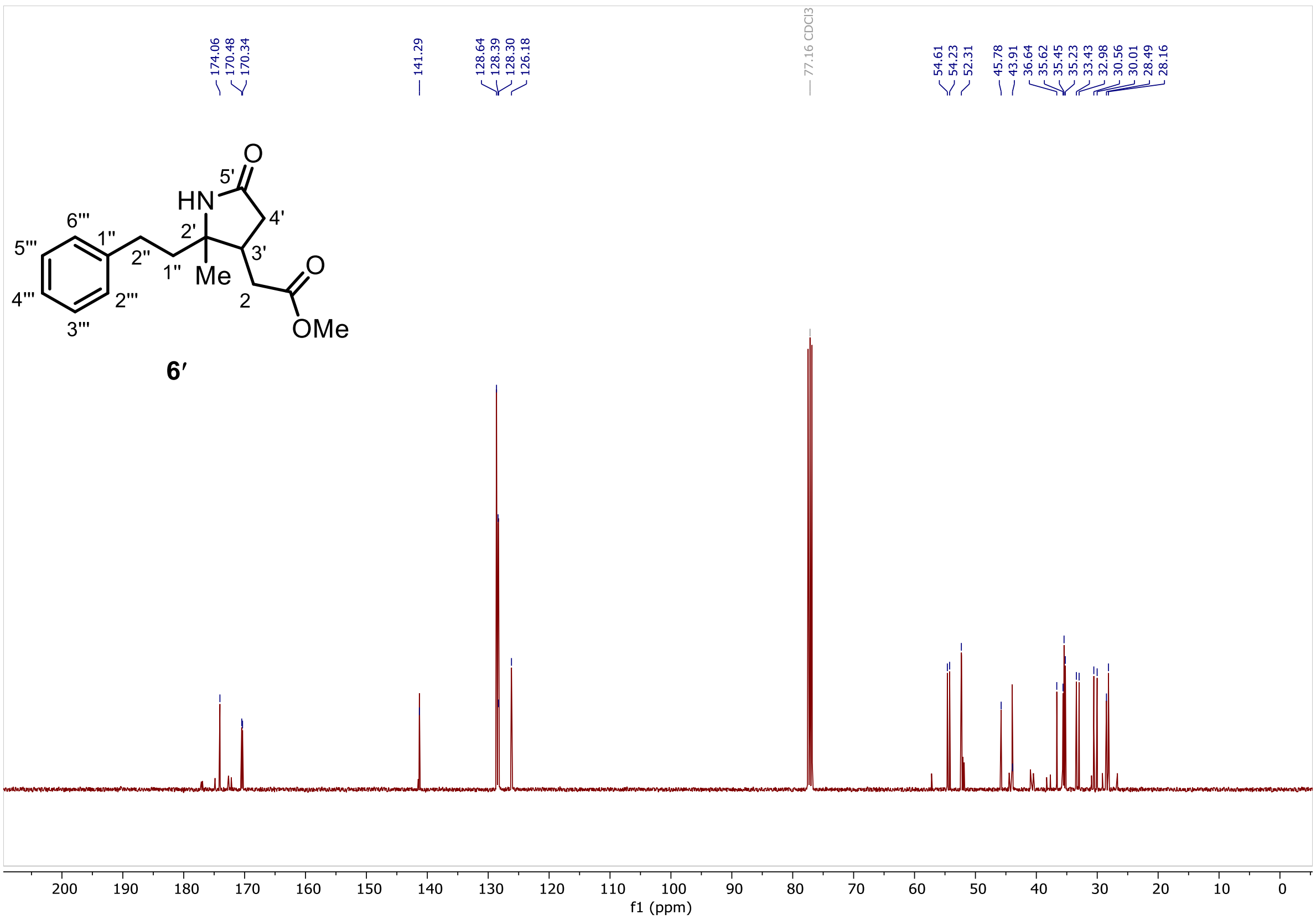

**COSY (500 MHz, CDCl$_3$)**

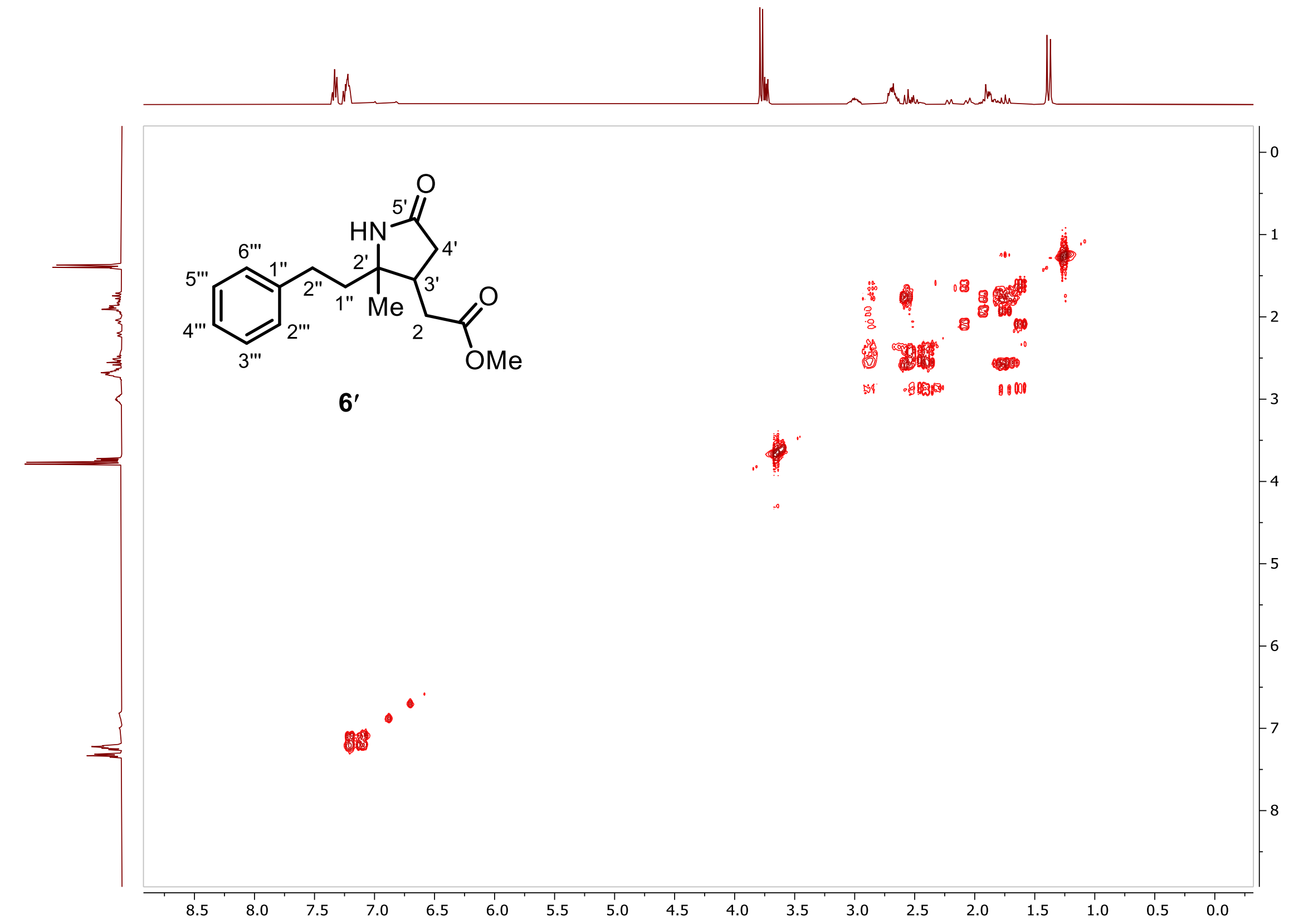

**HSQC ($^1$H 500 MHz/$^{13}$C 126 MHz, CDCl$_3$)**

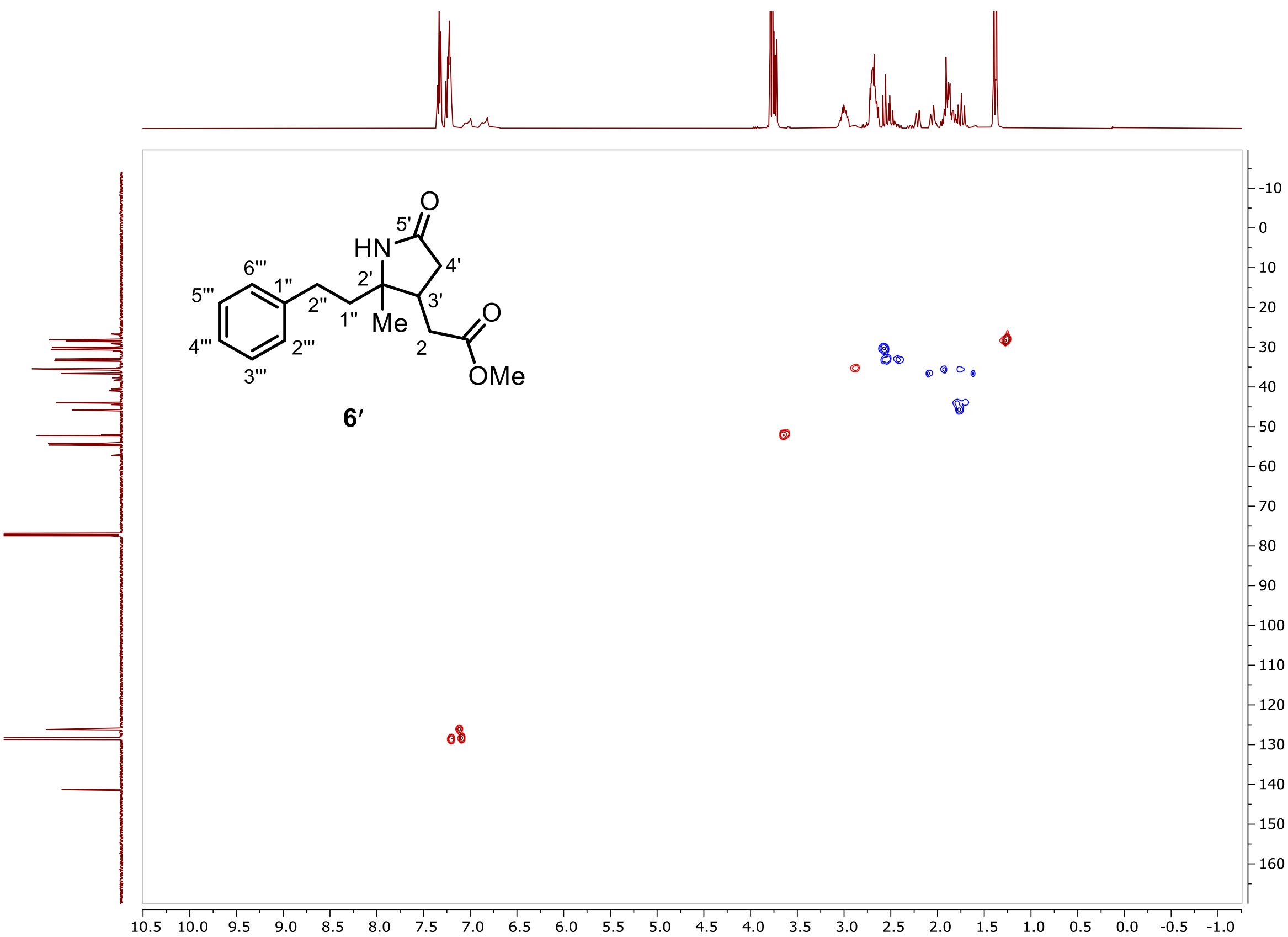

## $^{1}$H NMR (400 MHz, CDCl$_3$)

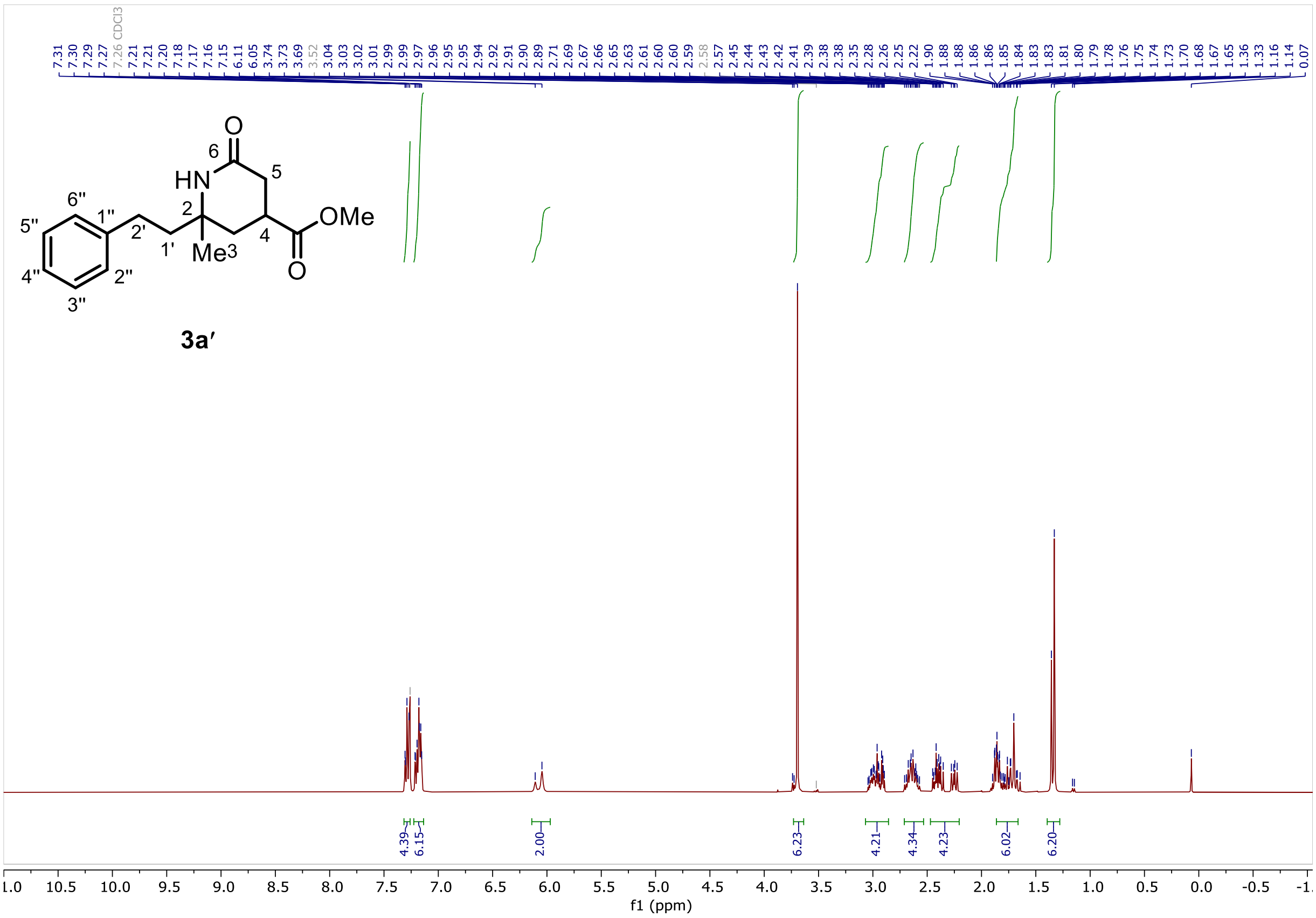


## $^{13}$C NMR (101 MHz, CDCl$_3$)

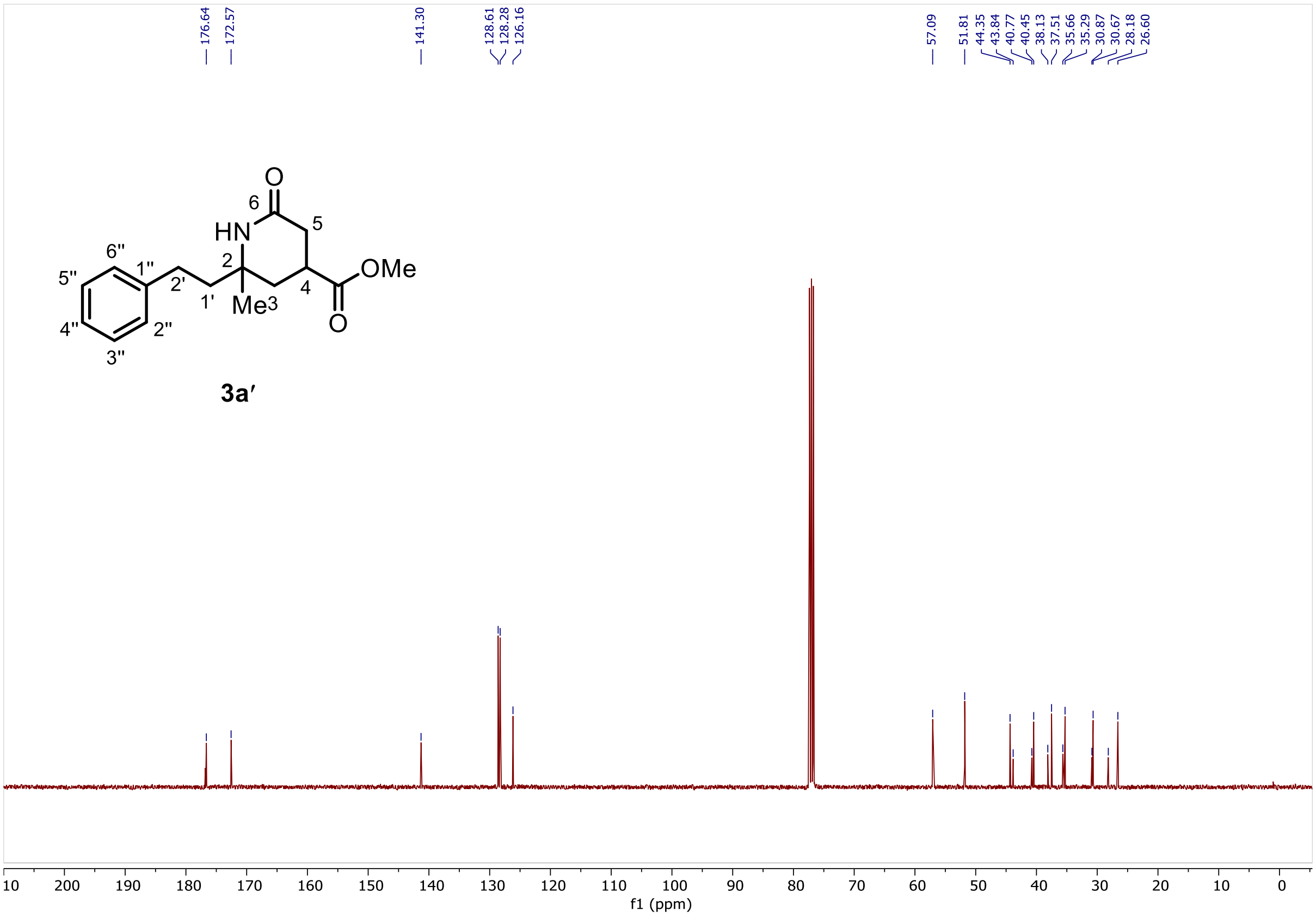

**COSY (500 MHz, $CDCl_3$)**

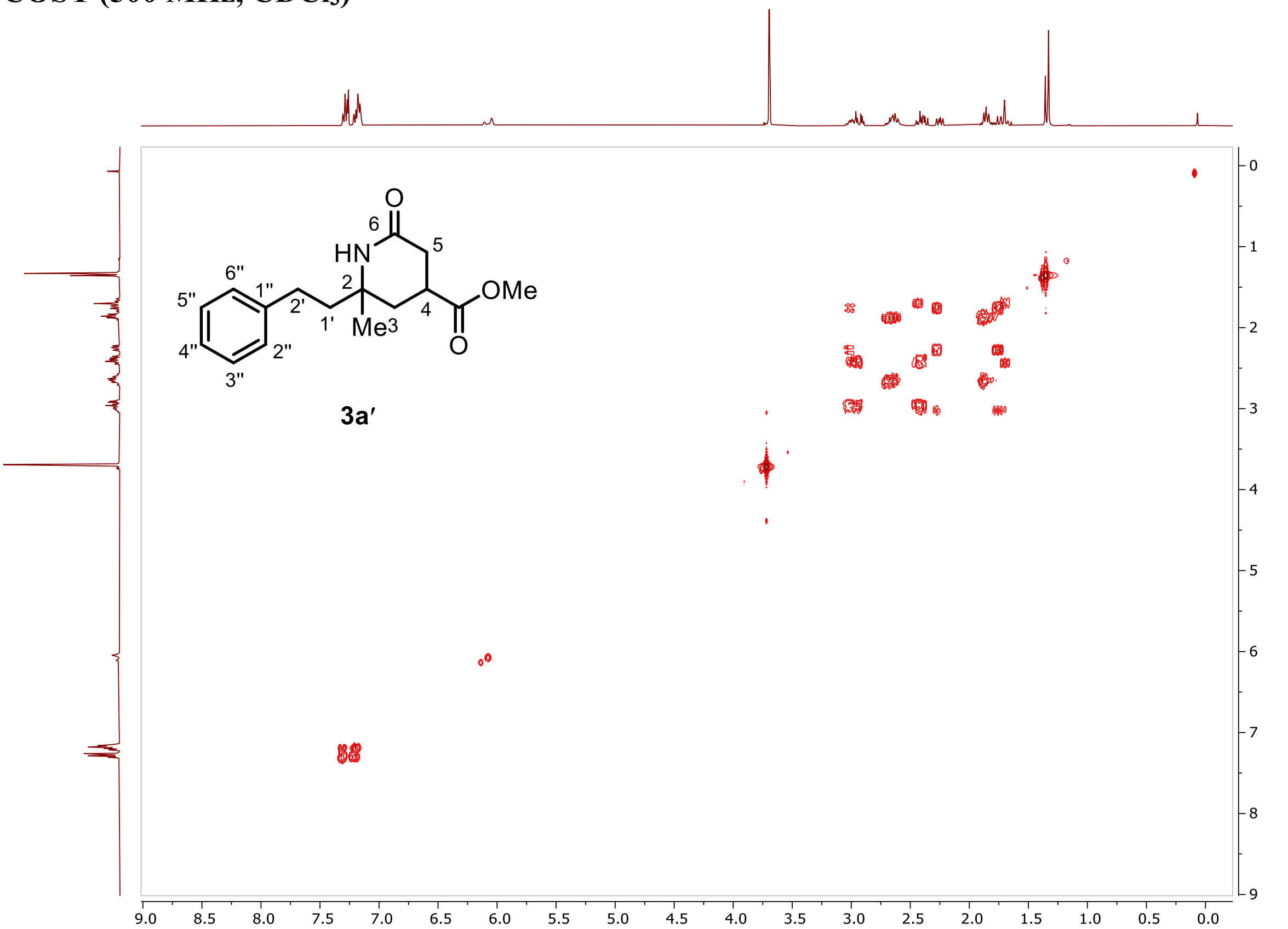


**HSQC ($^1$H 500 MHz/$^{13}$C 126 MHz, $CDCl_3$)**

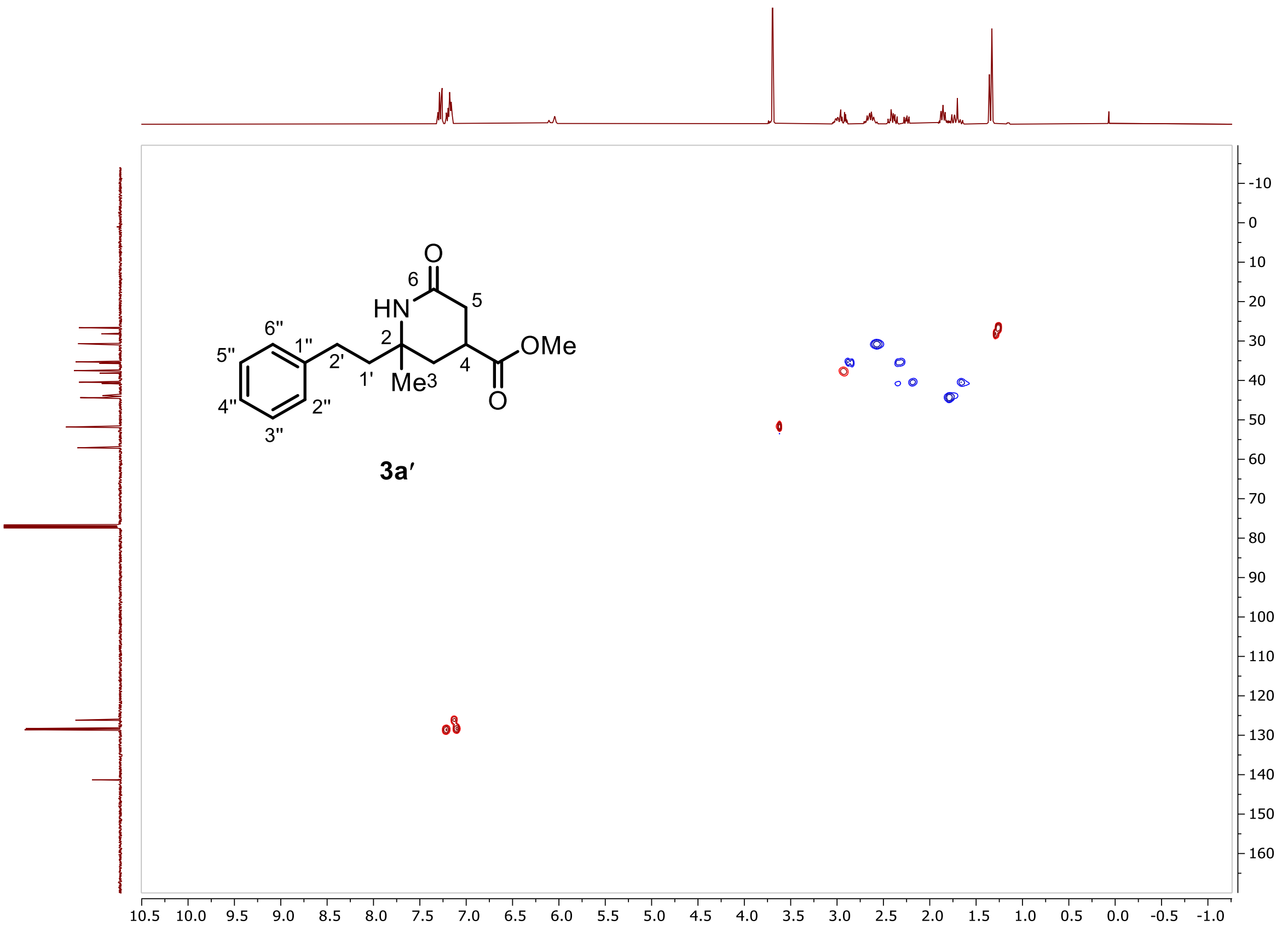

**$^{1}$H NMR (500 MHz, CDCl$_3$)**

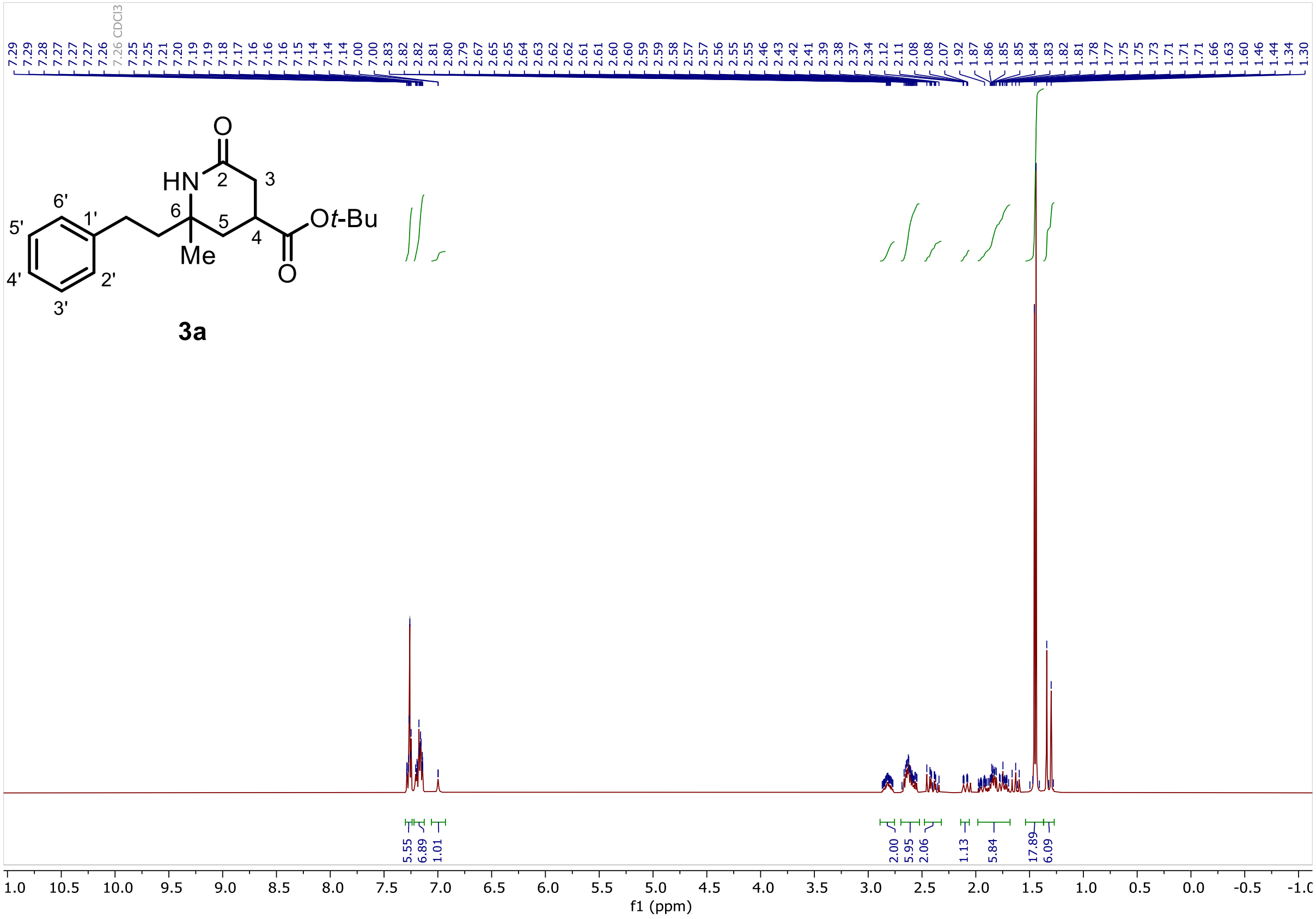

**$^{13}$C NMR (126 MHz, CDCl$_3$)**

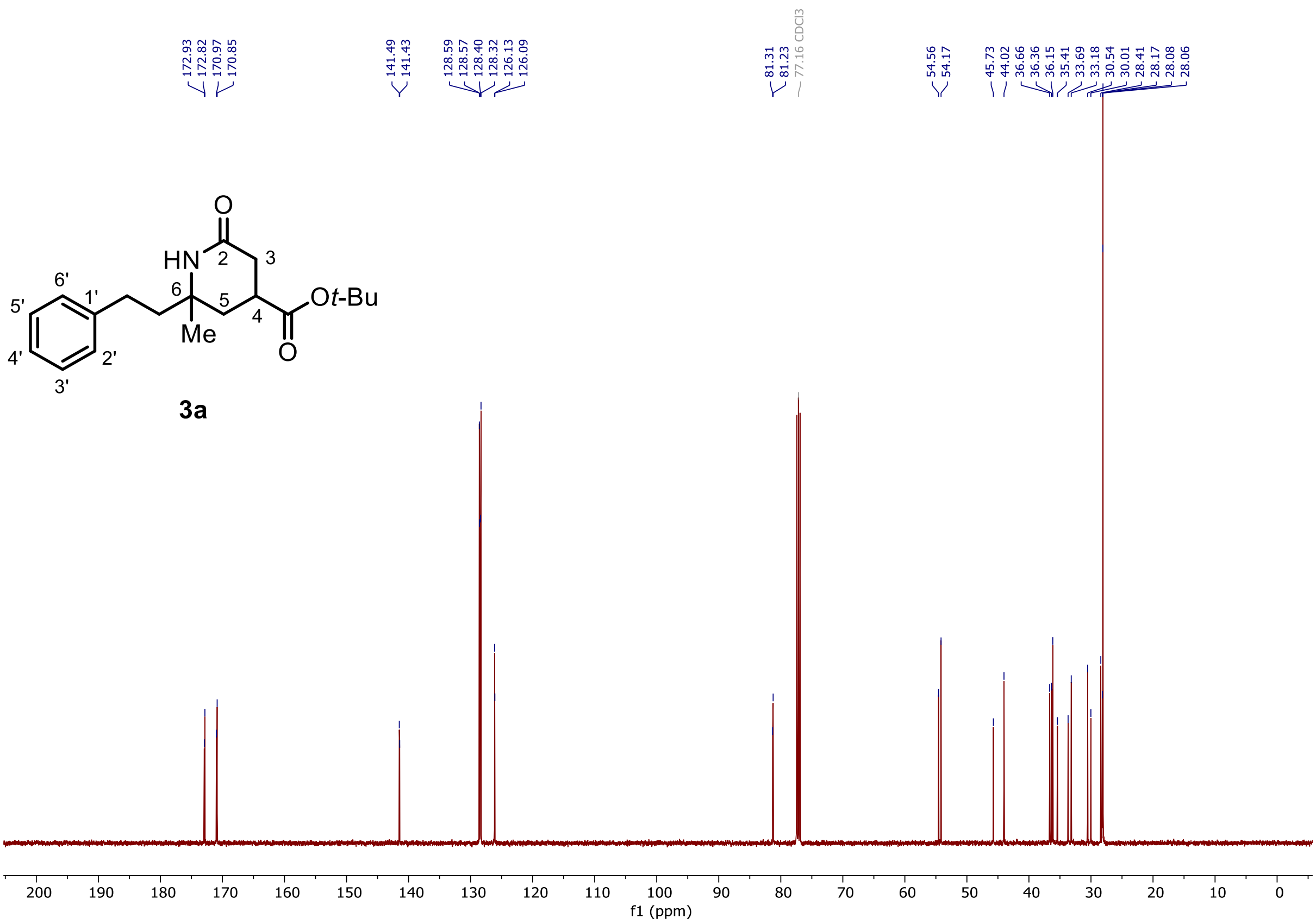

**COSY (500 MHz, $CDCl_3$)**

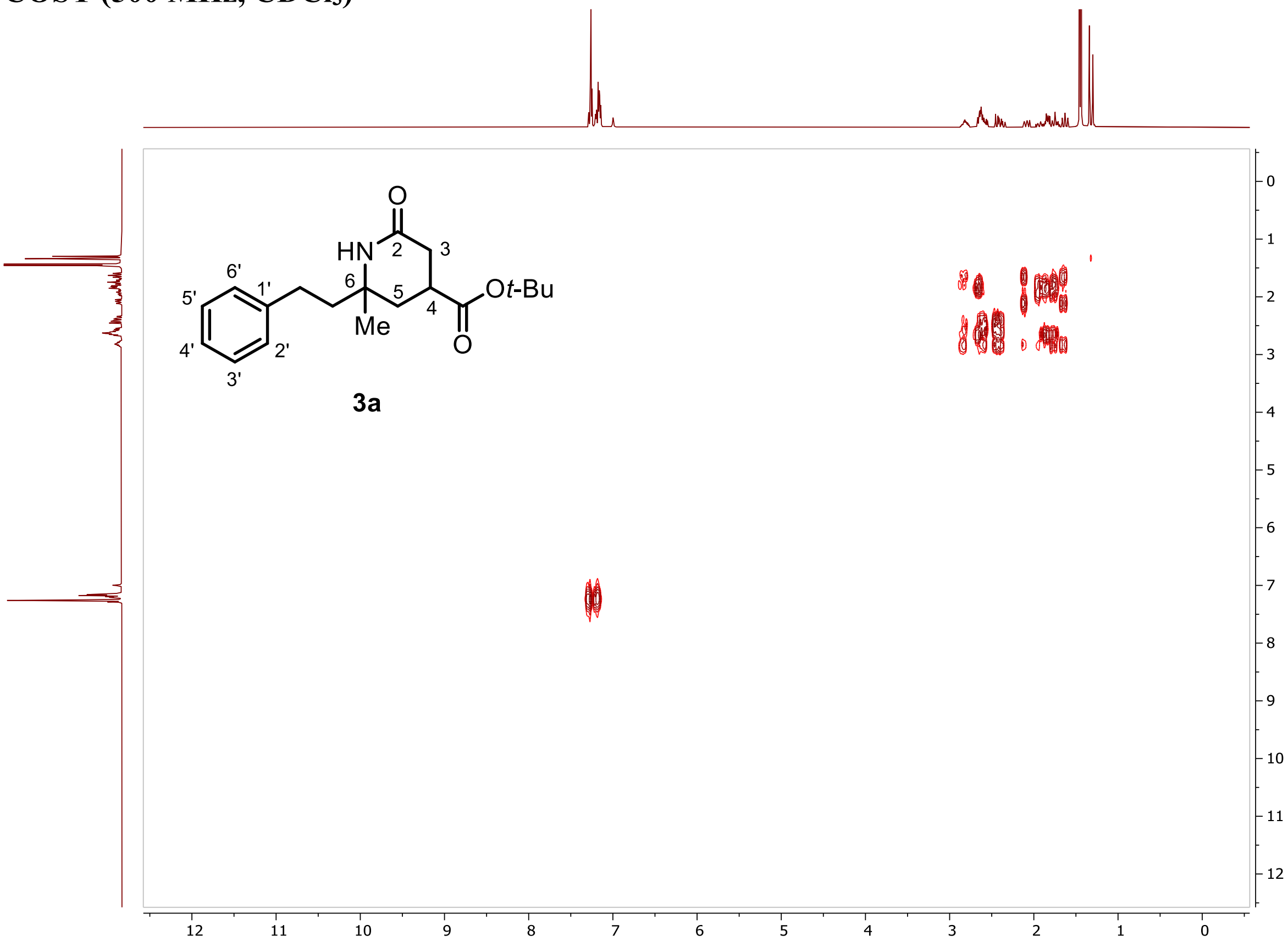


**HSQC ($^{1}H$ 500 MHz/$^{13}C$ 126 MHz, $CDCl_3$)**

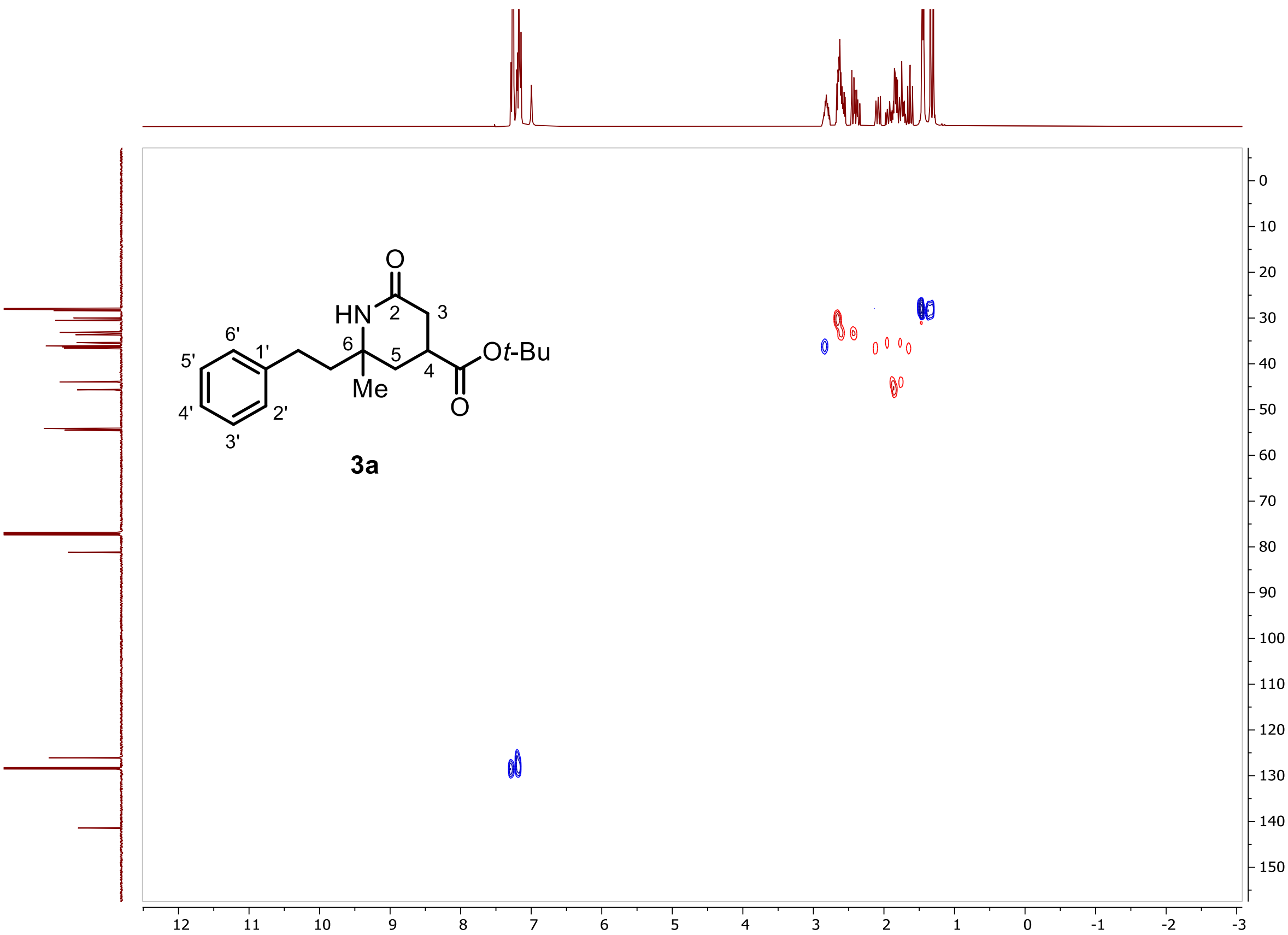

**$^{1}$H NMR (500 MHz, CDCl$_3$)**

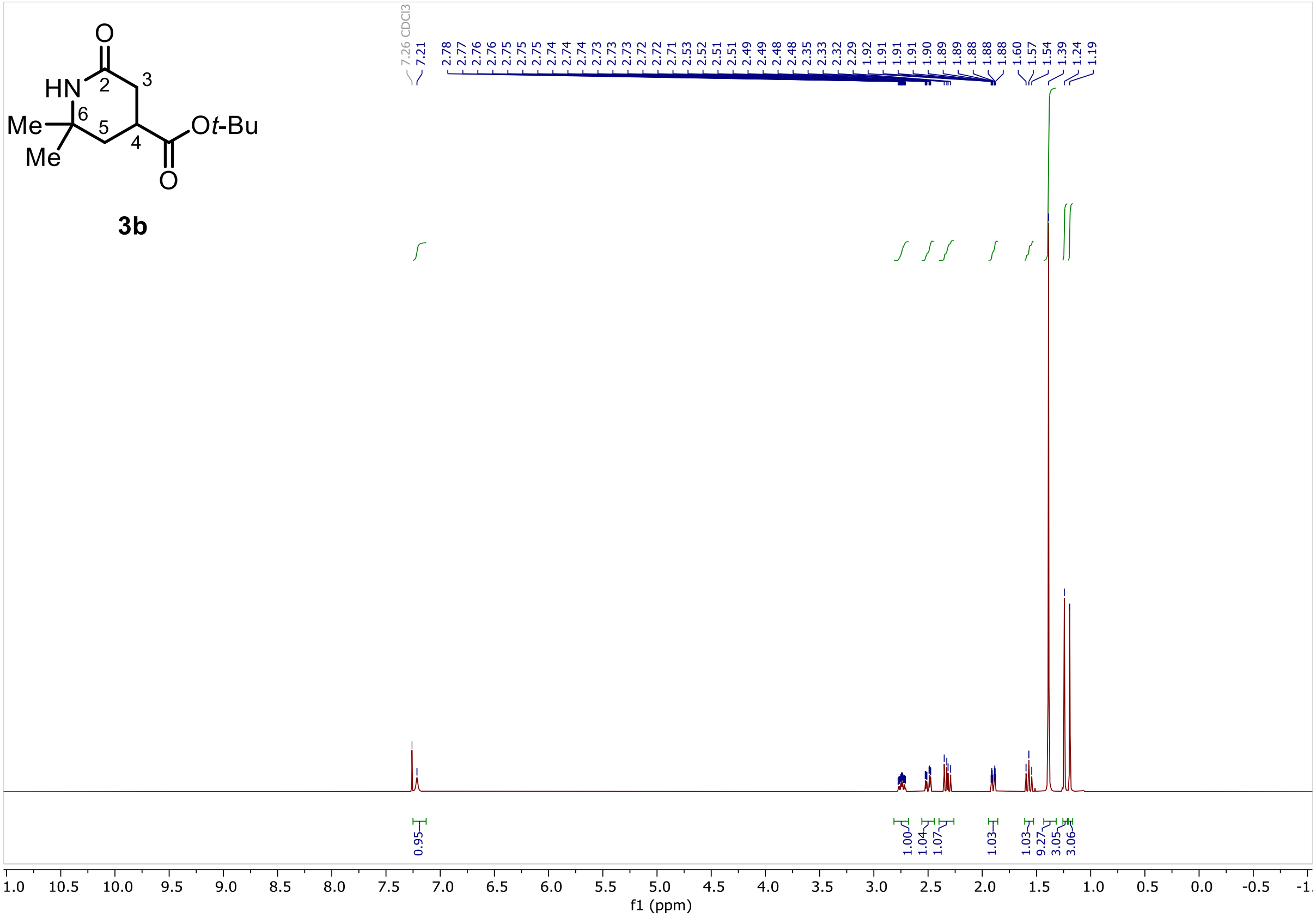


**$^{13}$C NMR (126 MHz, CDCl$_3$)**

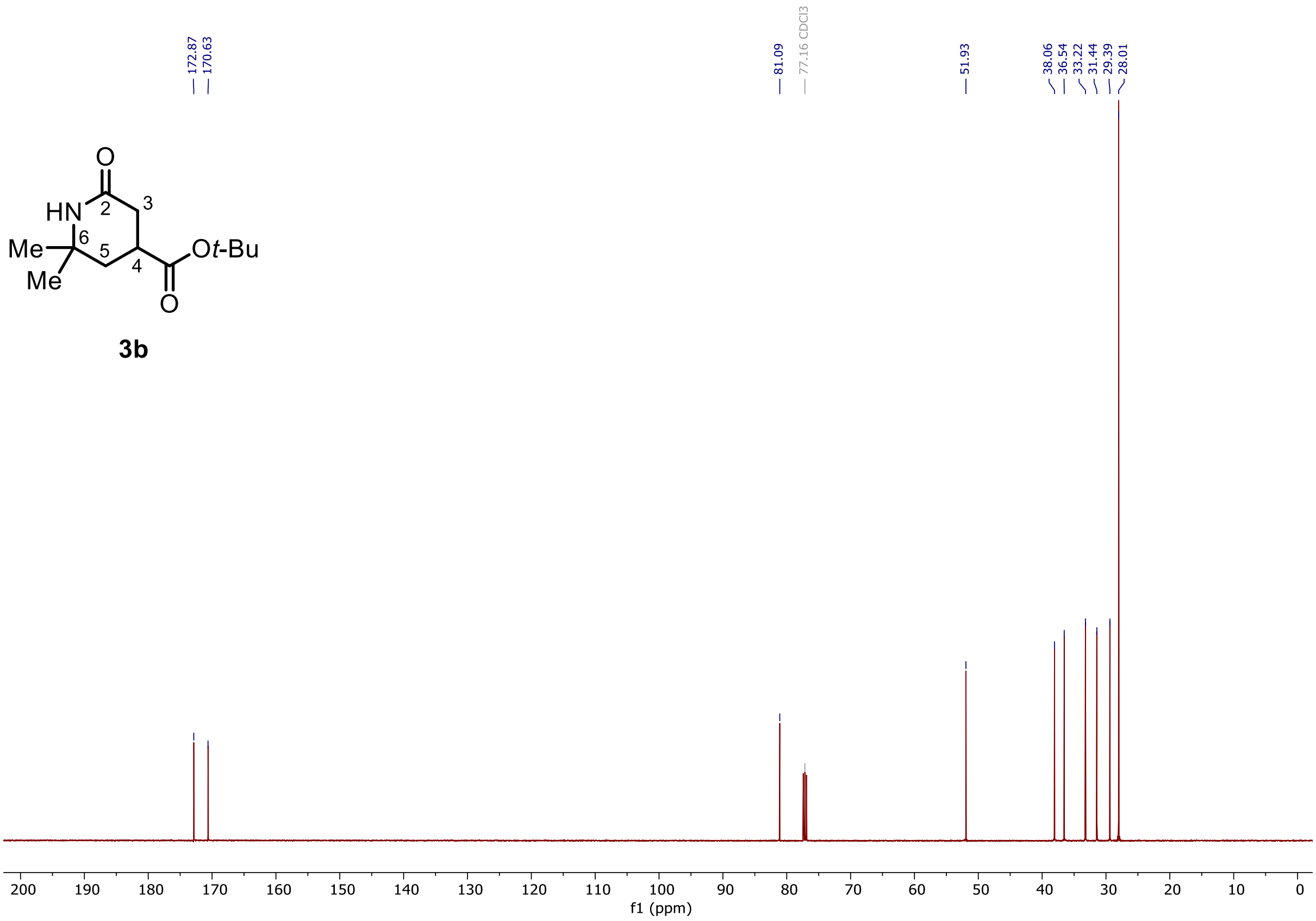

**COSY (500 MHz, $CDCl_3$)**

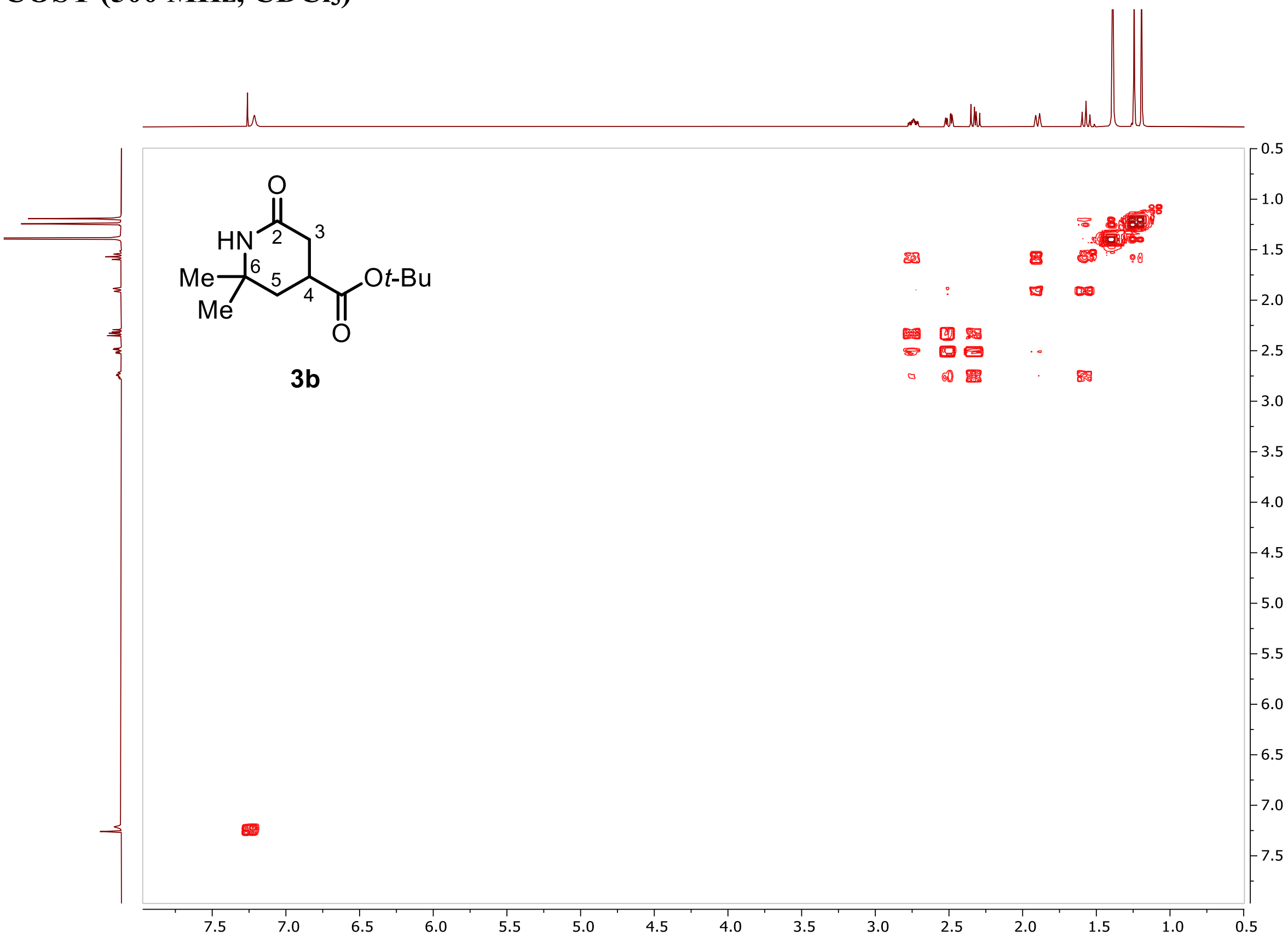


**HSQC ($^{1}H$ 500 MHz/$^{13}C$ 126 MHz, $CDCl_3$)**

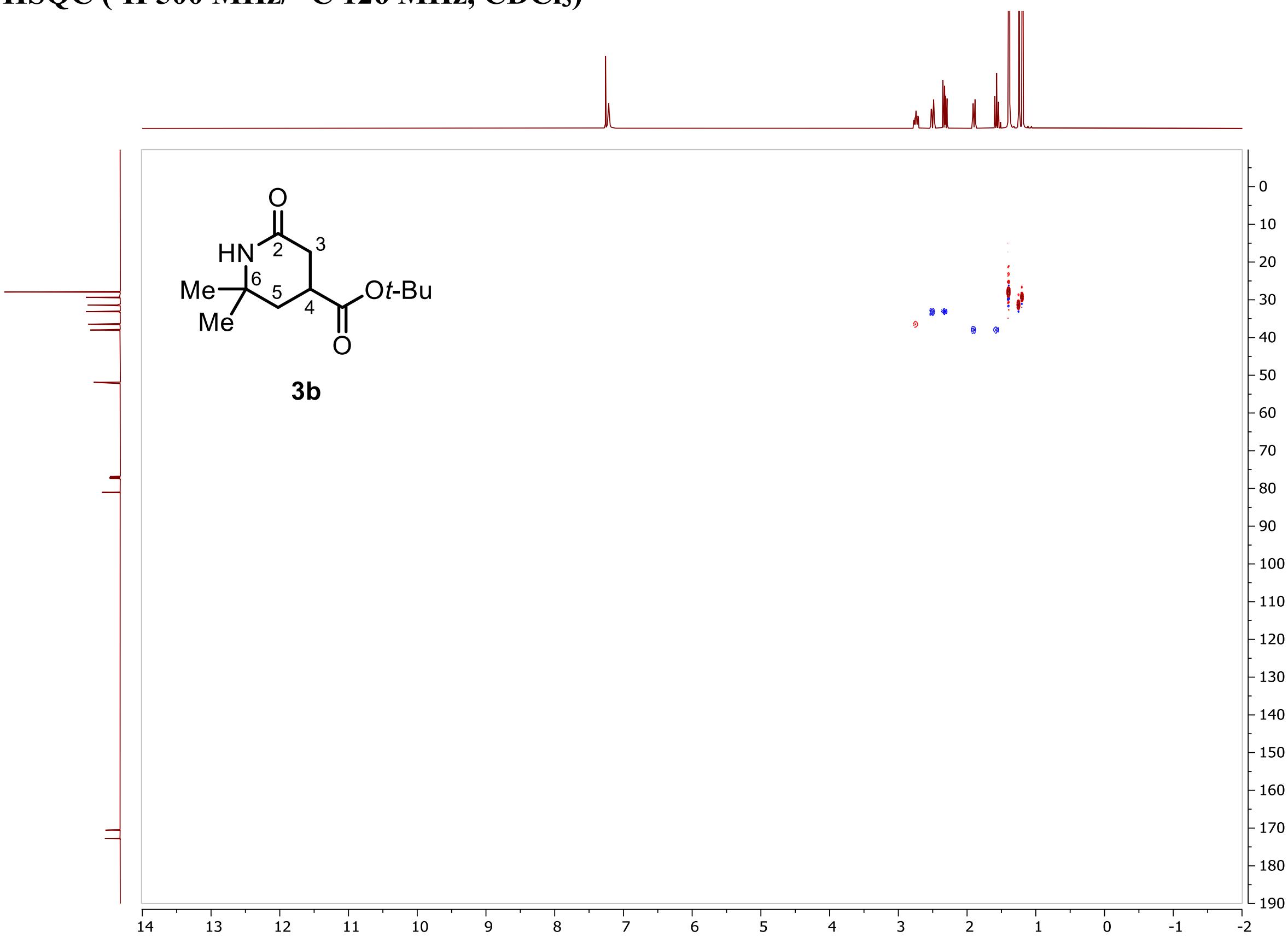

**$^{1}$H NMR (500 MHz, CDCl$_3$)**

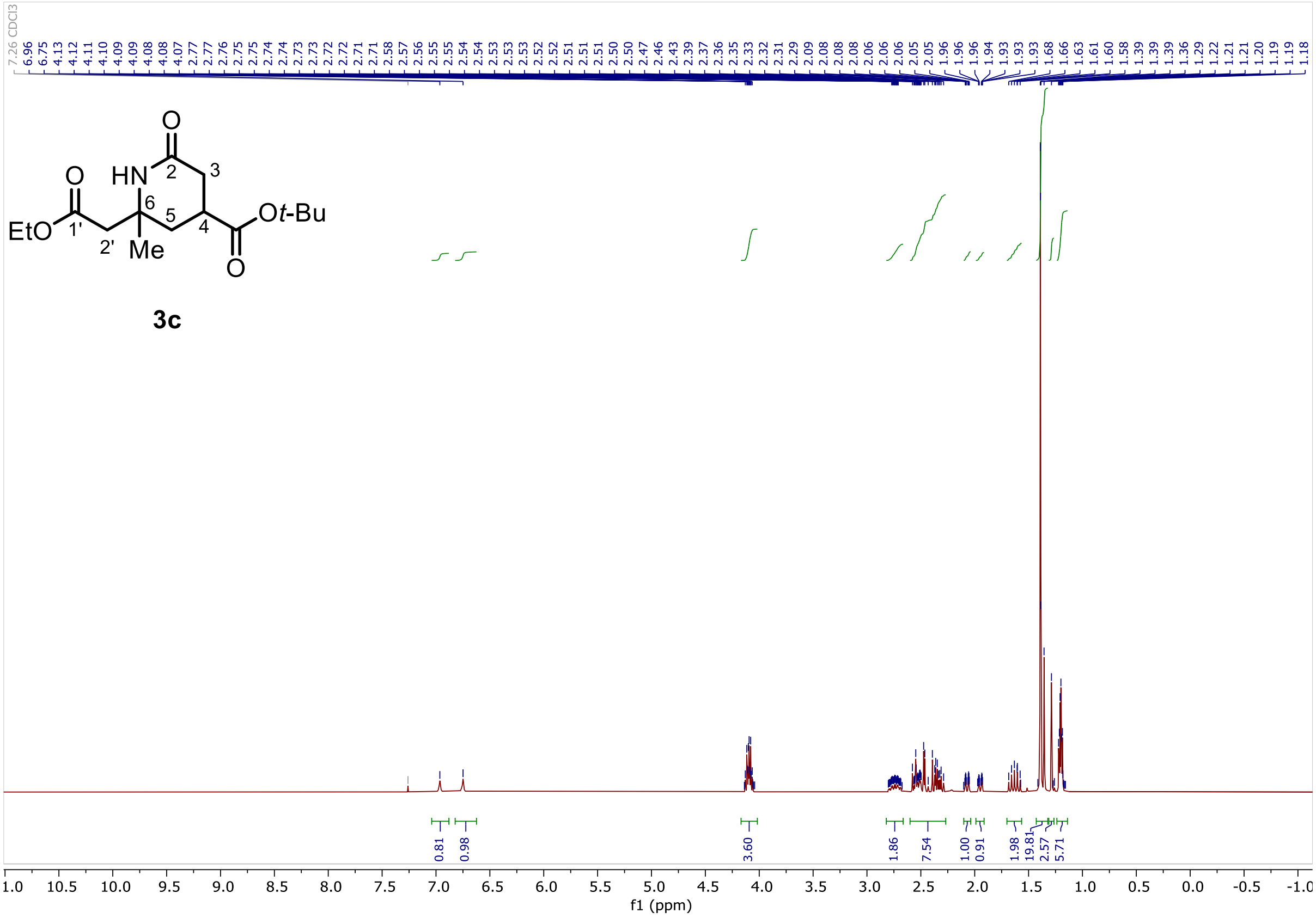


**$^{13}$C NMR (126 MHz, CDCl$_3$)**

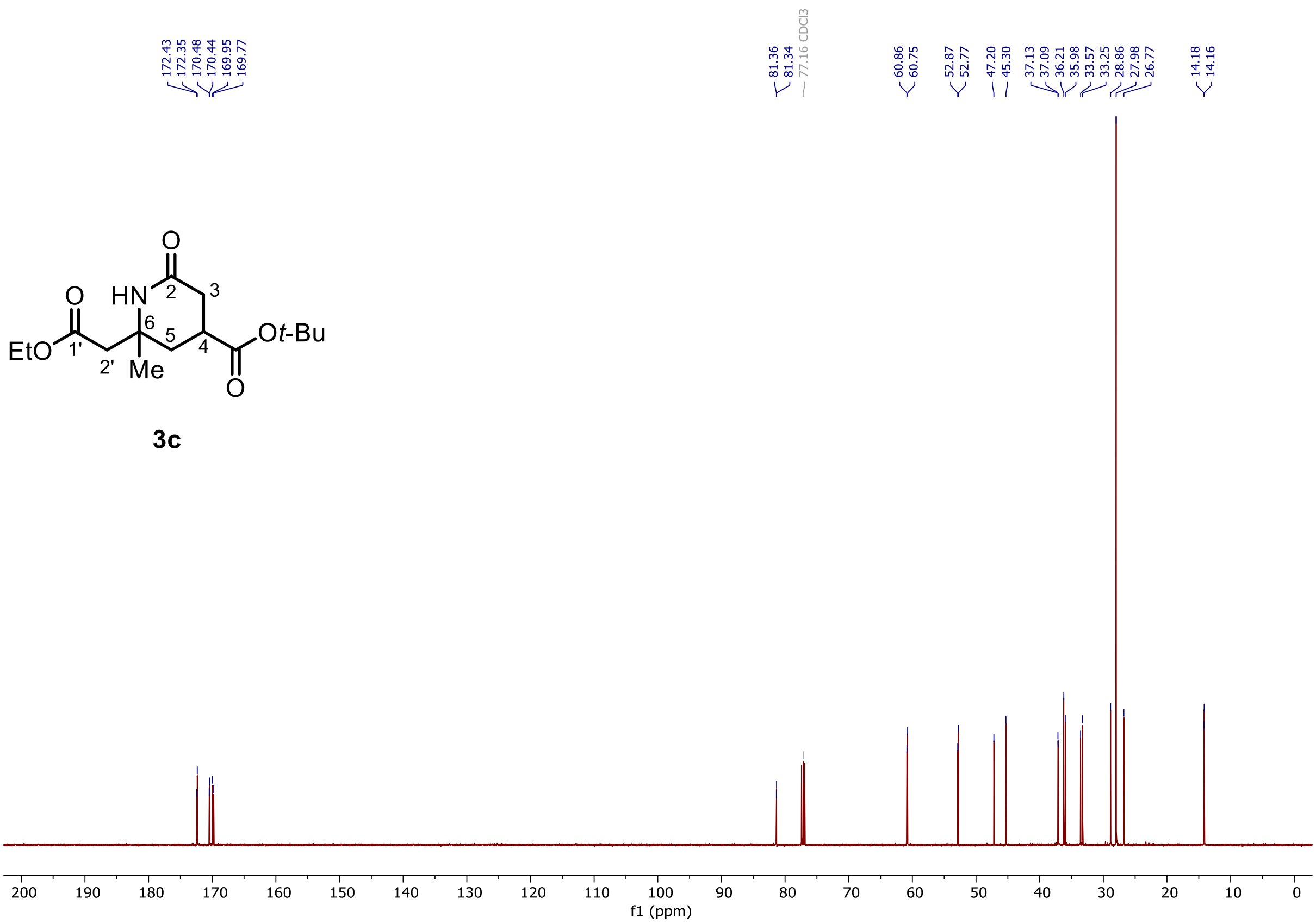

**COSY (500 MHz, CDCl$_3$)**

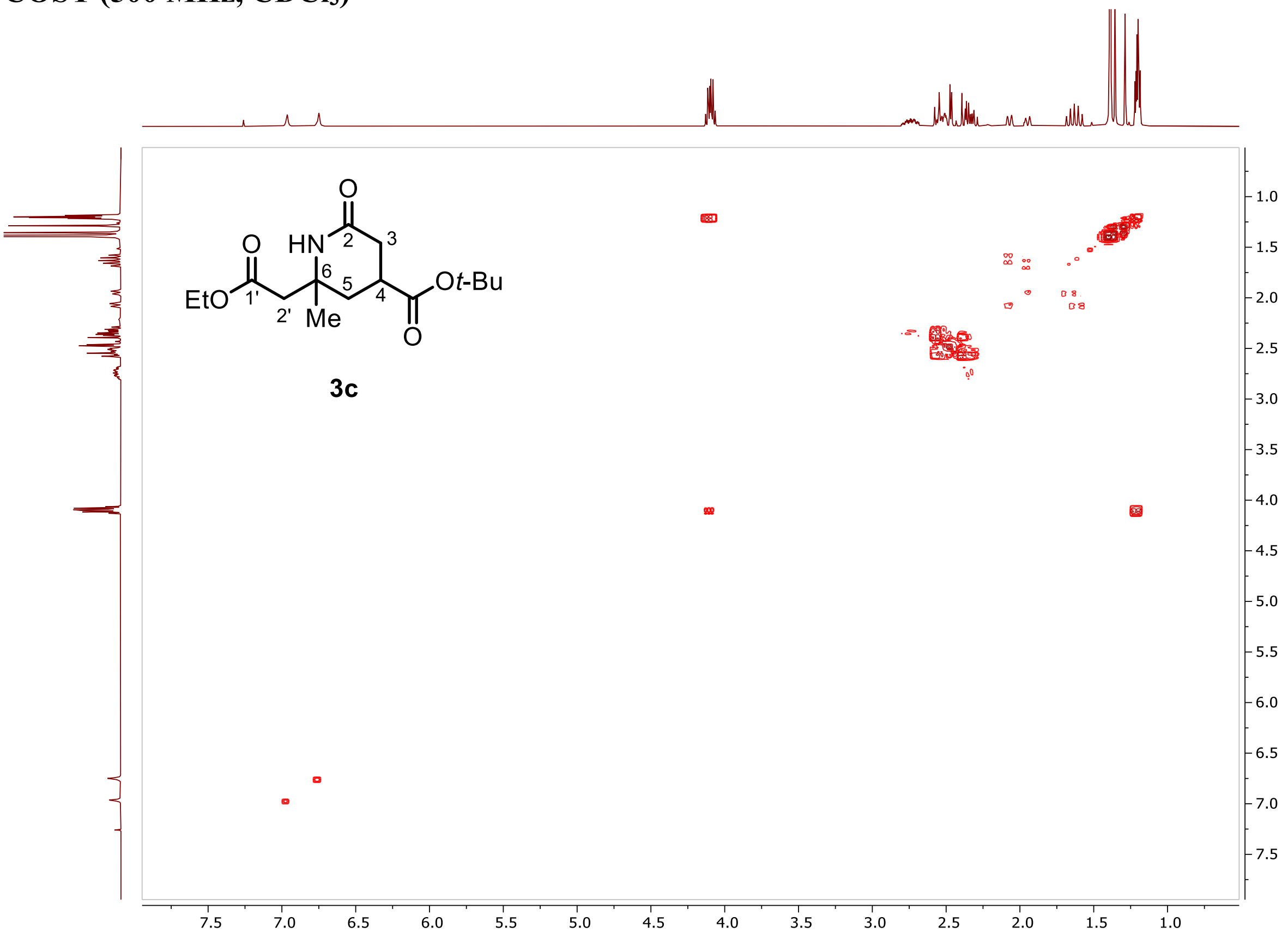

**HSQC ($^1$H 500 MHz/$^{13}$C 126 MHz, CDCl$_3$)**

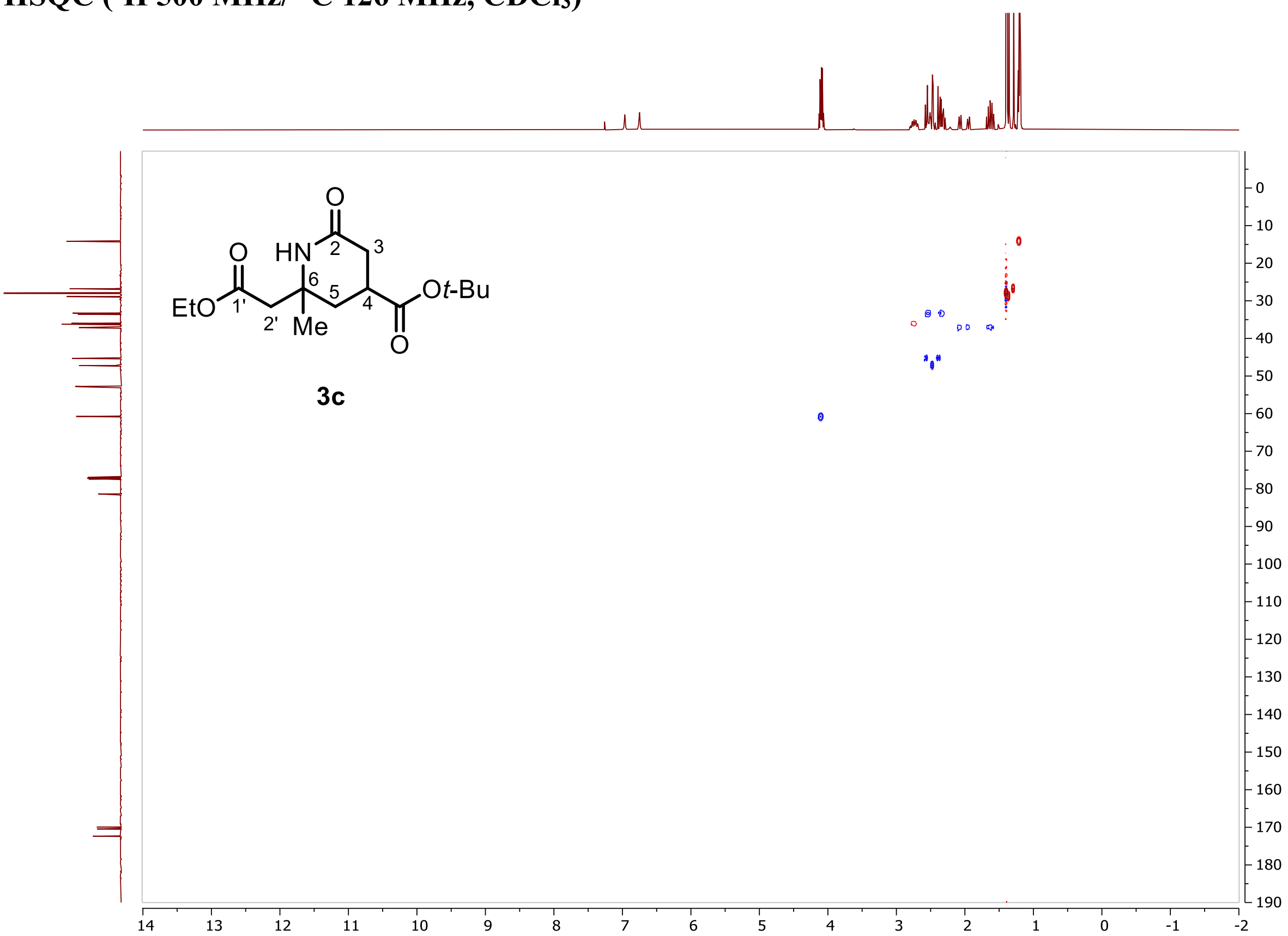

## $^{1}$H NMR (500 MHz, $CDCl_3$)

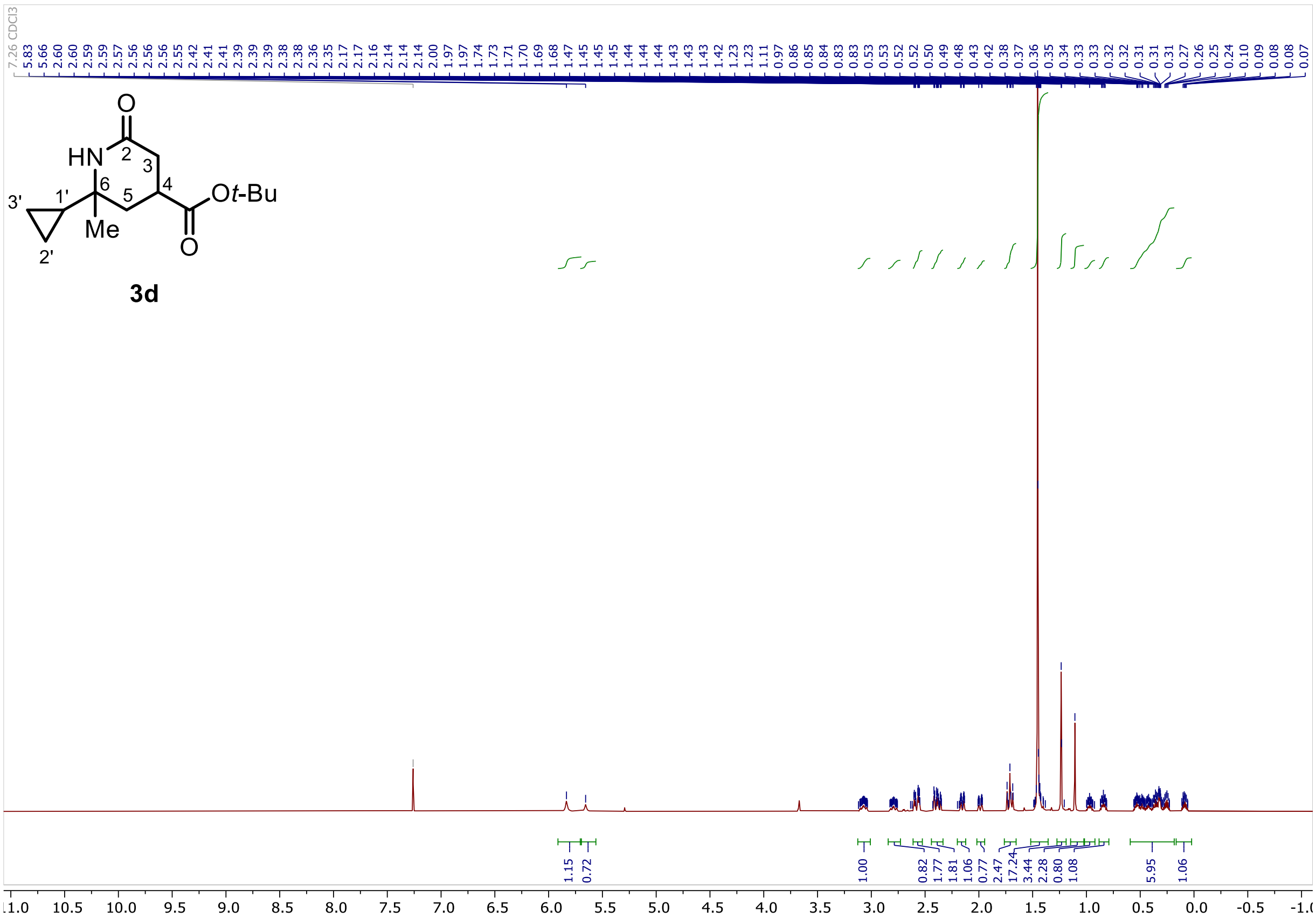


## $^{13}$C NMR (126 MHz, $CDCl_3$)

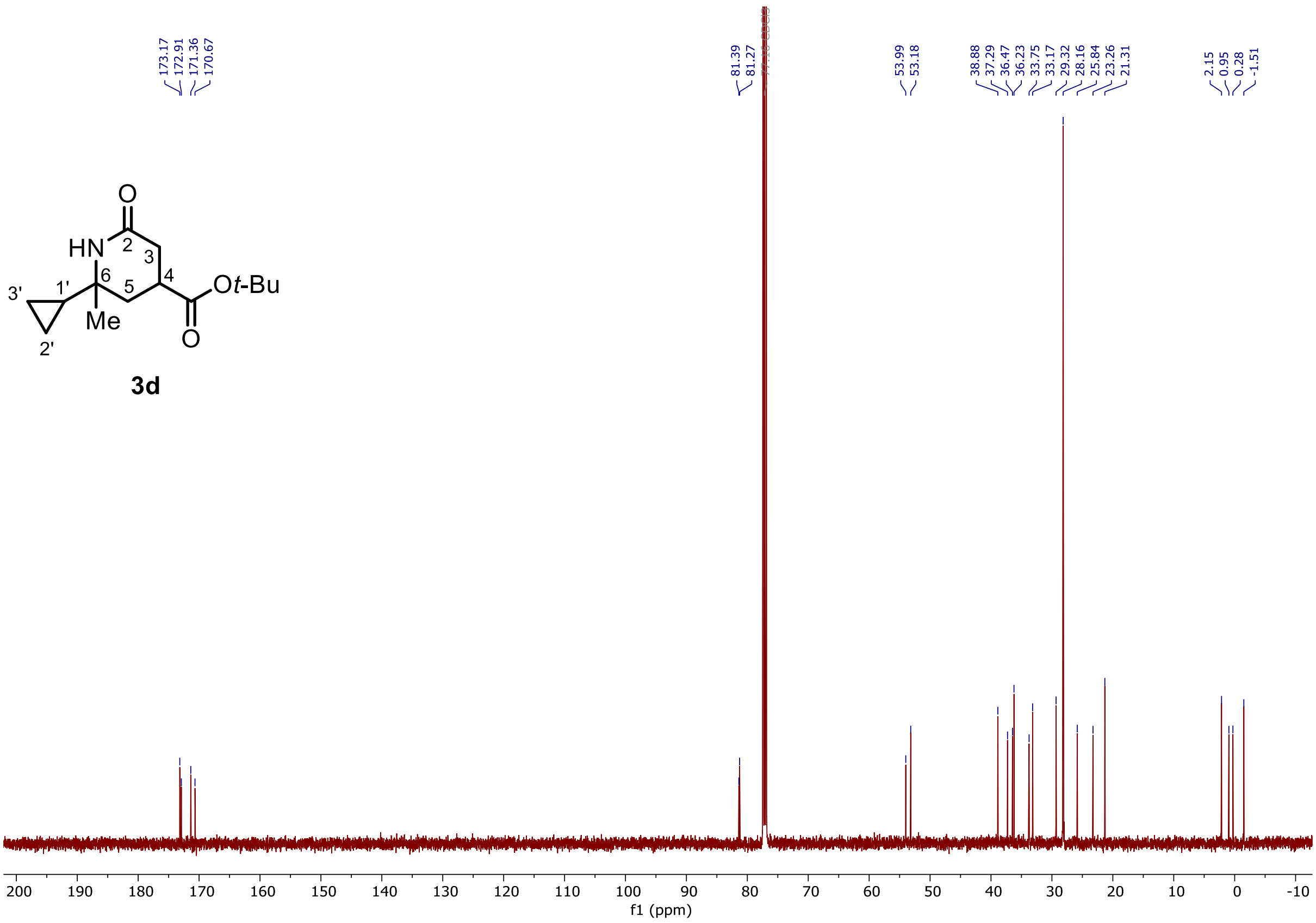

**COSY (500 MHz, CDCl$_3$)**

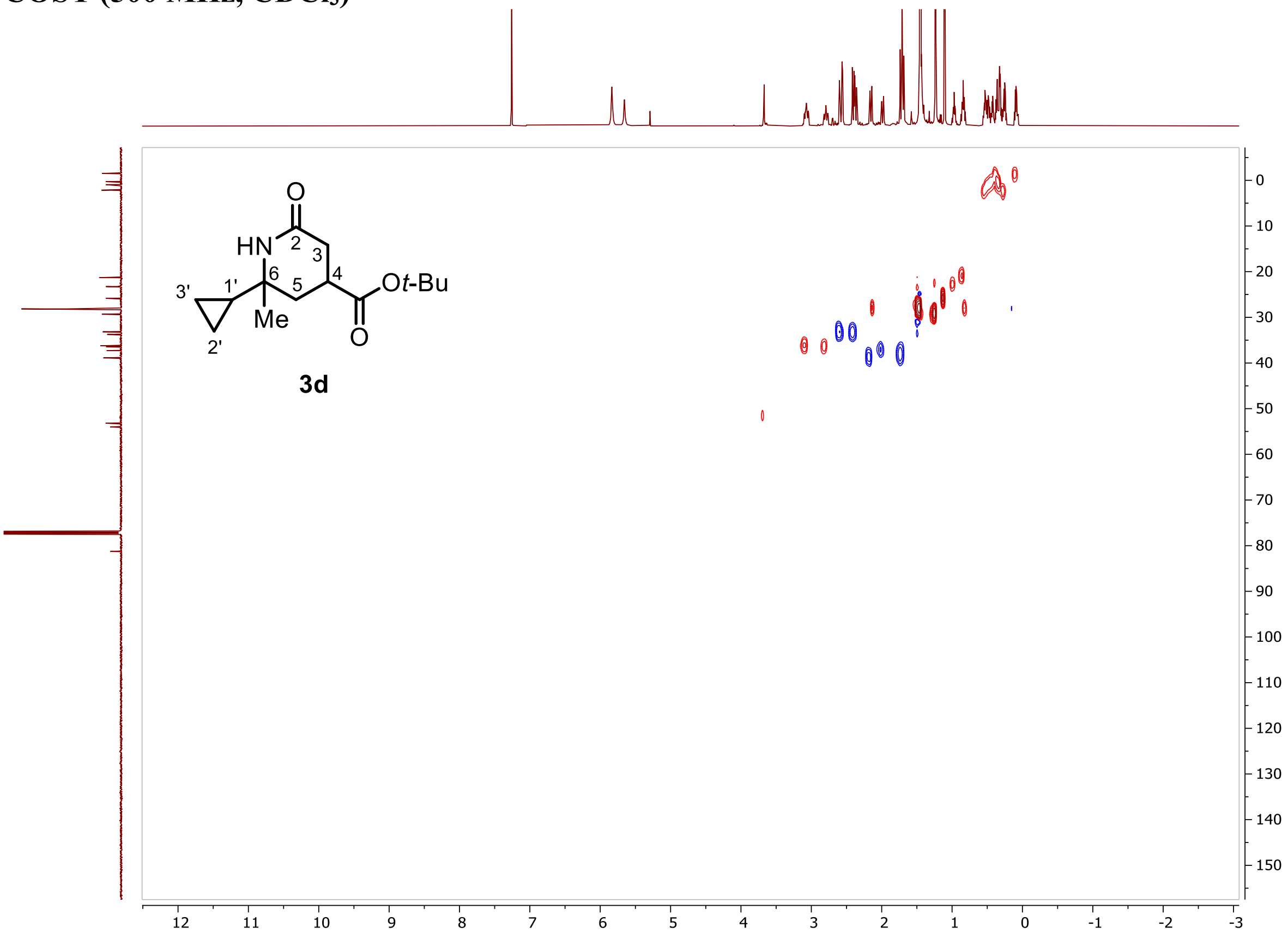


**HSQC ($^1$H 500 MHz/$^{13}$C 126 MHz, CDCl$_3$)**

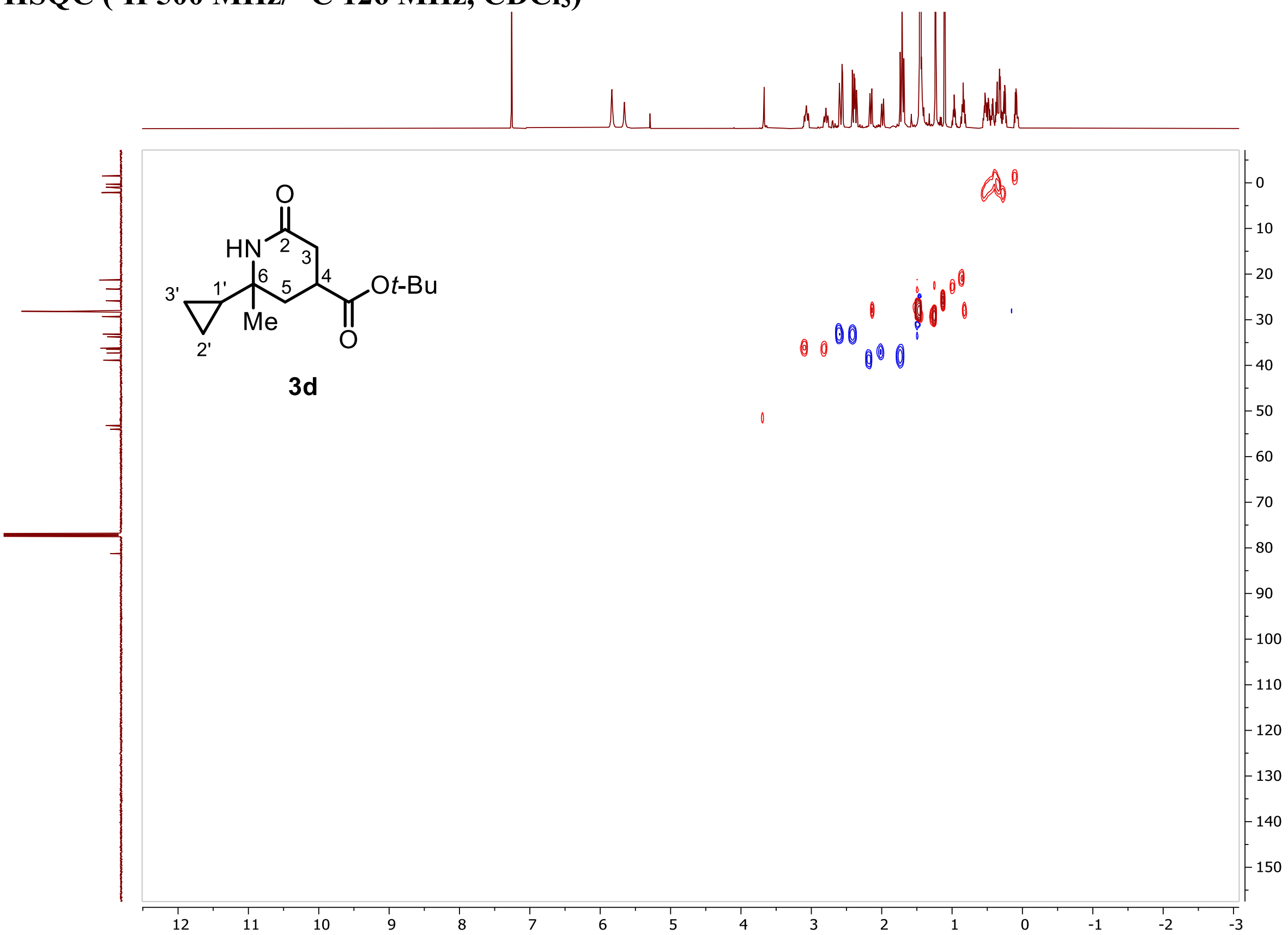

## $^{1}$H NMR (500 MHz, CDCl$_3$)

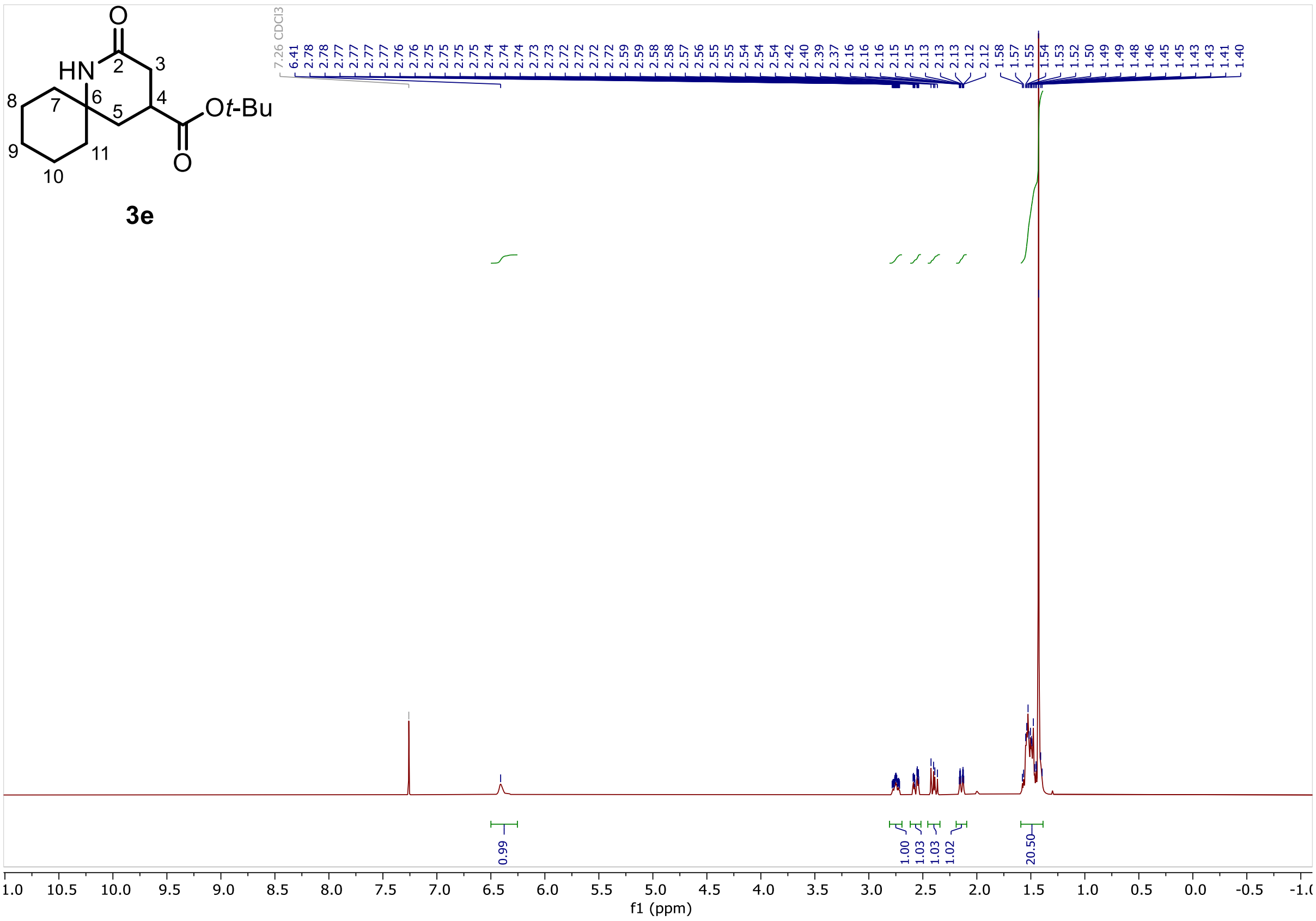


## $^{13}$C NMR (126 MHz, CDCl$_3$)

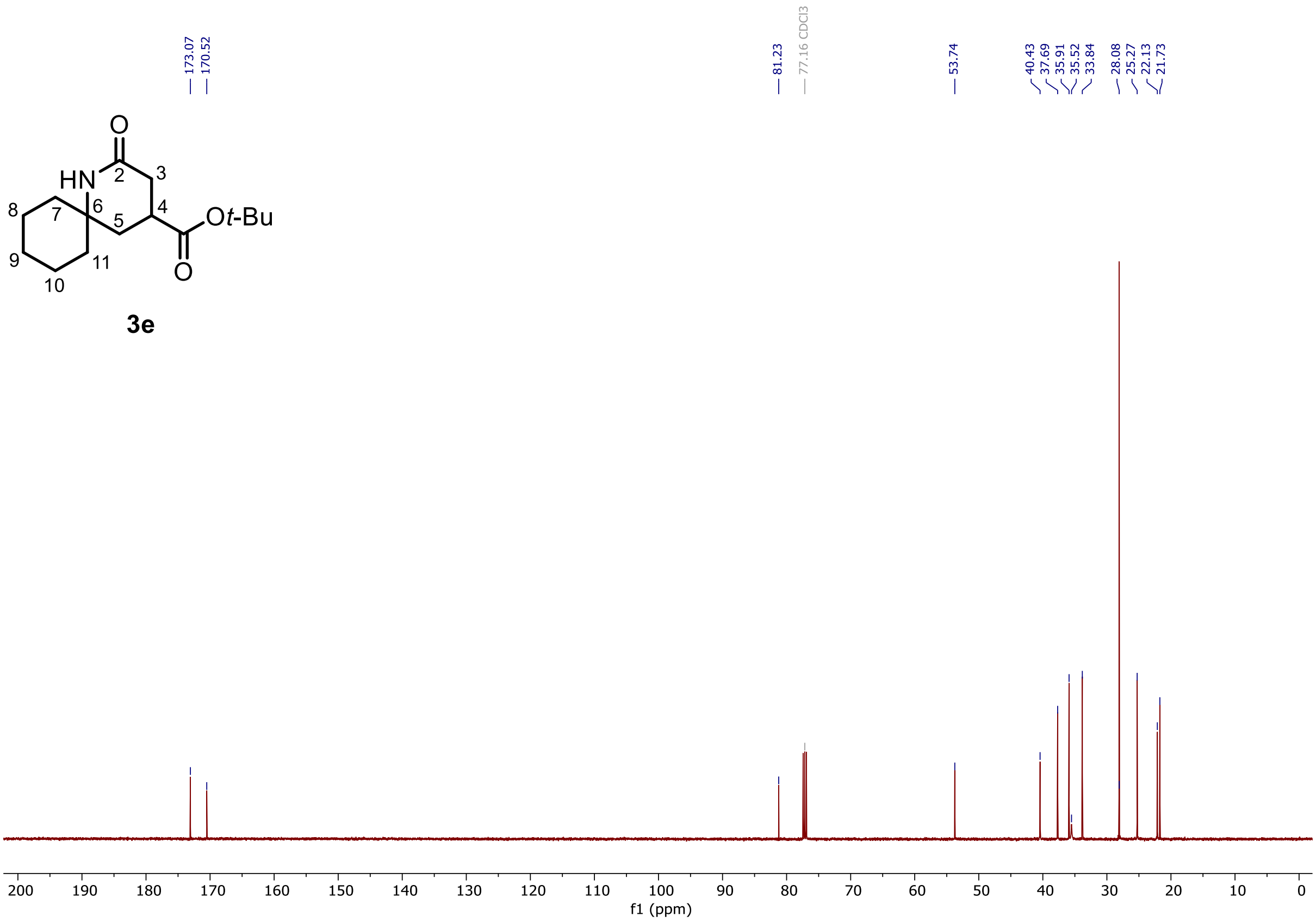

**COSY (500 MHz, $CDCl_3$)**

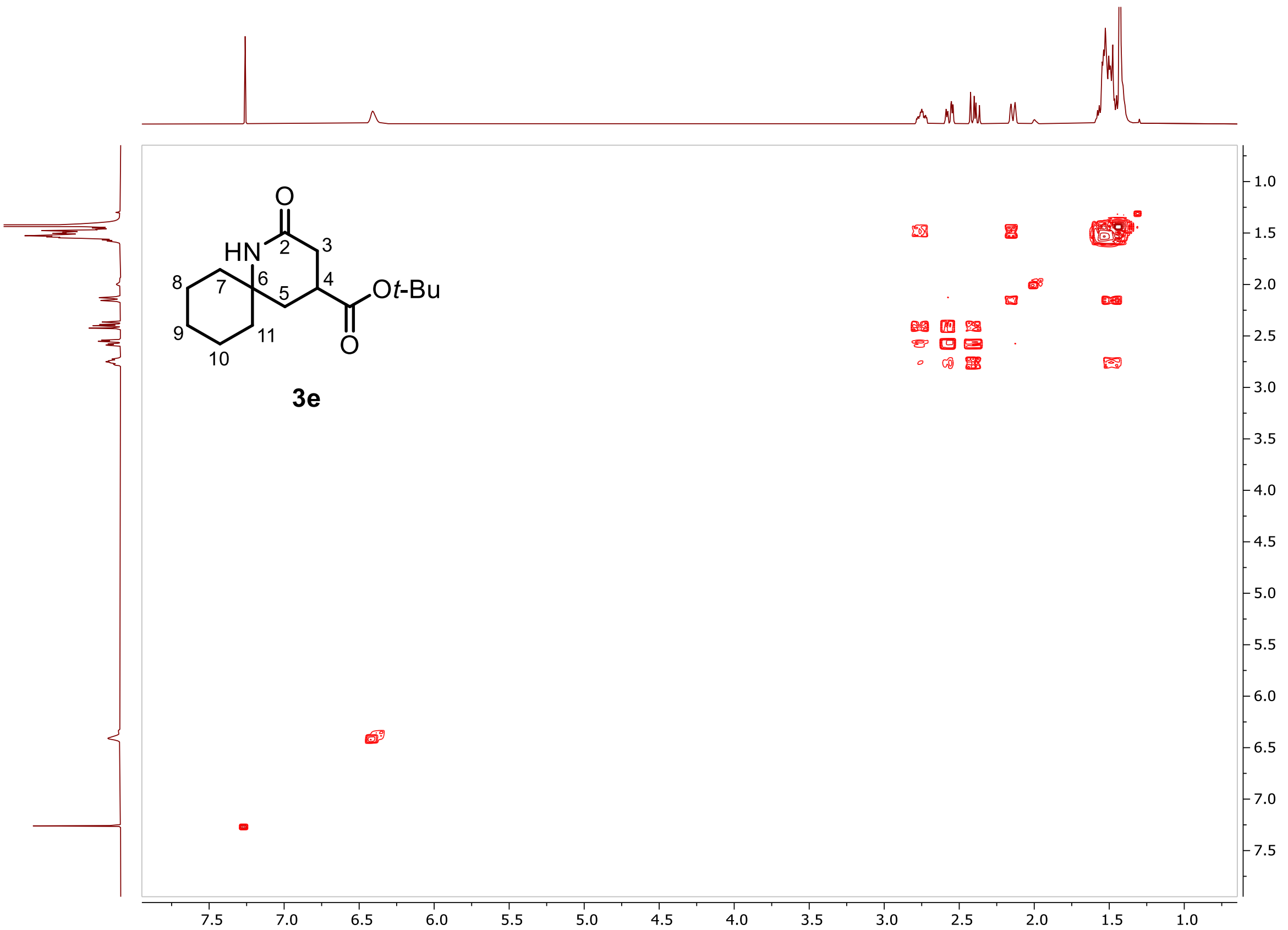


**HSQC ($^1H$ 500 MHz/$^{13}C$ 126 MHz, $CDCl_3$)**

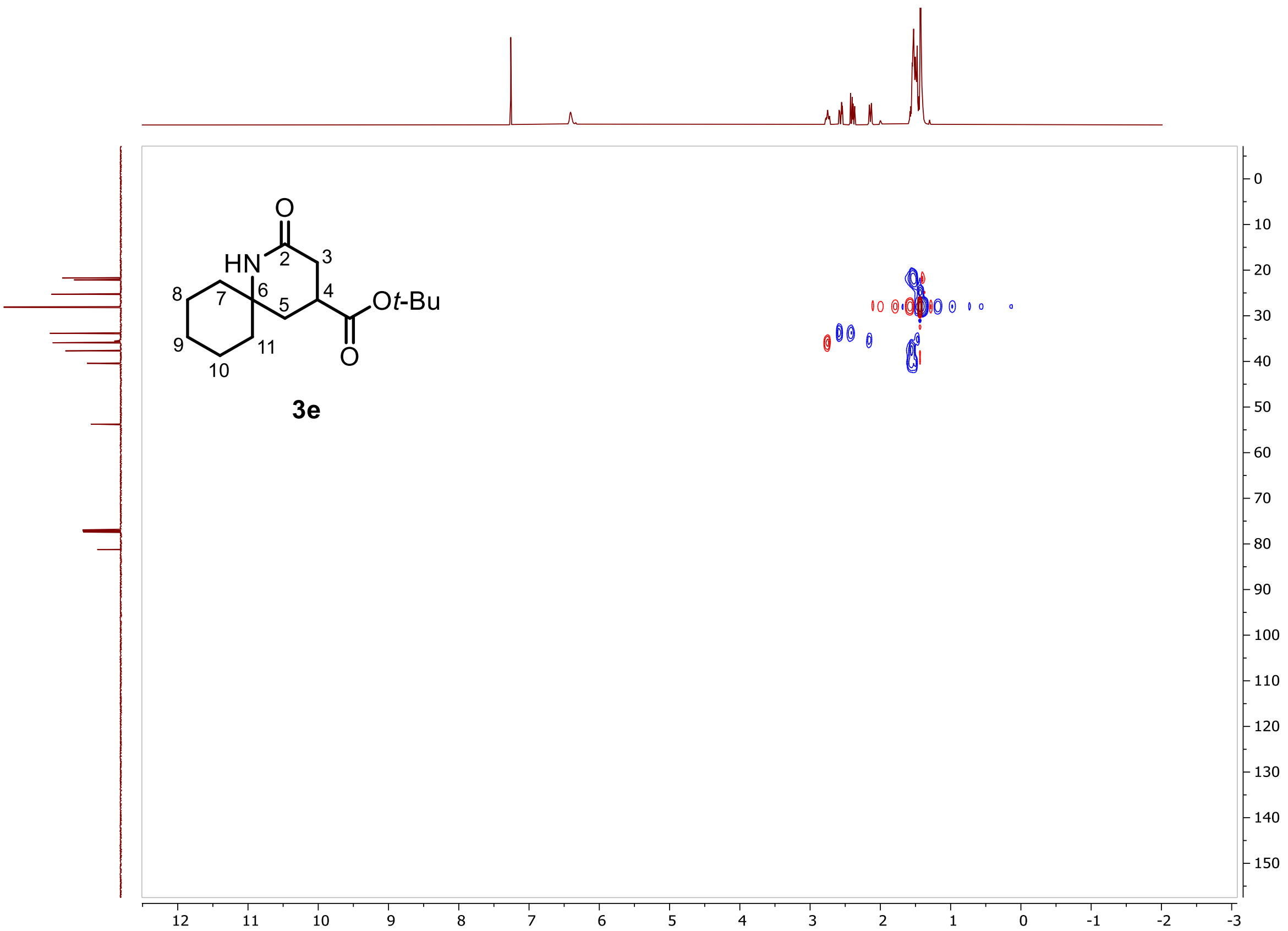

**$^{1}$H NMR (500 MHz, $CDCl_3$)**

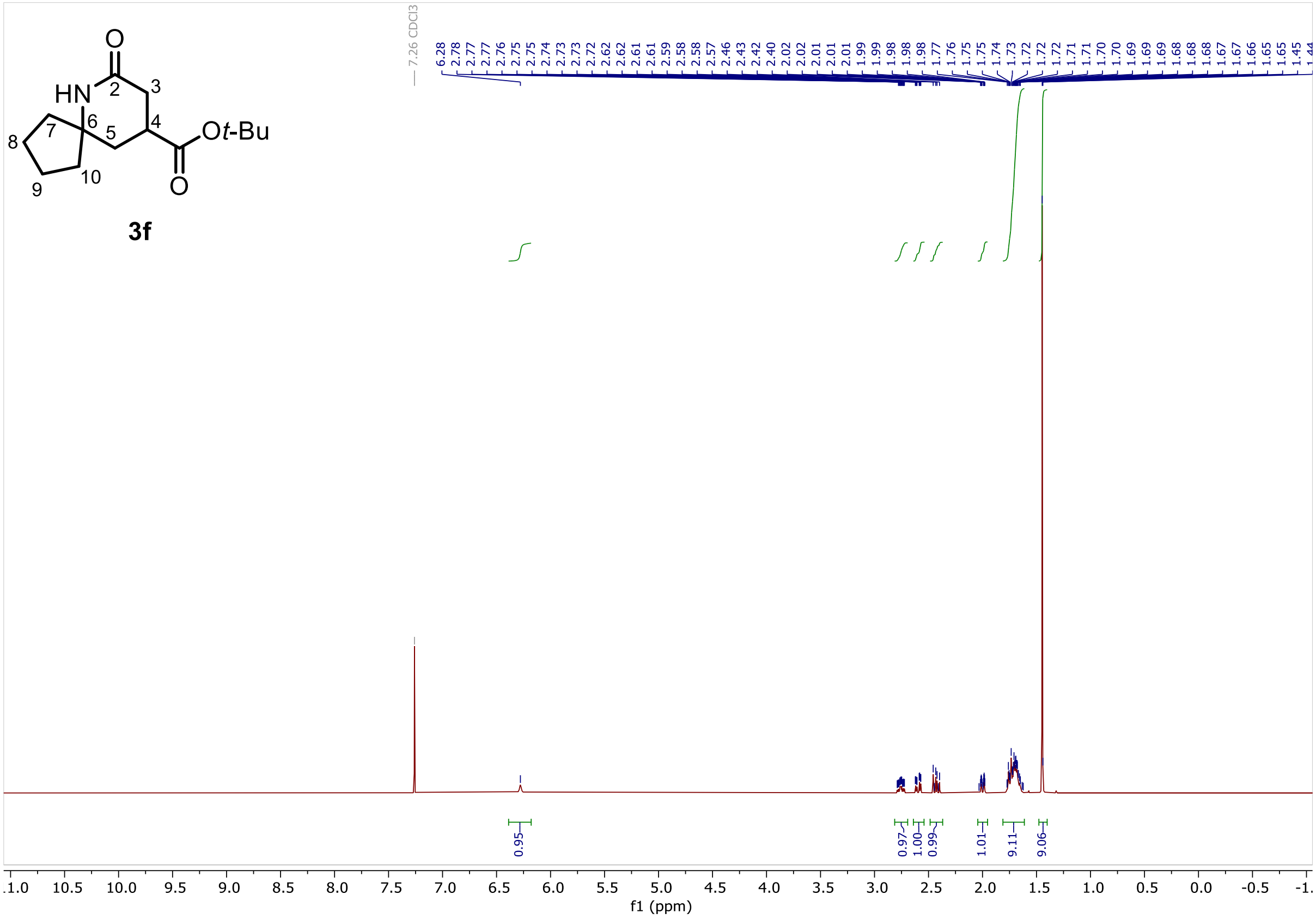

**$^{13}$C NMR (126 MHz, $CDCl_3$)**

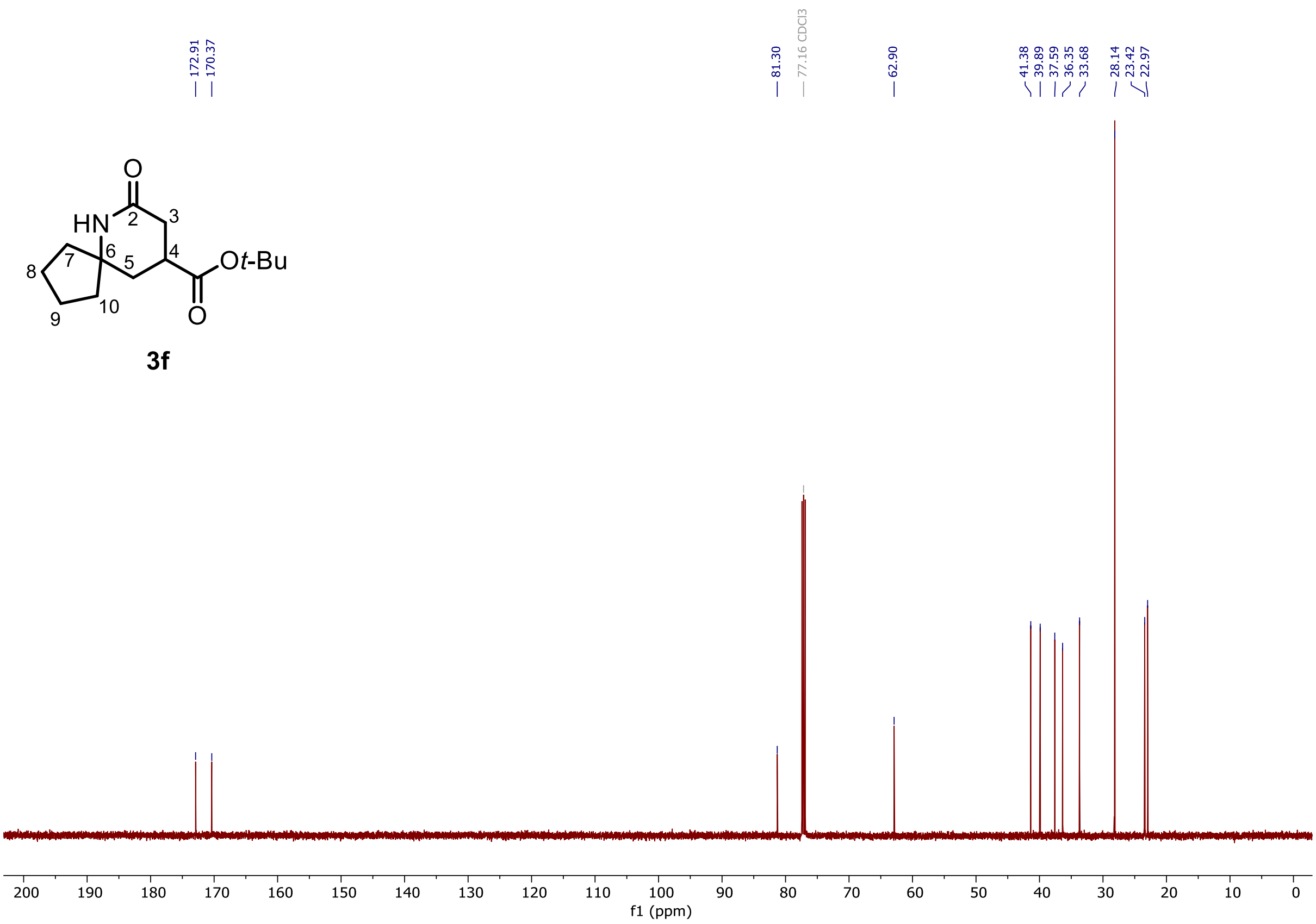

**COSY (500 MHz, $CDCl_3$)**

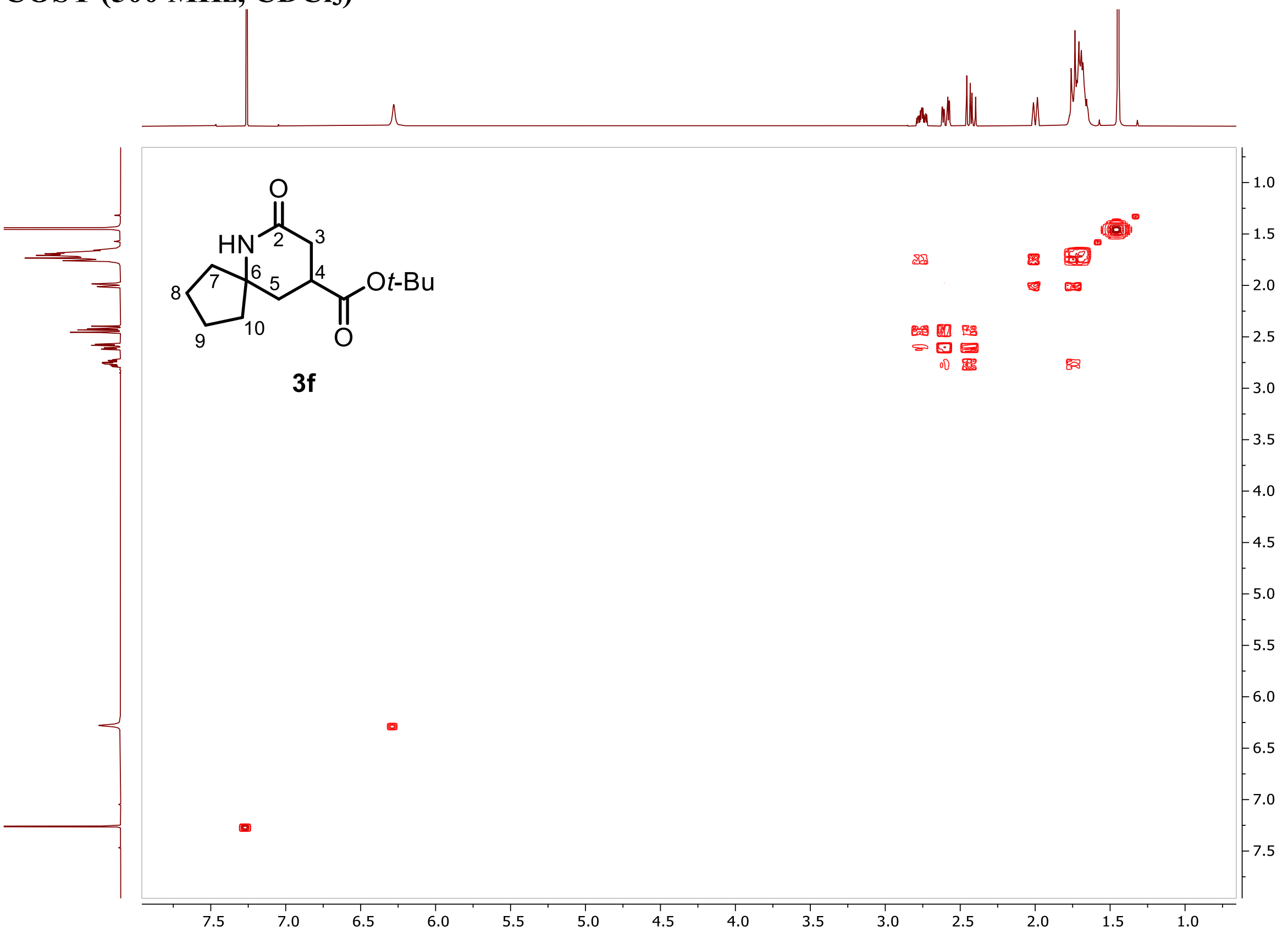


**HSQC ($^1H$ 500 MHz/$^{13}C$ 126 MHz, $CDCl_3$)**

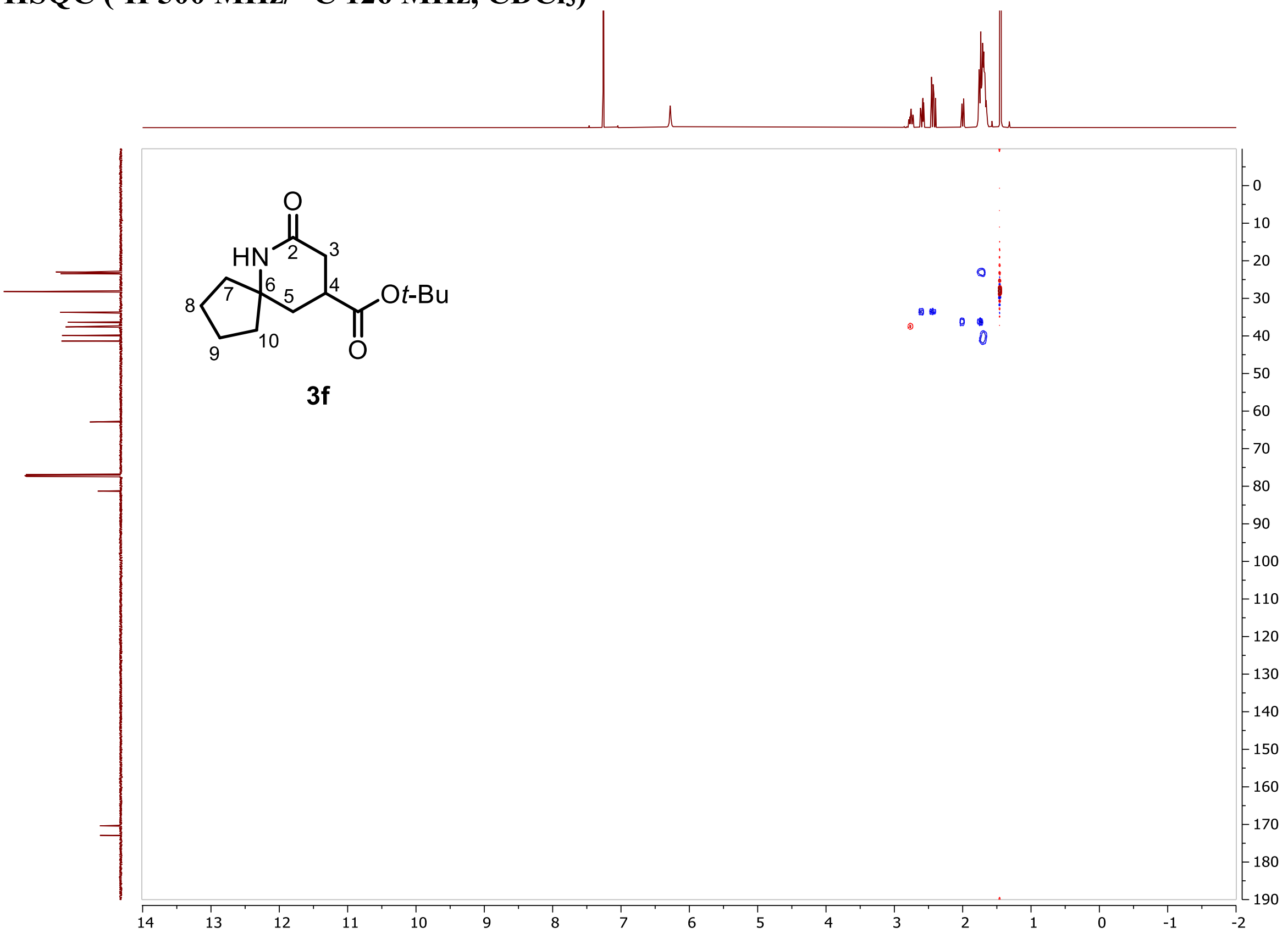

**$^{1}$H NMR (500 MHz, $CDCl_3$)**

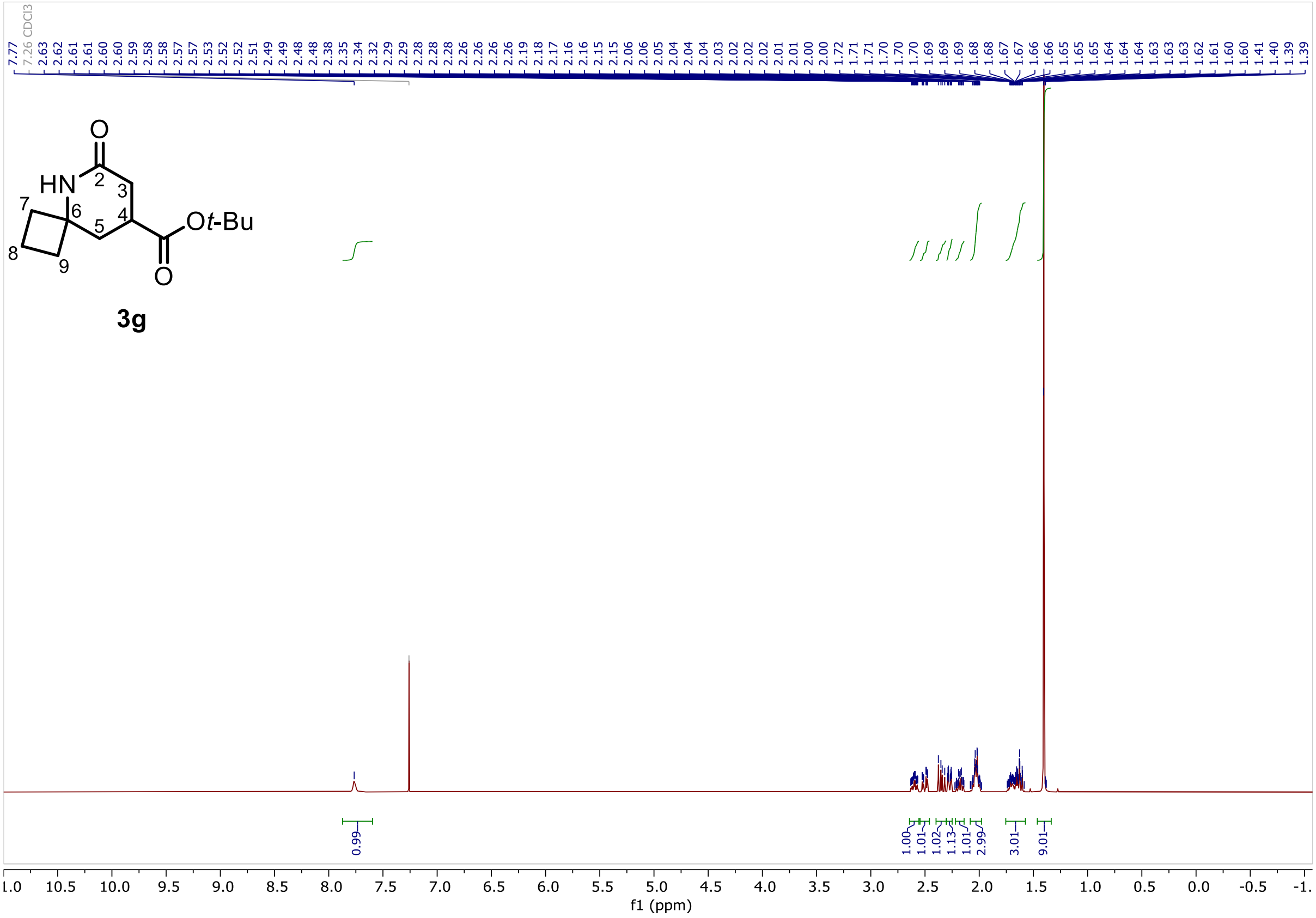


**$^{13}$C NMR (126 MHz, $CDCl_3$)**

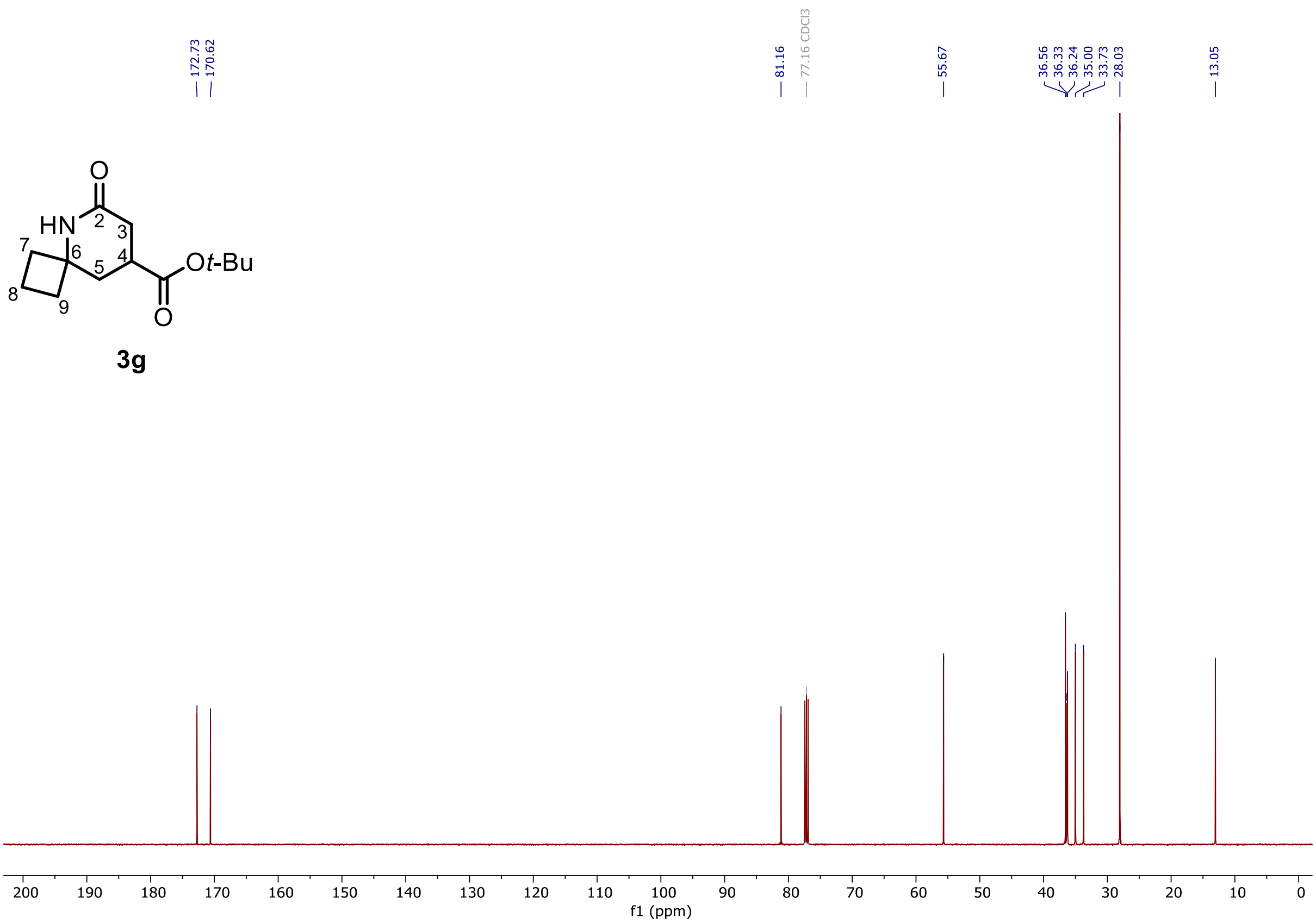

**COSY (500 MHz, CDCl$_3$)**

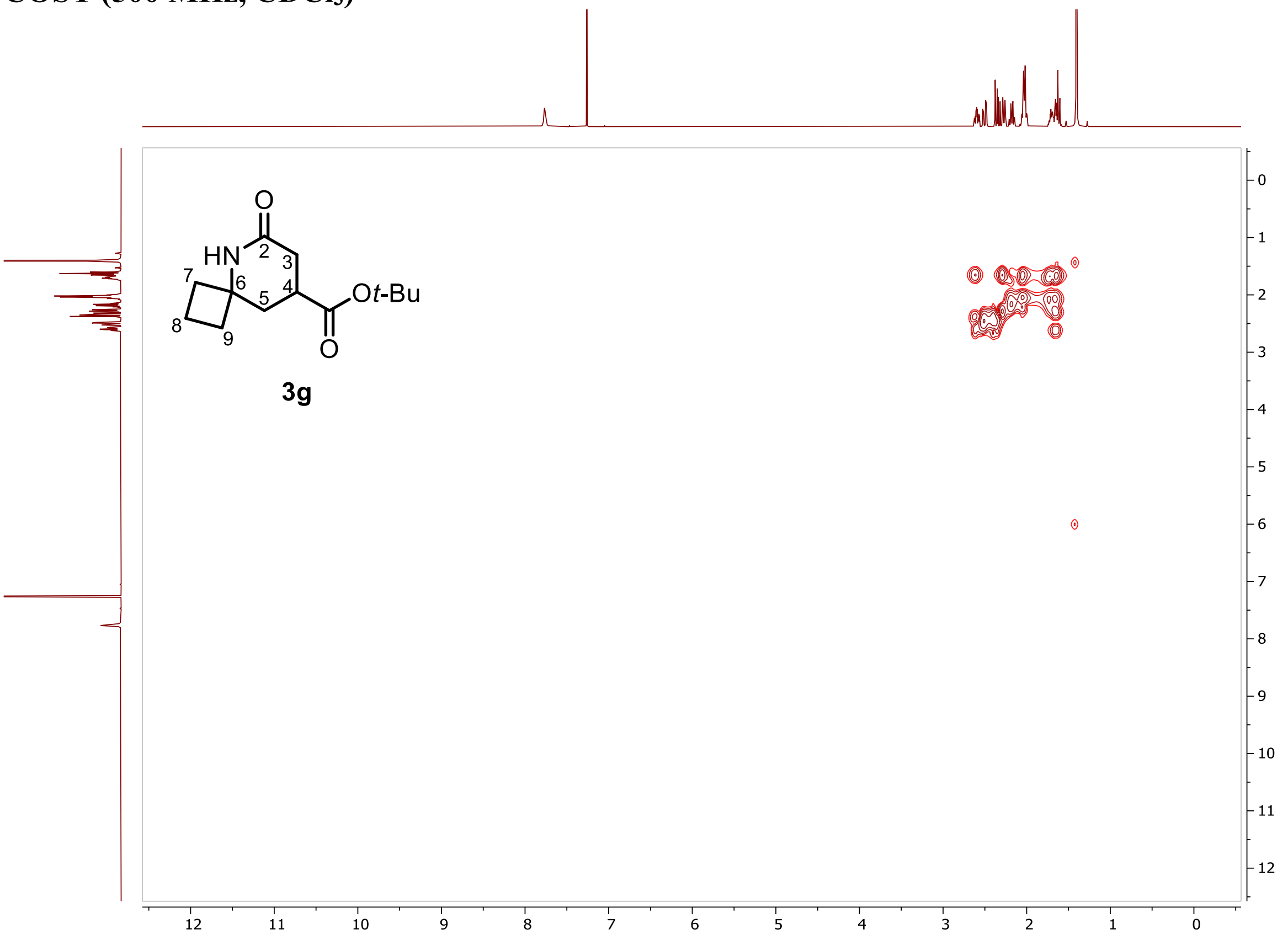


**HSQC ($^1$H 500 MHz/$^{13}$C 126 MHz, CDCl$_3$)**

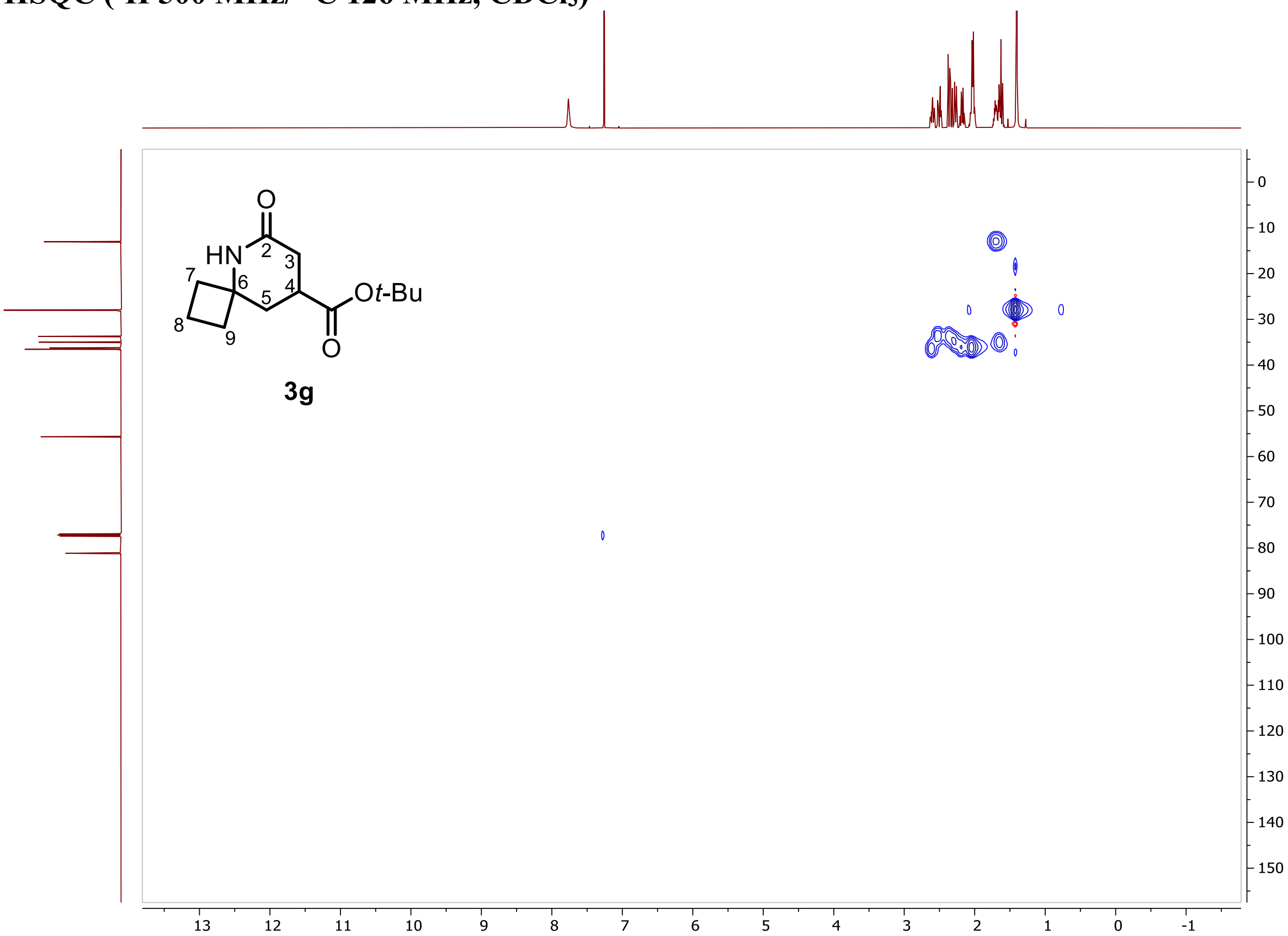

**$^{1}$H NMR (500 MHz, CDCl$_3$)**

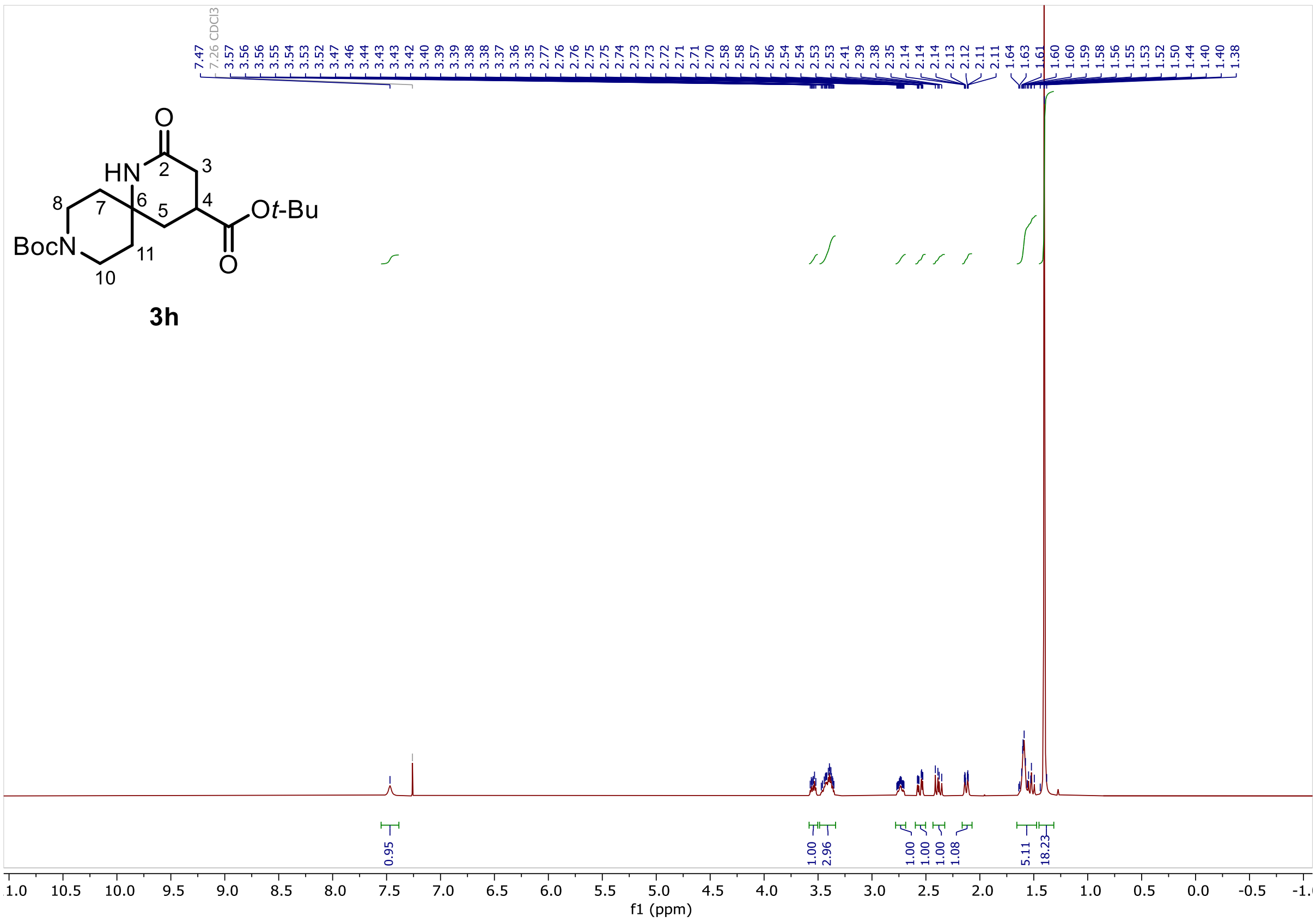


**$^{13}$C NMR (126 MHz, CDCl$_3$)**

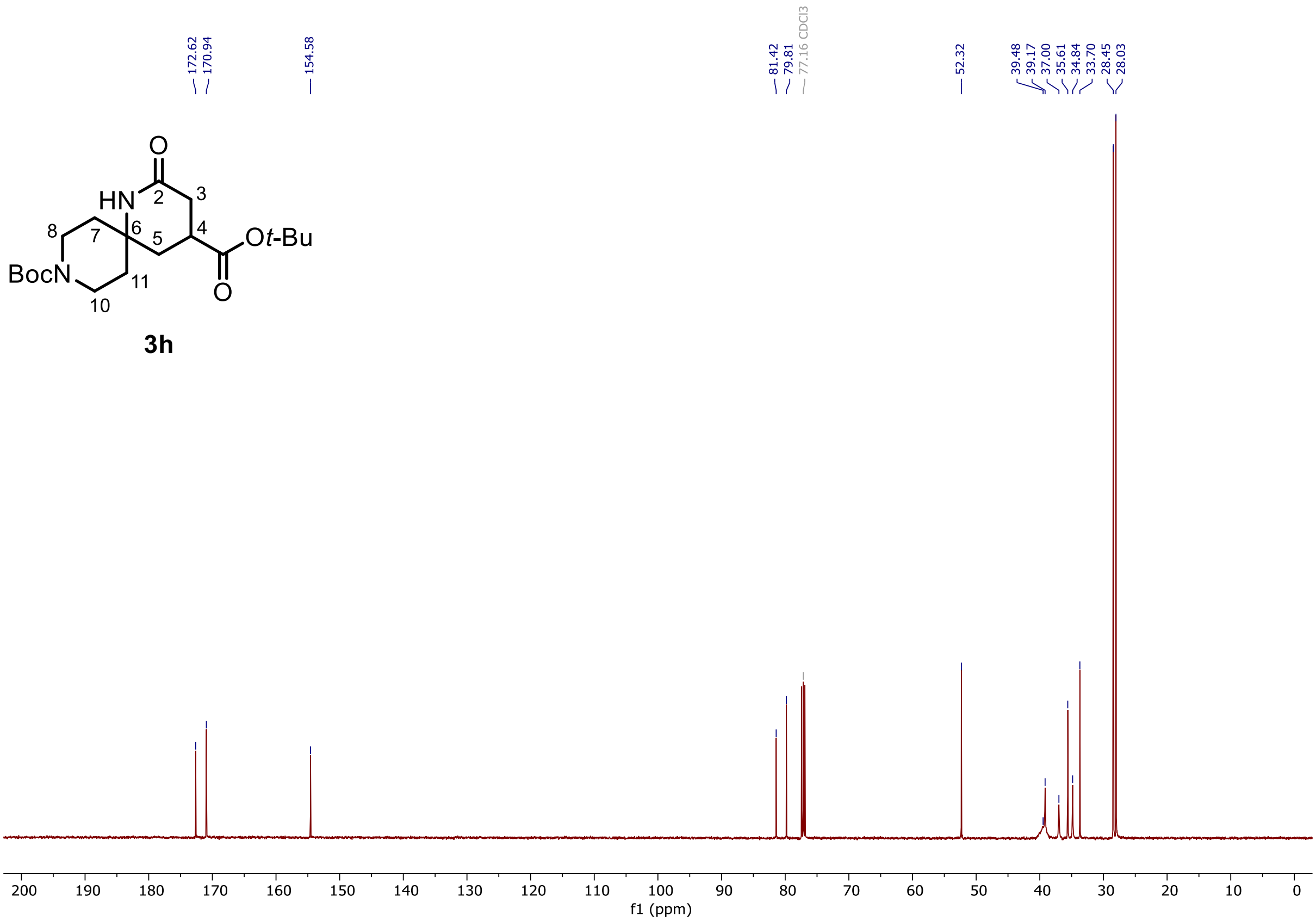

**COSY (500 MHz, CDCl$_3$)**

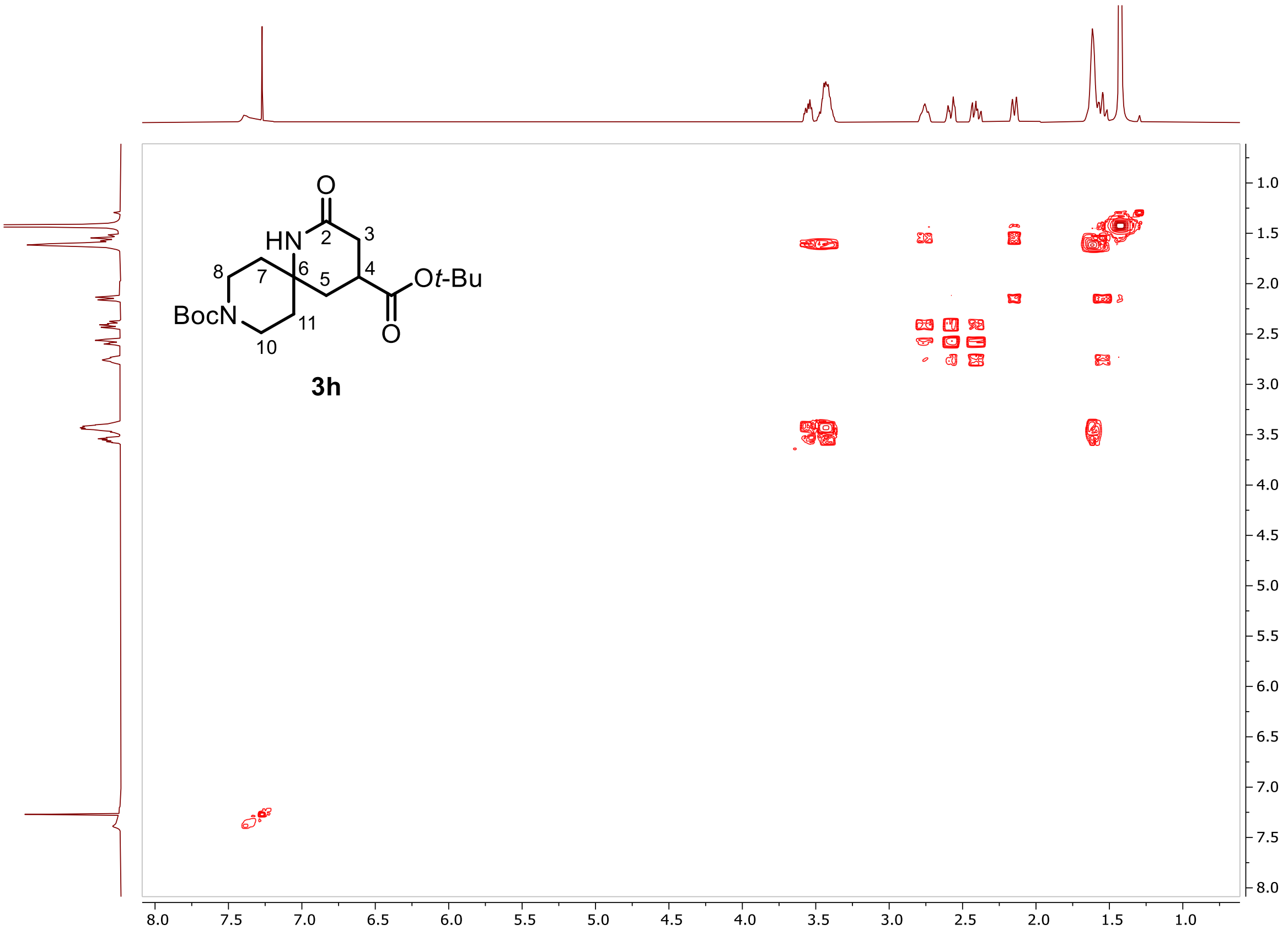


**HSQC ($^1$H 500 MHz/$^{13}$C 126 MHz, CDCl$_3$)**

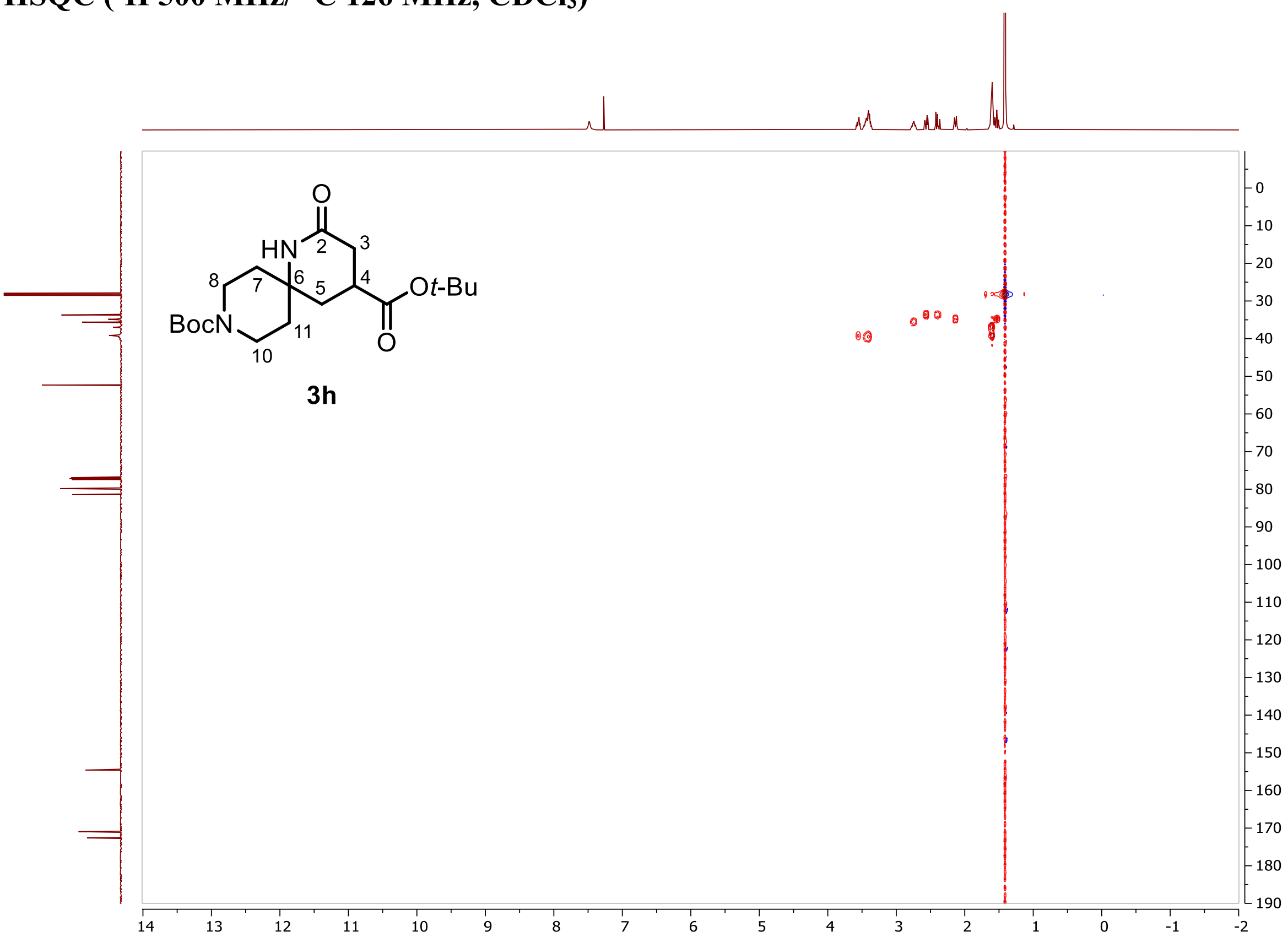

## $^{1}$H NMR (500 MHz, $CDCl_3$)

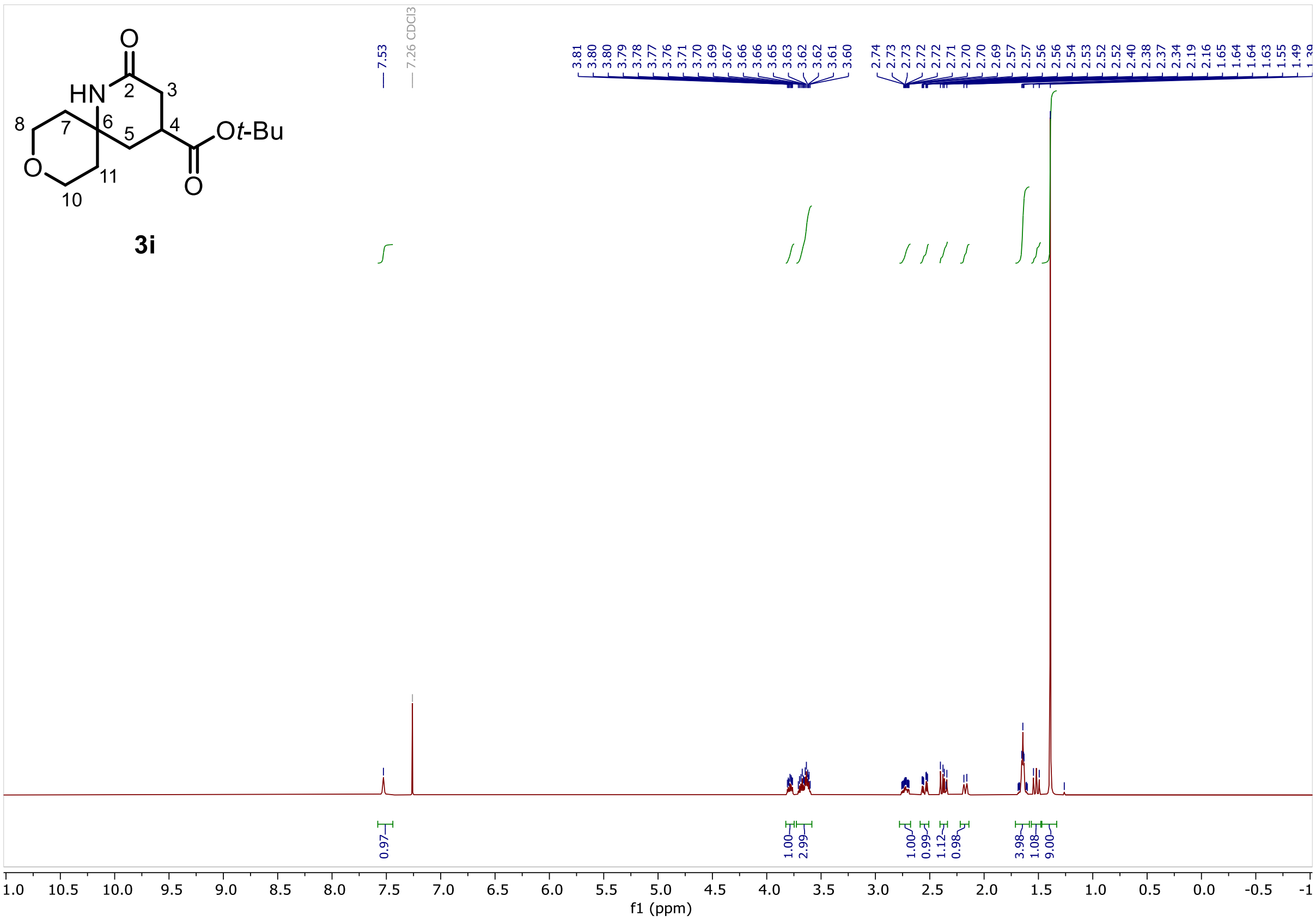


## $^{13}$C NMR (126 MHz, $CDCl_3$)

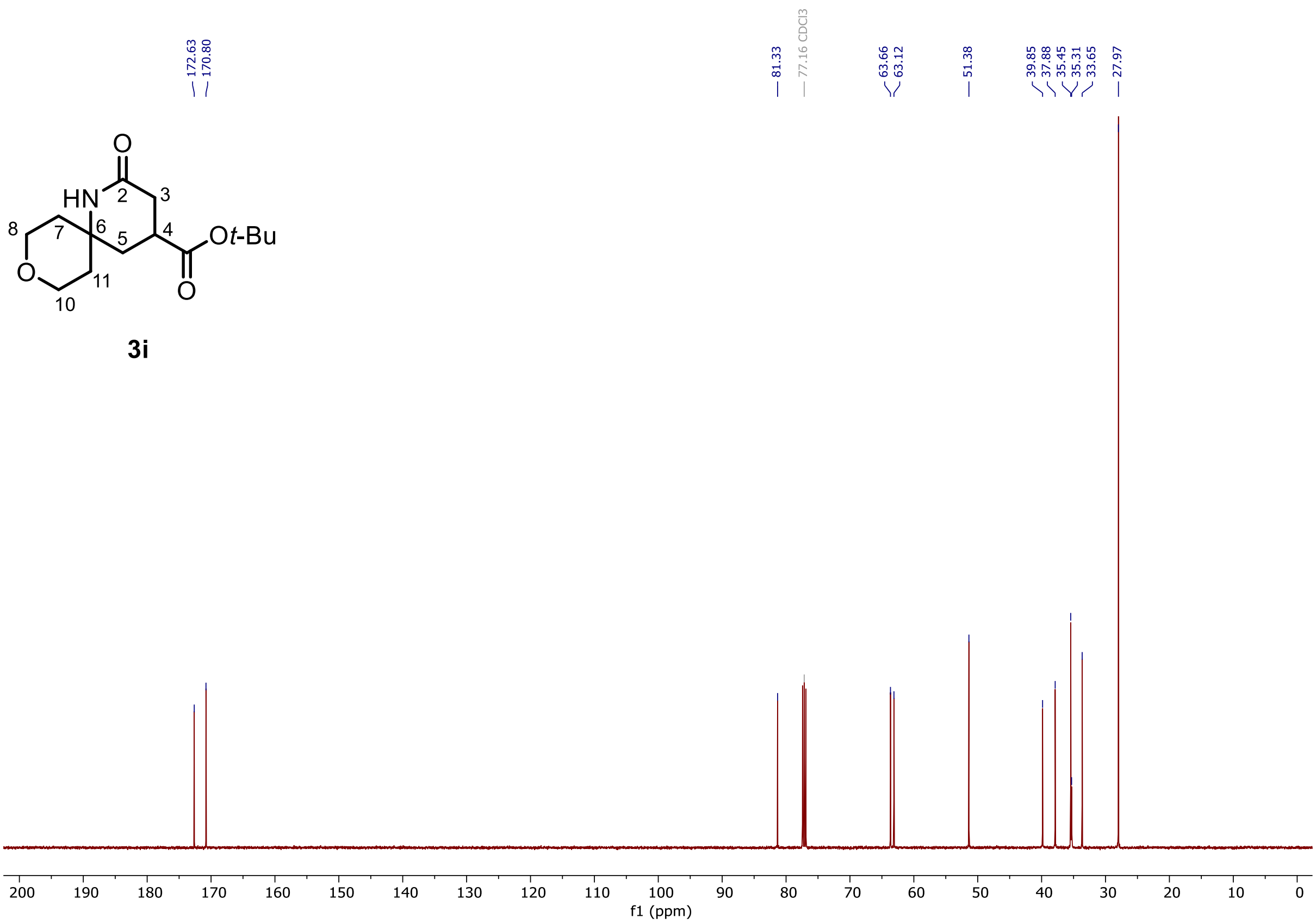

**COSY (500 MHz, CDCl$_3$)**

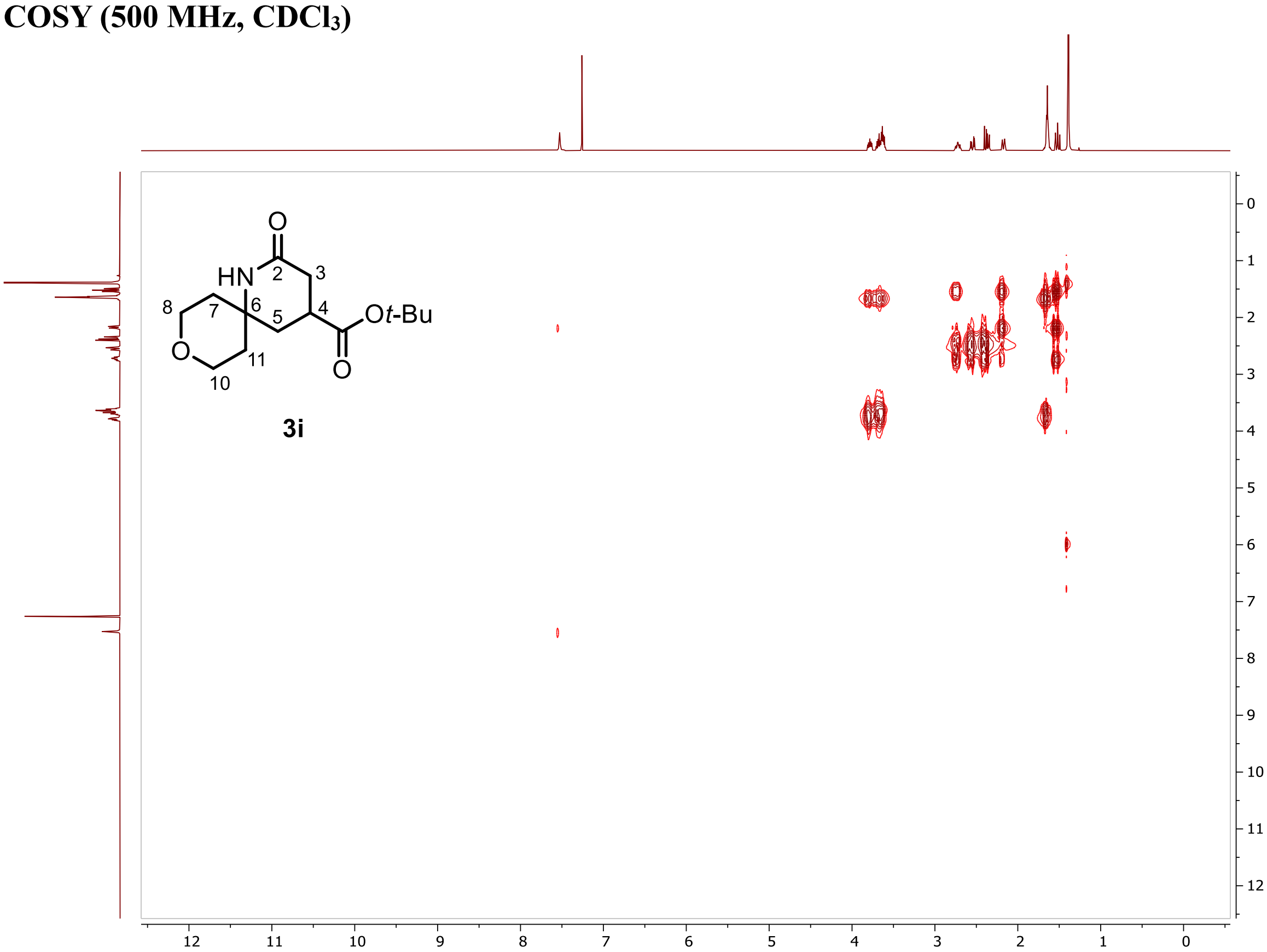


**HSQC ($^1$H 500 MHz/$^{13}$C 126 MHz, CDCl$_3$)**

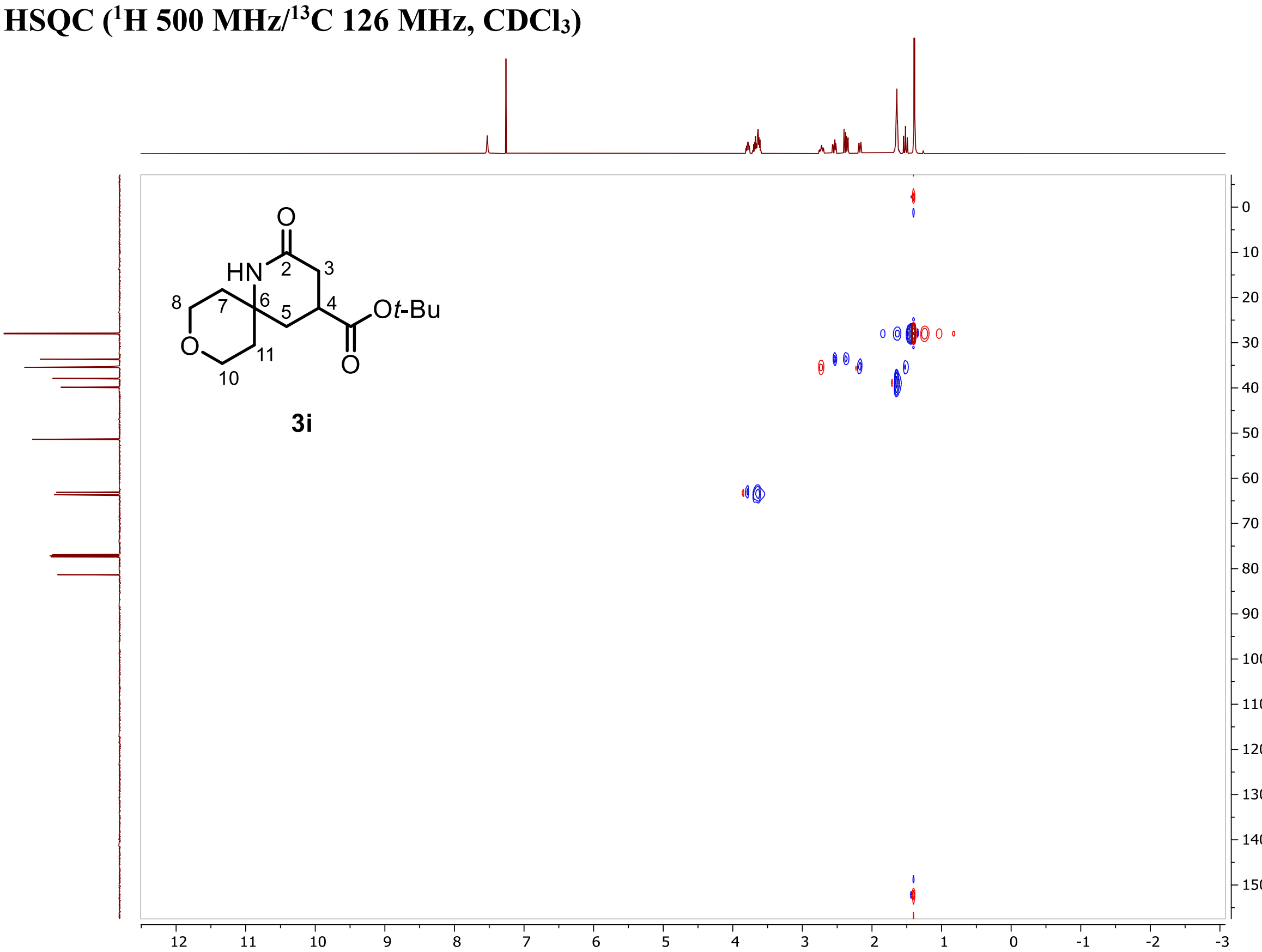

**$^{1}$H NMR (500 MHz, CDCl$_3$)**

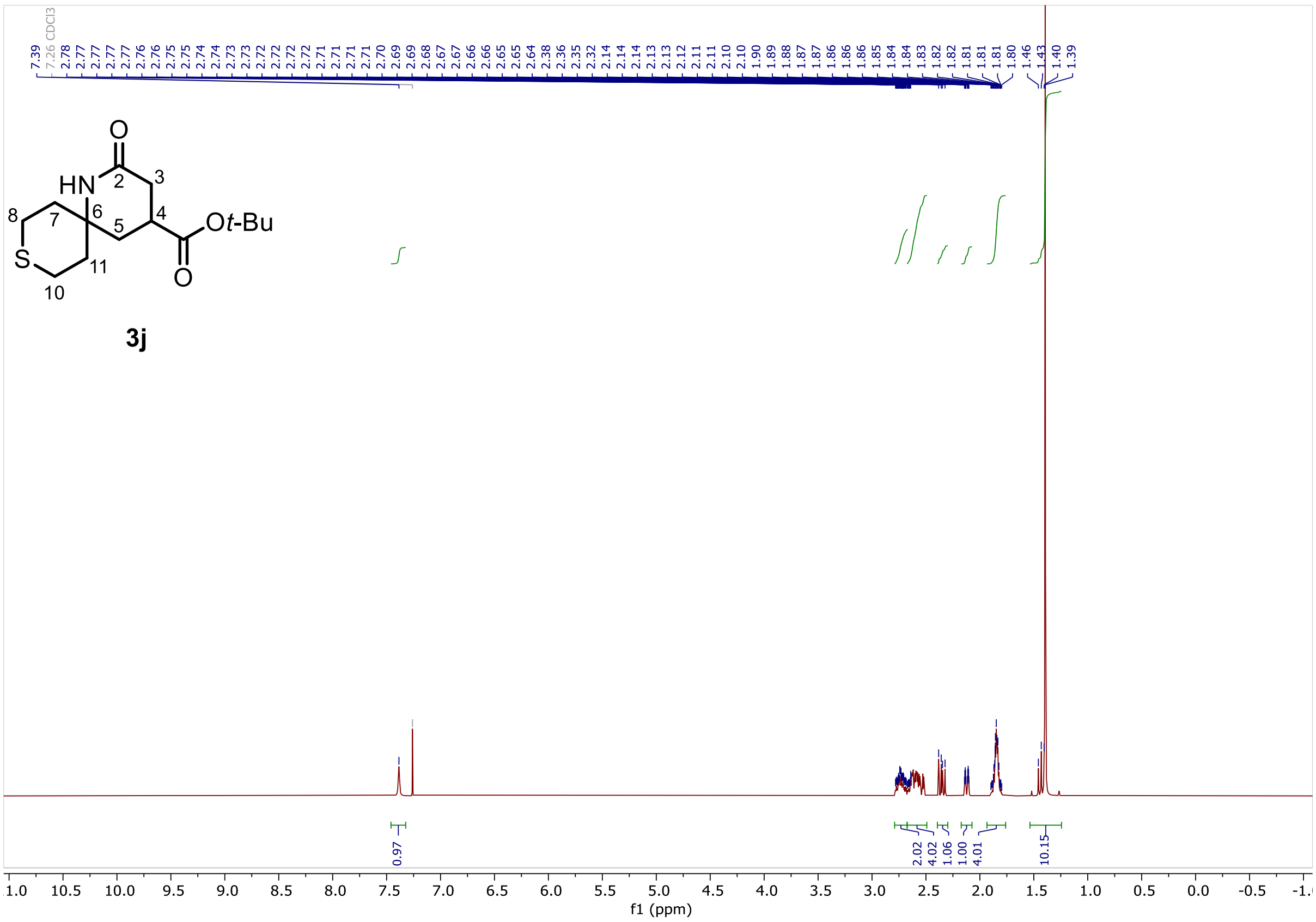


**$^{13}$C NMR (126 MHz, CDCl$_3$)**

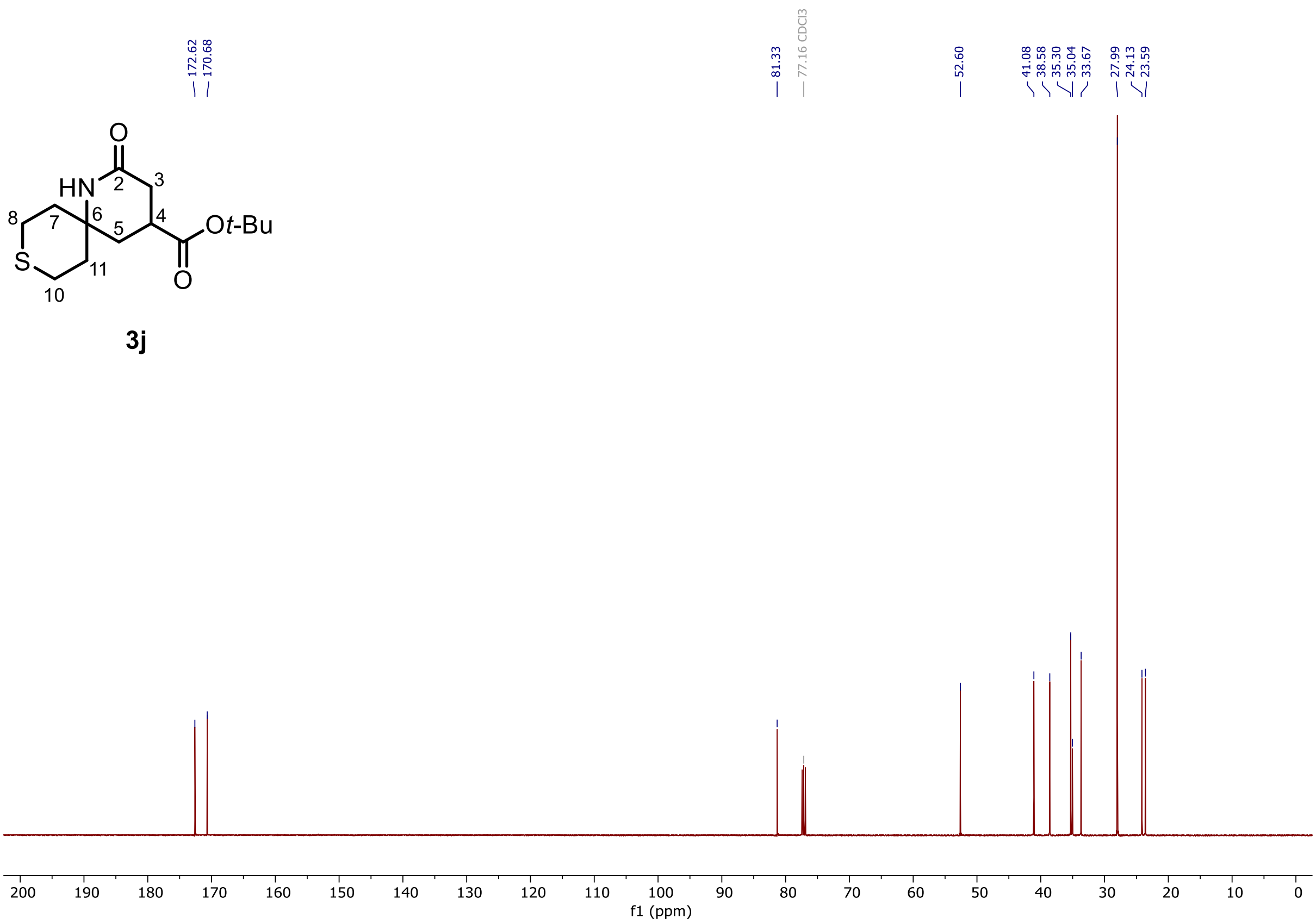

**COSY (500 MHz, CDCl$_3$)**

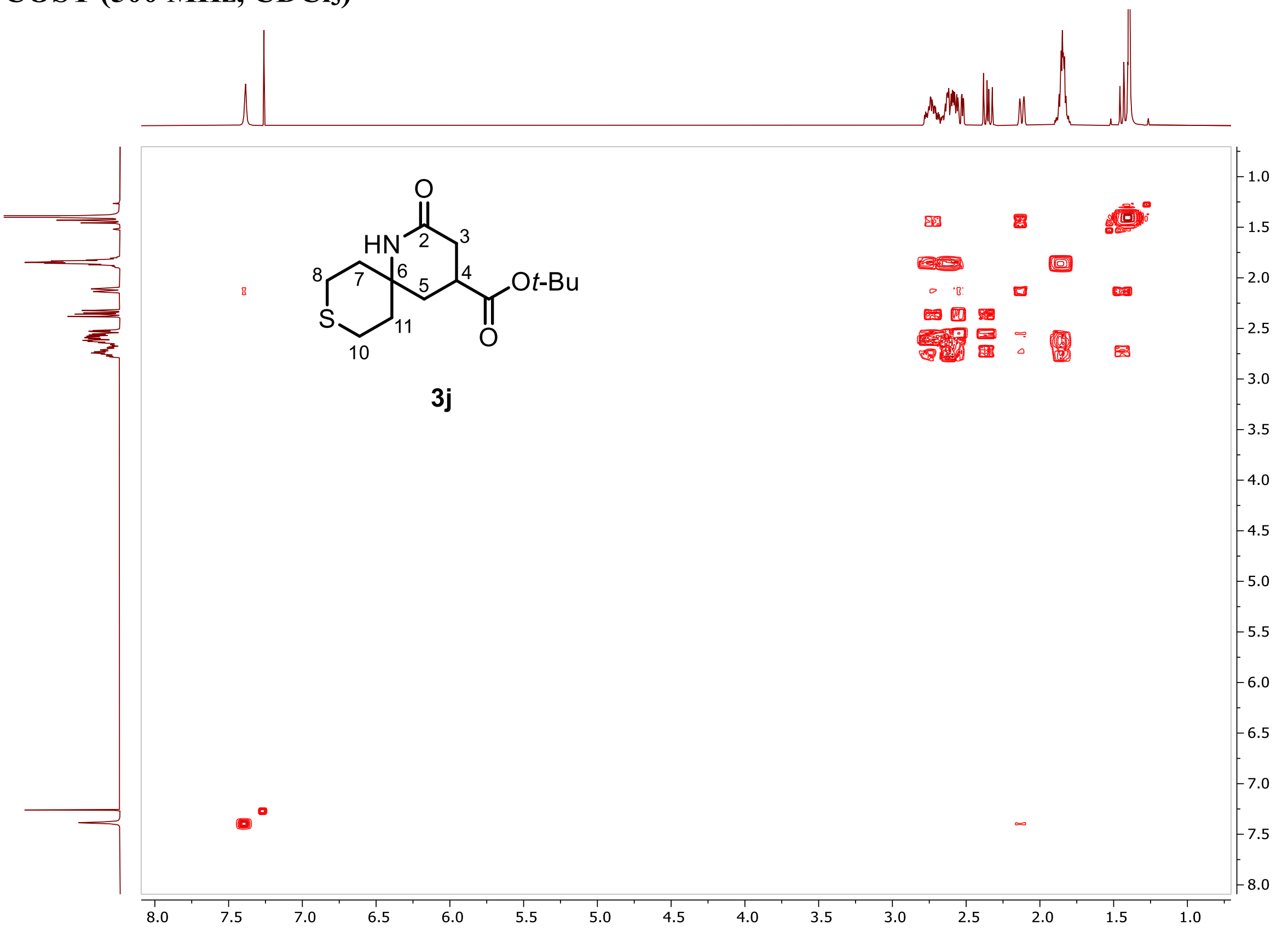


**HSQC ($^1$H 500 MHz/$^{13}$C 126 MHz, CDCl$_3$)**

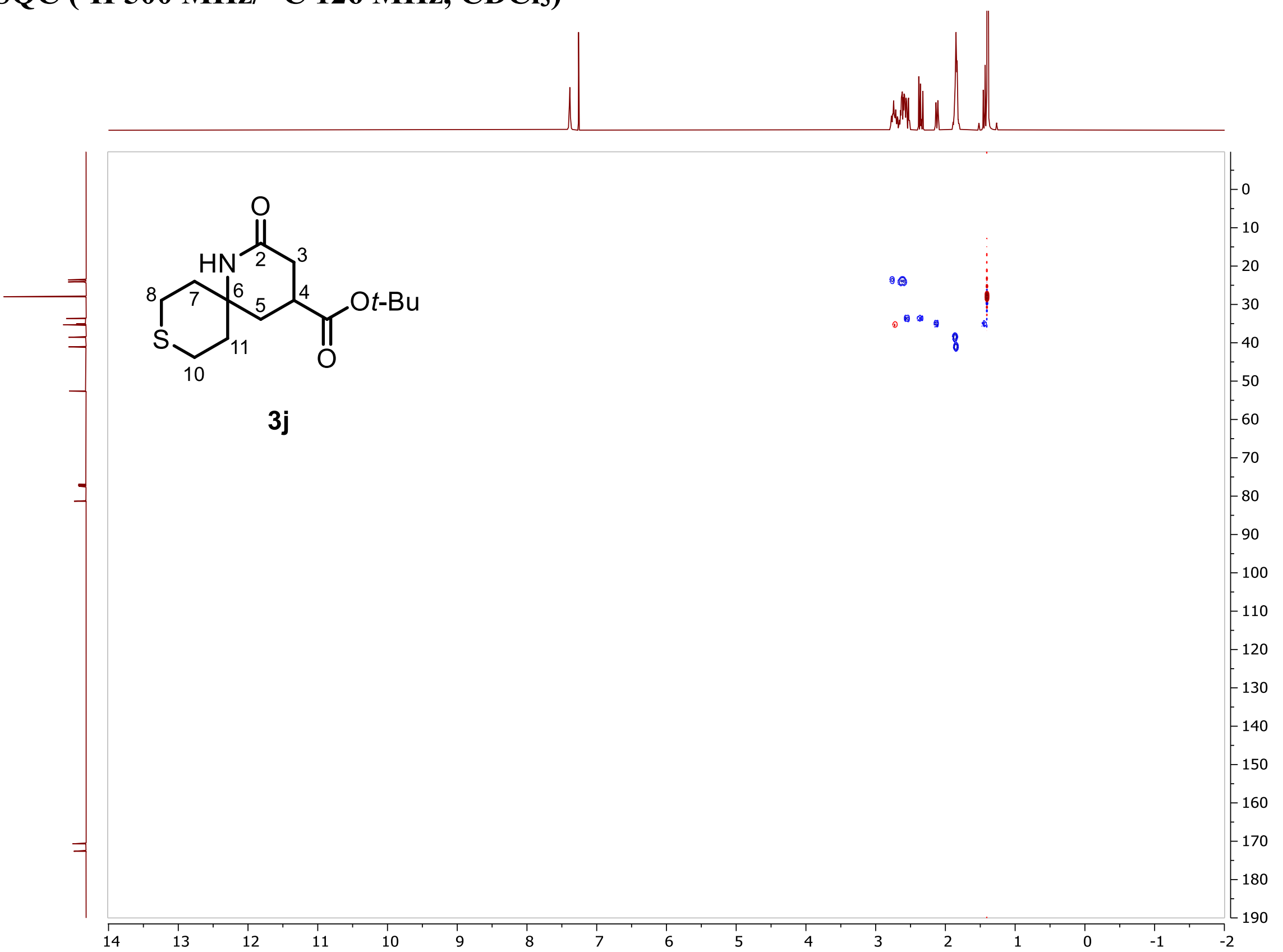

**$^{1}$H NMR (500 MHz, CDCl$_3$)**

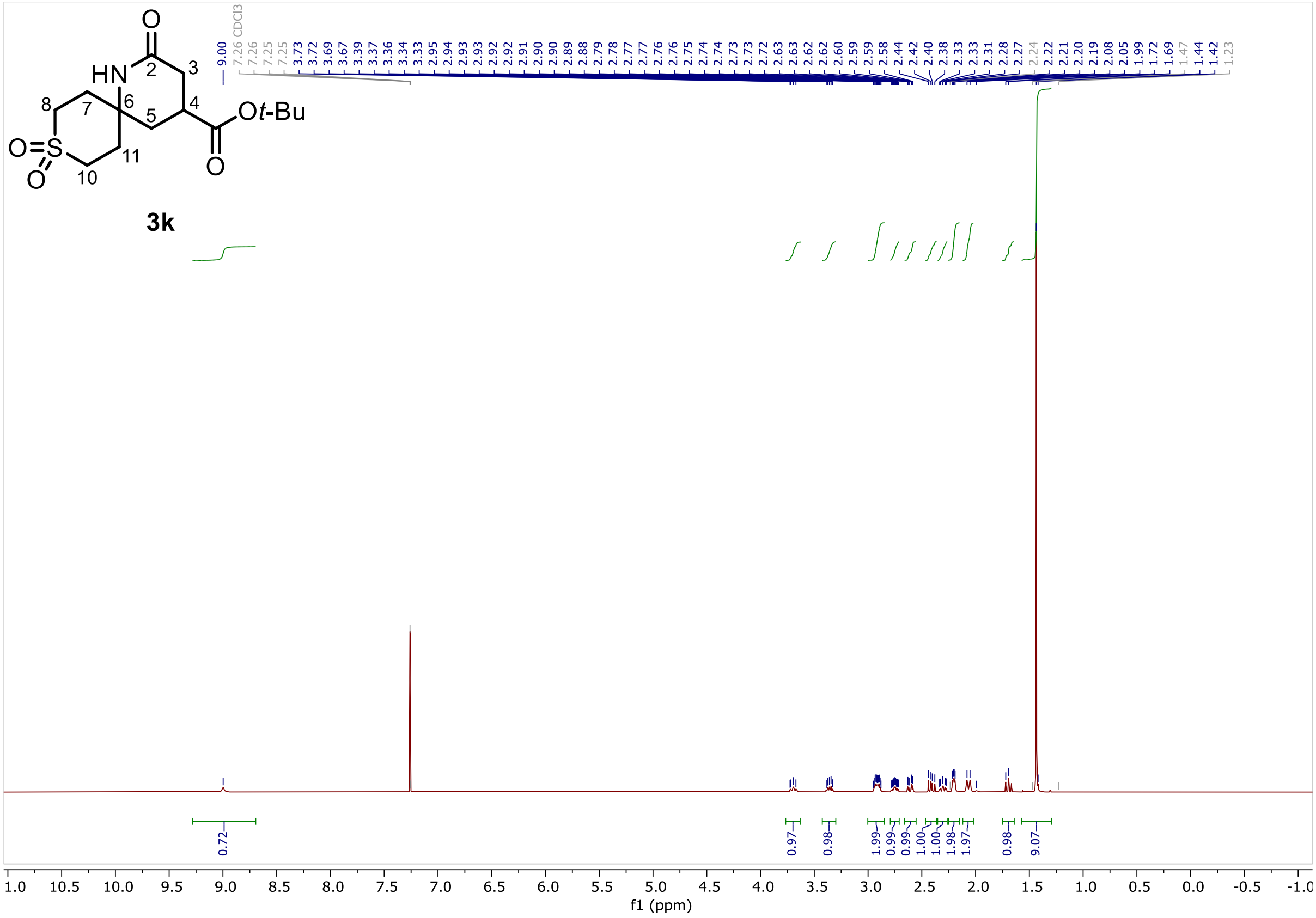


**$^{13}$C NMR (126 MHz, CDCl$_3$)**

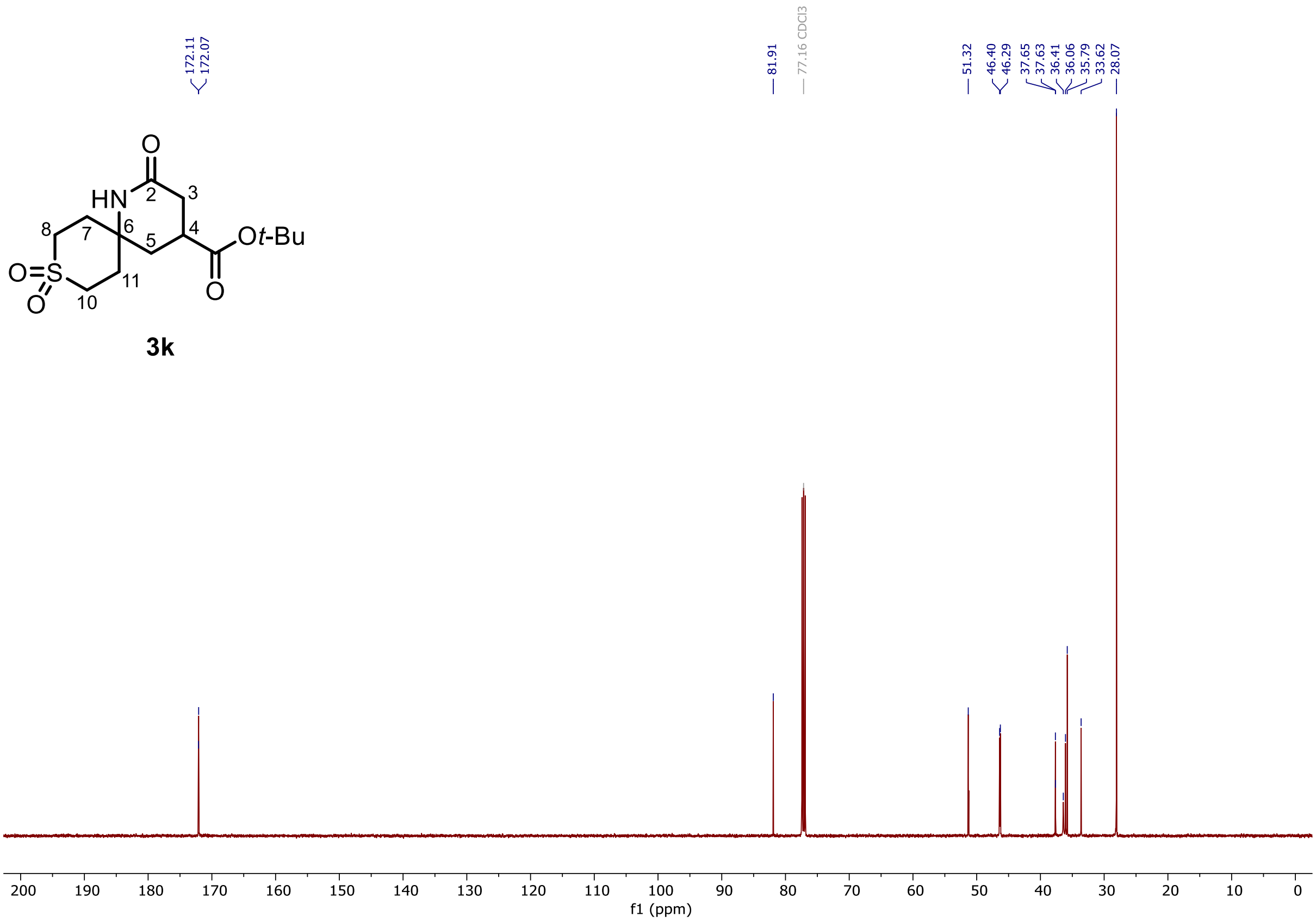

**COSY (500 MHz, CDCl$_3$)**

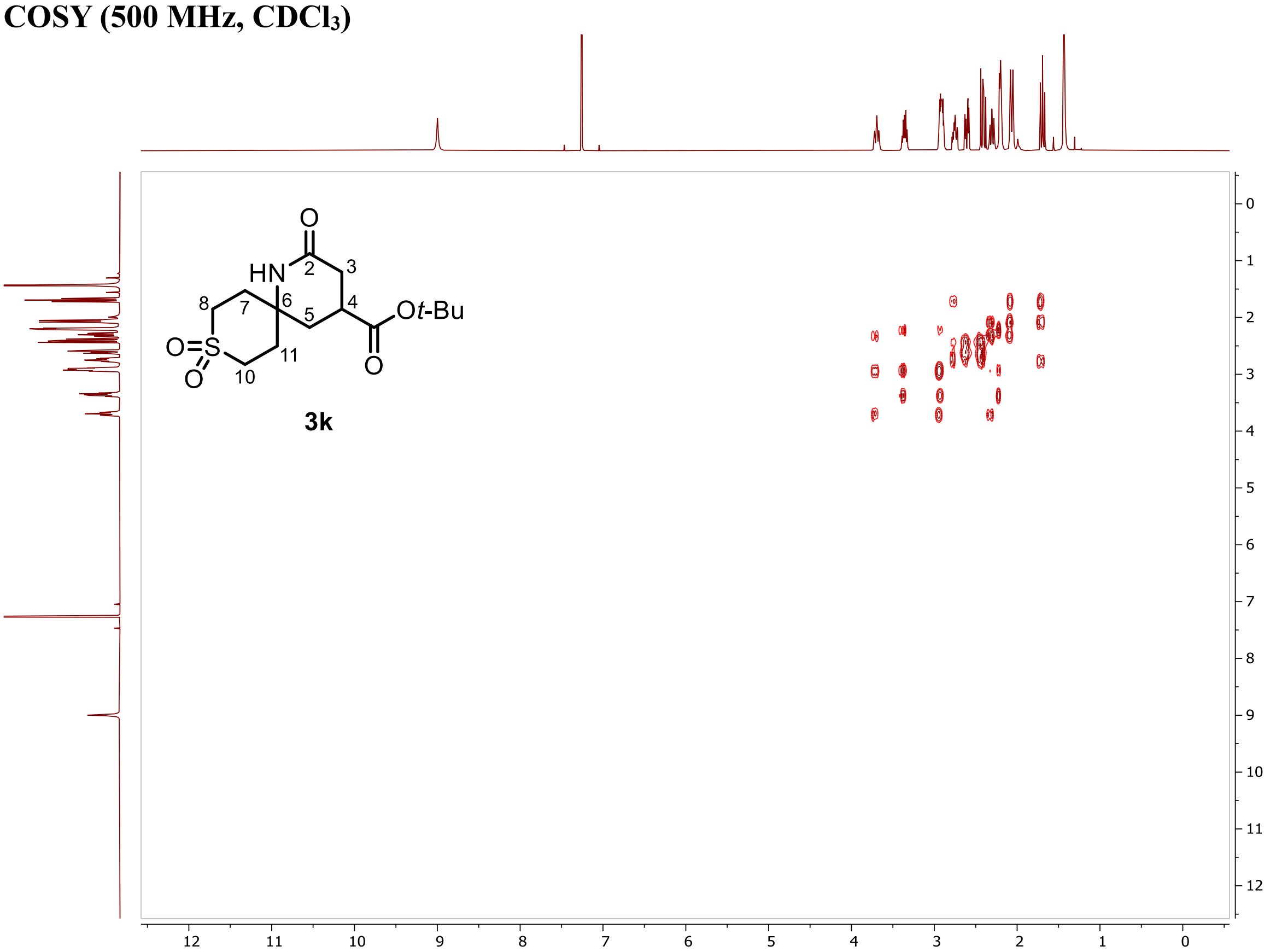


**HSQC ($^1$H 500 MHz/$^{13}$C 126 MHz, CDCl$_3$)**

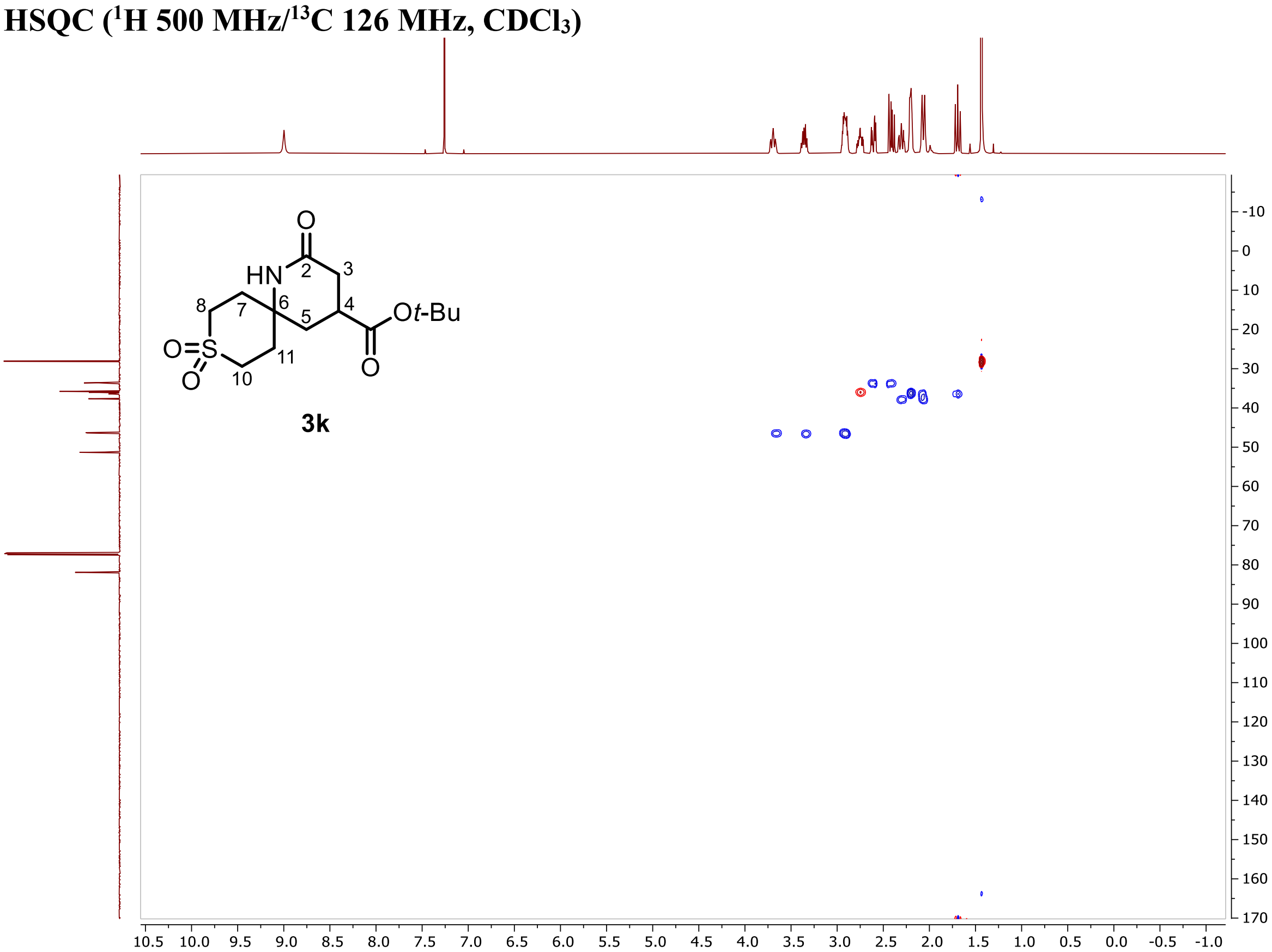

**$^{1}$H NMR (500 MHz, CDCl$_3$)**

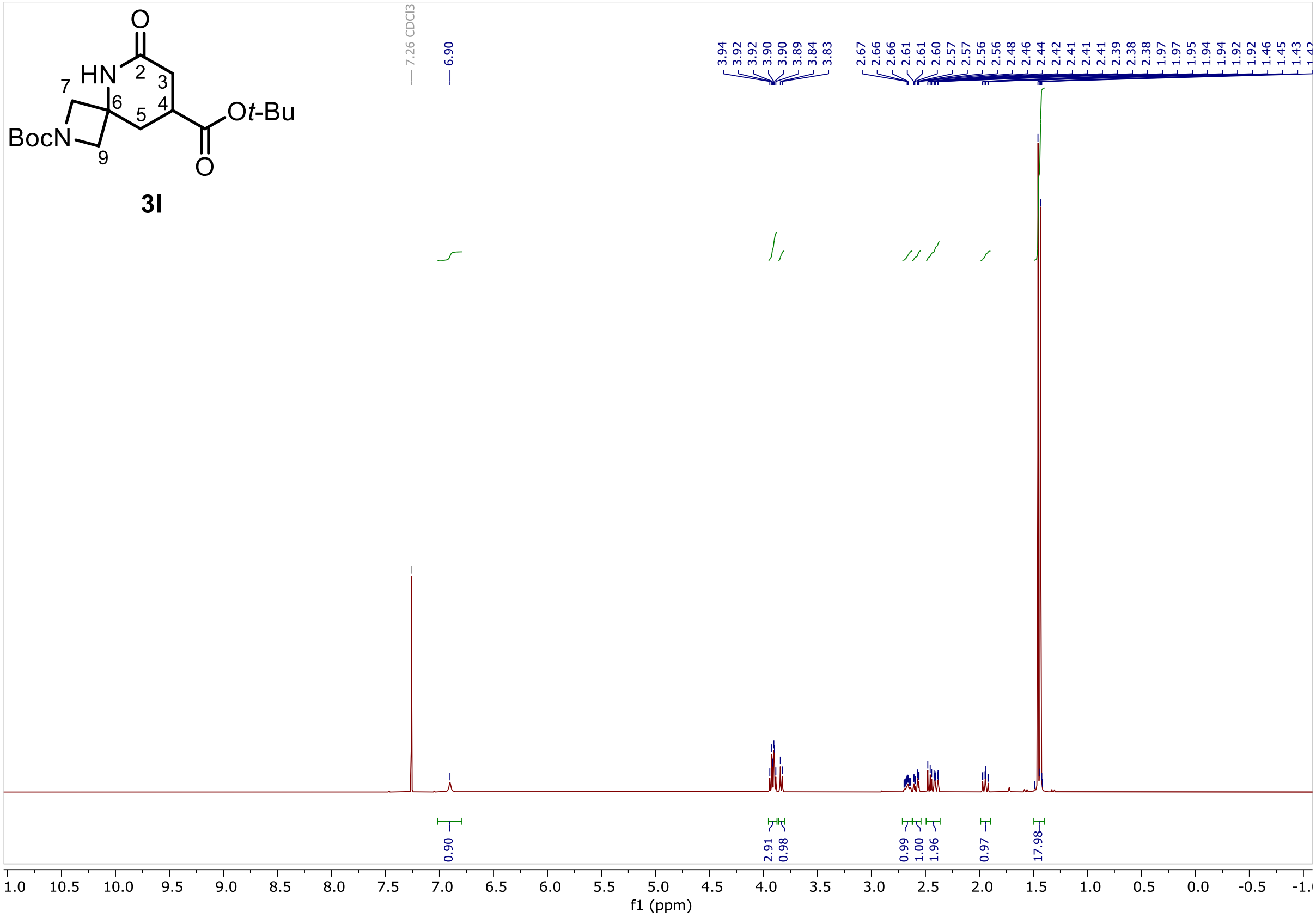


**$^{13}$C NMR (126 MHz, CDCl$_3$)**

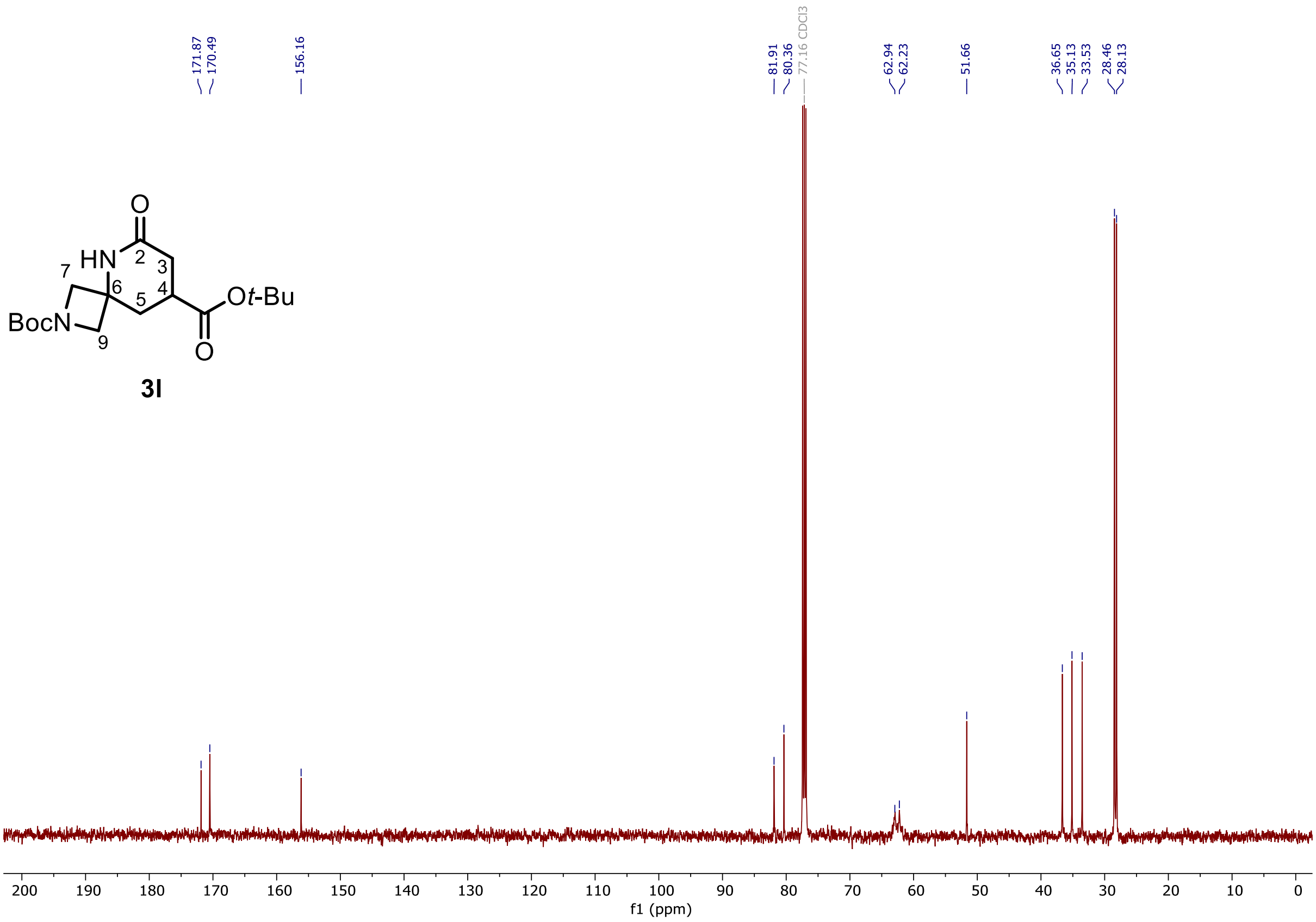

**COSY (500 MHz, CDCl$_3$)**

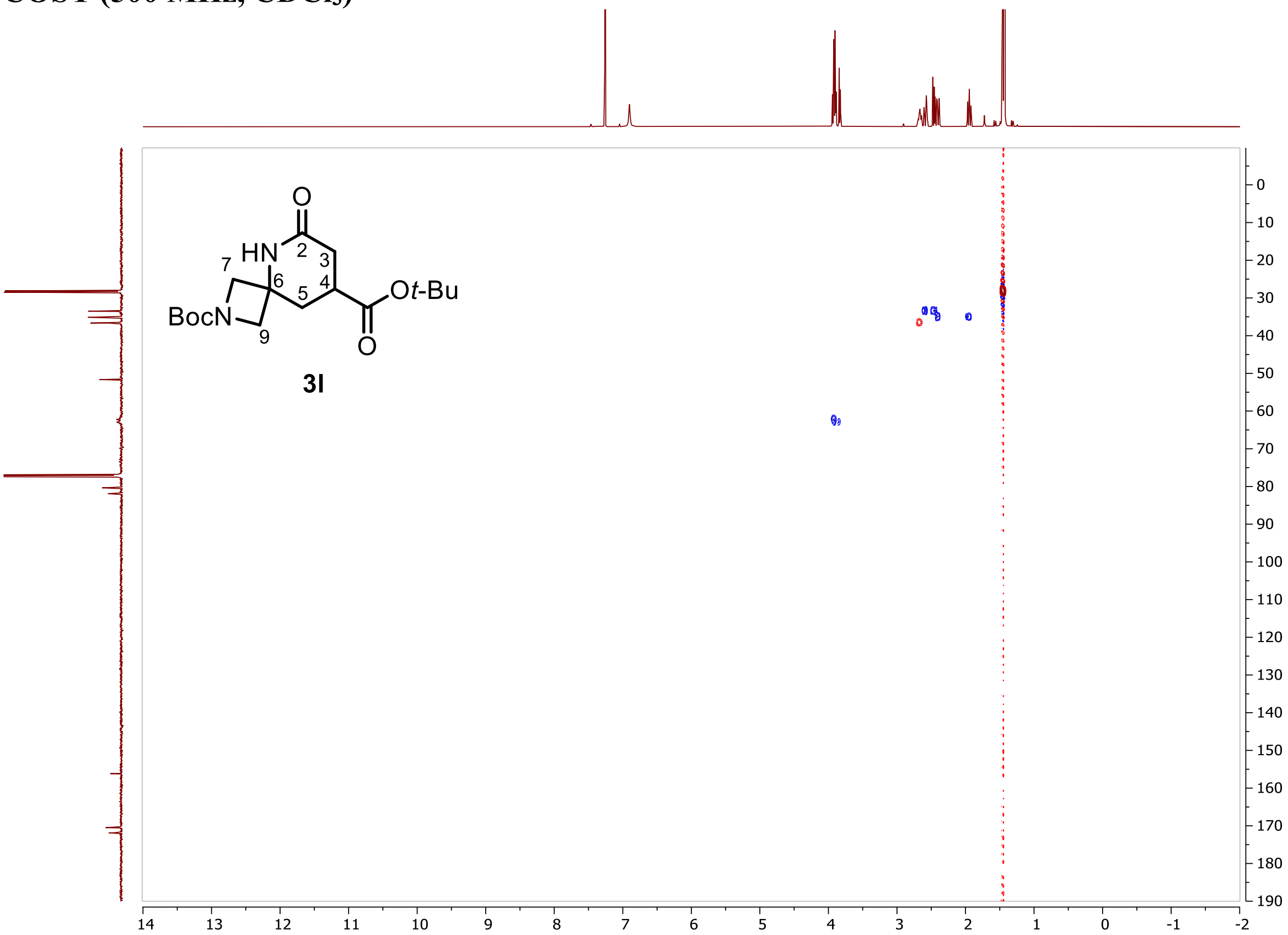


**HSQC ($^1$H 500 MHz/$^{13}$C 126 MHz, CDCl$_3$)**

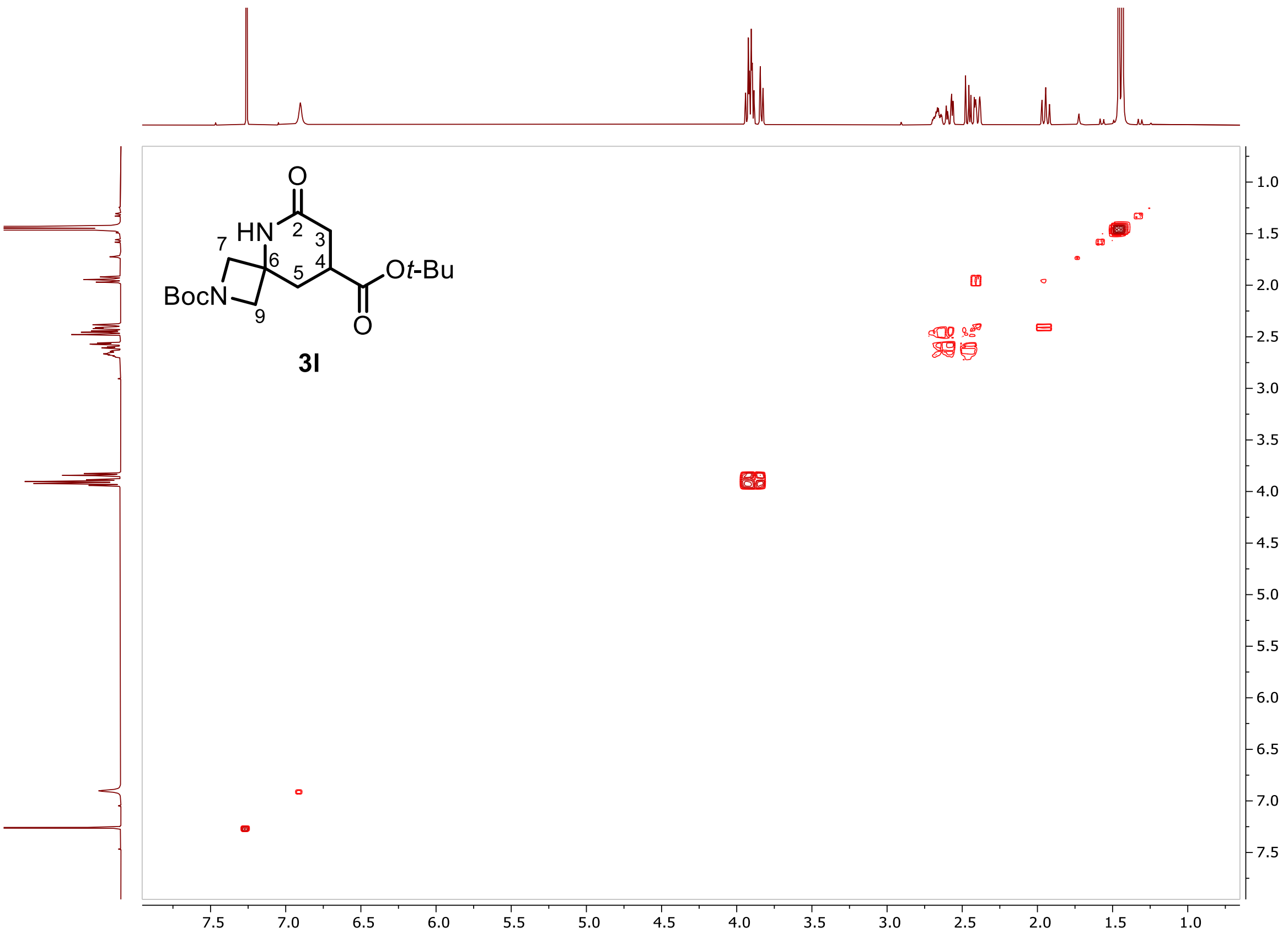

**$^1$H NMR (500 MHz, CDCl$_3$)**

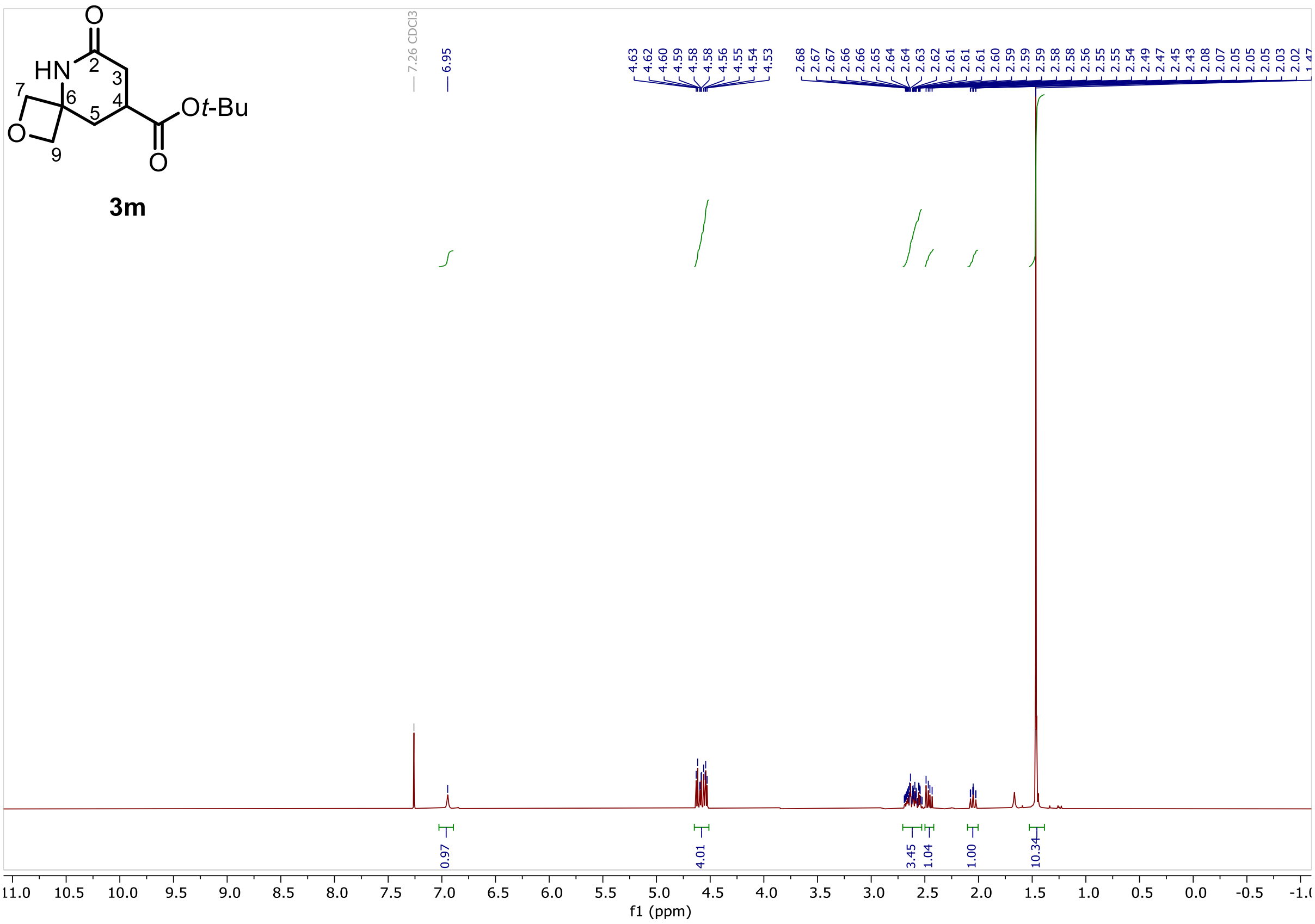

**$^{13}$C NMR (126 MHz, CDCl$_3$)**

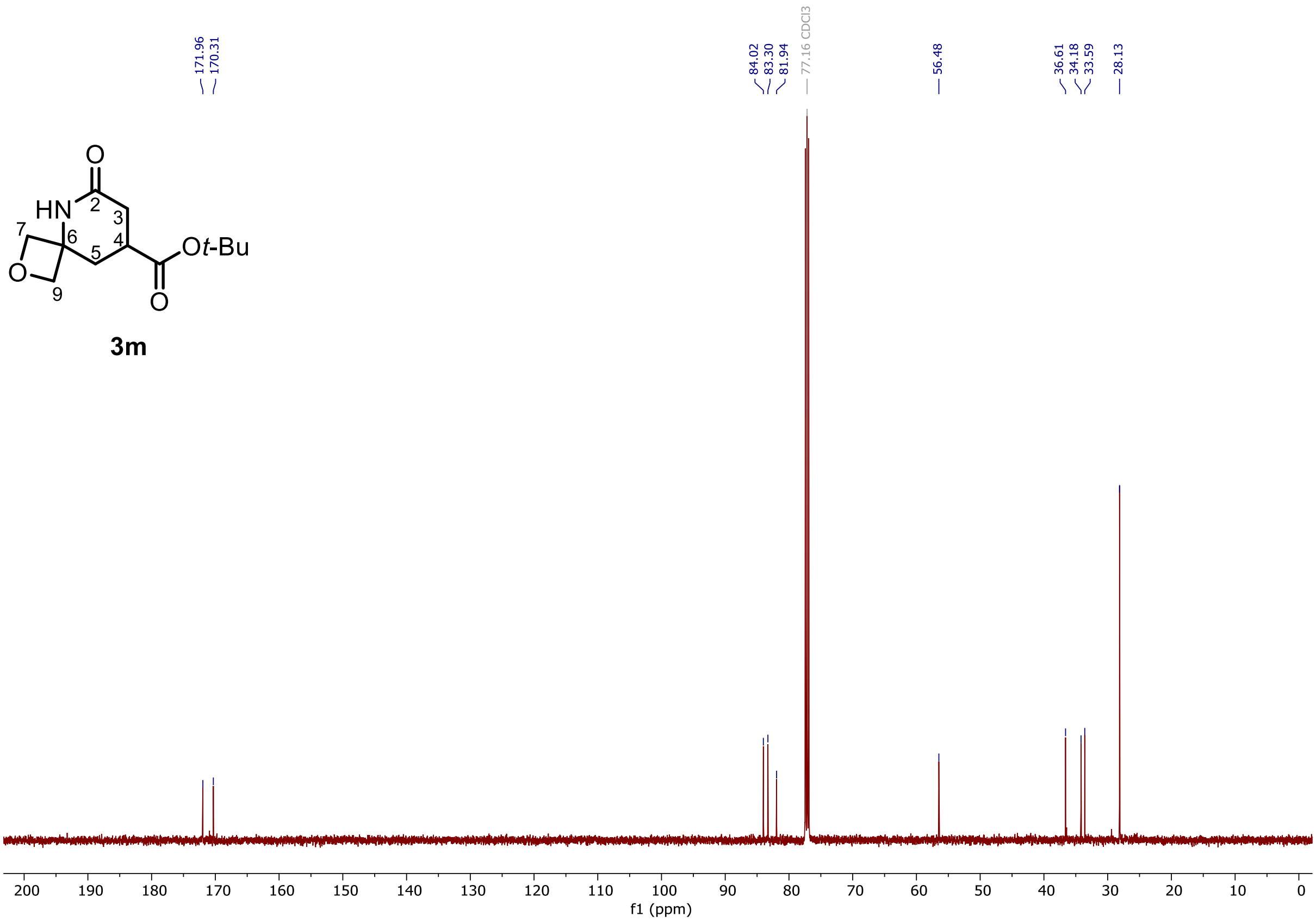

**COSY (500 MHz, $CDCl_3$)**

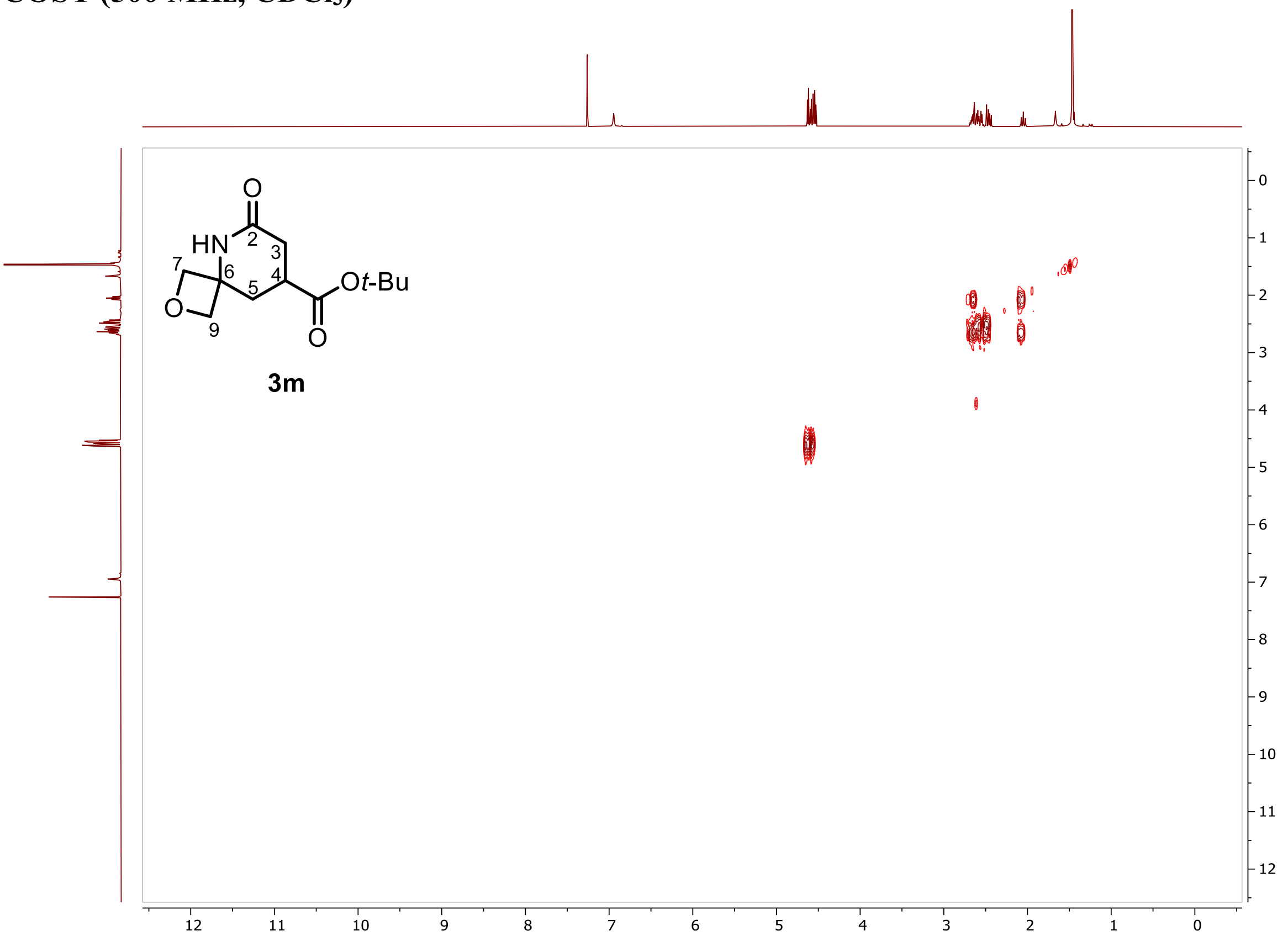


**HSQC ($^1H$ 500 MHz/$^{13}C$ 126 MHz, $CDCl_3$)**

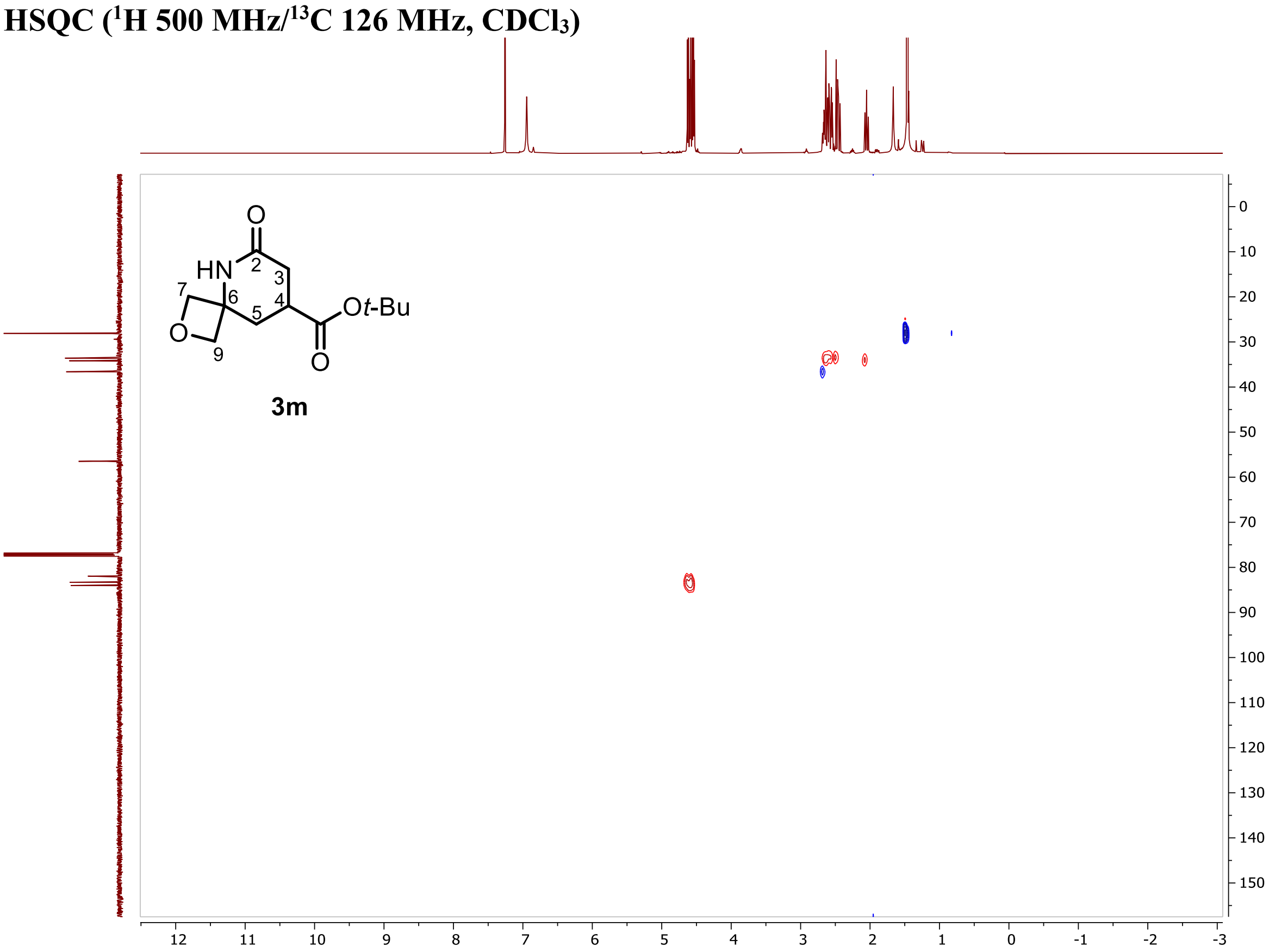

**$^{1}$H NMR (500 MHz, $CDCl_3$)**

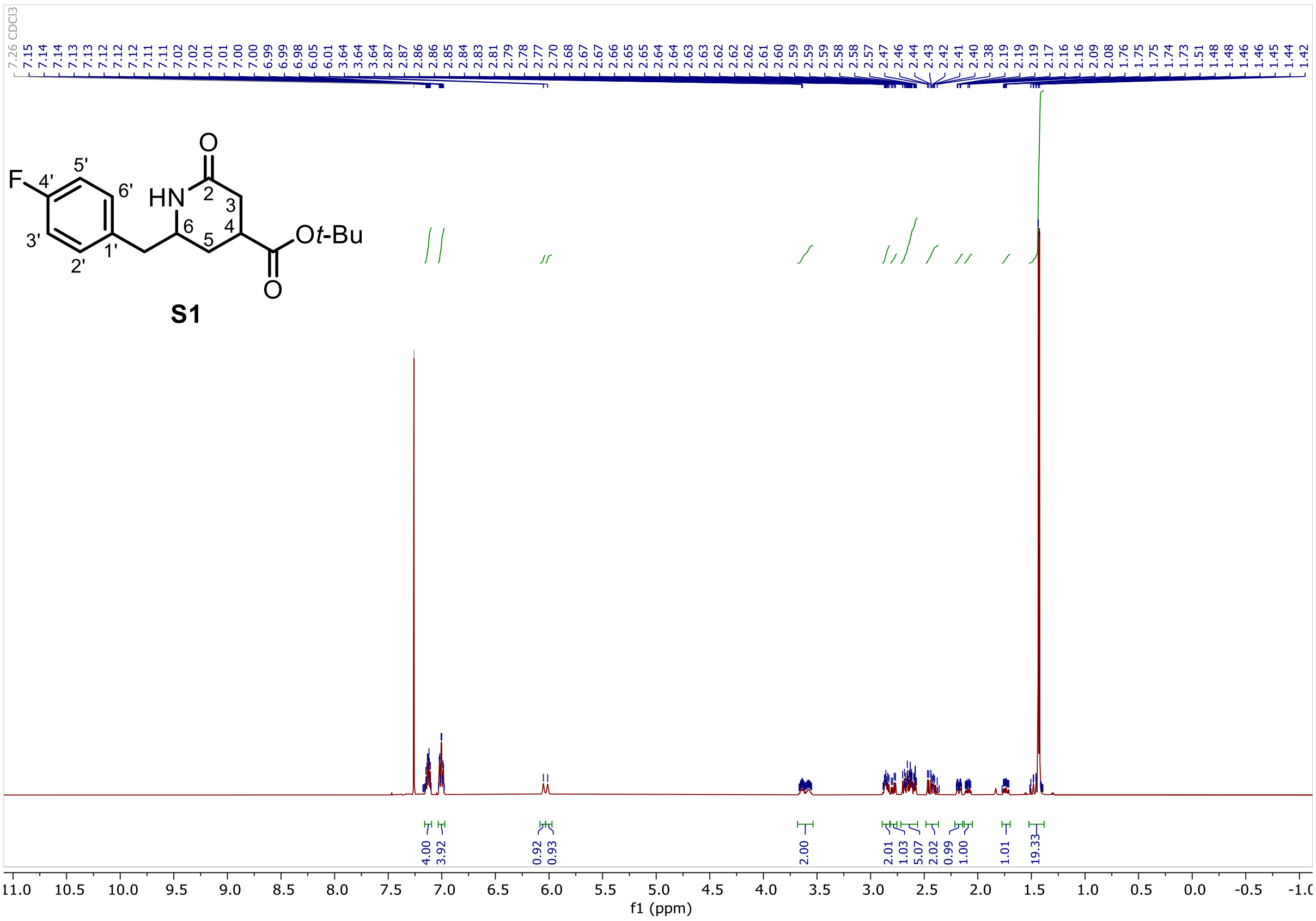


**$^{13}$C NMR (126 MHz, $CDCl_3$)**

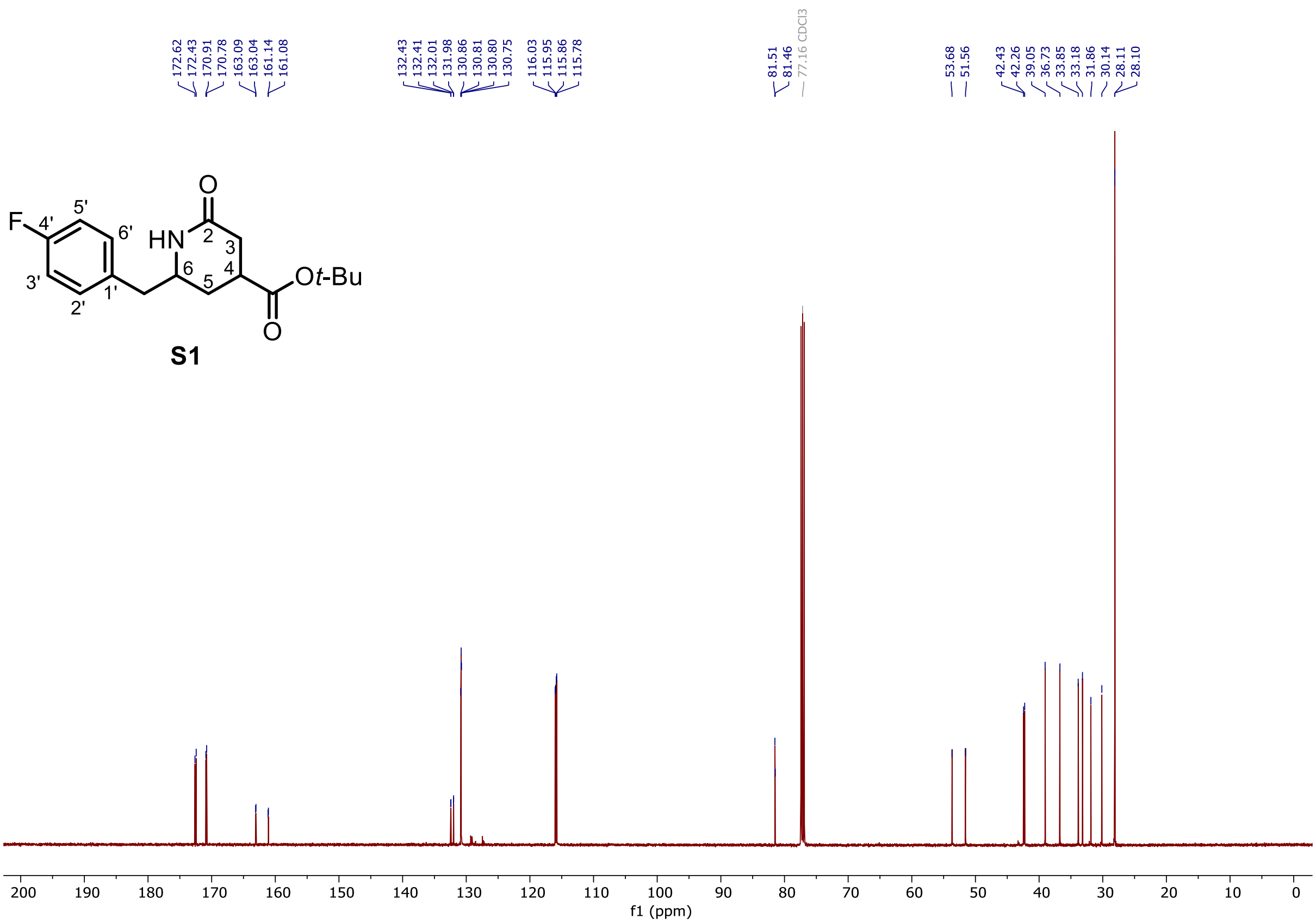

**COSY (500 MHz, CDCl$_3$)**

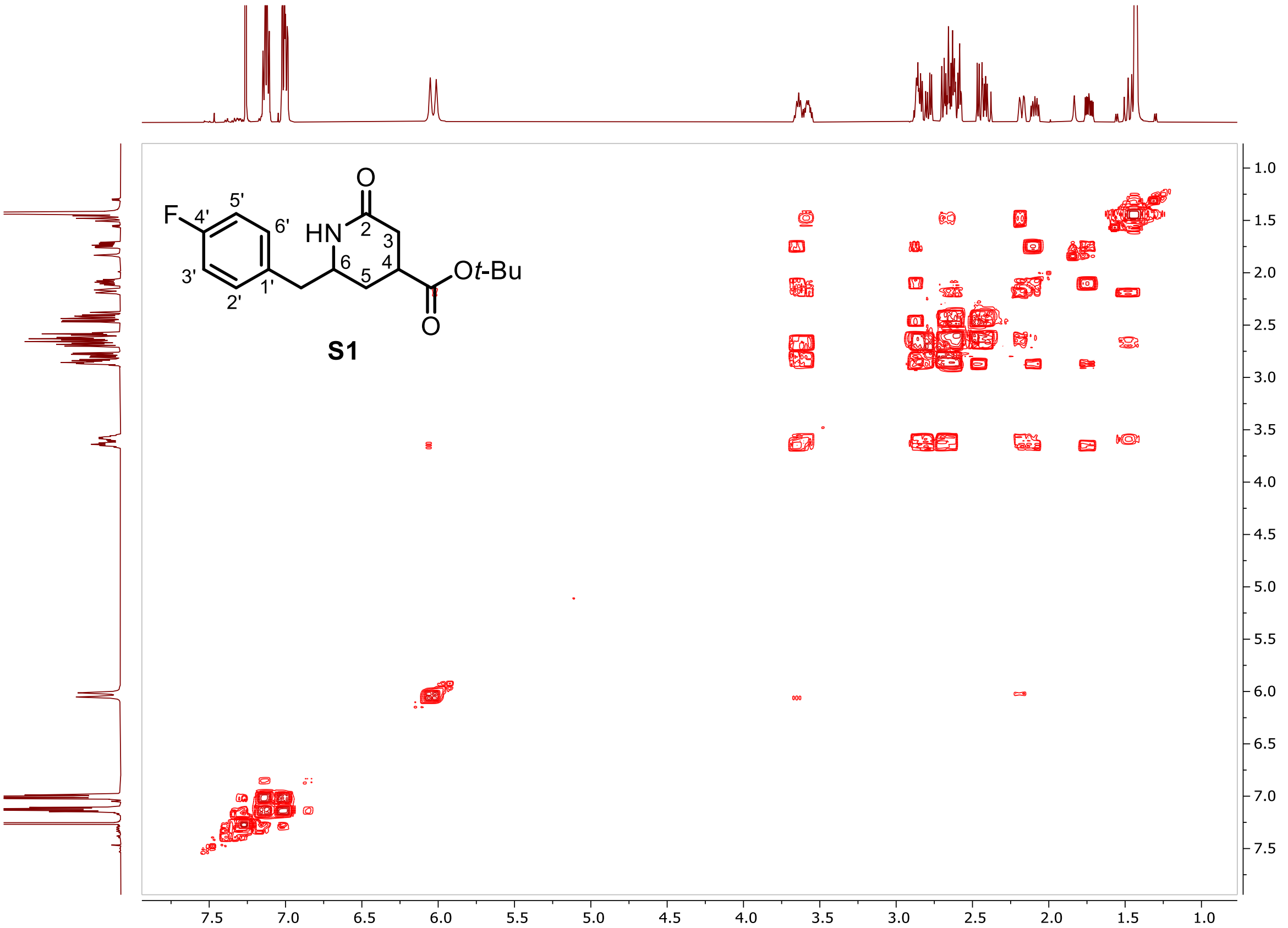


**HSQC ($^1$H 500 MHz/$^{13}$C 126 MHz, CDCl$_3$)**

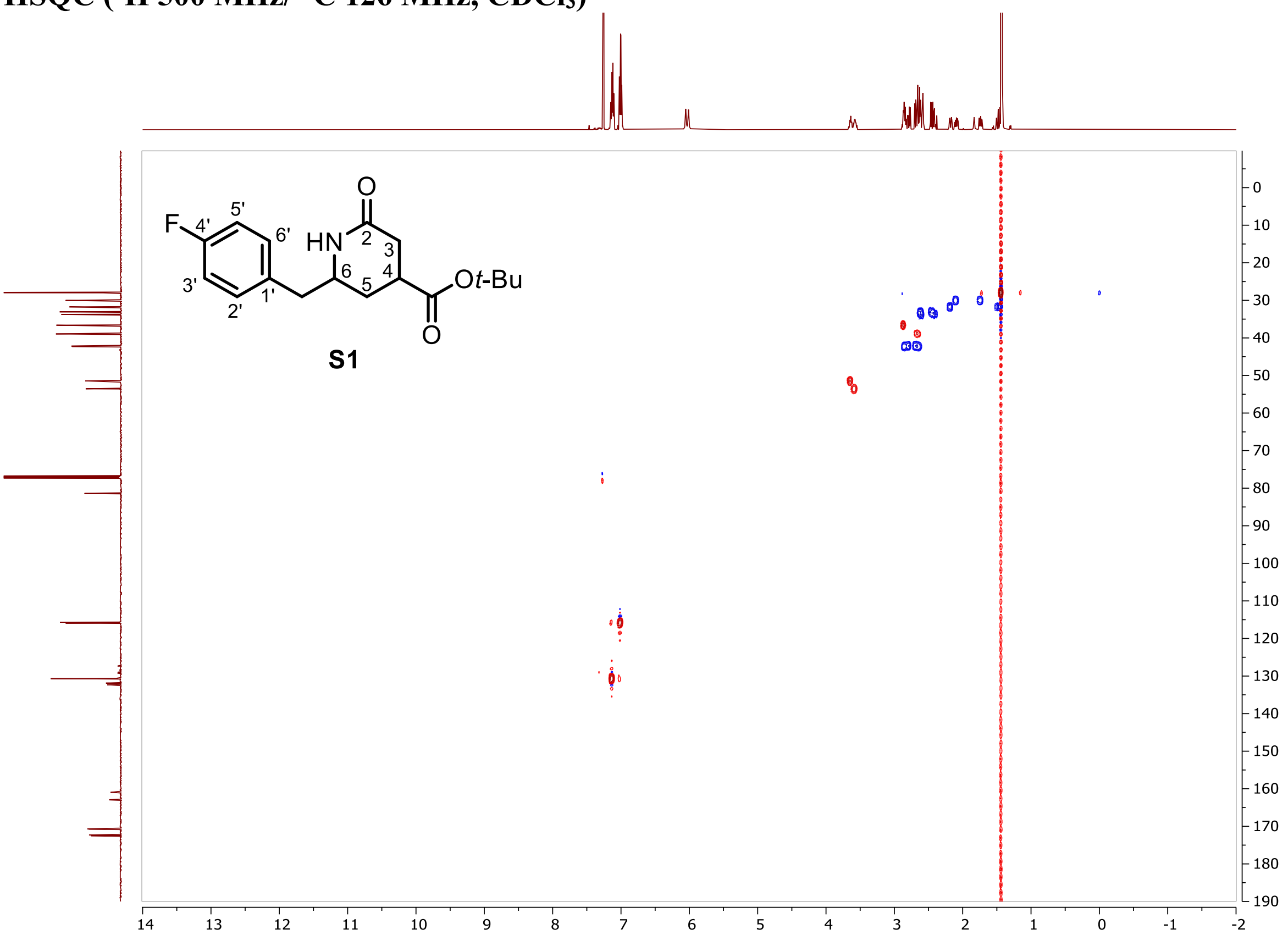

## $^{1}$H NMR (400 MHz, $CDCl_3$)

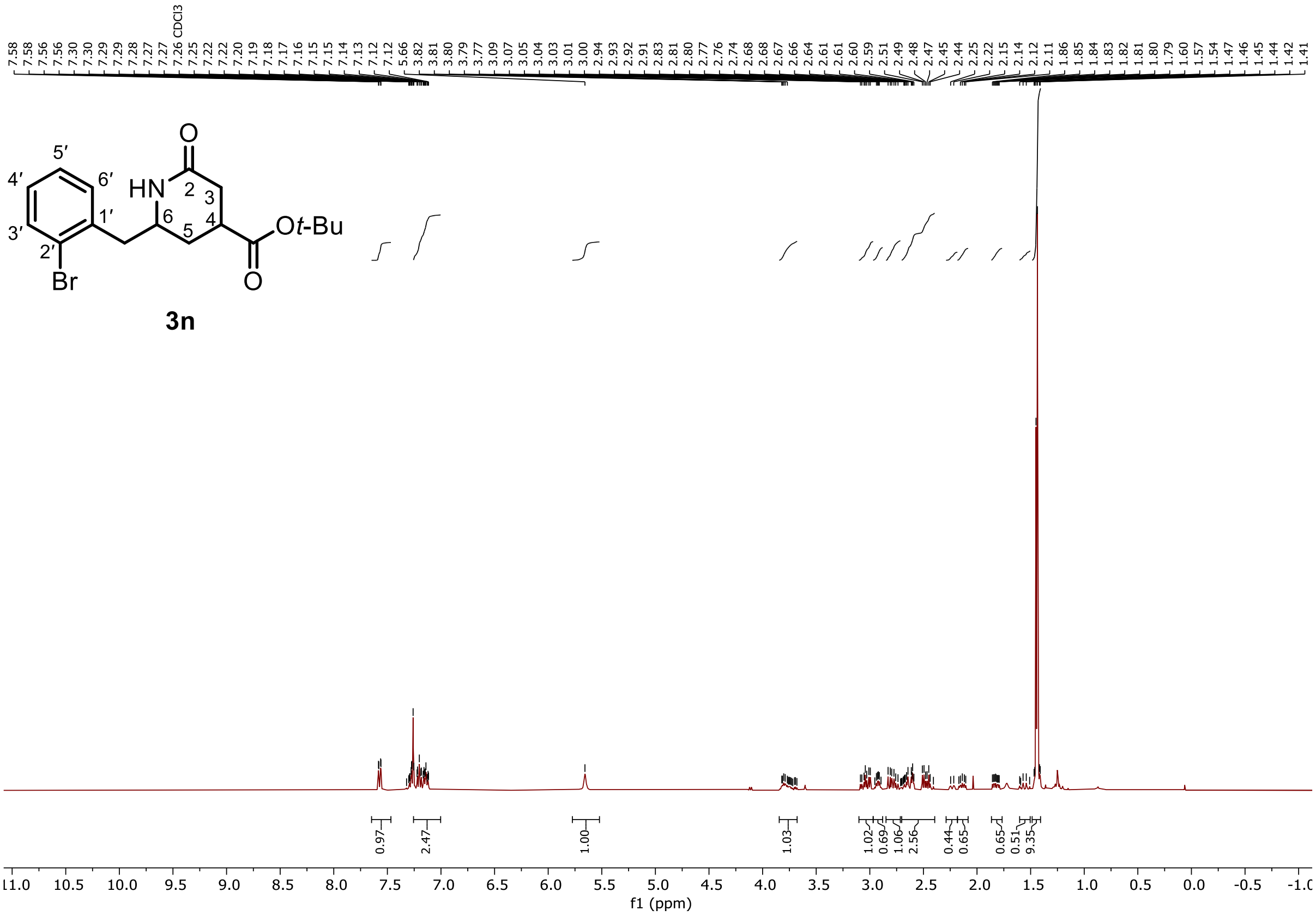


## $^{13}$C NMR (101 MHz, $CDCl_3$)

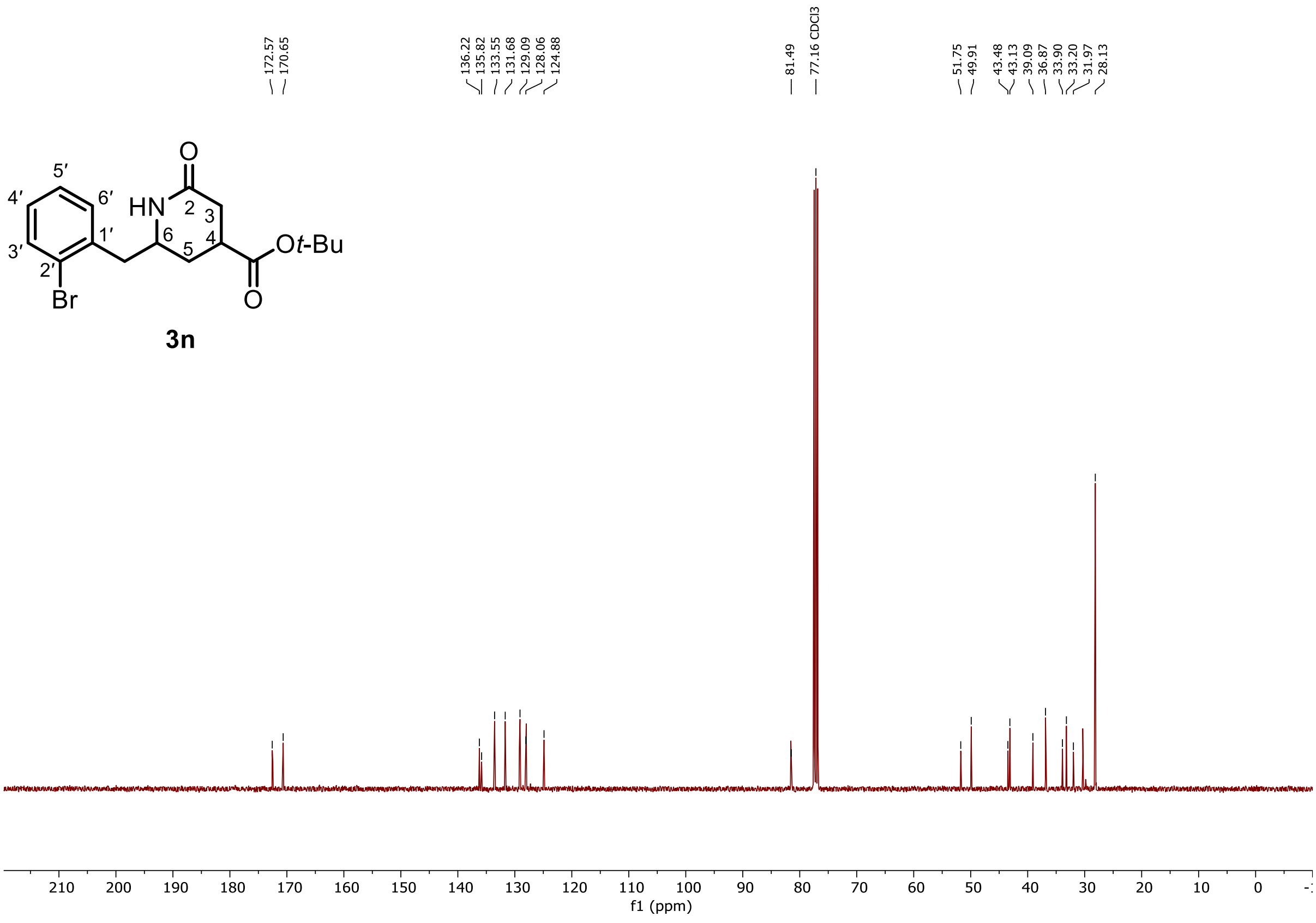

**COSY (500 MHz, $CDCl_3$)**

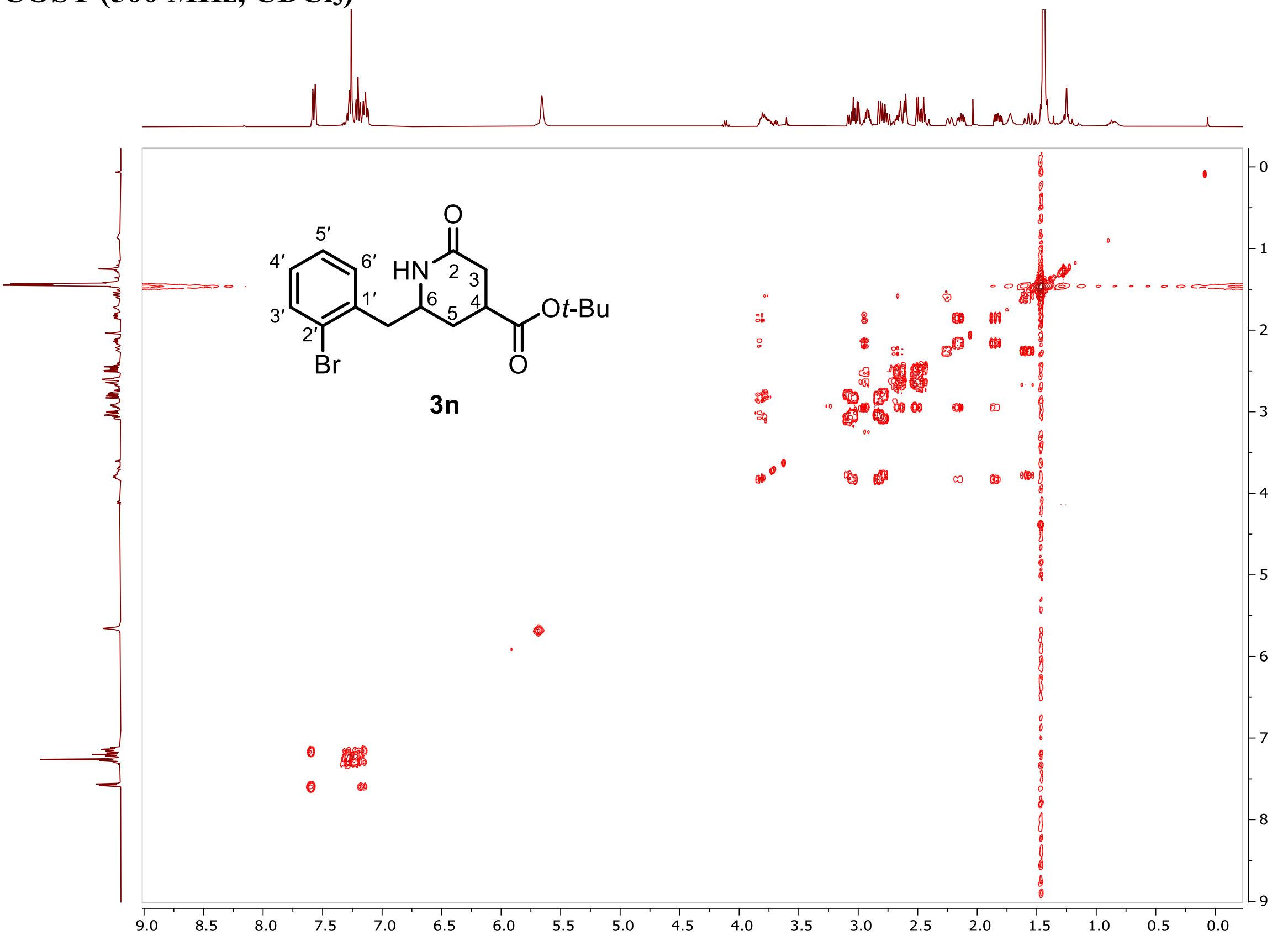


**HSQC ($^1H$ 500 MHz/$^{13}C$ 126 MHz, $CDCl_3$)**

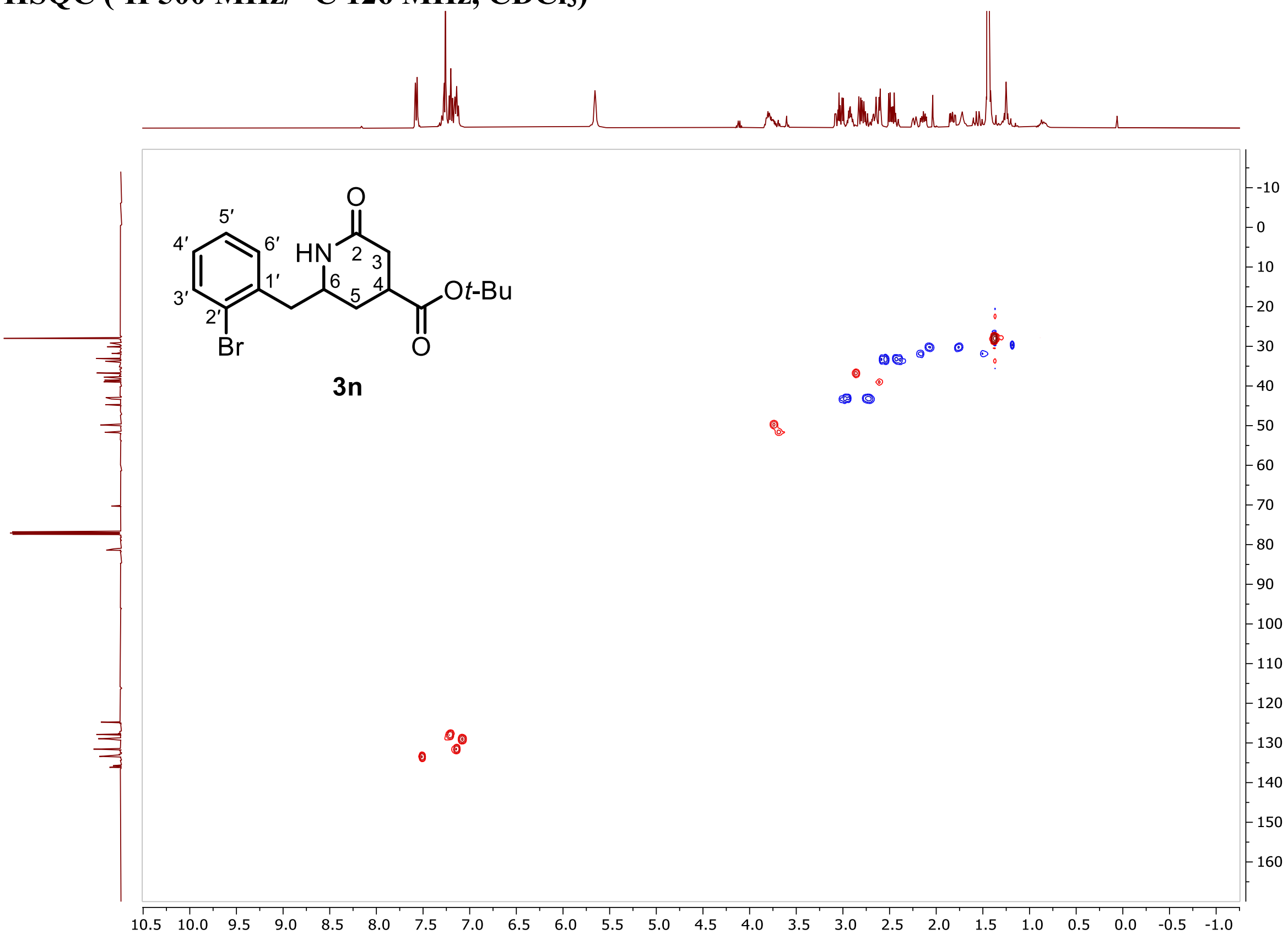

## $^{1}$H NMR (500 MHz, $CDCl_3$)

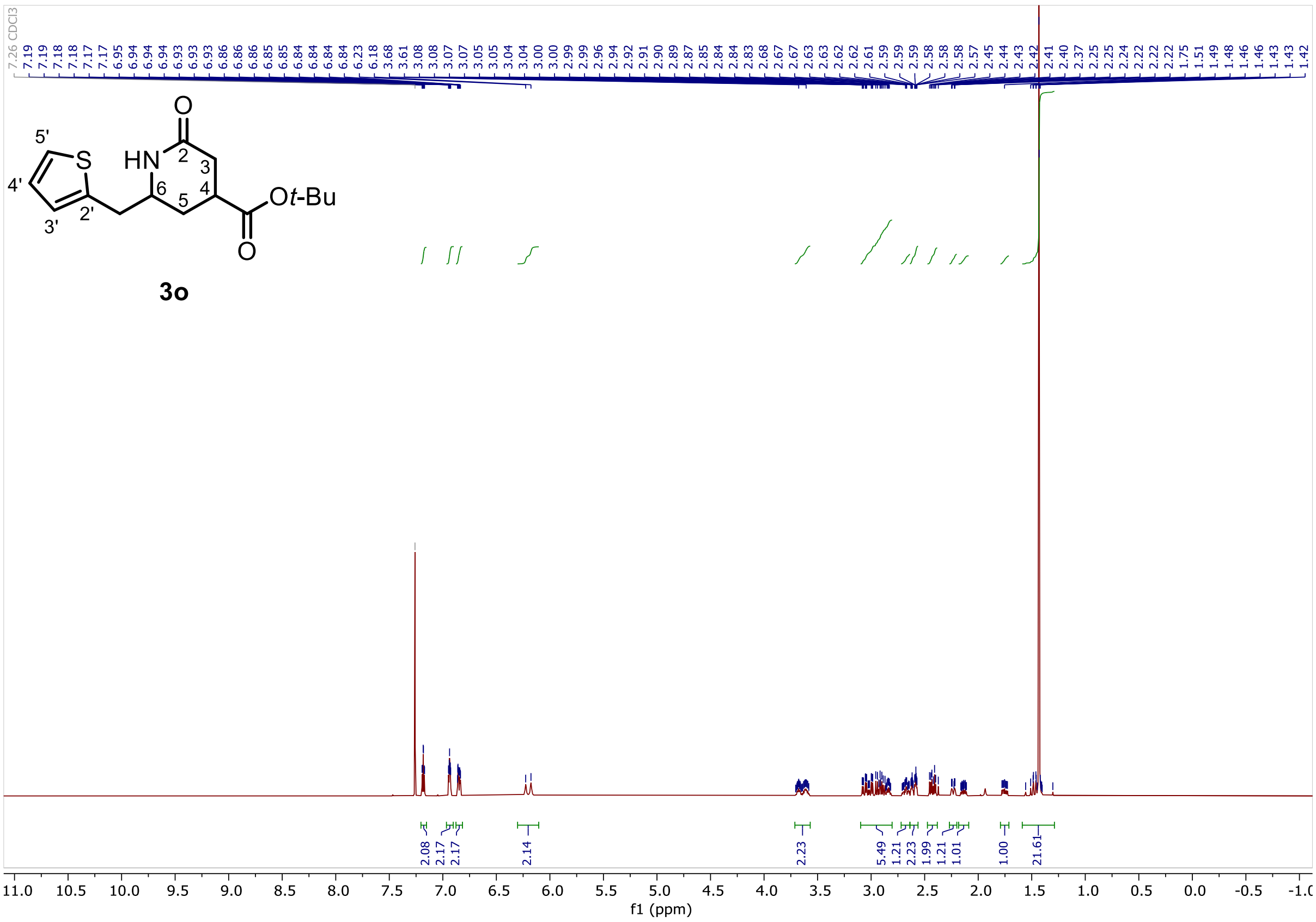


## $^{13}$C NMR (126 MHz, $CDCl_3$)

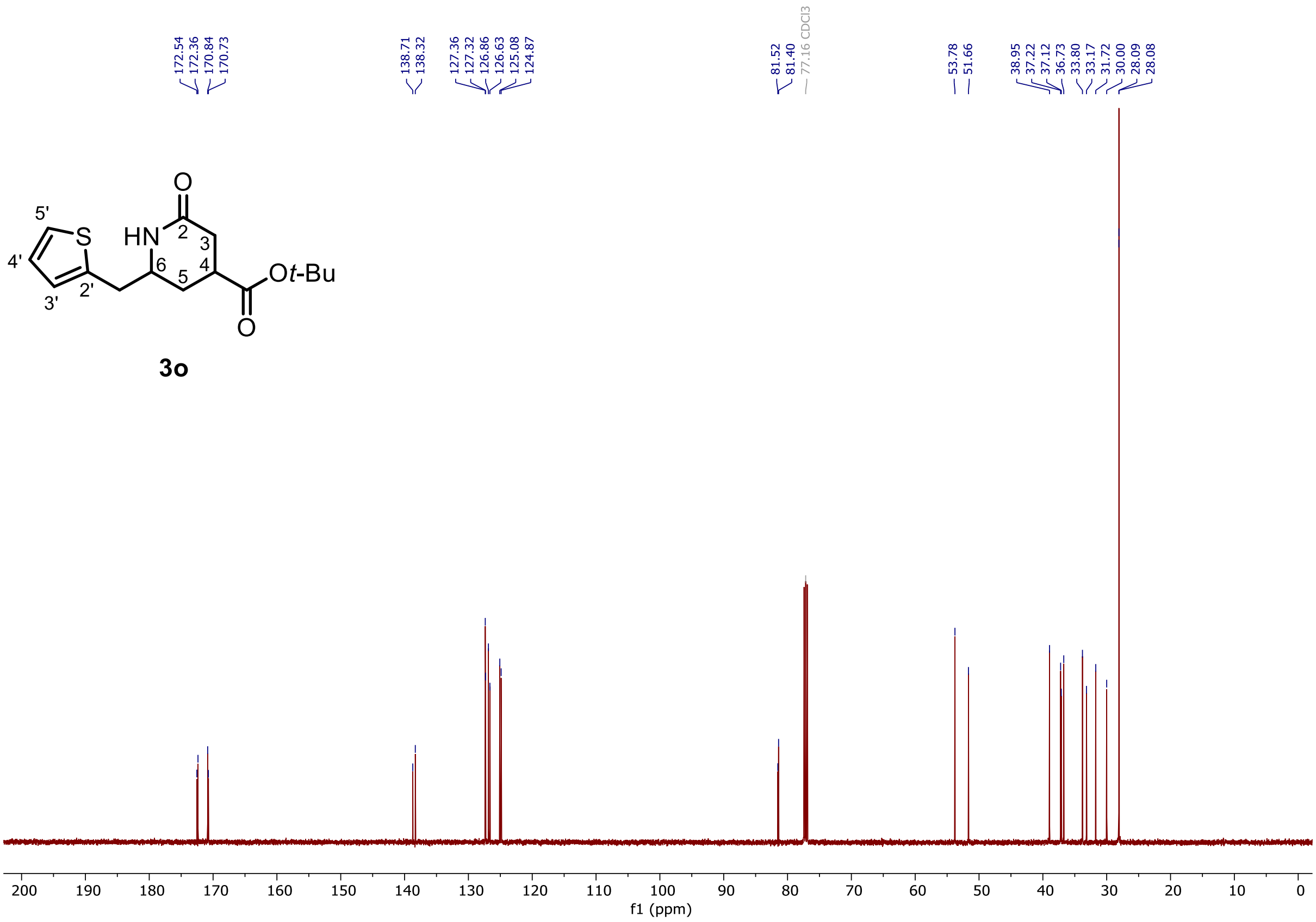

**COSY (500 MHz, CDCl$_3$)**

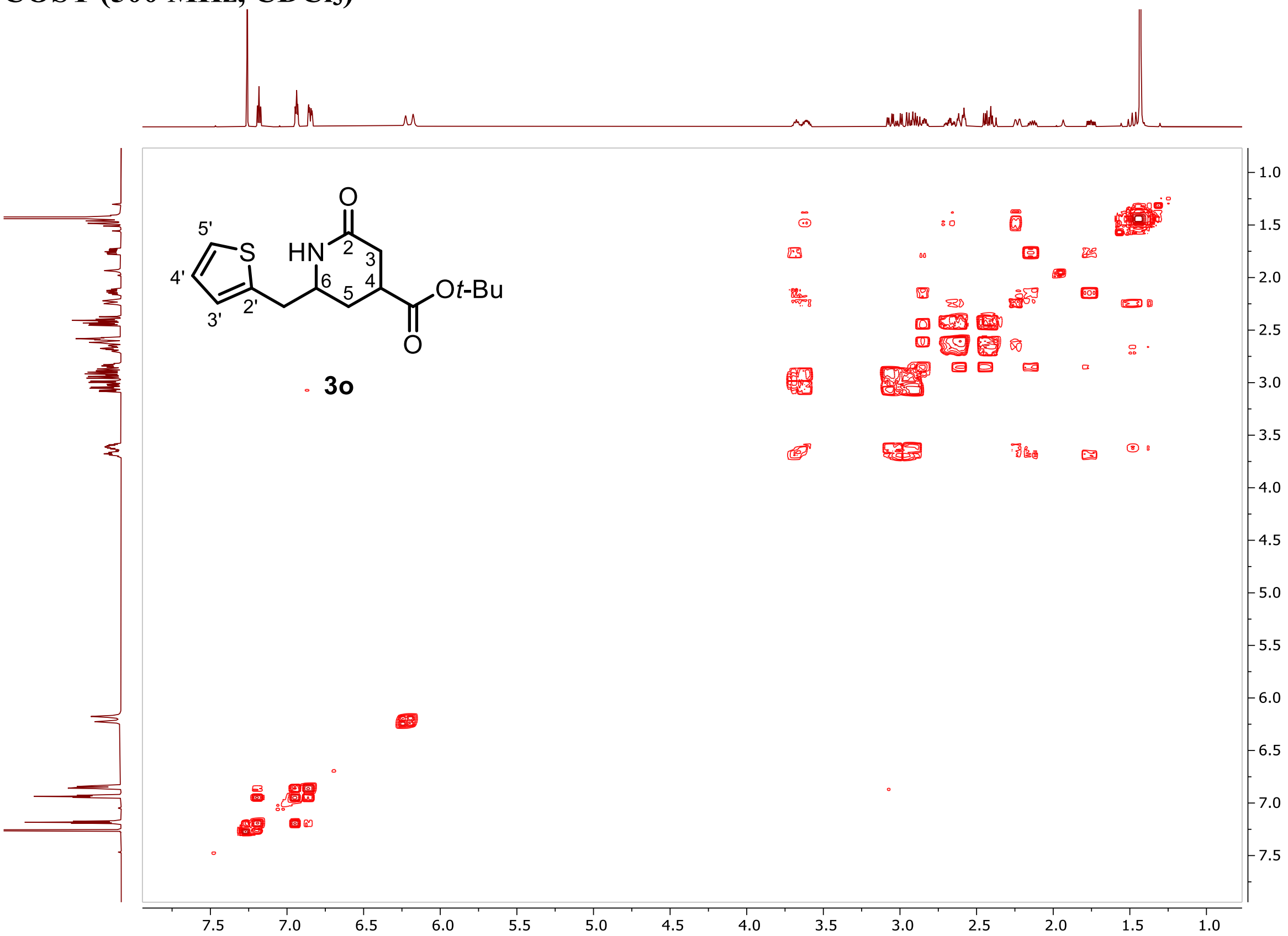


**HSQC ($^1$H 500 MHz/$^{13}$C 126 MHz, CDCl$_3$)**

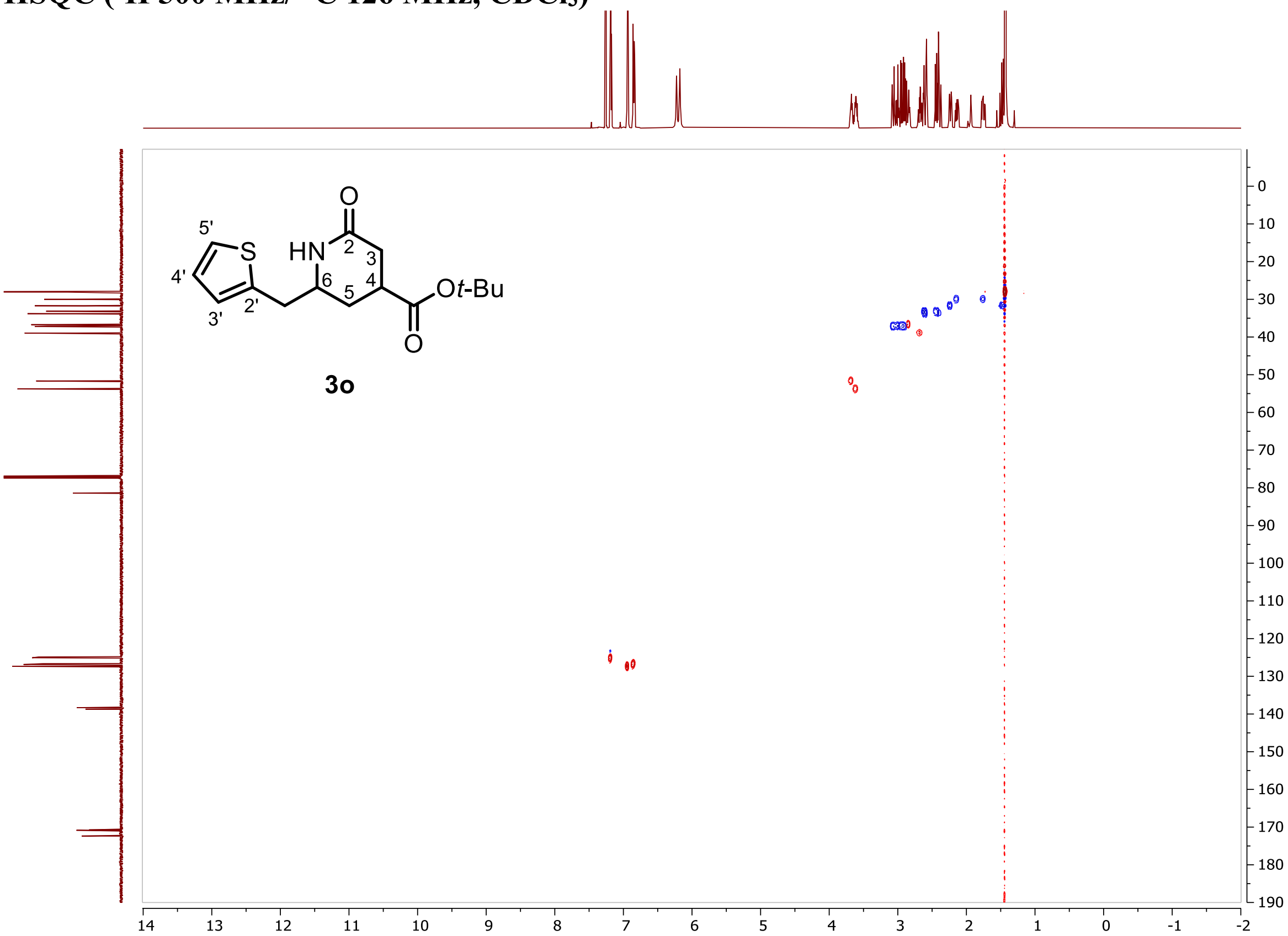

**$^{1}$H NMR (500 MHz, CDCl$_3$)**

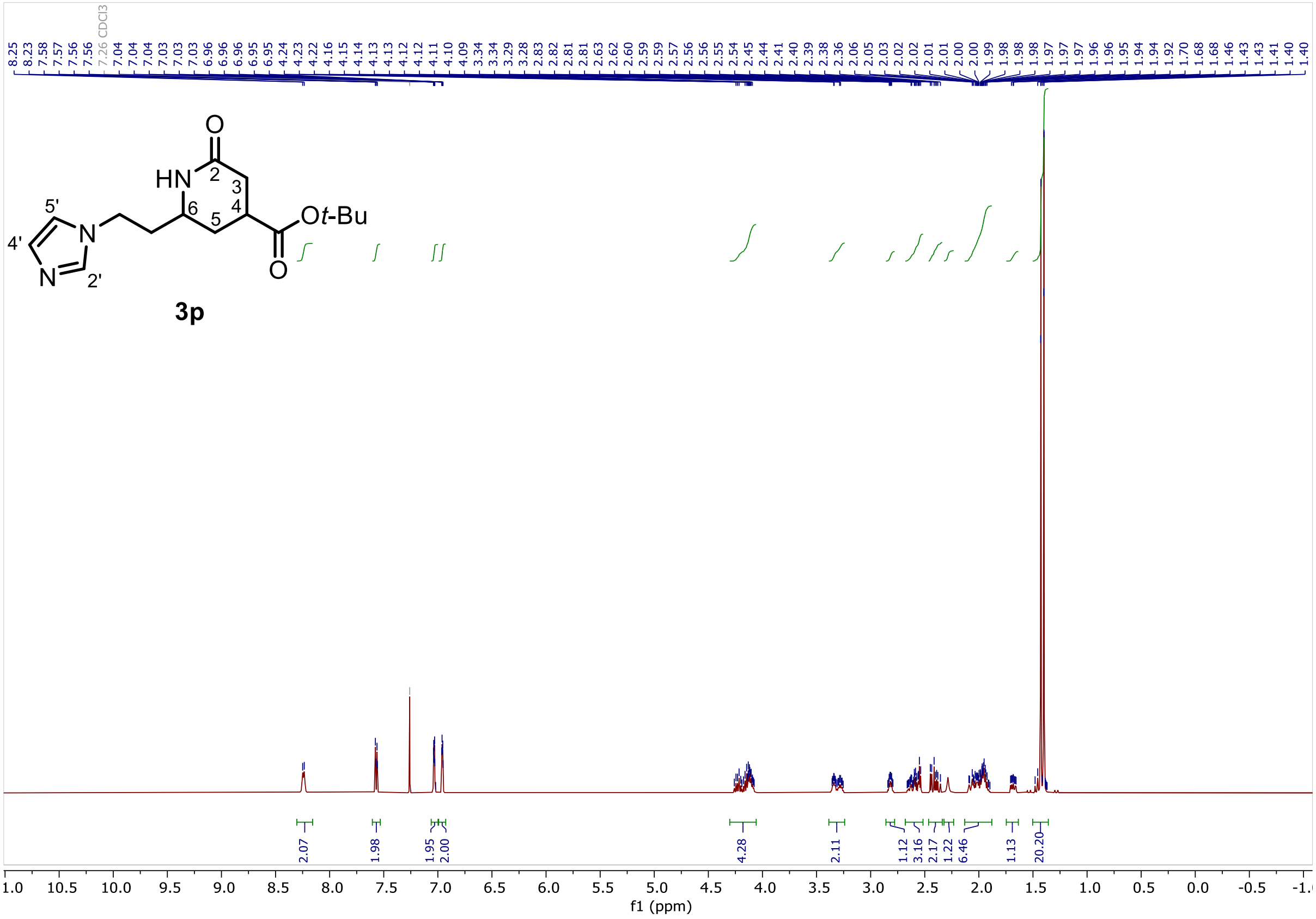

**$^{13}$C NMR (126 MHz, CDCl$_3$)**

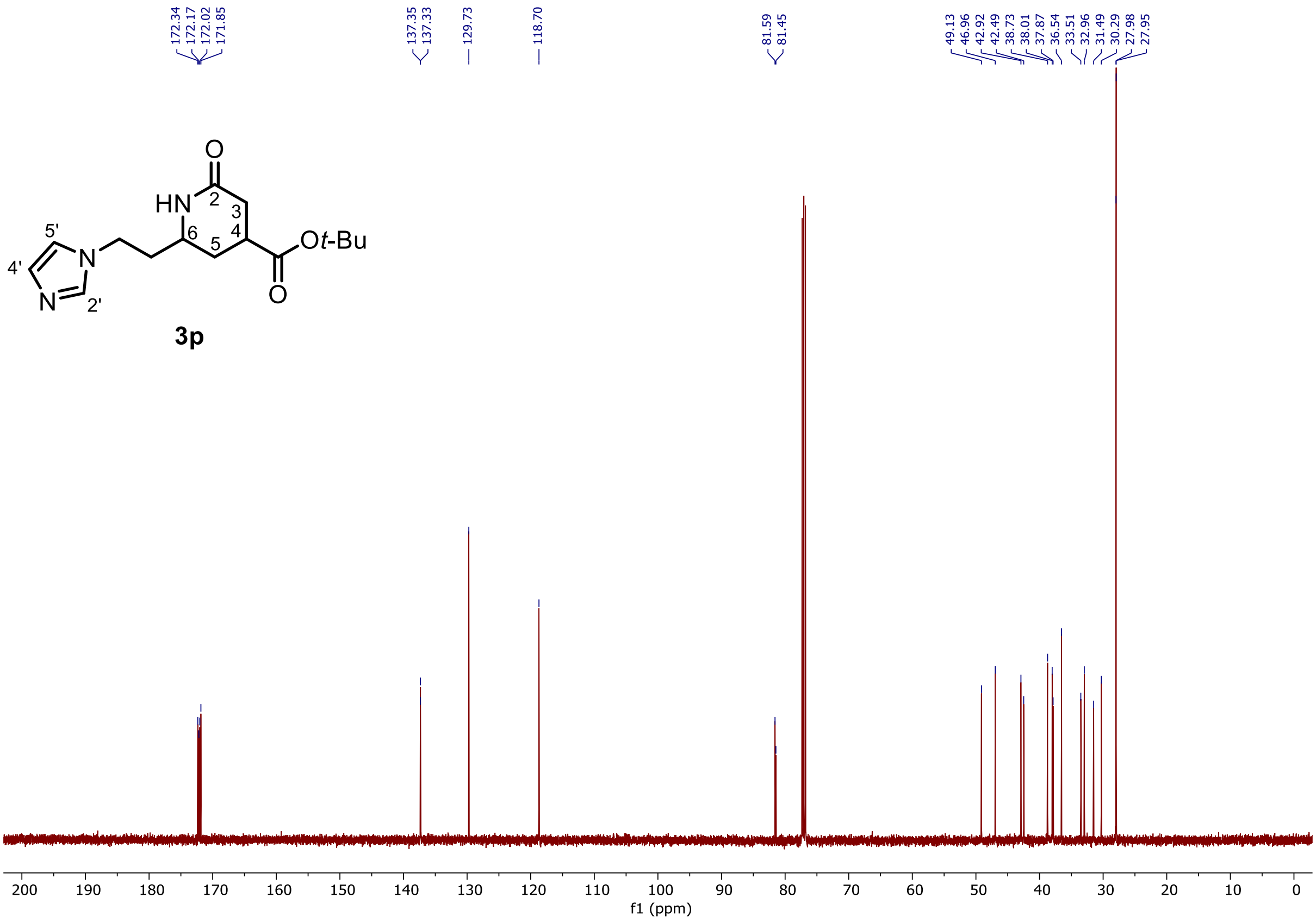

**COSY (500 MHz, $CDCl_3$)**

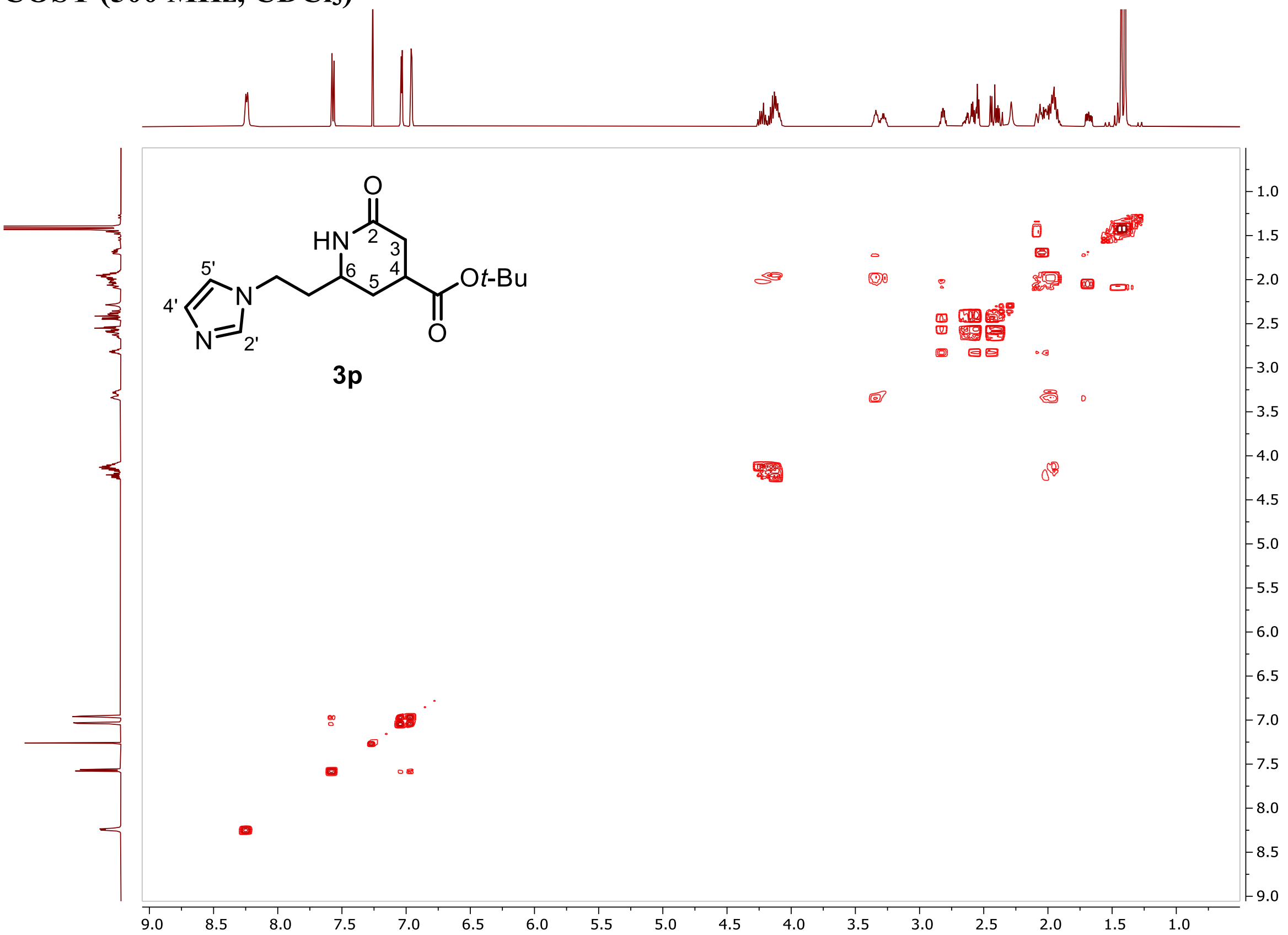


**HSQC ($^1$H 500 MHz/$^{13}$C 126 MHz, $CDCl_3$)**

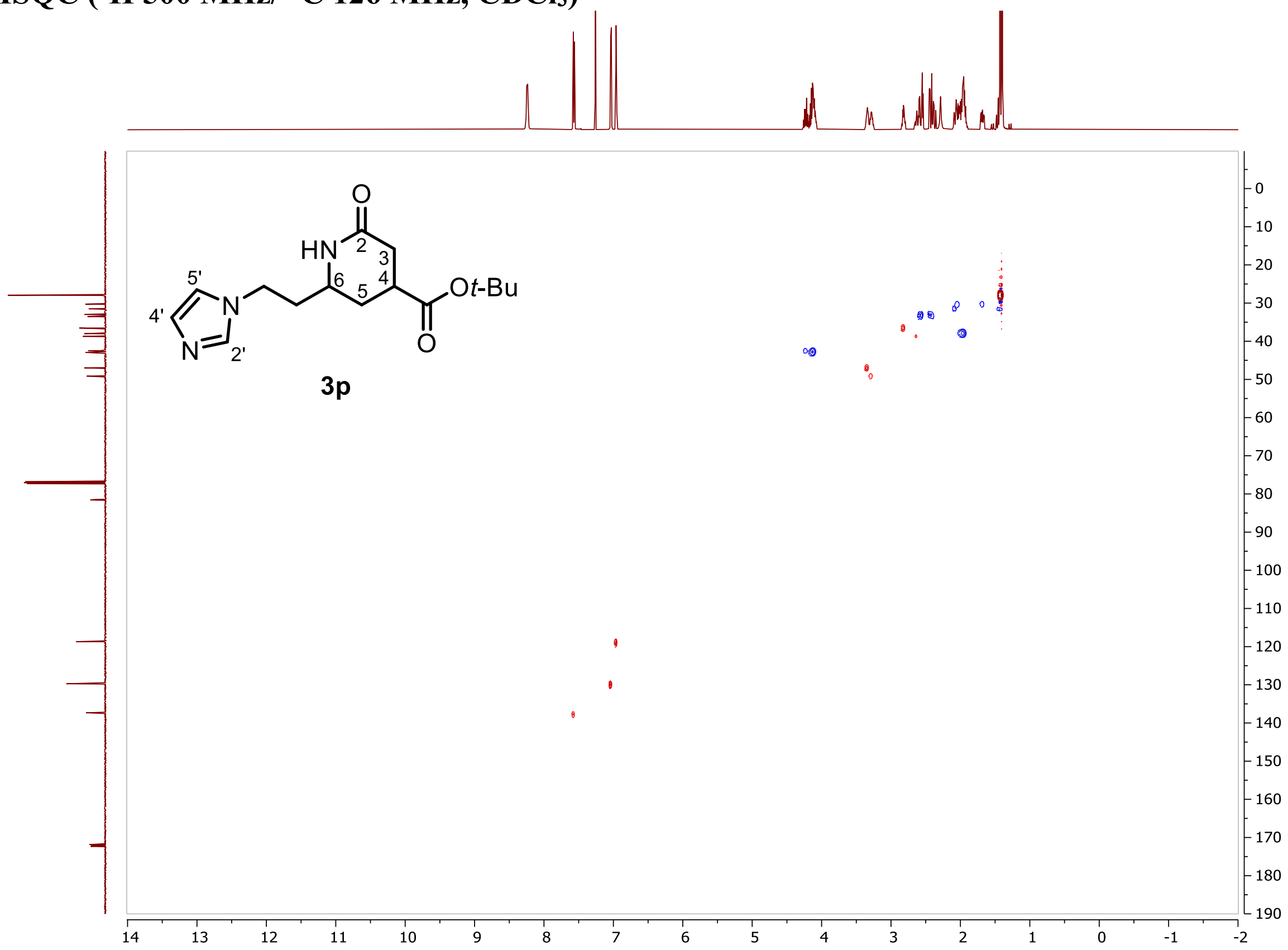

$^{1}$H NMR (500 MHz, $CDCl_3$)

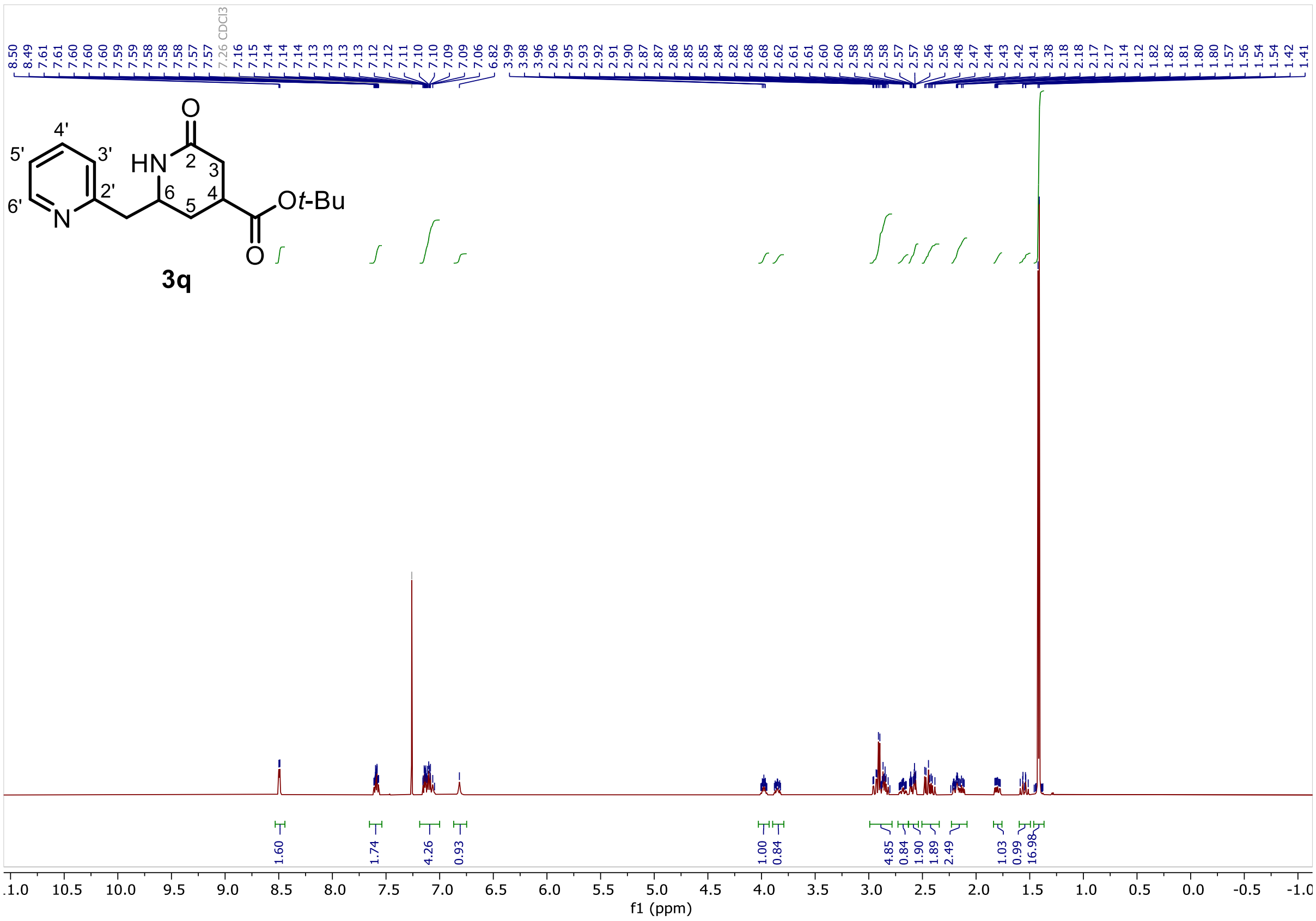

$^{13}$C NMR (126 MHz, $CDCl_3$)

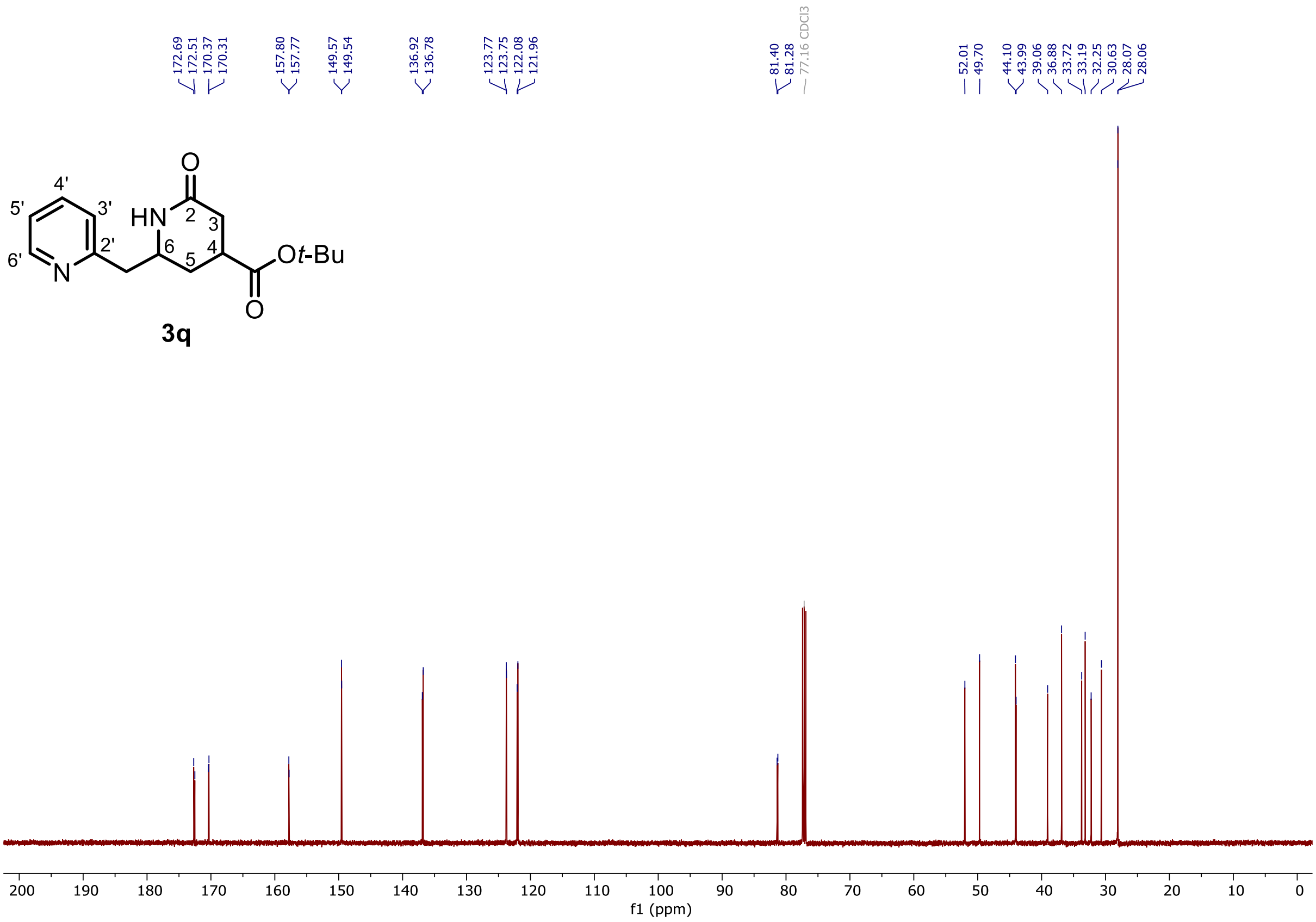

**COSY (500 MHz, $CDCl_3$)**

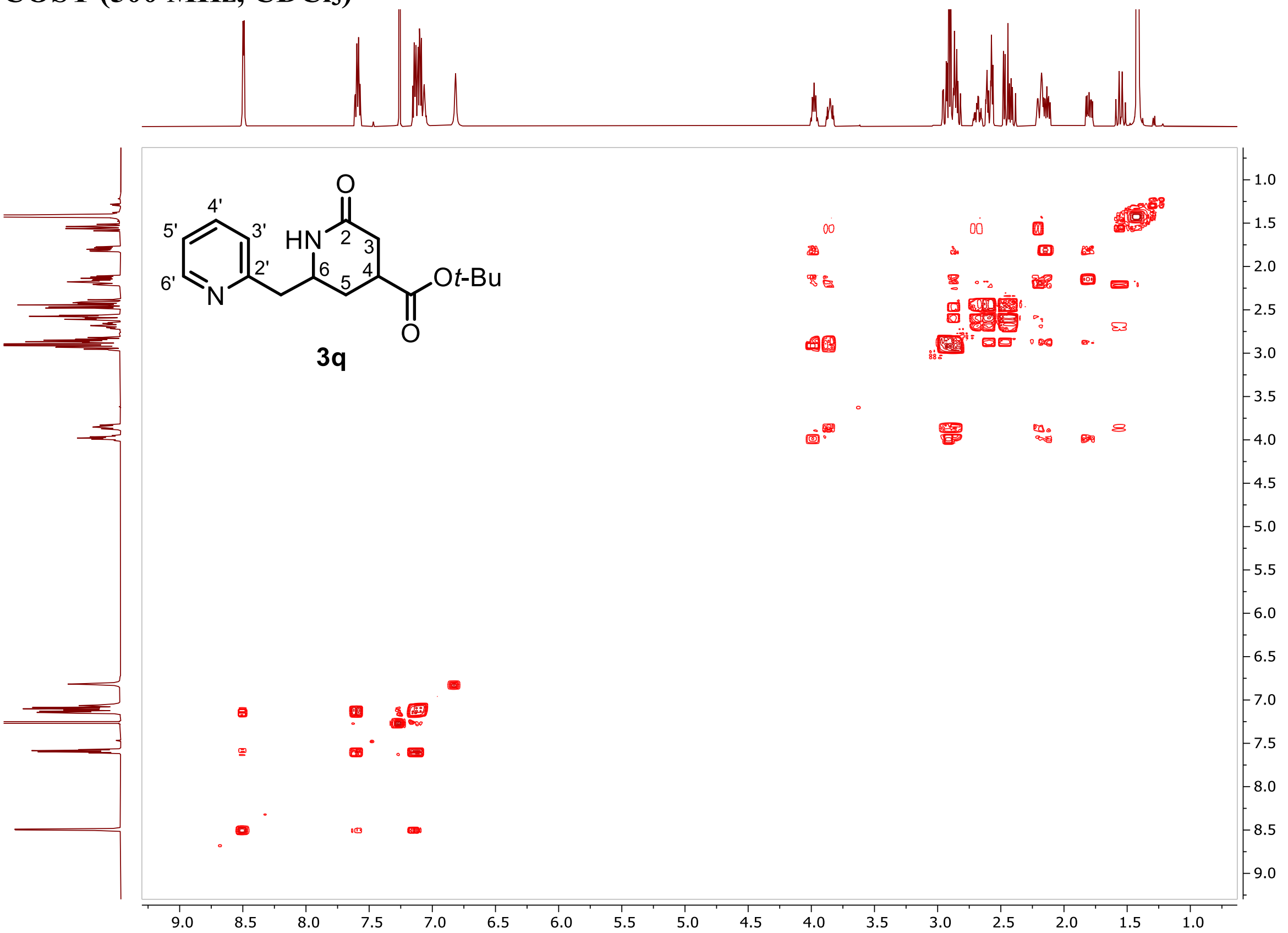


**HSQC ($^1$H 500 MHz/$^{13}$C 126 MHz, $CDCl_3$)**

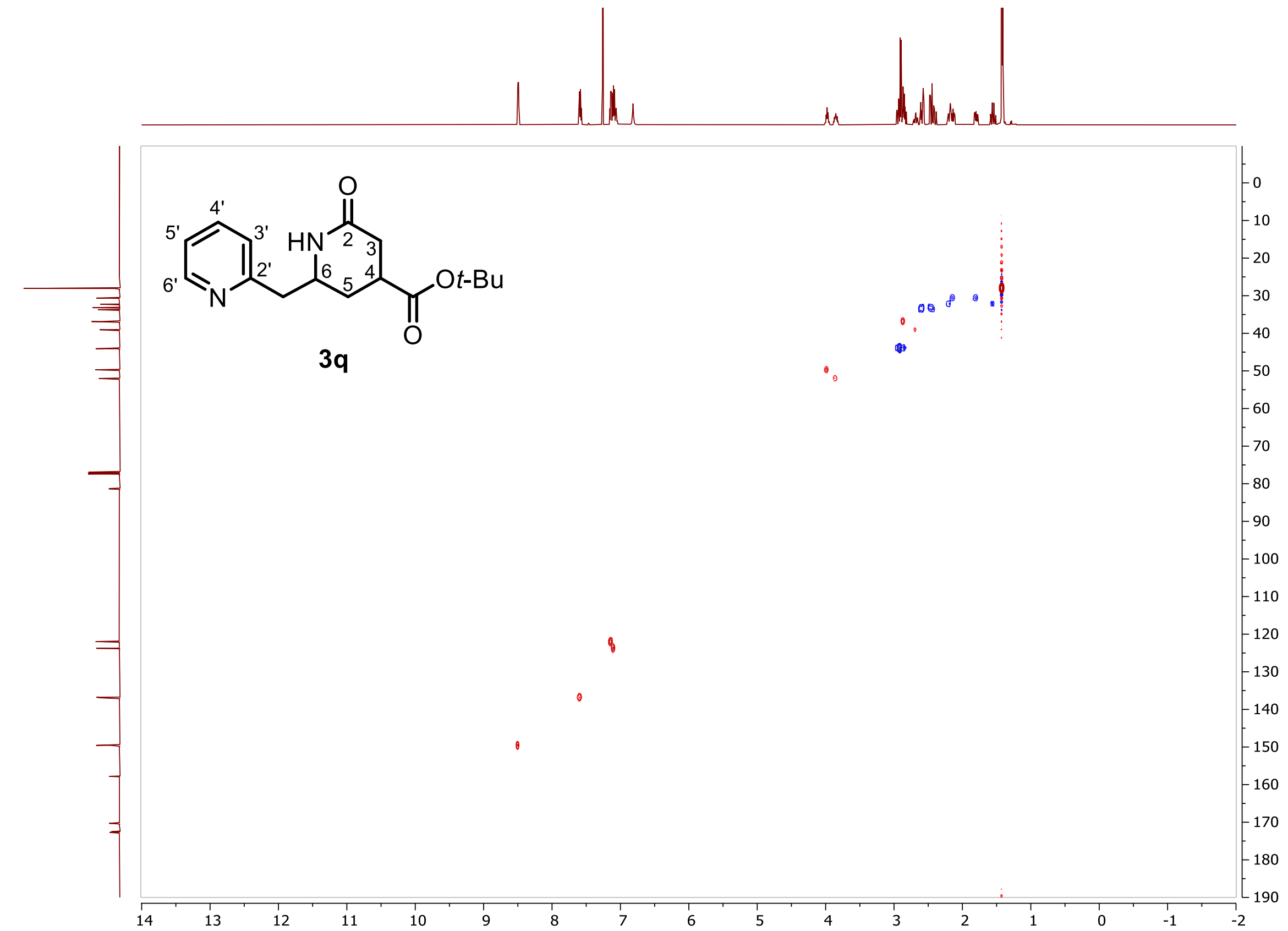

## $^{1}H$ NMR (500 MHz, $CDCl_3$)

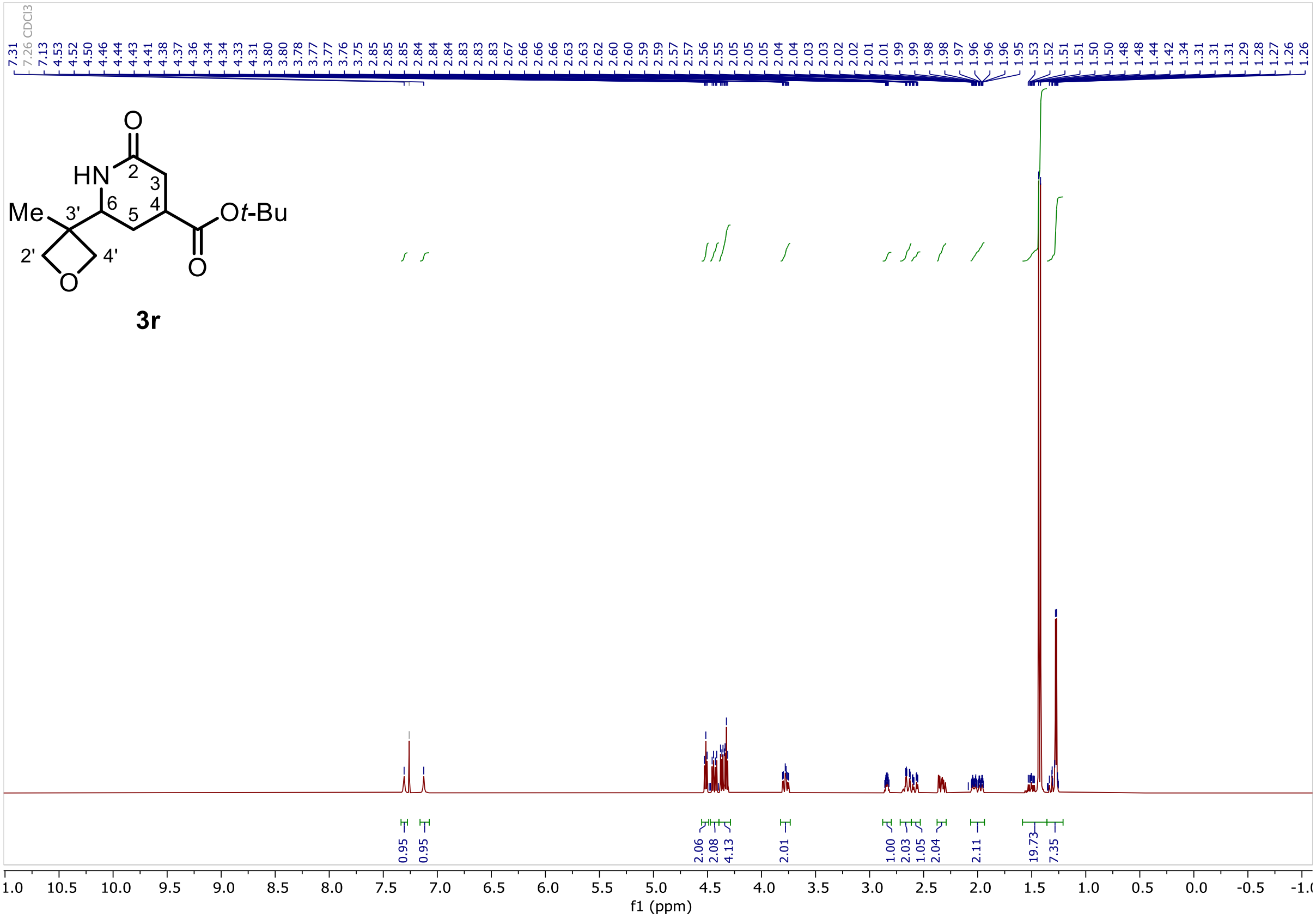


## $^{13}C$ NMR (126 MHz, $CDCl_3$)

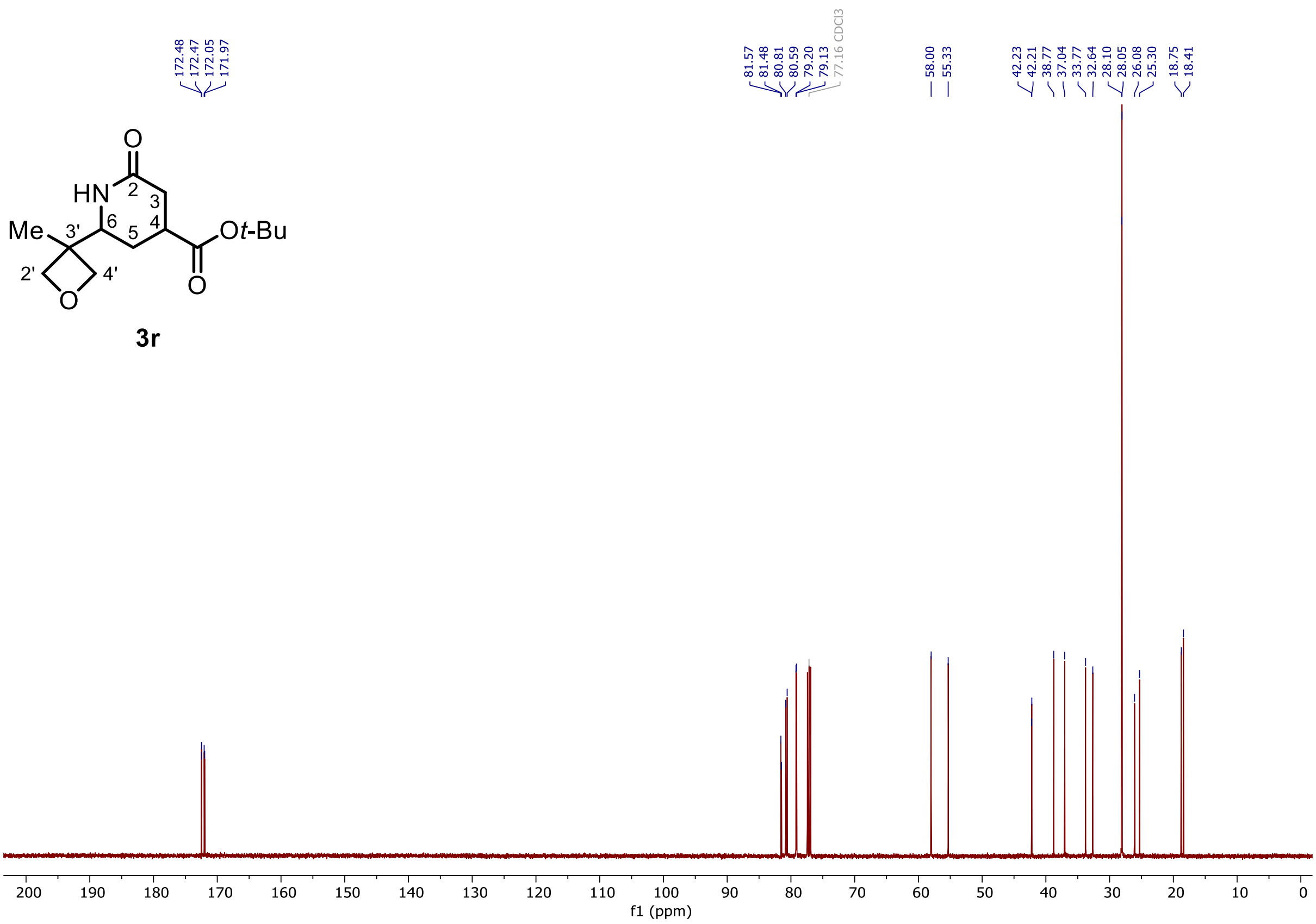

## COSY (500 MHz, $CDCl_3$)

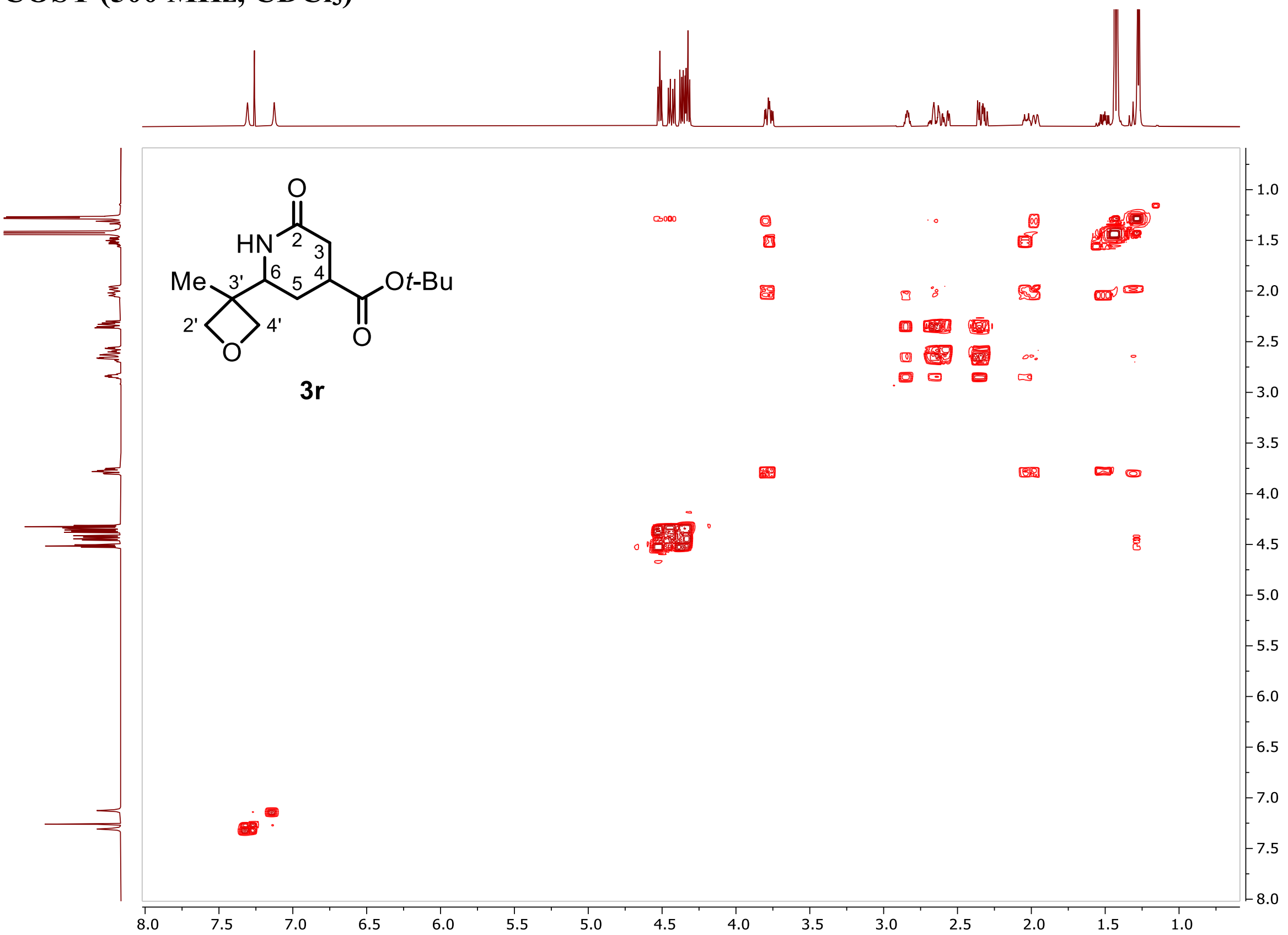


## HSQC ($^1H$ 500 MHz/$^{13}C$ 126 MHz, $CDCl_3$)

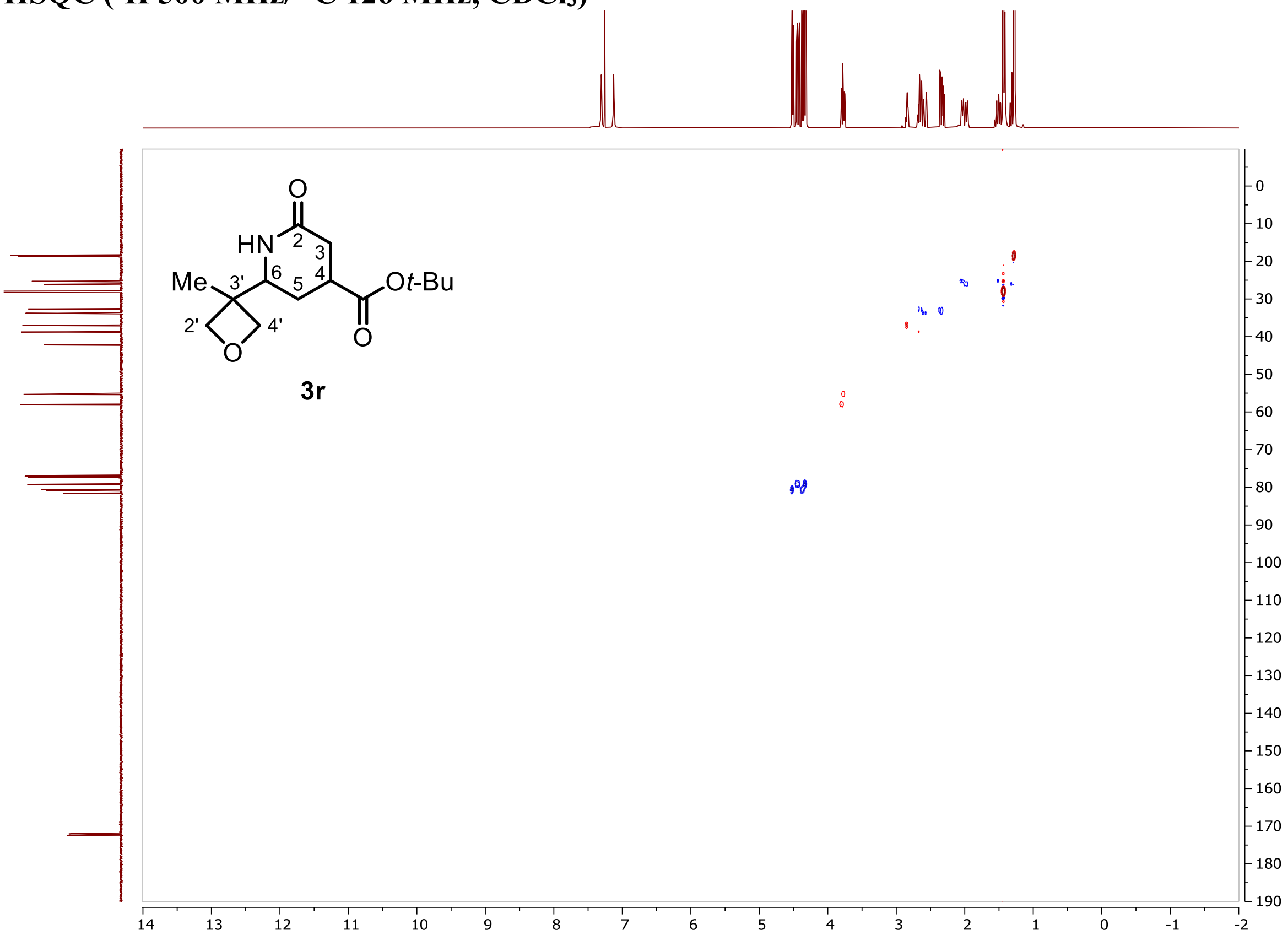

## $^{1}H$ NMR (500 MHz, $CDCl_3$)

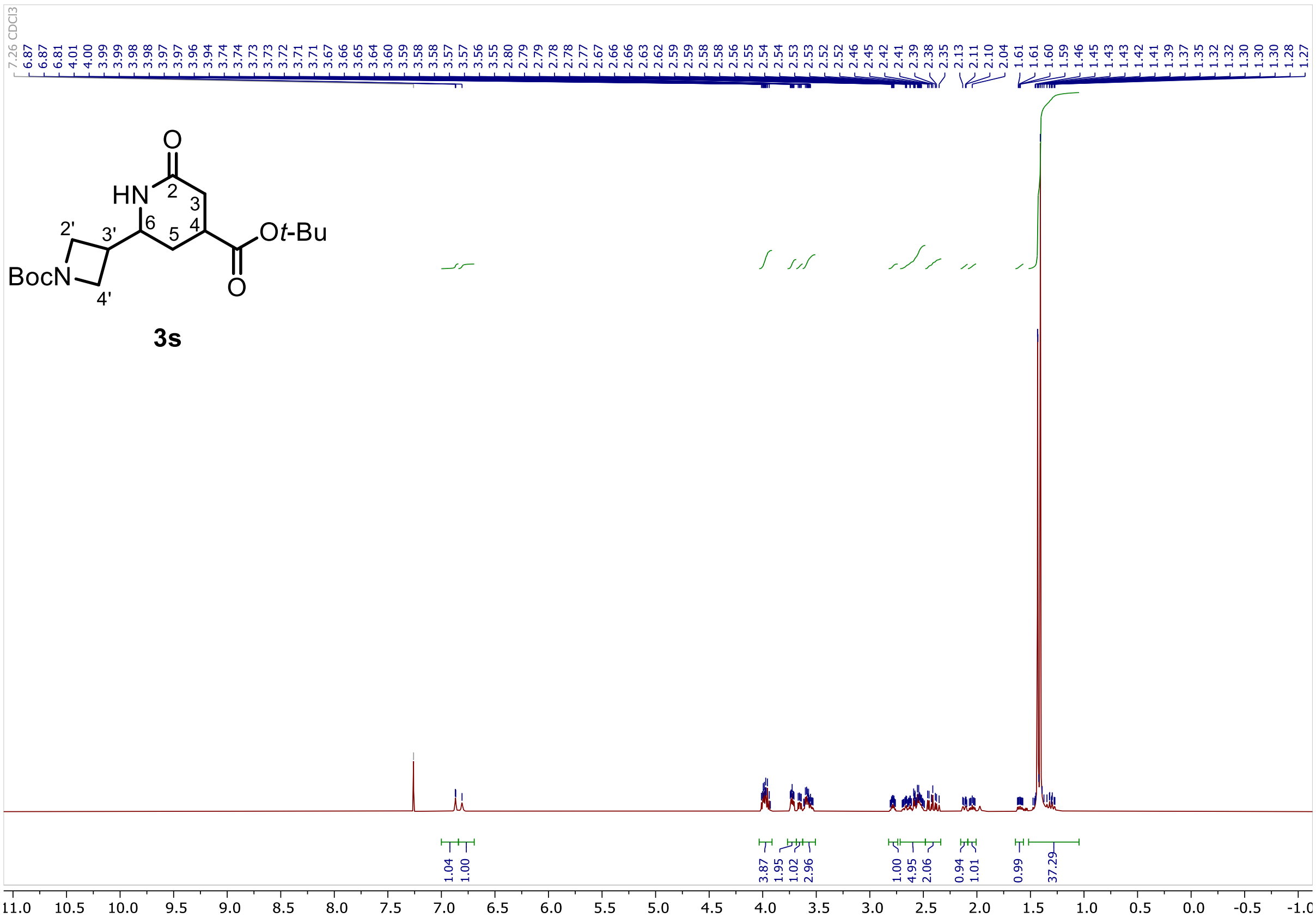


## $^{13}C$ NMR (126 MHz, $CDCl_3$)

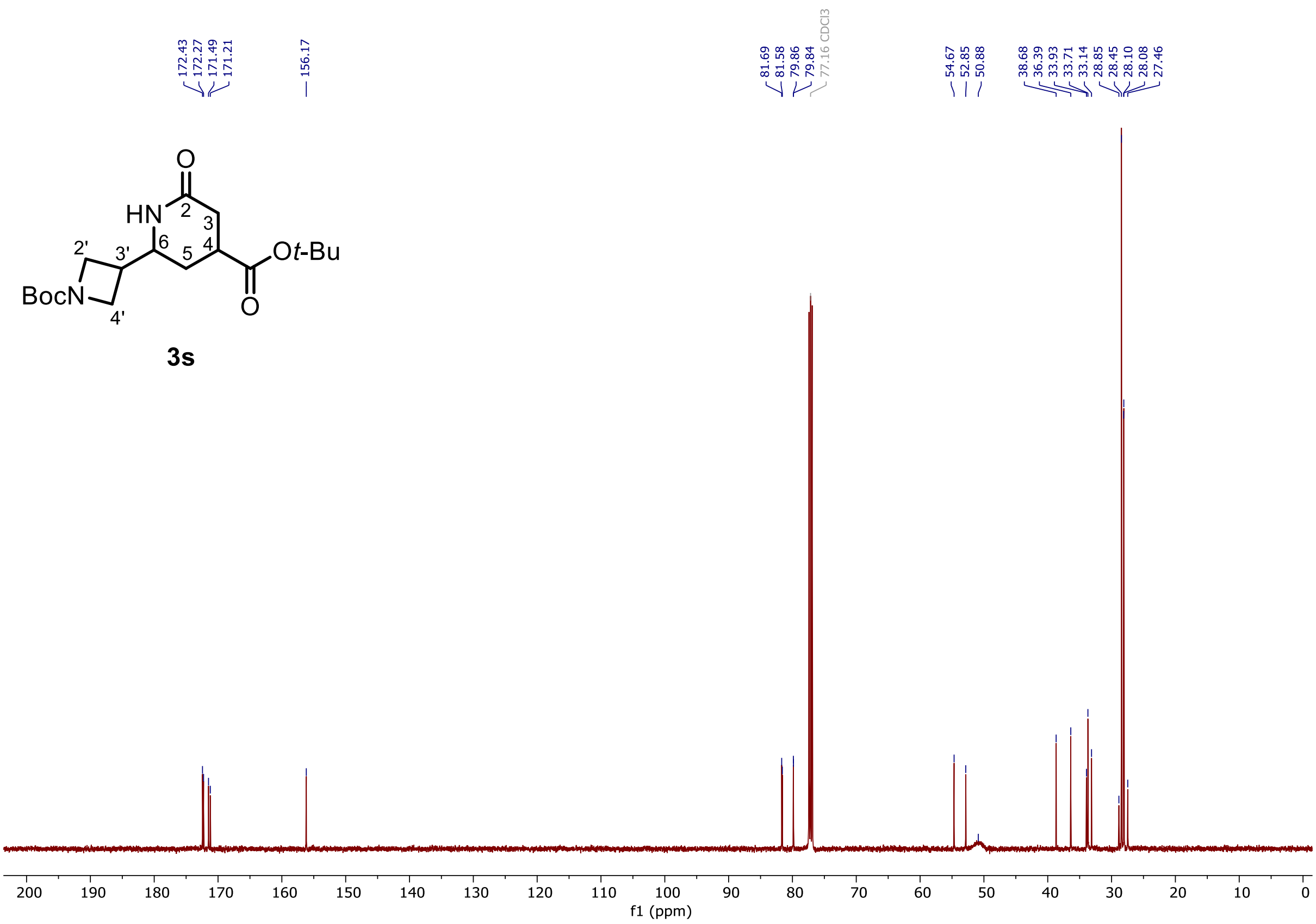

**COSY (500 MHz, CDCl$_3$)**

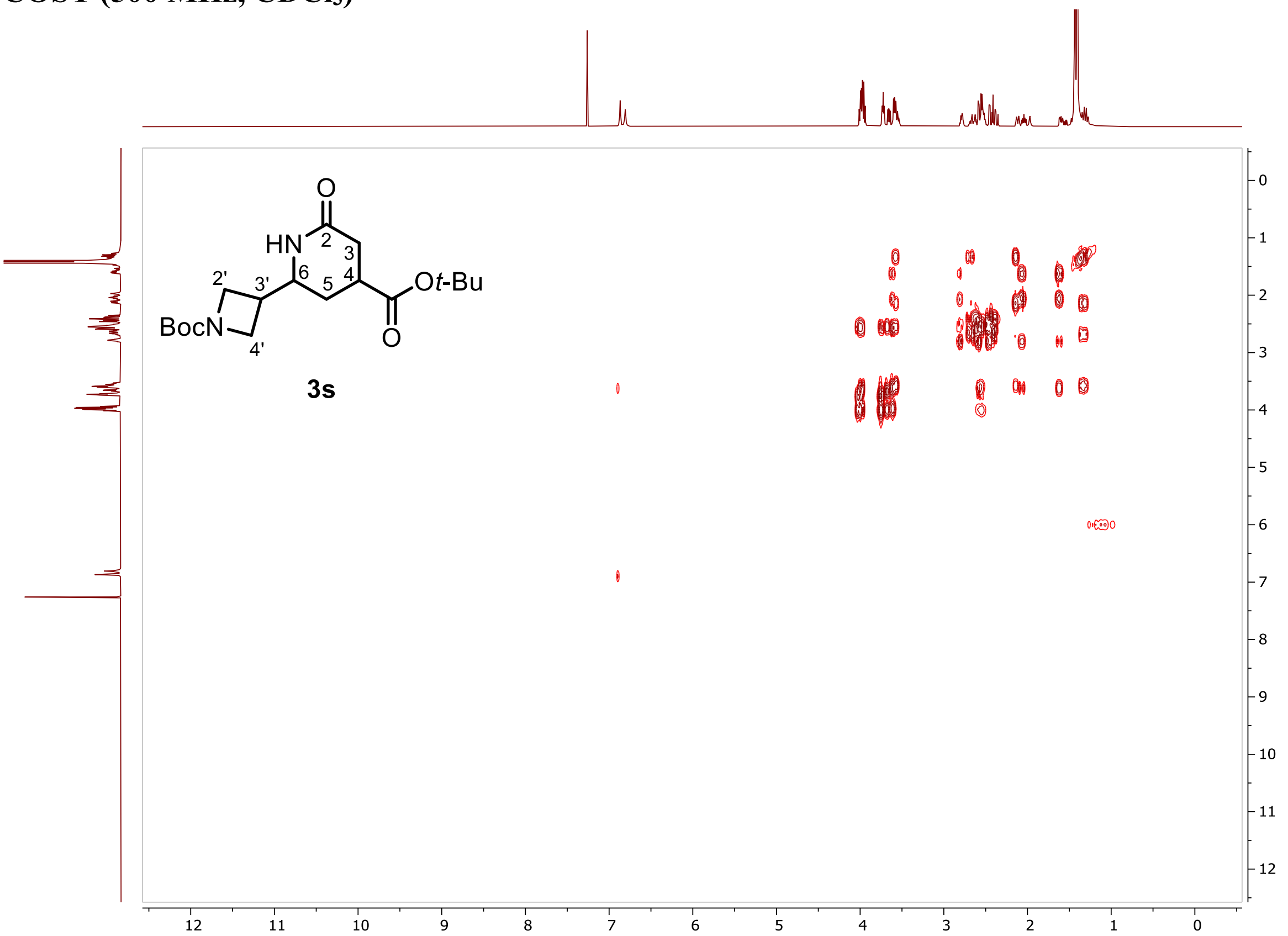


**HSQC ($^1$H 500 MHz/$^{13}$C 126 MHz, CDCl$_3$)**

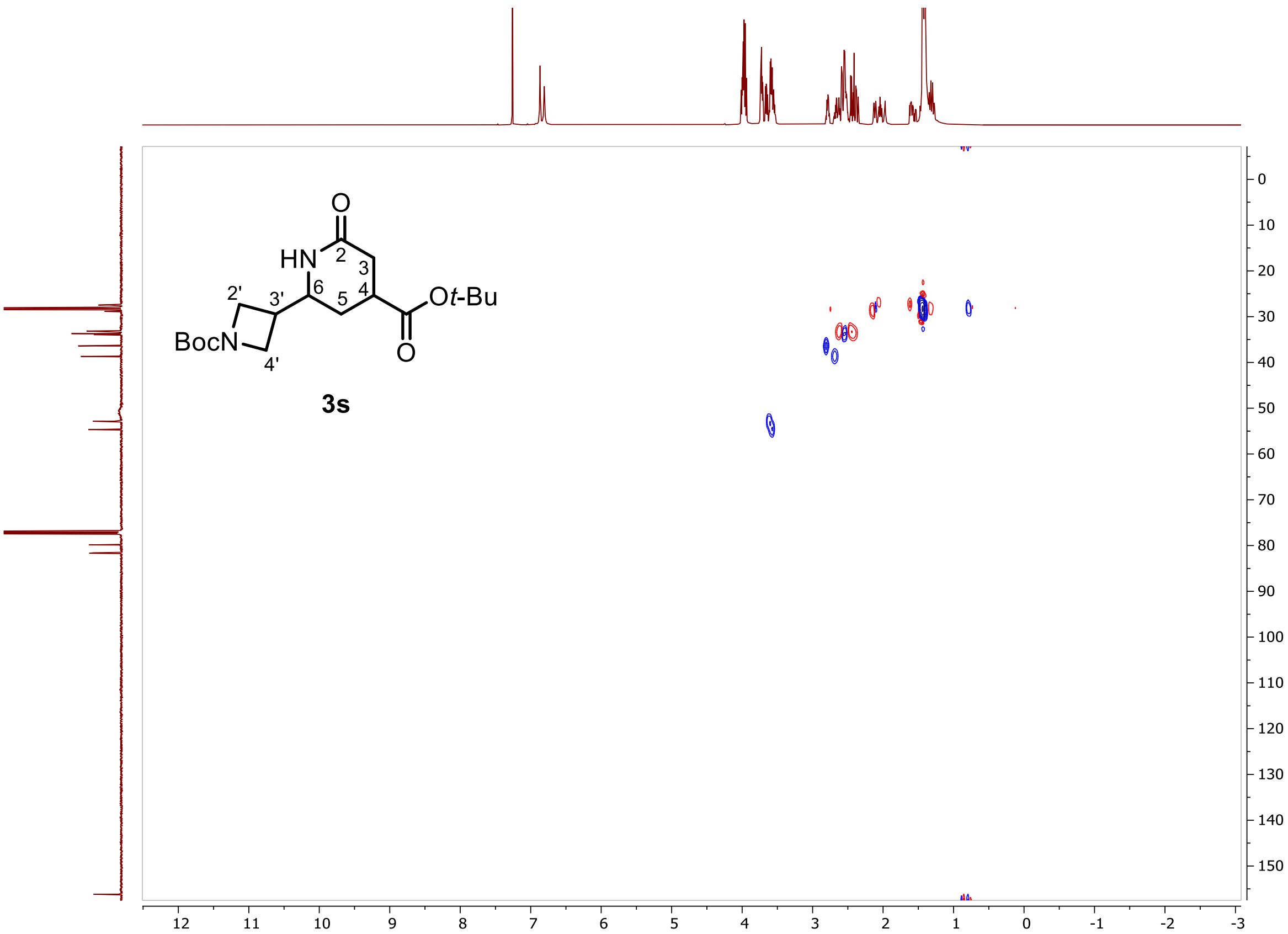

**$^{1}H$ NMR (500 MHz, $CDCl_3$)**

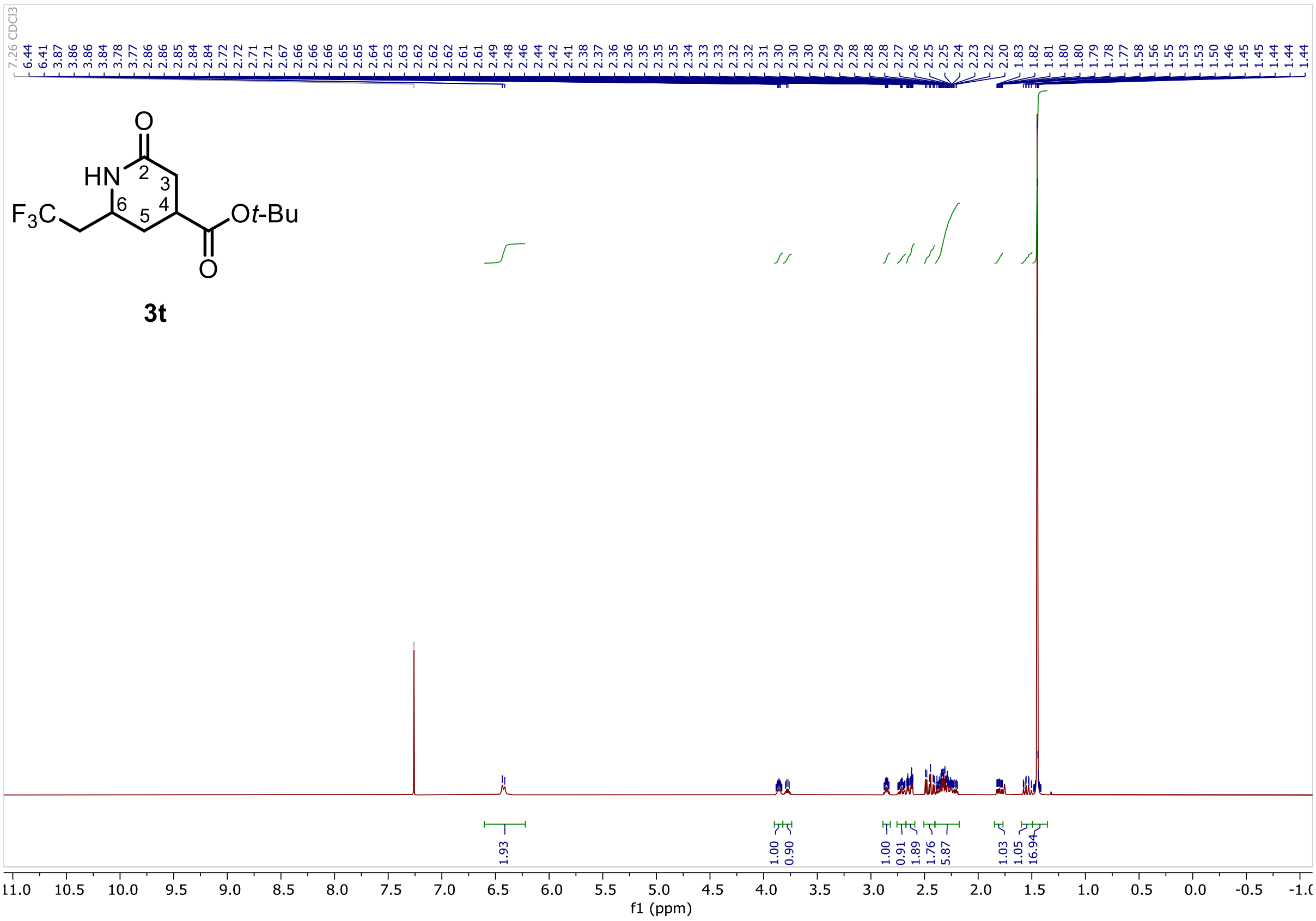


**$^{13}C$ NMR (126 MHz, $CDCl_3$)**

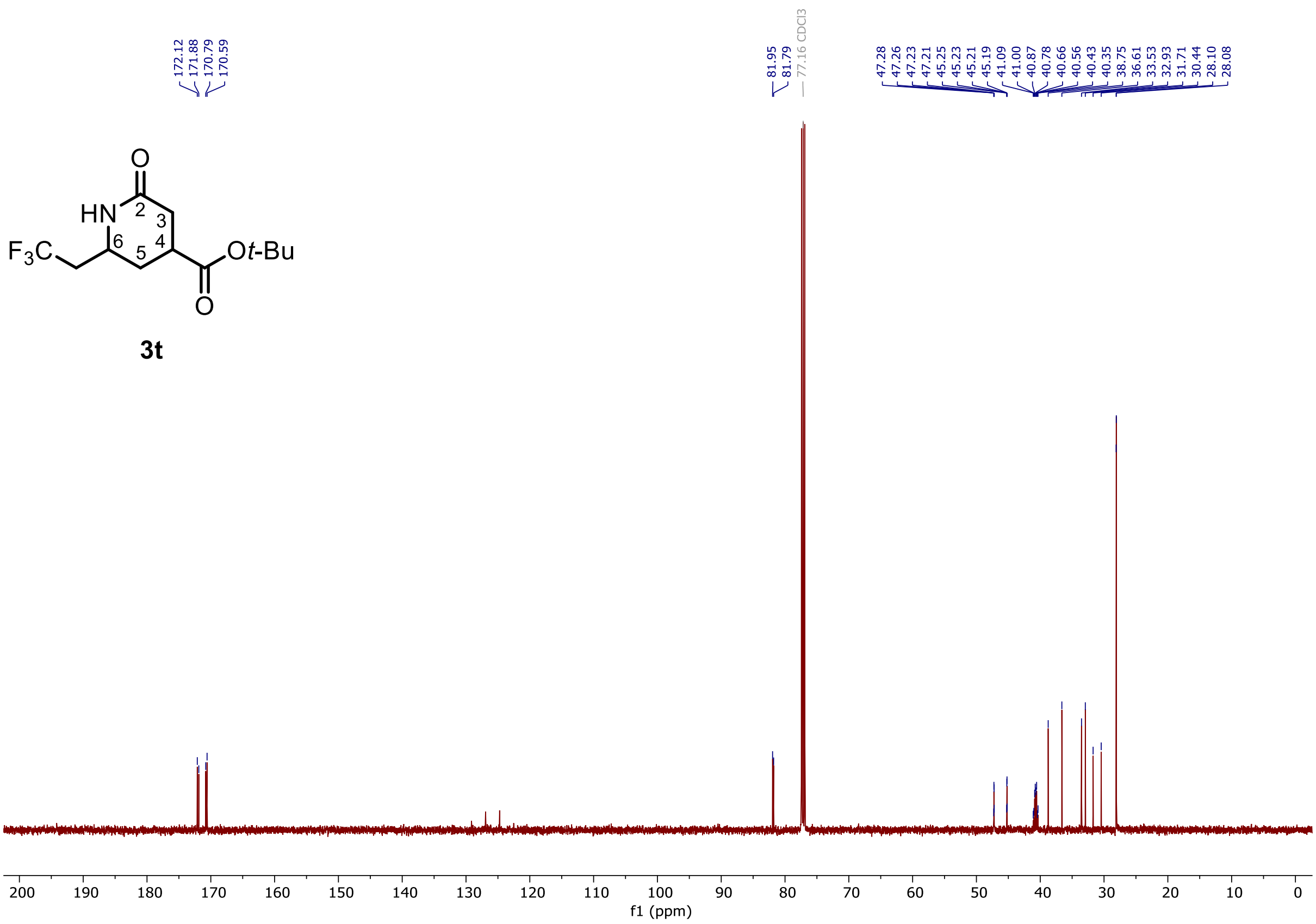

**COSY (500 MHz, $CDCl_3$)**

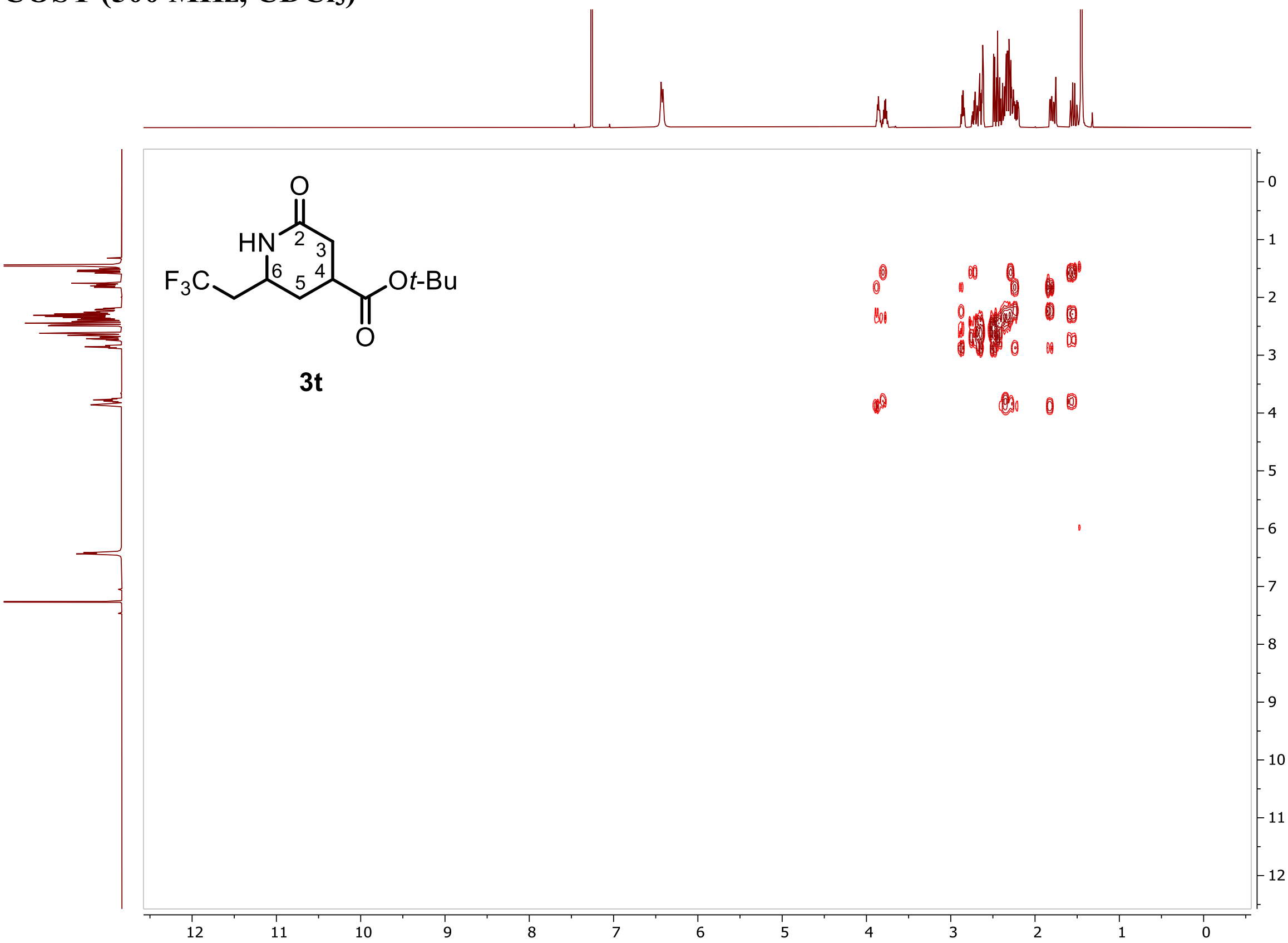


**HSQC ($^1H$ 500 MHz/$^{13}C$ 126 MHz, $CDCl_3$)**

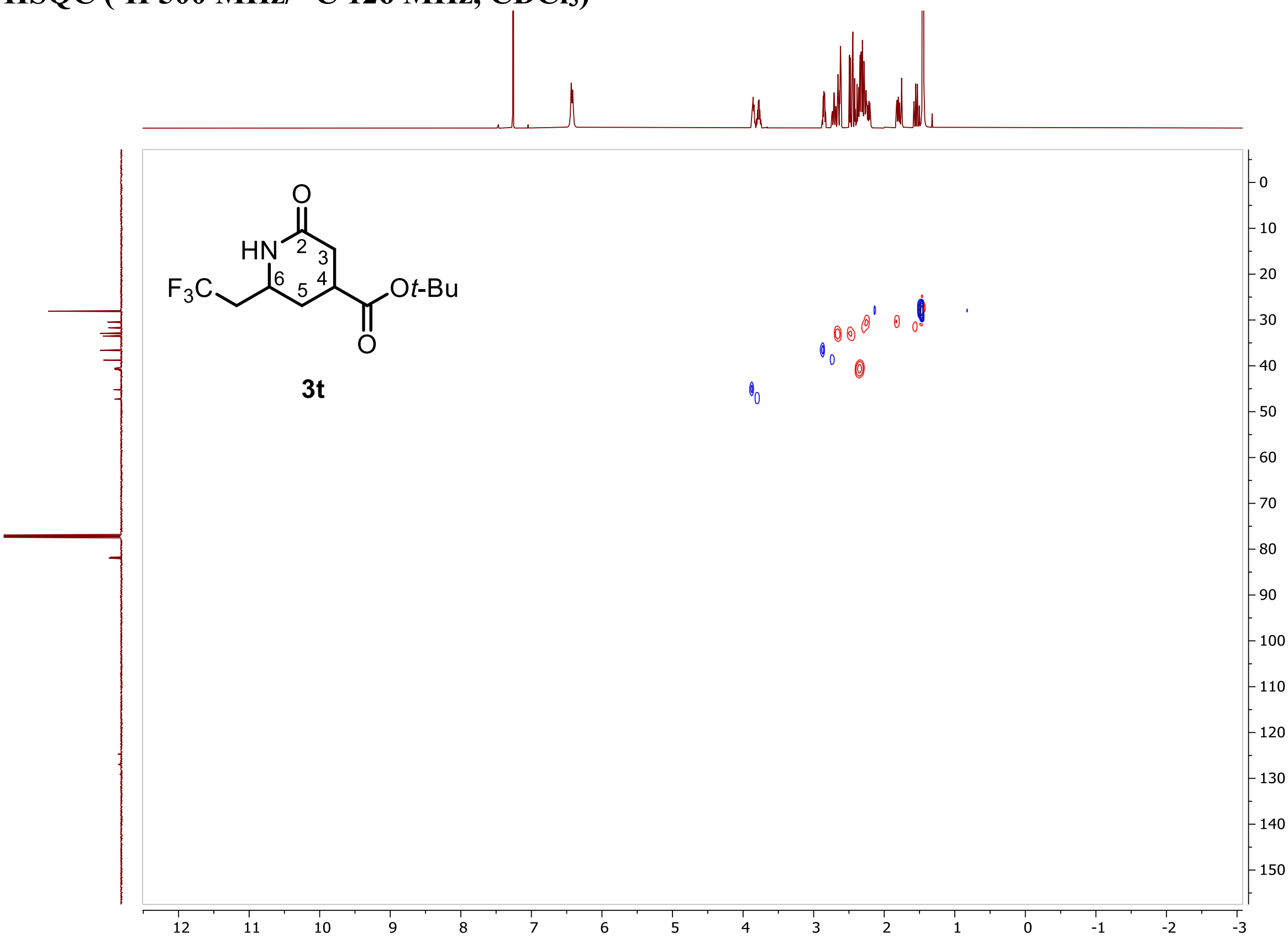

**$^1$H NMR (500 MHz, CDCl$_3$)**

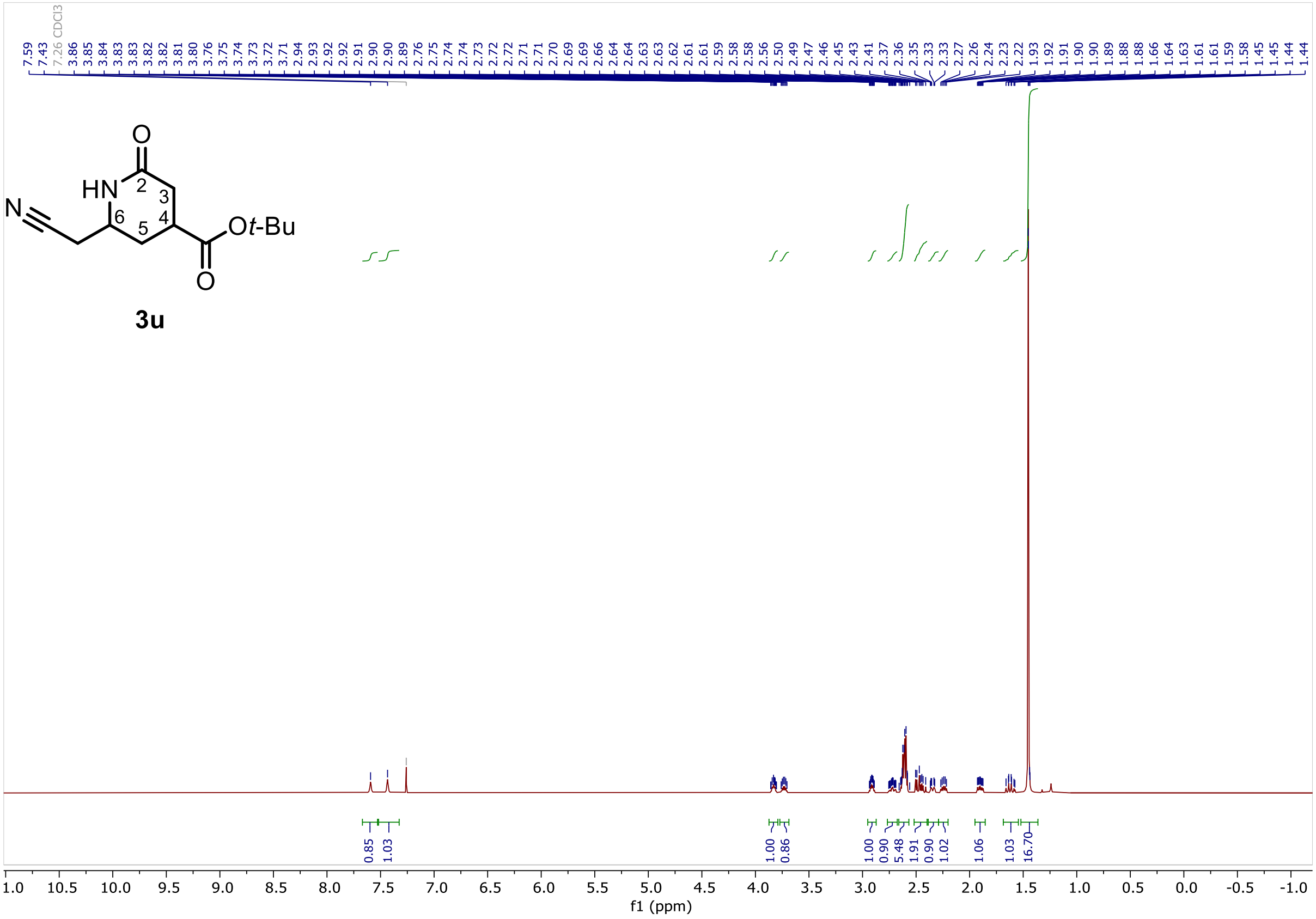

**$^{13}$C NMR (126 MHz, CDCl$_3$)**

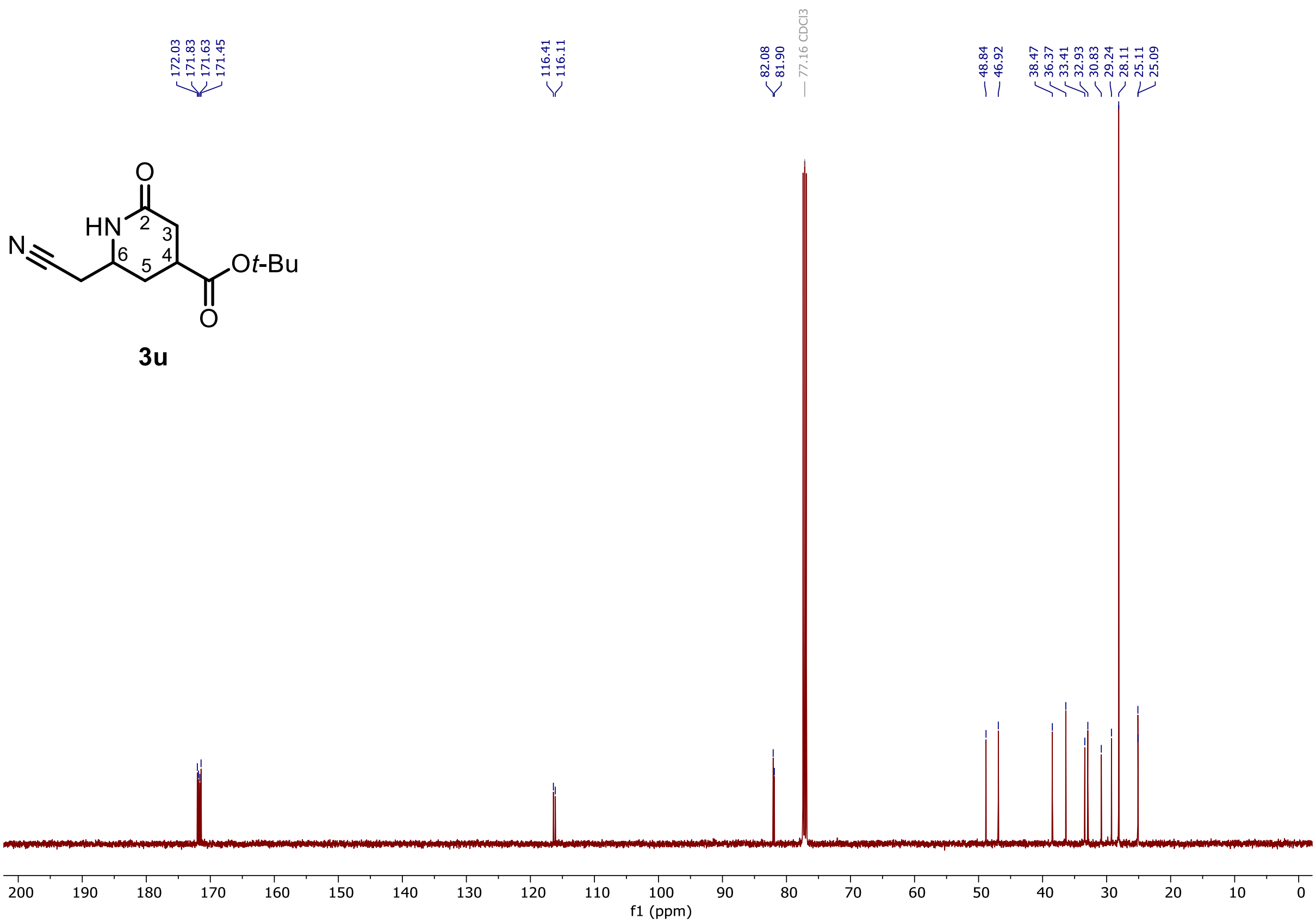

**COSY (500 MHz, $CDCl_3$)**

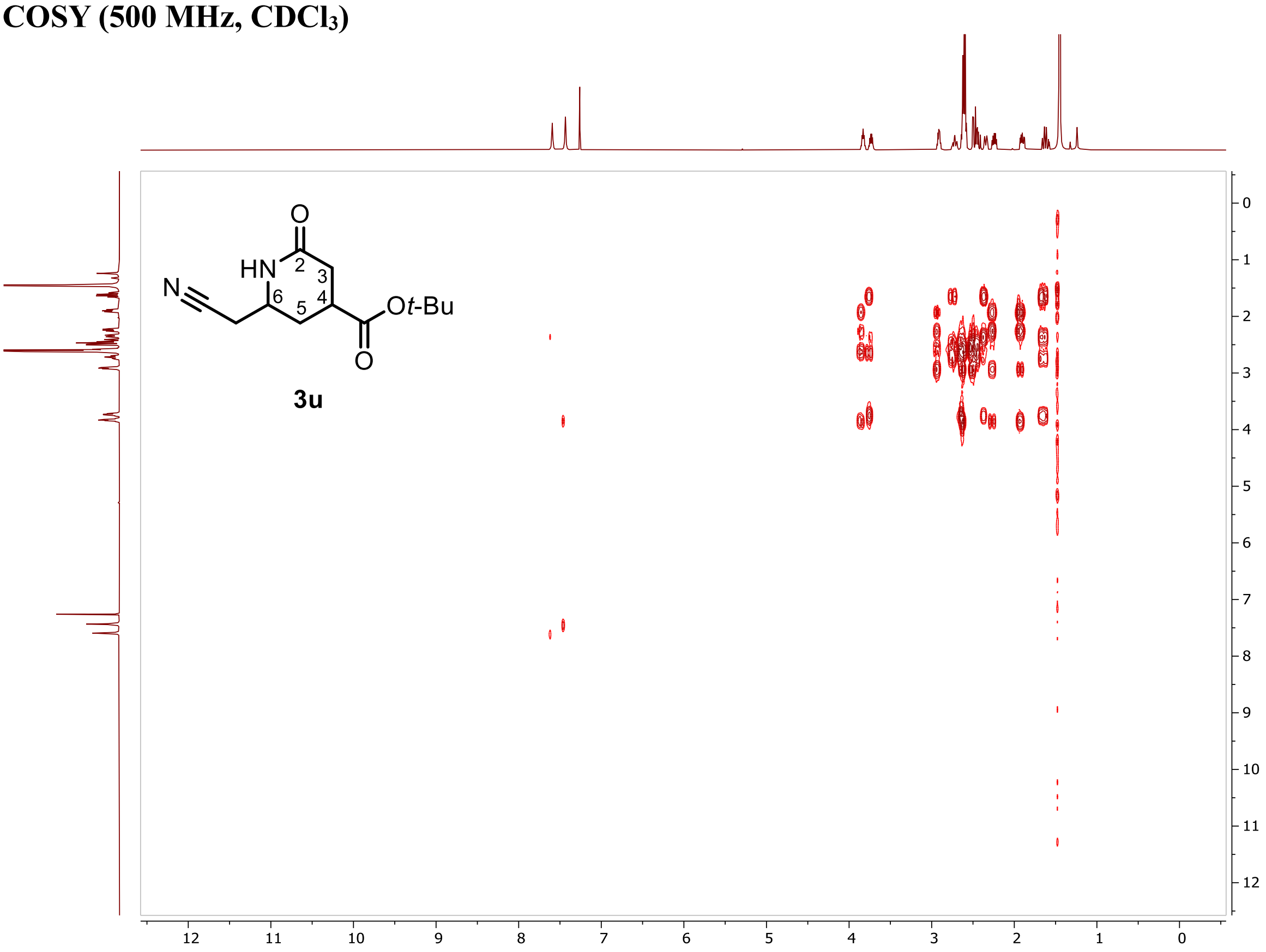


**HSQC ($^{1}H$ 500 MHz/$^{13}C$ 126 MHz, $CDCl_3$)**

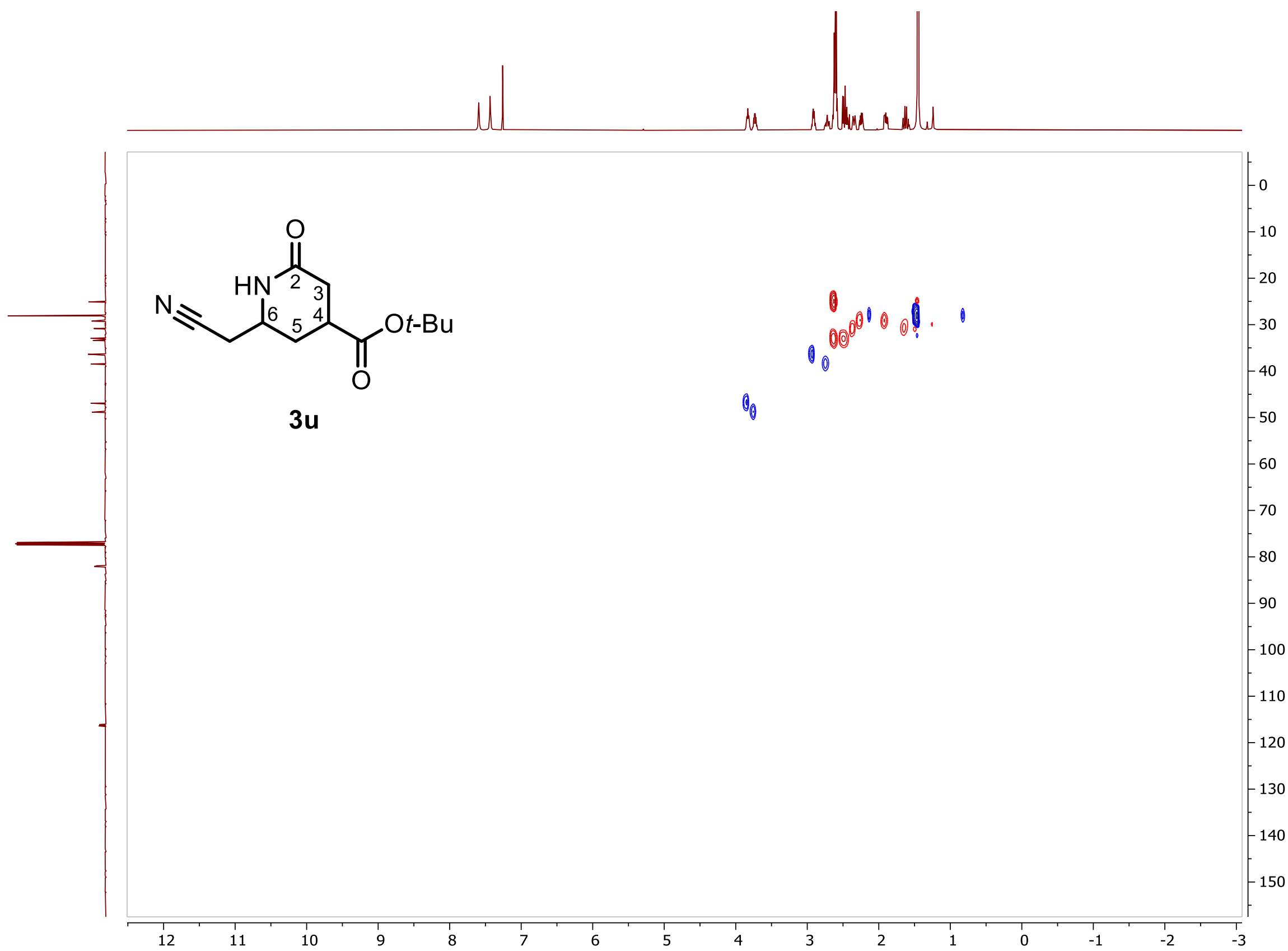

## $^{1}$H NMR (500 MHz, $CDCl_3$)

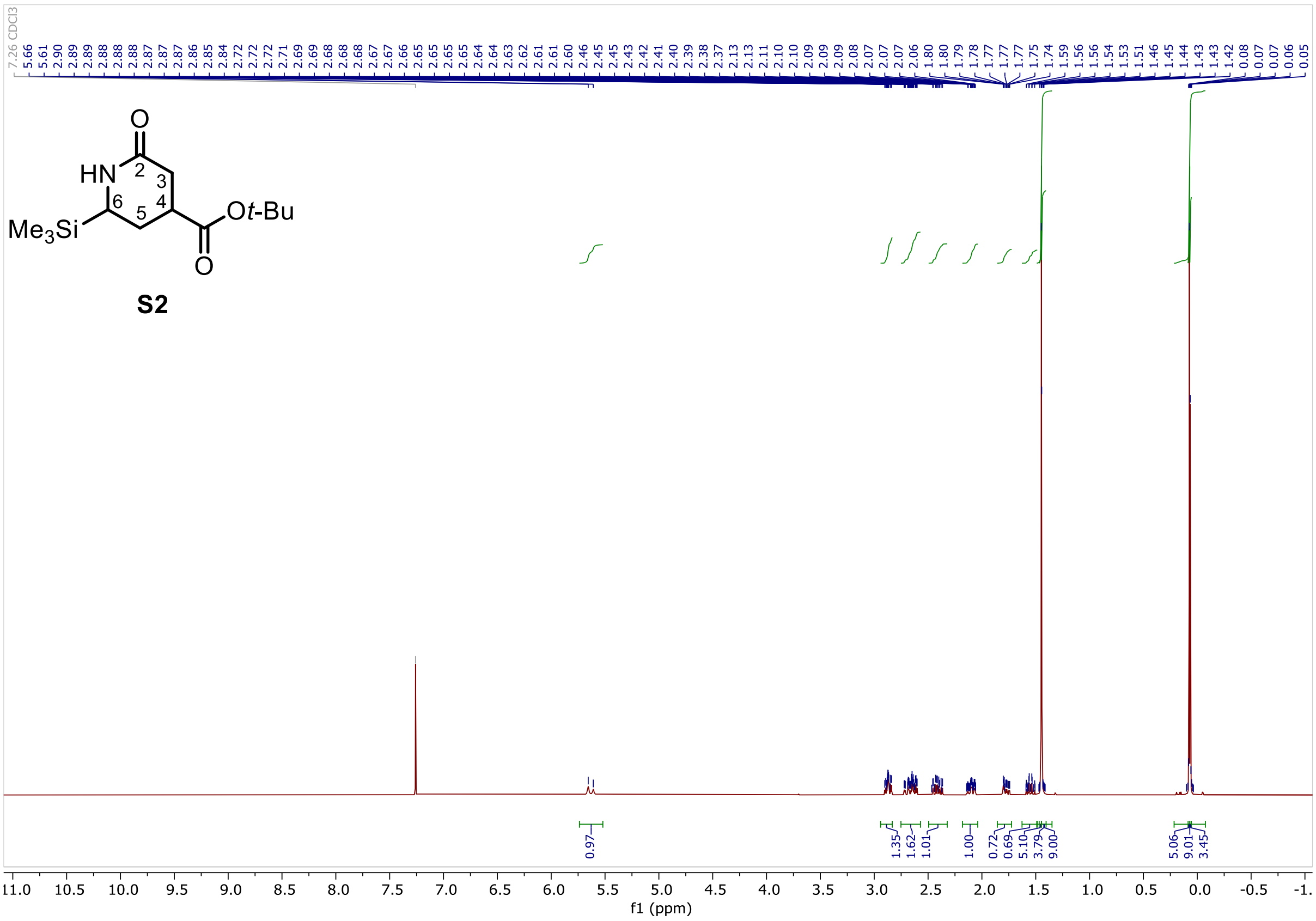


## $^{13}$C NMR (126 MHz, $CDCl_3$)

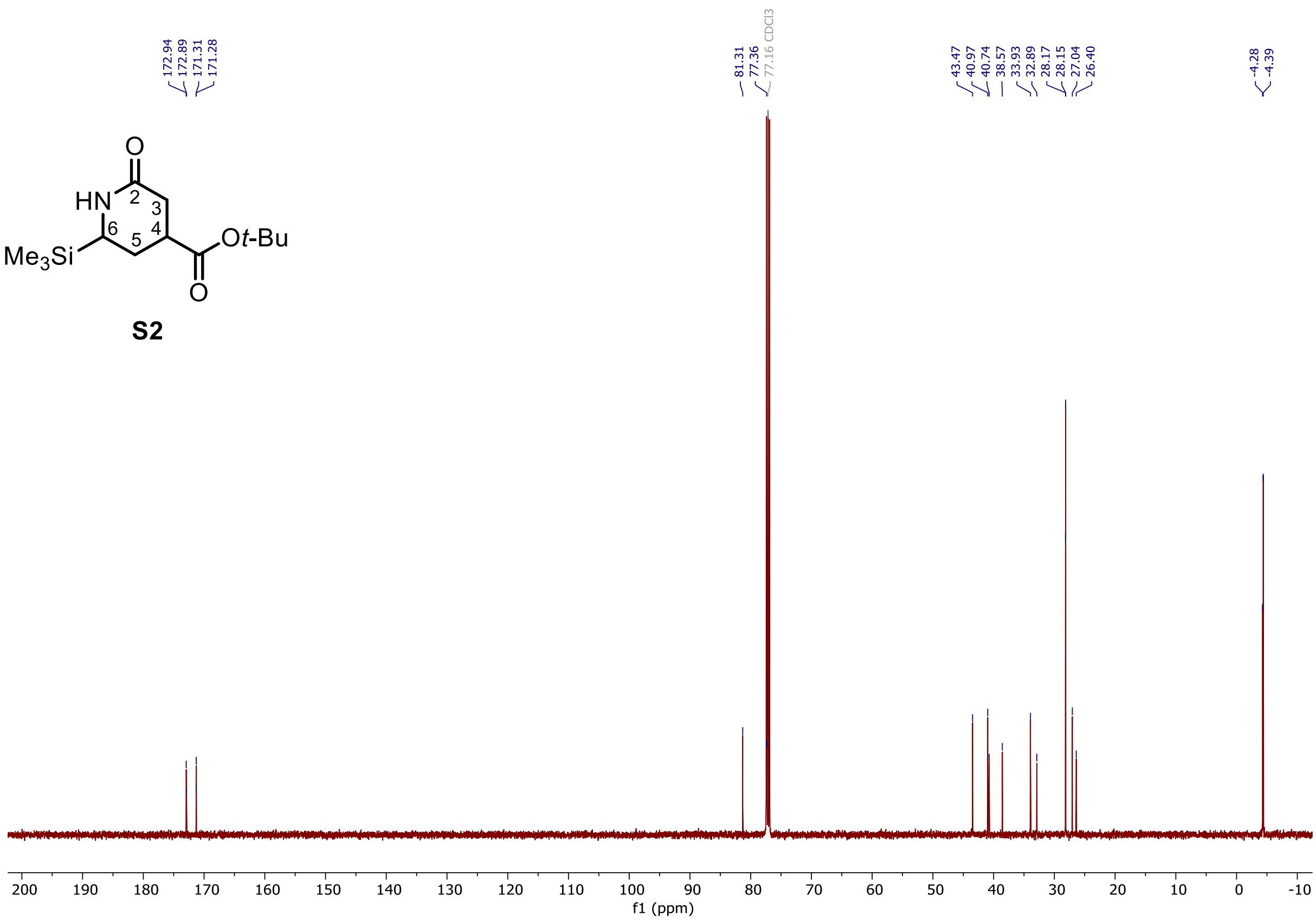

**COSY (500 MHz, $CDCl_3$)**

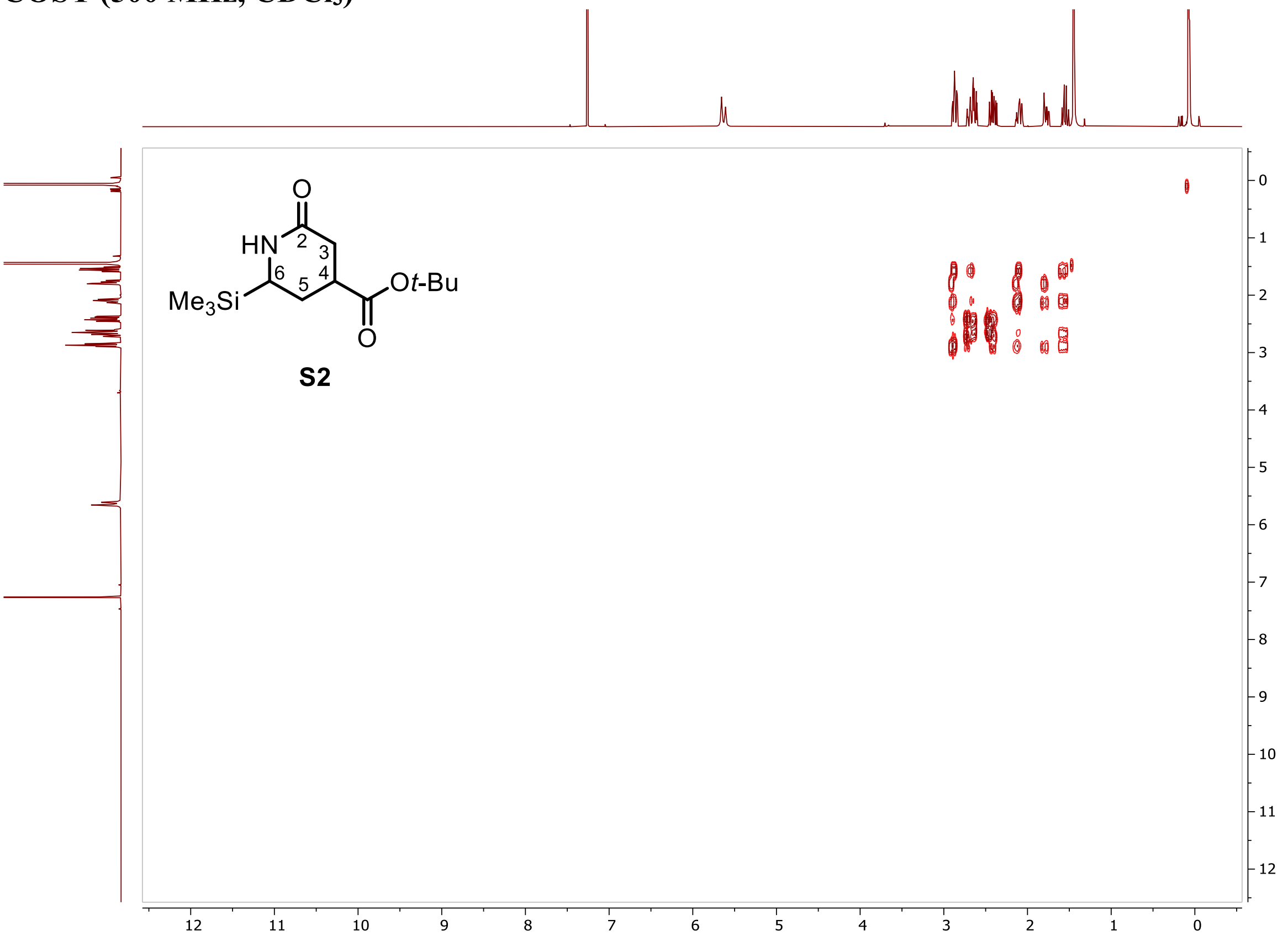


**HSQC ($^1$H 500 MHz/$^{13}$C 126 MHz, $CDCl_3$)**

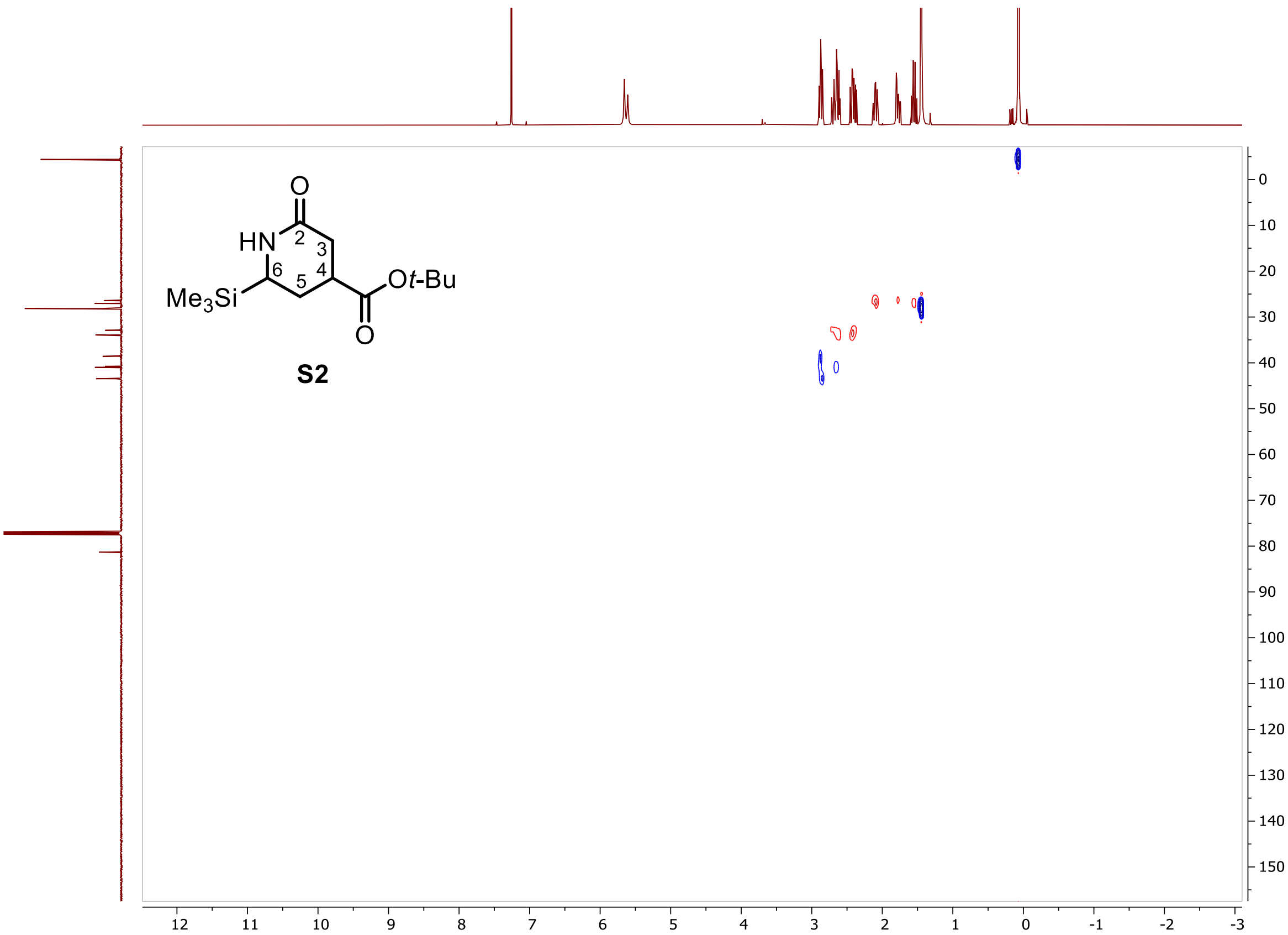

**$^{1}$H NMR (400 MHz, CDCl$_3$)**

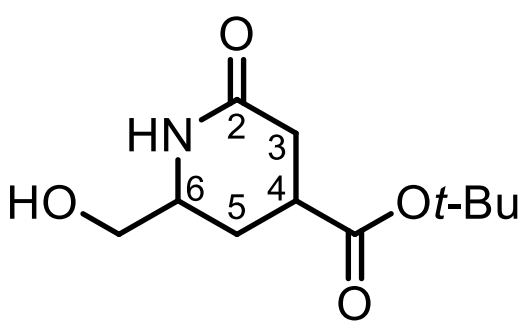


**3v**

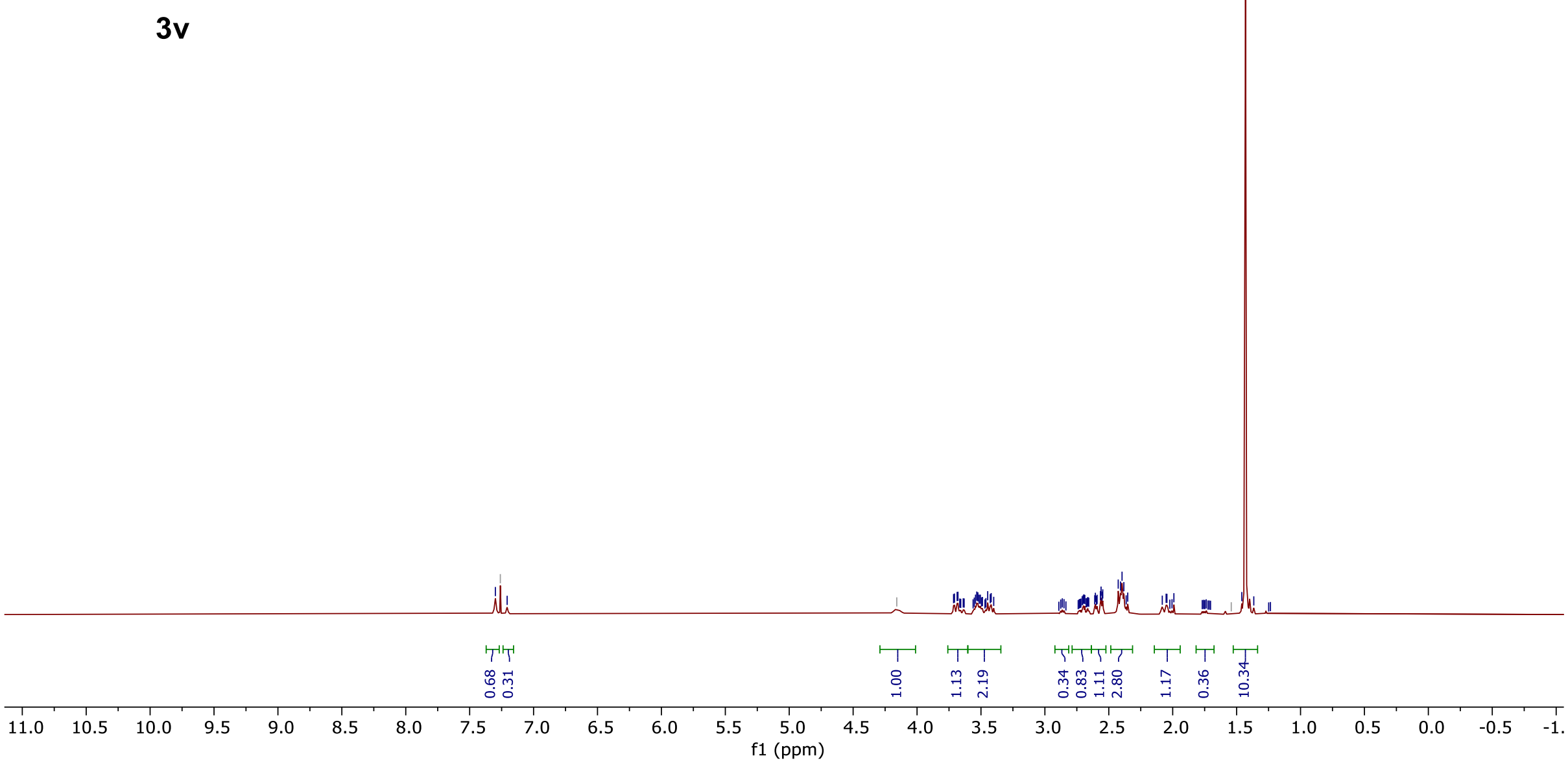


**$^{13}$C NMR (101 MHz, CDCl$_3$)**

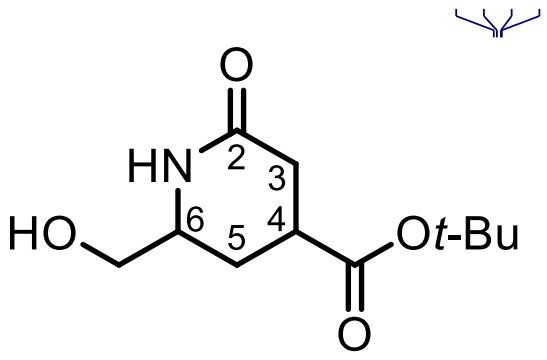


**3v**

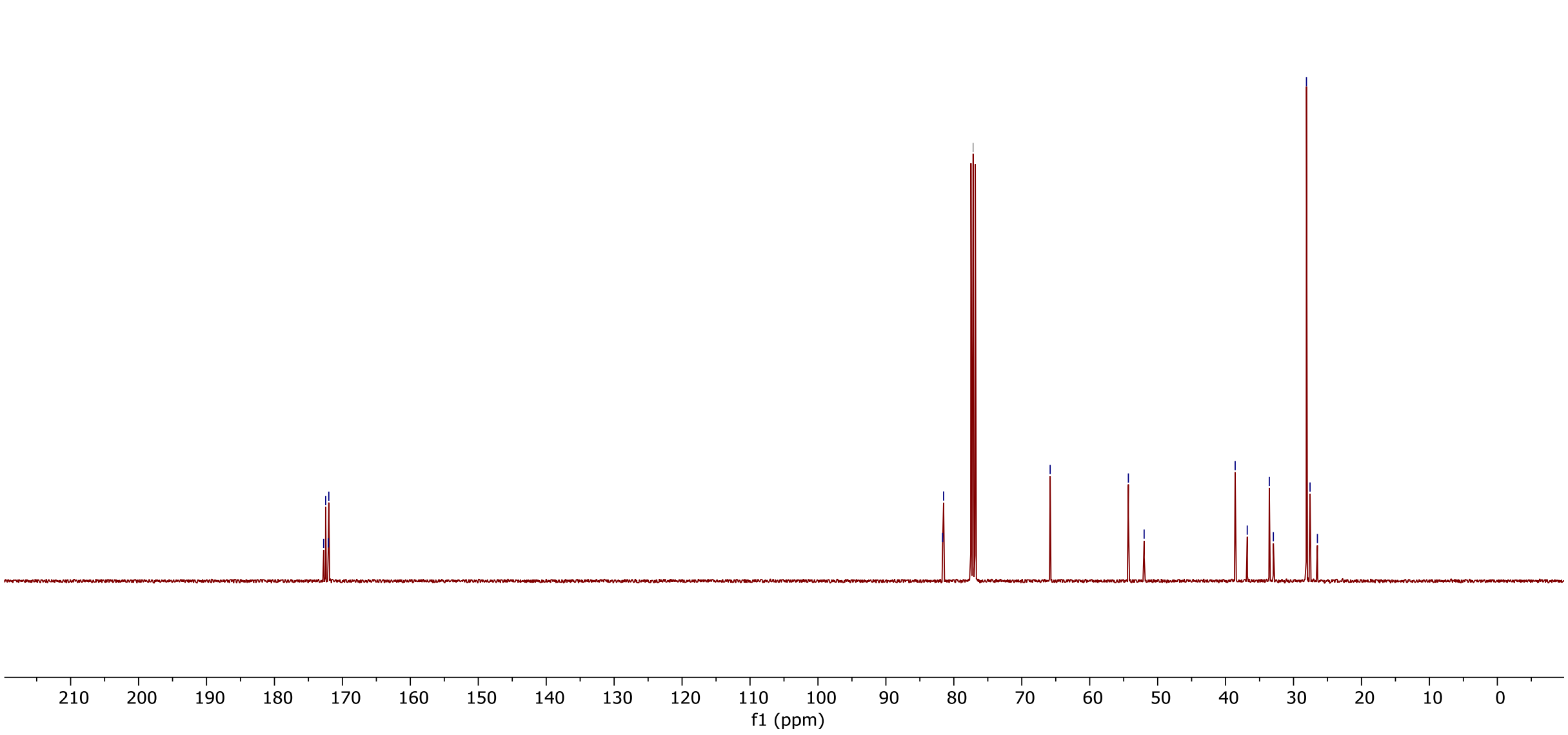

**COSY (500 MHz, $CDCl_3$)**

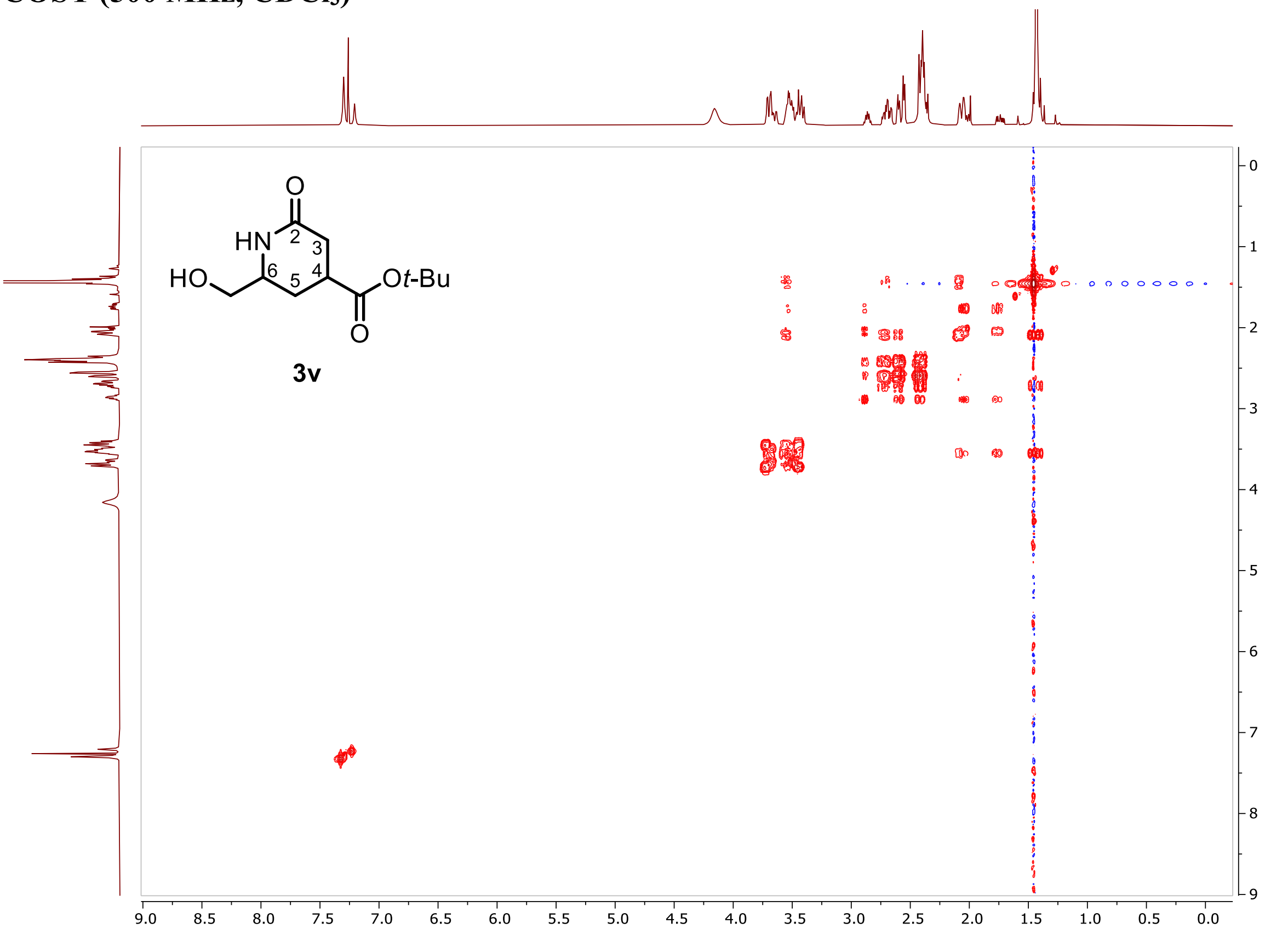


**HSQC ($^1H$ 500 MHz/$^{13}C$ 126 MHz, $CDCl_3$)**

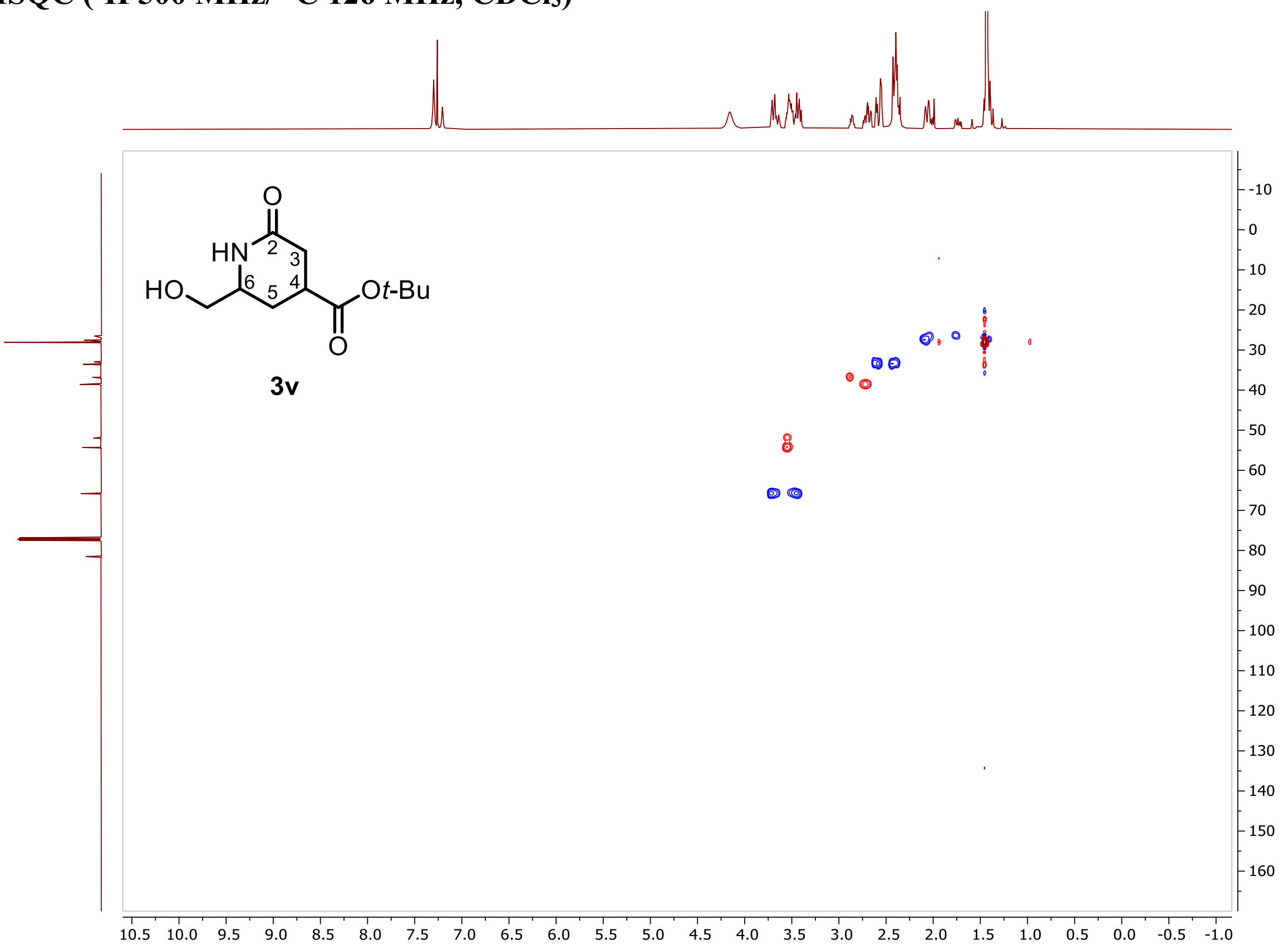

$^{1}$H NMR (500 MHz, $CDCl_3$)

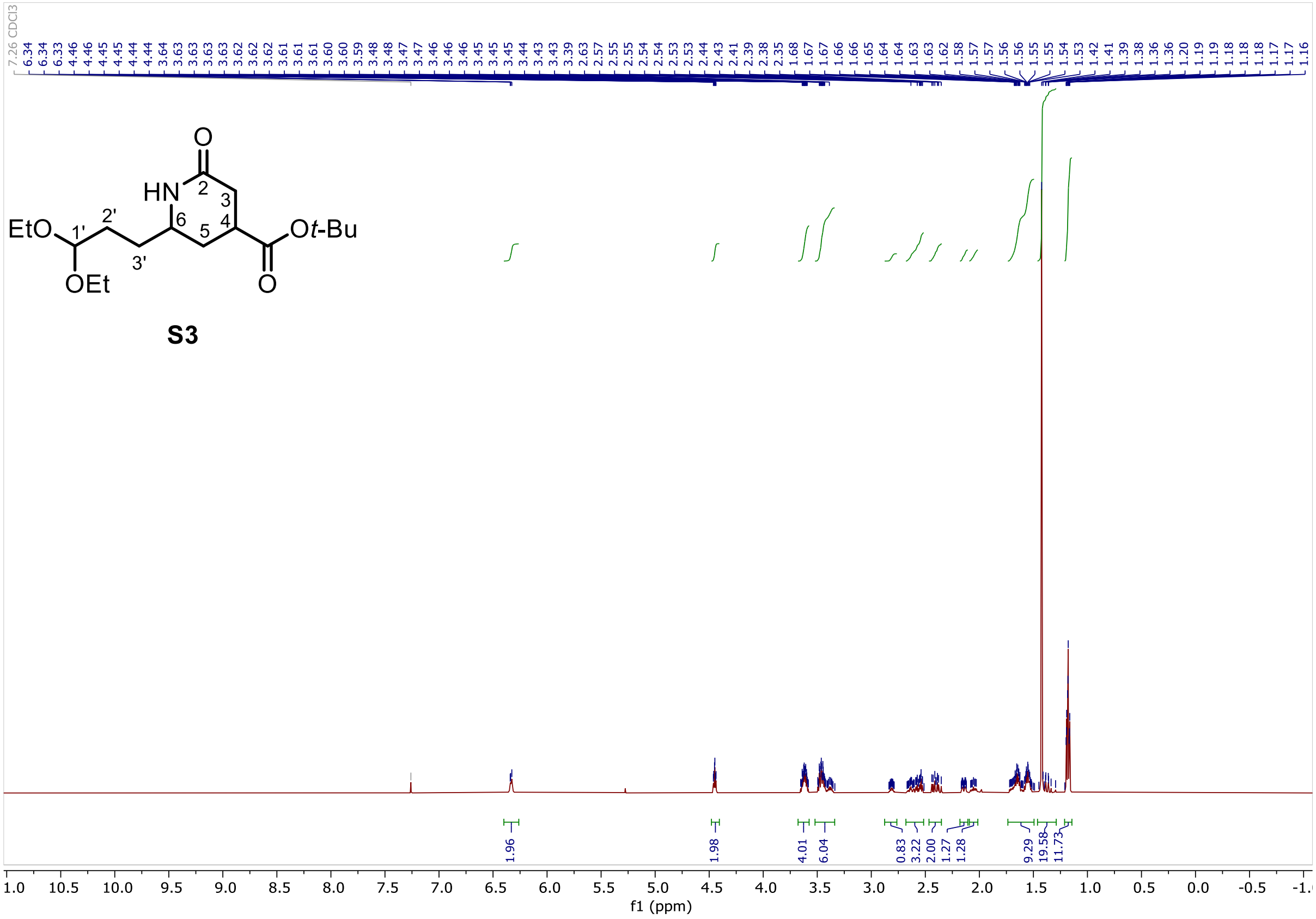

$^{13}$C NMR (126 MHz, $CDCl_3$)

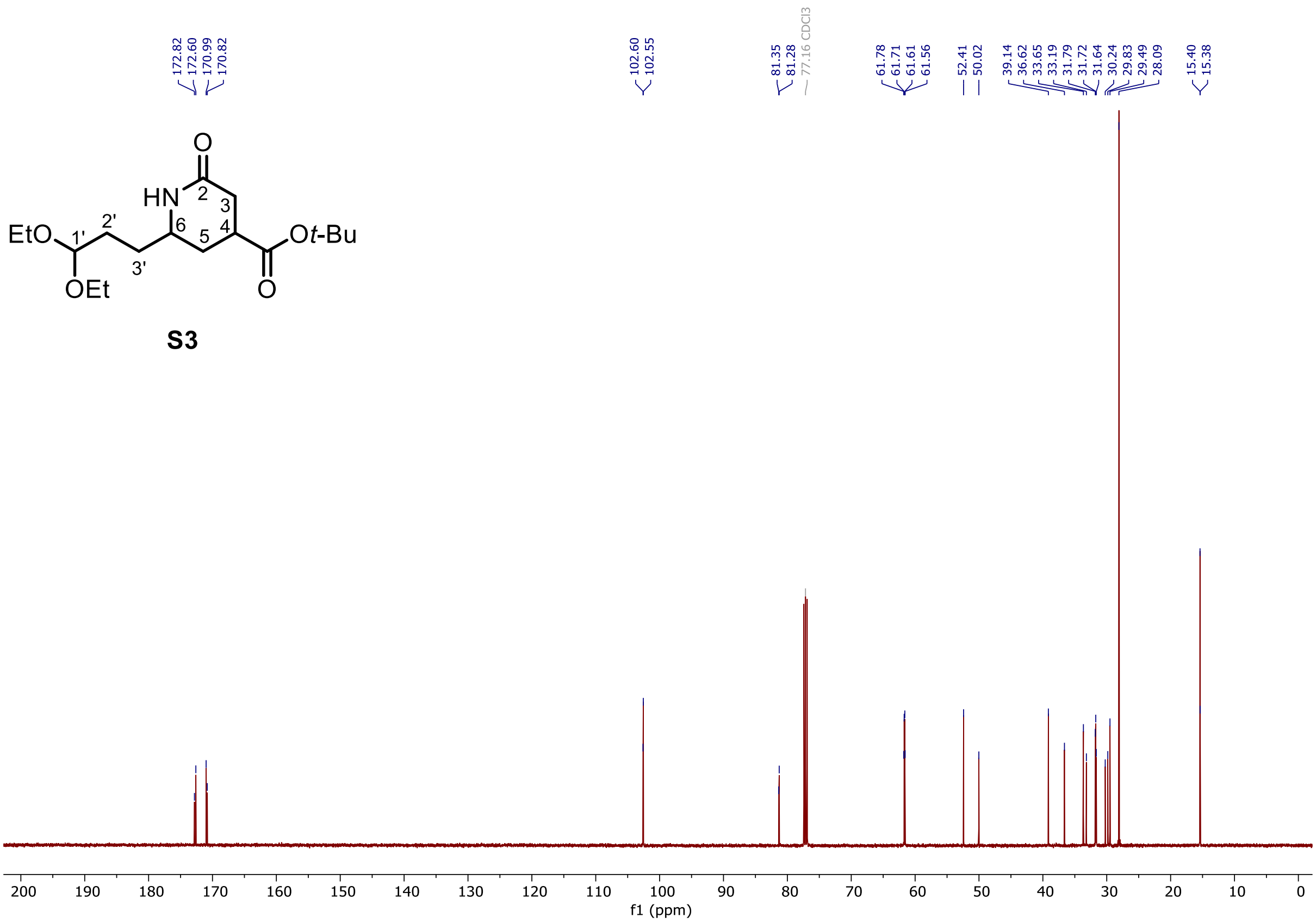

**COSY (500 MHz, CDCl$_3$)**

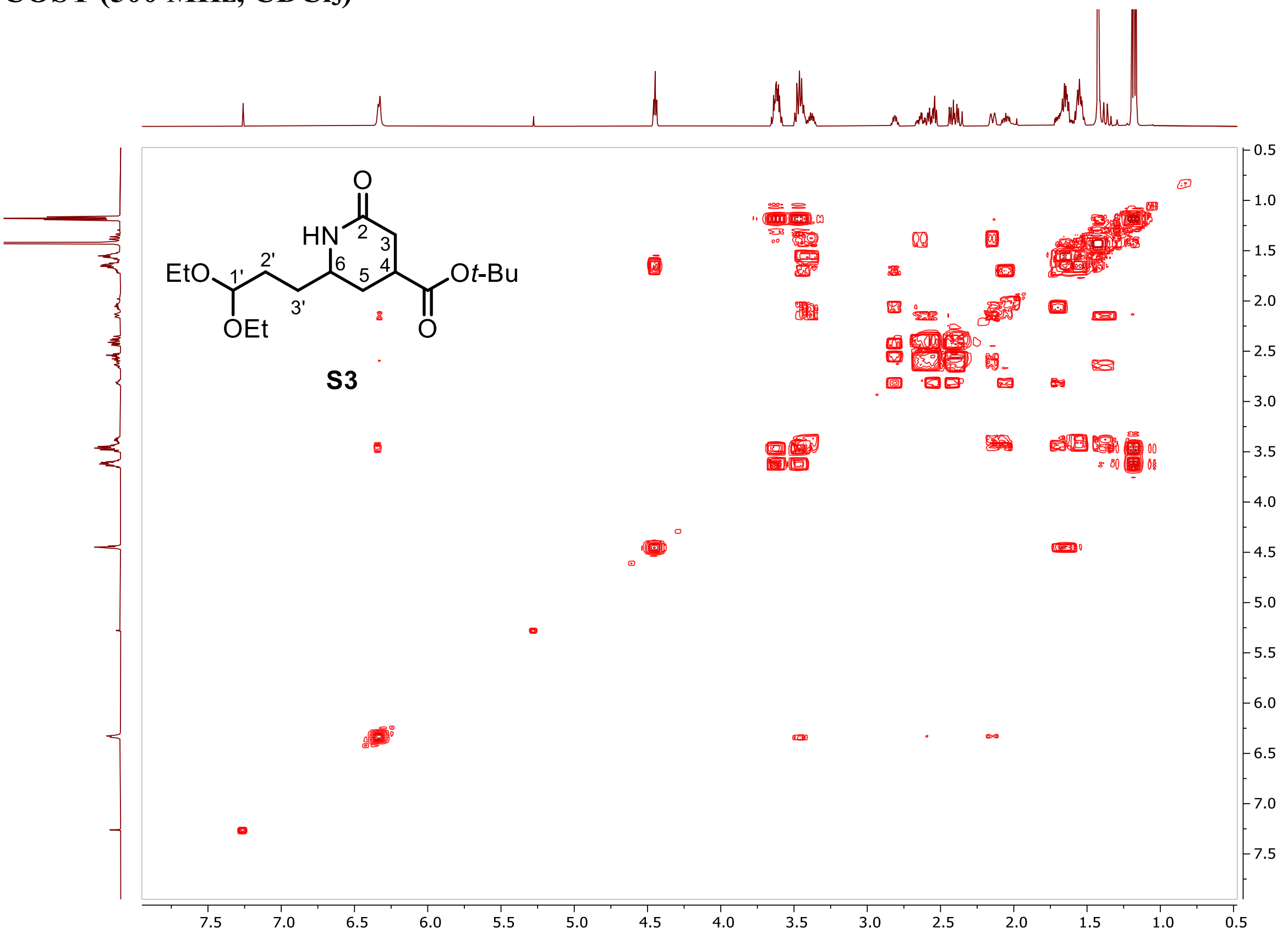


**HSQC ($^1$H 500 MHz/$^{13}$C 126 MHz, CDCl$_3$)**

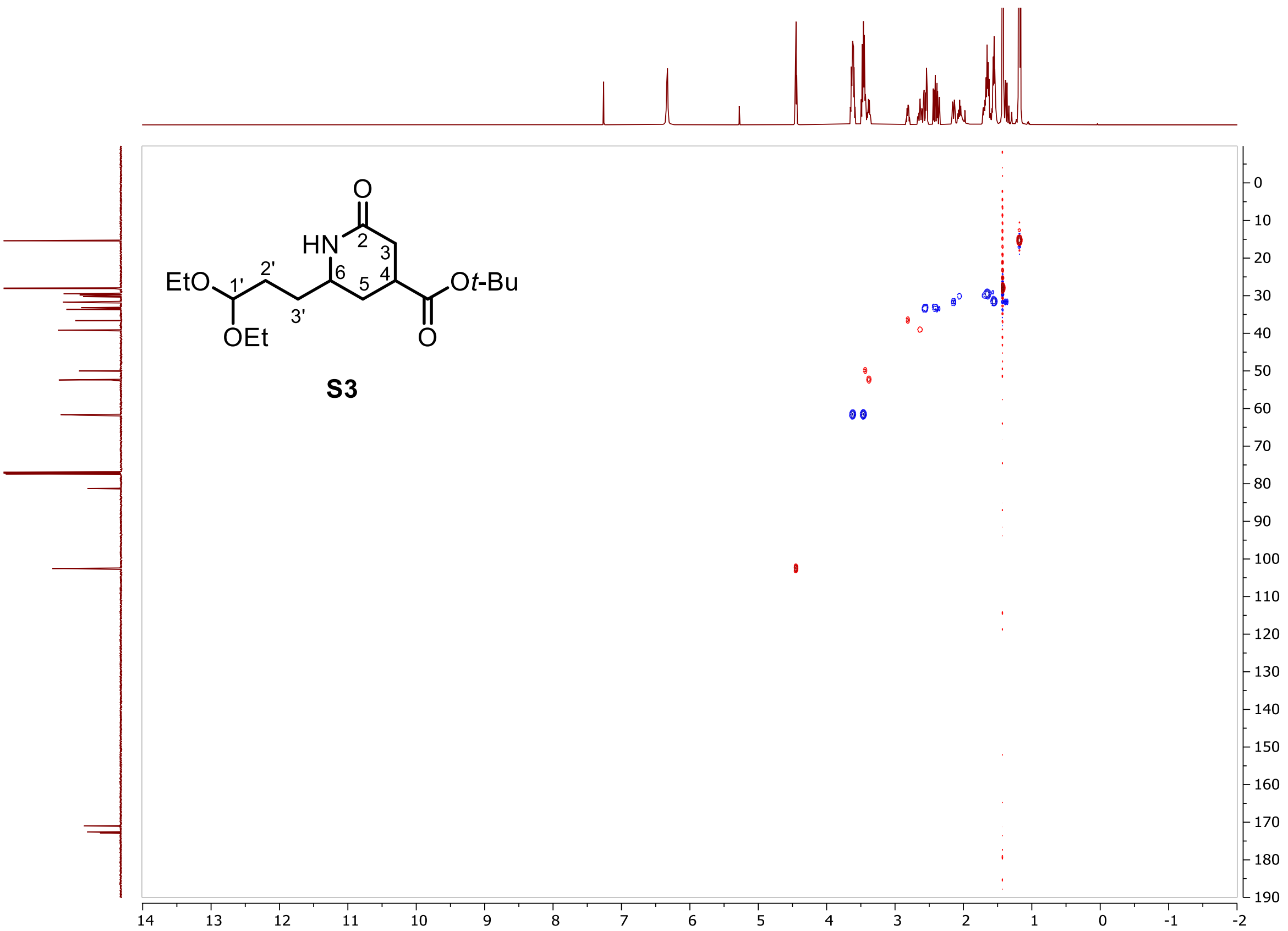

**$^{1}$H NMR (500 MHz, $CDCl_3$)**

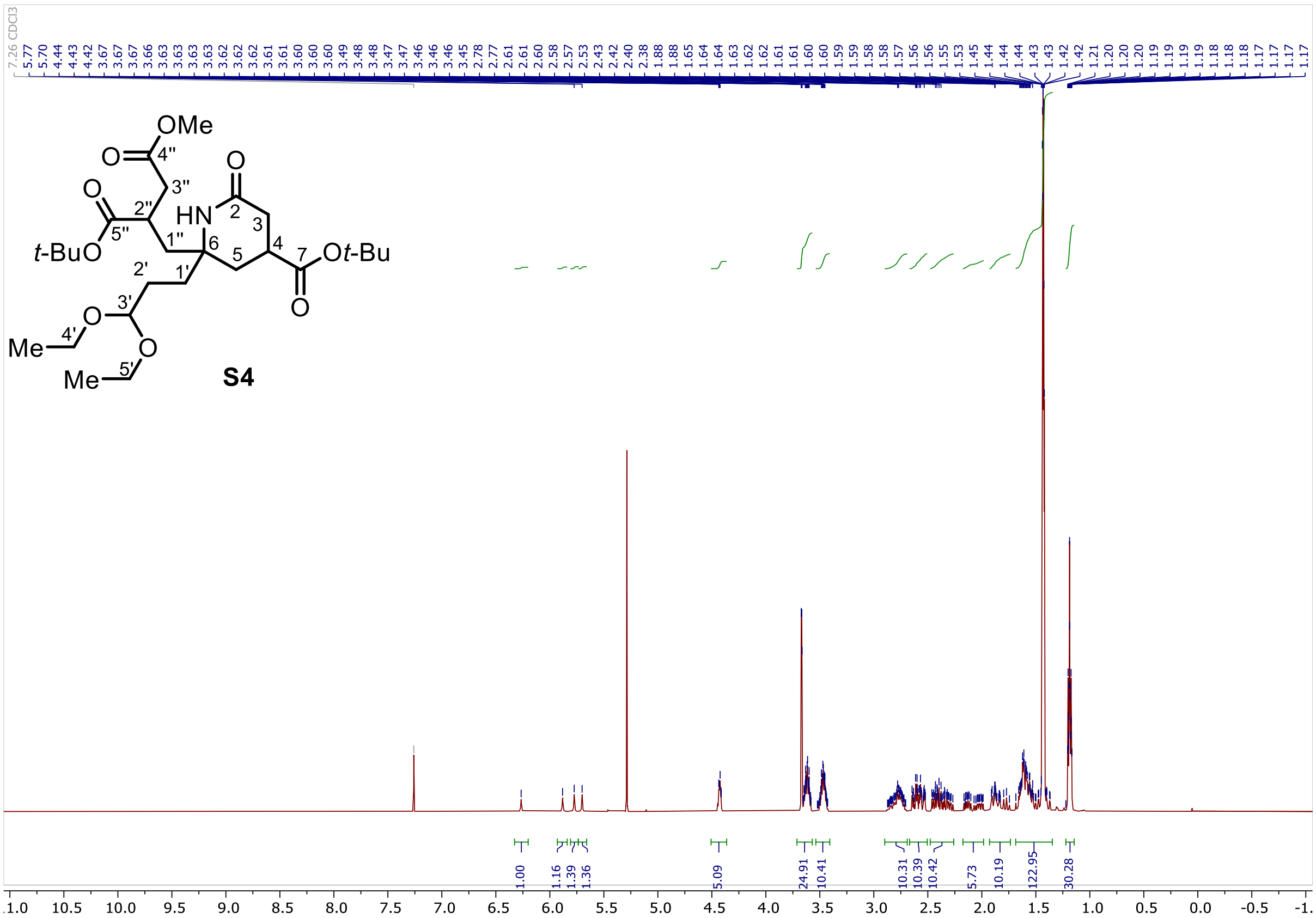


**$^{13}$C NMR (126 MHz, $CDCl_3$)**

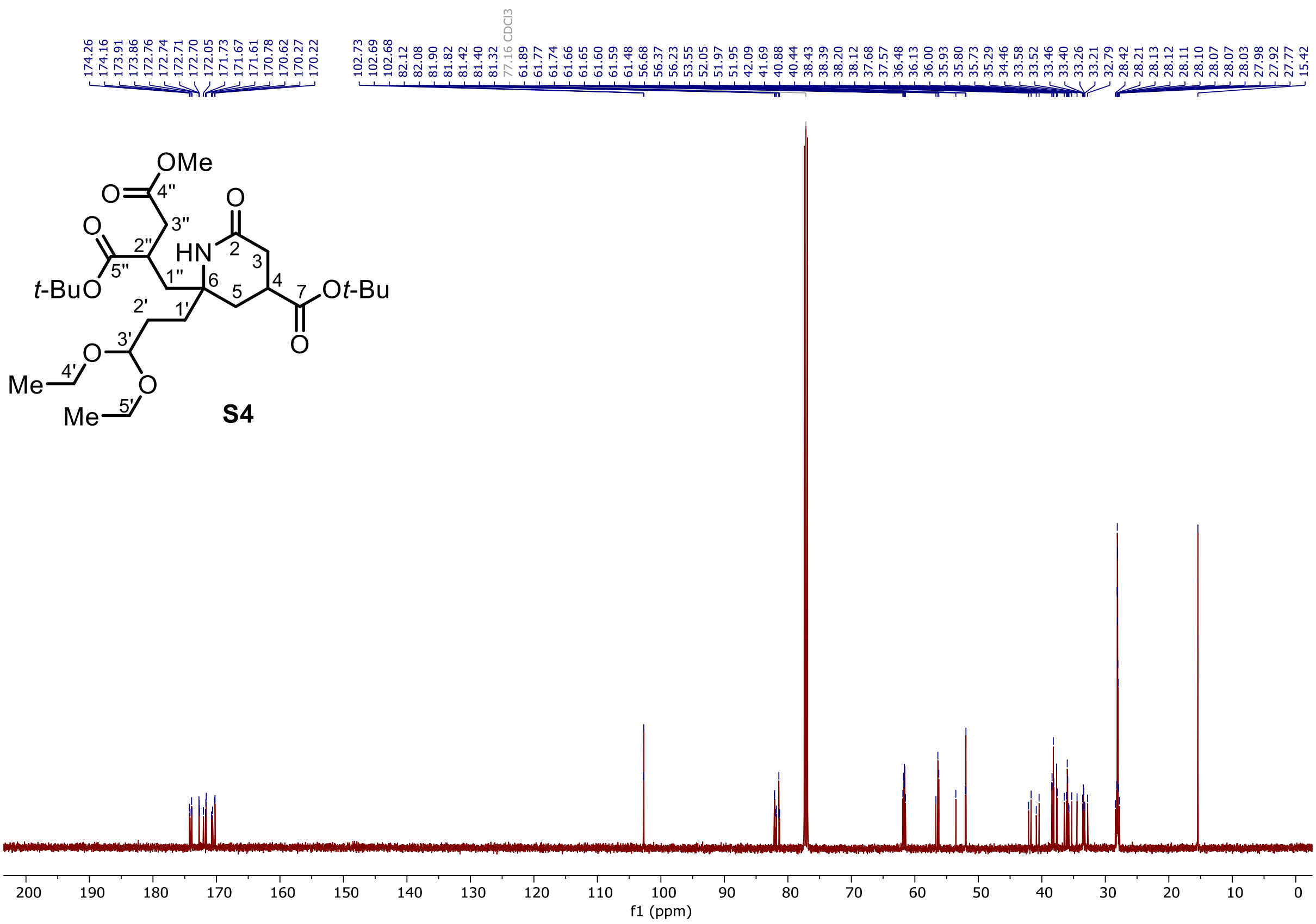

**COSY (500 MHz, CDCl$_3$)**

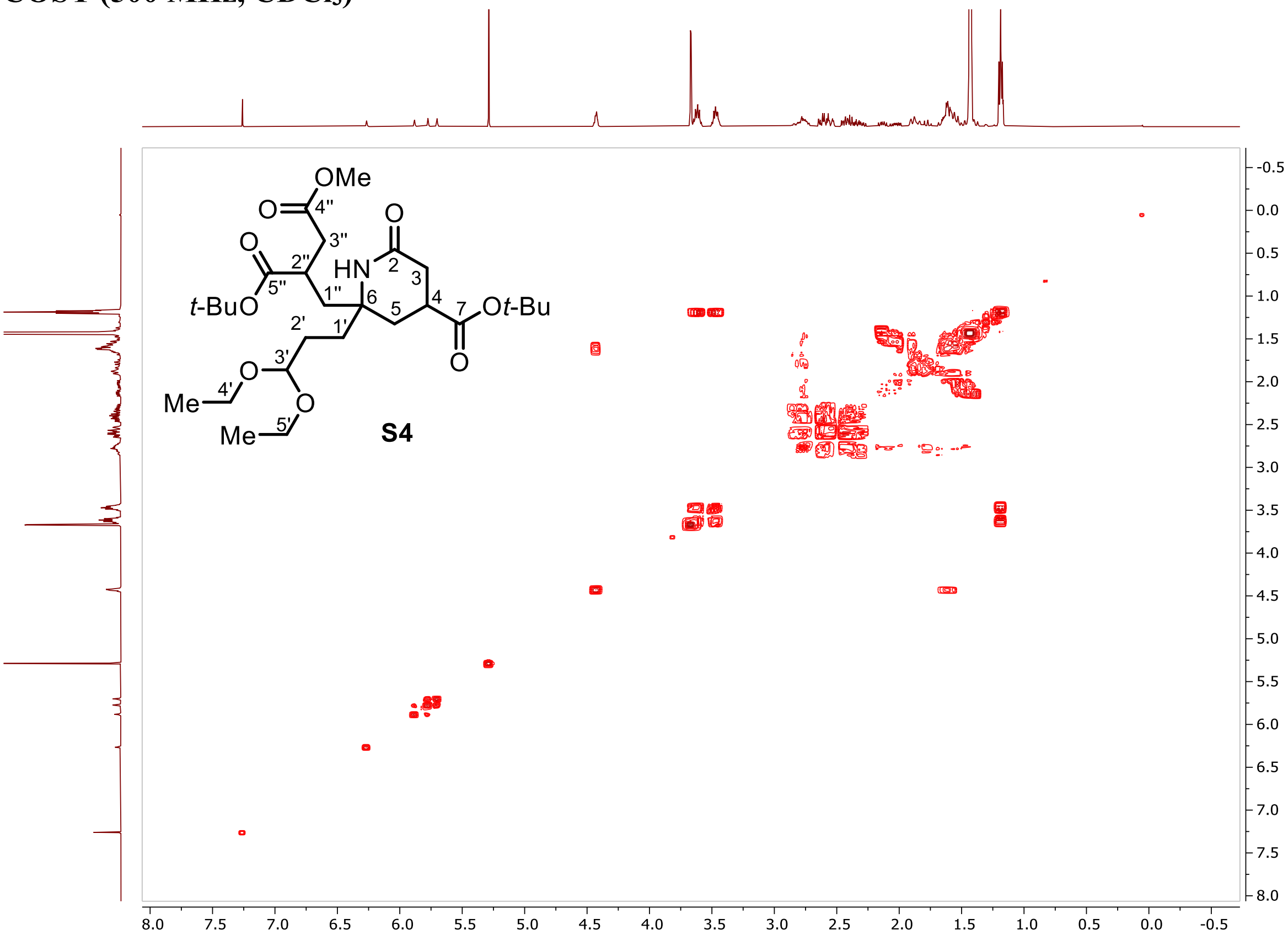

**HSQC ($^1$H 500 MHz/$^{13}$C 126 MHz, CDCl$_3$)**

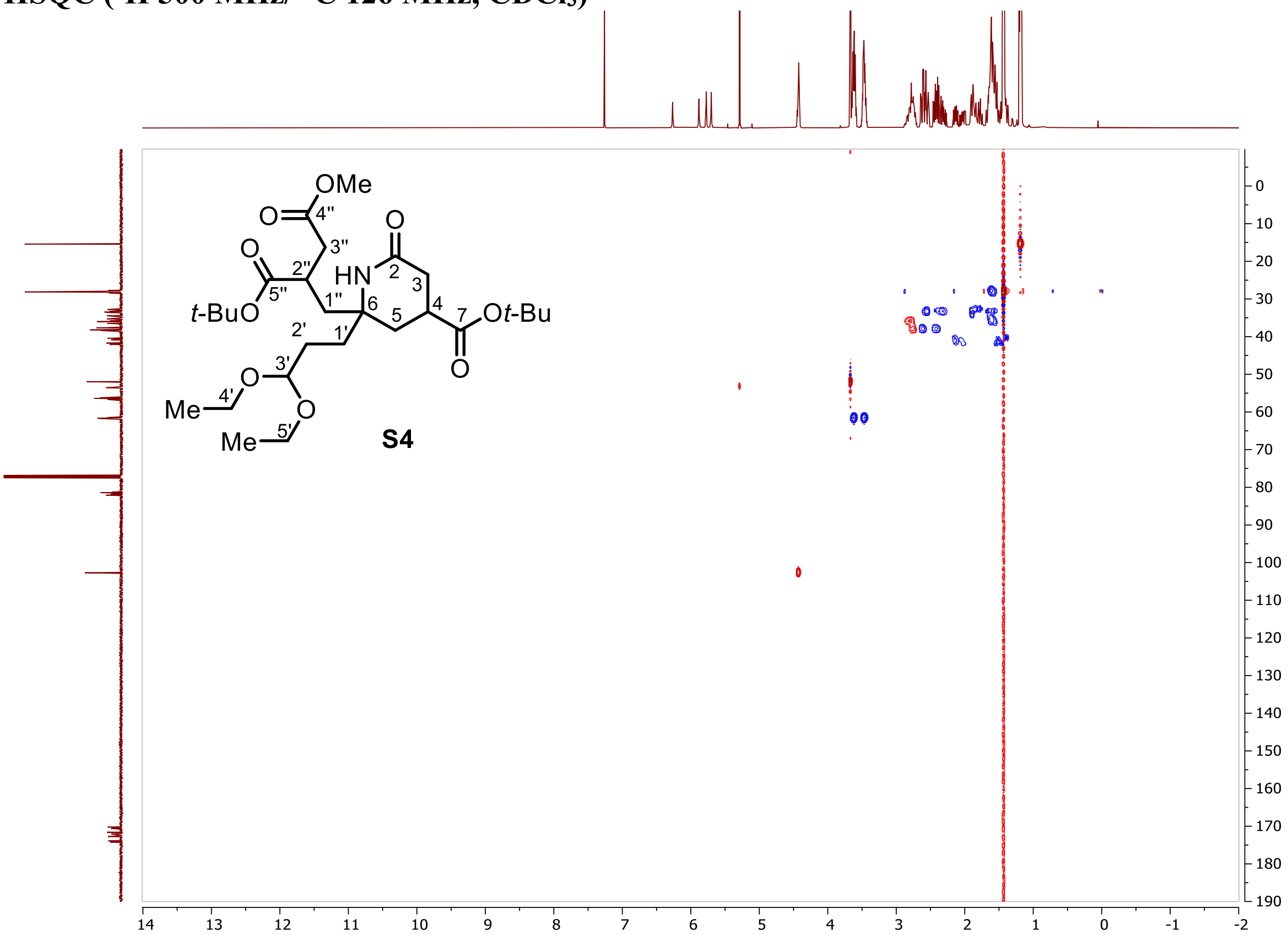

**$^{1}$H NMR (400 MHz, $CDCl_3$)**

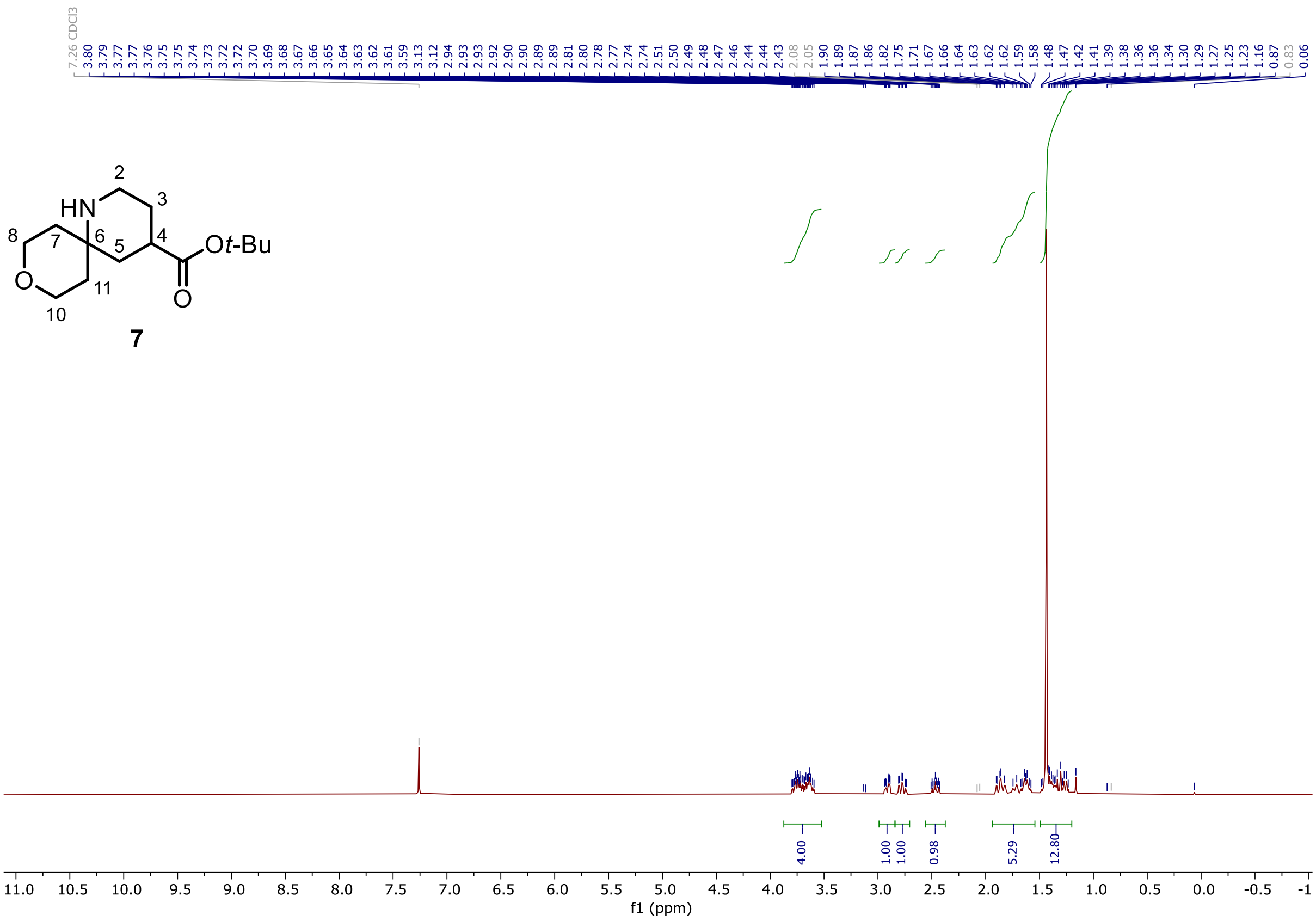


**$^{13}$C NMR (101 MHz, $CDCl_3$)**

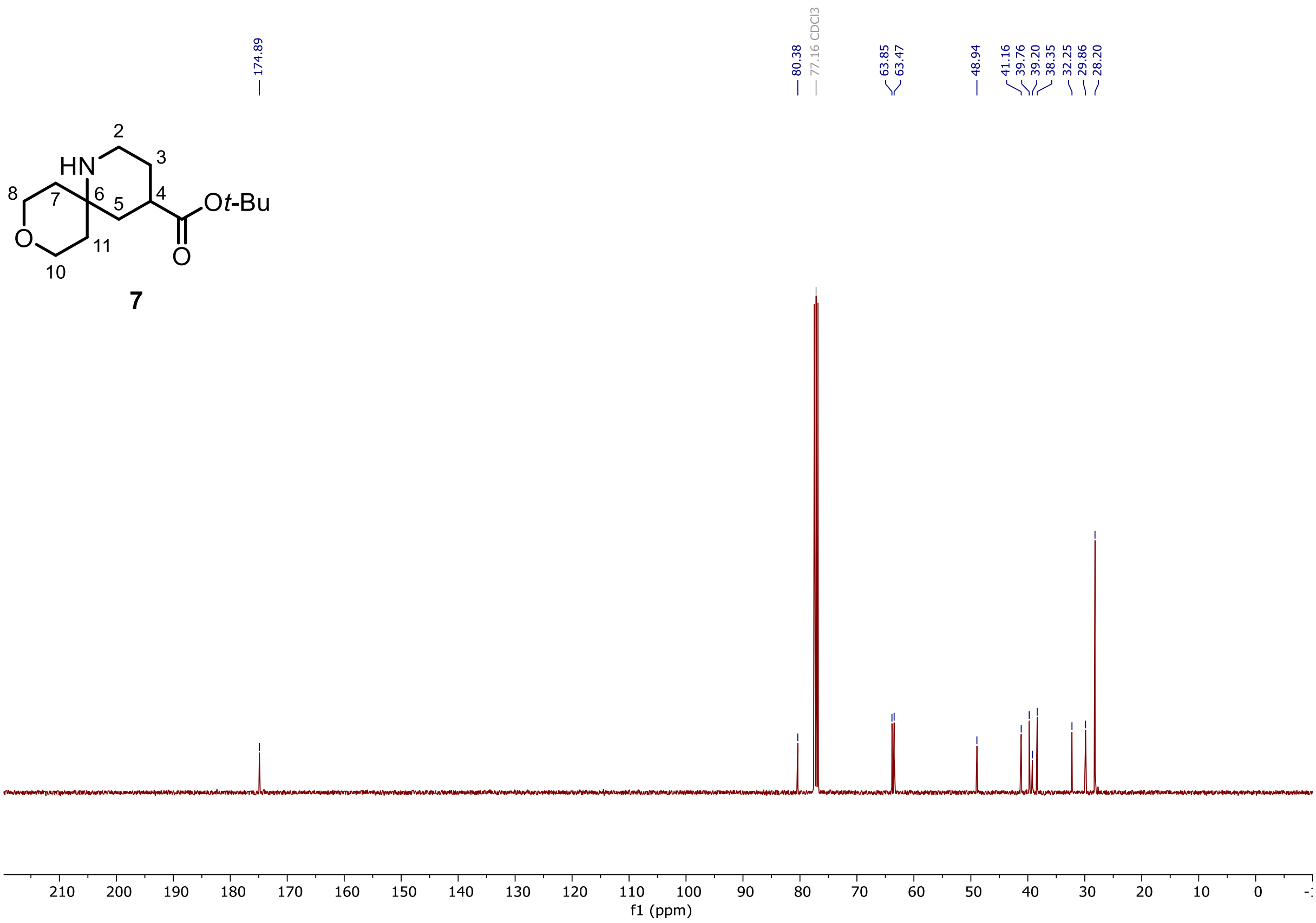

**COSY (500 MHz, $CDCl_3$)**

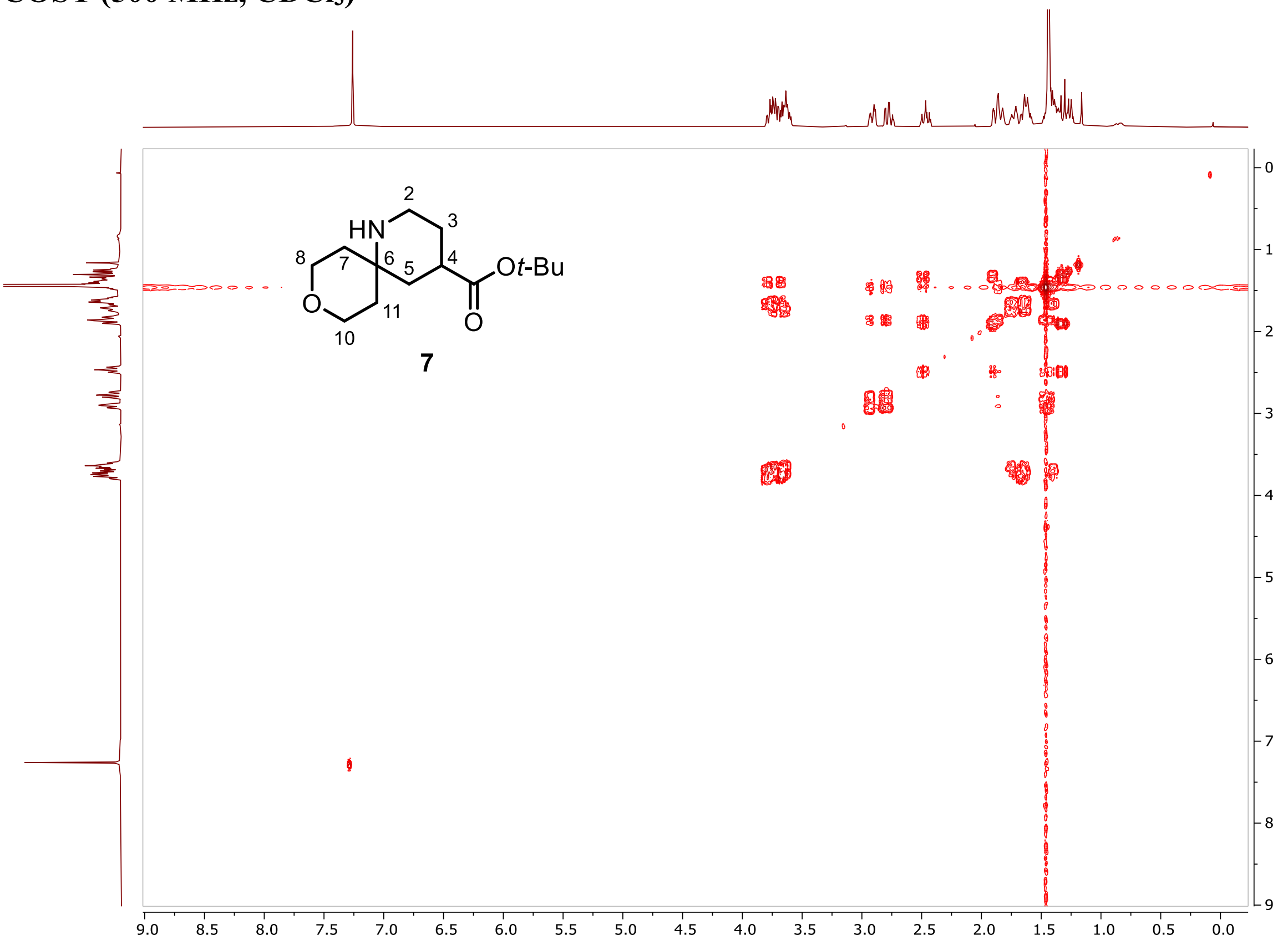

**HSQC ($^1$H 500 MHz/$^{13}$C 126 MHz, $CDCl_3$)**

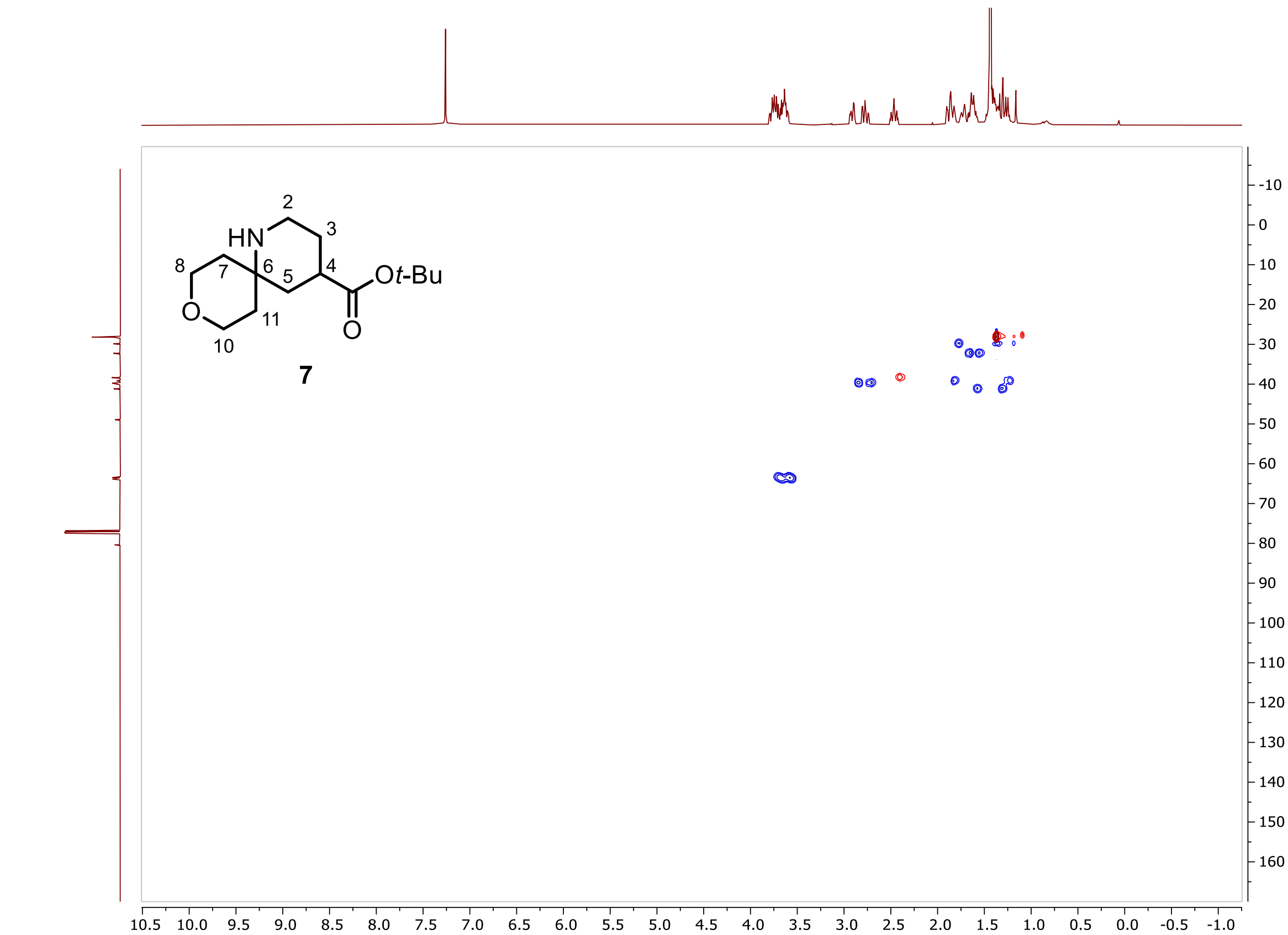

**$^{1}$H NMR (500 MHz, AcOH-$d_4$)**

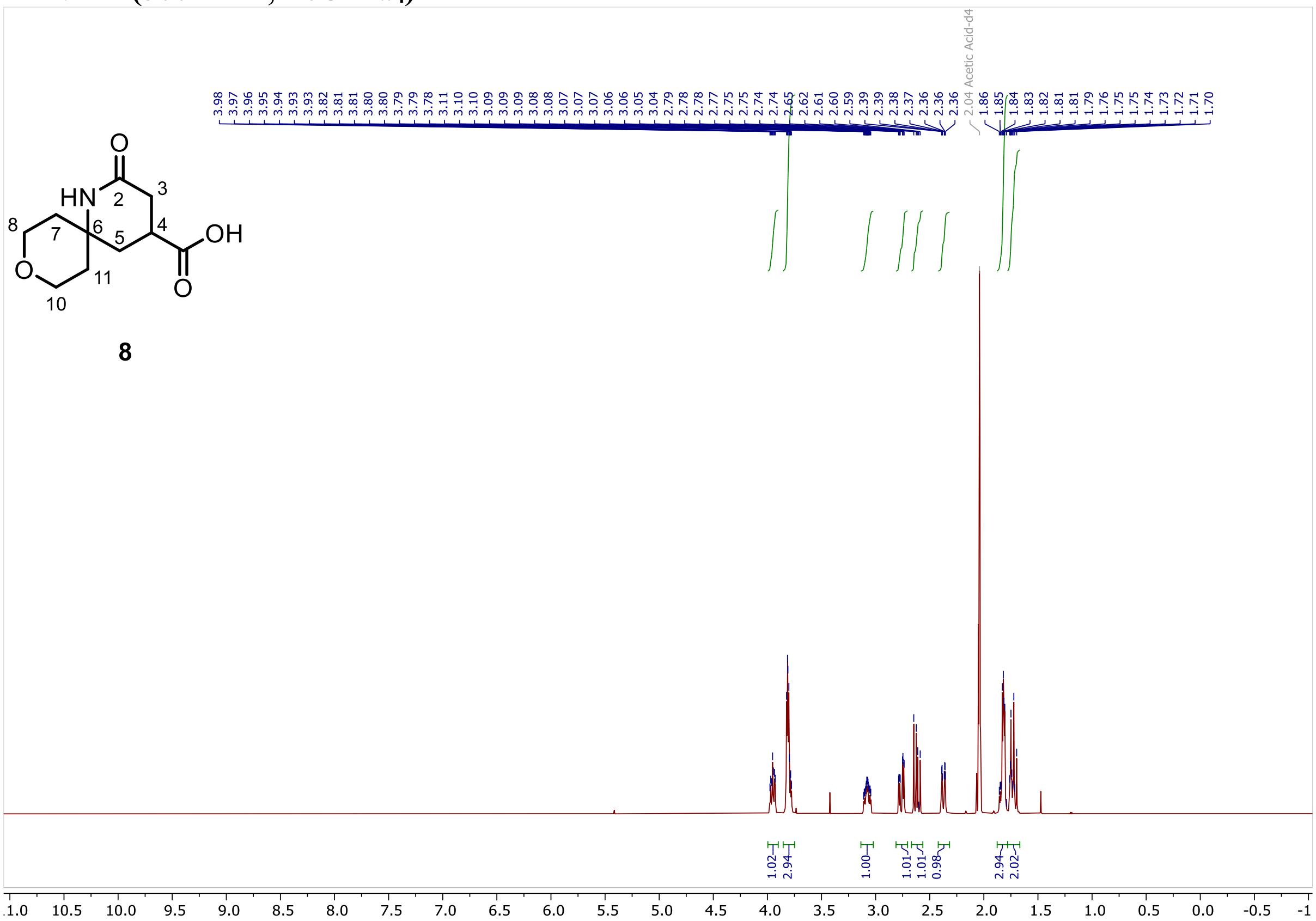

**$^{13}$C NMR (126 MHz, AcOH-$d_4$)**

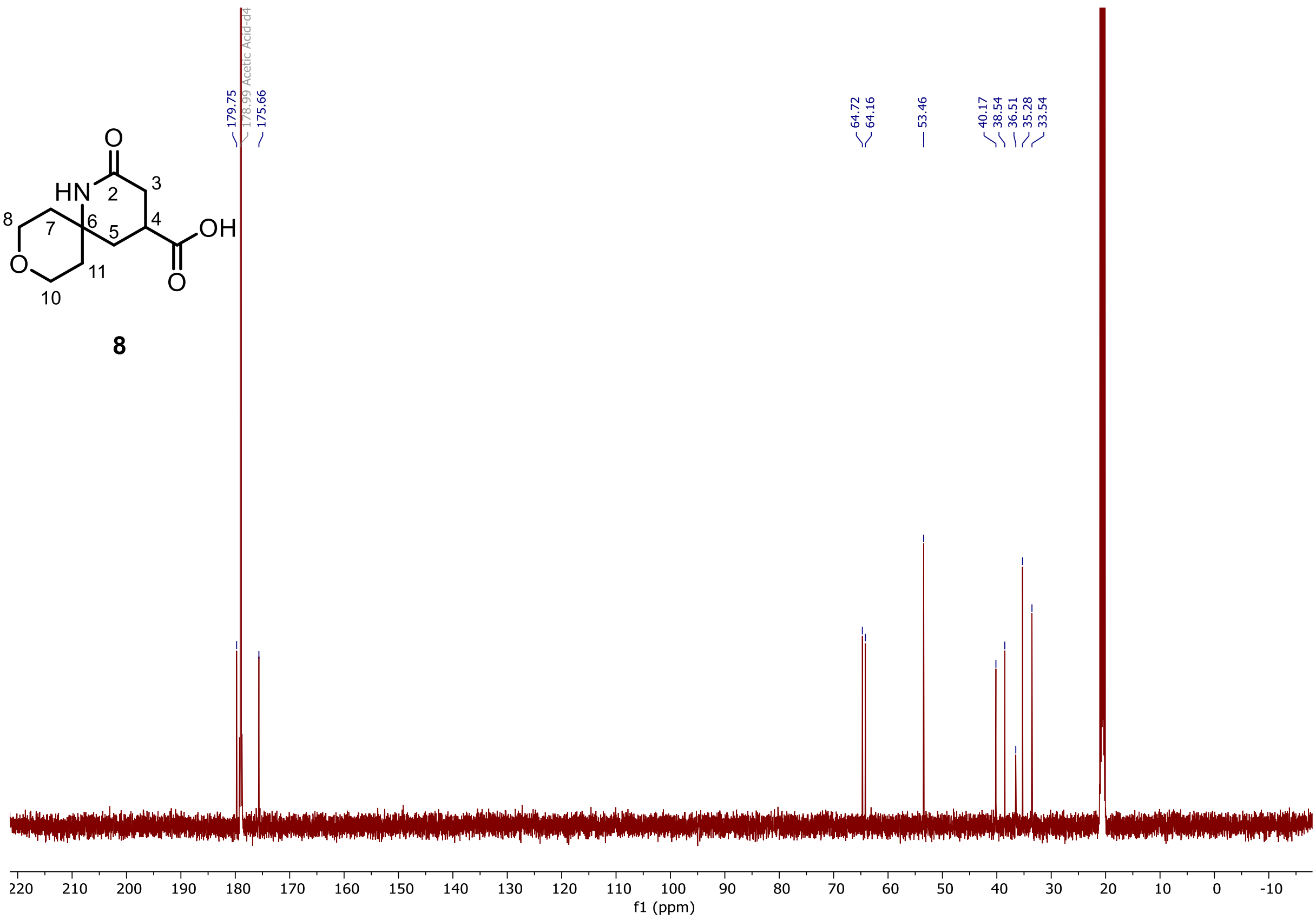

**COSY (500 MHz, AcOH-$d_4$)**

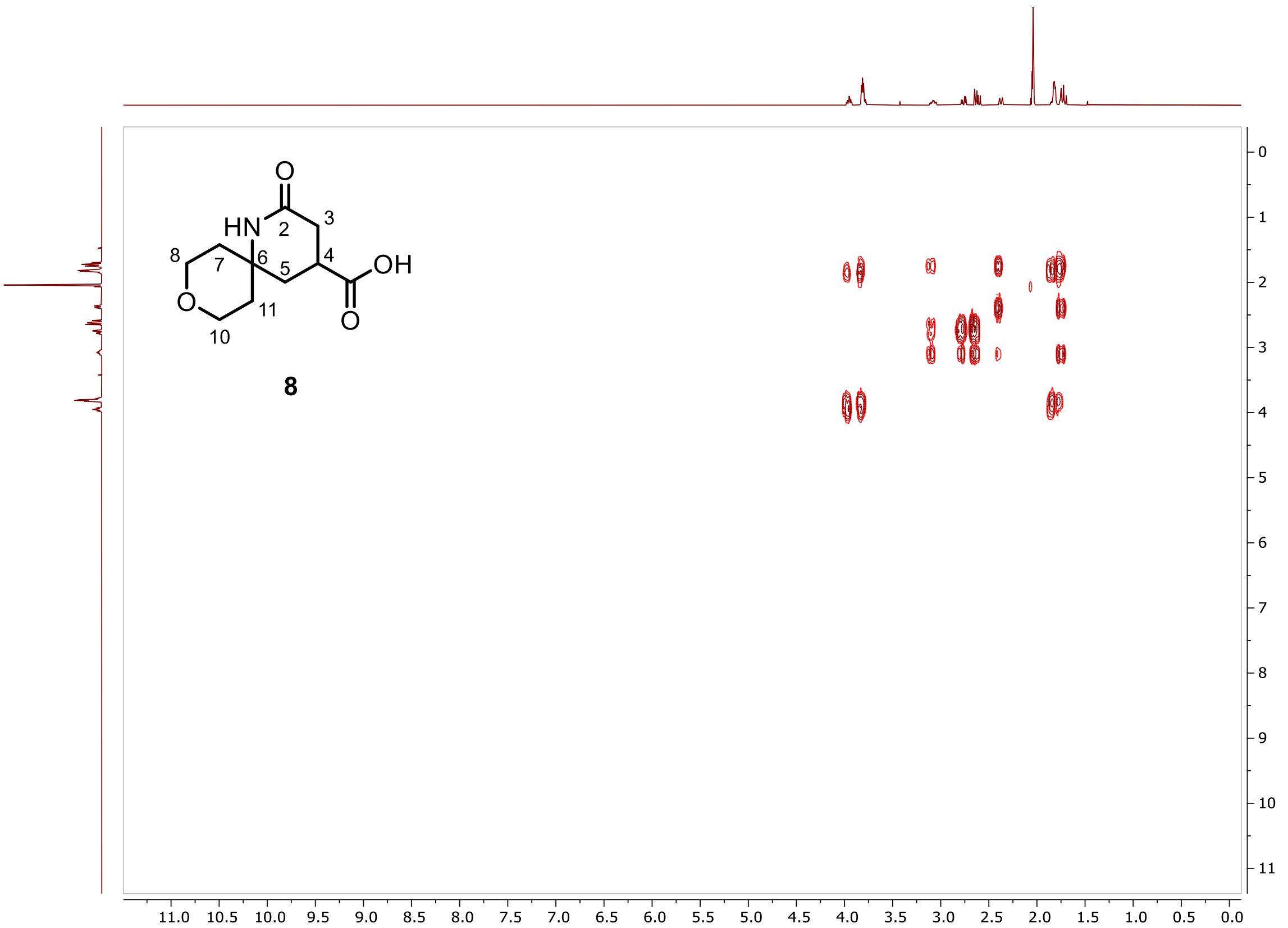


**HSQC ($^1$H 500 MHz/$^{13}$C 126 MHz, AcOH-$d_4$)**

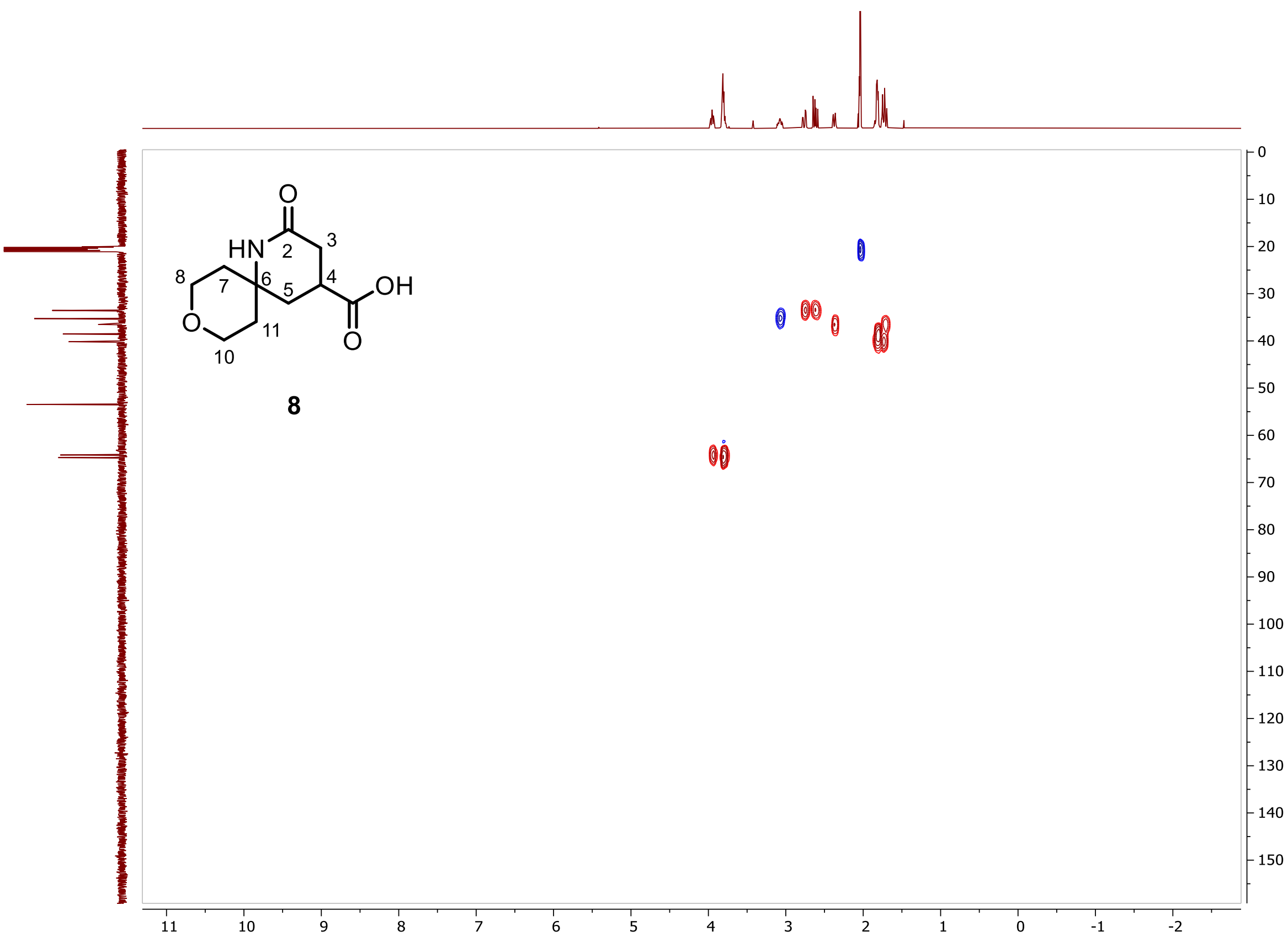

**$^{1}$H NMR (500 MHz, CDCl$_3$)**

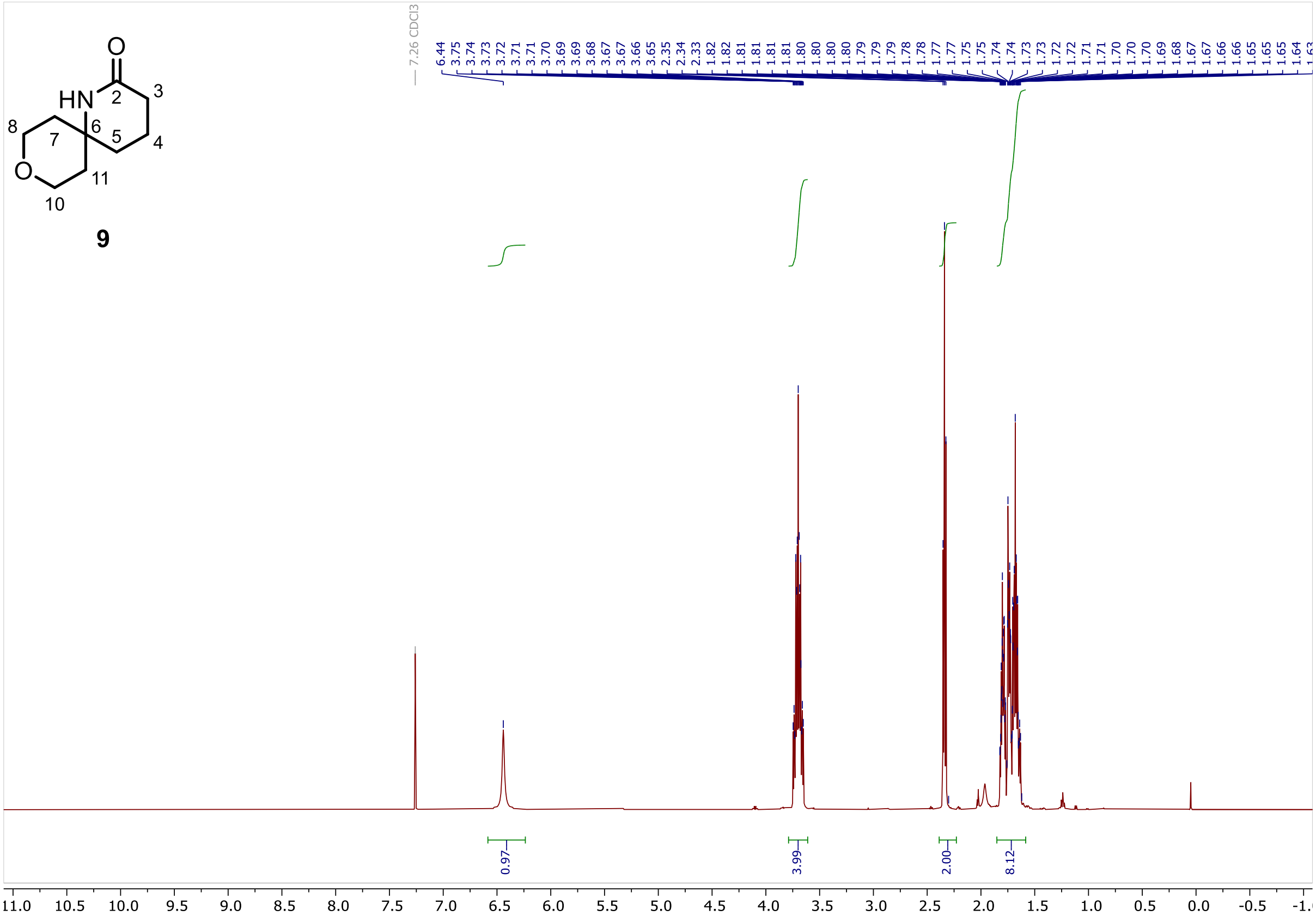


**$^{13}$C NMR (126 MHz, CDCl$_3$)**

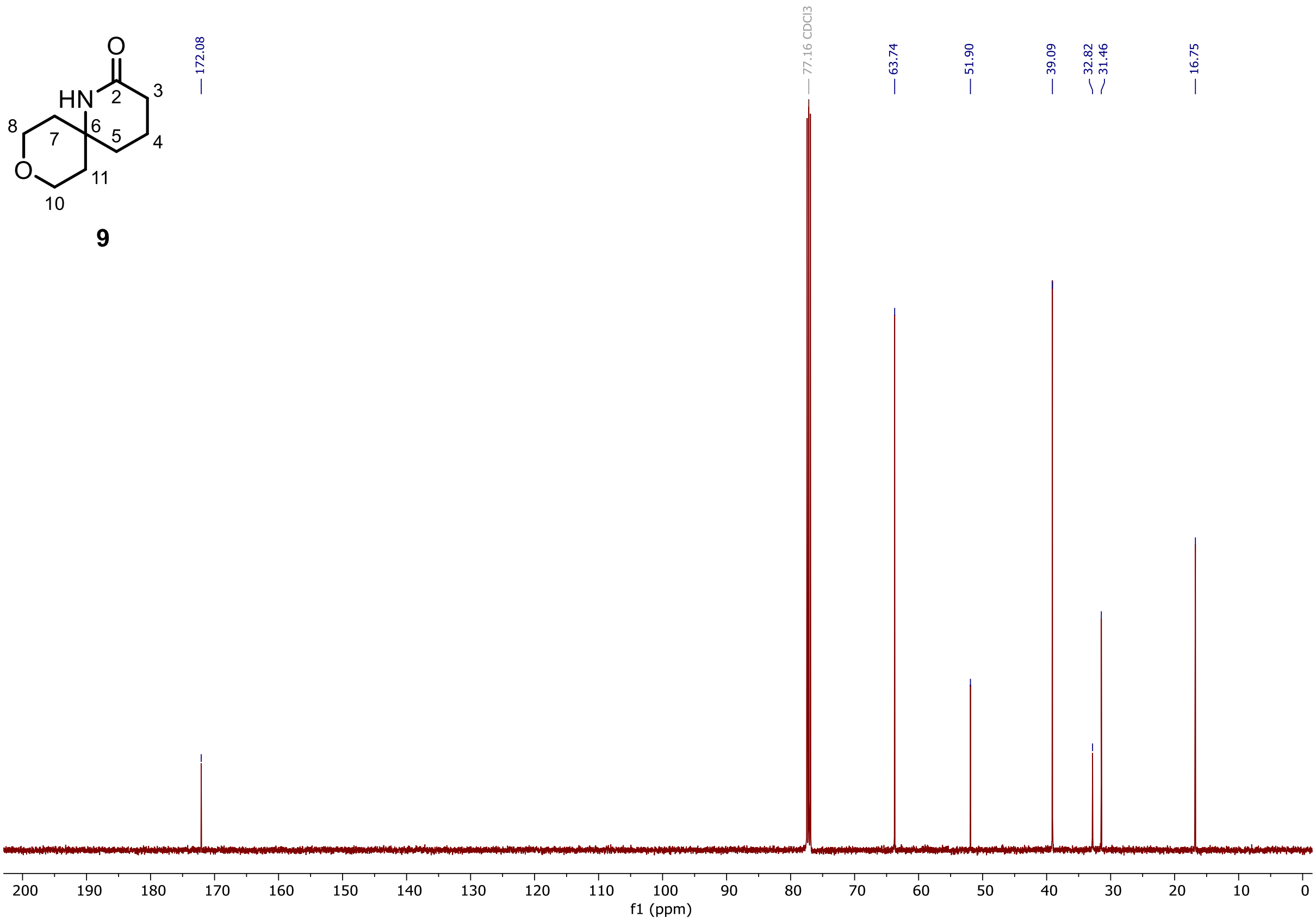

**COSY (500 MHz, CDCl$_3$)**

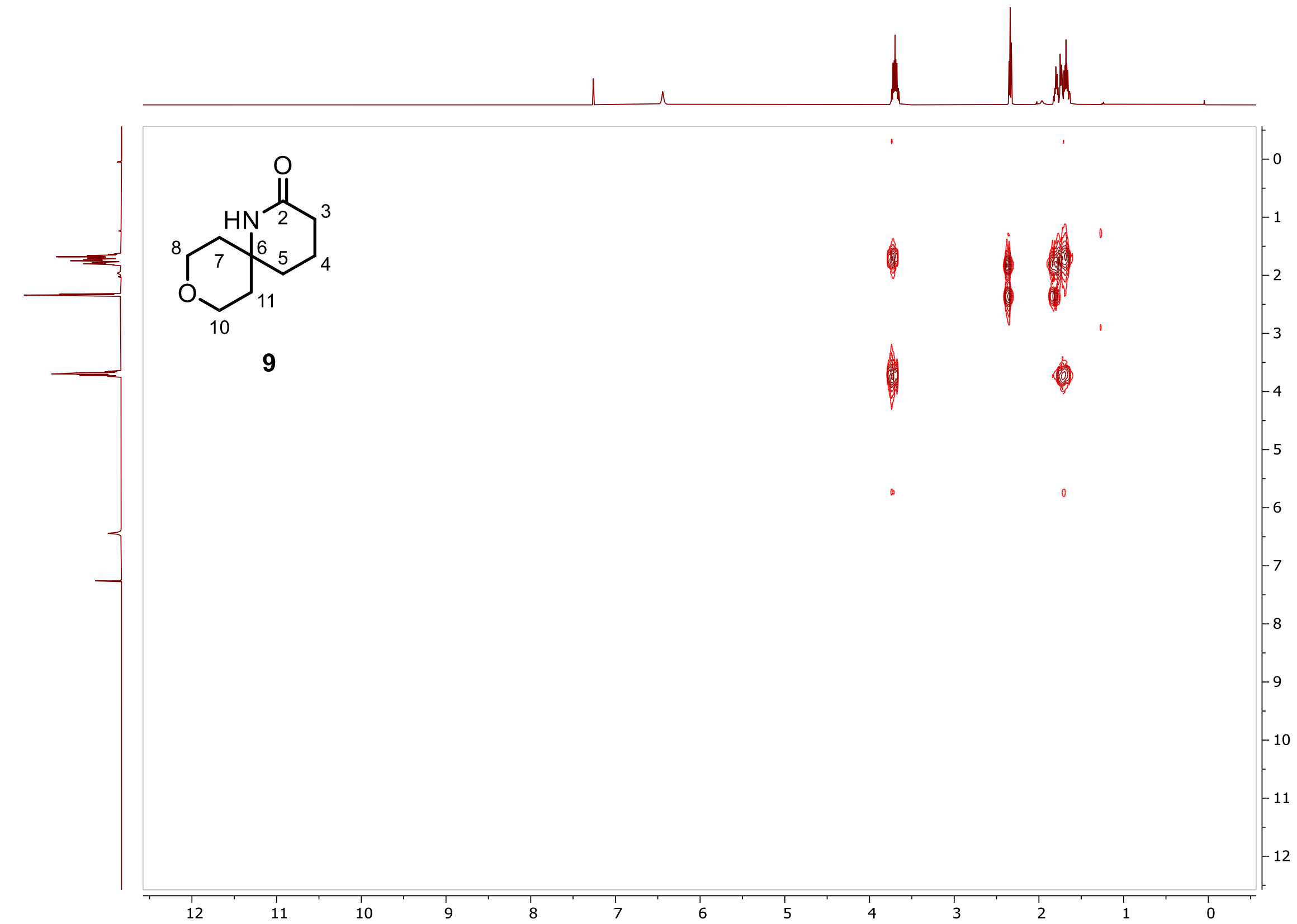


**HSQC ($^1$H 500 MHz/$^{13}$C 126 MHz, CDCl$_3$)**

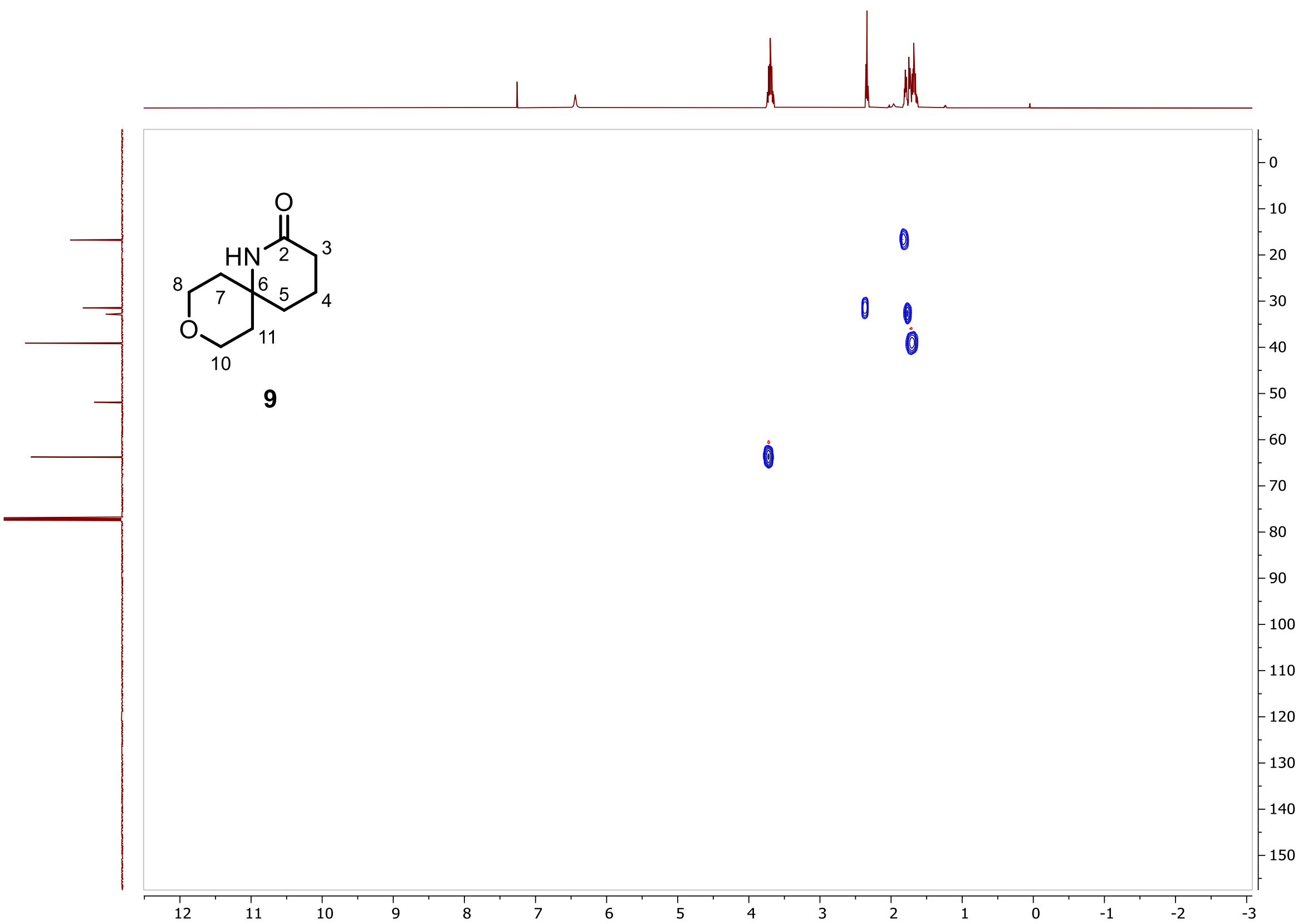

## $^{1}H$ NMR (400 MHz, $CDCl_3$)

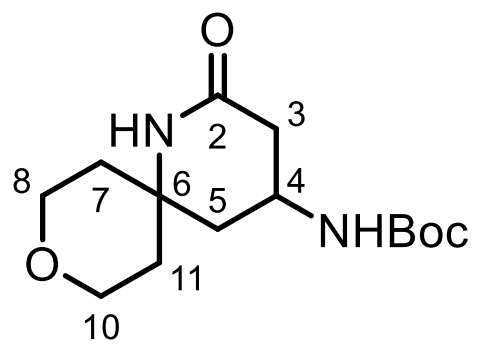


**10**

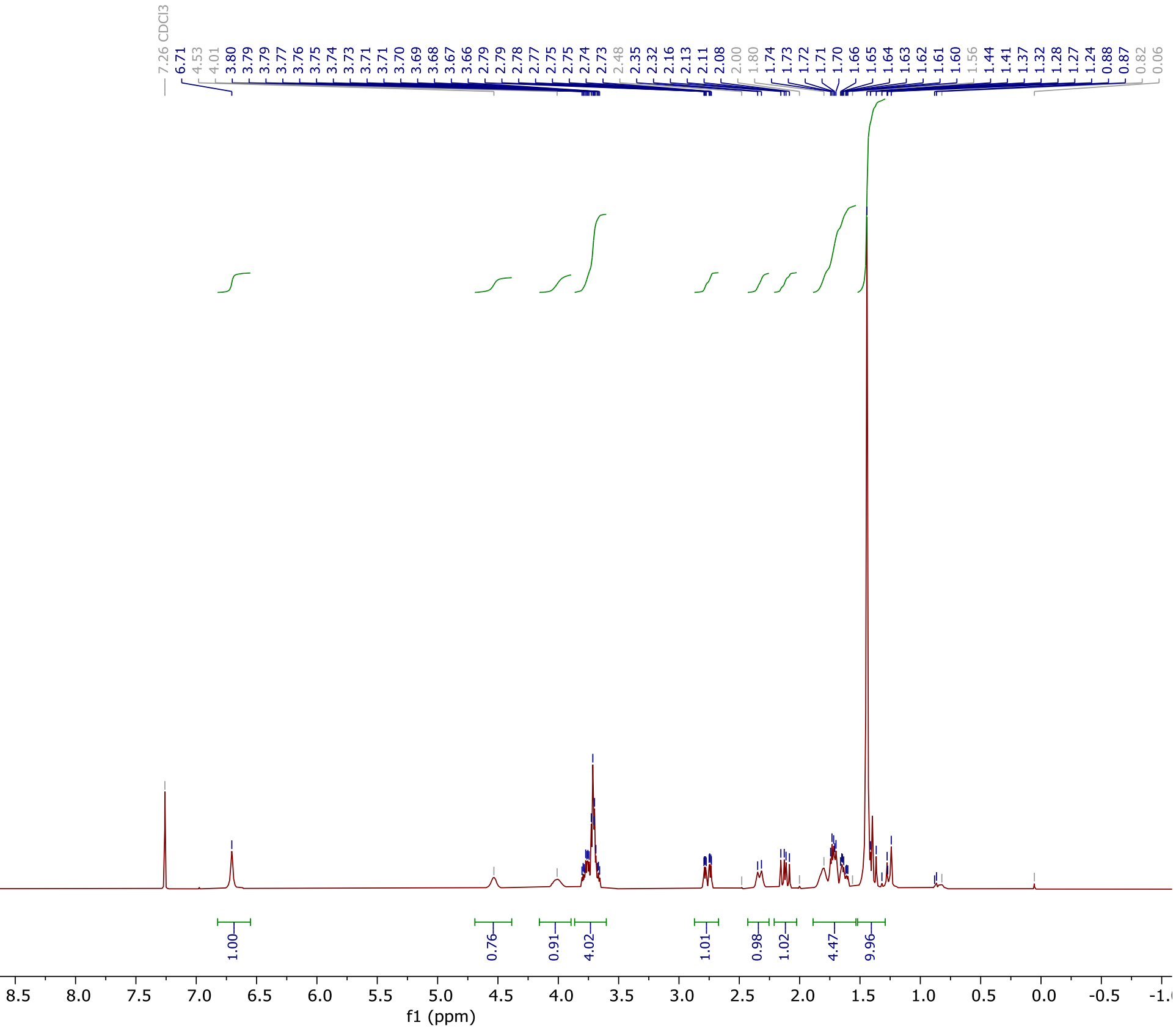


## $^{13}C$ NMR (101 MHz, $CDCl_3$)

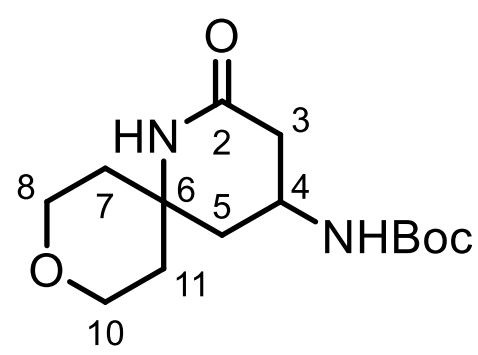


**10**

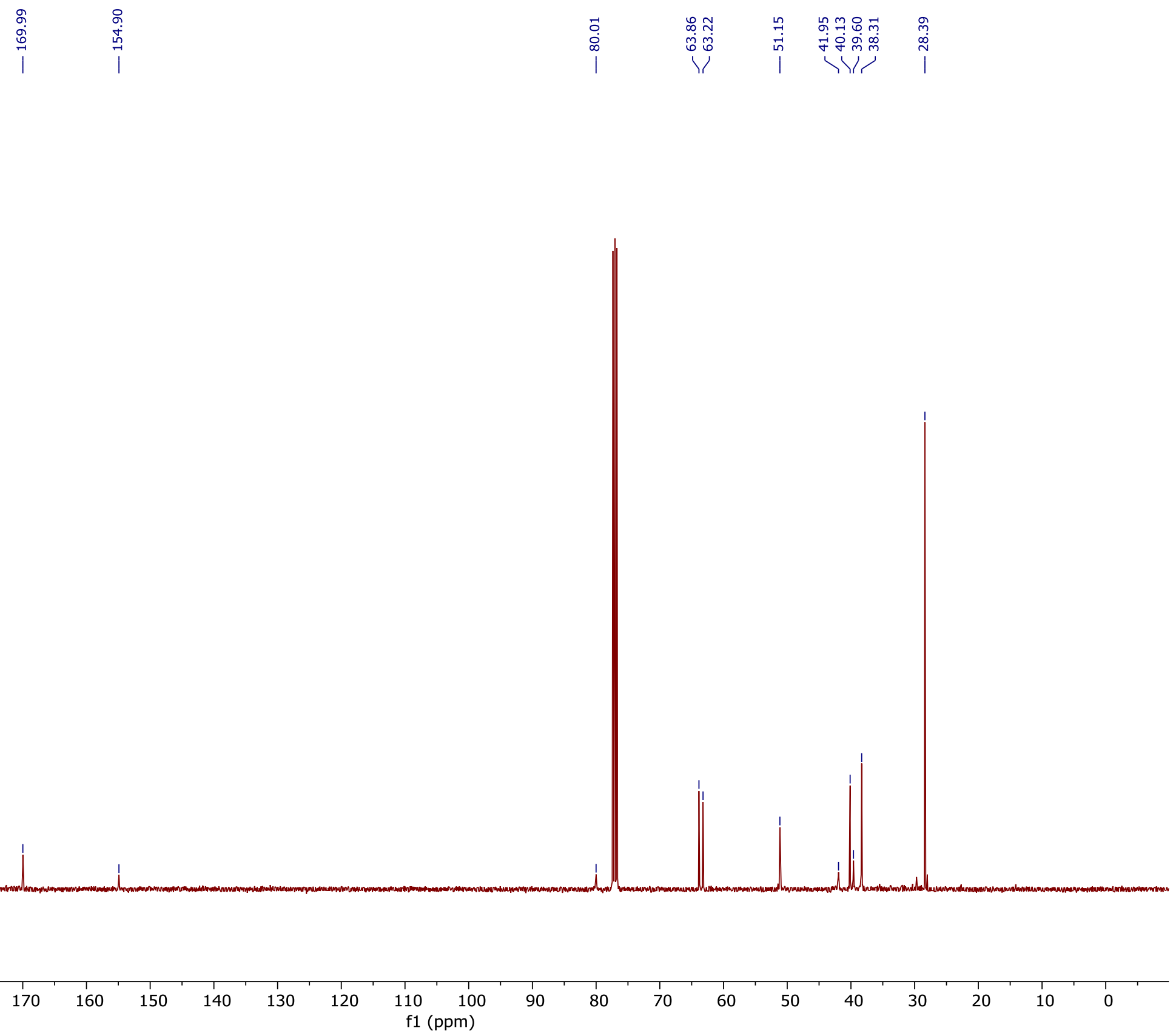

**COSY (500 MHz, CDCl$_3$)**

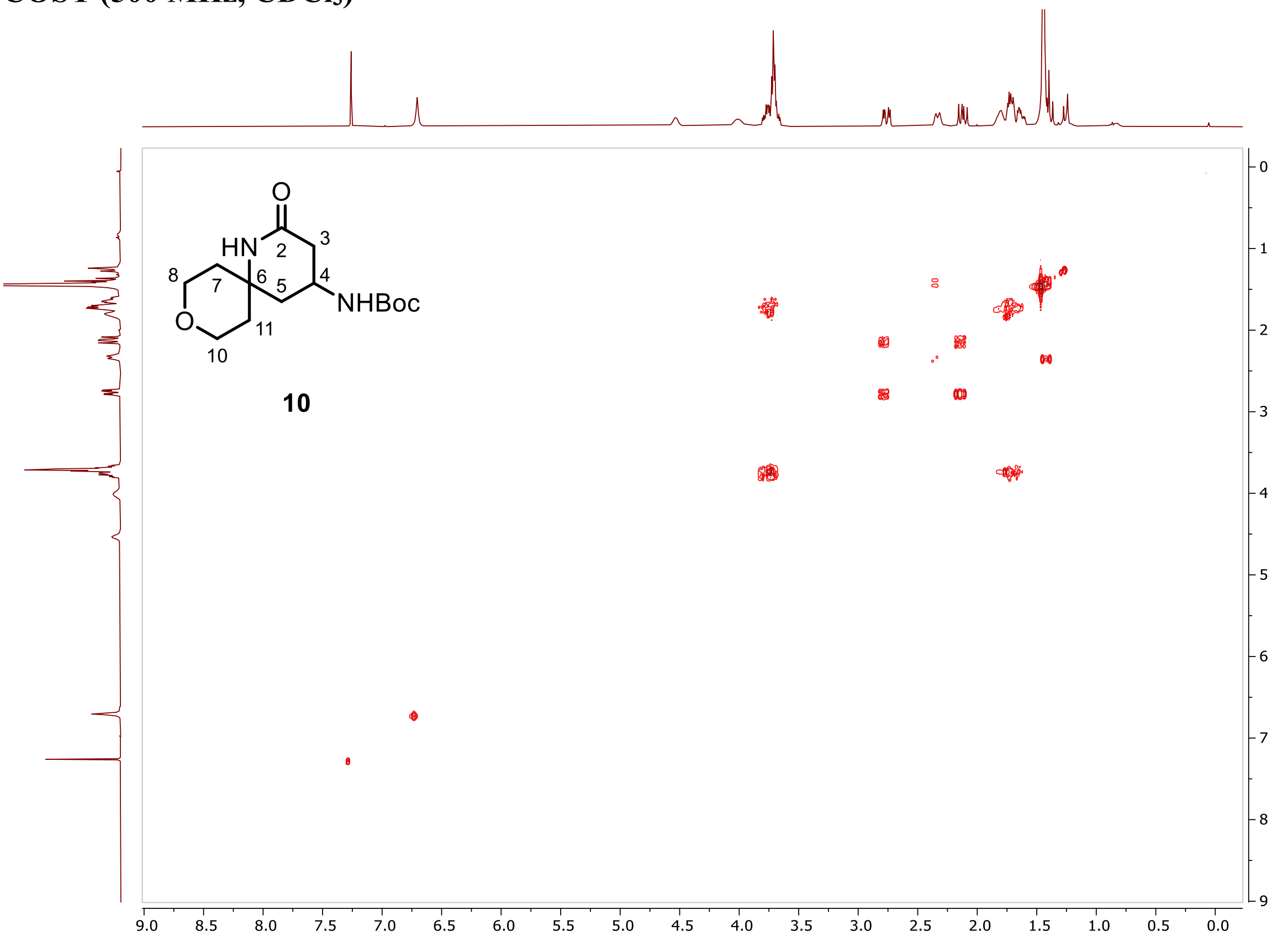


**HSQC ($^1$H 500 MHz/$^{13}$C 126 MHz, CDCl$_3$)**

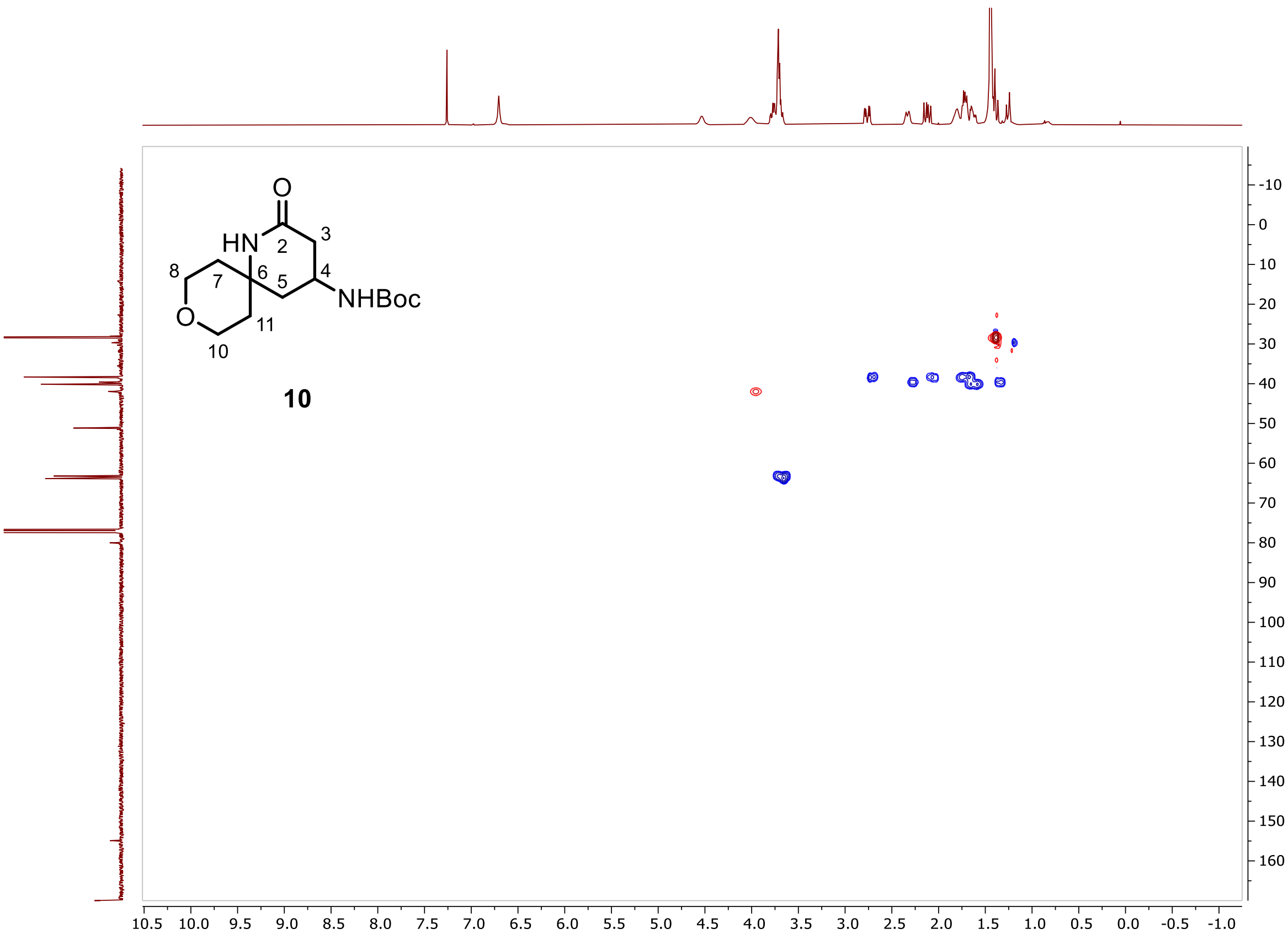

## $^1$H NMR (400 MHz, $CDCl_3$)

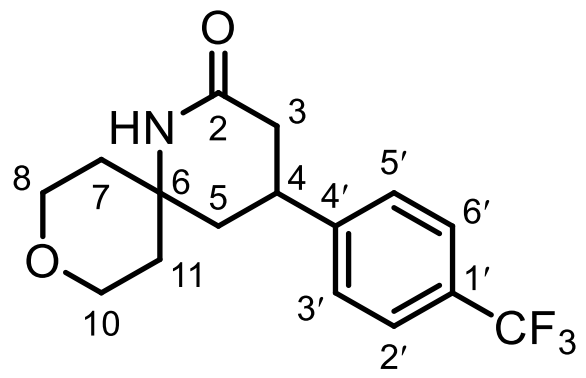

11

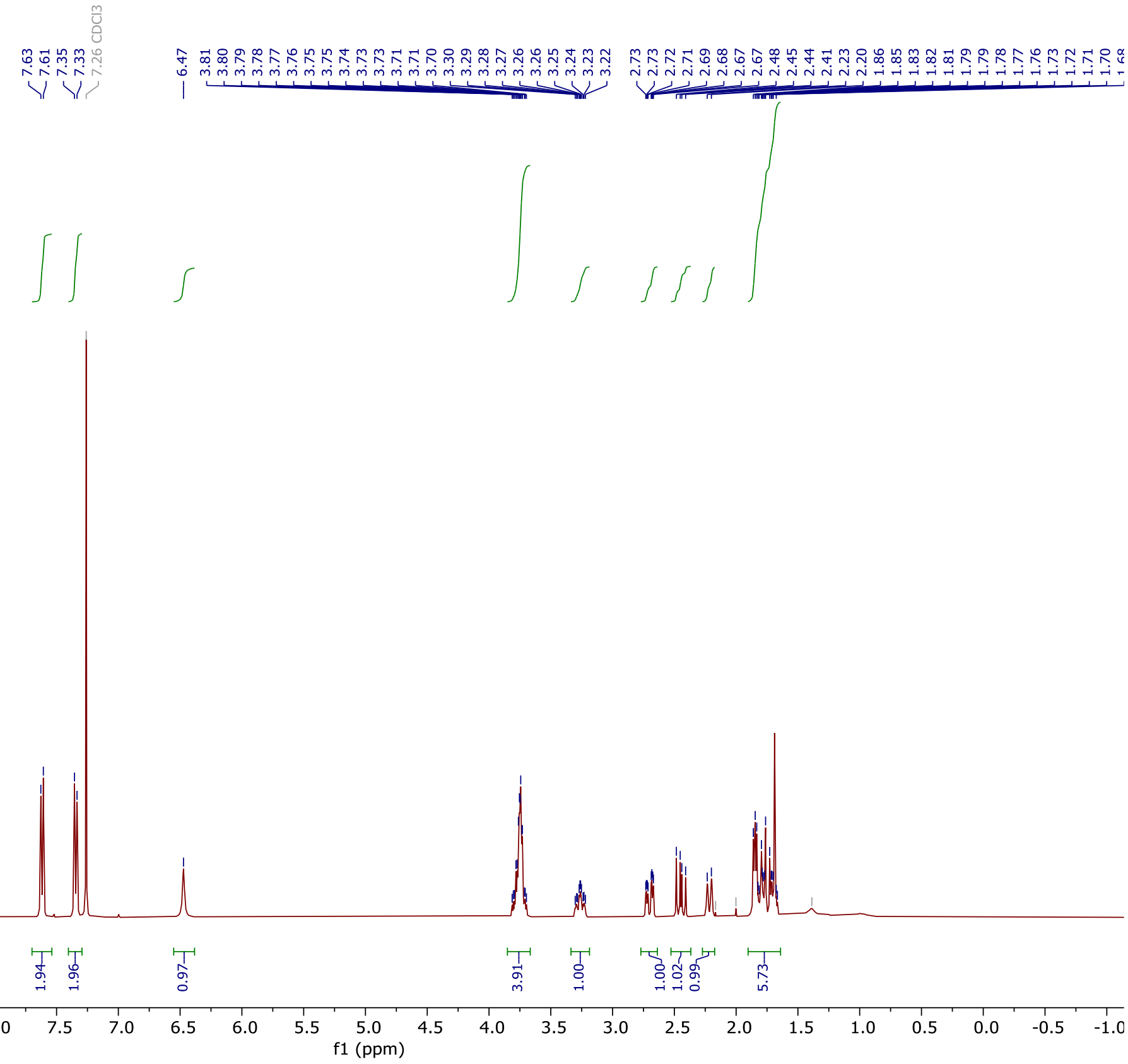

## $^{13}$C NMR (101 MHz, $CDCl_3$)

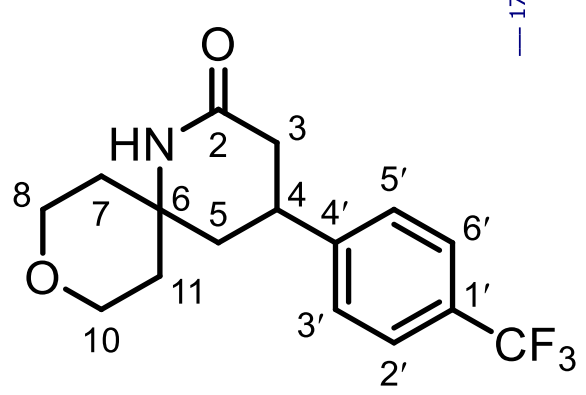

11

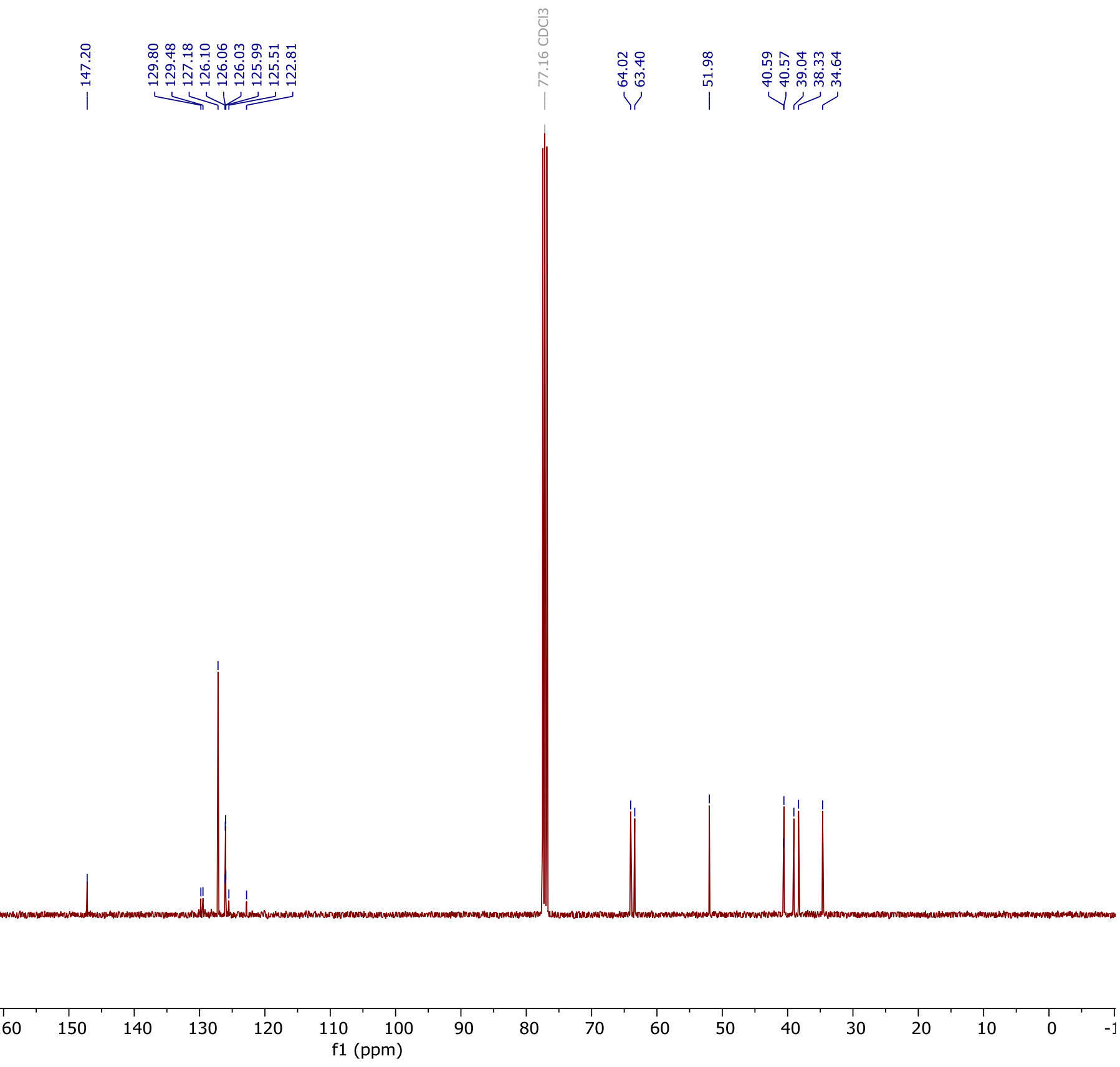

**COSY (500 MHz, CDCl$_3$)**

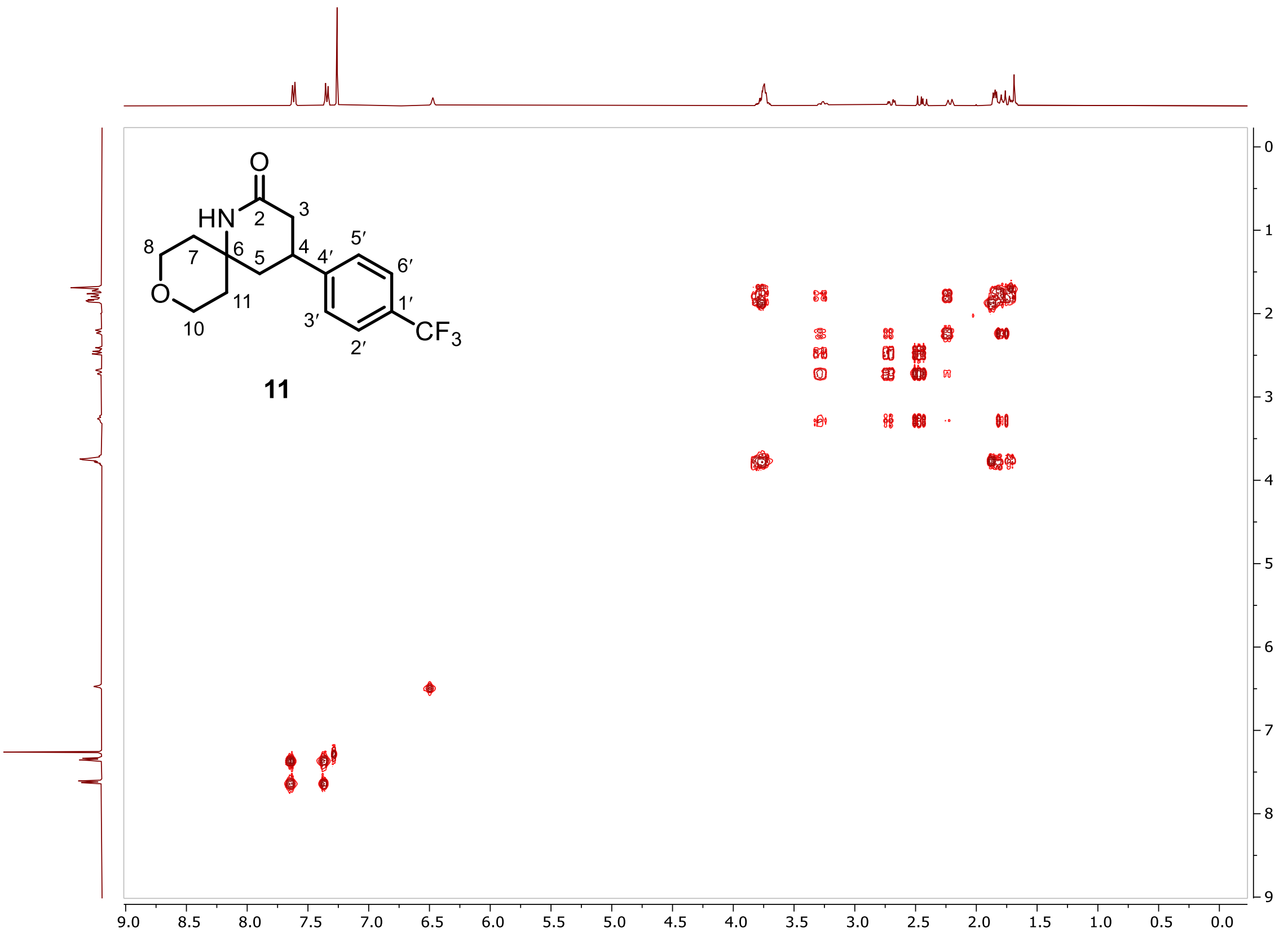

**HSQC ($^1$H 500 MHz/$^{13}$C 126 MHz, CDCl$_3$)**

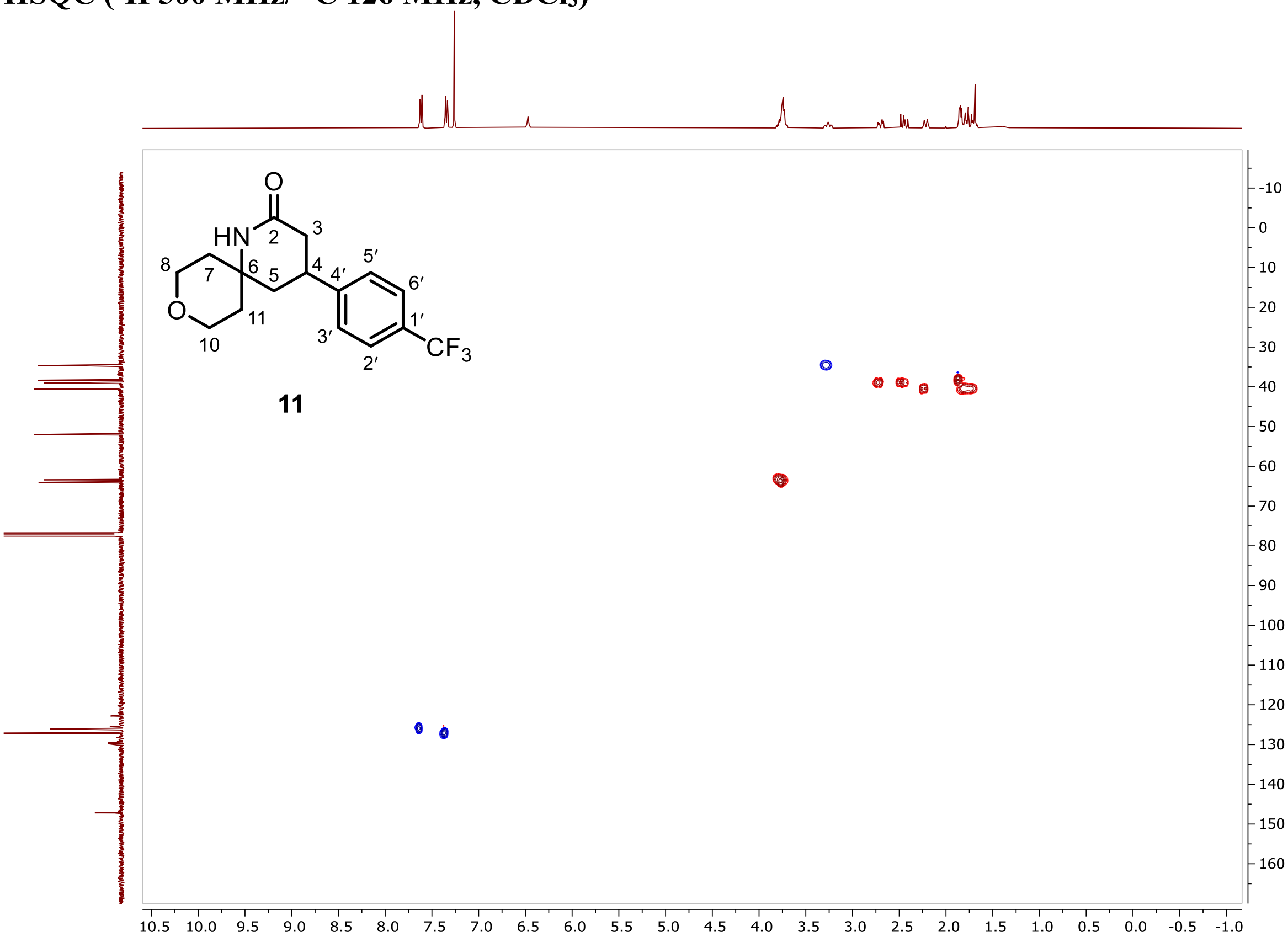

$^{1}$H NMR (500 MHz, $CDCl_3$)

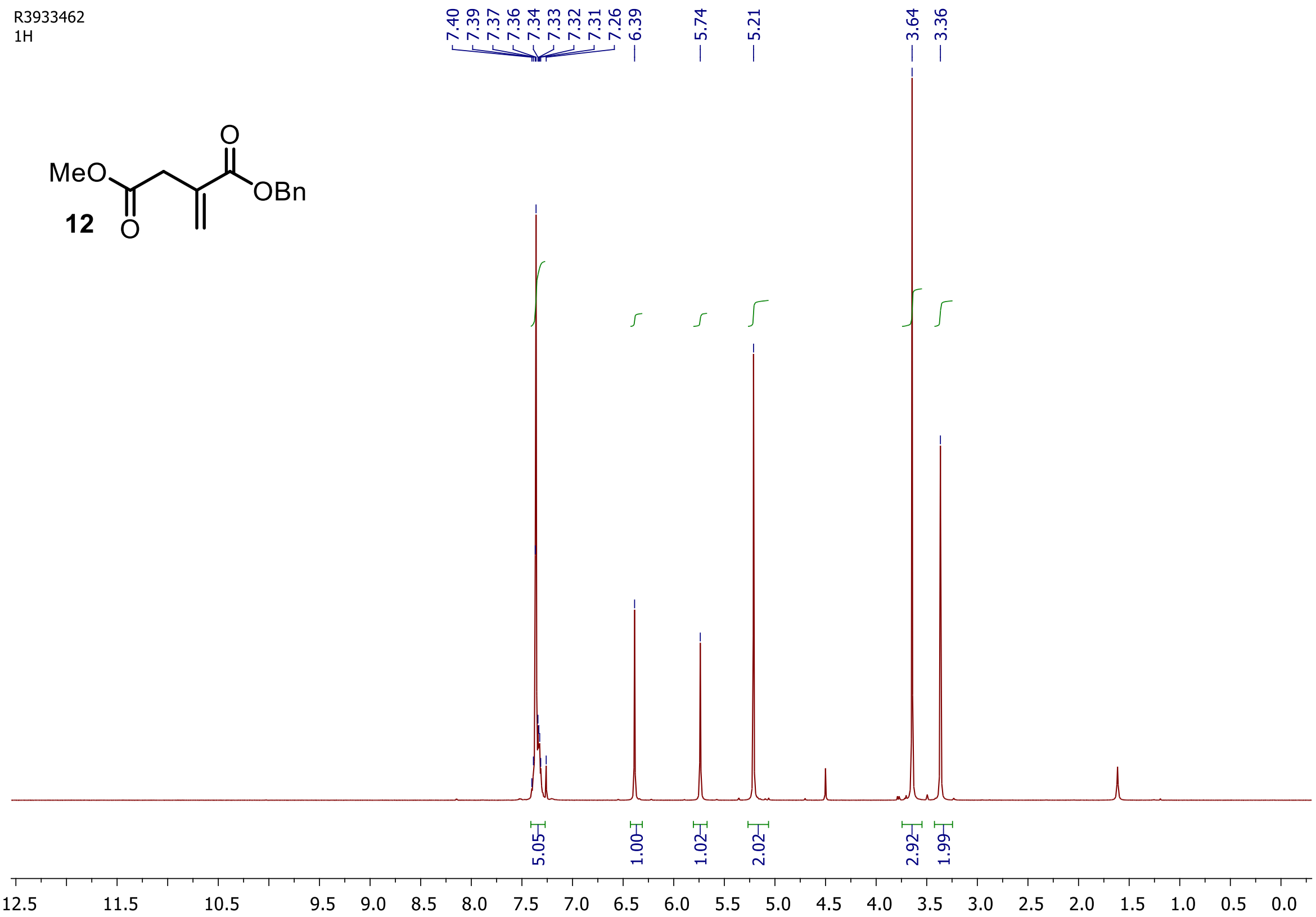


$^{13}$C NMR (126 MHz, $CDCl_3$)

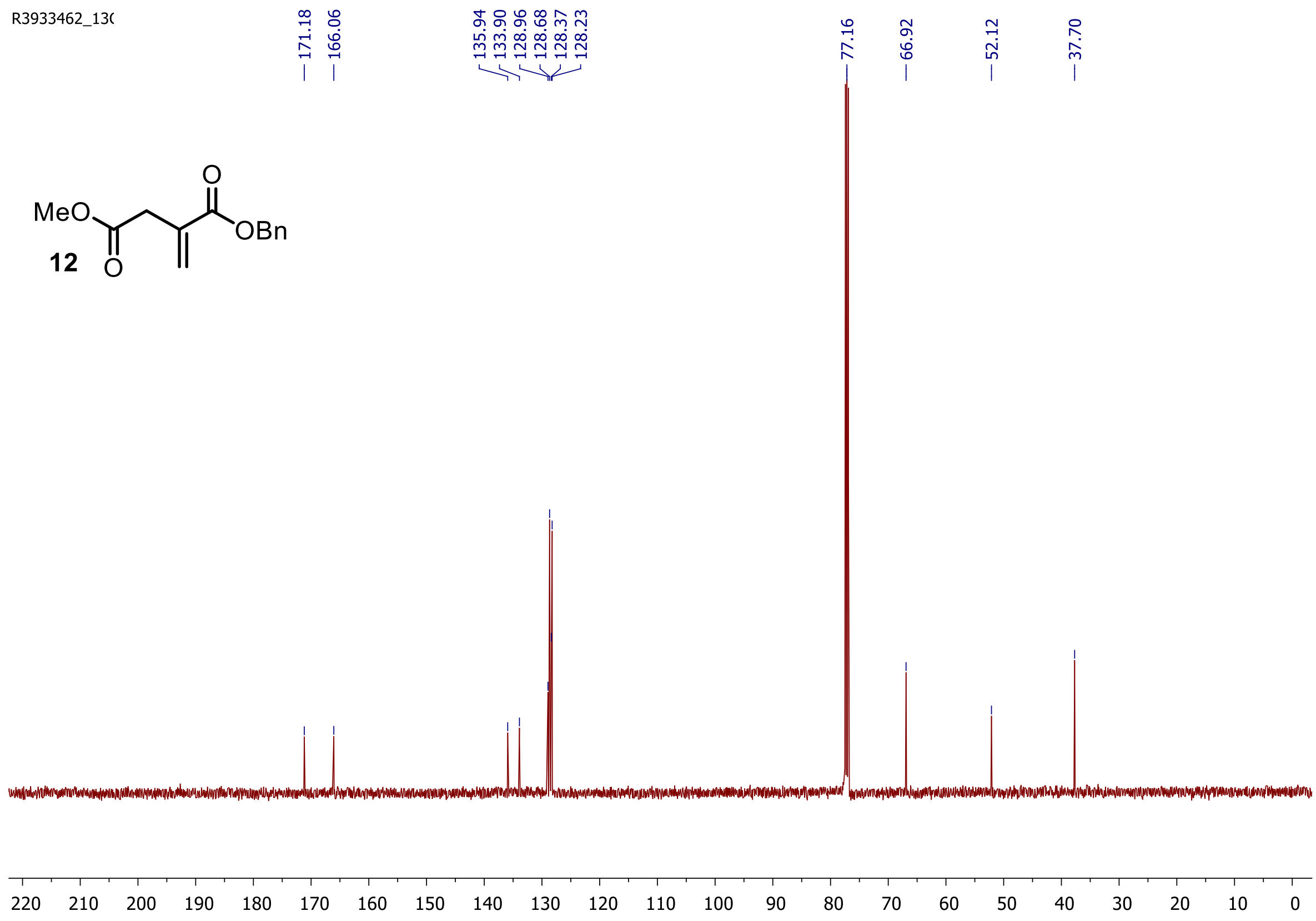

**$^{1}$H NMR (500 MHz, DMSO-$d_6$)**

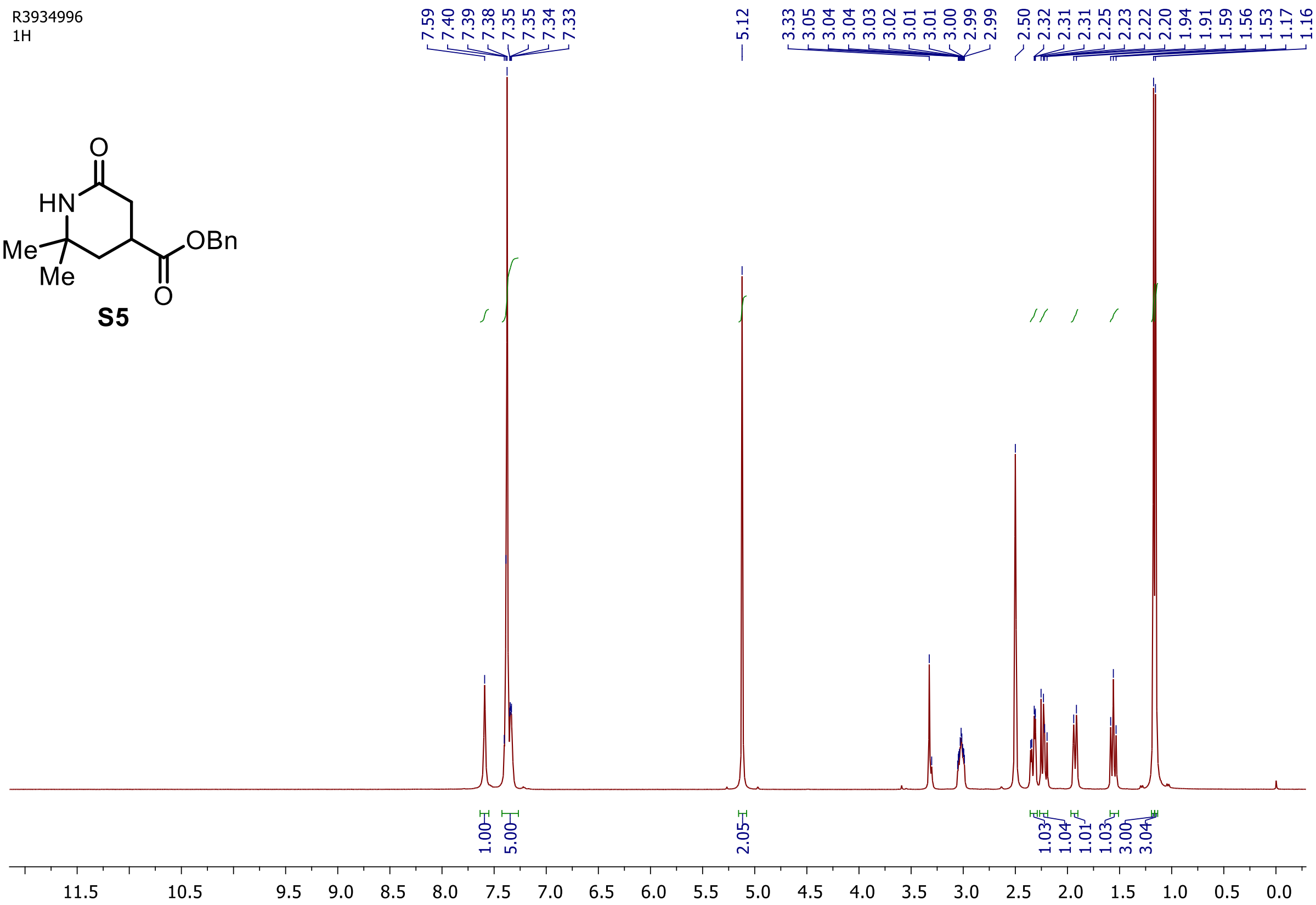


**$^{13}$C NMR (126 MHz, DMSO-$d_6$)**

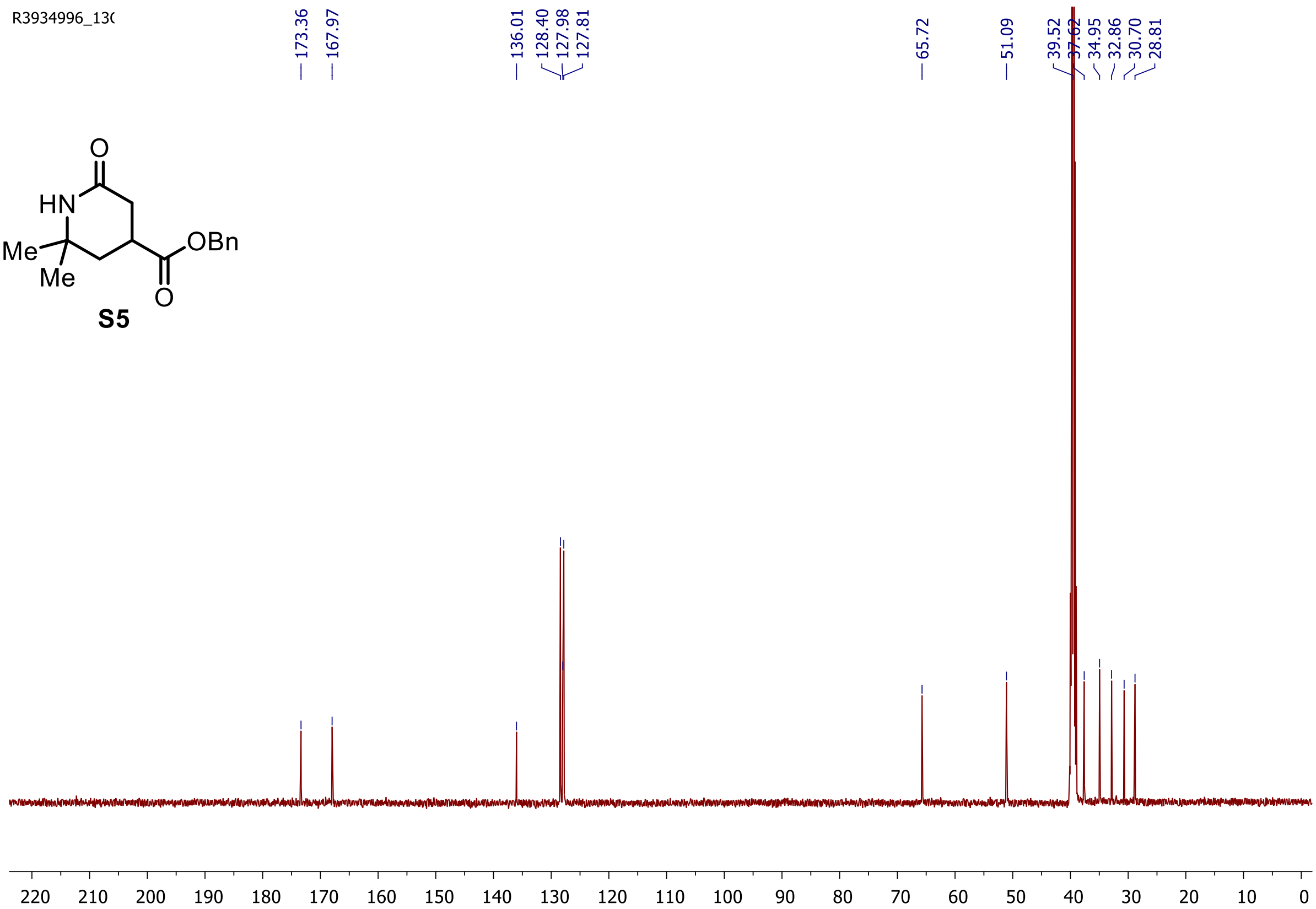

**$^{1}$H NMR (500 MHz, DMSO-$d_6$)**

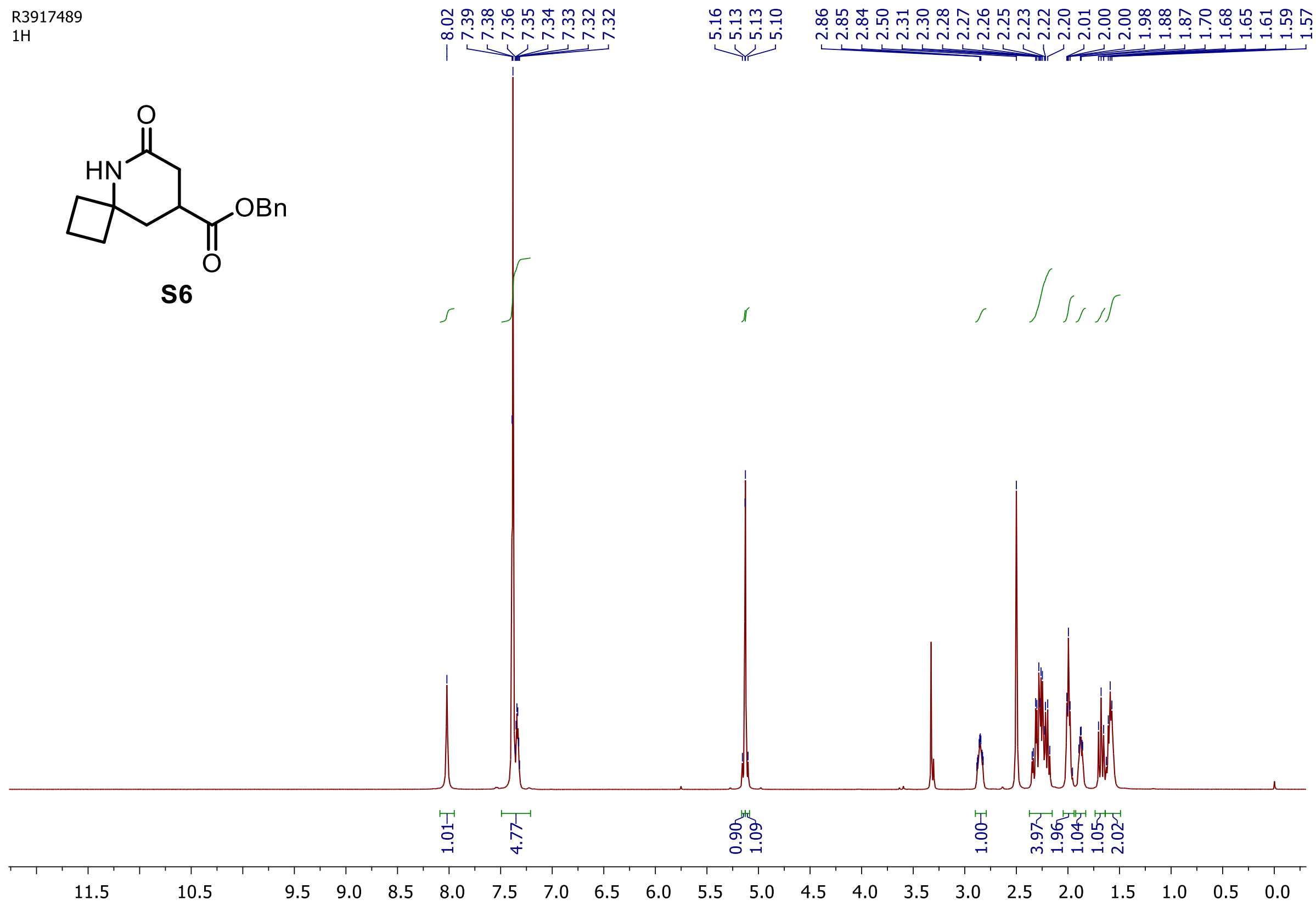


**$^{13}$C NMR (126 MHz, DMSO-$d_6$)**

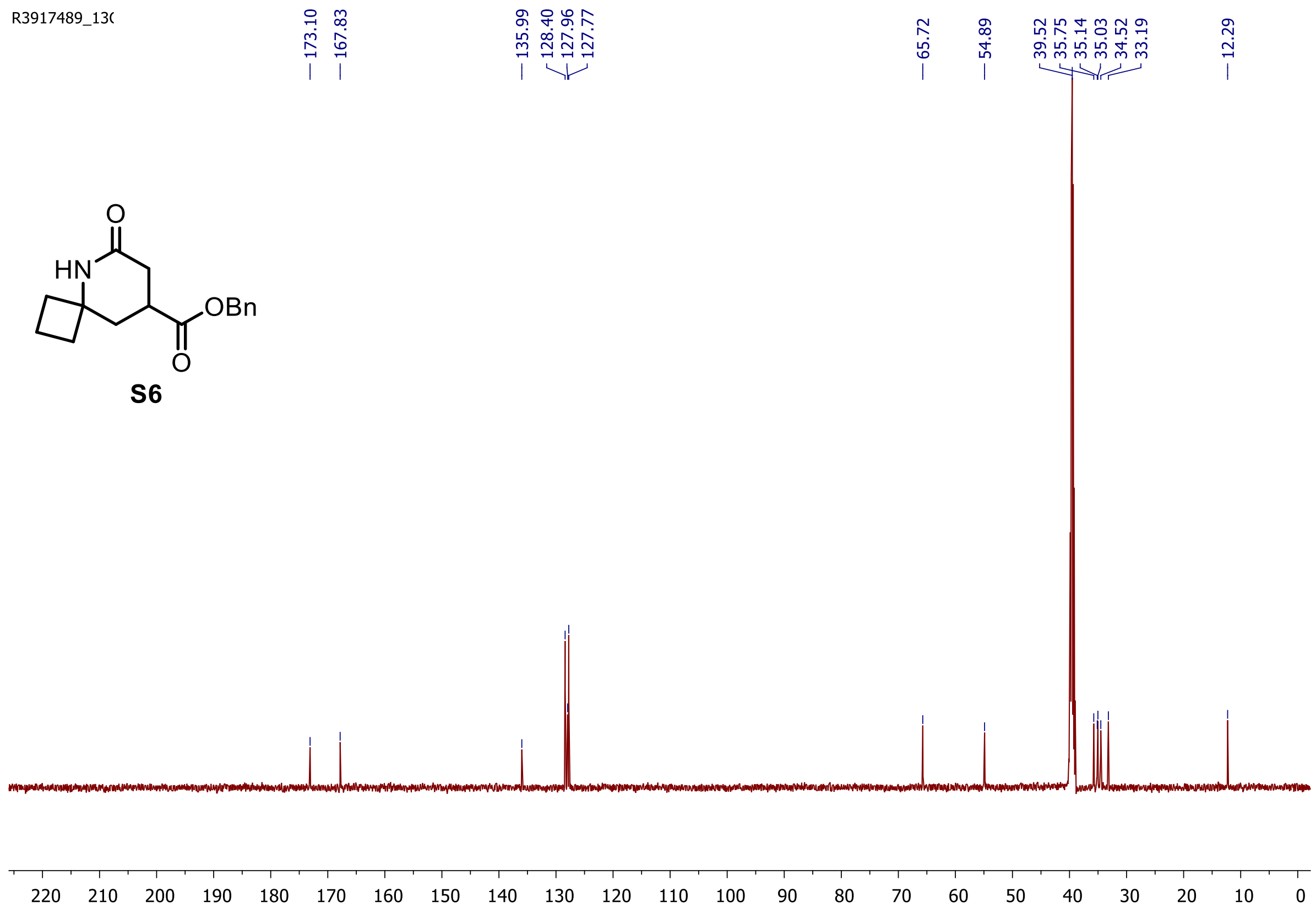

**$^{1}$H NMR (500 MHz, DMSO-$d_6$)**

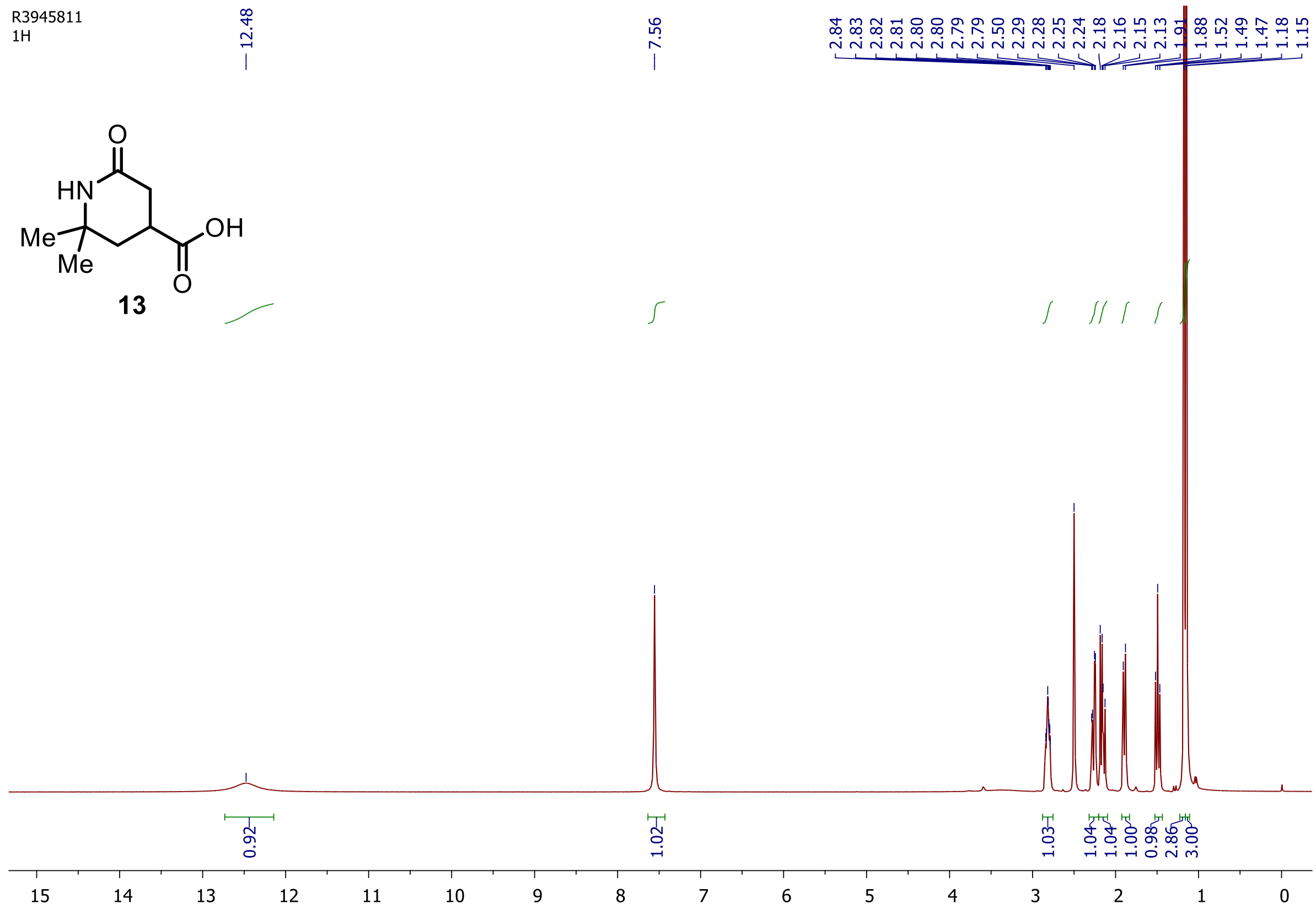

**$^{13}$C NMR (126 MHz, DMSO-$d_6$)**

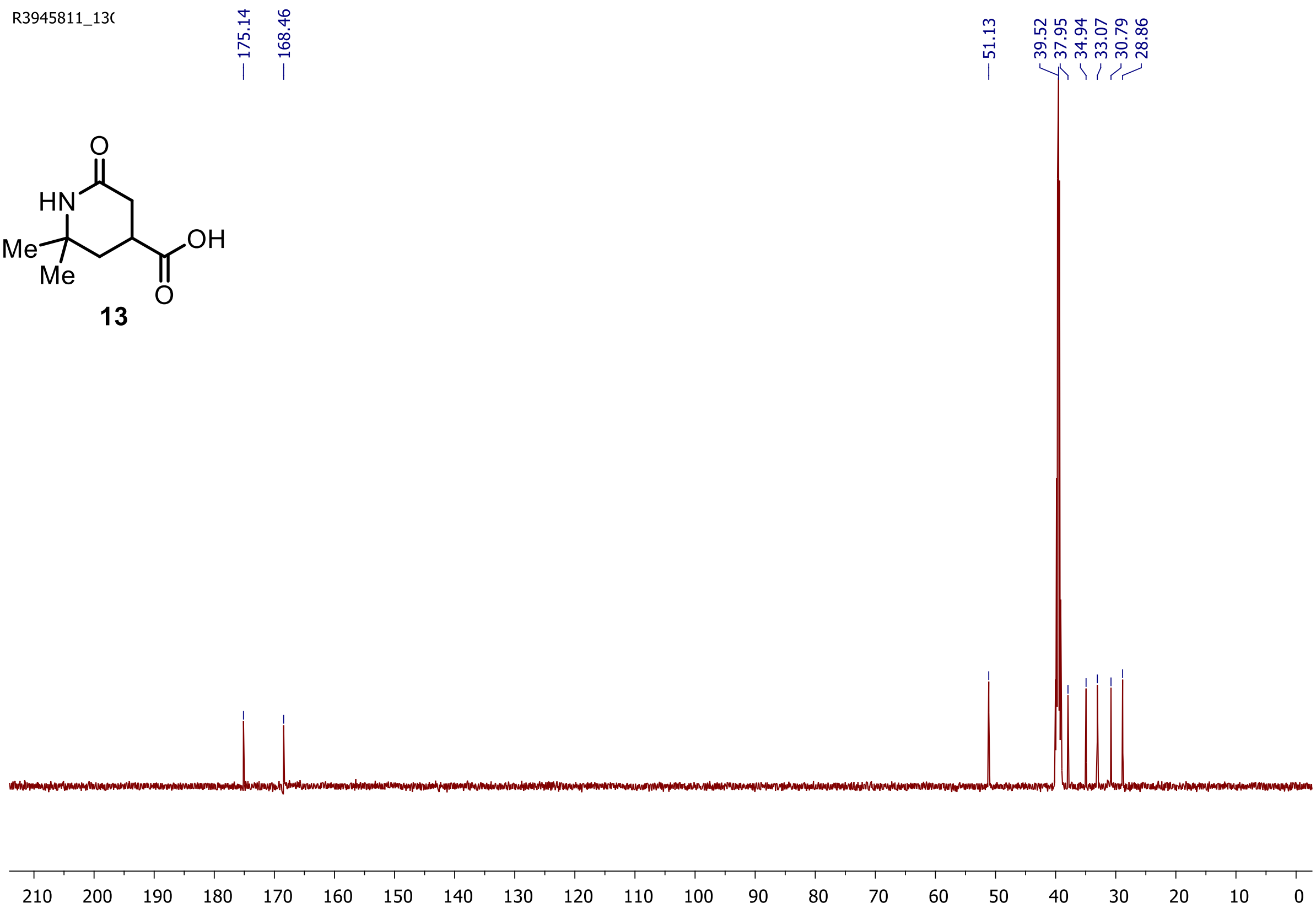

$^{1}$H NMR (500 MHz, DMSO-$d_6$)

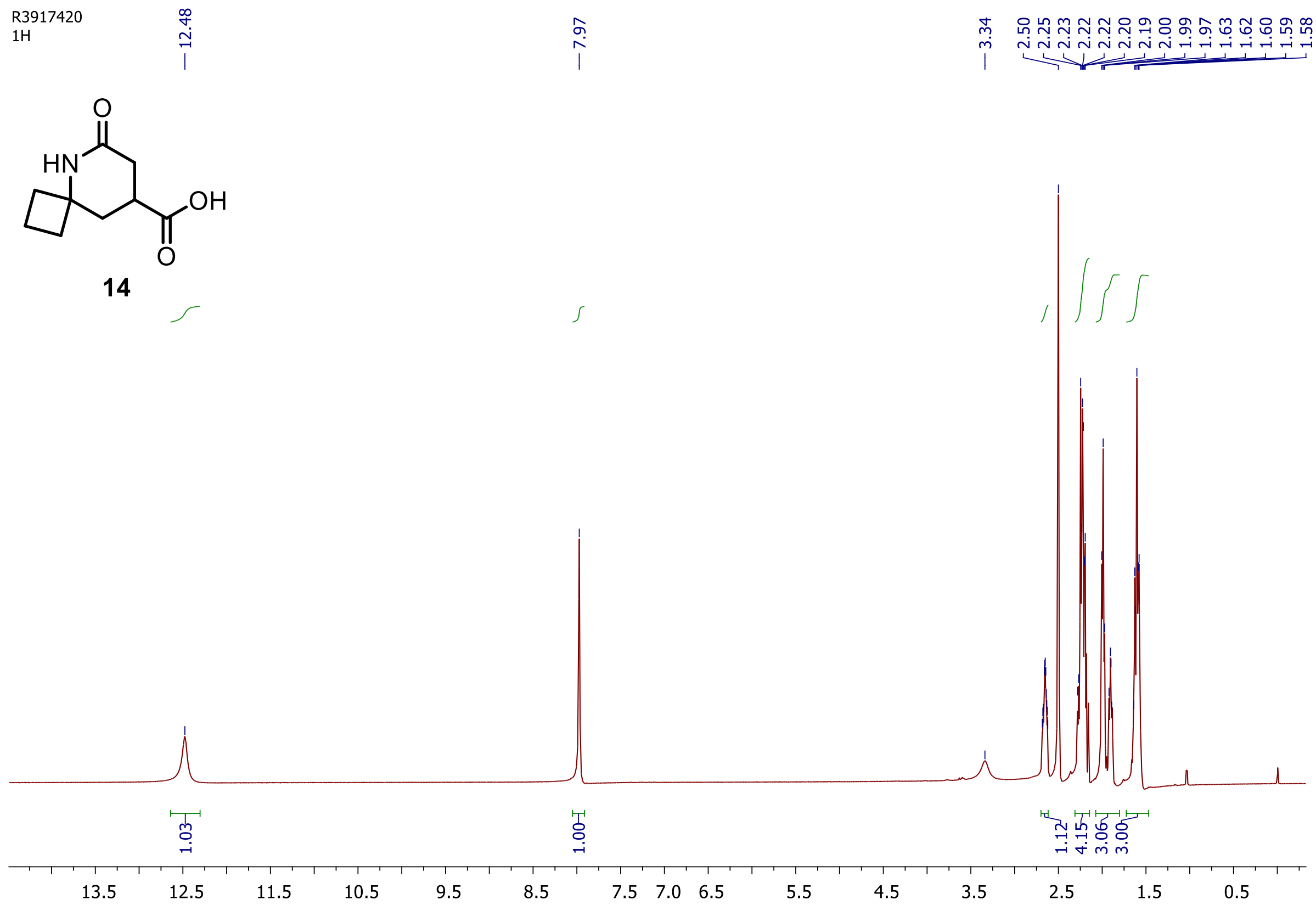


$^{13}$C NMR (126 MHz, DMSO-$d_6$)

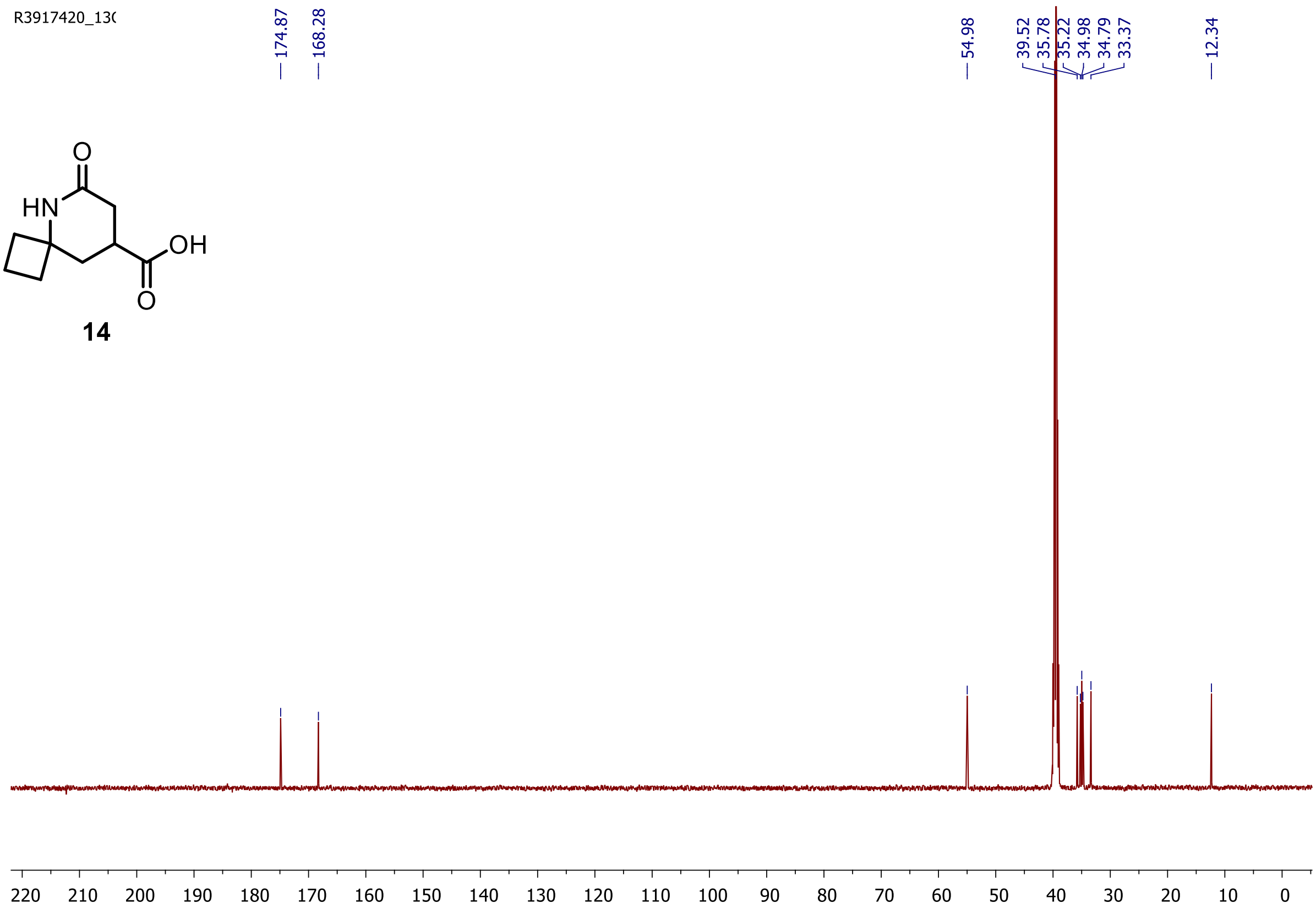

**$^{1}$H NMR (500 MHz, CDCl$_3$)**

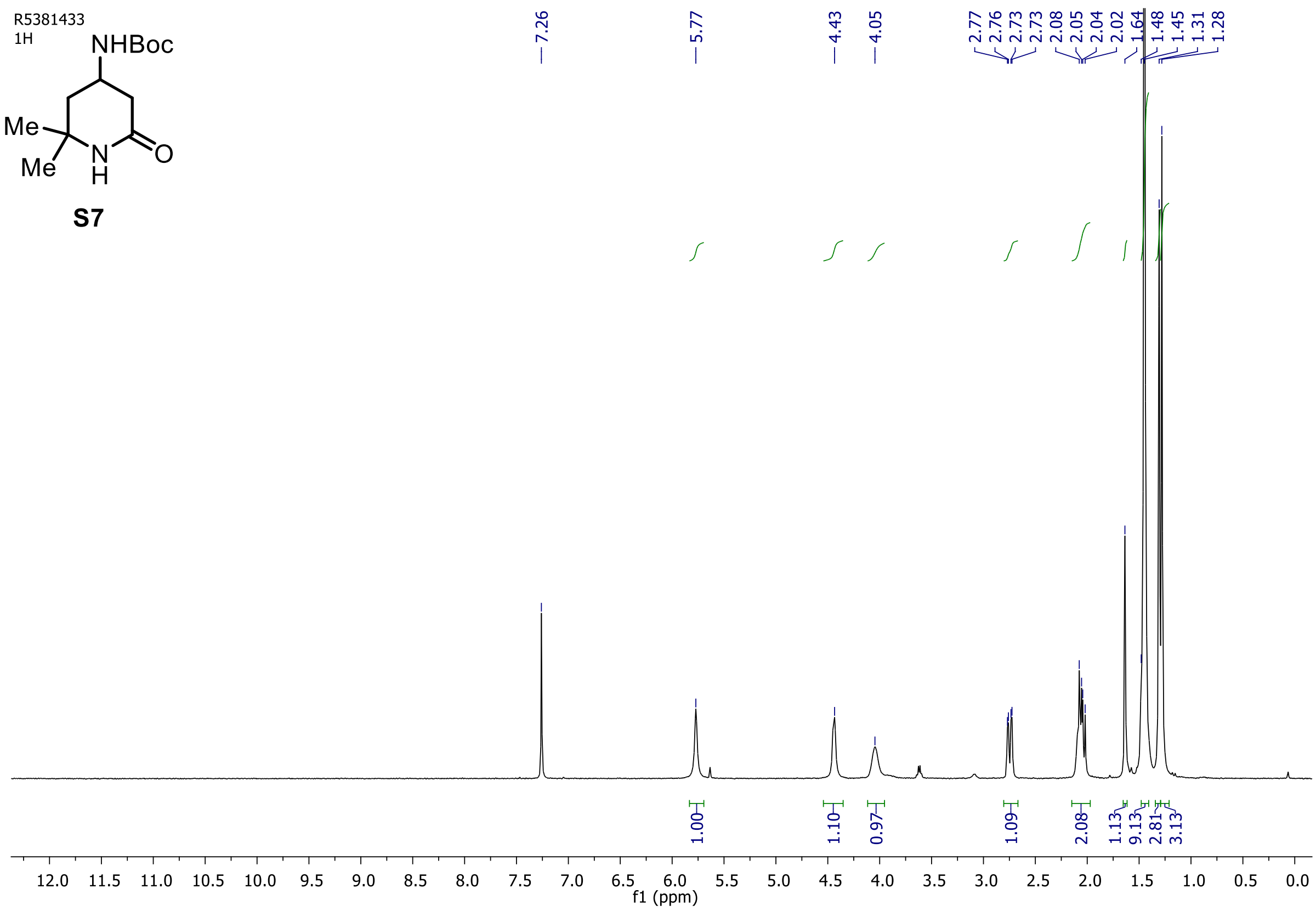


**$^{13}$C NMR (126 MHz, CDCl$_3$)**

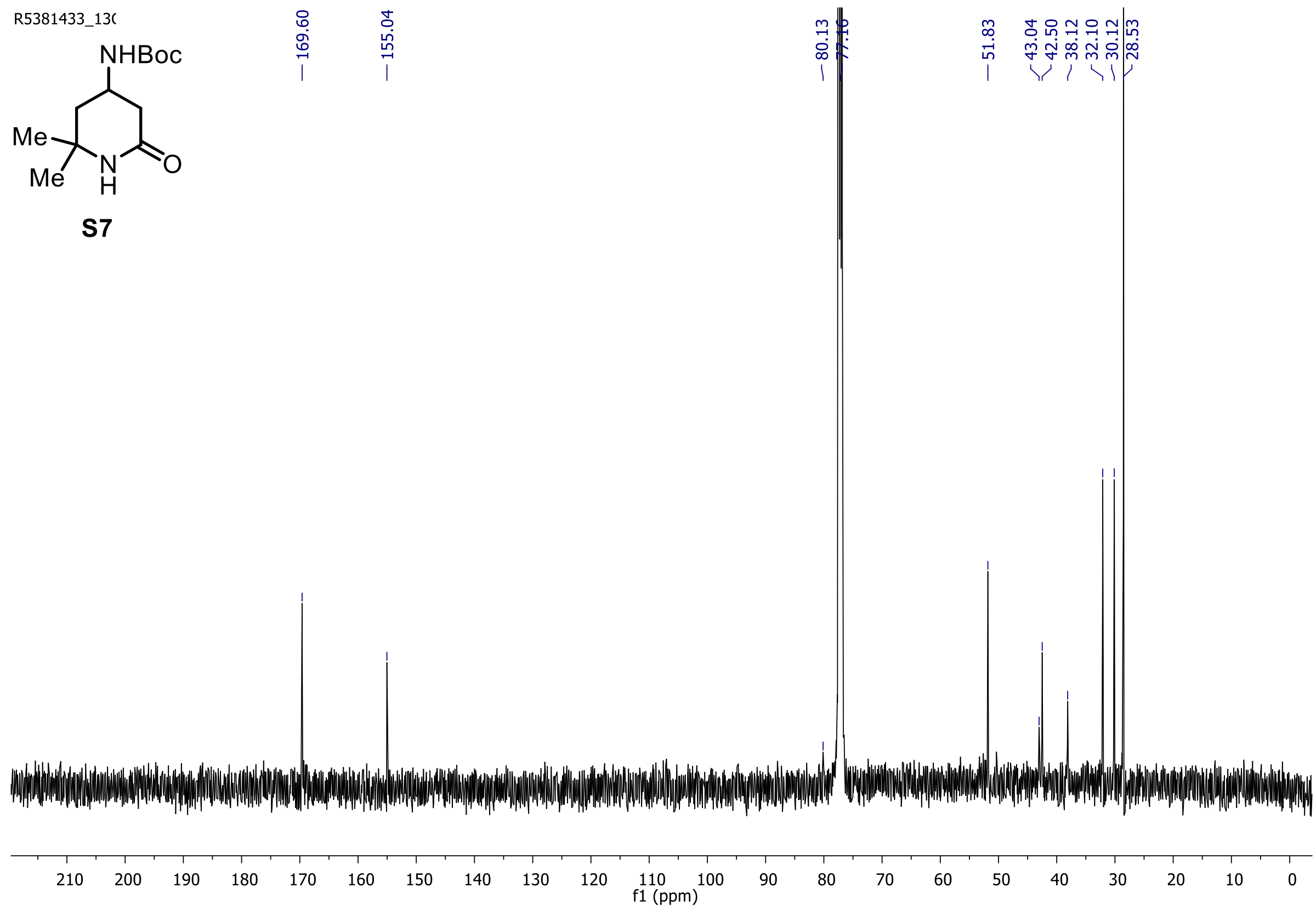

**$^{1}$H NMR (500 MHz, DMSO-$d_6$)**

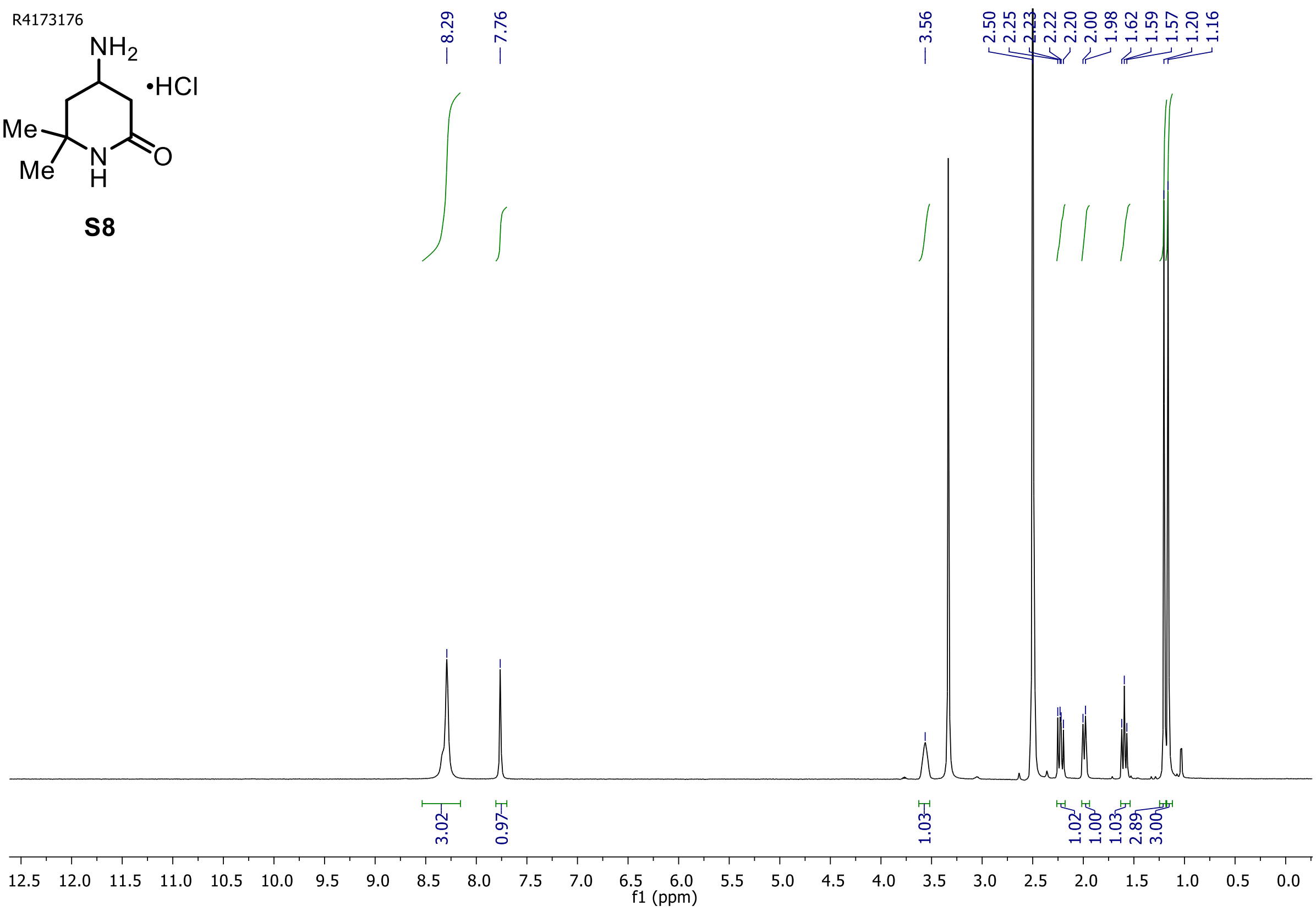


**$^{13}$C NMR (126 MHz, DMSO-$d_6$)**

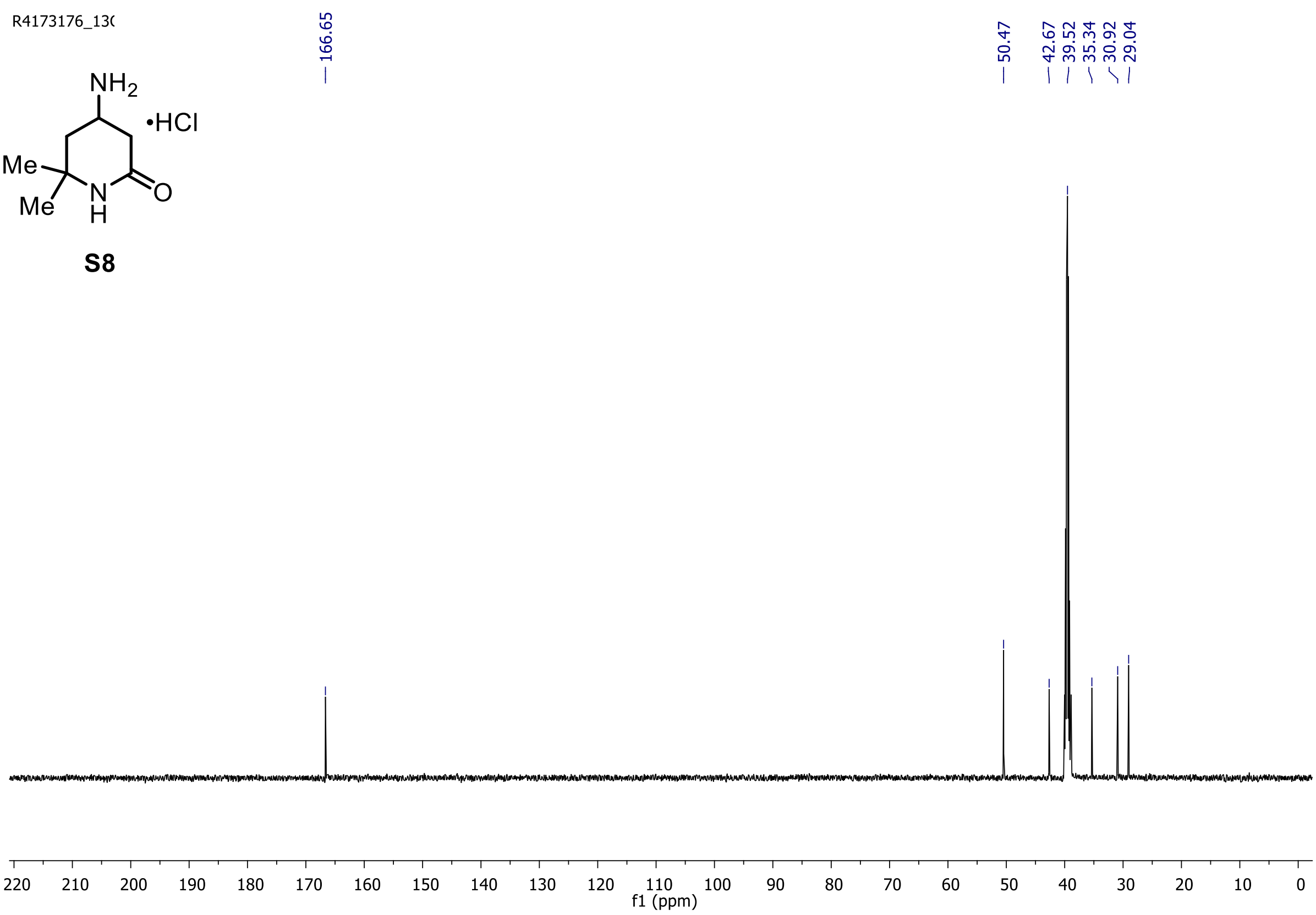

## $^{1}H$ NMR (500 MHz, $CDCl_3$)

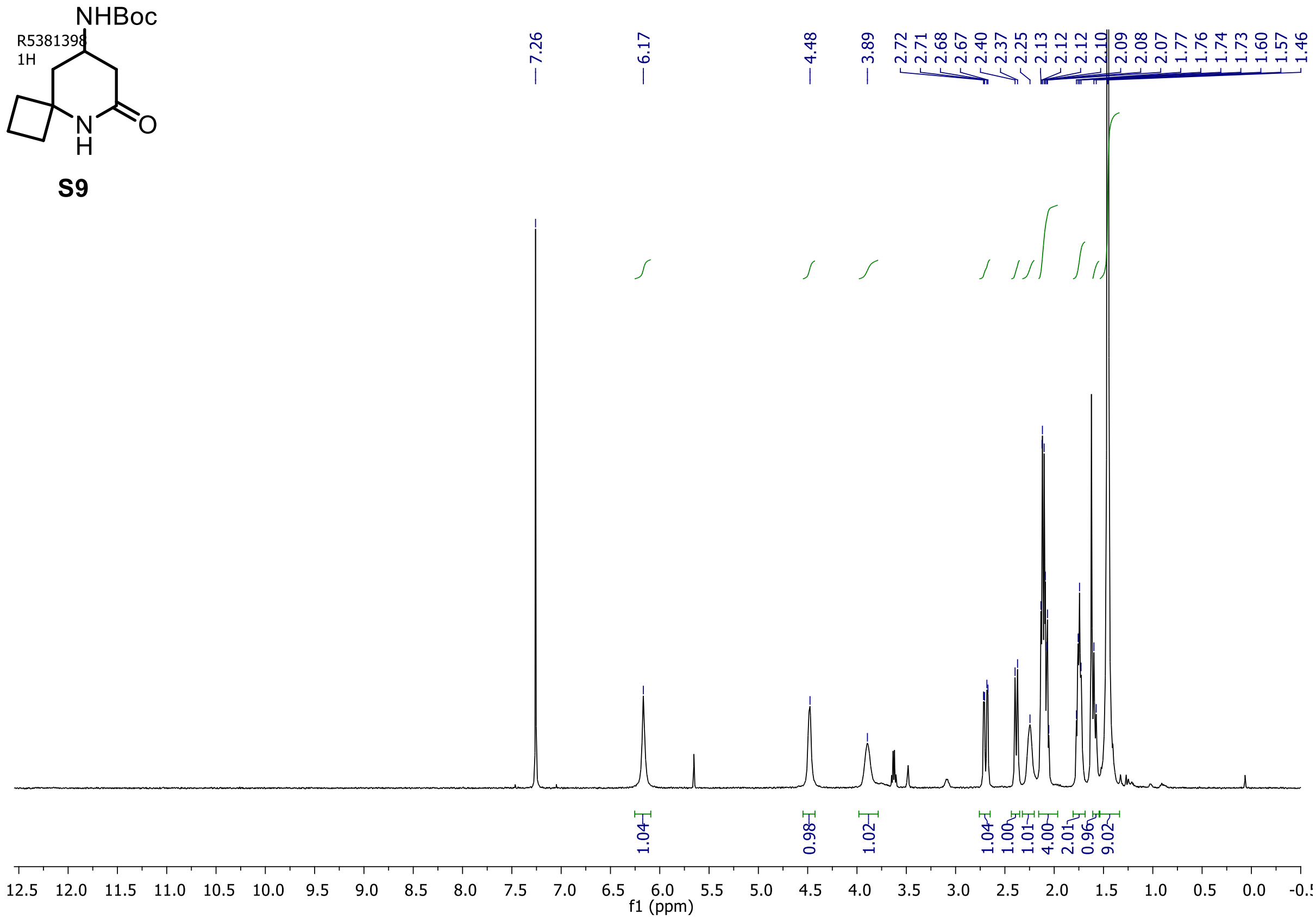


## $^{13}C$ NMR (126 MHz, $CDCl_3$)

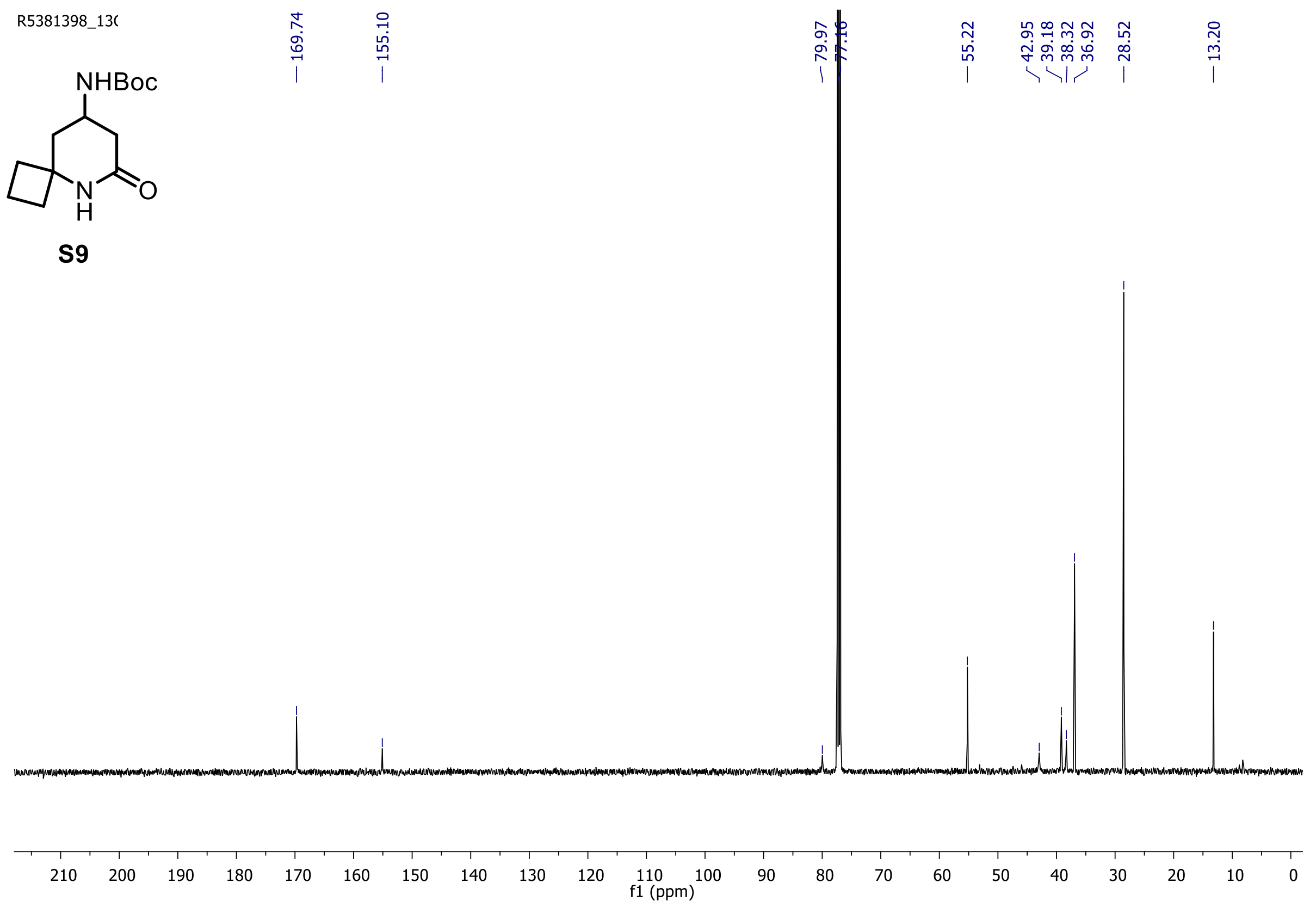

**$^{1}$H NMR (500 MHz, DMSO-$d_6$)**

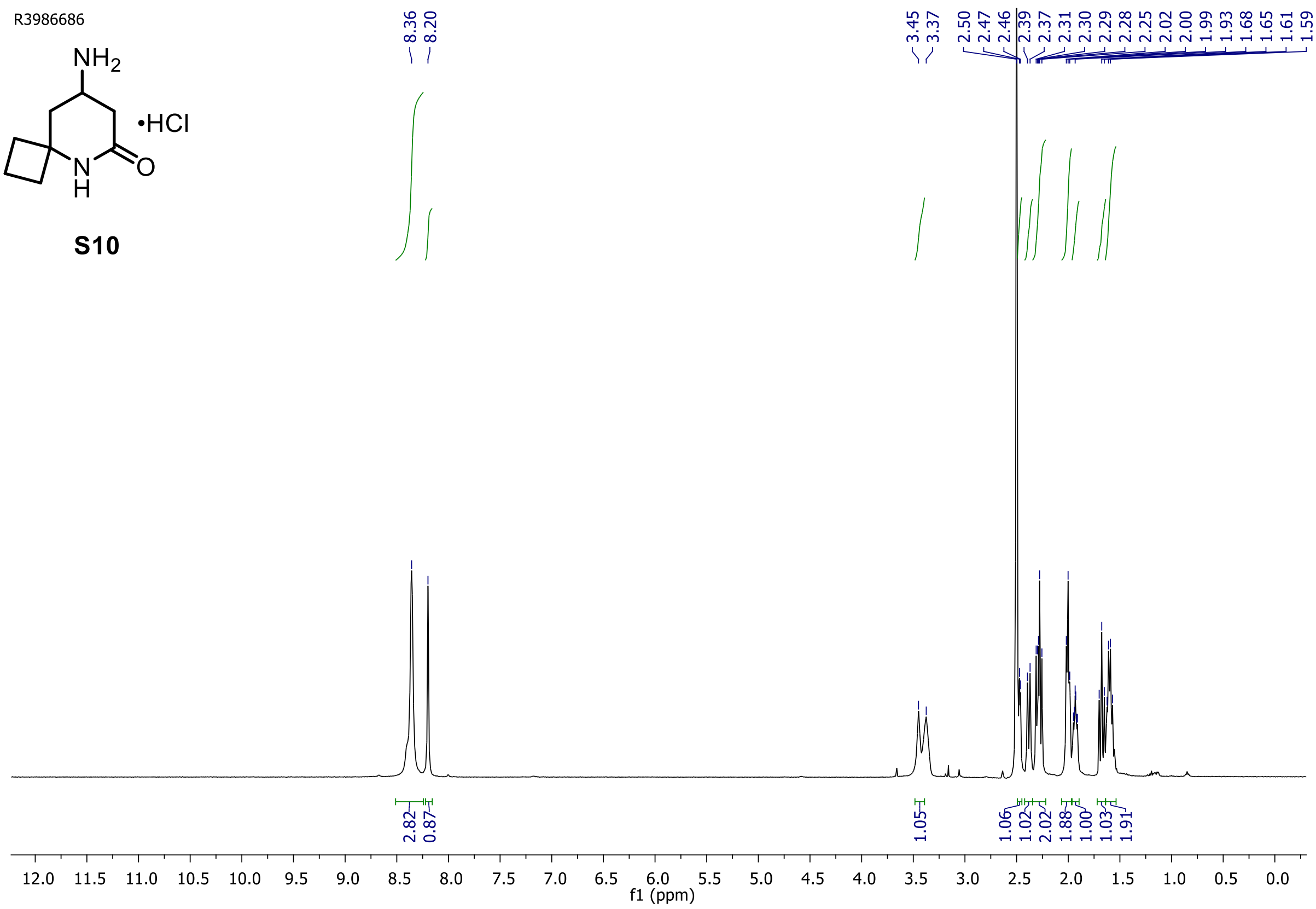


**$^{13}$C NMR (151 MHz, DMSO-$d_6$)**

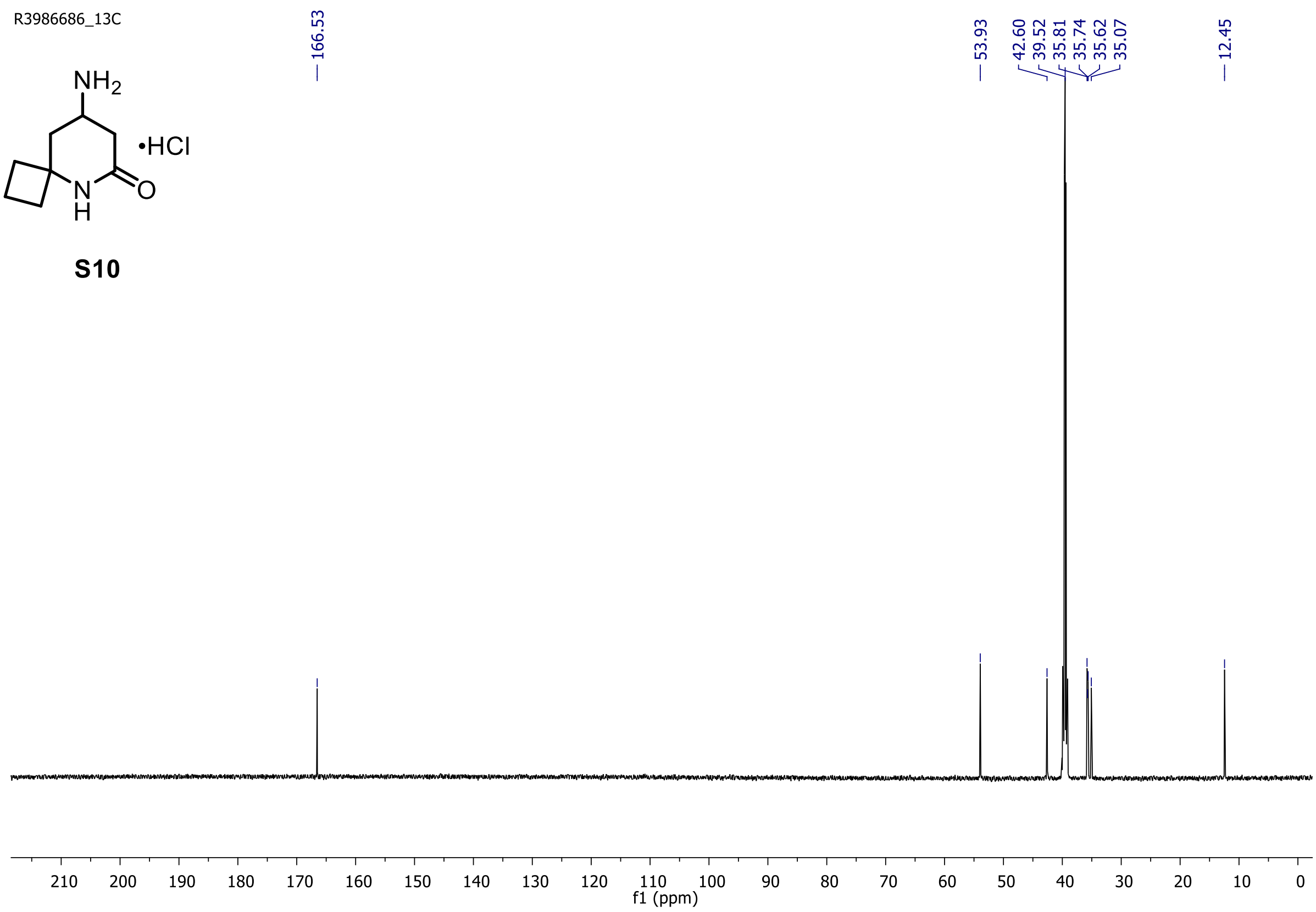

**$^1$H NMR (500 MHz, DMSO-$d_6$)**

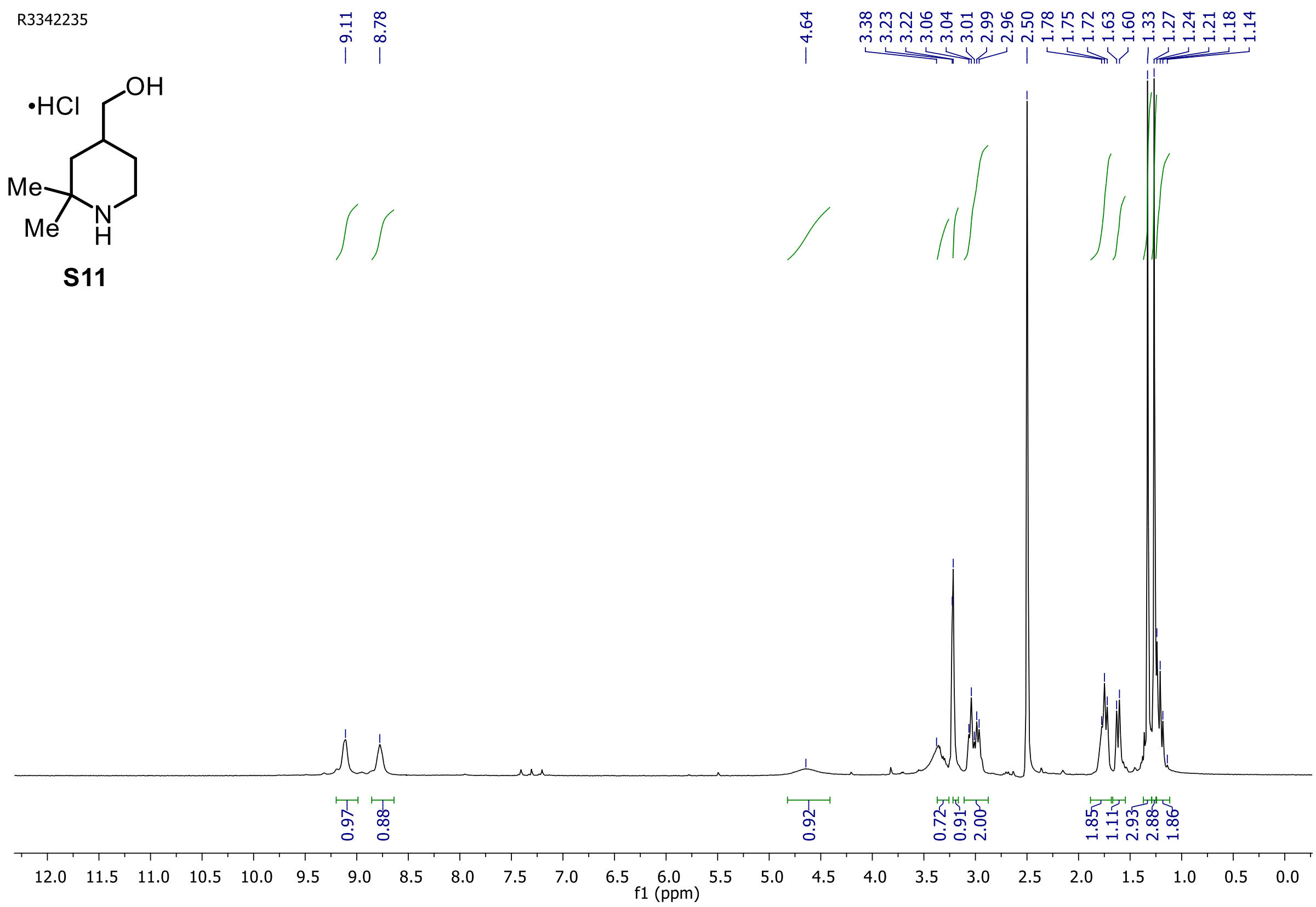

**$^{13}$C NMR (151 MHz, DMSO-$d_6$)**

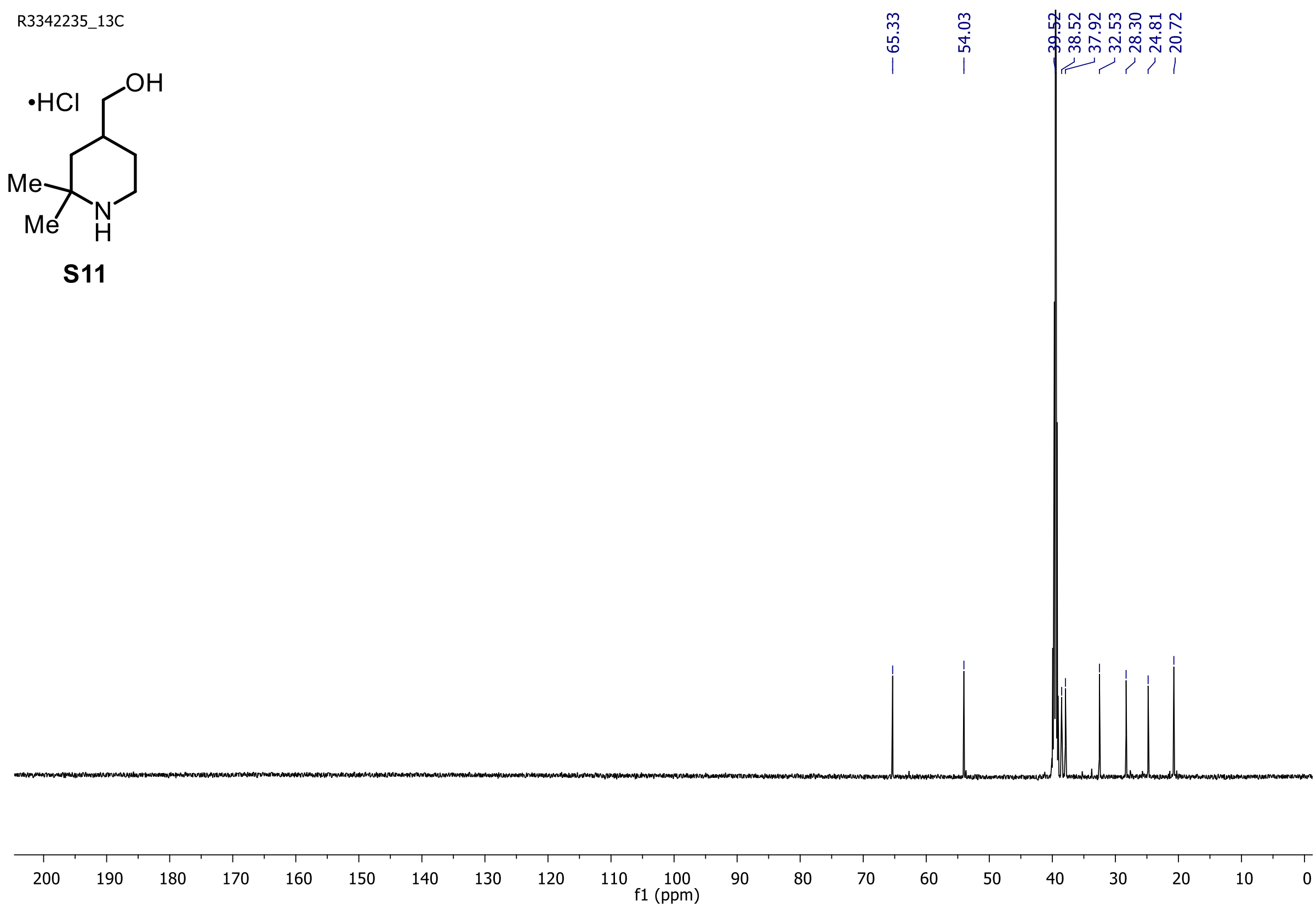

## $^{1}$H NMR (500 MHz, DMSO-$d_6$)

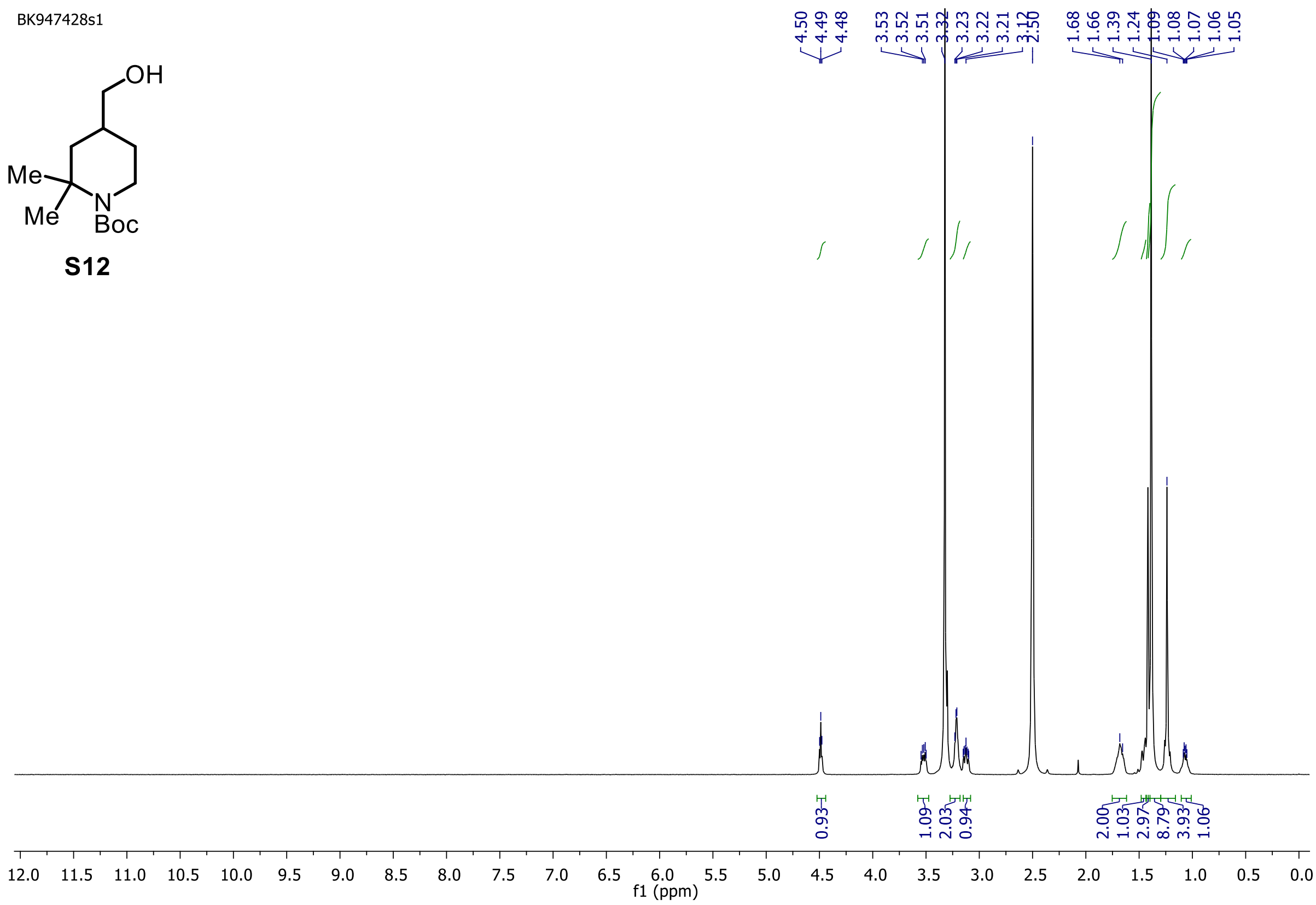


## $^{13}$C NMR (151 MHz, DMSO-$d_6$)

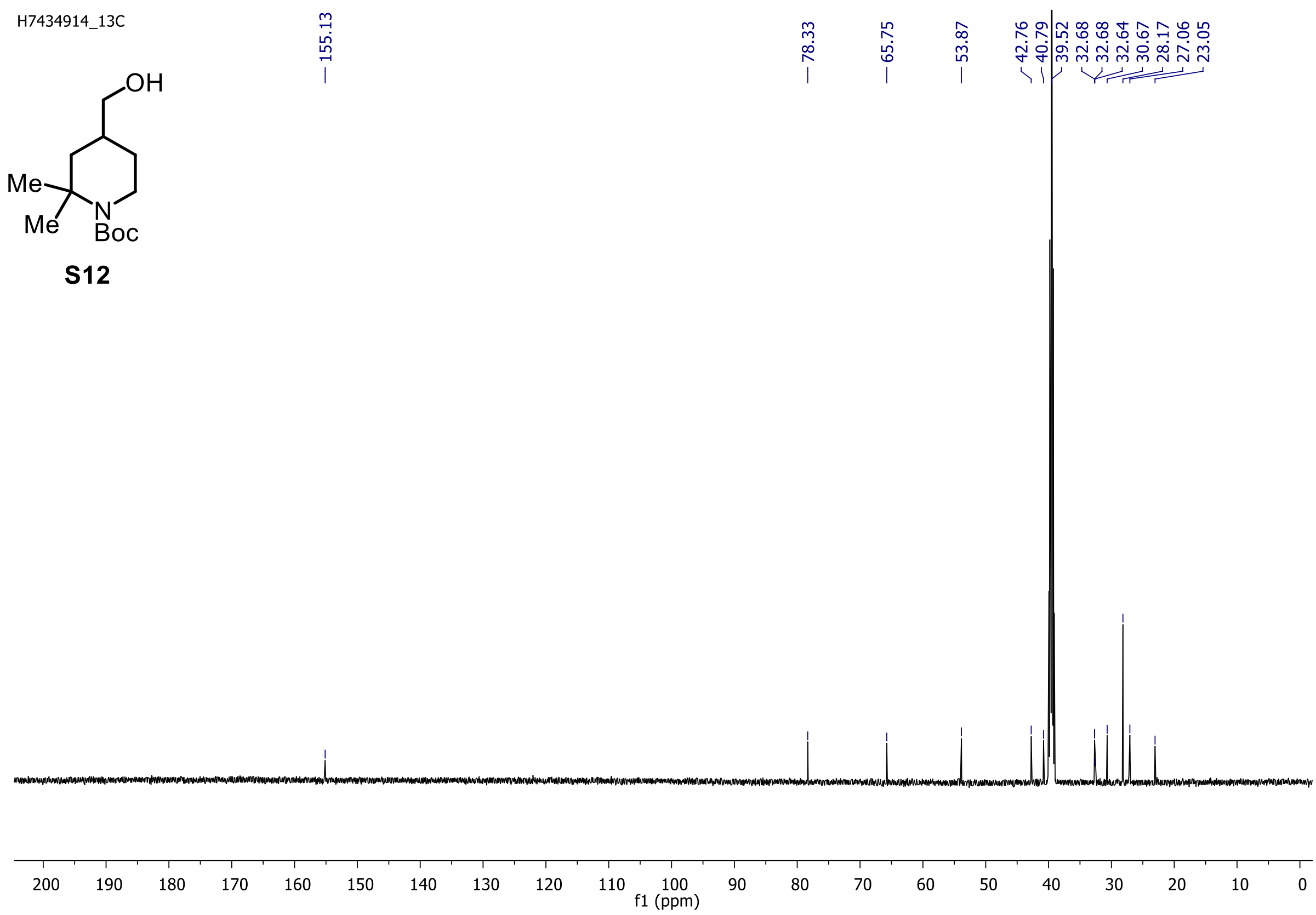

## $^1$H NMR (500 MHz, $CDCl_3$)

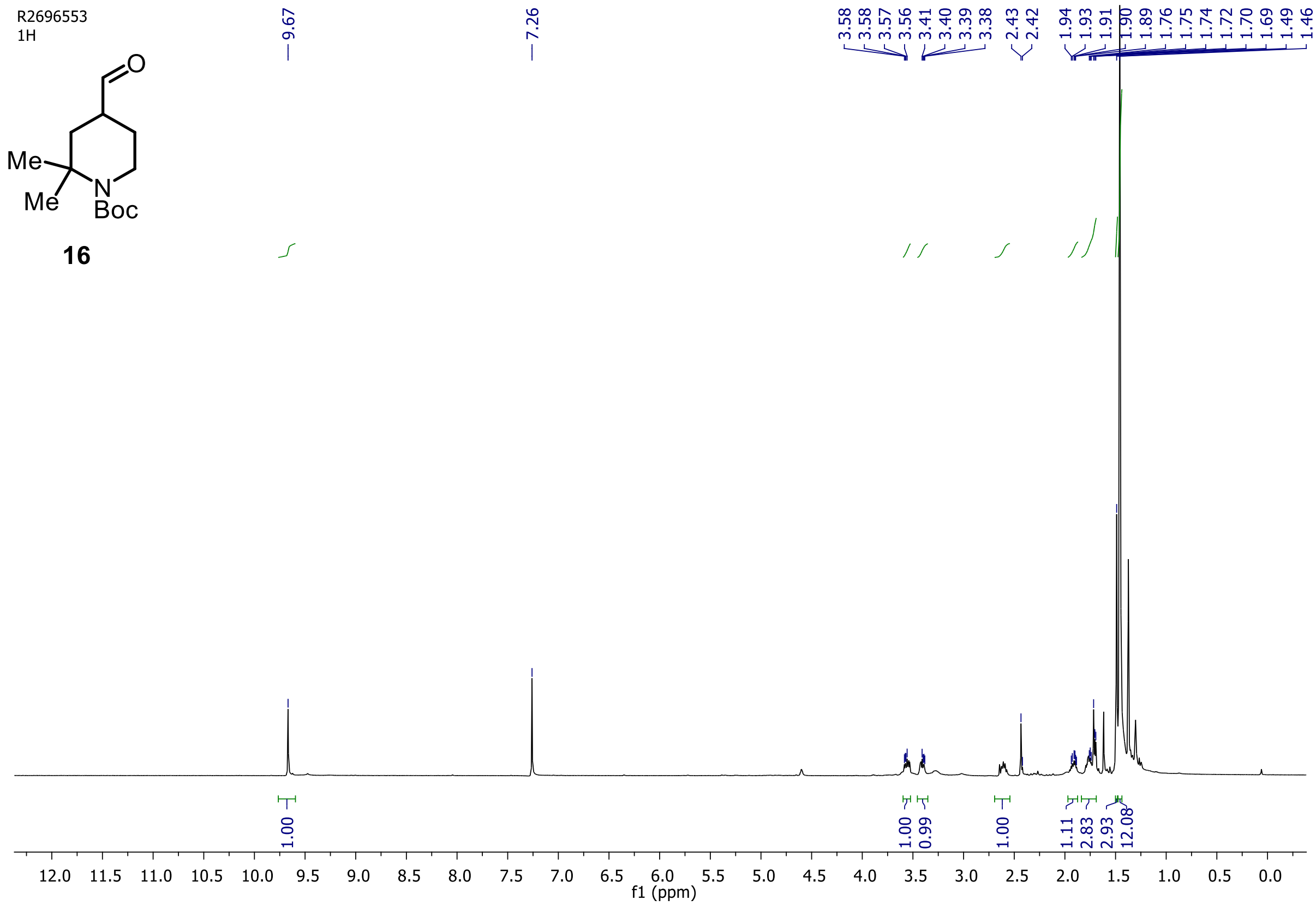


## $^{13}$C NMR (126 MHz, $CDCl_3$)

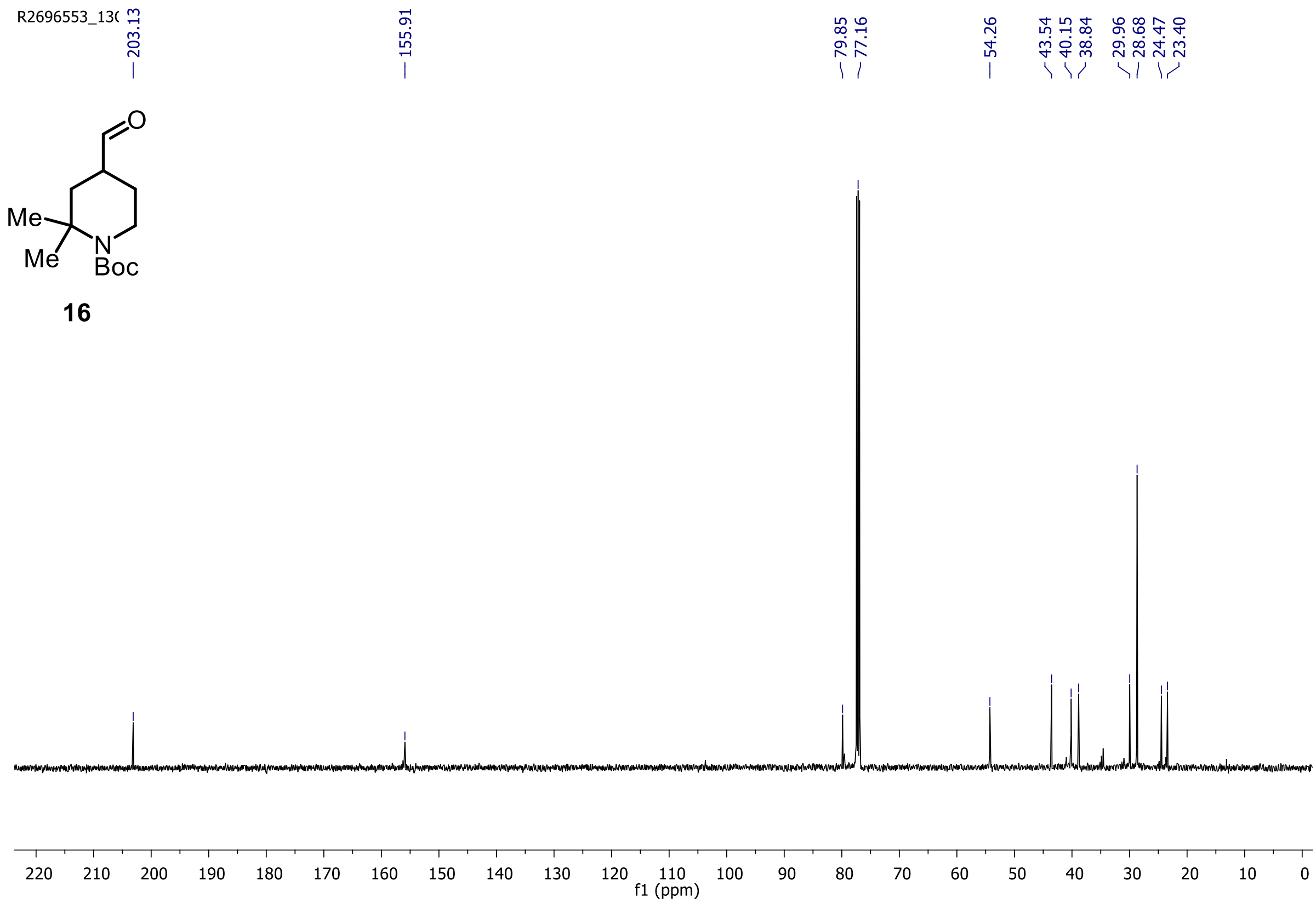

## $^{1}$H NMR (500 MHz, CDCl$_3$)

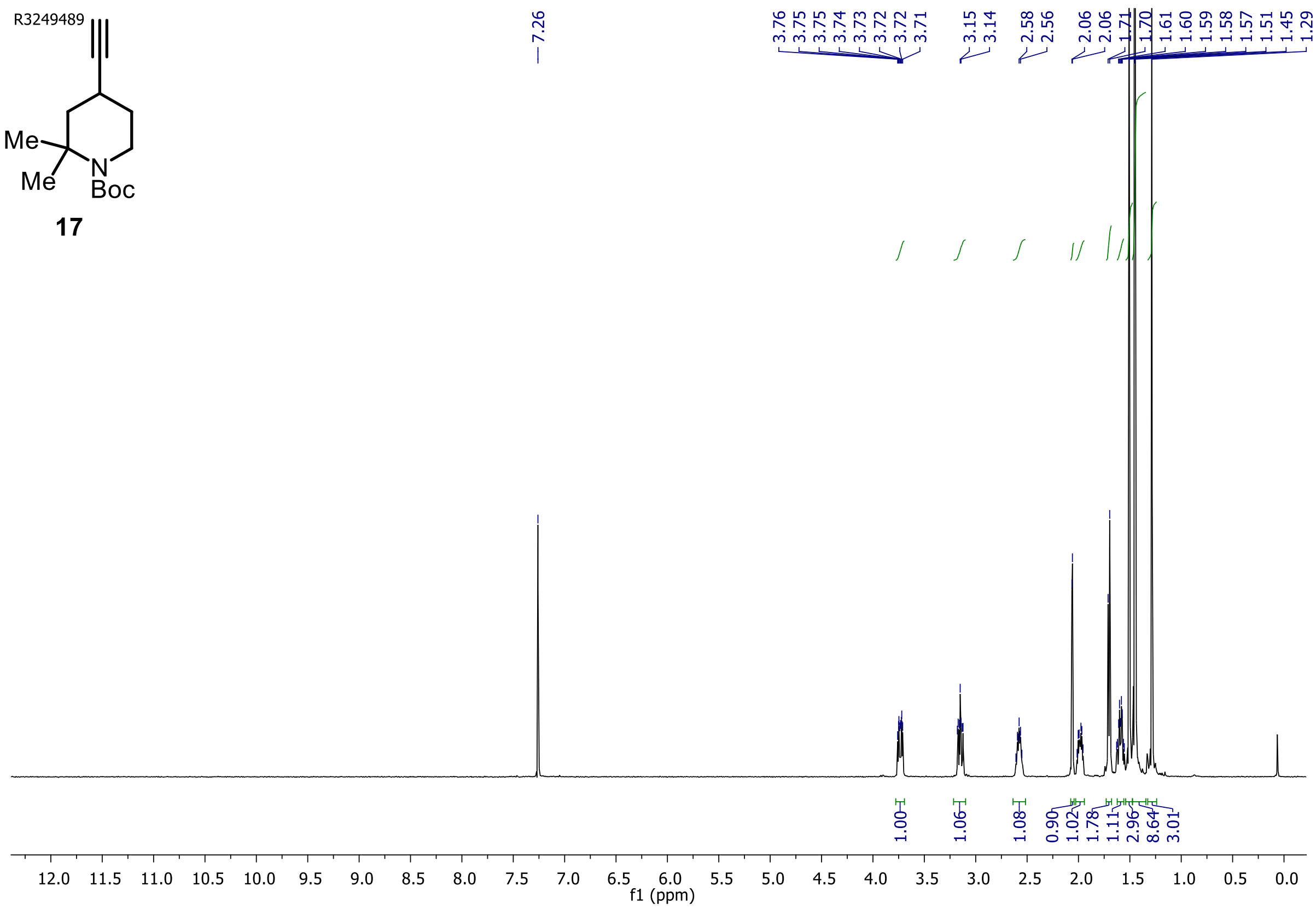

17

## $^{13}$C NMR (126 MHz, CDCl$_3$)

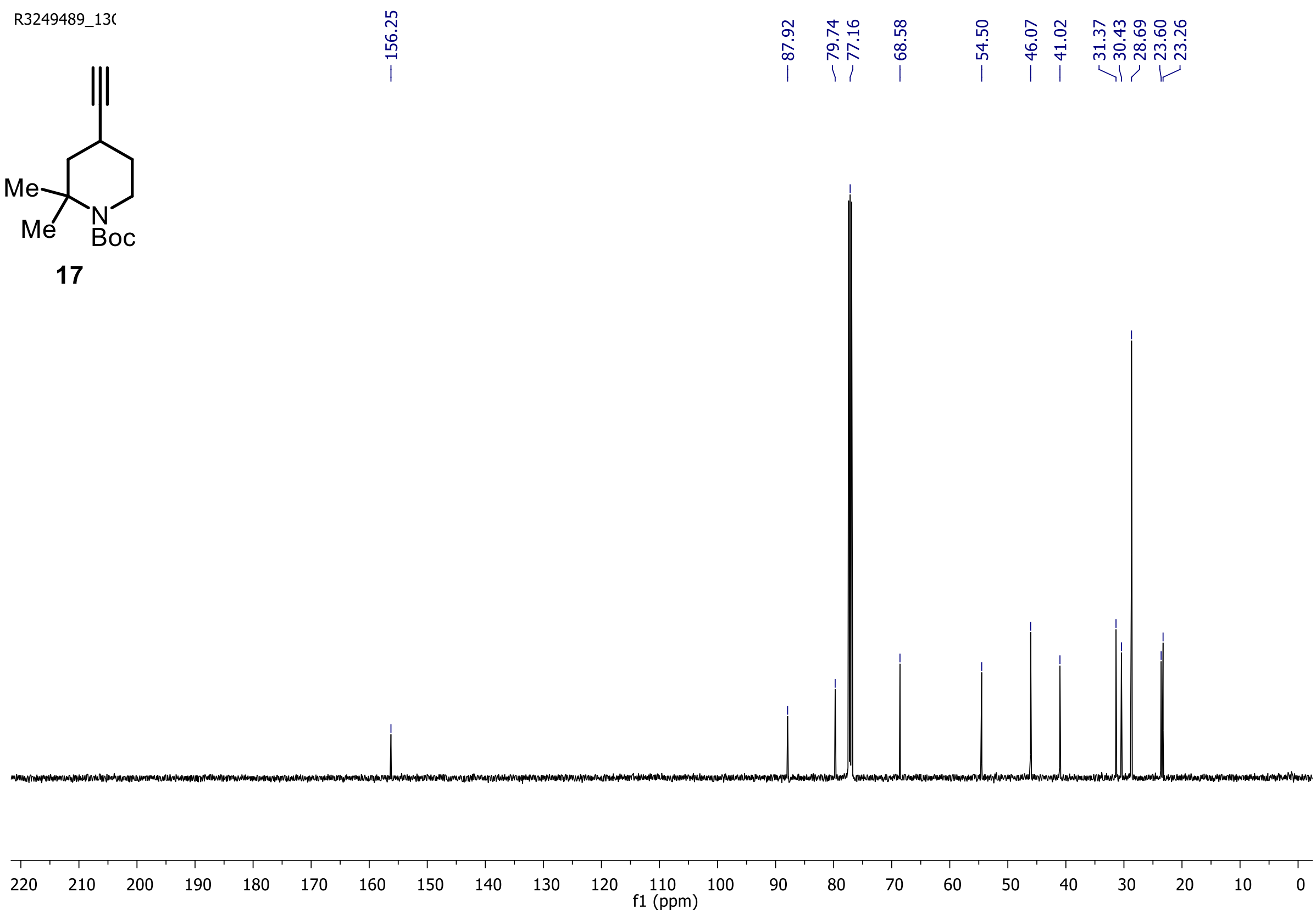

17

**$^{1}$H NMR (500 MHz, $CDCl_3$)**

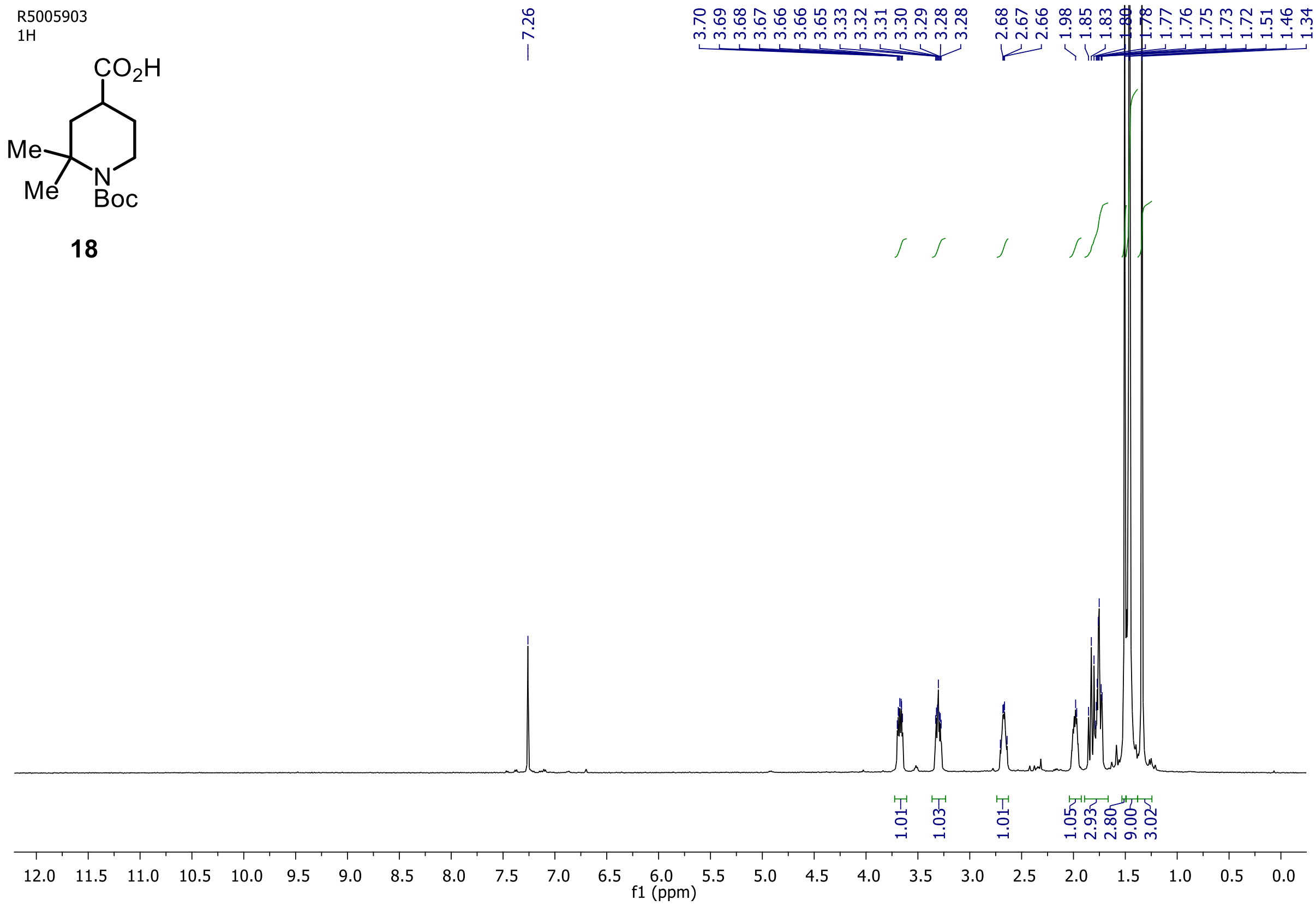


**$^{13}$C NMR (126 MHz, DMSO-$d_6$)**

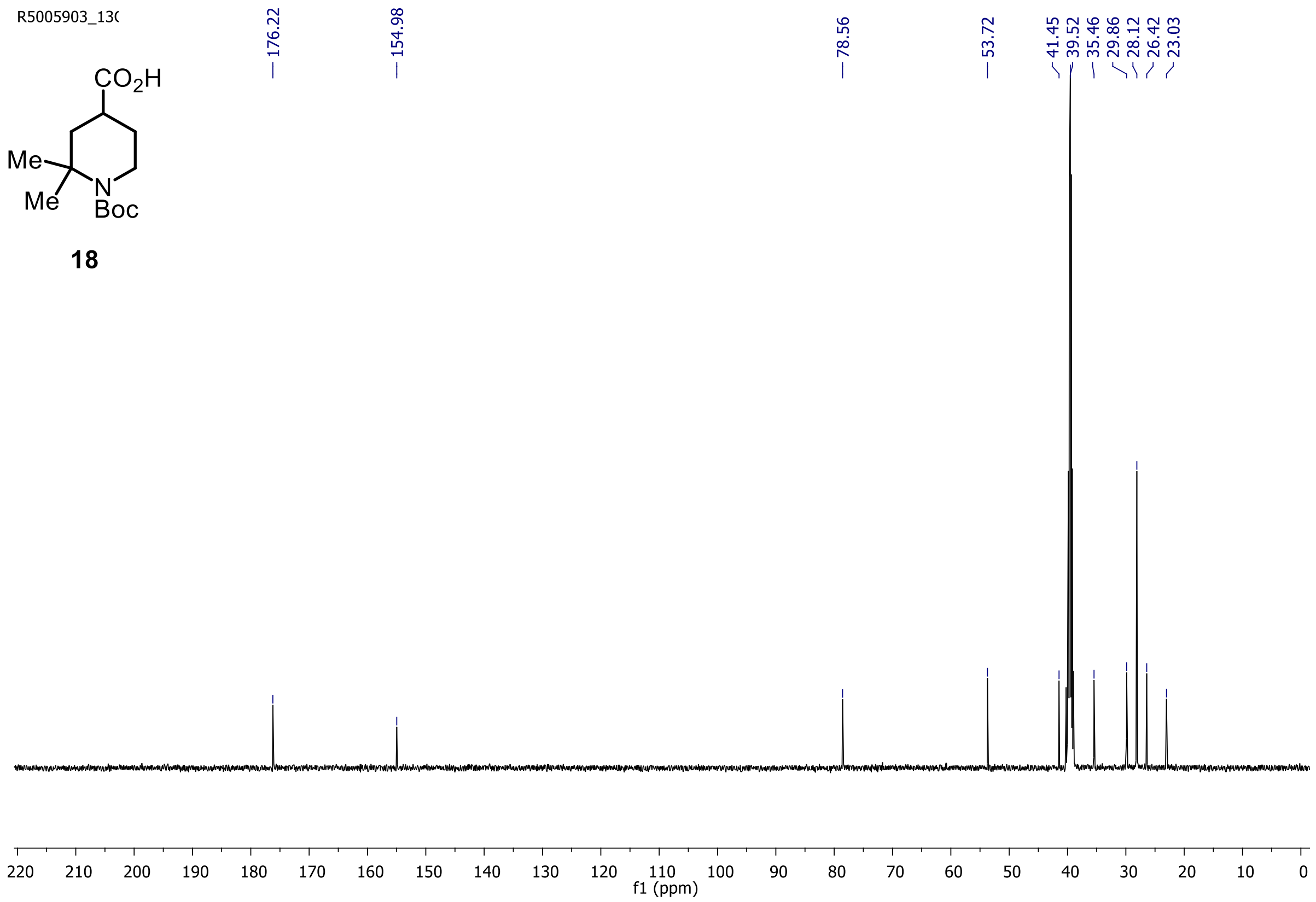

**$^{1}$H NMR (500 MHz, $D_2O$)**

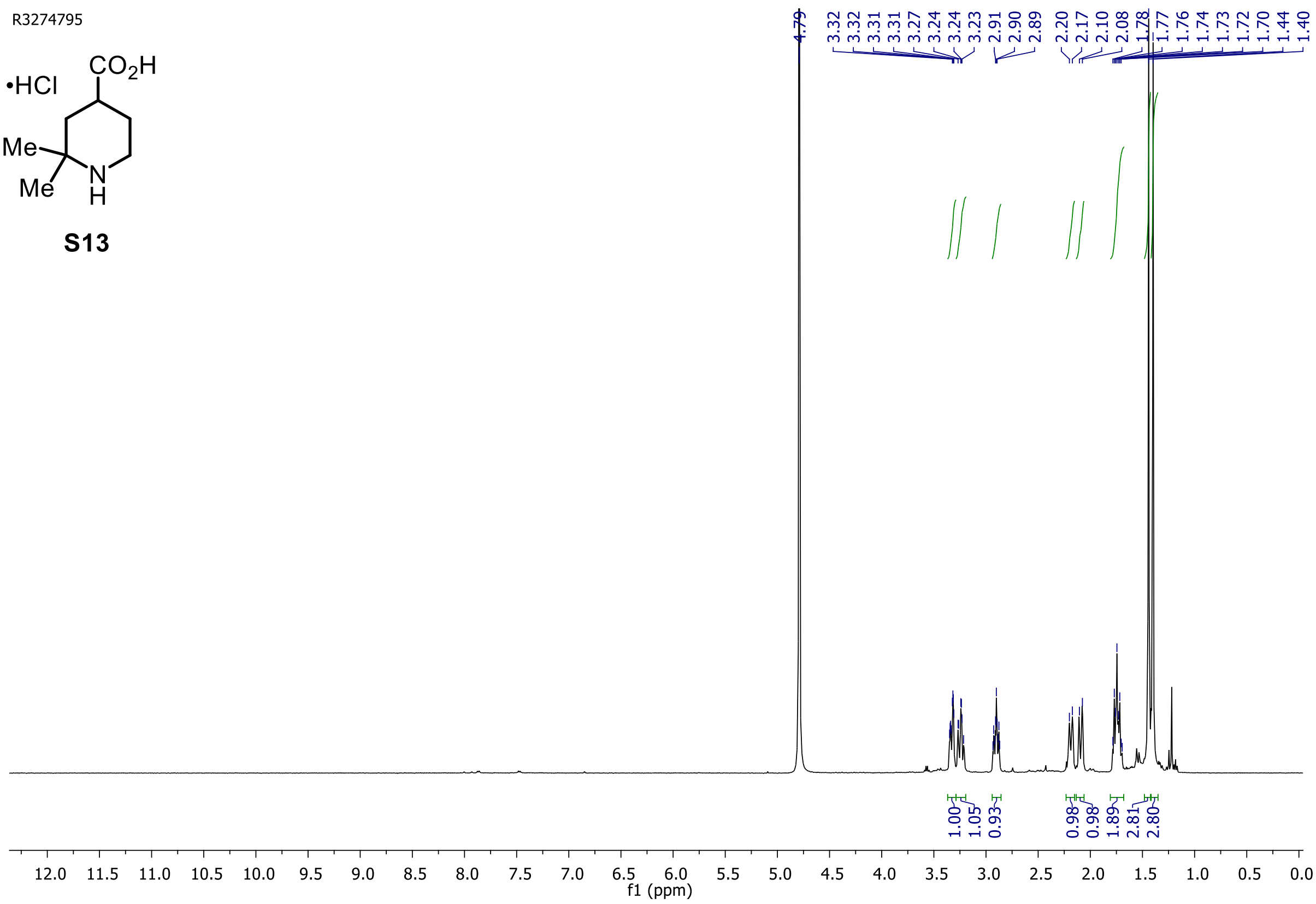


**$^{13}$C NMR (126 MHz, DMSO-*$d_6$*)**

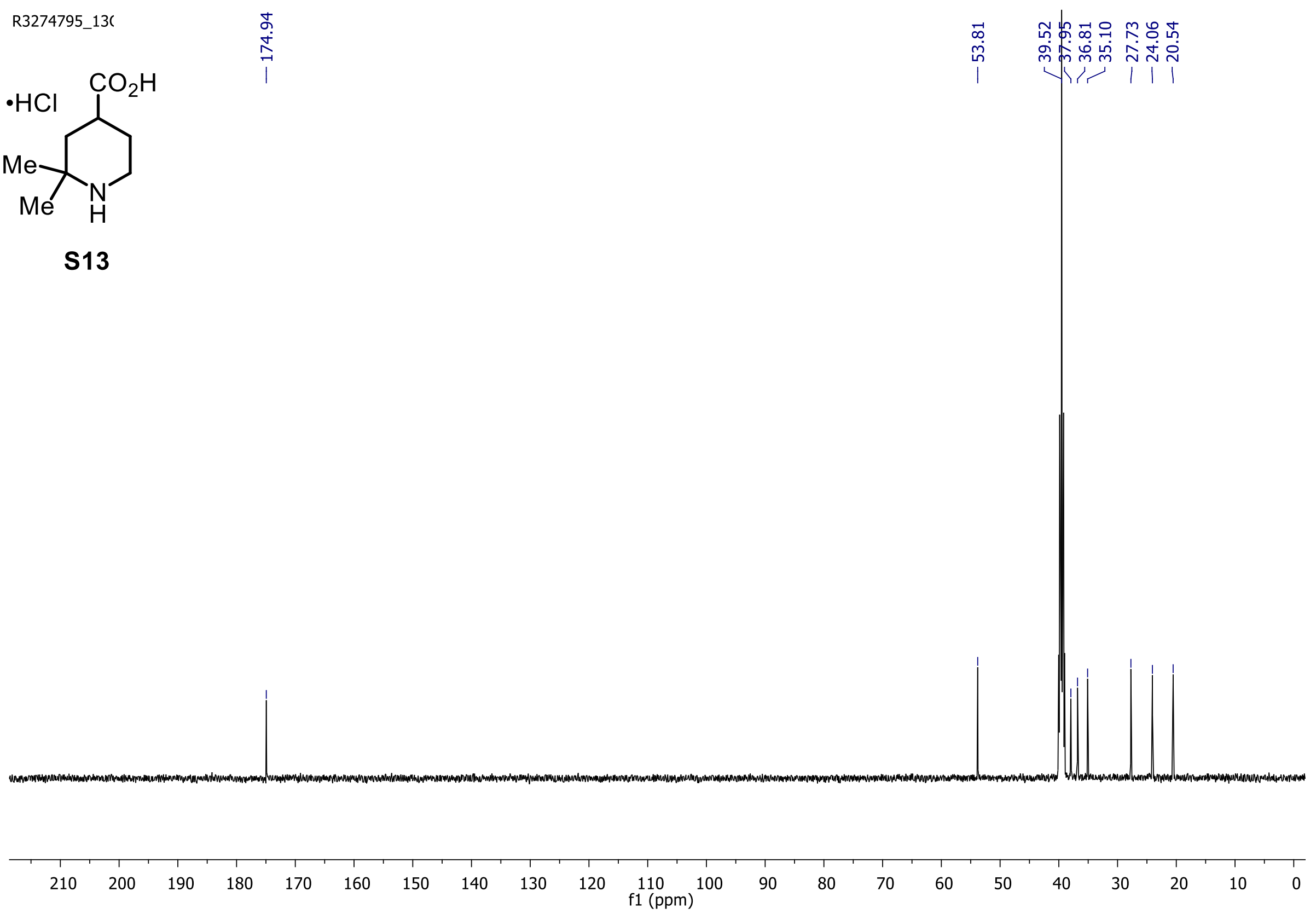

$^{1}$H NMR (500 MHz, DMSO-$d_6$)

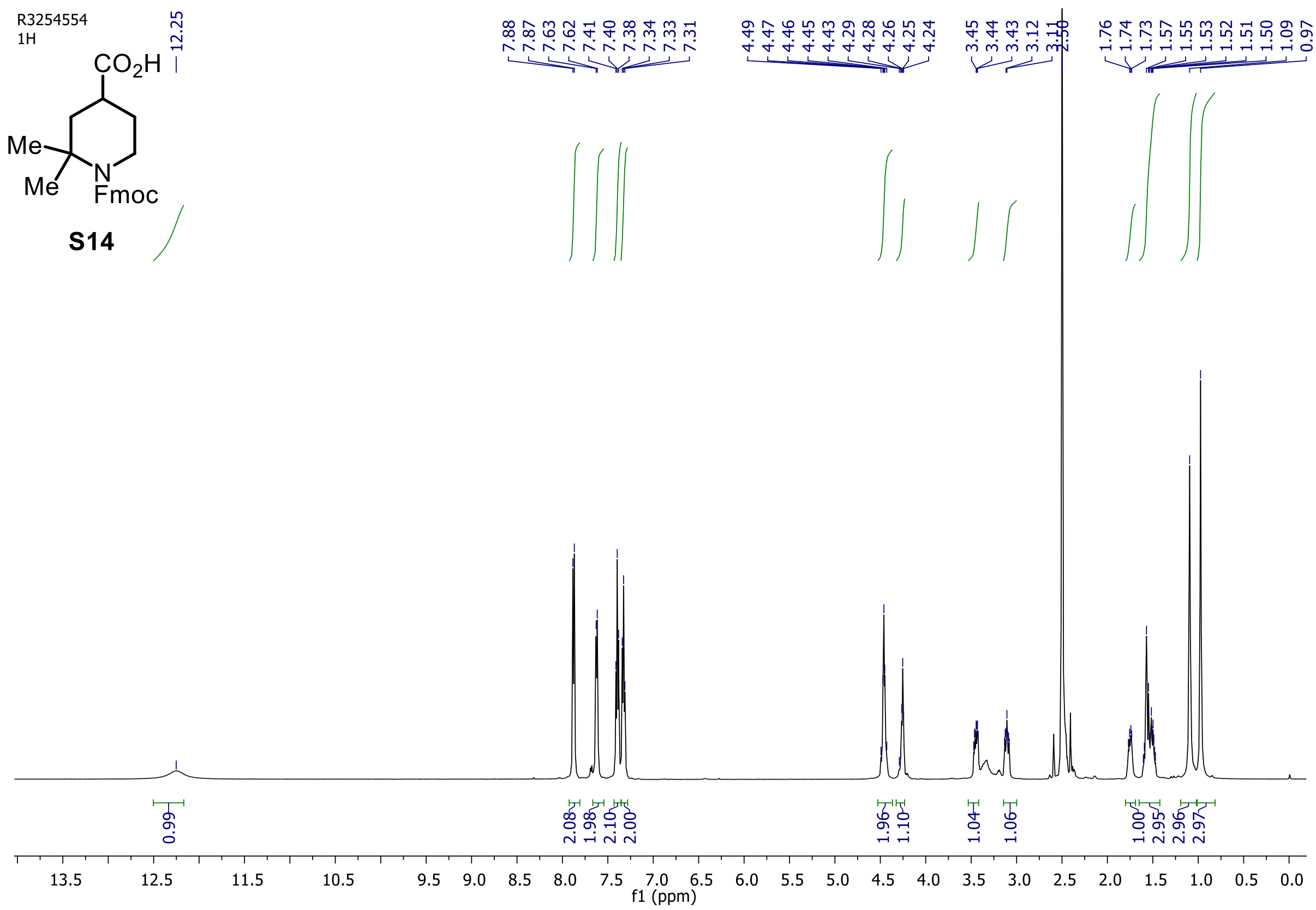


$^{13}$C NMR (101 MHz, DMSO-$d_6$)

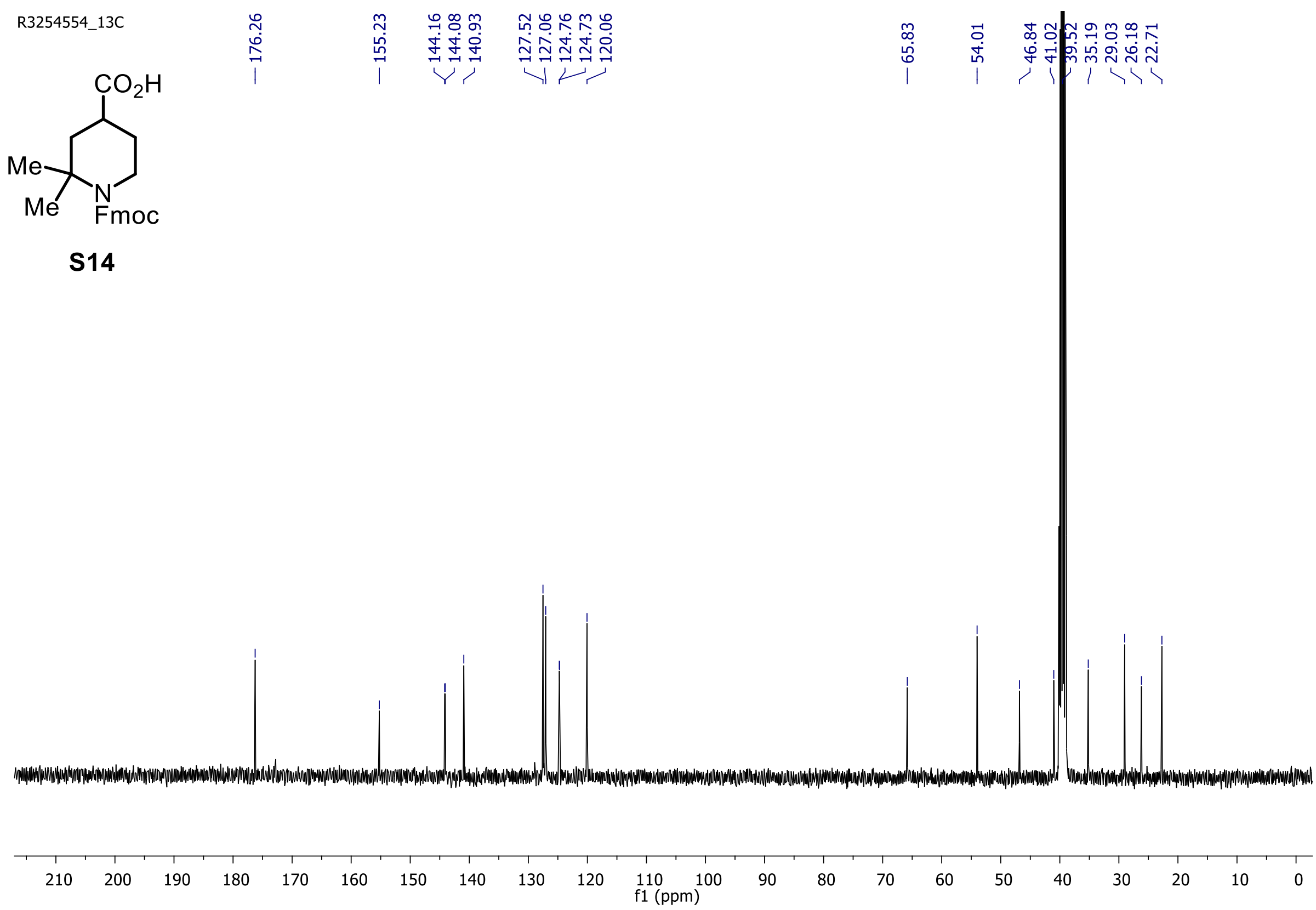

**$^{1}$H NMR (500 MHz, DMSO-$d_6$)**

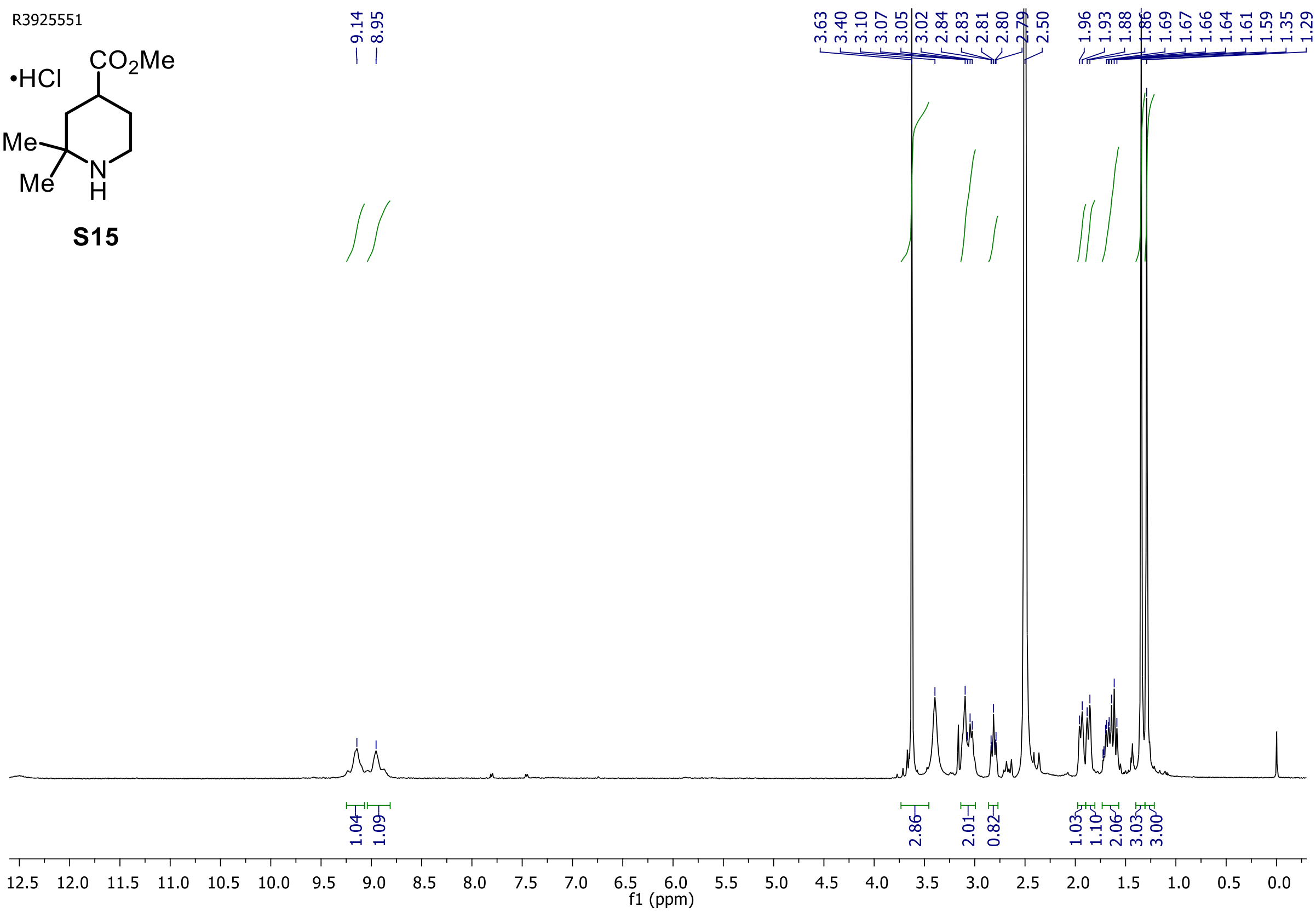


**$^{13}$C NMR (126 MHz, DMSO-$d_6$)**

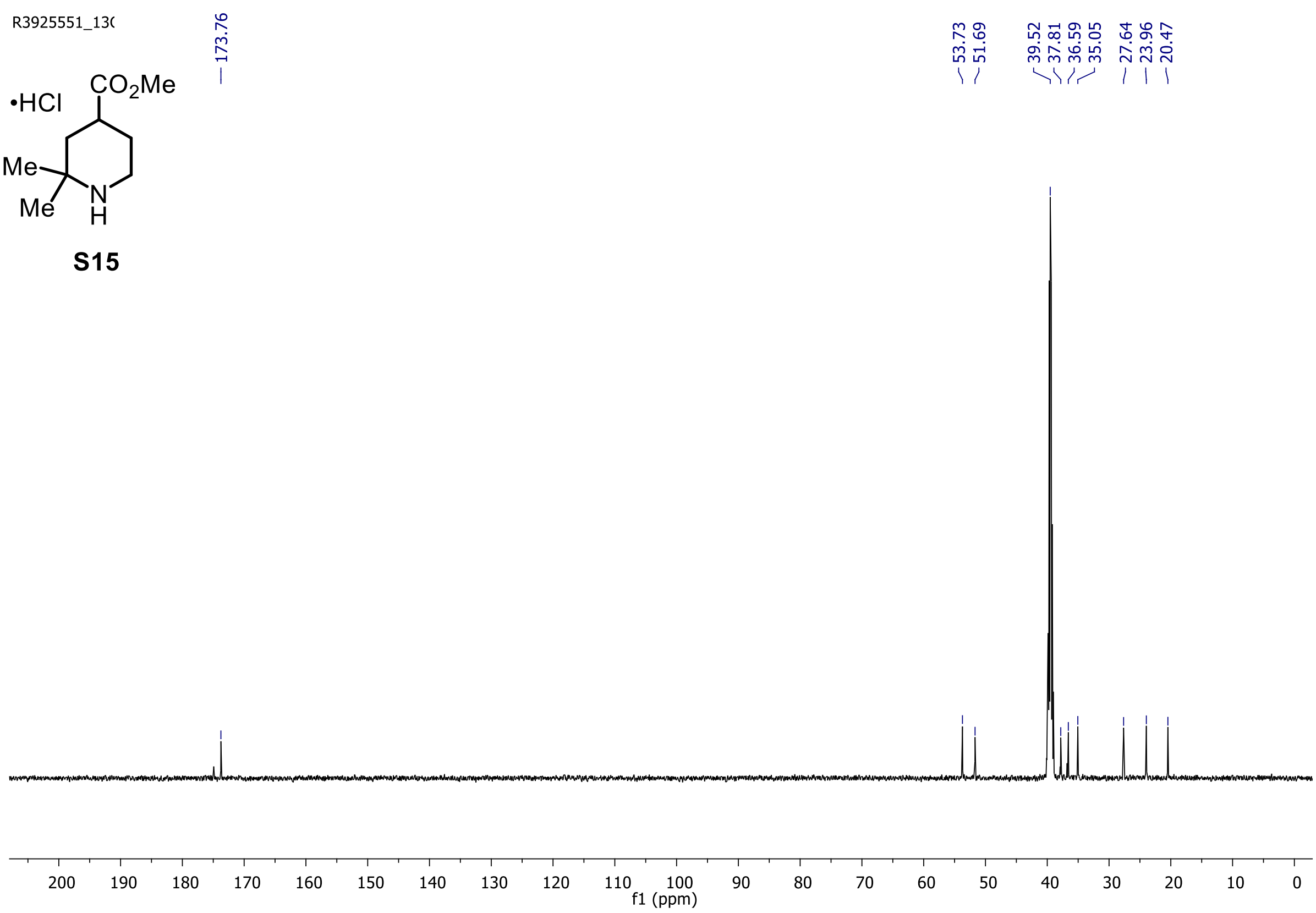

## $^{1}H$ NMR (500 MHz, $CDCl_3$)

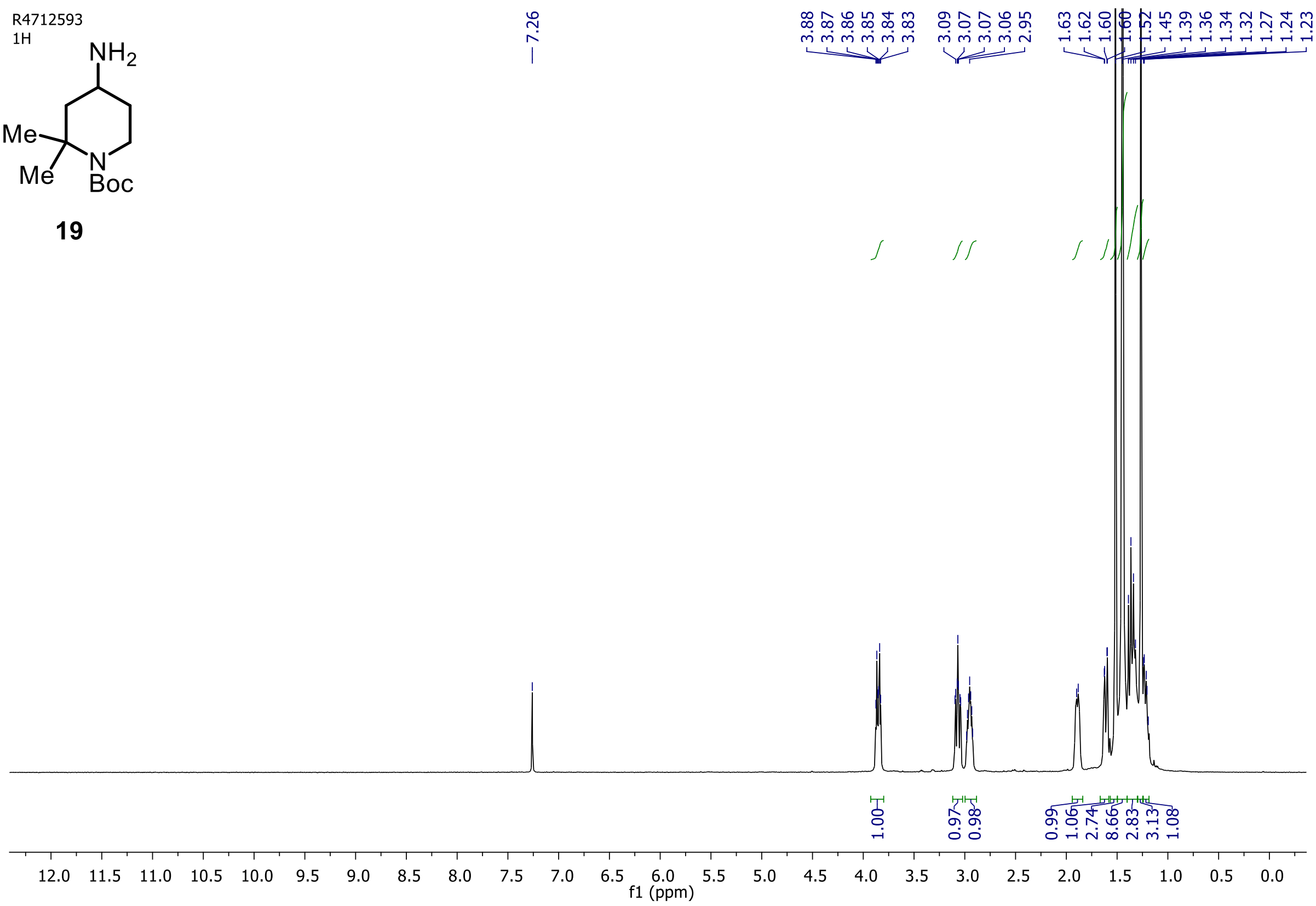


## $^{13}C$ NMR (126 MHz, $CDCl_3$)

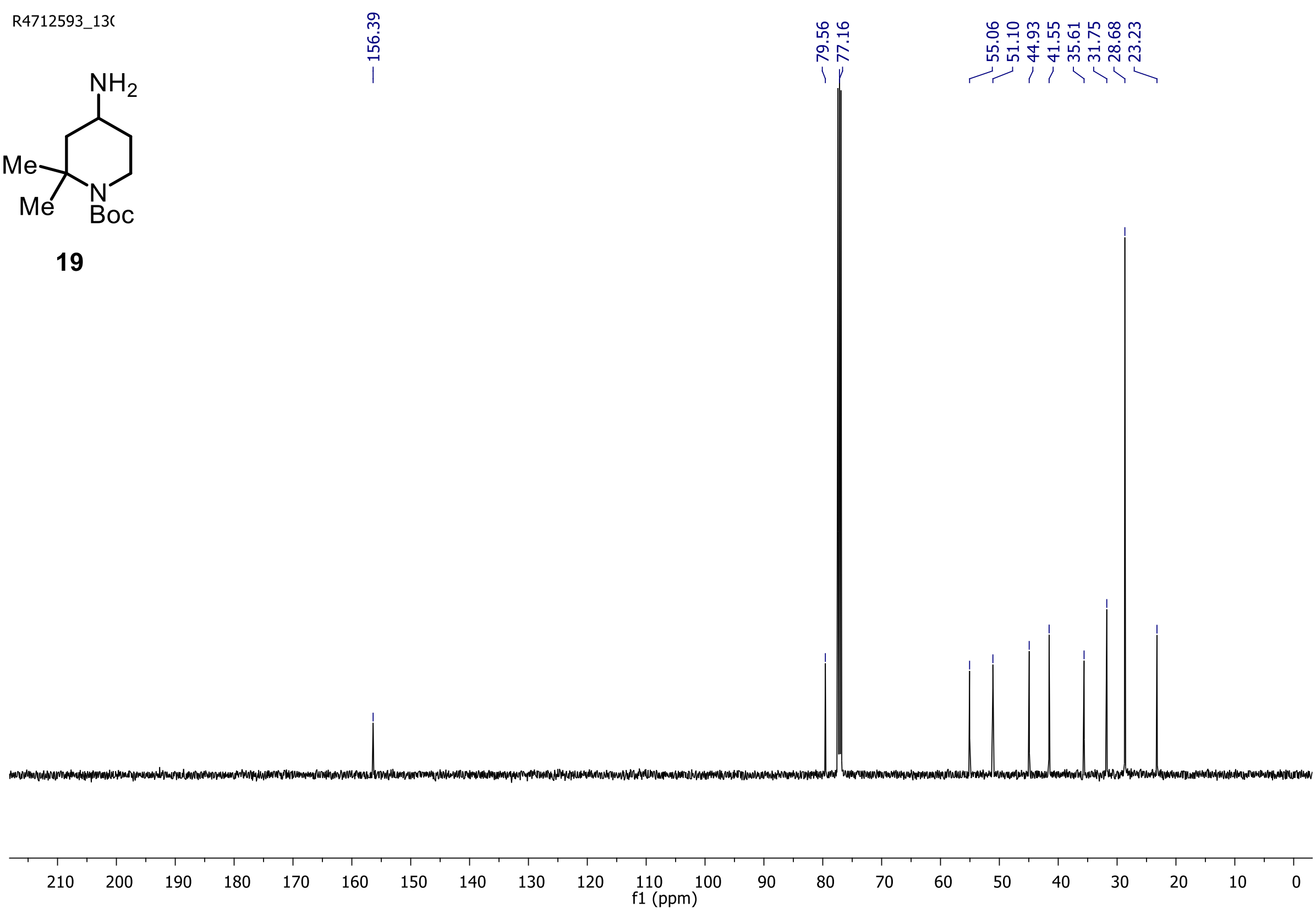

Supplement: Supplementary file 1 [file ol6c02237_si_001.pdf]
